# Development of a PCR-based assay for specific and sensitive detection of *Fusarium buharicum* from infected okra plant

**DOI:** 10.1371/journal.pone.0302256

**Published:** 2024-04-16

**Authors:** Swapan Kumar Paul, Dipali Rani Gupta, Masatoshi Ino, Makoto Ueno

**Affiliations:** 1 Laboratory of Plant Pathology, Faculty of Life and Environmental Sciences, Shimane University, Shimane, Japan; 2 Department of Agronomy, Bangladesh Agricultural University, Mymensingh, Bangladesh; 3 Institute of Biotechnology and Genetic Engineering, Bangabandhu Sheikh Mujibur Rahman Agricultural University, Gazipur, Bangladesh; University of Florida Institute of Food and Agricultural Sciences, UNITED STATES

## Abstract

Fusarium wilt, caused by the fungus *Fusarium buharicum*, is an emerging disease of okra in Japan. The disease was first reported in Japan in 2015, causing significant damage to okra seedlings. Due to the potential threat in okra cultivation, the development of an accurate detection method for *F*. *buharicum* is needed for the surveillance and management of the disease. In this study, we designed a primer set and developed conventional and nested PCR assays for the specific detection of *F*. *buharicum* in infected okra plants and contaminated soil, respectively. We compared the diversity of the translation elongation factor 1 alpha (EF-1α) gene of *F*. *buharicum* with 103 other fungal species/isolates to design a species-specific primer. This primer pair successfully amplified approximately 400 bp of PCR product that was only detected in the *F*. *buharicum* isolate, not in the other fungal isolates. The developed nested PCR method was highly sensitive and could detect the fungus from a 0.01 fg DNA sample. The primer successfully detected the pathogen in artificially infected plants and soil by conventional and nested PCR, respectively. This is the first report of the development of the *F*. *buharicum*-specific primer set and detection assays, which can be used for the specific and sensitive detection of *F*. *buharicum* in field samples and for taking early control measures.

## Introduction

Phytopathogenic fungi have experienced a rapid increase in recent decades, posing a recurring threat to stable global food production and supply [1]. Furthermore, it is hypothesized that the damages caused by diseases, particularly in vegetables, will continue to escalate. Okra (*Abelmoschus esculentus* L. Moench) stands as one of the popular vegetables extensively cultivated in temperate, tropical, and subtropical regions worldwide, including Japan [2, 3]. Nevertheless, the cultivation of okra faces a significant challenge due to the occurrence of soil-borne diseases caused by the fungal genera *Pythium*, *Rhizoctonia*, and *Fusarium* [4].

In recent years, the manifestation of damping-off/wilt symptoms in okra, induced by an emerging fungal pathogen *Fusarium buharicum*, has been reported in Okinawa, Japan [5, 6]. Interestingly, this pathogen was initially identified in cotton in Russia and kenaf in Iran [7, 8]. *F*. *buharicum* is categorized as a species complex (FBSC) due to its inclusion of numerous clonally reproducing lineages. To date, seven lineages of this species have been identified [8]. Among these, *F*. *buharicum* has been observed to be pathogenic on cotton, kenaf, and okra. *Fusarium abutilonis*, another member of the complex, infects various weedy Malvaceae plants such as *Abutilon theophrasti*, *Anoda cristata*, *Sida spinosa*, and a fabaceous host, *Senna obtusifolia* [7]. An unnamed sister species of *F*. *buharicum*, *Fusarium* sp. ex, identified in Washington state, has been linked to crown rot and vascular wilt disease in common rose mallow (*Hibiscus moscheutos*) [9]. Multilocus gene analysis of all these species strongly supports their clustering as a clade of malvaceous pathogens. Notably, the remaining four species within the FBSC are not known to be phytopathogenic [8].

Traditionally, the identification of *Fusarium* genus has relied on observing cultural characteristics and morphological traits such as macroconidia size and shape, the presence of microconidia or chlamydospores, and culture color [6, 8]. However, these methods are labor-intensive, time-consuming, and may not consistently provide accurate identification, especially at the species level [10, 11]. Molecular methods have emerged as a more efficient alternative, overcoming this limitation and offering fast, sensitive, and reliable identification of pathogens. In recent years, PCR-based technologies have proven to be powerful diagnostic tools for the detection and identification of pathogenic fungi [10, 12–14].

The development of specific primers, based on the comparison of DNA sequences from a particular gene or conserved region, is widely utilized for detecting specific pathogens in either pure or contaminated cultures of infected samples [15–19]. Additionally, nested PCR assays are employed to enhance the sensitivity of pathogen detection in environmental samples [20–24]. In a nested PCR, universal fungal primers are utilized in the first round to enrich fungal DNA against the backdrop of host genomic material. In the second round, selective amplification of the target pathogen occurs with specific primers. This approach is particularly advantageous when fungal pathogens are present in very low amounts in the host or environmental sample.

The Translation Elongation Factor 1-α (EF-1α) gene serves as a widely employed tool for identifying Fusarium species, showcasing a notable degree of sequence polymorphism among closely related species of Fusarium [8, 25–27]. Furthermore, this gene consistently appears as a single copy within the Fusarium genus, enabling more accurate quantitative comparisons between species [28]. Consequently, EF-1α has become the preferred marker, functioning as a single-locus detection tool in Fusarium. With the advancements in sequencing technology, numerous EF-1α sequences are now available in databases, streamlining sequence-based species identification processes.

While wilt disease in okra is currently confined to Okinawa, the swift pace of global warming and the movement of agricultural commodities raise concerns about its potential spread to other parts of the country, posing a looming threat to okra production. Despite this, there has been no prior investigation into the detection methods for this disease or its potential spread within Japan. In fields susceptible to new and recurrent diseases, it is imperative to develop rapid, efficient, and cost-effective methods for early detection of causative phytopathogenic fungi. These methods are crucial for controlling the disease’s spread within and across fields. This study aimed to address this gap by developing a specific primer set that leverages the uniqueness of the EF-1α gene for a PCR-based assay targeting the detection of *F*. *buharicum*. This method serves as a rapid and sensitive tool applicable to detecting the pathogen in pure culture, infected plants, and soil samples. The implementation of nested PCR, utilizing universal EF-1α gene primers, further enhances the assay’s sensitivity. This approach holds promise for pathogen detection and the effective implementation of disease management practices in okra cultivation.

## Materials and methods

### Fungal culture conditions

Potato sucrose agar (PSA) media were prepared by the methods described by [6]. All the fungal isolated used in this study were reactivated in PSA media from culture collection of plant pathology laboratory, Shimane University. Isolates were grown in PSA media for ten days at 25°C and mycelia were harvested and used for DNA extraction.

### Preparation of inoculum

Fungal inoculum was prepared by following the method described by [29] with some modification. Briefly, 5.0 g of bran, 5.0 g of bagasse, and 15 mL of dH2O (1:1:3 ratio) were taken in an Erlenmeyer flask and mixed thoroughly. The mixture was then autoclaved at 121°C for 20 minutes. The sterilized mixture of burn and bagasse were then inoculated with mycelial plugs from 3-day-old cultures of *F*. *buharicum*. The flask was then incubated at 25°C for 10–14 days with daily shaking of flask to facilitate uniform growth. The prepared inoculum was subsequently used for the inoculation of plants and soils.

### Inoculation of plant and soil

For the artificial inoculation of plants, 10-day-old okra seedlings were inoculated with 0.5–1.0 g of *F*. *buharicum* inoculum (~10^5^ CFU/g) by placing it adjacent to the collar region of the seedlings and covering it with vermiculite. Plants were then placed in a growth chamber with a 12-hour light/12-hour dark photoperiod at 25 ± 2°C with 70–75% relative humidity (RH) after inoculation. Control plants were inoculated with a mixture of bran and bagasse without *F*. *buharicum* inoculum and kept in the same environment. The development of symptoms was monitored at 24-hour intervals. For soil inoculation, 80 g of commercial soil was inoculated with 2.0 g, 1.0 g, 0.5 g, 0.1 g, 0.05 g, 0.025 g, 0.012 g, and 0 g of *F*. *buharicum* inoculum and kept in an incubator for seven days, maintaining the same environmental conditions described above. 0.5 g of each contaminated soil was used for DNA extraction.

### DNA amplification and design of primers

To design a specific primer set, we first analyzed the nucleotide sequence of EF-1α region of *F*. *bauharicum* comparing with other 103 fungal ioslates/species. The nucleotide sequences of EF-1α regions in *F*. *buharicum* and other fungus were obtained from NCBI Genbank nucleotide databases. A multiple alignment of the nucleotide sequences of EF-1α region was made by GENETYX ver. 16 (https://www.genetyx.co.jp) (S1 Fig). The sequences of *F*. *buharicum* that showed highly mismatch with other sequences were used to design specific primers. To ensure the specificity of the PCR assay, the primers underwent an initial screening against sequences in GenBank using the BLAST function to assess their potential homology to other fungi. The "Search for short, nearly exact matches" program was utilized. These primers demonstrated more than 20% mismatches with any other fungal sequences in GenBank. Under stringent PCR conditions, a >20% mismatch between the target molecule and the primer would preclude specific amplification.

### Genomic DNA extraction

The genomic DNA of fungal isolates was extracted and purified using the ISOPLANT II DAN extraction kit (Nippon Gene Co, Ltd., Tokyo, Japan). The isolation was performed according to the manufacturer’s protocol. Final concentration of each sample was adjusted to 40 ng/μL. Genomic DNA from inoculated plant was extracted by crushing the plant sample in liquid nitrogen using the same protocol. The DNA of artificially inoculated commercial soil samples was extracted by using the ISOIL for Beads Beating kit (Nippon Gene Co., LTD. Japan). Approximately, 0.5g of soil sample was homogenized in a 1.5 ml microtube in a bead beater (Tomy, Micro Smash MS-100, GeneCrafts, Japan) at 4500 rpm for 45 seconds. Rest of the procedures were followed as described by the manufacturer. The isolated DNA was quantified using a nano drop spectrophotometer (NanoDrop 1000, Thermo Scientific, USA).

### DNA amplification

To test the specificity of the primers, the PCR reaction was carried out in a volume of 10 μL PCR mixtures consisted of 1.7 μL autoclaved dH_2_O, 5 μL 2× polymerase buffer, 2 μL of dNTPs, 0.2 μL KOD FX Taq polymerase, 0.3 μL of 10μM of each primer combinations (EF1-Fb- F_1_ /EF1-Fb-R_5_) and 0.5 μL of DNA template. The PCR conditions were optimized and comprised an initial denaturation 94°C for 2 min, followed by 30 cycles of 98°C for 10 s, 56°C for 30 s, and 68°C for 30 s with a final extension at 72°C for 10 min. Fungal genomic DNA was amplified for the nuclear ribosomal internal transcribed spacer (ITS) region with ITS1/ ITS4 primer pair [30].

### Specificity and sensitivity test

The specificity of the newly designated primers for the detection of *F*. *buharicum* was determined by using the genomic DNAs isolated from *F*. *buharicum* and 20 different fungal species (Table 1). The sensitivity of the conventional PCR for the detection of *F*. *buharicum* was determined by using the different concentrations of genomic DNA (40 ng, 20 ng, 10 ng, 5 ng, 1 ng, 0.1 ng and .01 ng/μl) as template.

**Table 1:** List of fungal isolates used in this study.

Sl no.	Fungal isolates used
1	*Fusarium buharicum* OKI LC727425.1
2	*F*. *nirenbergiae* (*F*. *oxysporum* Schlechtendal f. sp. *batatas*) MAFF103070
3	*F*. *cugenangense* (*F*. *oxysporum* f. sp. *spinaciae*)
4	*F*. *languescens* (*F*. *oxysporum* f. sp. *lycopersici)* MAFF744006
5	*F*. *nirenbergiae* (*F*. *oxysporum* f. sp. *lycopersici*) MAFF305914
6	*F*. *languescens* (*F*. *oxysporum* f. sp. *lycopersici*) MAFF103044
7	*Athelia rolfsii*
8	*Sclerotinia sclerotiorum*
9	*Pythium aphanidermatum*
10	*Stagonosporopsis cucurbitacearum* MAFF744016
11	*Colletotricum orbicularae*
12	*Pyricularia oryzae*
13	*Alternaria Alternata pear*
14	*Botrytis cineria*
15	*Stemphylium lycopersici* MAFF150020
16	*Ceratocystis fimbriata* MAFF237660
17	*Monosporascus cannonballus* MAFF305581
18	*Corynespora cassiicola*
19	*Cylindrocarpon destructans*
20	*Cochliobolus miyabeanus*

### Nested PCR assay

For nested PCR assay, first round of amplifications were carried out using the EF-1α universal primers (EF1-F; 5’-ATGGGTAAGGARGACAAGAC-3’ and EF1-R; 5’-GGARGTACCAGTSATCATGTT-3’) from the DNA samples extracted from fungal culture (1.0 ng–0.01fg)) or infected soils. To amplify the EF-1α region of *F*. *buharicum*, PCR amplification was performed in a total volume of 50 μL containing 10 μL autoclaved dH_2_O, 25 μl 2× polymerase buffer, 10 μL of dNTPs, 1.0 μL KOD FX Taq polymerase, 1.5 μL of 10μM of each primer combinations (EF1- F/ EF1- R) and 1.0 μL of DNA template. The PCR conditions for this amplification were comprise an initial denaturation at 95°C for 3 min, followed by 30 cycles of 98°C for 10 s, 55°C for 30 s and 68°C for 1.45 min. followed by a final extension of 68°C for 10 min and run in a thermocycler (GeneAtlas G02; Astec Co., Ltd., Fukuoka, Japan). In the second round PCR, a 0.5 μl aliquot of the first round PCR product was as template with the *F*. *buharicum* specific primers the EF1-Fb-F_1_ / EF1-Fb-R_5_. The PCR condition was same as described above except the PCR cycle in second round PCR was decreased to 25 cycles. After addition of 1 μL gel-loading buffer with 5 μl, the PCR products were analyzed by electrophoresis on 1% agarose gel and visualized under UV light using a gel documentation system.

## Results

### Primer design and specificity test

**Fig 1:**
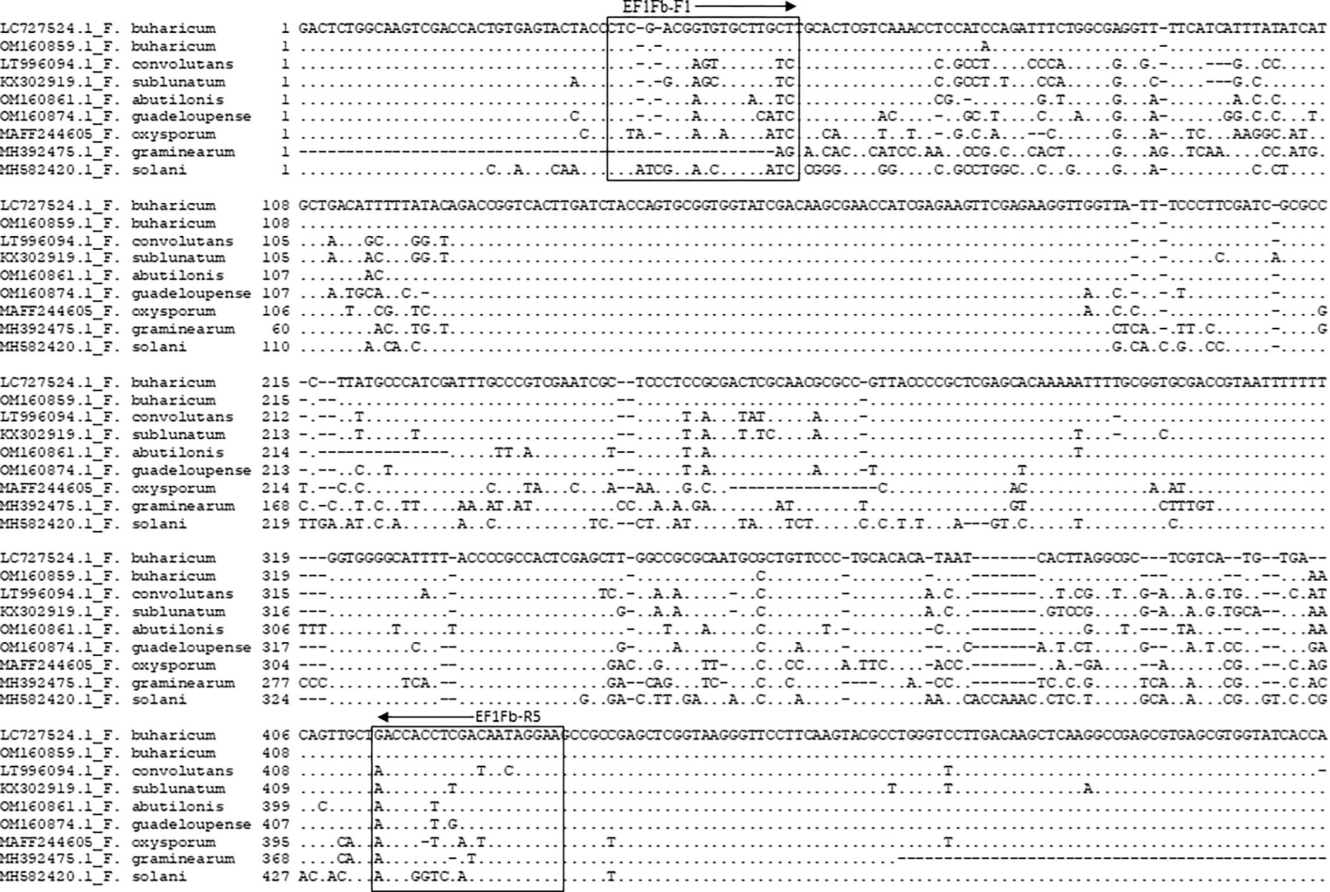
Design of specific primers for *F*. *buharicum*. Partial sequences of EF-1α gene regions of selected fungal species were aligned along with *F*. *buharicum* and the sequences of *F*. *buharicum* that showed highly mismatch with other sequences were used to design specific primers using the GENETYX ver. 16 software. The location of the *F*. *buharicum* species-specific primers (EF1Fb-F1 and EF1Fb-R5) sequences are indicated in red boxes.

For the development of specific primers, multiple sequences of the EF-1α region from various *Fusarium* species were aligned to identify sequence elements unique to *F*. *buharicum*. Approximately 650 bp of the EF-1α region was analyzed, leading to the design of two primers; one forward (EF1-Fb-F1) and one reverse (EF1-Fb-R5)-based on nucleotide variations specific to the *F*. *buharicum* isolate. The position of the primer pair is illustrated in Fig 1. Subsequently, the primer pair was employed to amplify the EF-1α region from *F*. *buharicum*.

**Fig 2:**
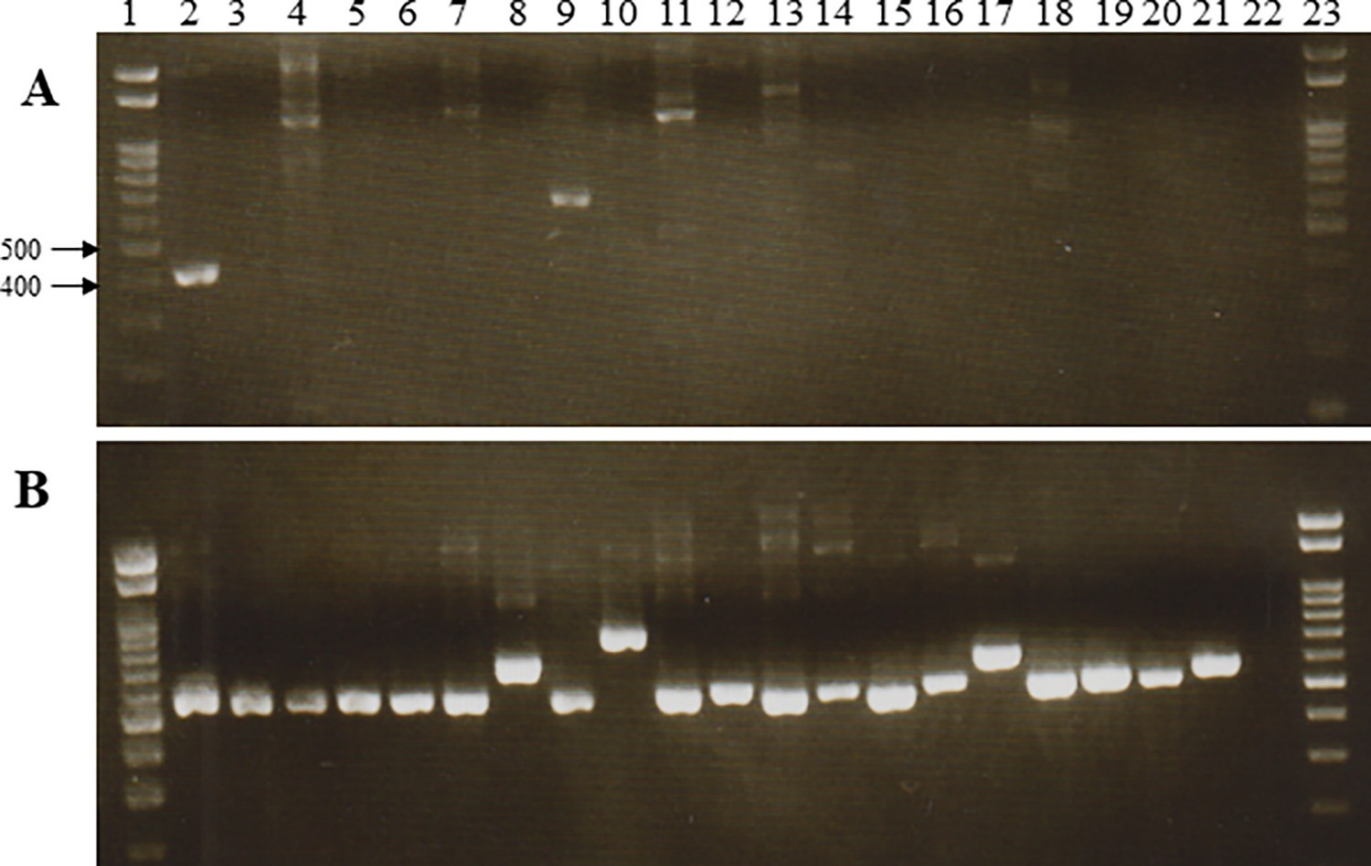
PCR for the detection of *F*. *buharicum* with EF1Fb-F1/EF1Fb-R5 primers. (**A**) EF-1α and (**B**) ITS regions from *F*. *buharicum* and the other fungal species. Lane 1: DNA marker, lane 2: *F*. *buharicum*, lane 3: *F*. *nirenbergiae (*MAFF103070), lane 4: *F*. *cugenangense*, lane 5: *F*. *languescens (*MAFF744006*)*, lane 6: *F*. *nirenbergiae* (MAFF305914), lane 7: *F*. *languescens (*MAFF103044), lane 8: *Athelia rolfsii*, lane 9: *Sclerotinia sclerotiorum*, lane 10: *Pythium aphanidermatum*, lane 11: *Stagonosporopsis cucurbitacearum*, lane 12: *Colletotricum orbicularae*, lane 13: *Pyricularia oryzae*, lane 14: *Alternaria Alternata*, lane 15: *Botrytis cineria*, lane 16: *Stemphylium lycopersici*, lane 17: *Ceratocystis fimbriata*, lane 18: *Monosporascus cannonballus*, lane 19: *Corynespora cassiicola*, lane 20: *Cylindrocarpon destructans*, lane 21: *Cochliobolus miyabeanus*, lane 22: negative control, lane 23: DNA marker.

To assess the specificity of the newly designated primers, purified DNA (~40 ng) from one *F*. *buharicum* isolate, five isolates of other *Fusarium* species, and 14 isolates of other plant pathogenic fungal species were subjected to amplification with the designated primer pairs. The primer pairs generated an approximately 400 bp amplicon only for the *F*. *buharicum* isolates, not for other fungal isolates (Fig 2A). Although, some other fungal isolates DNA yielded non-specific band during PCR but that is not in the expected size (400 bp). However, no nonspecific bands were observed in the amplified product using the primer pairs. Thus, PCR with species-specific primers enabled the detection of *F*. *buharicum* at the species level.

### Sensitivity of the primers

**Fig 3:**
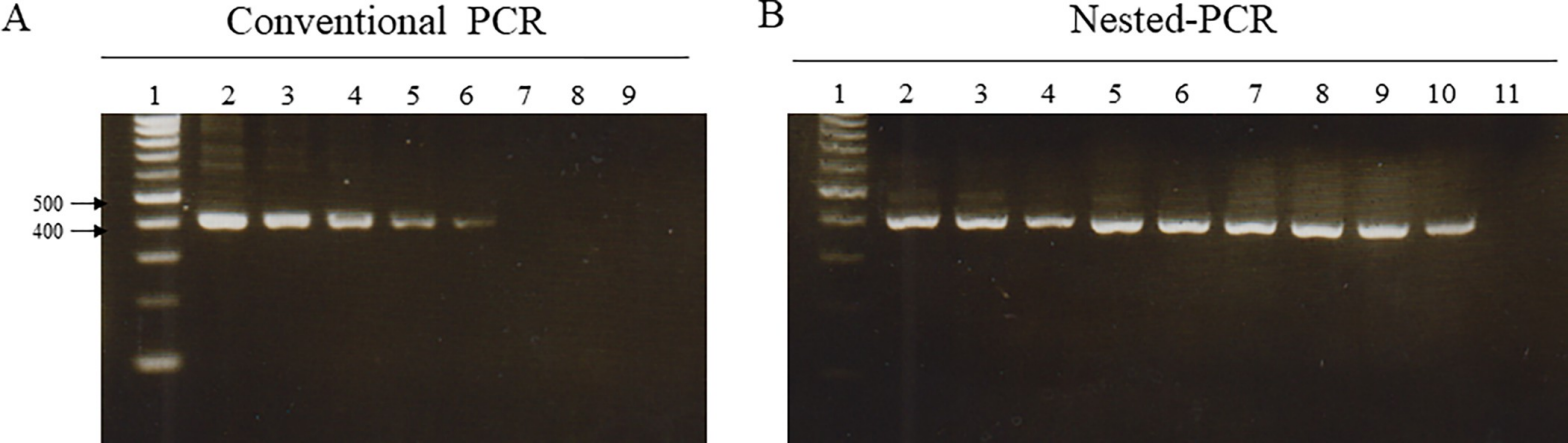
Sensitivity of the primers EF1-Fb-F1/EF1-Fb-R5 for the detection of *F*. *buharicum*. (**A)** Conventional PCR [Lane 1: DNA ladder, lane 2–8: template DNA concentrations (40ng, 20ng, 10ng, 5ng, 1ng, 0.1ng, 0.01ng, respectively) lane 9: negative control]. **(B)** Nested PCR [Lane 1: DNA ladder, lane 2–10: template DNA concentrations (1.0ng, 100 pg, 10 pg, 1pg, 100 fg, 10 fg, 1 fg 0.1fg, 0.01 fg, respectively); lane 11: negative control].

For sensitivity testing of the newly designed primer pairs, a serial dilution of fungal DNA from *F*. *buharicum*, ranging from 40 ng to 0.001 ng, was utilized. In a PCR assay, the specific primer pair successfully amplified the expected size of the DNA fragment up to 1.0 ng of DNA template. The intensity of the amplified band diminished as the DNA concentration decreased and became undetectable at 0.1 ng of DNA concentration (Fig 3A). Therefore, the detection limit of the primer was determined to be 1.0 ng for the conventional PCR assay. To enhance the sensitivity of the primers, we conducted a nested PCR assay for pathogen detection. The developed nested PCR assay proved highly effective in detecting F. *buharicum* and could identify the pathogen from at least 0.01 fg of DNA template (Fig 3B).

### Detection of *F*. *buharicum* in infected plants

**Fig 4:**
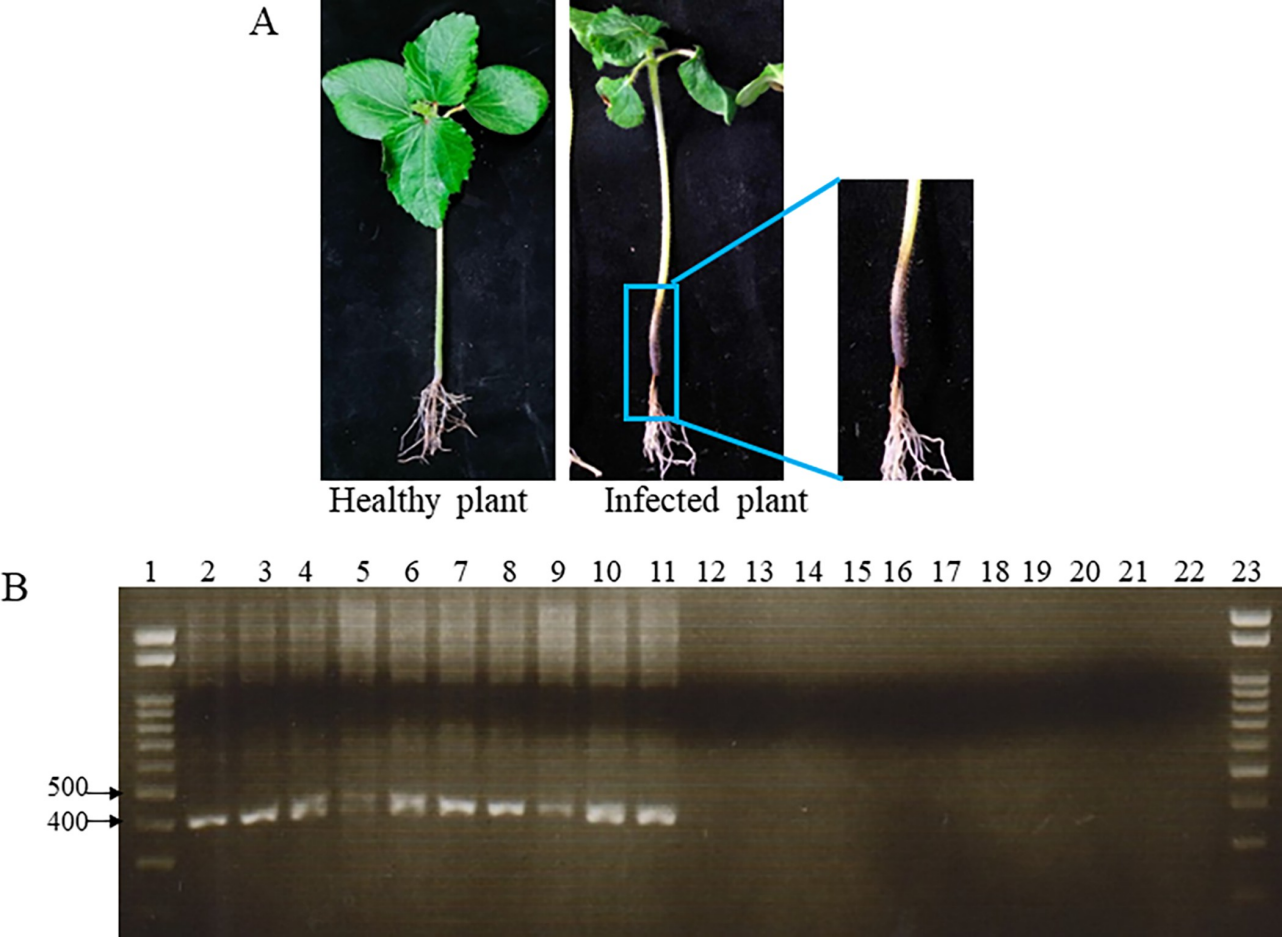
Detection of *F*. *buharicum* in infected okra plant. **(A)** Development of disease symptoms on okra plant infected by *F*. *buharicum*. **(B)** Conventional PCR assay using EF1-Fb-F1/EF1-Fb-R5 primer pair as diagnostic test for the detection of *F*. *buharicum* in the infected okra cultivars. Lane 1: DNA ladder; lane 2–11: artificially infected okra cultivars (Early five, Green Sword, Green Star, Herushie, Shima Okura, Hachijyojima Okura, Shirohisui, Emerald, Benny, Hoshihime, respectively); lane 12–21: healthy plants as control (Early Five, Green Sword, Green Star, Herushie, Shima Okura, Hachijyojima Okura, Shirohisui, Emerald, Benny, Hoshihime, respectively); lane 22: negative control; Lane 23: DNA marker.

To assess the ability of the PCR assay in detecting the fungus in infected plants, we inoculated plants with *F*. *buharicum* isolate OKI-1. The infected plants exhibited brown discoloration at the main stem of the okra (Fig 4A). Sections of these infected plants were utilized for DNA extraction, serving as templates for conventional PCR. The primer pair EF1-Fb-F1/EF1-Fb-R5 successfully generated bands of the expected size from 10 varieties of okra plants infected with F. *buharicum*, while no bands were observed in samples obtained from healthy plants (Fig 4B). Therefore, the developed assay demonstrated its capability to detect the pathogen in infected okra plants.

### Detection of *F*. *buharicum* in inoculated soil

**Fig 5:**
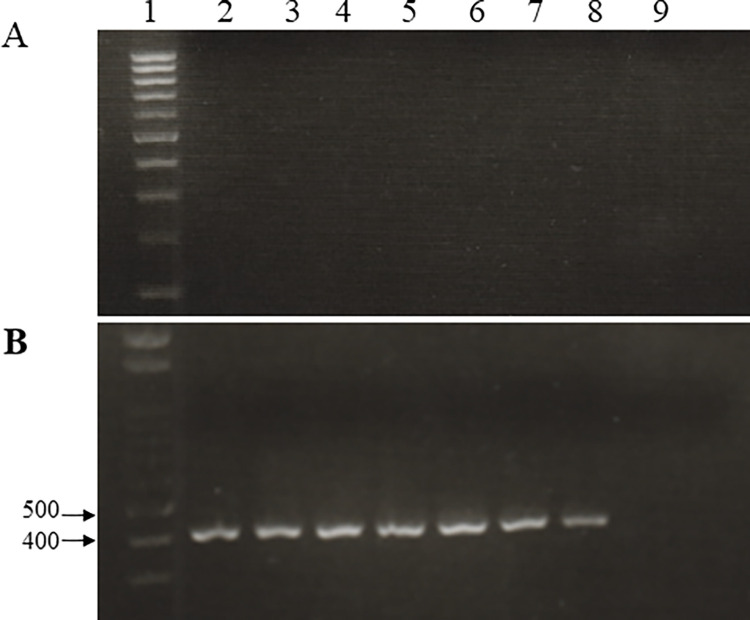
The nested PCR assay for the detection of *F*. *buharicum* in the artificially contaminated soil samples. **(A)** Conventional PCR and **(B)** nested PCR assay were done using the genomic DNA extracted from artificially contaminated soil sample with the designed primers EF1-Fb-F1/EF1-Fb-R5 [Lane 1: DNA ladder; lane 2–8: contaminated soil samples (concentrations of inoculum; 2, 1, 0.5, 0.1, 0.05, 0.025, 0.012 g inoculum/80 g soil, respectively), lane 9: control (uninoculated soil sample)].

In our attempt to detect *F*. *buharicum* in infected soil samples, we initially conducted conventional PCR using DNA isolated from artificially contaminated soil. However, no visible bands were observed in any of the DNA samples obtained from the contaminated soil (Fig 5A). Subsequently, we employed a nested PCR assay to enhance the sensitivity of pathogen detection in contaminated soil. The first round of nested PCR utilized the primer pair EF1-F/EF1-R, followed by a second PCR using the specific primer pair EF1-Fb-F1/EF1-Fb-R5. This primer pair successfully amplified a 400 bp band from soil inoculated with at least 0.012 g of mycelial mat in 80g of soil (Fig 5B).

## Discussion

Fusarium wilt, induced by *F*. *buharicum*, poses an emerging threat to okra production in Japan. While the pathogen was initially reported in okra in Japan, it has been identified as capable of causing diseases in other Malvaceae plants as well [7, 9]. This pathogen exhibits survival capabilities in the soil, finding favorable conditions to infect okra plants. Although both seedlings and adult plants can fall prey to the pathogen, infections at the seedling stage can be particularly destructive, often leading to severe damage and eventual seedling death. Therefore, the development of a detection method for *F*. *buharicum* in infected plants and soils is crucial for the effective management of wilt disease. The detection method must be specific and sensitive to accurately identify the pathogen. In this study, we meticulously designed a species-specific primer and developed an assay for the specific and sensitive detection of *F*. *buharicum*. The designated primers, ELF Fb-F/ELF Fb-R, proved to be sensitive enough to detect the pathogen not only in fungal cultures but also in infected plants. Additionally, we developed a nested PCR assay to detect the pathogen in contaminated soil, especially in situations where its presence is in lower abundance.

The sequence analysis of the EF-1α gene in *F*. *buharicum* and 103 other fungal isolates was conducted to design a specific PCR primer pair. The designated primers successfully amplified a 400 bp fragment from *F*. *buharicum* genomic DNA, allowing for the discrimination of *F*. *buharicum* from other fungal species. Several species-specific primers, utilizing this gene, have been developed and employed for detecting various plant pathogenic fungi, including *Fusarium* [10, 31–33]. The primer developed in this study demonstrated high specificity for *F*. *buharicum* and sensitivity, capable of detecting the pathogen at concentrations as low as 1.0 ng of fungal genomic DNA in conventional PCR assays.

While it wasn’t possible to test the primer pair’s specificity against two closely related *F*. *buharicum* species, *Fusarium abutilonis* and *Fusarium* sp. ex, due to restrictions on the entry of new pathogens in Japan, a primer blast search and homology alignment with these species indicated a significant mismatch (>80%) between the alleles of the related species (Fig 1). The designed primers were also tested against other fungal pathogens, with no PCR amplification of expected size (~400 bp) detected in any of these cases. However, two of the fungal species, *S*. *sclerotiorum* and *S*. *cucurbitacearum*, produced amplicon larger than the expected size despite the primer mismatch higher than 60%. We assume that, if the primers anneal with the respective regions of *S*. *sclerotiorum* and *S*. *cucurbitacearum*, the amplicon should be approximately 400bp in size (S2 Fig). But the amplicon, detected in the gel electrophoresis, is much larger than the expected size. Therefore, the band observed in case of *S*. *sclerotiorum* and *S*. *cucurbitacearum* is not due to the primer mismatch. However, further research warrants the sequencing of the amplicons to resolve the problem. Furthermore, the primers exhibited sensitivity in detecting the pathogen from a 0.01 fg DNA sample in nested PCR assays. Overall, these results confirm the developed primer pair’s specificity and sensitivity for the detection of *F*. *buharicum*.

The extraction of sufficient fungal DNA from an infected plant sample is crucial for the detection of the pathogen. Typically, fungal pathogens are present in very low amounts during the early stages of infection, making the sensitivity of detection dependent on the infection stage. The detection method developed in this study successfully amplified a *F*. *buharicum*-specific band at a very early stage (7–10 days after pathogen inoculation) of infection, where the pathogen exhibited a thin black discoloration in the vascular system (Fig 4). However, this method could not detect the pathogen in artificially infected soil samples, likely due to the presence of inhibitors such as humic acid and degraded compounds from organic matter in the soil sample. These compounds are known to be strong inhibitors of Taq polymerase [34]. Subsequently, we employed a nested PCR assay to enhance the detection sensitivity of the pathogen in contaminated soil. The nested PCR assay successfully detected the *F*. *buharicum*-specific band in soil infested with 0.125g inoculum/80g of soil but not in the control soil sample (Fig 5). Several studies have emphasized the need for nested PCR to amplify DNA from soil samples and generate visible DNA bands [35–38].

It is crucial to take prompt actions to monitor the spread of the pathogen to other parts of Japan. The detection method developed in this study can be utilized to assess field soil before planting and after infection. This method is not only useful for monitoring the spread of the newly emerging pathogen but also for implementing necessary control measures against it.

## Supporting information

S1 Fig A multiple alignment of the nucleotide sequences of EF-1α region of *F*. *buharicum* and other fungus. The sequences were obtained from NCBI Genbank nucleotide databases and the alignment was made by GENETYX ver. 16 (https://www.genetyx.co.jp).
(PDF)

S2 Fig A multiple sequence alignment of EF-1α region of *F*. *buharicum* (FbOKI-1) along with *Sclerotinia sclerotiorum* (SsHAY30) and *Stagonosporopsis vannaccii* (SvPHB17). The sequences were obtained from NCBI Genbank nucleotide databases and aligned by using Multiple Sequence Alignment software **CLUSTALW**. The blue boxes indicate the *F*. *buharicum* species-specific primers (EF1Fb-F1 and EF1Fb-R5) sequences.
(PDF)

S1 Raw images (PDF)
