# Supplementary material for: Development of a PCR-based assay for specific and sensitive detection of Fusarium buharicum from infected okra plant
Source: PLoS One. 2024 Apr 16;19(4):e0302256. doi: 10.1371/journal.pone.0302256 (PMC11020393; doi:10.1371/journal.pone.0302256)
Supplement: S1 Fig — The sequences were obtained from NCBI Genbank nucleotide databases and the alignment was made by GENETYX ver. 16 (https://www.genetyx.co.jp). (PDF) [file pone.0302256.s001.pdf]

```
2.OM160859.1_F.buharicum                                             0 ---------------------------------------- 0
1.LC727524.1_F.buharicum_OKI-1_Okura                                 1 ---------------------TCACCTTAACGTCGTCGTC 19
3.KX302919.1_F.sublunatum                                            0 ---------------------------------------- 0
4.LT996094.1_F.convolutans                                           0 ---------------------------------------- 0
5.OM160861.1_F.abutilonis                                            0 ---------------------------------------- 0
6.OM160874.1_F.guadeloupense                                         0 ---------------------------------------- 0
7.MH392475.1_F.graminearum                                           0 ---------------------------------------- 0
8.MH582420.1_F.solani                                                0 ---------------------------------------- 0
9.MAFF244605_F.oxysporum                                             0 ---------------------------------------- 0
10.MAFF237278_F.contaminatum_Hylocereus                              1 ---GGTAAGG-AGGACAAGACTCACCTTAACGTCGTCGTC 36
11.MAFF237649_F.concentricum__Riceroot                               1 -----------AGGACAAGACTCACCTTAACGTCGTCGTC 29
12.MAFF237650_F.concentricum__Wheat                                  1 -----------AGGACAAGACTCACCTTAACGTCGTCGTC 29
13.MAFF239869_F.mangiferae__Ryukyupine                               1 ---------------------TCACCTTAACGTCGTCGTC 19
14.MAFF240460_F.fujikuroi_Passionfruit                               1 ---------------------TCACCTTAACGTCGTCGTC 19
15.MAFF241317_F.graminearum_Wheat                                    1 ---------------------TCACCTTAACGTCGTCGTC 19
16.MAFF242670_F.ipomoeae_Wheat                                       1 ------------------GACTCACCTTAACGTCGTCGTC 22
17.MAFF245129_F.concentricum_Fraxinus                                1 ---------------------TCACCTTAACGTCGTCGTC 19
18.MAFF245395_F.cugenangense_Rhubarb                                 1 ---------------------TCACCTTAACGTCGTCGTC 19
19.MAFF246637_F.nirenbergiae_Strawberry                              1 ---------------------TCACCTTAACGTCGTCGTC 19
20.MAFF246672_F.nirenbergiae_ChinesePeony                            1 ---------------------TCACCTTAACGTCGTCGTC 19
21.MAFF246697_F.commune_Urallicoriceroot                             1 ---------------------TCACCTTAACGTCGTCGTC 19
22.MAFF246729_F.falciforme_Angelica                                  1 ---------------------TCACCTCAACGTCGTCGTC 19
23.MAFF247220_F.duplospermum__Euwallaceasp                           1 ---------------------TCACCTCAACGTCGTCGTC 19
24.MAFF410760_F.odoratissimum_alpha                                  1 ---------------------TCACCTTAACGTCGTCGTC 19
25.MAFF244605_FusariumoxysporumSchlechtendal_MAFF244605_Tomato       1 ---------------------TCACCTTAACGTCGTCGTC 19
26.MAFF241326_F.asiaticum_Wheat                                      1 ------------------GACTCACCTTAACGTCGTCGTC 22
27.MAFF245014_F.asiaticum_Wildsoybean                                1 ---------------------TCACCTTAACGTCGTCGTC 19
28.MAFF150124_F.asiaticum__Wheat                                     1 ---------------------TCACCTTAACGTCGTCGTC 19
29.OM135603.1F.algeriense                                            1 ----------GAGGACAAGACTCACCTTAACGTCGTCGTC 30
30.MAFF237465_F.penzigii_Aloe                                        1 -----------AGGACAAGACTCACCTTAACGTCGTTGTC 29
31.MAFF103054_F.oxysporumSchlechtendalf.sp.cucumerinum_Cucumber      1 ---------------------TCACCTTAACGTCGTCGTC 19
32.MAFF712246_F.oxysporumSchlechtendalf.sp.dianthi__Carnation        1 ---------------------TCACCTTAACGTCGTCGTC 19
33.MAFF305558_F.oxysporumSchlechtendalf.sp.fragariae__Watermelon     1 ---------------------TCACCTTAACGTCGTCGTC 19
34.MAFF744087_F.oxysporumSchlechtendalf.sp.lactucae__Lettuce         1 ---------------------TCACCTTAACGTCGTCGTC 19
35.MAFF726924_F.oxysporumSchlechtendalf.sp.lagenariae_Whitefloweredgourd 1 ---------------------TCACCTTAACGTCGTCGTC 19
36.MAFF744003_F.oxysporumSchlechtendalf.sp.lagenariae_Squash)        1 ---------------------TCACCTTAACGTCGTCGTC 19
37.MAFF305122_F.oxysporumSchlechtendalf.sp.melonis__Melon            1 ---------------------TCACCTTAACGTCGTCGTC 19
38.MAFF306714_F.oxysporumSchlechtendalf.sp.momordicae_Balsampear     1 ---------------------TCACCTTAACGTCGTCGTC 19
39.MAFF238905_F.oxysporumSchlechtendalf.sp.radicis-lycopersici_Tomato 1 ---------------------TCACCTTAACGTCGTCGTC 19
40.MAFF150004_F.oxysporumSchlechtendalf.sp.spinaciae_Spinach         1 ---------------------TCACCTTAACGTCGTCGTC 19
41.MAFF247034_F.oxysporumSchlechtendal__Goldenchain                  1 ---------------------TCACCTTAACGTCGTCGTC 19
42.MAFF245747_F.oxysporumSchlechtendalf.sp.callistephi__Chinaaster   1 ---------------------TCACCTTAACGTCGTCGTC 19
43.MAFF305115_FoxysporumSchlechtendalf.sp.batatas__Sweatpotato       1 ---------------------TCACCTCAACGTCGTCGTC 19
44.MAFF150126_F.asiaticum_Seed                                       1 ---------------------TCACCTTAACGTCGTCGTC 19
45.MAFF246738_F.solani_Angelica                                      1 ---------------------TCACCTCAACGTCGTCGTC 19
46.MAFF246664_F.cugenangense_Perilla                                 1 ---------------------TCACCTTAACGTCGTCGTC 19
```

```
47.MH582420.1F.solanistrainMRC256                                    1 --------------------------------TCGTCGTC 8
48.MAFF240361_F.babinda_Soil                                         1 ---------------------TCACCTTAACGTCGTCGTC 19
49.MAFF242368_F.azukicola_Azukibean                                  1 ---------------------TCACCTCAACGTCGTGGTC 19
50.MAFF241312_F.asiaticum_Soil,welshonionfield                       1 ---------------------TCACCTTAACGTCGTCGTC 19
51.LT548416.1_F.culmorumpartialtef1a                                 1 -------------------------CTTAACGTCGTCGTC 15
52.MAFF150124_F.asiaticum__Wheat                                     1 ---------------------TCACCTTAACGTCGTCGTC 19
53.MAFF238806_F.begoniae_Oncidiumsp                                  1 --GGGTAAGGGAAGACAAGACTCACCTTAACGTCGTCGTC 38
54.MW594399.1_FusariumincarnatumisolateUD01C                         1 -------------GACAAGACTCACCTTAACGTCGTCGTC 27
55.OP414923.1Pucciniagraminisf.sp.triticiisolateSHZPgt19             0 ---------------------------------------- 0
56.MT027094.1_BipolarisoryzaestrainOrL-2                             0 ---------------------------------------- 0
57.ON734360.1_AlternariaalternataisolateH126                         0 ---------------------------------------- 0
58.LC333578.1_StemphyliumlycopersiciSOasp2                           0 ---------------------------------------- 0
59.HQ718583.1_Colletotrichumgloeosporioidesisolateq-1                1 ---------------------TCACATCAACGTCGTCGTC 19
60.JN241603.1_AtheliarolfsiiisolateSR1                               0 ---------------------------------------- 0
61.KJ866474.1_RhizoctoniasolanistrainMHL-1                           0 ---------------------------------------- 0
62.JQ672424.1AlternariatriticinaisolateEGS17-061                     0 ---------------------------------------- 0
63.LT707559.1_P.capsicipartialtef1agene                              0 ---------------------------------------- 0
64.MW090051.1_CurvularialunatastrainCls-3                            0 ---------------------------------------- 0
65.DQ400892.1_Aspergillusterreus                                     0 ---------------------------------------- 0
66.DQ911416.1_Pythiumsp.quercumstrainPy292                           0 ---------------------------------------- 0
67.EU797495.1_Phytophthorasp.oaksoilPoland                           0 ---------------------------------------- 0
68.HM148321.1_Cladosporiumcucumerinum                                0 ---------------------------------------- 0
69.AF398888.1_SclerotiniasclerotiorumisolateSS1                      0 ---------------------------------------- 0
70.AF398888.1_S.sclerotiorumisolateSS1                               0 ---------------------------------------- 0
71.HPAB545908.1_Verticilliumnonalfalfaeisolate                       0 ---------------------------------------- 0
72.EF433315.1_CeratocystisfimbriatavoucherCMW15052                   0 ---------------------------------------- 0
73.MN159912.1_Botrytiscinerea                                        0 ---------------------------------------- 0
74.MF034741.1_PeltasterfructicolaisolateSRB92                        0 ---------------------------------------- 0
75.LC440360.1_CercosporaasparagiCOasp2                               0 ---------------------------------------- 0
76.AY944105.1_MagnaportheoryzaeisolateSAG00T3()                      0 ---------------------------------------- 0
77.JX266586.1_CochliobolusmiyabeanusvoucherMFLUCC10-0733             0 ---------------------------------------- 0
78.MN393253.1_CorynesporacassiicolaisolateQHD001(MN393253.1UNVERIFIED) 0 ---------------------------------------- 0
79.MF375218.1_AgroatheliarolfsiiisolateBJB24                         0 ---------------------------------------- 0
80.MN106270.1_AgroatheliarolfsiistrainJ-12                           0 ---------------------------------------- 0
81.OQ732628.1_AgroatheliarolfsiiisolateBTCBSr3                       0 ---------------------------------------- 0
82.KY196185.1_ColletotrichumtruncatumstrainPAK53                     1 ATGGGTAAGG-AGGACAAGACTCACATCAACGTCGTCGTT 39
83.GU935835.1_ColletotrichumcoccodesisolateC96002                    0 ---------------------------------------- 0
84.MK085963.1_AlternariatenuissimaisolateSCCZ06                      0 ---------------------------------------- 0
85.MT548042.1_AlternarialongipesstrainKY_2019_012                    0 ---------------------------------------- 0
86.MN356465.1CalonectriamontanaisolateHSP4                           0 ---------------------------------------- 0
87.OL694224.1_CalonectriacanadianastrainF099                         0 ---------------------------------------- 0
88.MK803351.1_NeoscytalidiumdimidiatumstrainKale4-C                  0 ---------------------------------------- 0
89.ON376993.1_CurvulariachiangmaiensisisolateND00J7                  0 ---------------------------------------- 0
90.OQ383346.1_NeoscytalidiumdimidiatumisolateGKH-2                   0 ---------------------------------------- 0
91.MF662595.1_NeoscytalidiumnovaehollandiaeisolateNeNo1              0 ---------------------------------------- 0
92.EF560588.1Melampsoralini                                          0 ---------------------------------------- 0
```

93.LC590862.1_NeoscytalidiumdimidiatumPSU-HP01TEF1	0	--	0
94.KX278106.1_BotryosphaeriaqingyuanensisstrainCERC2947	0	--	0
95.AJ578763.1_Blumeriagraminisf.sp.hordeicyp51	0	--	0
96.MF490858.1_CurvulariadactylocteniicolastrainCPC28810	0	--	0
97.KT287115.1_Bipolariscactivoraisolate3.8.6	0	--	0
98.MT560940.1_CurvulariacactivorastrainHLGH0118	0	--	0
99.OM714565.1_CurvulariaplantarumstrainM0134	0	--	0
100.MN159911.1_BotrytiscinereaSICAUCC19-0003	0	--	0
102.GU294713.1LasiodiplodiatheobromaestrainUCD2430TX	0	--	0
103.KX868094.1_Mycosphaerellasp.isolateCRM20.1	0	--	0
104.LC599478.1Pseudocercosporapini-densifloraeMUCC534	0	--	0
105.N584698.1BipolarissetariaestrainKBS4-2	0	--	0

2.OM160859.1_F.buharicum	1	------------GACTCTGGCAAGTCGACCACTGTGAGTA	28
1.LC727524.1_F.buharicum_OKI-1_Okura	20	ATCGGCCACGTCGACTCTGGCAAGTCGACCACTGTGAGTA	59
3.KX302919.1_F.sublunatum	1	------------GACTCTGGCAAGTCGACCACTGTGAGTA	28
4.LT996094.1_F.convolutans	1	------------GACTCTGGCAAGTCGACCACTGTGAGTA	28
5.OM160861.1_F.abutilonis	1	------------GACTCTGGCAAGTCGACCACTGTGAGTA	28
6.OM160874.1_F.guadeloupense	1	------------GACTCTGGCAAGTCGACCACTGTGAGTA	28
7.MH392475.1_F.graminearum	0	--	0
8.MH582420.1_F.solani	1	------------GACTCTGGCAAGTCGACCACCGTAAGTC	28
9.MAFF244605_F.oxysporum	1	------------GACTCTGGCAAGTCGACCACTGTGAGTA	28
10.MAFF237278_F.contaminatum_Hylocereus	37	ATCGGCCACGTCGACTCTGGCAAGTCGACCACTGTGAGTA	76
11.MAFF237649_F.concentricum__Riceroot	30	ATCGGCCACGTCGACTCTGGCAAGTCGACCACTGTGAGTA	69
12.MAFF237650_F.concentricum__Wheat	30	ATCGGCCACGTCGACTCTGGCAAGTCGACCACTGTGAGTA	69
13.MAFF239869_F.mangiferae__Ryukyupine	20	ATCGGCCACGTCGACTCTGGCAAGTCGACCACTGTGAGTA	59
14.MAFF240460_F.fujikuroi_Passionfruit	20	ATCGGCCACGTCGACTCTGGCAAGTCGACCACTGTGAGTA	59
15.MAFF241317_F.graminearum_Wheat	20	ATCGGCCACGTCGACTCTGGCAAGTCGACCACTGTGAGTA	59
16.MAFF242670_F.ipomoeae_Wheat	23	ATCGGCCACGTCGACTCTGGCAAGTCGACCACTGTGAGTA	62
17.MAFF245129_F.concentricum_Fraxinus	20	ATCGGCCACGTCGACTCTGGCAAGTCGACCACTGTGAGTA	59
18.MAFF245395_F.cugenangense_Rhubarb	20	ATCGGCCACGTCGACTCTGGCAAGTCGACCACTGTGAGTA	59
19.MAFF246637_F.nirenbergiae_Strawberry	20	ATCGGCCACGTCGACTCTGGCAAGTCGACCACTGTGAGTA	59
20.MAFF246672_F.nirenbergiae_ChinesePeony	20	ATCGGCCACGTCGACTCTGGCAAGTCGACCACTGTGAGTA	59
21.MAFF246697_F.commune_Urallicoriceroot	20	ATCGGCCACGTCGACTCTGGCAAGTCGACCACTGTGAGTA	59
22.MAFF246729_F.falciforme_Angelica	20	ATCGGCCACGTCGACTCTGGCAAGTCGACCACCGTAAGTC	59
23.MAFF247220_F.duplospermum__Euwallaceasp	20	ATCGGCCACGTCGACTCTGGCAAGTCGACCACCGTAAGTC	59
24.MAFF410760_F.odoratissimum_alpha	20	ATCGGCCACGTCGACTCTGGCAAGTCGACCACTGTGAGTA	59
25.MAFF244605_FusariumoxysporumSchlechtendal_MAFF244605_Tomato	20	ATCGGCCACGTCGACTCTGGCAAGTCGACCACTGTGAGTA	59
26.MAFF241326_F.asiaticum_Wheat	23	ATCGGCCACGTCGACTCTGGCAAGTCGACCACTGTGAGTA	62
27.MAFF245014_F.asiaticum_Wildsoybean	20	ATCGGCCACGTCGACTCTGGCAAGTCGACCACTGTGAGTA	59
28.MAFF150124_F.asiaticum__Wheat	20	ATCGGCCACGTCGACTCTGGCAAGTCGACCACTGTGAGTA	59
29.OM135603.1F.algeriense	31	ATCGGCCACGTCGACTCTGGCAAGTCGACCACTGTGAGTA	70
30.MAFF237465_F.penzigii_Aloe	30	ATCGGACACGTCGACTCCGGAAAGTCCACCACTGTAAGTT	69
31.MAFF103054_F.oxysporumSchlechtendalf.sp.cucumerinum_Cucumber	20	ATCGGCCACGTCGACTCTGGCAAGTCGACCACTGTGAGTA	59
32.MAFF712246_F.oxysporumSchlechtendalf.sp.dianthi__Carnation	20	ATCGGCCACGTCGACTCTGGCAAGTCGACCACTGTGAGTA	59
33.MAFF305558_F.oxysporumSchlechtendalf.sp.fragariae__Watermelon	20	ATCGGCCACGTCGACTCTGGCAAGTCGACCACTGTGAGTA	59

34.MAFF744087_F.oxysporumSchlechtendalf.sp.lactucae__Lettuce	20	ATCGGCCACGTCGACTCTGGCAAGTCGACCACTGTGAGTA	59
35.MAFF726924_F.oxysporumSchlechtendalf.sp.lagenariae_Whitefloweredgourd	20	ATCGGCCACGTCGACTCTGGCAAGTCGACCACTGTGAGTA	59
36.MAFF744003_F.oxysporumSchlechtendalf.sp.lagenariae_Squash)	20	ATCGGCCACGTCGACTCTGGCAAGTCGACCACTGTGAGTA	59
37.MAFF305122_F.oxysporumSchlechtendalf.sp.melonis__Melon	20	ATCGGCCACGTCGACTCTGGCAAGTCGACCACTGTGAGTA	59
38.MAFF306714_F.oxysporumSchlechtendalf.sp.momordicae_Balsampear	20	ATCGGCCACGTCGACTCTGGCAAGTCGACCACTGTGAGTA	59
39.MAFF238905_F.oxysporumSchlechtendalf.sp.radicis-lycopersici_Tomato	20	ATCGGCCACGTCGACTCTGGCAAGTCGACCACTGTGAGTA	59
40.MAFF150004_F.oxysporumSchlechtendalf.sp.spinaciae_Spinach	20	ATCGGCCACGTCGACTCTGGCAAGTCGACCACTGTGAGTA	59
41.MAFF247034_F.oxysporumSchlechtendal__Goldenchain	20	ATCGGCCACGTCGACTCTGGCAAGTCGACCACTGTGAGTA	59
42.MAFF245747_F.oxysporumSchlechtendalf.sp.callistephi__Chinaaster	20	ATCGGCCACGTCGACTCTGGCAAGTCGACCACTGTGAGTA	59
43.MAFF305115_FoxysporumSchlechtendalf.sp.batatas__Sweatpotato	20	ATCGGCCACGTCGACTCTGGCAAGTCGACCACCGTAAGTC	59
44.MAFF150126_F.asiaticum_Seed	20	ATCGGCCACGTCGACTCTGGCAAGTCGACCACTGTGAGTA	59
45.MAFF246738_F.solani_Angelica	20	ATCGGCCACGTCGACTCTGGCAAGTCGACCACCGTAAGTC	59
46.MAFF246664_F.cugenangense_Perilla	20	ATCGGCCACGTCGACTCTGGCAAGTCGACCACTGTGAGTA	59
47.MH582420.1F.solanistrainMRC256	9	ATCGGCCACGTCGACTCTGGCAAGTCGACCACCGTAAGTC	48
48.MAFF240361_F.babinda_Soil	20	ATCGGCCACGTCGACTCTGGCAAGTCGACCACTGTGAGTA	59
49.MAFF242368_F.azukicola_Azukibean	20	ATCGGCCACGTCGACTCTGGCAAGTCGACCACCGTAAGTC	59
50.MAFF241312_F.asiaticum_Soil,welshonionfield	20	ATCGGCCACGTCGACTCTGGCAAGTCGACCACTGTGAGTA	59
51.LT548416.1_F.culmorumpartialtef1a	16	ATCGGCCACGTCGACTCTGGCAAGTCGACCACTGTGAGTA	55
52.MAFF150124_F.asiaticum__Wheat	20	ATCGGCCACGTCGACTCTGGCAAGTCGACCACTGTGAGTA	59
53.MAFF238806_F.begoniae_Oncidiumsp	39	ATCGGCCACGTCGACTCTGGCAAGTCGACCACTGTGAGTA	78
54.MW594399.1_FusariumincarnatumisolateUD01C	28	ATCGGCCACGTCGACTCTGGCAAGTCGACCACTGTGAGTA	67
55.OP414923.1Pucciniagraminisf.sp.triticiisolateSHZPgt19	0	--	0
56.MT027094.1_BipolarisoryzaestrainOrL-2	0	--	0
57.ON734360.1_AlternariaalternataisolateH126	0	--	0
58.LC333578.1_StemphyliumlycopersiciSOasp2	0	--	0
59.HQ718583.1_Colletotrichumgloeosporioidesisolateq-1	20	ATCGGCCACGTCGACTCTGGCAAGTCGACCACCACCGTAA	59
60.JN241603.1_AtheliarolfsiiisolateSR1	0	--	0
61.KJ866474.1_RhizoctoniasolanistrainMHL-1	0	--	0
62.JQ672424.1AlternariatriticinaisolateEGS17-061	0	--	0
63.LT707559.1_P.capsicipartialtef1agene	0	--	0
64.MW090051.1_CurvularialunatastrainCls-3	0	--	0
65.DQ400892.1_Aspergillusterreus	0	--	0
66.DQ911416.1_Pythiumsp.quercumstrainPy292	0	--	0
67.EU797495.1_Phytophthorasp.oaksoilPoland	0	--	0
68.HM148321.1_Cladosporiumcucumerinum	0	--	0
69.AF398888.1_SclerotiniasclerotiorumisolateSS1	0	--	0
70.AF398888.1_S.sclerotiorumisolateSS1	0	--	0
71.HPAB545908.1_Verticilliumnonalfalfaeisolate	0	--	0
72.EF433315.1_CeratocystisfimbriatavoucherCMW15052	1	-----------------------------------CCATT	5
73.MN159912.1_Botrytiscinerea	0	--	0
74.MF034741.1_PeltasterfructicolaisolateSRB92	0	--	0
75.LC440360.1_CercosporaasparagiCOasp2	0	--	0
76.AY944105.1_MagnaportheoryzaeisolateSAG00T3()	0	--	0
77.JX266586.1_CochliobolusmiyabeanusvoucherMFLUCC10-0733	0	--	0
78.MN393253.1_CorynesporacassiicolaisolateQHD001(MN393253.1UNVERIFIED)	0	--	0
79.MF375218.1_AgroatheliarolfsiiisolateBJB24	0	--	0

```
80.MN106270.1_AgroatheliarolfsiistrainJ-12                        0 ---------------------------------------- 0
81.OQ732628.1_AgroatheliarolfsiiisolateBTCBSr3                    0 ---------------------------------------- 0
82.KY196185.1_ColletotrichumtruncatumstrainPAK53                 40 ATCGGCCACGTCGACTCTGGCAAGTCGACCACCACT---- 75
83.GU935835.1_ColletotrichumcoccodesisolateC96002                 0 ---------------------------------------- 0
84.MK085963.1_AlternariatenuissimaisolateSCCZ06                   0 ---------------------------------------- 0
85.MT548042.1_AlternarialongipesstrainKY_2019_012                 0 ---------------------------------------- 0
86.MN356465.1CalonectriamontanaisolateHSP4                        0 ---------------------------------------- 0
87.OL694224.1_CalonectriacanadianastrainF099                      0 ---------------------------------------- 0
88.MK803351.1_NeoscytalidiumdimidiatumstrainKale4-C               0 ---------------------------------------- 0
89.ON376993.1_CurvulariachiangmaiensisisolateND00J7               0 ---------------------------------------- 0
90.OQ383346.1_NeoscytalidiumdimidiatumisolateGKH-2                0 ---------------------------------------- 0
91.MF662595.1_NeoscytalidiumnovaehollandiaeisolateNeNo1           0 ---------------------------------------- 0
92.EF560588.1Melampsoralini                                       0 ---------------------------------------- 0
93.LC590862.1_NeoscytalidiumdimidiatumPSU-HP01TEF1                0 ---------------------------------------- 0
94.KX278106.1_BotryosphaeriaqingyuanensisstrainCERC2947           0 ---------------------------------------- 0
95.AJ578763.1_Blumeriagraminisf.sp.hordeicyp51                    0 ---------------------------------------- 0
96.MF490858.1_CurvulariadactylocteniicolastrainCPC28810           0 ---------------------------------------- 0
97.KT287115.1_Bipolariscactivoraisolate3.8.6                      0 ---------------------------------------- 0
98.MT560940.1_CurvulariacactivorastrainHLGH0118                   0 ---------------------------------------- 0
99.OM714565.1_CurvulariaplantarumstrainM0134                      0 ---------------------------------------- 0
100.MN159911.1_BotrytiscinereaSICAUCC19-0003                      0 ---------------------------------------- 0
102.GU294713.1LasiodiplodiatheobromaestrainUCD2430TX              0 ---------------------------------------- 0
103.KX868094.1_Mycosphaerellasp.isolateCRM20.1                    0 ---------------------------------------- 0
104.LC599478.1Pseudocercosporapini-densifloraeMUCC534             0 ---------------------------------------- 0
105.N584698.1BipolarissetariaestrainKBS4-2                        0 ---------------------------------------- 0

2.OM160859.1_F.buharicum                                         29 CTACCC--TC-GACGGTGTGCTTGCTTGCACTCGTCAAAC 65
1.LC727524.1_F.buharicum_OKI-1_Okura                             60 CTACCC--TC-GACGGTGTGCTTGCTTGCACTCGTCAAAC 96
3.KX302919.1_F.sublunatum                                        29 CAACCC--TC-GGCGAGCTGCTTGTCTGCACTCGTCAAAC 65
4.LT996094.1_F.convolutans                                       29 CTACCC--TC-GACGAGTTGCTTGTCTGCACTCGTCAAAC 65
5.OM160861.1_F.abutilonis                                        29 CTACCC--TC-GACGATGTGCATGTCTGCACTCGTCAAAC 65
6.OM160874.1_F.guadeloupense                                     29 CCACCC--TC-GACGATGTGCTCATCTGCACTCGTACAAC 65
7.MH392475.1_F.graminearum                                        1 ------------------------AGTACCACCGCATCCC 16
8.MH582420.1_F.solani                                            29 AAACCC-TCATCGCGATCTGCTTATCTCGGGTCGTGGAAC 67
9.MAFF244605_F.oxysporum                                         29 CTCCCC--TTAGACGATGAGCTTATCTGCCATCGTTAATC 66
10.MAFF237278_F.contaminatum_Hylocereus                          77 CTCTCC--TC-GACAATGAGCTTATCTGCCATCGTCAATC 113
11.MAFF237649_F.concentricum__Riceroot                           70 CTACCC--CT-GACGATGAGCTTATCTGCCATCGT-AATC 105
12.MAFF237650_F.concentricum__Wheat                              70 CTACCC--CT-GACGATGAGCTTATCTGCCATCGT-AATC 105
13.MAFF239869_F.mangiferae__Ryukyupine                           60 CTACCC--TT-GATGATGAGCTTATCTGCCATCGT-AATC 95
14.MAFF240460_F.fujikuroi_Passionfruit                           60 CTACCC--TC-GACGATGAGCTTATCTGTCATCGT-GATC 95
15.MAFF241317_F.graminearum_Wheat                                60 CCA-CC----------------------GCA-TCC-CAACC 75
16.MAFF242670_F.ipomoeae_Wheat                                   63 CTA-CC--CTCAATGACCTGCTTATCAGCAGTCATCAACC 99
17.MAFF245129_F.concentricum_Fraxinus                            60 CTACCC--CT-GACGATGAGCTTATCTGCCATCGT-AATC 95
18.MAFF245395_F.cugenangense_Rhubarb                             60 CTCTCC--TC-GACAATGAGCATATCTGCCATCGTCAATC 96
19.MAFF246637_F.nirenbergiae_Strawberry                          60 CTCTCC--TC-GACAATGAGCTTATCTGCCATCGTCAATC 96
20.MAFF246672_F.nirenbergiae_ChinesePeony                        60 CTCTCC--TC-GACAATGAGCTTATCTGCCATCGTCAATC 96
```

```
21.MAFF246697_F.commune_Urallicoriceroot                                  60 CTCCCC--TTGGACGATGAGCTTATCTGCCATCGTTAATC 97
22.MAFF246729_F.falciforme_Angelica                                       60 AAACCC-TCATCGCGATCTGCTTATCTCGGGTCGTGGAAC 98
23.MAFF247220_F.duplospermum__Euwallaceasp                                60 AAACCC-TCATCGCGATCTACTTATCTCGGATCGTGGAAC 98
24.MAFF410760_F.odoratissimum_alpha                                       60 CTCTCC--TC-GACAATGAGCTTATCTGCCATCGTCAATC 96
25.MAFF244605_FusariumoxysporumSchlechtendal_MAFF244605_Tomato            60 CTCCCC--TTAGACGATGAGCTTATCTGCCATCGTTAATC 97
26.MAFF241326_F.asiaticum_Wheat                                           63 CCA-CC---------------------GTA-TCC-CAACC 78
27.MAFF245014_F.asiaticum_Wildsoybean                                     60 CCA-CC---------------------GCA-TCC-CAACC 75
28.MAFF150124_F.asiaticum__Wheat                                          60 CCA-CC---------------------GCA-TCC-CAACC 75
29.OM135603.1F.algeriense                                                 71 CTCCCC--TC-GACGATTCGCTTATTTGCCATCGTCAACC 107
30.MAFF237465_F.penzigii_Aloe                                             70 CTCACT-CACCCATGA-CTGTCCATCAGCATCAGTCTTAC 107
31.MAFF103054_F.oxysporumSchlechtendalf.sp.cucumerinum_Cucumber           60 CTCTCC--TC-GACAATGAGCATATCTGCCATCGTCAATC 96
32.MAFF712246_F.oxysporumSchlechtendalf.sp.dianthi__Carnation             60 CTCTCC--TC-GACAATGAGCTTATCTGCCATCGTCAATC 96
33.MAFF305558_F.oxysporumSchlechtendalf.sp.fragariae__Watermelon          60 CTCTCC--TC-GACAATGAGCATATCTGCCATCGTCAATC 96
34.MAFF744087_F.oxysporumSchlechtendalf.sp.lactucae__Lettuce              60 CTCTCC--TC-GACAATGAGCATATCTGCCATCGTCAATC 96
35.MAFF726924_F.oxysporumSchlechtendalf.sp.lagenariae_Whitefloweredgourd  60 CTCTCC--TC-GACAATGAGCATATCTGCCATCGTCAATC 96
36.MAFF744003_F.oxysporumSchlechtendalf.sp.lagenariae_Squash)             60 CTCTCC--TC-GACAATGAGCTTATCTGCCATCGTCAATC 96
37.MAFF305122_F.oxysporumSchlechtendalf.sp.melonis__Melon                 60 CTCTCC--TC-GACAATGAGCATATCTGCCATCGTCAATC 96
38.MAFF306714_F.oxysporumSchlechtendalf.sp.momordicae_Balsampear          60 CTCTCC--TC-GACAATGAGCATATCTGCCATCGTCAATC 96
39.MAFF238905_F.oxysporumSchlechtendalf.sp.radicis-lycopersici_Tomato     60 CTCTCC--TC-GACAATGAGCTTATCTGCCATCGTCAATC 96
40.MAFF150004_F.oxysporumSchlechtendalf.sp.spinaciae_Spinach              60 CTCTCC--TC-GACAATGAGCATATCTGCCATCGTCAATC 96
41.MAFF247034_F.oxysporumSchlechtendal__Goldenchain                       60 CTCTCC--TC-GACAATGAGCATATCTGCCATCGTCAATC 96
42.MAFF245747_F.oxysporumSchlechtendalf.sp.callistephi__Chinaaster        60 CTCTCC--TC-GACAATGAGCATATCTGCCATCGTCAATC 96
43.MAFF305115_FoxysporumSchlechtendalf.sp.batatas__Sweatpotato            60 AAACCC-TCATCGCGATCTGCTTATCTCGGGTCGTGGAAC 98
44.MAFF150126_F.asiaticum_Seed                                            60 CCA-CC---------------------GCA-TCC-CAACC 75
45.MAFF246738_F.solani_Angelica                                           60 AAACCC-TCATCGCGATCTGCTTATCTCGGGTCGTGGAAC 98
46.MAFF246664_F.cugenangense_Perilla                                      60 CTCTCC--TC-GACAATGAGCATATCTGCCATCGTCAATC 96
47.MH582420.1F.solanistrainMRC256                                         49 AAACCC-TCATCGCGATCTGCTTATCTCGGGTCGTGGAAC 87
48.MAFF240361_F.babinda_Soil                                              60 CTACCC--TC-GACGATCTGCTTCTTTGCACTCGTCAATC 96
49.MAFF242368_F.azukicola_Azukibean                                       60 ACCCCCCTCATGGCGACCTGCTTATCTCGGGTCGTGGAAC 99
50.MAFF241312_F.asiaticum_Soil,welshonionfield                            60 CCA-CC---------------------GCA-TCC-CAACC 75
51.LT548416.1_F.culmorumpartialtef1a                                      56 CCA-CT---------------------GCA-TCC-CAACC 71
52.MAFF150124_F.asiaticum__Wheat                                          60 CCA-CC---------------------GCA-TCC-CAACC 75
53.MAFF238806_F.begoniae_Oncidiumsp                                       79 CTACCC--TC-GACGATGAGCTTATCTGCCATCGT-TATC 114
54.MW594399.1_FusariumincarnatumisolateUD01C                              68 CTACCC--AC-GATGATTTGCTTATCAGCAGTCATCAACC 104
55.OP414923.1Pucciniagraminisf.sp.triticiisolateSHZPgt19                   0 ---------------------------------------- 0
56.MT027094.1_BipolarisoryzaestrainOrL-2                                   0 ---------------------------------------- 0
57.ON734360.1_AlternariaalternataisolateH126                               0 ---------------------------------------- 0
58.LC333578.1_StemphyliumlycopersiciSOasp2                                 0 ---------------------------------------- 0
59.HQ718583.1_Colletotrichumgloeosporioidesisolateq-1                     60 GTTCAATCACCCATGCCTGCATCAGCTACTGCGACATCAT 99
60.JN241603.1_AtheliarolfsiiisolateSR1                                     0 ---------------------------------------- 0
61.KJ866474.1_RhizoctoniasolanistrainMHL-1                                 0 ---------------------------------------- 0
62.JQ672424.1AlternariatriticinaisolateEGS17-061                           0 ---------------------------------------- 0
63.LT707559.1_P.capsicipartialtef1agene                                    0 ---------------------------------------- 0
64.MW090051.1_CurvularialunatastrainCls-3                                  0 ---------------------------------------- 0
65.DQ400892.1_Aspergillusterreus                                           0 ---------------------------------------- 0
66.DQ911416.1_Pythiumsp.quercumstrainPy292                                 0 ---------------------------------------- 0
```

```
67.EU797495.1_Phytophthorasp.oaksoilPoland                                0 ---------------------------------------- 0
68.HM148321.1_Cladosporiumcucumerinum                                     0 ---------------------------------------- 0
69.AF398888.1_SclerotiniasclerotiorumisolateSS1                           0 ---------------------------------------- 0
70.AF398888.1_S.sclerotiorumisolateSS1                                    0 ---------------------------------------- 0
71.HPAB545908.1_Verticilliumnonalfalfaeisolate                            0 ---------------------------------------- 0
72.EF433315.1_CeratocystisfimbriatavoucherCMW15052                        6 GAGAAGTTCGAGAATAAGTTTCCCCTATTTCCCCTCATTG 45
73.MN159912.1_Botrytiscinerea                                             0 ---------------------------------------- 0
74.MF034741.1_PeltasterfructicolaisolateSRB92                             0 ---------------------------------------- 0
75.LC440360.1_CercosporaasparagiCOasp2                                    0 ---------------------------------------- 0
76.AY944105.1_MagnaportheoryzaeisolateSAG00T3()                           0 ---------------------------------------- 0
77.JX266586.1_CochliobolusmiyabeanusvoucherMFLUCC10-0733                  0 ---------------------------------------- 0
78.MN393253.1_CorynesporacassiicolaisolateQHD001(MN393253.1UNVERIFIED)    0 ---------------------------------------- 0
79.MF375218.1_AgroatheliarolfsiiisolateBJB24                              0 ---------------------------------------- 0
80.MN106270.1_AgroatheliarolfsiistrainJ-12                                0 ---------------------------------------- 0
81.OQ732628.1_AgroatheliarolfsiiisolateBTCBSr3                            0 ---------------------------------------- 0
82.KY196185.1_ColletotrichumtruncatumstrainPAK53                         75 ---------------------------------------- 75
83.GU935835.1_ColletotrichumcoccodesisolateC96002                         0 ---------------------------------------- 0
84.MK085963.1_AlternariatenuissimaisolateSCCZ06                           0 ---------------------------------------- 0
85.MT548042.1_AlternarialongipesstrainKY_2019_012                         0 ---------------------------------------- 0
86.MN356465.1CalonectriamontanaisolateHSP4                                0 ---------------------------------------- 0
87.OL694224.1_CalonectriacanadianastrainF099                              0 ---------------------------------------- 0
88.MK803351.1_NeoscytalidiumdimidiatumstrainKale4-C                       0 ---------------------------------------- 0
89.ON376993.1_CurvulariachiangmaiensisisolateND00J7                       0 ---------------------------------------- 0
90.OQ383346.1_NeoscytalidiumdimidiatumisolateGKH-2                        0 ---------------------------------------- 0
91.MF662595.1_NeoscytalidiumnovaehollandiaeisolateNeNo1                   0 ---------------------------------------- 0
92.EF560588.1Melampsoralini                                               0 ---------------------------------------- 0
93.LC590862.1_NeoscytalidiumdimidiatumPSU-HP01TEF1                        0 ---------------------------------------- 0
94.KX278106.1_BotryosphaeriaqingyuanensisstrainCERC2947                   0 ---------------------------------------- 0
95.AJ578763.1_Blumeriagraminisf.sp.hordeicyp51                            0 ---------------------------------------- 0
96.MF490858.1_CurvulariadactylocteniicolastrainCPC28810                   0 ---------------------------------------- 0
97.KT287115.1_Bipolariscactivoraisolate3.8.6                              0 ---------------------------------------- 0
98.MT560940.1_CurvulariacactivorastrainHLGH0118                           0 ---------------------------------------- 0
99.OM714565.1_CurvulariaplantarumstrainM0134                              0 ---------------------------------------- 0
100.MN159911.1_BotrytiscinereaSICAUCC19-0003                              0 ---------------------------------------- 0
102.GU294713.1LasiodiplodiatheobromaestrainUCD2430TX                      0 ---------------------------------------- 0
103.KX868094.1_Mycosphaerellasp.isolateCRM20.1                            0 ---------------------------------------- 0
104.LC599478.1Pseudocercosporapini-densifloraeMUCC534                     0 ---------------------------------------- 0
105.N584698.1BipolarissetariaestrainKBS4-2                                0 ---------------------------------------- 0

2.OM160859.1_F.buharicum                                                 66 CTCCATACAGATTTCTGGCGAGGTTTTCATCATT-TATAT 104
1.LC727524.1_F.buharicum_OKI-1_Okura                                     97 CTCCATCCAGATTTCTGGCGAGGTTTTCATCATT-TATAT 135
3.KX302919.1_F.sublunatum                                                66 CCCGCCTCTGATCCATGGCGGGGT---CTTCAGT-CATAT 101
4.LT996094.1_F.convolutans                                               66 CCCGCCTCAGACCCATGGCGGGGG---TTTCAGT-TCCAT 101
5.OM160861.1_F.abutilonis                                                66 CCGC-TCCAGATGTTTGGCGGGGTATTCATCAAT-CACAT 103
6.OM160874.1_F.guadeloupense                                             66 C-CCGCCTAGATCTCTAGCGGGGTATTCATCGGT-CACAT 103
7.MH392475.1_F.graminearum                                               17 AACCCCGCCGACACTTGGCGGGGTAGTTTCAAATTTCCAA 56
```

8.MH582420.1_F.solani	68	CCCGCCTGGCATCTCGGGCGGGGTATTCATCATT-CACTT	106
9.MAFF244605_F.oxysporum	67	CCGAC-CAAGAC--CTGGCGGGGTATTTCTCAAA-GGCAA	102
10.MAFF237278_F.contaminatum_Hylocereus	114	CCGAC-CAAGAC--CTGGTGGGGTATTTCTCAAA-GTCAA	149
11.MAFF237649_F.concentricum__Riceroot	106	CTGAC-CAAGAT--CTGGCGGGGTGTATCTCAAAAGACAA	142
12.MAFF237650_F.concentricum__Wheat	106	CTGAC-CAAGAT--CTGGCGGGGTGTATCTCAAAAGACAA	142
13.MAFF239869_F.mangiferae__Ryukyupine	96	CTGAC-CAAGAT--CTGGCGGGGTATATCTCAAAAGACAA	132
14.MAFF240460_F.fujikuroi_Passionfruit	96	CTGAC-CAAGAT--CTGGCGGGGTATATCTCAGAAGACAA	132
15.MAFF241317_F.graminearum_Wheat	76	CCGCC--GACAC--TTGGCGGGGTAGTTTCAAATTTCCAA	111
16.MAFF242670_F.ipomoeae_Wheat	100	CCGCC---ATAC--TTGGTGGGGTAAATTCAACTTACACA	134
17.MAFF245129_F.concentricum_Fraxinus	96	CTGAC-CAAGAT--CTGGCGGGGTGTATCTCAAAAGACAA	132
18.MAFF245395_F.cugenangense_Rhubarb	97	CCGAC-CAAGAC--CTGGCGGGGTATTTCTCAAA-GTCAA	132
19.MAFF246637_F.nirenbergiae_Strawberry	97	CCGAC-CAAGAC--CTGGTGGGGTATTTCTCAAA-GTCAA	132
20.MAFF246672_F.nirenbergiae_ChinesePeony	97	CCGAC-CAAGAC--CTGGTGGGGTATTTCTCAAA-GTCAA	132
21.MAFF246697_F.commune_Urallicoriceroot	98	CCGAC-CAAGAC--CTGGCGGGGTATTTCTCAAA-GGCAA	133
22.MAFF246729_F.falciforme_Angelica	99	CCCGCCTGGCATCTCGGGCGGGGTATTCATCAGT-CACTT	137
23.MAFF247220_F.duplospermum__Euwallaceasp	99	CCCGCCTGGCATCTCGGGCGGGGTATGCATCAGT-CACTT	137
24.MAFF410760_F.odoratissimum_alpha	97	CCGAC-CAAGAC--CTGGCGGGGTACTTCTCAAA-GGCAA	132
25.MAFF244605_FusariumoxysporumSchlechtendal_MAFF244605_Tomato	98	CCGAC-CAAGAC--CTGGCGGGGTATTTCTCAAA-GGCAA	133
26.MAFF241326_F.asiaticum_Wheat	79	CCGCC--GACAC--TTGGCGGGGTAGTTTCAAATTTCCAA	114
27.MAFF245014_F.asiaticum_Wildsoybean	76	CCGCC--GACAC--TTGGCGGGGTAGTTTCAAATTTCCAA	111
28.MAFF150124_F.asiaticum__Wheat	76	CCGCC--GACAC--TTGGCGGGGTAGTTTCAAATTTCCAA	111
29.OM135603.1F.algeriense	108	CCGCCTCTAAAT-------GGGGTCTACCTGAAG-CGCAA	139
30.MAFF237465_F.penzigii_Aloe	108	CCCGC-CATCTCATCTGGTGGGAGTTTAATCAA----CAA	142
31.MAFF103054_F.oxysporumSchlechtendalf.sp.cucumerinum_Cucumber	97	CCGAC-CAAGAC--CTGGCGGGGTATTTCTCAAA-GTCAA	132
32.MAFF712246_F.oxysporumSchlechtendalf.sp.dianthi__Carnation	97	CCGAC-CAAGAC--CTGGCGGGGTATTTCTCAAA-GTCAA	132
33.MAFF305558_F.oxysporumSchlechtendalf.sp.fragariae__Watermelon	97	CCGAC-CAAGAC--CTGGCGGGGTATTTCTCAAA-GTCAA	132
34.MAFF744087_F.oxysporumSchlechtendalf.sp.lactucae__Lettuce	97	CCGAC-CAAGAC--CTGGCGGGGTATTTCTCAAA-GTCAA	132
35.MAFF726924_F.oxysporumSchlechtendalf.sp.lagenariae_Whitefloweredgourd	97	CCGAC-CAAGAC--CTGGCGGGGTATTTCTCAAA-GTCAA	132
36.MAFF744003_F.oxysporumSchlechtendalf.sp.lagenariae_Squash)	97	CCGAC-CAAGAC--CTGGCAGGGTATTTCTCAAA-GTCAA	132
37.MAFF305122_F.oxysporumSchlechtendalf.sp.melonis__Melon	97	CCGAC-CAAGAC--CTGGCGGGGTATTTCTCAAA-GTCAA	132
38.MAFF306714_F.oxysporumSchlechtendalf.sp.momordicae_Balsampear	97	CCGAC-CAAGAC--CTGGCGGGGTATTTCTCAAA-GTCAA	132
39.MAFF238905_F.oxysporumSchlechtendalf.sp.radicis-lycopersici_Tomato	97	CCGAC-CAAGAC--CTGGCGGGGTATTTCTCAAA-GTCAA	132
40.MAFF150004_F.oxysporumSchlechtendalf.sp.spinaciae_Spinach	97	CCGAC-CAAGAC--CTGGCGGGGTATTTCTCAAA-GTCAA	132
41.MAFF247034_F.oxysporumSchlechtendal__Goldenchain	97	CCGAC-CAAGAC--CTGGCGGGGTATTTCTCAAA-GTCAA	132
42.MAFF245747_F.oxysporumSchlechtendalf.sp.callistephi__Chinaaster	97	CCGAC-CAAGAC--CTGGCGGGGTATTTCTCAAA-GTCAA	132
43.MAFF305115_FoxysporumSchlechtendalf.sp.batatas__Sweatpotato	99	CCCGCCTGGCATCTCGGGCGGGGTATTCATCATT-CACTT	137
44.MAFF150126_F.asiaticum_Seed	76	CCGCC--GACAC--TTGGCGGGGTAGTTTCAAATTTCCAA	111
45.MAFF246738_F.solani_Angelica	99	CCCGCCTGGCATCTCGGGCGGGGTATTCATCATT-CACTT	137
46.MAFF246664_F.cugenangense_Perilla	97	CCGAC-CAAGAC--CTGGCGGGGTATTTCTCAAA-GTCAA	132
47.MH582420.1F.solanistrainMRC256	88	CCCGCCTGGCATCTCGGGCGGGGTATTCATCATT-CACTT	126
48.MAFF240361_F.babinda_Soil	97	TCGCCTTTAGAT--ATGGCGGGGTATGCCTCAAAACGCAA	134
49.MAFF242368_F.azukicola_Azukibean	100	CCCGCCCTCGGACTCGGGCGGGGTCTTCATCGGT-CACTC	138
50.MAFF241312_F.asiaticum_Soil,welshonionfield	76	CCGCC--GACAC--TTGGCGGGGTAGTTTCAAATTTCCAA	111
51.LT548416.1_F.culmorumpartialtef1a	72	CCGCC--GATAC--TTGGCGGGGTAGTTTCAAATTTCCAA	107
52.MAFF150124_F.asiaticum__Wheat	76	CCGCC--GACAC--TTGGCGGGGTAGTTTCAAATTTCCAA	111
53.MAFF238806_F.begoniae_Oncidiumsp	115	CCGAC-CAAGAC--CTGGCGGGGTA--TCTCAAA-GATAA	148

```
54.MW594399.1_FusariumincarnatumisolateUD01C                                 105 CCGCC---AGAT--GTGGCGGGGTAATTTCAACTTGAATA 139
55.OP414923.1Pucciniagraminisf.sp.triticiisolateSHZPgt19                       0 ---------------------------------------- 0
56.MT027094.1_BipolarisoryzaestrainOrL-2                                       0 ---------------------------------------- 0
57.ON734360.1_AlternariaalternataisolateH126                                   0 ---------------------------------------- 0
58.LC333578.1_StemphyliumlycopersiciSOasp2                                     0 ---------------------------------------- 0
59.HQ718583.1_Colletotrichumgloeosporioidesisolateq-1                        100 GCTAACGTTTTTTTTTTCCAGGGTCACTTGATCTACCAGT 139
60.JN241603.1_AtheliarolfsiiisolateSR1                                         0 ---------------------------------------- 0
61.KJ866474.1_RhizoctoniasolanistrainMHL-1                                     0 ---------------------------------------- 0
62.JQ672424.1AlternariatriticinaisolateEGS17-061                               0 ---------------------------------------- 0
63.LT707559.1_P.capsicipartialtef1agene                                        0 ---------------------------------------- 0
64.MW090051.1_CurvularialunatastrainCls-3                                      0 ---------------------------------------- 0
65.DQ400892.1_Aspergillusterreus                                               0 ---------------------------------------- 0
66.DQ911416.1_Pythiumsp.quercumstrainPy292                                     0 ---------------------------------------- 0
67.EU797495.1_Phytophthorasp.oaksoilPoland                                     0 ---------------------------------------- 0
68.HM148321.1_Cladosporiumcucumerinum                                          0 ---------------------------------------- 0
69.AF398888.1_SclerotiniasclerotiorumisolateSS1                                0 ---------------------------------------- 0
70.AF398888.1_S.sclerotiorumisolateSS1                                         0 ---------------------------------------- 0
71.HPAB545908.1_Verticilliumnonalfalfaeisolate                                 0 ---------------------------------------- 0
72.EF433315.1_CeratocystisfimbriatavoucherCMW15052                            46 TTGTGGACAGCATGCAATTGGTGTTATTGAGCTCTACTTT 85
73.MN159912.1_Botrytiscinerea                                                  0 ---------------------------------------- 0
74.MF034741.1_PeltasterfructicolaisolateSRB92                                  0 ---------------------------------------- 0
75.LC440360.1_CercosporaasparagiCOasp2                                         0 ---------------------------------------- 0
76.AY944105.1_MagnaportheoryzaeisolateSAG00T3()                                0 ---------------------------------------- 0
77.JX266586.1_CochliobolusmiyabeanusvoucherMFLUCC10-0733                       0 ---------------------------------------- 0
78.MN393253.1_CorynesporacassiicolaisolateQHD001(MN393253.1UNVERIFIED)         0 ---------------------------------------- 0
79.MF375218.1_AgroatheliarolfsiiisolateBJB24                                   0 ---------------------------------------- 0
80.MN106270.1_AgroatheliarolfsiistrainJ-12                                     0 ---------------------------------------- 0
81.OQ732628.1_AgroatheliarolfsiiisolateBTCBSr3                                 0 ---------------------------------------- 0
82.KY196185.1_ColletotrichumtruncatumstrainPAK53                              76 ---------------------GGTCACTTGATCTACCAGT 94
83.GU935835.1_ColletotrichumcoccodesisolateC96002                              0 ---------------------------------------- 0
84.MK085963.1_AlternariatenuissimaisolateSCCZ06                                0 ---------------------------------------- 0
85.MT548042.1_AlternarialongipesstrainKY_2019_012                              0 ---------------------------------------- 0
86.MN356465.1CalonectriamontanaisolateHSP4                                     0 ---------------------------------------- 0
87.OL694224.1_CalonectriacanadianastrainF099                                   0 ---------------------------------------- 0
88.MK803351.1_NeoscytalidiumdimidiatumstrainKale4-C                            0 ---------------------------------------- 0
89.ON376993.1_CurvulariachiangmaiensisisolateND00J7                            0 ---------------------------------------- 0
90.OQ383346.1_NeoscytalidiumdimidiatumisolateGKH-2                             0 ---------------------------------------- 0
91.MF662595.1_NeoscytalidiumnovaehollandiaeisolateNeNo1                        0 ---------------------------------------- 0
92.EF560588.1Melampsoralini                                                    0 ---------------------------------------- 0
93.LC590862.1_NeoscytalidiumdimidiatumPSU-HP01TEF1                             0 ---------------------------------------- 0
94.KX278106.1_BotryosphaeriaqingyuanensisstrainCERC2947                        0 ---------------------------------------- 0
95.AJ578763.1_Blumeriagraminisf.sp.hordeicyp51                                 0 ---------------------------------------- 0
96.MF490858.1_CurvulariadactylocteniicolastrainCPC28810                        0 ---------------------------------------- 0
97.KT287115.1_Bipolariscactivoraisolate3.8.6                                   0 ---------------------------------------- 0
98.MT560940.1_CurvulariacactivorastrainHLGH0118                                0 ---------------------------------------- 0
99.OM714565.1_CurvulariaplantarumstrainM0134                                   0 ---------------------------------------- 0
```

```
100.MN159911.1_BotrytiscinereaSICAUCC19-0003                            0 ---------------------------------------- 0
102.GU294713.1LasiodiplodiatheobromaestrainUCD2430TX                    0 ---------------------------------------- 0
103.KX868094.1_Mycosphaerellasp.isolateCRM20.1                          0 ---------------------------------------- 0
104.LC599478.1Pseudocercosporapini-densifloraeMUCC534                   0 ---------------------------------------- 0
105.N584698.1BipolarissetariaestrainKBS4-2                              0 ---------------------------------------- 0

2.OM160859.1_F.buharicum                                              105 CATGCTGACATTTTTATACAGACC---------------- 128
1.LC727524.1_F.buharicum_OKI-1_Okura                                  136 CATGCTGACATTTTTATACAGACC---------------- 159
3.KX302919.1_F.sublunatum                                             102 CATGCTAACAACTTTGGATAGACC---------------- 125
4.LT996094.1_F.convolutans                                            102 CATGCTAACAGCTTTGGATAGACC---------------- 125
5.OM160861.1_F.abutilonis                                             104 CATGCTGACAACTTTATACAGACC---------------- 127
6.OM160874.1_F.guadeloupense                                          104 CTTGCTAATGCATTCA-ACAGACC---------------- 126
7.MH392475.1_F.graminearum                                             57 TGTGCTGACATACTTTGATAGACC---------------- 80
8.MH582420.1_F.solani                                                 107 CATGCTGACAATCATCTACAGACC---------------- 130
9.MAFF244605_F.oxysporum                                              103 TATGCTGATATCGTTTCACAGACC---------------- 126
10.MAFF237278_F.contaminatum_Hylocereus                               150 CATACTGACATCGTTTCACAGACC---------------- 173
11.MAFF237649_F.concentricum__Riceroot                                143 CATGCTGACATAGCTTCACAGACC---------------- 166
12.MAFF237650_F.concentricum__Wheat                                   143 CATGCTGACATAGCTTCACAGACC---------------- 166
13.MAFF239869_F.mangiferae__Ryukyupine                                133 TATGCTGACATAGCTTCAAAGACC---------------- 156
14.MAFF240460_F.fujikuroi_Passionfruit                                133 TATGCTGACATCGCTTCACAGACC---------------- 156
15.MAFF241317_F.graminearum_Wheat                                     112 TGTGCTGACATACTTTGATAGACC---------------- 135
16.MAFF242670_F.ipomoeae_Wheat                                        135 TTTGCTGACAAGATTGCATAGACC---------------- 158
17.MAFF245129_F.concentricum_Fraxinus                                 133 CATGCTGACATAGCTTCACAGACC---------------- 156
18.MAFF245395_F.cugenangense_Rhubarb                                  133 CATACTGACATCGTTTCACAGACC---------------- 156
19.MAFF246637_F.nirenbergiae_Strawberry                               133 CATACTGACATCGTTTCACAGACC---------------- 156
20.MAFF246672_F.nirenbergiae_ChinesePeony                             133 CATACTGACATCGTTTCACAGACC---------------- 156
21.MAFF246697_F.commune_Urallicoriceroot                              134 TATGCTGATATCGTTTCACAGACC---------------- 157
22.MAFF246729_F.falciforme_Angelica                                   138 CATGCTGACAATCATCTACAGACC---------------- 161
23.MAFF247220_F.duplospermum__Euwallaceasp                            138 CATGCTGACAATCATTTACAGACC---------------- 161
24.MAFF410760_F.odoratissimum_alpha                                   133 CATACTGACATCGTTTCACAGACC---------------- 156
25.MAFF244605_FusariumoxysporumSchlechtendal_MAFF244605_Tomato        134 TATGCTGATATCGTTTCACAGACC---------------- 157
26.MAFF241326_F.asiaticum_Wheat                                       115 TGTGCTGACATACTTTGATAGACC---------------- 138
27.MAFF245014_F.asiaticum_Wildsoybean                                 112 TGTGCTGACATACTTTGATAGACC---------------- 135
28.MAFF150124_F.asiaticum__Wheat                                      112 TGTGCTGACATACTTTGATAGACC---------------- 135
29.OM135603.1F.algeriense                                             140 CATGCTGACATTCTTTGACAGACC---------------- 163
30.MAFF237465_F.penzigii_Aloe                                         143 TATGCTGACATCTACATCTAGACC---------------- 166
31.MAFF103054_F.oxysporumSchlechtendalf.sp.cucumerinum_Cucumber       133 CATACTGACATCGTTTCACAGACC---------------- 156
32.MAFF712246_F.oxysporumSchlechtendalf.sp.dianthi__Carnation         133 CATACTGACATCGTTTCACAGACC---------------- 156
33.MAFF305558_F.oxysporumSchlechtendalf.sp.fragariae__Watermelon      133 CATACTGACATCGTTTCACAGACC---------------- 156
34.MAFF744087_F.oxysporumSchlechtendalf.sp.lactucae__Lettuce          133 CATACTGACATCGTTTCACAGACC---------------- 156
35.MAFF726924_F.oxysporumSchlechtendalf.sp.lagenariae_Whitefloweredgourd 133 CATACTGACATCGTTTCACAGACC---------------- 156
36.MAFF744003_F.oxysporumSchlechtendalf.sp.lagenariae_Squash)         133 CATACTGACATCGTTTCACAGACC---------------- 156
37.MAFF305122_F.oxysporumSchlechtendalf.sp.melonis__Melon             133 CATACTGACATCGTTTCACAGACC---------------- 156
38.MAFF306714_F.oxysporumSchlechtendalf.sp.momordicae_Balsampear      133 CATACTGACATCGTTTCACAGACC---------------- 156
39.MAFF238905_F.oxysporumSchlechtendalf.sp.radicis-lycopersici_Tomato 133 CATACTGACATCGTTTCACAGACC---------------- 156
40.MAFF150004_F.oxysporumSchlechtendalf.sp.spinaciae_Spinach          133 CATACTGACATCGTTTCACAGACC---------------- 156
```

41.MAFF247034_F.oxysporumSchlechtendal__Goldenchain	133	CATACTGACATCGTTTCACAGACC----------------	156
42.MAFF245747_F.oxysporumSchlechtendalf.sp.callistephi__Chinaaster	133	CATACTGACATCGTTTCACAGACC----------------	156
43.MAFF305115_FoxysporumSchlechtendalf.sp.batatas__Sweatpotato	138	CATGCTGACAATCATCTACAGACC----------------	161
44.MAFF150126_F.asiaticum_Seed	112	TGTGCTGACATACTTTGATAGACC----------------	135
45.MAFF246738_F.solani_Angelica	138	CATGCTGACAATCATCTACAGACC----------------	161
46.MAFF246664_F.cugenangense_Perilla	133	CATACTGACATCGTTTCACAGACC----------------	156
47.MH582420.1F.solanistrainMRC256	127	CATGCTGACAATCATCTACAGACC----------------	150
48.MAFF240361_F.babinda_Soil	135	CATGCTGACATCCTTTAACAGACC----------------	158
49.MAFF242368_F.azukicola_Azukibean	139	CATGCTGACAATCAATCACAGACC----------------	162
50.MAFF241312_F.asiaticum_Soil,welshonionfield	112	TGTGCTGACATACTTTGATAGACC----------------	135
51.LT548416.1_F.culmorumpartialtef1a	108	TGTGCTGACATACTTTGATAGACC----------------	131
52.MAFF150124_F.asiaticum__Wheat	112	TGTGCTGACATACTTTGATAGACC----------------	135
53.MAFF238806_F.begoniae_Oncidiumsp	149	CATGCTGACATCGCTCCACAGACC----------------	172
54.MW594399.1_FusariumincarnatumisolateUD01C	140	TTTGCTGACAAGATTGCATAGACC----------------	163
55.OP414923.1Pucciniagraminisf.sp.triticiisolateSHZPgt19	0	--	0
56.MT027094.1_BipolarisoryzaestrainOrL-2	0	--	0
57.ON734360.1_AlternariaalternataisolateH126	0	--	0
58.LC333578.1_StemphyliumlycopersiciSOasp2	0	--	0
59.HQ718583.1_Colletotrichumgloeosporioidesisolateq-1	140	GCGGCGGTATCGACAAGAGAACGATTGAGAAGTTCGAGAA	179
60.JN241603.1_AtheliarolfsiiisolateSR1	0	--	0
61.KJ866474.1_RhizoctoniasolanistrainMHL-1	0	--	0
62.JQ672424.1AlternariatriticinaisolateEGS17-061	0	--	0
63.LT707559.1_P.capsicipartialtef1agene	0	--	0
64.MW090051.1_CurvularialunatastrainCls-3	0	--	0
65.DQ400892.1_Aspergillusterreus	0	--	0
66.DQ911416.1_Pythiumsp.quercumstrainPy292	0	--	0
67.EU797495.1_Phytophthorasp.oaksoilPoland	0	--	0
68.HM148321.1_Cladosporiumcucumerinum	0	--	0
69.AF398888.1_SclerotiniasclerotiorumisolateSS1	0	--	0
70.AF398888.1_S.sclerotiorumisolateSS1	0	--	0
71.HPAB545908.1_Verticilliumnonalfalfaeisolate	0	--	0
72.EF433315.1_CeratocystisfimbriatavoucherCMW15052	86	TTTGATGACGCTTTTCCCTCTGTTCTTCTGGCCATCGAAG	125
73.MN159912.1_Botrytiscinerea	0	--	0
74.MF034741.1_PeltasterfructicolaisolateSRB92	0	--	0
75.LC440360.1_CercosporaasparagiCOasp2	0	--	0
76.AY944105.1_MagnaportheoryzaeisolateSAG00T3()	0	--	0
77.JX266586.1_CochliobolusmiyabeanusvoucherMFLUCC10-0733	0	--	0
78.MN393253.1_CorynesporacassiicolaisolateQHD001(MN393253.1UNVERIFIED)	0	--	0
79.MF375218.1_AgroatheliarolfsiiisolateBJB24	0	--	0
80.MN106270.1_AgroatheliarolfsiistrainJ-12	0	--	0
81.OQ732628.1_AgroatheliarolfsiiisolateBTCBSr3	0	--	0
82.KY196185.1_ColletotrichumtruncatumstrainPAK53	95	GCGGTGGTATCGACAAGCGTACCATCGAGAAGTTCGAGAA	134
83.GU935835.1_ColletotrichumcoccodesisolateC96002	0	--	0
84.MK085963.1_AlternariatenuissimaisolateSCCZ06	0	--	0
85.MT548042.1_AlternarialongipesstrainKY_2019_012	0	--	0
86.MN356465.1CalonectriamontanaisolateHSP4	0	--	0

```
87.OL694224.1_CalonectriacanadianastrainF099                 0 ---------------------------------------- 0
88.MK803351.1_NeoscytalidiumdimidiatumstrainKale4-C          0 ---------------------------------------- 0
89.ON376993.1_CurvulariachiangmaiensisisolateND00J7          0 ---------------------------------------- 0
90.OQ383346.1_NeoscytalidiumdimidiatumisolateGKH-2           0 ---------------------------------------- 0
91.MF662595.1_NeoscytalidiumnovaehollandiaeisolateNeNo1      0 ---------------------------------------- 0
92.EF560588.1Melampsoralini                                  0 ---------------------------------------- 0
93.LC590862.1_NeoscytalidiumdimidiatumPSU-HP01TEF1           0 ---------------------------------------- 0
94.KX278106.1_BotryosphaeriaqingyuanensisstrainCERC2947      0 ---------------------------------------- 0
95.AJ578763.1_Blumeriagraminisf.sp.hordeicyp51               0 ---------------------------------------- 0
96.MF490858.1_CurvulariadactylocteniicolastrainCPC28810      0 ---------------------------------------- 0
97.KT287115.1_Bipolariscactivoraisolate3.8.6                 0 ---------------------------------------- 0
98.MT560940.1_CurvulariacactivorastrainHLGH0118              0 ---------------------------------------- 0
99.OM714565.1_CurvulariaplantarumstrainM0134                 0 ---------------------------------------- 0
100.MN159911.1_BotrytiscinereaSICAUCC19-0003                 0 ---------------------------------------- 0
102.GU294713.1LasiodiplodiatheobromaestrainUCD2430TX         0 ---------------------------------------- 0
103.KX868094.1_Mycosphaerellasp.isolateCRM20.1               0 ---------------------------------------- 0
104.LC599478.1Pseudocercosporapini-densifloraeMUCC534        0 ---------------------------------------- 0
105.N584698.1BipolarissetariaestrainKBS4-2                   0 ---------------------------------------- 0

2.OM160859.1_F.buharicum                                   128 ---------------------------------------- 128
1.LC727524.1_F.buharicum_OKI-1_Okura                       159 ---------------------------------------- 159
3.KX302919.1_F.sublunatum                                  125 ---------------------------------------- 125
4.LT996094.1_F.convolutans                                 125 ---------------------------------------- 125
5.OM160861.1_F.abutilonis                                  127 ---------------------------------------- 127
6.OM160874.1_F.guadeloupense                               126 ---------------------------------------- 126
7.MH392475.1_F.graminearum                                  80 ---------------------------------------- 80
8.MH582420.1_F.solani                                      130 ---------------------------------------- 130
9.MAFF244605_F.oxysporum                                   126 ---------------------------------------- 126
10.MAFF237278_F.contaminatum_Hylocereus                    173 ---------------------------------------- 173
11.MAFF237649_F.concentricum__Riceroot                     166 ---------------------------------------- 166
12.MAFF237650_F.concentricum__Wheat                        166 ---------------------------------------- 166
13.MAFF239869_F.mangiferae__Ryukyupine                     156 ---------------------------------------- 156
14.MAFF240460_F.fujikuroi_Passionfruit                     156 ---------------------------------------- 156
15.MAFF241317_F.graminearum_Wheat                          135 ---------------------------------------- 135
16.MAFF242670_F.ipomoeae_Wheat                             158 ---------------------------------------- 158
17.MAFF245129_F.concentricum_Fraxinus                      156 ---------------------------------------- 156
18.MAFF245395_F.cugenangense_Rhubarb                       156 ---------------------------------------- 156
19.MAFF246637_F.nirenbergiae_Strawberry                    156 ---------------------------------------- 156
20.MAFF246672_F.nirenbergiae_ChinesePeony                  156 ---------------------------------------- 156
21.MAFF246697_F.commune_Urallicoriceroot                   157 ---------------------------------------- 157
22.MAFF246729_F.falciforme_Angelica                        161 ---------------------------------------- 161
23.MAFF247220_F.duplospermum__Euwallaceasp                 161 ---------------------------------------- 161
24.MAFF410760_F.odoratissimum_alpha                        156 ---------------------------------------- 156
25.MAFF244605_FusariumoxysporumSchlechtendal_MAFF244605_Tomato 157 ---------------------------------------- 157
26.MAFF241326_F.asiaticum_Wheat                            138 ---------------------------------------- 138
27.MAFF245014_F.asiaticum_Wildsoybean                      135 ---------------------------------------- 135
```

```
28.MAFF150124_F.asiaticum__Wheat                                135 ---------------------------------------- 135
29.OM135603.1F.algeriense                                       163 ---------------------------------------- 163
30.MAFF237465_F.penzigii_Aloe                                   166 ---------------------------------------- 166
31.MAFF103054_F.oxysporumSchlechtendalf.sp.cucumerinum_Cucumber 156 ---------------------------------------- 156
32.MAFF712246_F.oxysporumSchlechtendalf.sp.dianthi__Carnation   156 ---------------------------------------- 156
33.MAFF305558_F.oxysporumSchlechtendalf.sp.fragariae__Watermelon 156 ---------------------------------------- 156
34.MAFF744087_F.oxysporumSchlechtendalf.sp.lactucae__Lettuce    156 ---------------------------------------- 156
35.MAFF726924_F.oxysporumSchlechtendalf.sp.lagenariae_WhiteflOweredgourd 156 ---------------------------------------- 156
36.MAFF744003_F.oxysporumSchlechtendalf.sp.lagenariae_Squash)   156 ---------------------------------------- 156
37.MAFF305122_F.oxysporumSchlechtendalf.sp.melonis__Melon       156 ---------------------------------------- 156
38.MAFF306714_F.oxysporumSchlechtendalf.sp.momordicae_Balsampear 156 ---------------------------------------- 156
39.MAFF238905_F.oxysporumSchlechtendalf.sp.radicis-lycopersici_Tomato 156 ---------------------------------------- 156
40.MAFF150004_F.oxysporumSchlechtendalf.sp.spinaciae_Spinach    156 ---------------------------------------- 156
41.MAFF247034_F.oxysporumSchlechtendal__Goldenchain             156 ---------------------------------------- 156
42.MAFF245747_F.oxysporumSchlechtendalf.sp.callistephi__Chinaaster 156 ---------------------------------------- 156
43.MAFF305115_FoxysporumSchlechtendalf.sp.batatas__Sweatpotato  161 ---------------------------------------- 161
44.MAFF150126_F.asiaticum_Seed                                  135 ---------------------------------------- 135
45.MAFF246738_F.solani_Angelica                                 161 ---------------------------------------- 161
46.MAFF246664_F.cugenangense_Perilla                            156 ---------------------------------------- 156
47.MH582420.1F.solanistrainMRC256                               150 ---------------------------------------- 150
48.MAFF240361_F.babinda_Soil                                    158 ---------------------------------------- 158
49.MAFF242368_F.azukicola_Azukibean                             162 ---------------------------------------- 162
50.MAFF241312_F.asiaticum_Soil,welshonionfield                  135 ---------------------------------------- 135
51.LT548416.1_F.culmorumpartialtef1a                            131 ---------------------------------------- 131
52.MAFF150124_F.asiaticum__Wheat                                135 ---------------------------------------- 135
53.MAFF238806_F.begoniae_Oncidiumsp                             172 ---------------------------------------- 172
54.MW594399.1_FusariumincarnatumisolateUD01C                    163 ---------------------------------------- 163
55.OP414923.1Pucciniagraminisf.sp.triticiisolateSHZPgt19          1 ---------------------------------------A 1
56.MT027094.1_BipolarisoryzaestrainOrL-2                          0 ---------------------------------------- 0
57.ON734360.1_AlternariaalternataisolateH126                      0 ---------------------------------------- 0
58.LC333578.1_StemphyliumlycopersiciSOasp2                        0 ---------------------------------------- 0
59.HQ718583.1_Colletotrichumgloeosporioidesisolateq-1           180 GGAGGCTGCCGAGCTGCAAGTAC--CACCTT-TCCATCAC 216
60.JN241603.1_AtheliarolfsiiisolateSR1                            0 ---------------------------------------- 0
61.KJ866474.1_RhizoctoniasolanistrainMHL-1                        0 ---------------------------------------- 0
62.JQ672424.1AlternariatriticinaisolateEGS17-061                  0 ---------------------------------------- 0
63.LT707559.1_P.capsicipartialtef1agene                           0 ---------------------------------------- 0
64.MW090051.1_CurvularialunatastrainCls-3                         0 ---------------------------------------- 0
65.DQ400892.1_Aspergillusterreus                                  0 ---------------------------------------- 0
66.DQ911416.1_Pythiumsp.quercumstrainPy292                        0 ---------------------------------------- 0
67.EU797495.1_Phytophthorasp.oaksoilPoland                        0 ---------------------------------------- 0
68.HM148321.1_Cladosporiumcucumerinum                             0 ---------------------------------------- 0
69.AF398888.1_SclerotiniasclerotiorumisolateSS1                   0 ---------------------------------------- 0
70.AF398888.1_S.sclerotiorumisolateSS1                            0 ---------------------------------------- 0
71.HPAB545908.1_Verticilliumnonalfalfaeisolate                    0 ---------------------------------------- 0
72.EF433315.1_CeratocystisfimbriatavoucherCMW15052              126 GGCGGGGTAGCGTCACTGAAGTGGGGCTGCTATTTTTTCT 165
73.MN159912.1_Botrytiscinerea                                     0 ---------------------------------------- 0
```

```
74.MF034741.1_PeltasterfructicolaisolateSRB92                              0 ---------------------------------------- 0
75.LC440360.1_CercosporaasparagiCOasp2                                     0 ---------------------------------------- 0
76.AY944105.1_MagnaportheoryzaeisolateSAG00T3()                            0 ---------------------------------------- 0
77.JX266586.1_CochliobolusmiyabeanusvoucherMFLUCC10-0733                   0 ---------------------------------------- 0
78.MN393253.1_CorynesporacassiicolaisolateQHD001(MN393253.1UNVERIFIED)     0 ---------------------------------------- 0
79.MF375218.1_AgroatheliarolfsiiisolateBJB24                               0 ---------------------------------------- 0
80.MN106270.1_AgroatheliarolfsiistrainJ-12                                 0 ---------------------------------------- 0
81.OQ732628.1_AgroatheliarolfsiiisolateBTCBSr3                             0 ---------------------------------------- 0
82.KY196185.1_ColletotrichumtruncatumstrainPAK53                         135 GGAGGCCGCCGAGTTGGTATGATTTCACATCATCGATGTT 174
83.GU935835.1_ColletotrichumcoccodesisolateC96002                          0 ---------------------------------------- 0
84.MK085963.1_AlternariatenuissimaisolateSCCZ06                            0 ---------------------------------------- 0
85.MT548042.1_AlternarialongipesstrainKY_2019_012                          0 ---------------------------------------- 0
86.MN356465.1CalonectriamontanaisolateHSP4                                 0 ---------------------------------------- 0
87.OL694224.1_CalonectriacanadianastrainF099                               0 ---------------------------------------- 0
88.MK803351.1_NeoscytalidiumdimidiatumstrainKale4-C                        0 ---------------------------------------- 0
89.ON376993.1_CurvulariachiangmaiensisisolateND00J7                        0 ---------------------------------------- 0
90.OQ383346.1_NeoscytalidiumdimidiatumisolateGKH-2                         0 ---------------------------------------- 0
91.MF662595.1_NeoscytalidiumnovaehollandiaeisolateNeNo1                    0 ---------------------------------------- 0
92.EF560588.1Melampsoralini                                                1 -------------------------AAGCGAACCATTGAG 15
93.LC590862.1_NeoscytalidiumdimidiatumPSU-HP01TEF1                         0 ---------------------------------------- 0
94.KX278106.1_BotryosphaeriaqingyuanensisstrainCERC2947                    0 ---------------------------------------- 0
95.AJ578763.1_Blumeriagraminisf.sp.hordeicyp51                             0 ---------------------------------------- 0
96.MF490858.1_CurvulariadactylocteniicolastrainCPC28810                    0 ---------------------------------------- 0
97.KT287115.1_Bipolariscactivoraisolate3.8.6                               0 ---------------------------------------- 0
98.MT560940.1_CurvulariacactivorastrainHLGH0118                            0 ---------------------------------------- 0
99.OM714565.1_CurvulariaplantarumstrainM0134                               0 ---------------------------------------- 0
100.MN159911.1_BotrytiscinereaSICAUCC19-0003                               0 ---------------------------------------- 0
102.GU294713.1LasiodiplodiatheobromaestrainUCD2430TX                       0 ---------------------------------------- 0
103.KX868094.1_Mycosphaerellasp.isolateCRM20.1                             0 ---------------------------------------- 0
104.LC599478.1Pseudocercosporapini-densifloraeMUCC534                      0 ---------------------------------------- 0
105.N584698.1BipolarissetariaestrainKBS4-2                                 0 ---------------------------------------- 0

2.OM160859.1_F.buharicum                                                 128 ---------------------------------------- 128
1.LC727524.1_F.buharicum_OKI-1_Okura                                     159 ---------------------------------------- 159
3.KX302919.1_F.sublunatum                                                125 ---------------------------------------- 125
4.LT996094.1_F.convolutans                                               125 ---------------------------------------- 125
5.OM160861.1_F.abutilonis                                                127 ---------------------------------------- 127
6.OM160874.1_F.guadeloupense                                             126 ---------------------------------------- 126
7.MH392475.1_F.graminearum                                                80 ---------------------------------------- 80
8.MH582420.1_F.solani                                                    130 ---------------------------------------- 130
9.MAFF244605_F.oxysporum                                                 126 ---------------------------------------- 126
10.MAFF237278_F.contaminatum_Hylocereus                                  173 ---------------------------------------- 173
11.MAFF237649_F.concentricum__Riceroot                                   166 ---------------------------------------- 166
12.MAFF237650_F.concentricum__Wheat                                      166 ---------------------------------------- 166
13.MAFF239869_F.mangiferae__Ryukyupine                                   156 ---------------------------------------- 156
14.MAFF240460_F.fujikuroi_Passionfruit                                   156 ---------------------------------------- 156
```

```
15.MAFF241317_F.graminearum_Wheat                                  135 ---------------------------------------- 135
16.MAFF242670_F.ipomoeae_Wheat                                     158 ---------------------------------------- 158
17.MAFF245129_F.concentricum_Fraxinus                              156 ---------------------------------------- 156
18.MAFF245395_F.cugenangense_Rhubarb                               156 ---------------------------------------- 156
19.MAFF246637_F.nirenbergiae_Strawberry                            156 ---------------------------------------- 156
20.MAFF246672_F.nirenbergiae_ChinesePeony                          156 ---------------------------------------- 156
21.MAFF246697_F.commune_Urallicoriceroot                           157 ---------------------------------------- 157
22.MAFF246729_F.falciforme_Angelica                                161 ---------------------------------------- 161
23.MAFF247220_F.duplospermum__Euwallaceasp                         161 ---------------------------------------- 161
24.MAFF410760_F.odoratissimum_alpha                                156 ---------------------------------------- 156
25.MAFF244605_FusariumoxysporumSchlechtendal_MAFF244605_Tomato     157 ---------------------------------------- 157
26.MAFF241326_F.asiaticum_Wheat                                    138 ---------------------------------------- 138
27.MAFF245014_F.asiaticum_Wildsoybean                              135 ---------------------------------------- 135
28.MAFF150124_F.asiaticum__Wheat                                   135 ---------------------------------------- 135
29.OM135603.1F.algeriense                                          163 ---------------------------------------- 163
30.MAFF237465_F.penzigii_Aloe                                      166 ---------------------------------------- 166
31.MAFF103054_F.oxysporumSchlechtendalf.sp.cucumerinum_Cucumber    156 ---------------------------------------- 156
32.MAFF712246_F.oxysporumSchlechtendalf.sp.dianthi__Carnation      156 ---------------------------------------- 156
33.MAFF305558_F.oxysporumSchlechtendalf.sp.fragariae__Watermelon   156 ---------------------------------------- 156
34.MAFF744087_F.oxysporumSchlechtendalf.sp.lactucae__Lettuce       156 ---------------------------------------- 156
35.MAFF726924_F.oxysporumSchlechtendalf.sp.lagenariae_Whiteflോweredgourd 156 ---------------------------------------- 156
36.MAFF744003_F.oxysporumSchlechtendalf.sp.lagenariae_Squash)      156 ---------------------------------------- 156
37.MAFF305122_F.oxysporumSchlechtendalf.sp.melonis__Melon          156 ---------------------------------------- 156
38.MAFF306714_F.oxysporumSchlechtendalf.sp.momordicae_Balsampear   156 ---------------------------------------- 156
39.MAFF238905_F.oxysporumSchlechtendalf.sp.radicis-lycopersici_Tomato 156 ---------------------------------------- 156
40.MAFF150004_F.oxysporumSchlechtendalf.sp.spinaciae_Spinach       156 ---------------------------------------- 156
41.MAFF247034_F.oxysporumSchlechtendal__Goldenchain                156 ---------------------------------------- 156
42.MAFF245747_F.oxysporumSchlechtendalf.sp.callistephi__Chinaaster 156 ---------------------------------------- 156
43.MAFF305115_FoxysporumSchlechtendalf.sp.batatas__Sweatpotato     161 ---------------------------------------- 161
44.MAFF150126_F.asiaticum_Seed                                     135 ---------------------------------------- 135
45.MAFF246738_F.solani_Angelica                                    161 ---------------------------------------- 161
46.MAFF246664_F.cugenangense_Perilla                               156 ---------------------------------------- 156
47.MH582420.1F.solanistrainMRC256                                  150 ---------------------------------------- 150
48.MAFF240361_F.babinda_Soil                                       158 ---------------------------------------- 158
49.MAFF242368_F.azukicola_Azukibean                                162 ---------------------------------------- 162
50.MAFF241312_F.asiaticum_Soil,welshonionfield                     135 ---------------------------------------- 135
51.LT548416.1_F.culmorumpartialtef1a                               131 ---------------------------------------- 131
52.MAFF150124_F.asiaticum__Wheat                                   135 ---------------------------------------- 135
53.MAFF238806_F.begoniae_Oncidiumsp                                172 ---------------------------------------- 172
54.MW594399.1_FusariumincarnatumisolateUD01C                       163 ---------------------------------------- 163
55.OP414923.1Pucciniagraminisf.sp.triticiisolateSHZPgt19             2 AGGAAGCCGCCGAACTGGGTAAAGGATCCTTCAAGTGAGC 41
56.MT027094.1_BipolarisoryzaestrainOrL-2                             0 ---------------------------------------- 0
57.ON734360.1_AlternariaalternataisolateH126                         0 ---------------------------------------- 0
58.LC333578.1_StemphyliumlycopersiciSOasp2                           0 ---------------------------------------- 0
59.HQ718583.1_Colletotrichumgloeosporioidesisolateq-1              217 ATCATCGTCATTTACTAACAAAAAGATAGTGGCAAGGGCT 256
60.JN241603.1_AtheliarolfsiiisolateSR1                               0 ---------------------------------------- 0
```

```
61.KJ866474.1_RhizoctoniasolanistrainMHL-1                            0 ---------------------------------------- 0
62.JQ672424.1AlternariatriticinaisolateEGS17-061                      0 ---------------------------------------- 0
63.LT707559.1_P.capsicipartialtef1agene                               0 ---------------------------------------- 0
64.MW090051.1_CurvularialunatastrainCls-3                             0 ---------------------------------------- 0
65.DQ400892.1_Aspergillusterreus                                      0 ---------------------------------------- 0
66.DQ911416.1_Pythiumsp.quercumstrainPy292                            0 ---------------------------------------- 0
67.EU797495.1_Phytophthorasp.oaksoilPoland                            0 ---------------------------------------- 0
68.HM148321.1_Cladosporiumcucumerinum                                 0 ---------------------------------------- 0
69.AF398888.1_SclerotiniasclerotiorumisolateSS1                       0 ---------------------------------------- 0
70.AF398888.1_S.sclerotiorumisolateSS1                                0 ---------------------------------------- 0
71.HPAB545908.1_Verticilliumnonalfalfaeisolate                        0 ---------------------------------------- 0
72.EF433315.1_CeratocystisfimbriatavoucherCMW15052                  166 AAATGACGTGCATGCTGTCTCCACTTTCATGTTTCAATTC 205
73.MN159912.1_Botrytiscinerea                                         0 ---------------------------------------- 0
74.MF034741.1_PeltasterfructicolaisolateSRB92                         0 ---------------------------------------- 0
75.LC440360.1_CercosporaasparagiCOasp2                                0 ---------------------------------------- 0
76.AY944105.1_MagnaportheoryzaeisolateSAG00T3()                       0 ---------------------------------------- 0
77.JX266586.1_CochliobolusmiyabeanusvoucherMFLUCC10-0733              0 ---------------------------------------- 0
78.MN393253.1_CorynesporacassiicolaisolateQHD001(MN393253.1UNVERIFIED) 0 ---------------------------------------- 0
79.MF375218.1_AgroatheliarolfsiiisolateBJB24                          0 ---------------------------------------- 0
80.MN106270.1_AgroatheliarolfsiistrainJ-12                            0 ---------------------------------------- 0
81.OQ732628.1_AgroatheliarolfsiiisolateBTCBSr3                        0 ---------------------------------------- 0
82.KY196185.1_ColletotrichumtruncatumstrainPAK53                    175 ACCAA-GACTGGTACTAATAAATTCTACAGGGCAAGGGTT 213
83.GU935835.1_ColletotrichumcoccodesisolateC96002                     0 ---------------------------------------- 0
84.MK085963.1_AlternariatenuissimaisolateSCCZ06                       0 ---------------------------------------- 0
85.MT548042.1_AlternarialongipesstrainKY_2019_012                     0 ---------------------------------------- 0
86.MN356465.1CalonectriamontanaisolateHSP4                            0 ---------------------------------------- 0
87.OL694224.1_CalonectriacanadianastrainF099                          0 ---------------------------------------- 0
88.MK803351.1_NeoscytalidiumdimidiatumstrainKale4-C                   0 ---------------------------------------- 0
89.ON376993.1_CurvulariachiangmaiensisisolateND00J7                   0 ---------------------------------------- 0
90.OQ383346.1_NeoscytalidiumdimidiatumisolateGKH-2                    0 ---------------------------------------- 0
91.MF662595.1_NeoscytalidiumnovaehollandiaeisolateNeNo1               0 ---------------------------------------- 0
92.EF560588.1Melampsoralini                                          16 AAGTTCGAGAAAGAAGCTGCTGAGCTTGGAAAGGGTTCCT 55
93.LC590862.1_NeoscytalidiumdimidiatumPSU-HP01TEF1                    0 ---------------------------------------- 0
94.KX278106.1_BotryosphaeriaqingyuanensisstrainCERC2947               0 ---------------------------------------- 0
95.AJ578763.1_Blumeriagraminisf.sp.hordeicyp51                        0 ---------------------------------------- 0
96.MF490858.1_CurvulariadactylocteniicolastrainCPC28810               0 ---------------------------------------- 0
97.KT287115.1_Bipolariscactivoraisolate3.8.6                          0 ---------------------------------------- 0
98.MT560940.1_CurvulariacactivorastrainHLGH0118                       0 ---------------------------------------- 0
99.OM714565.1_CurvulariaplantarumstrainM0134                          0 ---------------------------------------- 0
100.MN159911.1_BotrytiscinereaSICAUCC19-0003                          0 ---------------------------------------- 0
102.GU294713.1LasiodiplodiatheobromaestrainUCD2430TX                  0 ---------------------------------------- 0
103.KX868094.1_Mycosphaerellasp.isolateCRM20.1                        0 ---------------------------------------- 0
104.LC599478.1Pseudocercosporapini-densifloraeMUCC534                 0 ---------------------------------------- 0
105.N584698.1BipolarissetariaestrainKBS4-2                            0 ---------------------------------------- 0

2.OM160859.1_F.buharicum                                            128 ---------------------------------------- 128
```

1.LC727524.1_F.buharicum_OKI-1_Okura	159	--	159
3.KX302919.1_F.sublunatum	125	--	125
4.LT996094.1_F.convolutans	125	--	125
5.OM160861.1_F.abutilonis	127	--	127
6.OM160874.1_F.guadeloupense	126	--	126
7.MH392475.1_F.graminearum	80	--	80
8.MH582420.1_F.solani	130	--	130
9.MAFF244605_F.oxysporum	126	--	126
10.MAFF237278_F.contaminatum_Hylocereus	173	--	173
11.MAFF237649_F.concentricum__Riceroot	166	--	166
12.MAFF237650_F.concentricum__Wheat	166	--	166
13.MAFF239869_F.mangiferae__Ryukyupine	156	--	156
14.MAFF240460_F.fujikuroi_Passionfruit	156	--	156
15.MAFF241317_F.graminearum_Wheat	135	--	135
16.MAFF242670_F.ipomoeae_Wheat	158	--	158
17.MAFF245129_F.concentricum_Fraxinus	156	--	156
18.MAFF245395_F.cugenangense_Rhubarb	156	--	156
19.MAFF246637_F.nirenbergiae_Strawberry	156	--	156
20.MAFF246672_F.nirenbergiae_ChinesePeony	156	--	156
21.MAFF246697_F.commune_Urallicoriceroot	157	--	157
22.MAFF246729_F.falciforme_Angelica	161	--	161
23.MAFF247220_F.duplospermum__Euwallaceasp	161	--	161
24.MAFF410760_F.odoratissimum_alpha	156	--	156
25.MAFF244605_FusariumoxysporumSchlechtendal_MAFF244605_Tomato	157	--	157
26.MAFF241326_F.asiaticum_Wheat	138	--	138
27.MAFF245014_F.asiaticum_Wildsoybean	135	--	135
28.MAFF150124_F.asiaticum__Wheat	135	--	135
29.OM135603.1F.algeriense	163	--	163
30.MAFF237465_F.penzigii_Aloe	166	--	166
31.MAFF103054_F.oxysporumSchlechtendalf.sp.cucumerinum_Cucumber	156	--	156
32.MAFF712246_F.oxysporumSchlechtendalf.sp.dianthi__Carnation	156	--	156
33.MAFF305558_F.oxysporumSchlechtendalf.sp.fragariae__Watermelon	156	--	156
34.MAFF744087_F.oxysporumSchlechtendalf.sp.lactucae__Lettuce	156	--	156
35.MAFF726924_F.oxysporumSchlechtendalf.sp.lagenariae_Whitefloweredgourd	156	--	156
36.MAFF744003_F.oxysporumSchlechtendalf.sp.lagenariae_Squash)	156	--	156
37.MAFF305122_F.oxysporumSchlechtendalf.sp.melonis__Melon	156	--	156
38.MAFF306714_F.oxysporumSchlechtendalf.sp.momordicae_Balsampear	156	--	156
39.MAFF238905_F.oxysporumSchlechtendalf.sp.radicis-lycopersici_Tomato	156	--	156
40.MAFF150004_F.oxysporumSchlechtendalf.sp.spinaciae_Spinach	156	--	156
41.MAFF247034_F.oxysporumSchlechtendal__Goldenchain	156	--	156
42.MAFF245747_F.oxysporumSchlechtendalf.sp.callistephi__Chinaaster	156	--	156
43.MAFF305115_FoxysporumSchlechtendalf.sp.batatas__Sweatpotato	161	--	161
44.MAFF150126_F.asiaticum_Seed	135	--	135
45.MAFF246738_F.solani_Angelica	161	--	161
46.MAFF246664_F.cugenangense_Perilla	156	--	156
47.MH582420.1F.solanistrainMRC256	150	--	150

```
48.MAFF240361_F.babinda_Soil                                        158 ---------------------------------------- 158
49.MAFF242368_F.azukicola_Azukibean                                 162 ---------------------------------------- 162
50.MAFF241312_F.asiaticum_Soil,welshonionfield                      135 ---------------------------------------- 135
51.LT548416.1_F.culmorumpartialtef1a                                131 ---------------------------------------- 131
52.MAFF150124_F.asiaticum__Wheat                                    135 ---------------------------------------- 135
53.MAFF238806_F.begoniae_Oncidiumsp                                 172 ---------------------------------------- 172
54.MW594399.1_FusariumincarnatumisolateUD01C                        163 ---------------------------------------- 163
55.OP414923.1Pucciniagraminisf.sp.triticiisolateSHZPgt19             42 TTTATCTTCTGCTTCCTATCTGGGACCTCCTTGTGTTCGT 81
56.MT027094.1_BipolarisoryzaestrainOrL-2                              0 ---------------------------------------- 0
57.ON734360.1_AlternariaalternataisolateH126                          0 ---------------------------------------- 0
58.LC333578.1_StemphyliumlycopersiciSOasp2                            0 ---------------------------------------- 0
59.HQ718583.1_Colletotrichumgloeosporioidesisolateq-1               257 CTTTCAAGTAC--GCCTGGGTGGGTCGCCGTCTCTTCGCG 294
60.JN241603.1_AtheliarolfsiiisolateSR1                                0 ---------------------------------------- 0
61.KJ866474.1_RhizoctoniasolanistrainMHL-1                            0 ---------------------------------------- 0
62.JQ672424.1AlternariatriticinaisolateEGS17-061                      0 ---------------------------------------- 0
63.LT707559.1_P.capsicipartialtef1agene                               0 ---------------------------------------- 0
64.MW090051.1_CurvularialunatastrainCls-3                             0 ---------------------------------------- 0
65.DQ400892.1_Aspergillusterreus                                      0 ---------------------------------------- 0
66.DQ911416.1_Pythiumsp.quercumstrainPy292                            0 ---------------------------------------- 0
67.EU797495.1_Phytophthorasp.oaksoilPoland                            0 ---------------------------------------- 0
68.HM148321.1_Cladosporiumcucumerinum                                 0 ---------------------------------------- 0
69.AF398888.1_SclerotiniasclerotiorumisolateSS1                       0 ---------------------------------------- 0
70.AF398888.1_S.sclerotiorumisolateSS1                                0 ---------------------------------------- 0
71.HPAB545908.1_Verticilliumnonalfalfaeisolate                        0 ---------------------------------------- 0
72.EF433315.1_CeratocystisfimbriatavoucherCMW15052                  206 AATTCATGTACTGACCTTCTTTGCACAGGAGGCCGCTGAG 245
73.MN159912.1_Botrytiscinerea                                         0 ---------------------------------------- 0
74.MF034741.1_PeltasterfructicolaisolateSRB92                         0 ---------------------------------------- 0
75.LC440360.1_CercosporaasparagiCOasp2                                0 ---------------------------------------- 0
76.AY944105.1_MagnaportheoryzaeisolateSAG00T3()                       0 ---------------------------------------- 0
77.JX266586.1_CochliobolusmiyabeanusvoucherMFLUCC10-0733              0 ---------------------------------------- 0
78.MN393253.1_CorynesporacassiicolaisolateQHD001(MN393253.1UNVERIFIED) 0 ---------------------------------------- 0
79.MF375218.1_AgroatheliarolfsiiisolateBJB24                          0 ---------------------------------------- 0
80.MN106270.1_AgroatheliarolfsiistrainJ-12                            0 ---------------------------------------- 0
81.OQ732628.1_AgroatheliarolfsiiisolateBTCBSr3                        0 ---------------------------------------- 0
82.KY196185.1_ColletotrichumtruncatumstrainPAK53                    214 CCTTCAAGTAC--GCCTG---------------------- 229
83.GU935835.1_ColletotrichumcoccodesisolateC96002                     0 ---------------------------------------- 0
84.MK085963.1_AlternariatenuissimaisolateSCCZ06                       0 ---------------------------------------- 0
85.MT548042.1_AlternarialongipesstrainKY_2019_012                     0 ---------------------------------------- 0
86.MN356465.1CalonectriamontanaisolateHSP4                            0 ---------------------------------------- 0
87.OL694224.1_CalonectriacanadianastrainF099                          0 ---------------------------------------- 0
88.MK803351.1_NeoscytalidiumdimidiatumstrainKale4-C                   0 ---------------------------------------- 0
89.ON376993.1_CurvulariachiangmaiensisisolateND00J7                   0 ---------------------------------------- 0
90.OQ383346.1_NeoscytalidiumdimidiatumisolateGKH-2                    0 ---------------------------------------- 0
91.MF662595.1_NeoscytalidiumnovaehollandiaeisolateNeNo1               0 ---------------------------------------- 0
92.EF560588.1Melampsoralini                                          56 TCAAGTAAGTCATGCTCGTTTACCGCCCTTCACTCCTCTG 95
93.LC590862.1_NeoscytalidiumdimidiatumPSU-HP01TEF1                    0 ---------------------------------------- 0
```

```
94.KX278106.1_BotryosphaeriaqingyuanensisstrainCERC2947                   0 ---------------------------------------- 0
95.AJ578763.1_Blumeriagraminisf.sp.hordeicyp51                            0 ---------------------------------------- 0
96.MF490858.1_CurvulariadactylocteniicolastrainCPC28810                   0 ---------------------------------------- 0
97.KT287115.1_Bipolariscactivoraisolate3.8.6                              0 ---------------------------------------- 0
98.MT560940.1_CurvulariacactivorastrainHLGH0118                           0 ---------------------------------------- 0
99.OM714565.1_CurvulariaplantarumstrainM0134                              0 ---------------------------------------- 0
100.MN159911.1_BotrytiscinereaSICAUCC19-0003                              0 ---------------------------------------- 0
102.GU294713.1LasiodiplodiatheobromaestrainUCD2430TX                      0 ---------------------------------------- 0
103.KX868094.1_Mycosphaerellasp.isolateCRM20.1                            0 ---------------------------------------- 0
104.LC599478.1Pseudocercosporapini-densifloraeMUCC534                     0 ---------------------------------------- 0
105.N584698.1BipolarissetariaestrainKBS4-2                                0 ---------------------------------------- 0

2.OM160859.1_F.buharicum                                                128 ---------------------------------------- 128
1.LC727524.1_F.buharicum_OKI-1_Okura                                    159 ---------------------------------------- 159
3.KX302919.1_F.sublunatum                                               125 ---------------------------------------- 125
4.LT996094.1_F.convolutans                                              125 ---------------------------------------- 125
5.OM160861.1_F.abutilonis                                               127 ---------------------------------------- 127
6.OM160874.1_F.guadeloupense                                            126 ---------------------------------------- 126
7.MH392475.1_F.graminearum                                               80 ---------------------------------------- 80
8.MH582420.1_F.solani                                                   130 ---------------------------------------- 130
9.MAFF244605_F.oxysporum                                                126 ---------------------------------------- 126
10.MAFF237278_F.contaminatum_Hylocereus                                 173 ---------------------------------------- 173
11.MAFF237649_F.concentricum__Riceroot                                  166 ---------------------------------------- 166
12.MAFF237650_F.concentricum__Wheat                                     166 ---------------------------------------- 166
13.MAFF239869_F.mangiferae__Ryukyupine                                  156 ---------------------------------------- 156
14.MAFF240460_F.fujikuroi_Passionfruit                                  156 ---------------------------------------- 156
15.MAFF241317_F.graminearum_Wheat                                       135 ---------------------------------------- 135
16.MAFF242670_F.ipomoeae_Wheat                                          158 ---------------------------------------- 158
17.MAFF245129_F.concentricum_Fraxinus                                   156 ---------------------------------------- 156
18.MAFF245395_F.cugenangense_Rhubarb                                    156 ---------------------------------------- 156
19.MAFF246637_F.nirenbergiae_Strawberry                                 156 ---------------------------------------- 156
20.MAFF246672_F.nirenbergiae_ChinesePeony                               156 ---------------------------------------- 156
21.MAFF246697_F.commune_Urallicoriceroot                                157 ---------------------------------------- 157
22.MAFF246729_F.falciforme_Angelica                                     161 ---------------------------------------- 161
23.MAFF247220_F.duplospermum__Euwallaceasp                              161 ---------------------------------------- 161
24.MAFF410760_F.odoratissimum_alpha                                     156 ---------------------------------------- 156
25.MAFF244605_FusariumoxysporumSchlechtendal_MAFF244605_Tomato          157 ---------------------------------------- 157
26.MAFF241326_F.asiaticum_Wheat                                         138 ---------------------------------------- 138
27.MAFF245014_F.asiaticum_Wildsoybean                                   135 ---------------------------------------- 135
28.MAFF150124_F.asiaticum__Wheat                                        135 ---------------------------------------- 135
29.OM135603.1F.algeriense                                               163 ---------------------------------------- 163
30.MAFF237465_F.penzigii_Aloe                                           166 ---------------------------------------- 166
31.MAFF103054_F.oxysporumSchlechtendalf.sp.cucumerinum_Cucumber         156 ---------------------------------------- 156
32.MAFF712246_F.oxysporumSchlechtendalf.sp.dianthi__Carnation           156 ---------------------------------------- 156
33.MAFF305558_F.oxysporumSchlechtendalf.sp.fragariae__Watermelon        156 ---------------------------------------- 156
34.MAFF744087_F.oxysporumSchlechtendalf.sp.lactucae__Lettuce            156 ---------------------------------------- 156
```

```
35.MAFF726924_F.oxysporumSchlechtendalf.sp.lagenariae_Whitefloweredgourd 156 ---------------------------------------- 156
36.MAFF744003_F.oxysporumSchlechtendalf.sp.lagenariae_Squash) 156 ---------------------------------------- 156
37.MAFF305122_F.oxysporumSchlechtendalf.sp.melonis__Melon 156 ---------------------------------------- 156
38.MAFF306714_F.oxysporumSchlechtendalf.sp.momordicae_Balsampear 156 ---------------------------------------- 156
39.MAFF238905_F.oxysporumSchlechtendalf.sp.radicis-lycopersici_Tomato 156 ---------------------------------------- 156
40.MAFF150004_F.oxysporumSchlechtendalf.sp.spinaciae_Spinach 156 ---------------------------------------- 156
41.MAFF247034_F.oxysporumSchlechtendal__Goldenchain 156 ---------------------------------------- 156
42.MAFF245747_F.oxysporumSchlechtendalf.sp.callistephi__Chinaaster 156 ---------------------------------------- 156
43.MAFF305115_FoxysporumSchlechtendalf.sp.batatas__Sweatpotato 161 ---------------------------------------- 161
44.MAFF150126_F.asiaticum_Seed 135 ---------------------------------------- 135
45.MAFF246738_F.solani_Angelica 161 ---------------------------------------- 161
46.MAFF246664_F.cugenangense_Perilla 156 ---------------------------------------- 156
47.MH582420.1F.solanistrainMRC256 150 ---------------------------------------- 150
48.MAFF240361_F.babinda_Soil 158 ---------------------------------------- 158
49.MAFF242368_F.azukicola_Azukibean 162 ---------------------------------------- 162
50.MAFF241312_F.asiaticum_Soil,welshonionfield 135 ---------------------------------------- 135
51.LT548416.1_F.culmorumpartialtef1a 131 ---------------------------------------- 131
52.MAFF150124_F.asiaticum__Wheat 135 ---------------------------------------- 135
53.MAFF238806_F.begoniae_Oncidiumsp 172 ---------------------------------------- 172
54.MW594399.1_FusariumincarnatumisolateUD01C 163 ---------------------------------------- 163
55.OP414923.1Pucciniagraminisf.sp.triticiisolateSHZPgt19 82 CTACTGAGATAGTCTATCATAGGTACGCAGGGGTGCTTGA 121
56.MT027094.1_BipolarisoryzaestrainOrL-2 0 ---------------------------------------- 0
57.ON734360.1_AlternariaalternataisolateH126 0 ---------------------------------------- 0
58.LC333578.1_StemphyliumlycopersiciSOasp2 0 ---------------------------------------- 0
59.HQ718583.1_Colletotrichumgloeosporioidesisolateq-1 295 ATCCTGAAATTGTTTTACTGAT-TTTTCGCAGGTTCTTGA 333
60.JN241603.1_AtheliarolfsiiisolateSR1 0 ---------------------------------------- 0
61.KJ866474.1_RhizoctoniasolanistrainMHL-1 0 ---------------------------------------- 0
62.JQ672424.1AlternariatriticinaisolateEGS17-061 0 ---------------------------------------- 0
63.LT707559.1_P.capsicipartialtef1agene 0 ---------------------------------------- 0
64.MW090051.1_CurvularialunatastrainCls-3 0 ---------------------------------------- 0
65.DQ400892.1_Aspergillusterreus 0 ---------------------------------------- 0
66.DQ911416.1_Pythiumsp.quercumstrainPy292 0 ---------------------------------------- 0
67.EU797495.1_Phytophthorasp.oaksoilPoland 0 ---------------------------------------- 0
68.HM148321.1_Cladosporiumcucumerinum 0 ---------------------------------------- 0
69.AF398888.1_SclerotiniasclerotiorumisolateSS1 0 ---------------------------------------- 0
70.AF398888.1_S.sclerotiorumisolateSS1 0 ---------------------------------------- 0
71.HPAB545908.1_Verticilliumnonalfalfaeisolate 0 ---------------------------------------- 0
72.EF433315.1_CeratocystisfimbriatavoucherCMW15052 246 CTCG-GTAAGGGTTCCTTCAAG-TACGCCTGGGTTCTTGA 283
73.MN159912.1_Botrytiscinerea 0 ---------------------------------------- 0
74.MF034741.1_PeltasterfructicolaisolateSRB92 0 ---------------------------------------- 0
75.LC440360.1_CercosporaasparagiCOasp2 0 ---------------------------------------- 0
76.AY944105.1_MagnaportheoryzaeisolateSAG00T3() 0 ---------------------------------------- 0
77.JX266586.1_CochliobolusmiyabeanusvoucherMFLUCC10-0733 0 ---------------------------------------- 0
78.MN393253.1_CorynesporacassiicolaisolateQHD001(MN393253.1UNVERIFIED) 0 ---------------------------------------- 0
79.MF375218.1_AgroatheliarolfsiiisolateBJB24 0 ---------------------------------------- 0
80.MN106270.1_AgroatheliarolfsiistrainJ-12 0 ---------------------------------------- 0
```

81.OQ732628.1_AgroatheliarolfsiiisolateBTCBSr3	0	--	0
82.KY196185.1_ColletotrichumtruncatumstrainPAK53	230	-------------------------------GGTTCTTGA	238
83.GU935835.1_ColletotrichumcoccodesisolateC96002	0	--	0
84.MK085963.1_AlternariatenuissimaisolateSCCZ06	0	--	0
85.MT548042.1_AlternarialongipesstrainKY_2019_012	0	--	0
86.MN356465.1CalonectriamontanaisolateHSP4	0	--	0
87.OL694224.1_CalonectriacanadianastrainF099	0	--	0
88.MK803351.1_NeoscytalidiumdimidiatumstrainKale4-C	0	--	0
89.ON376993.1_CurvulariachiangmaiensisisolateND00J7	0	--	0
90.OQ383346.1_NeoscytalidiumdimidiatumisolateGKH-2	0	--	0
91.MF662595.1_NeoscytalidiumnovaehollandiaeisolateNeNo1	0	--	0
92.EF560588.1Melampsoralini	96	GCTCATGTTTGGTCACCTCCAGATATGCCTGGGTACTCGA	135
93.LC590862.1_NeoscytalidiumdimidiatumPSU-HP01TEF1	0	--	0
94.KX278106.1_BotryosphaeriaqingyuanensisstrainCERC2947	0	--	0
95.AJ578763.1_Blumeriagraminisf.sp.hordeicyp51	0	--	0
96.MF490858.1_CurvulariadactyloctenicolastrainCPC28810	0	--	0
97.KT287115.1_Bipolariscactivoraisolate3.8.6	0	--	0
98.MT560940.1_CurvulariacactivorastrainHLGH0118	0	--	0
99.OM714565.1_CurvulariaplantarumstrainM0134	0	--	0
100.MN159911.1_BotrytiscinereaSICAUCC19-0003	0	--	0
102.GU294713.1LasiodiplodiatheobromaestrainUCD2430TX	0	--	0
103.KX868094.1_Mycosphaerellasp.isolateCRM20.1	0	--	0
104.LC599478.1Pseudocercosporapini-densifloraeMUCC534	0	--	0
105.N584698.1BipolarissetariaestrainKBS4-2	0	--	0
2.OM160859.1_F.buharicum	128	--	128
1.LC727524.1_F.buharicum_OKI-1_Okura	159	--	159
3.KX302919.1_F.sublunatum	125	--	125
4.LT996094.1_F.convolutans	125	--	125
5.OM160861.1_F.abutilonis	127	--	127
6.OM160874.1_F.guadeloupense	126	--	126
7.MH392475.1_F.graminearum	80	--	80
8.MH582420.1_F.solani	130	--	130
9.MAFF244605_F.oxysporum	126	--	126
10.MAFF237278_F.contaminatum_Hylocereus	173	--	173
11.MAFF237649_F.concentricum__Riceroot	166	--	166
12.MAFF237650_F.concentricum__Wheat	166	--	166
13.MAFF239869_F.mangiferae__Ryukyupine	156	--	156
14.MAFF240460_F.fujikuroi_Passionfruit	156	--	156
15.MAFF241317_F.graminearum_Wheat	135	--	135
16.MAFF242670_F.ipomoeae_Wheat	158	--	158
17.MAFF245129_F.concentricum_Fraxinus	156	--	156
18.MAFF245395_F.cugenangense_Rhubarb	156	--	156
19.MAFF246637_F.nirenbergiae_Strawberry	156	--	156
20.MAFF246672_F.nirenbergiae_ChinesePeony	156	--	156
21.MAFF246697_F.commune_Urallicoriceroot	157	--	157

22.MAFF246729_F.falciforme_Angelica	161	--	161
23.MAFF247220_F.duplospermum__Euwallaceasp	161	--	161
24.MAFF410760_F.odoratissimum_alpha	156	--	156
25.MAFF244605_FusariumoxysporumSchlechtendal_MAFF244605_Tomato	157	--	157
26.MAFF241326_F.asiaticum_Wheat	138	--	138
27.MAFF245014_F.asiaticum_Wildsoybean	135	--	135
28.MAFF150124_F.asiaticum__Wheat	135	--	135
29.OM135603.1F.algeriense	163	--	163
30.MAFF237465_F.penzigii_Aloe	166	--	166
31.MAFF103054_F.oxysporumSchlechtendalf.sp.cucumerinum_Cucumber	156	--	156
32.MAFF712246_F.oxysporumSchlechtendalf.sp.dianthi__Carnation	156	--	156
33.MAFF305558_F.oxysporumSchlechtendalf.sp.fragariae__Watermelon	156	--	156
34.MAFF744087_F.oxysporumSchlechtendalf.sp.lactucae__Lettuce	156	--	156
35.MAFF726924_F.oxysporumSchlechtendalf.sp.lagenariae_Whitefloweredgourd	156	--	156
36.MAFF744003_F.oxysporumSchlechtendalf.sp.lagenariae_Squash)	156	--	156
37.MAFF305122_F.oxysporumSchlechtendalf.sp.melonis__Melon	156	--	156
38.MAFF306714_F.oxysporumSchlechtendalf.sp.momordicae_Balsampear	156	--	156
39.MAFF238905_F.oxysporumSchlechtendalf.sp.radicis-lycopersici_Tomato	156	--	156
40.MAFF150004_F.oxysporumSchlechtendalf.sp.spinaciae_Spinach	156	--	156
41.MAFF247034_F.oxysporumSchlechtendal__Goldenchain	156	--	156
42.MAFF245747_F.oxysporumSchlechtendalf.sp.callistephi__Chinaaster	156	--	156
43.MAFF305115_FoxysporumSchlechtendalf.sp.batatas__Sweatpotato	161	--	161
44.MAFF150126_F.asiaticum_Seed	135	--	135
45.MAFF246738_F.solani_Angelica	161	--	161
46.MAFF246664_F.cugenangense_Perilla	156	--	156
47.MH582420.1F.solanistrainMRC256	150	--	150
48.MAFF240361_F.babinda_Soil	158	--	158
49.MAFF242368_F.azukicola_Azukibean	162	--	162
50.MAFF241312_F.asiaticum_Soil,welshonionfield	135	--	135
51.LT548416.1_F.culmorumpartialtef1a	131	--	131
52.MAFF150124_F.asiaticum__Wheat	135	--	135
53.MAFF238806_F.begoniae_Oncidiumsp	172	--	172
54.MW594399.1_FusariumincarnatumisolateUD01C	163	--	163
55.OP414923.1Pucciniagraminisf.sp.triticiisolateSHZPgt19	122	CAAGCTGAAAGCCGAGCGTGAGCGTGGTATCACCATCGAC	161
56.MT027094.1_BipolarisoryzaestrainOrL-2	0	--	0
57.ON734360.1_AlternariaalternataisolateH126	0	--	0
58.LC333578.1_StemphyliumlycopersiciSOasp2	0	--	0
59.HQ718583.1_Colletotrichumgloeosporioidesisolateq-1	334	CAAGCTCAAGGCCGAGCGTGAGCGTGGTATCACCATCGAC	373
60.JN241603.1_AtheliarolfsiiisolateSR1	0	--	0
61.KJ866474.1_RhizoctoniasolanistrainMHL-1	0	--	0
62.JQ672424.1AlternariatriticinaisolateEGS17-061	0	--	0
63.LT707559.1_P.capsicipartialtef1agene	0	--	0
64.MW090051.1_CurvularialunatastrainCls-3	0	--	0
65.DQ400892.1_Aspergillusterreus	0	--	0
66.DQ911416.1_Pythiumsp.quercumstrainPy292	0	--	0
67.EU797495.1_Phytophthorasp.oaksoilPoland	0	--	0

68.HM148321.1_Cladosporiumcucumerinum	0	--	0
69.AF398888.1_SclerotiniasclerotiorumisolateSS1	0	--	0
70.AF398888.1_S.sclerotiorumisolateSS1	0	--	0
71.HPAB545908.1_Verticilliumnonalfalfaeisolate	0	--	0
72.EF433315.1_CeratocystisfimbriatavoucherCMW15052	284	CAAGCTCAAGGCCGAGCGTGAGCGTGGTATCACTATCGAC	323
73.MN159912.1_Botrytiscinerea	0	--	0
74.MF034741.1_PeltasterfructicolaisolateSRB92	0	--	0
75.LC440360.1_CercosporaasparagiCOasp2	0	--	0
76.AY944105.1_MagnaportheoryzaeisolateSAG00T3()	0	--	0
77.JX266586.1_CochliobolusmiyabeanusvoucherMFLUCC10-0733	0	--	0
78.MN393253.1_CorynesporacassiicolaisolateQHD001(MN393253.1UNVERIFIED)	0	--	0
79.MF375218.1_AgroatheliarolfsiiisolateBJB24	0	--	0
80.MN106270.1_AgroatheliarolfsiistrainJ-12	0	--	0
81.OQ732628.1_AgroatheliarolfsiiisolateBTCBSr3	0	--	0
82.KY196185.1_ColletotrichumtruncatumstrainPAK53	239	CAAGCTCAAGGCCGAGCGTGAGCGTGGTATCACCATCGAC	278
83.GU935835.1_ColletotrichumcoccodesisolateC96002	0	--	0
84.MK085963.1_AlternariatenuissimaisolateSCCZ06	0	--	0
85.MT548042.1_AlternarialongipesstrainKY_2019_012	0	--	0
86.MN356465.1CalonectriamontanaisolateHSP4	0	--	0
87.OL694224.1_CalonectriacanadianastrainF099	0	--	0
88.MK803351.1_NeoscytalidiumdimidiatumstrainKale4-C	0	--	0
89.ON376993.1_CurvulariachiangmaiensisisolateND00J7	0	--	0
90.OQ383346.1_NeoscytalidiumdimidiatumisolateGKH-2	0	--	0
91.MF662595.1_NeoscytalidiumnovaehollandiaeisolateNeNo1	0	--	0
92.EF560588.1Melampsoralini	136	CAAGCTCAAGGCCGAGCGAGAACGTGGTATCACCATCGAT	175
93.LC590862.1_NeoscytalidiumdimidiatumPSU-HP01TEF1	0	--	0
94.KX278106.1_BotryosphaeriaqingyuanensisstrainCERC2947	0	--	0
95.AJ578763.1_Blumeriagraminisf.sp.hordeicyp51	0	--	0
96.MF490858.1_CurvulariadactylocteniicolastrainCPC28810	0	--	0
97.KT287115.1_Bipolariscactivoraisolate3.8.6	0	--	0
98.MT560940.1_CurvulariacactivorastrainHLGH0118	0	--	0
99.OM714565.1_CurvulariaplantarumstrainM0134	0	--	0
100.MN159911.1_BotrytiscinereaSICAUCC19-0003	0	--	0
102.GU294713.1LasiodiplodiatheobromaestrainUCD2430TX	0	--	0
103.KX868094.1_Mycosphaerellasp.isolateCRM20.1	0	--	0
104.LC599478.1Pseudocercosporapini-densifloraeMUCC534	0	--	0
105.N584698.1BipolarissetariaestrainKBS4-2	0	--	0

2.OM160859.1_F.buharicum	128	--	128
1.LC727524.1_F.buharicum_OKI-1_Okura	159	--	159
3.KX302919.1_F.sublunatum	125	--	125
4.LT996094.1_F.convolutans	125	--	125
5.OM160861.1_F.abutilonis	127	--	127
6.OM160874.1_F.guadeloupense	126	--	126
7.MH392475.1_F.graminearum	80	--	80
8.MH582420.1_F.solani	130	--	130

9.MAFF244605_F.oxysporum	126	--	126
10.MAFF237278_F.contaminatum_Hylocereus	173	--	173
11.MAFF237649_F.concentricum__Riceroot	166	--	166
12.MAFF237650_F.concentricum__Wheat	166	--	166
13.MAFF239869_F.mangiferae__Ryukyupine	156	--	156
14.MAFF240460_F.fujikuroi_Passionfruit	156	--	156
15.MAFF241317_F.graminearum_Wheat	135	--	135
16.MAFF242670_F.ipomoeae_Wheat	158	--	158
17.MAFF245129_F.concentricum_Fraxinus	156	--	156
18.MAFF245395_F.cugenangense_Rhubarb	156	--	156
19.MAFF246637_F.nirenbergiae_Strawberry	156	--	156
20.MAFF246672_F.nirenbergiae_ChinesePeony	156	--	156
21.MAFF246697_F.commune_Urallicoriceroot	157	--	157
22.MAFF246729_F.falciforme_Angelica	161	--	161
23.MAFF247220_F.duplospermum__Euwallaceasp	161	--	161
24.MAFF410760_F.odoratissimum_alpha	156	--	156
25.MAFF244605_FusariumoxysporumSchlechtendal_MAFF244605_Tomato	157	--	157
26.MAFF241326_F.asiaticum_Wheat	138	--	138
27.MAFF245014_F.asiaticum_Wildsoybean	135	--	135
28.MAFF150124_F.asiaticum__Wheat	135	--	135
29.OM135603.1F.algeriense	163	--	163
30.MAFF237465_F.penzigii_Aloe	166	--	166
31.MAFF103054_F.oxysporumSchlechtendalf.sp.cucumerinum_Cucumber	156	--	156
32.MAFF712246_F.oxysporumSchlechtendalf.sp.dianthi__Carnation	156	--	156
33.MAFF305558_F.oxysporumSchlechtendalf.sp.fragariae__Watermelon	156	--	156
34.MAFF744087_F.oxysporumSchlechtendalf.sp.lactucae__Lettuce	156	--	156
35.MAFF726924_F.oxysporumSchlechtendalf.sp.lagenariae_Whiteflowereedgourd	156	--	156
36.MAFF744003_F.oxysporumSchlechtendalf.sp.lagenariae_Squash)	156	--	156
37.MAFF305122_F.oxysporumSchlechtendalf.sp.melonis__Melon	156	--	156
38.MAFF306714_F.oxysporumSchlechtendalf.sp.momordicae_Balsampear	156	--	156
39.MAFF238905_F.oxysporumSchlechtendalf.sp.radicis-lycopersici_Tomato	156	--	156
40.MAFF150004_F.oxysporumSchlechtendalf.sp.spinaciae_Spinach	156	--	156
41.MAFF247034_F.oxysporumSchlechtendal__Goldenchain	156	--	156
42.MAFF245747_F.oxysporumSchlechtendalf.sp.callistephi__Chinaaster	156	--	156
43.MAFF305115_FoxysporumSchlechtendalf.sp.batatas__Sweatpotato	161	--	161
44.MAFF150126_F.asiaticum_Seed	135	--	135
45.MAFF246738_F.solani_Angelica	161	--	161
46.MAFF246664_F.cugenangense_Perilla	156	--	156
47.MH582420.1F.solanistrainMRC256	150	--	150
48.MAFF240361_F.babinda_Soil	158	--	158
49.MAFF242368_F.azukicola_Azukibean	162	--	162
50.MAFF241312_F.asiaticum_Soil,welshonionfield	135	--	135
51.LT548416.1_F.culmorumpartialtef1a	131	--	131
52.MAFF150124_F.asiaticum__Wheat	135	--	135
53.MAFF238806_F.begoniae_Oncidiumsp	172	--	172
54.MW594399.1_FusariumincarnatumisolateUD01C	163	--	163

```
55.OP414923.1Pucciniagraminisf.sp.triticiisolateSHZPgt19                 162 ATTGCCGT--GAGTCGATCCTGAGCTTGTGAGGACTCTTA 199
56.MT027094.1_BipolarisoryzaestrainOrL-2                                   0 ---------------------------------------- 0
57.ON734360.1_AlternariaalternataisolateH126                               0 ---------------------------------------- 0
58.LC333578.1_StemphyliumlycopersiciSOasp2                                 0 ---------------------------------------- 0
59.HQ718583.1_Colletotrichumgloeosporioidesisolateq-1                    374 ATTGCCCT--CTGGAAGTTCGAGACTCCCAGGTACTATGT 411
60.JN241603.1_AtheliarolfsiiisolateSR1                                     0 ---------------------------------------- 0
61.KJ866474.1_RhizoctoniasolanistrainMHL-1                                 0 ---------------------------------------- 0
62.JQ672424.1AlternariatriticinaisolateEGS17-061                           0 ---------------------------------------- 0
63.LT707559.1_P.capsicipartialtef1agene                                    0 ---------------------------------------- 0
64.MW090051.1_CurvularialunatastrainCls-3                                  0 ---------------------------------------- 0
65.DQ400892.1_Aspergillusterreus                                           0 ---------------------------------------- 0
66.DQ911416.1_Pythiumsp.quercumstrainPy292                                 0 ---------------------------------------- 0
67.EU797495.1_Phytophthorasp.oaksoilPoland                                 0 ---------------------------------------- 0
68.HM148321.1_Cladosporiumcucumerinum                                      0 ---------------------------------------- 0
69.AF398888.1_SclerotiniasclerotiorumisolateSS1                            0 ---------------------------------------- 0
70.AF398888.1_S.sclerotiorumisolateSS1                                     0 ---------------------------------------- 0
71.HPAB545908.1_Verticilliumnonalfalfaeisolate                             0 ---------------------------------------- 0
72.EF433315.1_CeratocystisfimbriatavoucherCMW15052                       324 ATTGCCCT--GTGGAAGTTCGAGACCCCCAAGTACTACGT 361
73.MN159912.1_Botrytiscinerea                                              0 ---------------------------------------- 0
74.MF034741.1_PeltasterfructicolaisolateSRB92                              0 ---------------------------------------- 0
75.LC440360.1_CercosporaasparagiCOasp2                                     0 ---------------------------------------- 0
76.AY944105.1_MagnaportheoryzaeisolateSAG00T3()                            0 ---------------------------------------- 0
77.JX266586.1_CochliobolusmiyabeanusvoucherMFLUCC10-0733                   0 ---------------------------------------- 0
78.MN393253.1_CorynesporacassiicolaisolateQHD001(MN393253.1UNVERIFIED)     0 ---------------------------------------- 0
79.MF375218.1_AgroatheliarolfsiiisolateBJB24                               0 ---------------------------------------- 0
80.MN106270.1_AgroatheliarolfsiistrainJ-12                                 0 ---------------------------------------- 0
81.OQ732628.1_AgroatheliarolfsiiisolateBTCBSr3                             0 ---------------------------------------- 0
82.KY196185.1_ColletotrichumtruncatumstrainPAK53                         279 ATTGCCCT--CTGGAAGTTCGAGACTCCCAAGTACTATGT 316
83.GU935835.1_ColletotrichumcoccodesisolateC96002                          0 ---------------------------------------- 0
84.MK085963.1_AlternariatenuissimaisolateSCCZ06                            0 ---------------------------------------- 0
85.MT548042.1_AlternarialongipesstrainKY_2019_012                          0 ---------------------------------------- 0
86.MN356465.1CalonectriamontanaisolateHSP4                                 0 ---------------------------------------- 0
87.OL694224.1_CalonectriacanadianastrainF099                               0 ---------------------------------------- 0
88.MK803351.1_NeoscytalidiumdimidiatumstrainKale4-C                        0 ---------------------------------------- 0
89.ON376993.1_CurvulariachiangmaiensisisolateND00J7                        0 ---------------------------------------- 0
90.OQ383346.1_NeoscytalidiumdimidiatumisolateGKH-2                         0 ---------------------------------------- 0
91.MF662595.1_NeoscytalidiumnovaehollandiaeisolateNeNo1                    0 ---------------------------------------- 0
92.EF560588.1Melampsoralini                                              176 ATCGCTGTACGTTTATTTTGTCAACAGATATCTACCGCCT 215
93.LC590862.1_NeoscytalidiumdimidiatumPSU-HP01TEF1                         0 ---------------------------------------- 0
94.KX278106.1_BotryosphaeriaqingyuanensisstrainCERC2947                    0 ---------------------------------------- 0
95.AJ578763.1_Blumeriagraminisf.sp.hordeicyp51                             0 ---------------------------------------- 0
96.MF490858.1_CurvulariadactylocteniicolastrainCPC28810                    0 ---------------------------------------- 0
97.KT287115.1_Bipolariscactivoraisolate3.8.6                               0 ---------------------------------------- 0
98.MT560940.1_CurvulariacactivorastrainHLGH0118                            0 ---------------------------------------- 0
99.OM714565.1_CurvulariaplantarumstrainM0134                               0 ---------------------------------------- 0
100.MN159911.1_BotrytiscinereaSICAUCC19-0003                               0 ---------------------------------------- 0
```

102.GU294713.1LasiodiplodiatheobromaestrainUCD2430TX	0	--	0
103.KX868094.1_Mycosphaerellasp.isolateCRM20.1	0	--	0
104.LC599478.1Pseudocercosporapini-densifloraeMUCC534	0	--	0
105.N584698.1BipolarissetariaestrainKBS4-2	0	--	0
2.OM160859.1_F.buharicum	128	--	128
1.LC727524.1_F.buharicum_OKI-1_Okura	159	--	159
3.KX302919.1_F.sublunatum	125	--	125
4.LT996094.1_F.convolutans	125	--	125
5.OM160861.1_F.abutilonis	127	--	127
6.OM160874.1_F.guadeloupense	126	--	126
7.MH392475.1_F.graminearum	80	--	80
8.MH582420.1_F.solani	130	--	130
9.MAFF244605_F.oxysporum	126	--	126
10.MAFF237278_F.contaminatum_Hylocereus	173	--	173
11.MAFF237649_F.concentricum__Riceroot	166	--	166
12.MAFF237650_F.concentricum__Wheat	166	--	166
13.MAFF239869_F.mangiferae__Ryukyupine	156	--	156
14.MAFF240460_F.fujikuroi_Passionfruit	156	--	156
15.MAFF241317_F.graminearum_Wheat	135	--	135
16.MAFF242670_F.ipomoeae_Wheat	158	--	158
17.MAFF245129_F.concentricum_Fraxinus	156	--	156
18.MAFF245395_F.cugenangense_Rhubarb	156	--	156
19.MAFF246637_F.nirenbergiae_Strawberry	156	--	156
20.MAFF246672_F.nirenbergiae_ChinesePeony	156	--	156
21.MAFF246697_F.commune_Urallicoriceroot	157	--	157
22.MAFF246729_F.falciforme_Angelica	161	--	161
23.MAFF247220_F.duplospermum__Euwallaceasp	161	--	161
24.MAFF410760_F.odoratissimum_alpha	156	--	156
25.MAFF244605_FusariumoxysporumSchlechtendal_MAFF244605_Tomato	157	--	157
26.MAFF241326_F.asiaticum_Wheat	138	--	138
27.MAFF245014_F.asiaticum_Wildsoybean	135	--	135
28.MAFF150124_F.asiaticum__Wheat	135	--	135
29.OM135603.1F.algeriense	163	--	163
30.MAFF237465_F.penzigii_Aloe	166	--	166
31.MAFF103054_F.oxysporumSchlechtendalf.sp.cucumerinum_Cucumber	156	--	156
32.MAFF712246_F.oxysporumSchlechtendalf.sp.dianthi__Carnation	156	--	156
33.MAFF305558_F.oxysporumSchlechtendalf.sp.fragariae__Watermelon	156	--	156
34.MAFF744087_F.oxysporumSchlechtendalf.sp.lactucae__Lettuce	156	--	156
35.MAFF726924_F.oxysporumSchlechtendalf.sp.lagenariae_Whiteflowereddgourd	156	--	156
36.MAFF744003_F.oxysporumSchlechtendalf.sp.lagenariae_Squash)	156	--	156
37.MAFF305122_F.oxysporumSchlechtendalf.sp.melonis__Melon	156	--	156
38.MAFF306714_F.oxysporumSchlechtendalf.sp.momordicae_Balsampear	156	--	156
39.MAFF238905_F.oxysporumSchlechtendalf.sp.radicis-lycopersici_Tomato	156	--	156
40.MAFF150004_F.oxysporumSchlechtendalf.sp.spinaciae_Spinach	156	--	156
41.MAFF247034_F.oxysporumSchlechtendal__Goldenchain	156	--	156

42.MAFF245747_F.oxysporumSchlechtendalf.sp.callistephi__Chinaaster	156	--	156
43.MAFF305115_FoxysporumSchlechtendalf.sp.batatas__Sweatpotato	161	--	161
44.MAFF150126_F.asiaticum_Seed	135	--	135
45.MAFF246738_F.solani_Angelica	161	--	161
46.MAFF246664_F.cugenangense_Perilla	156	--	156
47.MH582420.1F.solanistrainMRC256	150	--	150
48.MAFF240361_F.babinda_Soil	158	--	158
49.MAFF242368_F.azukicola_Azukibean	162	--	162
50.MAFF241312_F.asiaticum_Soil,welshonionfield	135	--	135
51.LT548416.1_F.culmorumpartialtef1a	131	--	131
52.MAFF150124_F.asiaticum__Wheat	135	--	135
53.MAFF238806_F.begoniae_Oncidiumsp	172	--	172
54.MW594399.1_FusariumincarnatumisolateUD01C	163	--	163
55.OP414923.1Pucciniagraminisf.sp.triticiisolateSHZPgt19	200	TGCGGCCTGCTGACCATTCCCACTA-CAGTTGTGG-AAGT	237
56.MT027094.1_BipolarisoryzaestrainOrL-2	0	--	0
57.ON734360.1_AlternariaalternataisolateH126	0	--	0
58.LC333578.1_StemphyliumlycopersiciSOasp2	0	--	0
59.HQ718583.1_Colletotrichumgloeosporioidesisolateq-1	412	CACCGTCATTGGTAAGTCCGGCTTT-CTTGCGCTG-AAAA	449
60.JN241603.1_AtheliarolfsiiisolateSR1	0	--	0
61.KJ866474.1_RhizoctoniasolanistrainMHL-1	0	--	0
62.JQ672424.1AlternariatriticinaisolateEGS17-061	0	--	0
63.LT707559.1_P.capsicipartialtef1agene	0	--	0
64.MW090051.1_CurvularialunatastrainCls-3	0	--	0
65.DQ400892.1_Aspergillusterreus	0	--	0
66.DQ911416.1_Pythiumsp.quercumstrainPy292	0	--	0
67.EU797495.1_Phytophthorasp.oaksoilPoland	0	--	0
68.HM148321.1_Cladosporiumcucumerinum	0	--	0
69.AF398888.1_SclerotiniasclerotiorumisolateSS1	0	--	0
70.AF398888.1_S.sclerotiorumisolateSS1	0	--	0
71.HPAB545908.1_Verticilliumnonalfalfaeisolate	0	--	0
72.EF433315.1_CeratocystisfimbriatavoucherCMW15052	362	CACTGTCATTGGTAAGCCTTCTTAT-CTCTTATTA-TTTT	399
73.MN159912.1_Botrytiscinerea	0	--	0
74.MF034741.1_PeltasterfructicolaisolateSRB92	0	--	0
75.LC440360.1_CercosporaasparagiCOasp2	0	--	0
76.AY944105.1_MagnaportheoryzaeisolateSAG00T3()	0	--	0
77.JX266586.1_CochliobolusmiyabeanusvoucherMFLUCC10-0733	0	--	0
78.MN393253.1_CorynesporacassiicolaisolateQHD001(MN393253.1UNVERIFIED)	0	--	0
79.MF375218.1_AgroatheliarolfsiiisolateBJB24	0	--	0
80.MN106270.1_AgroatheliarolfsiistrainJ-12	0	--	0
81.OQ732628.1_AgroatheliarolfsiiisolateBTCBSr3	0	--	0
82.KY196185.1_ColletotrichumtruncatumstrainPAK53	317	CACCGTCATTGGTAAGTTTAACCTC-ACCTATC-G-CACA	353
83.GU935835.1_ColletotrichumcoccodesisolateC96002	0	--	0
84.MK085963.1_AlternariatenuissimaisolateSCCZ06	0	--	0
85.MT548042.1_AlternarialongipesstrainKY_2019_012	0	--	0
86.MN356465.1CalonectriamontanaisolateHSP4	0	--	0
87.OL694224.1_CalonectriacanadianastrainF099	0	--	0

```
88.MK803351.1_NeoscytalidiumdimidiatumstrainKale4-C                0 ---------------------------------------- 0
89.ON376993.1_CurvulariachiangmaiensisisolateND00J7                 0 ---------------------------------------- 0
90.OQ383346.1_NeoscytalidiumdimidiatumisolateGKH-2                  0 ---------------------------------------- 0
91.MF662595.1_NeoscytalidiumnovaehollandiaeisolateNeNo1             0 ---------------------------------------- 0
92.EF560588.1Melampsoralini                                       216 GATCGATTGCTGACATACTGGTGTCGCTAGCTCTGGAAAT 255
93.LC590862.1_NeoscytalidiumdimidiatumPSU-HP01TEF1                  0 ---------------------------------------- 0
94.KX278106.1_BotryosphaeriaqingyuanensisstrainCERC2947             0 ---------------------------------------- 0
95.AJ578763.1_Blumeriagraminisf.sp.hordeicyp51                      0 ---------------------------------------- 0
96.MF490858.1_CurvulariadactylocteniicolastrainCPC28810             0 ---------------------------------------- 0
97.KT287115.1_Bipolariscactivoraisolate3.8.6                        0 ---------------------------------------- 0
98.MT560940.1_CurvulariacactivorastrainHLGH0118                     0 ---------------------------------------- 0
99.OM714565.1_CurvulariaplantarumstrainM0134                        0 ---------------------------------------- 0
100.MN159911.1_BotrytiscinereaSICAUCC19-0003                        0 ---------------------------------------- 0
102.GU294713.1LasiodiplodiatheobromaestrainUCD2430TX                0 ---------------------------------------- 0
103.KX868094.1_Mycosphaerellasp.isolateCRM20.1                      0 ---------------------------------------- 0
104.LC599478.1Pseudocercosporapini-densifloraeMUCC534               0 ---------------------------------------- 0
105.N584698.1BipolarissetariaestrainKBS4-2                          0 ---------------------------------------- 0

2.OM160859.1_F.buharicum                                          128 ---------------------------------------- 128
1.LC727524.1_F.buharicum_OKI-1_Okura                              159 ---------------------------------------- 159
3.KX302919.1_F.sublunatum                                         125 ---------------------------------------- 125
4.LT996094.1_F.convolutans                                        125 ---------------------------------------- 125
5.OM160861.1_F.abutilonis                                         127 ---------------------------------------- 127
6.OM160874.1_F.guadeloupense                                      126 ---------------------------------------- 126
7.MH392475.1_F.graminearum                                         80 ---------------------------------------- 80
8.MH582420.1_F.solani                                             130 ---------------------------------------- 130
9.MAFF244605_F.oxysporum                                          126 ---------------------------------------- 126
10.MAFF237278_F.contaminatum_Hylocereus                           173 ---------------------------------------- 173
11.MAFF237649_F.concentricum__Riceroot                            166 ---------------------------------------- 166
12.MAFF237650_F.concentricum__Wheat                               166 ---------------------------------------- 166
13.MAFF239869_F.mangiferae__Ryukyupine                            156 ---------------------------------------- 156
14.MAFF240460_F.fujikuroi_Passionfruit                            156 ---------------------------------------- 156
15.MAFF241317_F.graminearum_Wheat                                 135 ---------------------------------------- 135
16.MAFF242670_F.ipomoeae_Wheat                                    158 ---------------------------------------- 158
17.MAFF245129_F.concentricum_Fraxinus                             156 ---------------------------------------- 156
18.MAFF245395_F.cugenangense_Rhubarb                              156 ---------------------------------------- 156
19.MAFF246637_F.nirenbergiae_Strawberry                           156 ---------------------------------------- 156
20.MAFF246672_F.nirenbergiae_ChinesePeony                         156 ---------------------------------------- 156
21.MAFF246697_F.commune_Urallicoriceroot                          157 ---------------------------------------- 157
22.MAFF246729_F.falciforme_Angelica                               161 ---------------------------------------- 161
23.MAFF247220_F.duplospermum__Euwallaceasp                        161 ---------------------------------------- 161
24.MAFF410760_F.odoratissimum_alpha                               156 ---------------------------------------- 156
25.MAFF244605_FusariumoxysporumSchlechtendal_MAFF244605_Tomato    157 ---------------------------------------- 157
26.MAFF241326_F.asiaticum_Wheat                                   138 ---------------------------------------- 138
27.MAFF245014_F.asiaticum_Wildsoybean                             135 ---------------------------------------- 135
28.MAFF150124_F.asiaticum__Wheat                                  135 ---------------------------------------- 135
```

```
29.OM135603.1F.algeriense                                        163 ---------------------------------------- 163
30.MAFF237465_F.penzigii_Aloe                                    166 ---------------------------------------- 166
31.MAFF103054_F.oxysporumSchlechtendalf.sp.cucumerinum_Cucumber  156 ---------------------------------------- 156
32.MAFF712246_F.oxysporumSchlechtendalf.sp.dianthi__Carnation    156 ---------------------------------------- 156
33.MAFF305558_F.oxysporumSchlechtendalf.sp.fragariae__Watermelon 156 ---------------------------------------- 156
34.MAFF744087_F.oxysporumSchlechtendalf.sp.lactucae__Lettuce     156 ---------------------------------------- 156
35.MAFF726924_F.oxysporumSchlechtendalf.sp.lagenariae_Whitefloweredgourd 156 ---------------------------------------- 156
36.MAFF744003_F.oxysporumSchlechtendalf.sp.lagenariae_Squash)    156 ---------------------------------------- 156
37.MAFF305122_F.oxysporumSchlechtendalf.sp.melonis__Melon        156 ---------------------------------------- 156
38.MAFF306714_F.oxysporumSchlechtendalf.sp.momordicae_Balsampear 156 ---------------------------------------- 156
39.MAFF238905_F.oxysporumSchlechtendalf.sp.radicis-lycopersici_Tomato 156 ---------------------------------------- 156
40.MAFF150004_F.oxysporumSchlechtendalf.sp.spinaciae_Spinach     156 ---------------------------------------- 156
41.MAFF247034_F.oxysporumSchlechtendal__Goldenchain              156 ---------------------------------------- 156
42.MAFF245747_F.oxysporumSchlechtendalf.sp.callistephi__Chinaaster 156 ---------------------------------------- 156
43.MAFF305115_FoxysporumSchlechtendalf.sp.batatas__Sweatpotato   161 ---------------------------------------- 161
44.MAFF150126_F.asiaticum_Seed                                   135 ---------------------------------------- 135
45.MAFF246738_F.solani_Angelica                                  161 ---------------------------------------- 161
46.MAFF246664_F.cugenangense_Perilla                             156 ---------------------------------------- 156
47.MH582420.1F.solanistrainMRC256                                150 ---------------------------------------- 150
48.MAFF240361_F.babinda_Soil                                     158 ---------------------------------------- 158
49.MAFF242368_F.azukicola_Azukibean                              162 ---------------------------------------- 162
50.MAFF241312_F.asiaticum_Soil,welshonionfield                   135 ---------------------------------------- 135
51.LT548416.1_F.culmorumpartialtef1a                             131 ---------------------------------------- 131
52.MAFF150124_F.asiaticum__Wheat                                 135 ---------------------------------------- 135
53.MAFF238806_F.begoniae_Oncidiumsp                              172 ---------------------------------------- 172
54.MW594399.1_FusariumincarnatumisolateUD01C                     163 ---------------------------------------- 163
55.OP414923.1Pucciniagraminisf.sp.triticiisolateSHZPgt19         238 TTGAAACCCCCAAGTACTACGTCACCGTCATTGATGCCCC 277
56.MT027094.1_BipolarisoryzaestrainOrL-2                           0 ---------------------------------------- 0
57.ON734360.1_AlternariaalternataisolateH126                       0 ---------------------------------------- 0
58.LC333578.1_StemphyliumlycopersiciSOasp2                         0 ---------------------------------------- 0
59.HQ718583.1_Colletotrichumgloeosporioidesisolateq-1            450 TTGGCTGTTCGAAATCCTCTTTTTTGG--ATGGGCGCGAT 487
60.JN241603.1_AtheliarolfsiiisolateSR1                             0 ---------------------------------------- 0
61.KJ866474.1_RhizoctoniasolanistrainMHL-1                         0 ---------------------------------------- 0
62.JQ672424.1AlternariatriticinaisolateEGS17-061                   0 ---------------------------------------- 0
63.LT707559.1_P.capsicipartialtef1agene                            0 ---------------------------------------- 0
64.MW090051.1_CurvularialunatastrainCls-3                          0 ---------------------------------------- 0
65.DQ400892.1_Aspergillusterreus                                   0 ---------------------------------------- 0
66.DQ911416.1_Pythiumsp.quercumstrainPy292                         0 ---------------------------------------- 0
67.EU797495.1_Phytophthorasp.oaksoilPoland                         0 ---------------------------------------- 0
68.HM148321.1_Cladosporiumcucumerinum                              0 ---------------------------------------- 0
69.AF398888.1_SclerotiniasclerotiorumisolateSS1                    0 ---------------------------------------- 0
70.AF398888.1_S.sclerotiorumisolateSS1                             0 ---------------------------------------- 0
71.HPAB545908.1_Verticilliumnonalfalfaeisolate                     0 ---------------------------------------- 0
72.EF433315.1_CeratocystisfimbriatavoucherCMW15052               400 TCATTATTAGATGCTAACAGTTCTAAA--ACAGATGCCCC 437
73.MN159912.1_Botrytiscinerea                                      0 ---------------------------------------- 0
74.MF034741.1_PeltasterfructicolaisolateSRB92                      0 ---------------------------------------- 0
```

```
75.LC440360.1_CercosporaasparagiCOasp2                                  0 ---------------------------------------- 0
76.AY944105.1_MagnaportheoryzaeisolateSAG00T3()                         0 ---------------------------------------- 0
77.JX266586.1_CochliobolusmiyabeanusvoucherMFLUCC10-0733                0 ---------------------------------------- 0
78.MN393253.1_CorynesporacassiicolaisolateQHD001(MN393253.1UNVERIFIED)  0 ---------------------------------------- 0
79.MF375218.1_AgroatheliarolfsiiisolateBJB24                            0 ---------------------------------------- 0
80.MN106270.1_AgroatheliarolfsiistrainJ-12                              0 ---------------------------------------- 0
81.OQ732628.1_AgroatheliarolfsiiisolateBTCBSr3                          0 ---------------------------------------- 0
82.KY196185.1_ColletotrichumtruncatumstrainPAK53                      354 TTA--TGCCCCAA------------------------GAT 367
83.GU935835.1_ColletotrichumcoccodesisolateC96002                       1 -----------------------------GTCGTTGTTAT 11
84.MK085963.1_AlternariatenuissimaisolateSCCZ06                         0 ---------------------------------------- 0
85.MT548042.1_AlternarialongipesstrainKY_2019_012                       0 ---------------------------------------- 0
86.MN356465.1CalonectriamontanaisolateHSP4                              0 ---------------------------------------- 0
87.OL694224.1_CalonectriacanadianastrainF099                            0 ---------------------------------------- 0
88.MK803351.1_NeoscytalidiumdimidiatumstrainKale4-C                     0 ---------------------------------------- 0
89.ON376993.1_CurvulariachiangmaiensisisolateND00J7                     0 ---------------------------------------- 0
90.OQ383346.1_NeoscytalidiumdimidiatumisolateGKH-2                      0 ---------------------------------------- 0
91.MF662595.1_NeoscytalidiumnovaehollandiaeisolateNeNo1                 0 ---------------------------------------- 0
92.EF560588.1Melampsoralini                                           256 TCGAGACCCCCAAGTACTTTGTCACCGTCATTGATGCCCC 295
93.LC590862.1_NeoscytalidiumdimidiatumPSU-HP01TEF1                      0 ---------------------------------------- 0
94.KX278106.1_BotryosphaeriaqingyuanensisstrainCERC2947                 0 ---------------------------------------- 0
95.AJ578763.1_Blumeriagraminisf.sp.hordeicyp51                          0 ---------------------------------------- 0
96.MF490858.1_CurvulariadactylocteniicolastrainCPC28810                 0 ---------------------------------------- 0
97.KT287115.1_Bipolariscactivoraisolate3.8.6                            0 ---------------------------------------- 0
98.MT560940.1_CurvulariacactivorastrainHLGH0118                         0 ---------------------------------------- 0
99.OM714565.1_CurvulariaplantarumstrainM0134                            0 ---------------------------------------- 0
100.MN159911.1_BotrytiscinereaSICAUCC19-0003                            0 ---------------------------------------- 0
102.GU294713.1LasiodiplodiatheobromaestrainUCD2430TX                     0 ---------------------------------------- 0
103.KX868094.1_Mycosphaerellasp.isolateCRM20.1                          0 ---------------------------------------- 0
104.LC599478.1Pseudocercosporapini-densifloraeMUCC534                   0 ---------------------------------------- 0
105.N584698.1BipolarissetariaestrainKBS4-2                              0 ---------------------------------------- 0

2.OM160859.1_F.buharicum                                              128 ---------------------------------------- 128
1.LC727524.1_F.buharicum_OKI-1_Okura                                  159 ---------------------------------------- 159
3.KX302919.1_F.sublunatum                                             125 ---------------------------------------- 125
4.LT996094.1_F.convolutans                                            125 ---------------------------------------- 125
5.OM160861.1_F.abutilonis                                             127 ---------------------------------------- 127
6.OM160874.1_F.guadeloupense                                          126 ---------------------------------------- 126
7.MH392475.1_F.graminearum                                             80 ---------------------------------------- 80
8.MH582420.1_F.solani                                                 130 ---------------------------------------- 130
9.MAFF244605_F.oxysporum                                              126 ---------------------------------------- 126
10.MAFF237278_F.contaminatum_Hylocereus                               173 ---------------------------------------- 173
11.MAFF237649_F.concentricum__Riceroot                                166 ---------------------------------------- 166
12.MAFF237650_F.concentricum__Wheat                                   166 ---------------------------------------- 166
13.MAFF239869_F.mangiferae__Ryukyupine                                156 ---------------------------------------- 156
14.MAFF240460_F.fujikuroi_Passionfruit                                156 ---------------------------------------- 156
15.MAFF241317_F.graminearum_Wheat                                     135 ---------------------------------------- 135
```

```
16.MAFF242670_F.ipomoeae_Wheat                                           158 ---------------------------------------- 158
17.MAFF245129_F.concentricum_Fraxinus                                    156 ---------------------------------------- 156
18.MAFF245395_F.cugenangense_Rhubarb                                     156 ---------------------------------------- 156
19.MAFF246637_F.nirenbergiae_Strawberry                                  156 ---------------------------------------- 156
20.MAFF246672_F.nirenbergiae_ChinesePeony                                156 ---------------------------------------- 156
21.MAFF246697_F.commune_Urallicoriceroot                                 157 ---------------------------------------- 157
22.MAFF246729_F.falciforme_Angelica                                      161 ---------------------------------------- 161
23.MAFF247220_F.duplospermum__Euwallaceasp                               161 ---------------------------------------- 161
24.MAFF410760_F.odoratissimum_alpha                                      156 ---------------------------------------- 156
25.MAFF244605_FusariumoxysporumSchlechtendal_MAFF244605_Tomato           157 ---------------------------------------- 157
26.MAFF241326_F.asiaticum_Wheat                                          138 ---------------------------------------- 138
27.MAFF245014_F.asiaticum_Wildsoybean                                    135 ---------------------------------------- 135
28.MAFF150124_F.asiaticum__Wheat                                         135 ---------------------------------------- 135
29.OM135603.1F.algeriense                                                163 ---------------------------------------- 163
30.MAFF237465_F.penzigii_Aloe                                            166 ---------------------------------------- 166
31.MAFF103054_F.oxysporumSchlechtendalf.sp.cucumerinum_Cucumber          156 ---------------------------------------- 156
32.MAFF712246_F.oxysporumSchlechtendalf.sp.dianthi__Carnation            156 ---------------------------------------- 156
33.MAFF305558_F.oxysporumSchlechtendalf.sp.fragariae__Watermelon         156 ---------------------------------------- 156
34.MAFF744087_F.oxysporumSchlechtendalf.sp.lactucae__Lettuce             156 ---------------------------------------- 156
35.MAFF726924_F.oxysporumSchlechtendalf.sp.lagenariae_Whitefloweredgourd 156 ---------------------------------------- 156
36.MAFF744003_F.oxysporumSchlechtendalf.sp.lagenariae_Squash)            156 ---------------------------------------- 156
37.MAFF305122_F.oxysporumSchlechtendalf.sp.melonis__Melon                156 ---------------------------------------- 156
38.MAFF306714_F.oxysporumSchlechtendalf.sp.momordicae_Balsampear         156 ---------------------------------------- 156
39.MAFF238905_F.oxysporumSchlechtendalf.sp.radicis-lycopersici_Tomato    156 ---------------------------------------- 156
40.MAFF150004_F.oxysporumSchlechtendalf.sp.spinaciae_Spinach             156 ---------------------------------------- 156
41.MAFF247034_F.oxysporumSchlechtendal__Goldenchain                      156 ---------------------------------------- 156
42.MAFF245747_F.oxysporumSchlechtendalf.sp.callistephi__Chinaaster       156 ---------------------------------------- 156
43.MAFF305115_FoxysporumSchlechtendalf.sp.batatas__Sweatpotato           161 ---------------------------------------- 161
44.MAFF150126_F.asiaticum_Seed                                           135 ---------------------------------------- 135
45.MAFF246738_F.solani_Angelica                                          161 ---------------------------------------- 161
46.MAFF246664_F.cugenangense_Perilla                                     156 ---------------------------------------- 156
47.MH582420.1F.solanistrainMRC256                                        150 ---------------------------------------- 150
48.MAFF240361_F.babinda_Soil                                             158 ---------------------------------------- 158
49.MAFF242368_F.azukicola_Azukibean                                      162 ---------------------------------------- 162
50.MAFF241312_F.asiaticum_Soil,welshonionfield                           135 ---------------------------------------- 135
51.LT548416.1_F.culmorumpartialtef1a                                     131 ---------------------------------------- 131
52.MAFF150124_F.asiaticum__Wheat                                         135 ---------------------------------------- 135
53.MAFF238806_F.begoniae_Oncidiumsp                                      172 ---------------------------------------- 172
54.MW594399.1_FusariumincarnatumisolateUD01C                             163 ---------------------------------------- 163
55.OP414923.1Pucciniagraminisf.sp.triticiisolateSHZPgt19                 278 CGGACATC-------------------------------- 285
56.MT027094.1_BipolarisoryzaestrainOrL-2                                   0 ---------------------------------------- 0
57.ON734360.1_AlternariaalternataisolateH126                               0 ---------------------------------------- 0
58.LC333578.1_StemphyliumlycopersiciSOasp2                                 0 ---------------------------------------- 0
59.HQ718583.1_Colletotrichumgloeosporioidesisolateq-1                    488 TTTGCAGTG--TGGAAATCTCTCGCAAGGACCACGCACTA 525
60.JN241603.1_AtheliarolfsiiisolateSR1                                     0 ---------------------------------------- 0
61.KJ866474.1_RhizoctoniasolanistrainMHL-1                                 0 ---------------------------------------- 0
```

62.JQ672424.1AlternariatriticinaisolateEGS17-061	0	--	0
63.LT707559.1_P.capsicipartialtef1agene	0	--	0
64.MW090051.1_CurvularialunatastrainCls-3	0	--	0
65.DQ400892.1_Aspergillusterreus	0	--	0
66.DQ911416.1_Pythiumsp.quercumstrainPy292	0	--	0
67.EU797495.1_Phytophthorasp.oaksoilPoland	0	--	0
68.HM148321.1_Cladosporiumcucumerinum	0	--	0
69.AF398888.1_SclerotiniasclerotiorumisolateSS1	0	--	0
70.AF398888.1_S.sclerotiorumisolateSS1	0	--	0
71.HPAB545908.1_Verticilliumnonalfalfaeisolate	0	--	0
72.EF433315.1_CeratocystisfimbriatavoucherCMW15052	438	TGGTCACAGAGATTTCATCAAGAACATGATCACTGGTA-C	476
73.MN159912.1_Botrytiscinerea	0	--	0
74.MF034741.1_PeltasterfructicolaisolateSRB92	0	--	0
75.LC440360.1_CercosporaasparagiCOasp2	0	--	0
76.AY944105.1_MagnaportheoryzaeisolateSAG00T3()	0	--	0
77.JX266586.1_CochliobolusmiyabeanusvoucherMFLUCC10-0733	0	--	0
78.MN393253.1_CorynesporacassiicolaisolateQHD001(MN393253.1UNVERIFIED)	0	--	0
79.MF375218.1_AgroatheliarolfsiiisolateBJB24	0	--	0
80.MN106270.1_AgroatheliarolfsiistrainJ-12	0	--	0
81.OQ732628.1_AgroatheliarolfsiiisolateBTCBSr3	0	--	0
82.KY196185.1_ColletotrichumtruncatumstrainPAK53	368	TTTTGTGTGCGTGAAAATTTTTTCCGTGTGACAAGTACTA	407
83.GU935835.1_ColletotrichumcoccodesisolateC96002	12	TGGTCACGTCGATTCCGGCAAGTCGACCACCACTGTAAGC	51
84.MK085963.1_AlternariatenuissimaisolateSCCZ06	0	--	0
85.MT548042.1_AlternarialongipesstrainKY_2019_012	0	--	0
86.MN356465.1CalonectriamontanaisolateHSP4	0	--	0
87.OL694224.1_CalonectriacanadianastrainF099	0	--	0
88.MK803351.1_NeoscytalidiumdimidiatumstrainKale4-C	0	--	0
89.ON376993.1_CurvulariachiangmaiensisisolateND00J7	0	--	0
90.OQ383346.1_NeoscytalidiumdimidiatumisolateGKH-2	0	--	0
91.MF662595.1_NeoscytalidiumnovaehollandiaeisolateNeNo1	0	--	0
92.EF560588.1Melampsoralini	296	AGGACATCGTGATTTCATCAAAAACATGATCACTGGAA-C	334
93.LC590862.1_NeoscytalidiumdimidiatumPSU-HP01TEF1	0	--	0
94.KX278106.1_BotryosphaeriaqingyuanensisstrainCERC2947	0	--	0
95.AJ578763.1_Blumeriagraminisf.sp.hordeicyp51	0	--	0
96.MF490858.1_CurvulariadactylocteniicolastrainCPC28810	0	--	0
97.KT287115.1_Bipolariscactivoraisolate3.8.6	0	--	0
98.MT560940.1_CurvulariacactivorastrainHLGH0118	0	--	0
99.OM714565.1_CurvulariaplantarumstrainM0134	0	--	0
100.MN159911.1_BotrytiscinereaSICAUCC19-0003	0	--	0
102.GU294713.1LasiodiplodiatheobromaestrainUCD2430TX	0	--	0
103.KX868094.1_Mycosphaerellasp.isolateCRM20.1	0	--	0
104.LC599478.1Pseudocercosporapini-densifloraeMUCC534	0	--	0
105.N584698.1BipolarissetariaestrainKBS4-2	0	--	0
2.OM160859.1_F.buharicum	128	--	128
1.LC727524.1_F.buharicum_OKI-1_Okura	159	--	159

```
3.KX302919.1_F.sublunatum                                          125 ---------------------------------------- 125
4.LT996094.1_F.convolutans                                         125 ---------------------------------------- 125
5.OM160861.1_F.abutilonis                                          127 ---------------------------------------- 127
6.OM160874.1_F.guadeloupense                                       126 ---------------------------------------- 126
7.MH392475.1_F.graminearum                                          80 ---------------------------------------- 80
8.MH582420.1_F.solani                                              130 ---------------------------------------- 130
9.MAFF244605_F.oxysporum                                           126 ---------------------------------------- 126
10.MAFF237278_F.contaminatum_Hylocereus                            173 ---------------------------------------- 173
11.MAFF237649_F.concentricum__Riceroot                             166 ---------------------------------------- 166
12.MAFF237650_F.concentricum__Wheat                                166 ---------------------------------------- 166
13.MAFF239869_F.mangiferae__Ryukyupine                             156 ---------------------------------------- 156
14.MAFF240460_F.fujikuroi_Passionfruit                             156 ---------------------------------------- 156
15.MAFF241317_F.graminearum_Wheat                                  135 ---------------------------------------- 135
16.MAFF242670_F.ipomoeae_Wheat                                     158 ---------------------------------------- 158
17.MAFF245129_F.concentricum_Fraxinus                              156 ---------------------------------------- 156
18.MAFF245395_F.cugenangense_Rhubarb                               156 ---------------------------------------- 156
19.MAFF246637_F.nirenbergiae_Strawberry                            156 ---------------------------------------- 156
20.MAFF246672_F.nirenbergiae_ChinesePeony                          156 ---------------------------------------- 156
21.MAFF246697_F.commune_Urallicoriceroot                           157 ---------------------------------------- 157
22.MAFF246729_F.falciforme_Angelica                                161 ---------------------------------------- 161
23.MAFF247220_F.duplospermum__Euwallaceasp                         161 ---------------------------------------- 161
24.MAFF410760_F.odoratissimum_alpha                                156 ---------------------------------------- 156
25.MAFF244605_FusariumoxysporumSchlechtendal_MAFF244605_Tomato     157 ---------------------------------------- 157
26.MAFF241326_F.asiaticum_Wheat                                    138 ---------------------------------------- 138
27.MAFF245014_F.asiaticum_Wildsoybean                              135 ---------------------------------------- 135
28.MAFF150124_F.asiaticum__Wheat                                   135 ---------------------------------------- 135
29.OM135603.1F.algeriense                                          163 ---------------------------------------- 163
30.MAFF237465_F.penzigii_Aloe                                      166 ---------------------------------------- 166
31.MAFF103054_F.oxysporumSchlechtendalf.sp.cucumerinum_Cucumber    156 ---------------------------------------- 156
32.MAFF712246_F.oxysporumSchlechtendalf.sp.dianthi__Carnation      156 ---------------------------------------- 156
33.MAFF305558_F.oxysporumSchlechtendalf.sp.fragariae__Watermelon   156 ---------------------------------------- 156
34.MAFF744087_F.oxysporumSchlechtendalf.sp.lactucae__Lettuce       156 ---------------------------------------- 156
35.MAFF726924_F.oxysporumSchlechtendalf.sp.lagenariae_Whiteflaweredgourd 156 ---------------------------------------- 156
36.MAFF744003_F.oxysporumSchlechtendalf.sp.lagenariae_Squash)      156 ---------------------------------------- 156
37.MAFF305122_F.oxysporumSchlechtendalf.sp.melonis__Melon          156 ---------------------------------------- 156
38.MAFF306714_F.oxysporumSchlechtendalf.sp.momordicae_Balsampear   156 ---------------------------------------- 156
39.MAFF238905_F.oxysporumSchlechtendalf.sp.radicis-lycopersici_Tomato 156 ---------------------------------------- 156
40.MAFF150004_F.oxysporumSchlechtendalf.sp.spinaciae_Spinach       156 ---------------------------------------- 156
41.MAFF247034_F.oxysporumSchlechtendal__Goldenchain                156 ---------------------------------------- 156
42.MAFF245747_F.oxysporumSchlechtendalf.sp.callistephi__Chinaaster 156 ---------------------------------------- 156
43.MAFF305115_FoxysporumSchlechtendalf.sp.batatas__Sweatpotato     161 ---------------------------------------- 161
44.MAFF150126_F.asiaticum_Seed                                     135 ---------------------------------------- 135
45.MAFF246738_F.solani_Angelica                                    161 ---------------------------------------- 161
46.MAFF246664_F.cugenangense_Perilla                               156 ---------------------------------------- 156
47.MH582420.1F.solanistrainMRC256                                  150 ---------------------------------------- 150
48.MAFF240361_F.babinda_Soil                                       158 ---------------------------------------- 158
```

```
49.MAFF242368_F.azukicola_Azukibean                                    162 ---------------------------------------- 162
50.MAFF241312_F.asiaticum_Soil,welshonionfield                         135 ---------------------------------------- 135
51.LT548416.1_F.culmorumpartialtef1a                                   131 ---------------------------------------- 131
52.MAFF150124_F.asiaticum__Wheat                                       135 ---------------------------------------- 135
53.MAFF238806_F.begoniae_Oncidiumsp                                    172 ---------------------------------------- 172
54.MW594399.1_FusariumincarnatumisolateUD01C                           163 ---------------------------------------- 163
55.OP414923.1Pucciniagraminisf.sp.triticiisolateSHZPgt19               285 ---------------------------------------- 285
56.MT027094.1_BipolarisoryzaestrainOrL-2                                 0 ---------------------------------------- 0
57.ON734360.1_AlternariaalternataisolateH126                             0 ---------------------------------------- 0
58.LC333578.1_StemphyliumlycopersiciSOasp2                               0 ---------------------------------------- 0
59.HQ718583.1_Colletotrichumgloeosporioidesisolateq-1                  526 ACATGTCATTCAGACGCTCCCGGTCACCGTGACTTCATCA 565
60.JN241603.1_AtheliarolfsiiisolateSR1                                   0 ---------------------------------------- 0
61.KJ866474.1_RhizoctoniasolanistrainMHL-1                               0 ---------------------------------------- 0
62.JQ672424.1AlternariatriticinaisolateEGS17-061                         0 ---------------------------------------- 0
63.LT707559.1_P.capsicipartialtef1agene                                  0 ---------------------------------------- 0
64.MW090051.1_CurvularialunatastrainCls-3                                0 ---------------------------------------- 0
65.DQ400892.1_Aspergillusterreus                                         0 ---------------------------------------- 0
66.DQ911416.1_Pythiumsp.quercumstrainPy292                               0 ---------------------------------------- 0
67.EU797495.1_Phytophthorasp.oaksoilPoland                               0 ---------------------------------------- 0
68.HM148321.1_Cladosporiumcucumerinum                                    0 ---------------------------------------- 0
69.AF398888.1_SclerotiniasclerotiorumisolateSS1                          0 ---------------------------------------- 0
70.AF398888.1_S.sclerotiorumisolateSS1                                   0 ---------------------------------------- 0
71.HPAB545908.1_Verticilliumnonalfalfaeisolate                           0 ---------------------------------------- 0
72.EF433315.1_CeratocystisfimbriatavoucherCMW15052                     477 TTCCCAGGCTGACTGCGCT--ATTCTGATCATTGCTGCCG 514
73.MN159912.1_Botrytiscinerea                                            0 ---------------------------------------- 0
74.MF034741.1_PeltasterfructicolaisolateSRB92                            0 ---------------------------------------- 0
75.LC440360.1_CercosporaasparagiCOasp2                                   0 ---------------------------------------- 0
76.AY944105.1_MagnaportheoryzaeisolateSAG00T3()                          0 ---------------------------------------- 0
77.JX266586.1_CochliobolusmiyabeanusvoucherMFLUCC10-0733                 0 ---------------------------------------- 0
78.MN393253.1_CorynesporacassiicolaisolateQHD001(MN393253.1UNVERIFIED)   0 ---------------------------------------- 0
79.MF375218.1_AgroatheliarolfsiiisolateBJB24                             0 ---------------------------------------- 0
80.MN106270.1_AgroatheliarolfsiistrainJ-12                               0 ---------------------------------------- 0
81.OQ732628.1_AgroatheliarolfsiiisolateBTCBSr3                           0 ---------------------------------------- 0
82.KY196185.1_ColletotrichumtruncatumstrainPAK53                       408 ACA-GAGAC-CAGACGCTCCCGGTCACCGTGACTTCATCA 445
83.GU935835.1_ColletotrichumcoccodesisolateC96002                       52 TTTCCCGTCCCATCGCTCTCCAGCCACCACATTGCTGACA 91
84.MK085963.1_AlternariatenuissimaisolateSCCZ06                          0 ---------------------------------------- 0
85.MT548042.1_AlternarialongipesstrainKY_2019_012                        0 ---------------------------------------- 0
86.MN356465.1CalonectriamontanaisolateHSP4                               0 ---------------------------------------- 0
87.OL694224.1_CalonectriacanadianastrainF099                             0 ---------------------------------------- 0
88.MK803351.1_NeoscytalidiumdimidiatumstrainKale4-C                      0 ---------------------------------------- 0
89.ON376993.1_CurvulariachiangmaiensisisolateND00J7                      0 ---------------------------------------- 0
90.OQ383346.1_NeoscytalidiumdimidiatumisolateGKH-2                       0 ---------------------------------------- 0
91.MF662595.1_NeoscytalidiumnovaehollandiaeisolateNeNo1                  0 ---------------------------------------- 0
92.EF560588.1Melampsoralini                                            335 TTCCCAGGCCGATTGTGCT--ATCTTGATCATTGCCTCCG 372
93.LC590862.1_NeoscytalidiumdimidiatumPSU-HP01TEF1                       0 ---------------------------------------- 0
94.KX278106.1_BotryosphaeriaqingyuanensisstrainCERC2947                  0 ---------------------------------------- 0
```

```
95.AJ578763.1_Blumeriagraminisf.sp.hordeicyp51                        0 ---------------------------------------- 0
96.MF490858.1_CurvulariadactylocteniicolastrainCPC28810               0 ---------------------------------------- 0
97.KT287115.1_Bipolariscactivoraisolate3.8.6                          0 ---------------------------------------- 0
98.MT560940.1_CurvulariacactivorastrainHLGH0118                       0 ---------------------------------------- 0
99.OM714565.1_CurvulariaplantarumstrainM0134                          0 ---------------------------------------- 0
100.MN159911.1_BotrytiscinereaSICAUCC19-0003                          0 ---------------------------------------- 0
102.GU294713.1LasiodiplodiatheobromaestrainUCD2430TX                  0 ---------------------------------------- 0
103.KX868094.1_Mycosphaerellasp.isolateCRM20.1                        0 ---------------------------------------- 0
104.LC599478.1Pseudocercosporapini-densifloraeMUCC534                 0 ---------------------------------------- 0
105.N584698.1BipolarissetariaestrainKBS4-2                            0 ---------------------------------------- 0

2.OM160859.1_F.buharicum                                            129 --------------GGTCACTTGATCTACCAGTGCGGTGG 154
1.LC727524.1_F.buharicum_OKI-1_Okura                                160 --------------GGTCACTTGATCTACCAGTGCGGTGG 185
3.KX302919.1_F.sublunatum                                           126 --------------GGTCACTTGATCTACCAGTGCGGTGG 151
4.LT996094.1_F.convolutans                                          126 --------------GGTCACTTGATCTACCAGTGCGGTGG 151
5.OM160861.1_F.abutilonis                                           128 --------------GGTCACTTGATCTACCAGTGCGGTGG 153
6.OM160874.1_F.guadeloupense                                        127 --------------GGTCACTTGATCTACCAGTGCGGTGG 152
7.MH392475.1_F.graminearum                                           81 --------------GGTCACTTGATCTACCAGTGCGGTGG 106
8.MH582420.1_F.solani                                               131 --------------GGTCACTTGATCTACCAGTGCGGTGG 156
9.MAFF244605_F.oxysporum                                            127 --------------GGTCACTTGATCTACCAGTGCGGTGG 152
10.MAFF237278_F.contaminatum_Hylocereus                             174 --------------GGTCACTTGATCTACCAGTGCGGTGG 199
11.MAFF237649_F.concentricum__Riceroot                              167 --------------GGTCACTTGATCTACCAGTGCGGTGG 192
12.MAFF237650_F.concentricum__Wheat                                 167 --------------GGTCACTTGATCTACCAGTGCGGTGG 192
13.MAFF239869_F.mangiferae__Ryukyupine                              157 --------------GGTCACTTGATCTACCAGTGCGGTGG 182
14.MAFF240460_F.fujikuroi_Passionfruit                              157 --------------GGTCACTTGATCTACCAGTGCGGTGG 182
15.MAFF241317_F.graminearum_Wheat                                   136 --------------GGTCACTTGATCTACCAGTGCGGTGG 161
16.MAFF242670_F.ipomoeae_Wheat                                      159 --------------GGTCACTTGATCTACCAGTGCGGTGG 184
17.MAFF245129_F.concentricum_Fraxinus                               157 --------------GGTCACTTGATCTACCAGTGCGGTGG 182
18.MAFF245395_F.cugenangense_Rhubarb                                157 --------------GGTCACTTGATCTACCAGTGCGGTGG 182
19.MAFF246637_F.nirenbergiae_Strawberry                             157 --------------GGTCACTTGATCTACCAGTGCGGTGG 182
20.MAFF246672_F.nirenbergiae_ChinesePeony                           157 --------------GGTCACTTGATCTACCAGTGCGGTGG 182
21.MAFF246697_F.commune_Urallicoriceroot                            158 --------------GGTCACTTGATCTACCAGTGCGGTGG 183
22.MAFF246729_F.falciforme_Angelica                                 162 --------------GGTCACTTGATCTACCAGTGCGGTGG 187
23.MAFF247220_F.duplospermum__Euwallaceasp                          162 --------------GGTCACTTGATCTACCAGTGCGGTGG 187
24.MAFF410760_F.odoratissimum_alpha                                 157 --------------GGTCACTTGATCTACCAGTGCGGTGG 182
25.MAFF244605_FusariumoxysporumSchlechtendal_MAFF244605_Tomato      158 --------------GGTCACTTGATCTACCAGTGCGGTGG 183
26.MAFF241326_F.asiaticum_Wheat                                     139 --------------GGTCACTTGATCTACCAGTGCGGTGG 164
27.MAFF245014_F.asiaticum_Wildsoybean                               136 --------------GGTCACTTGATCTACCAGTGCGGTGG 161
28.MAFF150124_F.asiaticum__Wheat                                    136 --------------GGTCACTTGATCTACCAGTGCGGTGG 161
29.OM135603.1F.algeriense                                           164 --------------GGTCACTTGATCTACCAGTGCGGTGG 189
30.MAFF237465_F.penzigii_Aloe                                       167 --------------GGTCACTTGATCTACCAGTGCGGTGG 192
31.MAFF103054_F.oxysporumSchlechtendalf.sp.cucumerinum_Cucumber     157 --------------GGTCACTTGATCTACCAGTGCGGTGG 182
32.MAFF712246_F.oxysporumSchlechtendalf.sp.dianthi__Carnation       157 --------------GGTCACTTGATCTACCAGTGCGGTGG 182
33.MAFF305558_F.oxysporumSchlechtendalf.sp.fragariae__Watermelon    157 --------------GGTCACTTGATCTACCAGTGCGGTGG 182
34.MAFF744087_F.oxysporumSchlechtendalf.sp.lactucae__Lettuce        157 --------------GGTCACTTGATCTACCAGTGCGGTGG 182
35.MAFF726924_F.oxysporumSchlechtendalf.sp.lagenariae_Whitefloweredgourd 157 --------------GGTCACTTGATCTACCAGTGCGGTGG 182
```

36.MAFF744003_F.oxysporumSchlechtendalf.sp.lagenariae_Squash)	157	--------------GGTCACTTGATCTACCAGTGCGGTGG	182
37.MAFF305122_F.oxysporumSchlechtendalf.sp.melonis__Melon	157	--------------GGTCACTTGATCTACCAGTGCGGTGG	182
38.MAFF306714_F.oxysporumSchlechtendalf.sp.momordicae_Balsampear	157	--------------GGTCACTTGATCTACCAGTGCGGTGG	182
39.MAFF238905_F.oxysporumSchlechtendalf.sp.radicis-lycopersici_Tomato	157	--------------GGTCACTTGATCTACCAGTGCGGTGG	182
40.MAFF150004_F.oxysporumSchlechtendalf.sp.spinaciae_Spinach	157	--------------GGTCACTTGATCTACCAGTGCGGTGG	182
41.MAFF247034_F.oxysporumSchlechtendal__Goldenchain	157	--------------GGTCACTTGATCTACCAGTGCGGTGG	182
42.MAFF245747_F.oxysporumSchlechtendalf.sp.callistephi__Chinaaster	157	--------------GGTCACTTGATCTACCAGTGCGGTGG	182
43.MAFF305115_FoxysporumSchlechtendalf.sp.batatas__Sweatpotato	162	--------------GGTCACTTGATCTACCAGTGCGGTGG	187
44.MAFF150126_F.asiaticum_Seed	136	--------------GGTCACTTGATCTACCAGTGCGGTGG	161
45.MAFF246738_F.solani_Angelica	162	--------------GGTCACTTGATCTACCAGTGCGGTGG	187
46.MAFF246664_F.cugenangense_Perilla	157	--------------GGTCACTTGATCTACCAGTGCGGTGG	182
47.MH582420.1F.solanistrainMRC256	151	--------------GGTCACTTGATCTACCAGTGCGGTGG	176
48.MAFF240361_F.babinda_Soil	159	--------------GGTCACTTGATCTACCAGTGCGGTGG	184
49.MAFF242368_F.azukicola_Azukibean	163	--------------GGTCACTTGATCTACCAGTGCGGTGG	188
50.MAFF241312_F.asiaticum_Soil,welshonionfield	136	--------------GGTCACTTGATCTACCAGTGCGGTGG	161
51.LT548416.1_F.culmorumpartialtef1a	132	--------------GGTCACTTGATCTACCAGTGCGGTGG	157
52.MAFF150124_F.asiaticum__Wheat	136	--------------GGTCACTTGATCTACCAGTGCGGTGG	161
53.MAFF238806_F.begoniae_Oncidiumsp	173	--------------GGTCACTTGATCTACCAGTGCGGTGG	198
54.MW594399.1_FusariumincarnatumisolateUD01C	164	--------------GGTCACTTGATCTACCAGTGCGGTGG	189
55.OP414923.1Pucciniagraminisf.sp.triticiisolateSHZPgt19	285	--	285
56.MT027094.1_BipolarisoryzaestrainOrL-2	0	--	0
57.ON734360.1_AlternariaalternataisolateH126	0	--	0
58.LC333578.1_StemphyliumlycopersiciSOasp2	0	--	0
59.HQ718583.1_Colletotrichumgloeosporioidesisolateq-1	566	AG--------------------------------------	567
60.JN241603.1_AtheliarolfsiiisolateSR1	0	--	0
61.KJ866474.1_RhizoctoniasolanistrainMHL-1	0	--	0
62.JQ672424.1AlternariatriticinaisolateEGS17-061	0	--	0
63.LT707559.1_P.capsicipartialtef1agene	0	--	0
64.MW090051.1_CurvularialunatastrainCls-3	0	--	0
65.DQ400892.1_Aspergillusterreus	0	--	0
66.DQ911416.1_Pythiumsp.quercumstrainPy292	0	--	0
67.EU797495.1_Phytophthorasp.oaksoilPoland	0	--	0
68.HM148321.1_Cladosporiumcucumerinum	0	--	0
69.AF398888.1_SclerotiniasclerotiorumisolateSS1	0	--	0
70.AF398888.1_S.sclerotiorumisolateSS1	0	--	0
71.HPAB545908.1_Verticilliumnonalfalfaeisolate	0	--	0
72.EF433315.1_CeratocystisfimbriatavoucherCMW15052	515	GTACCGGT--------------------------------	522
73.MN159912.1_Botrytiscinerea	0	--	0
74.MF034741.1_PeltasterfructicolaisolateSRB92	0	--	0
75.LC440360.1_CercosporaasparagiCOasp2	0	--	0
76.AY944105.1_MagnaportheoryzaeisolateSAG00T3()	0	--	0
77.JX266586.1_CochliobolusmiyabeanusvoucherMFLUCC10-0733	0	--	0
78.MN393253.1_CorynesporacassiicolaisolateQHD001(MN393253.1UNVERIFIED)	0	--	0
79.MF375218.1_AgroatheliarolfsiiisolateBJB24	0	--	0
80.MN106270.1_AgroatheliarolfsiistrainJ-12	0	--	0
81.OQ732628.1_AgroatheliarolfsiiisolateBTCBSr3	0	--	0

```
82.KY196185.1_ColletotrichumtruncatumstrainPAK53            446 AGAACATGATCACTGGTACCTCC----------------- 468
83.GU935835.1_ColletotrichumcoccodesisolateC96002            92 TCTTCCTTTTGTAGGGTCACTTGATCTACCAGTGCGGTGG 131
84.MK085963.1_AlternariatenuissimaisolateSCCZ06               0 ---------------------------------------- 0
85.MT548042.1_AlternarialongipesstrainKY_2019_012             0 ---------------------------------------- 0
86.MN356465.1CalonectriamontanaisolateHSP4                    0 ---------------------------------------- 0
87.OL694224.1_CalonectriacanadianastrainF099                  0 ---------------------------------------- 0
88.MK803351.1_NeoscytalidiumdimidiatumstrainKale4-C           0 ---------------------------------------- 0
89.ON376993.1_CurvulariachiangmaiensisisolateND00J7           0 ---------------------------------------- 0
90.OQ383346.1_NeoscytalidiumdimidiatumisolateGKH-2            0 ---------------------------------------- 0
91.MF662595.1_NeoscytalidiumnovaehollandiaeisolateNeNo1       0 ---------------------------------------- 0
92.EF560588.1Melampsoralini                                 373 GTGTCGGT-------------------------------- 380
93.LC590862.1_NeoscytalidiumdimidiatumPSU-HP01TEF1            0 ---------------------------------------- 0
94.KX278106.1_BotryosphaeriaqingyuanensisstrainCERC2947       0 ---------------------------------------- 0
95.AJ578763.1_Blumeriagraminisf.sp.hordeicyp51                0 ---------------------------------------- 0
96.MF490858.1_CurvulariadactylocteniicolastrainCPC28810       0 ---------------------------------------- 0
97.KT287115.1_Bipolariscactivoraisolate3.8.6                  0 ---------------------------------------- 0
98.MT560940.1_CurvulariacactivorastrainHLGH0118               0 ---------------------------------------- 0
99.OM714565.1_CurvulariaplantarumstrainM0134                  0 ---------------------------------------- 0
100.MN159911.1_BotrytiscinereaSICAUCC19-0003                  0 ---------------------------------------- 0
102.GU294713.1LasiodiplodiatheobromaestrainUCD2430TX          0 ---------------------------------------- 0
103.KX868094.1_Mycosphaerellasp.isolateCRM20.1                0 ---------------------------------------- 0
104.LC599478.1Pseudocercosporapini-densifloraeMUCC534         0 ---------------------------------------- 0
105.N584698.1BipolarissetariaestrainKBS4-2                    0 ---------------------------------------- 0

2.OM160859.1_F.buharicum                                    155 TATCGACAAGCGAACCATCGAGAAGTTCGAGAAGGTTGGT 194
1.LC727524.1_F.buharicum_OKI-1_Okura                        186 TATCGACAAGCGAACCATCGAGAAGTTCGAGAAGGTTGGT 225
3.KX302919.1_F.sublunatum                                   152 TATCGACAAGCGAACCATCGAGAAGTTCGAGAAGGTTGGT 191
4.LT996094.1_F.convolutans                                  152 TATCGACAAGCGAACCATCGAGAAGTTCGAGAAGGTTGGT 191
5.OM160861.1_F.abutilonis                                   154 TATCGACAAGCGAACCATCGAGAAGTTCGAGAAGGTTGGT 193
6.OM160874.1_F.guadeloupense                                153 TATCGACAAGCGAACCATCGAGAAGTTCGAGAAGGTTAGT 192
7.MH392475.1_F.graminearum                                  107 TATCGACAAGCGAACCATCGAGAAGTTCGAGAAGGTTGGT 146
8.MH582420.1_F.solani                                       157 TATCGACAAGCGAACCATCGAGAAGTTCGAGAAGGTTGGT 196
9.MAFF244605_F.oxysporum                                    153 TATCGACAAGCGAACCATCGAGAAGTTCGAGAAGGTTAGT 192
10.MAFF237278_F.contaminatum_Hylocereus                     200 TATCGACAAGCGAACCATCGAGAAGTTCGAGAAGGTTAGT 239
11.MAFF237649_F.concentricum__Riceroot                      193 TATCGACAAGCGAACCATCGAGAAGTTCGAGAAGGTTAGT 232
12.MAFF237650_F.concentricum__Wheat                         193 TATCGACAAGCGAACCATCGAGAAGTTCGAGAAGGTTAGT 232
13.MAFF239869_F.mangiferae__Ryukyupine                      183 TATCGACAAGCGAACCATCGAGAAGTTCGAGAAGGTTAGT 222
14.MAFF240460_F.fujikuroi_Passionfruit                      183 TATCGACAAGCGAACCATCGAGAAGTTCGAGAAGGTCAGT 222
15.MAFF241317_F.graminearum_Wheat                           162 TATCGACAAGCGAACCATCGAGAAGTTCGAGAAGGTTGGT 201
16.MAFF242670_F.ipomoeae_Wheat                              185 TATCGACAAGCGAACCATCGAGAAGTTCGAGAAGGTTGGT 224
17.MAFF245129_F.concentricum_Fraxinus                       183 TATCGACAAGCGAACCATCGAGAAGTTCGAGAAGGTTAGT 222
18.MAFF245395_F.cugenangense_Rhubarb                        183 TATCGACAAGCGAACCATCGAGAAGTTCGAGAAGGTTAGT 222
19.MAFF246637_F.nirenbergiae_Strawberry                     183 TATCGATAAGCGAACCATCGAGAAGTTCGAGAAGGTTAGT 222
20.MAFF246672_F.nirenbergiae_ChinesePeony                   183 TATCGATAAGCGAACCATCGAGAAGTTCGAGAAGGTTAGT 222
21.MAFF246697_F.commune_Urallicoriceroot                    184 TATCGACAAGCGAACCATCGAGAAGTTCGAGAAGGTTAGT 223
22.MAFF246729_F.falciforme_Angelica                         188 TATCGACAAGCGAACCATCGAGAAGTTCGAGAAGGTTGGT 227
```

```
23.MAFF247220_F.duplospermum__Euwallaceasp                           188 TATCGACAAGCGAACCATCGAGAAGTTCGAGAAGGTTGGT 227
24.MAFF410760_F.odoratissimum_alpha                                  183 TATCGACAAGCGAACCATCGAGAAGTTCGAGAAGGTTAGT 222
25.MAFF244605_FusariumoxysporumSchlechtendal_MAFF244605_Tomato       184 TATCGACAAGCGAACCATCGAGAAGTTCGAGAAGGTTAGT 223
26.MAFF241326_F.asiaticum_Wheat                                      165 TATCGACAAGCGAACCATCGAGAAGTTCGAGAAGGTTGGT 204
27.MAFF245014_F.asiaticum_Wildsoybean                                162 TATCGACAAGCGAACCATCGAGAAGTTCGAGAAGGTTGGT 201
28.MAFF150124_F.asiaticum__Wheat                                     162 TATCGACAAGCGAACCATCGAGAAGTTCGAGAAGGTTGGT 201
29.OM135603.1F.algeriense                                            190 TATCGACAAGCGAACCATCGAGAAGTTCGAGAAGGTTAGT 229
30.MAFF237465_F.penzigii_Aloe                                        193 TATCGACAAGCGAACCATCGAGAAGTTCGAGAAGGTAGGT 232
31.MAFF103054_F.oxysporumSchlechtendalf.sp.cucumerinum_Cucumber      183 TATCGACAAGCGAACCATCGAGAAGTTCGAGAAGGTTAGT 222
32.MAFF712246_F.oxysporumSchlechtendalf.sp.dianthi__Carnation        183 TATCGACAAGCGAACCATCGAGAAGTTCGAGAAGGTTAGT 222
33.MAFF305558_F.oxysporumSchlechtendalf.sp.fragariae__Watermelon     183 TATCGACAAGCGAACCATCGAGAAGTTCGAGAAGGTTAGT 222
34.MAFF744087_F.oxysporumSchlechtendalf.sp.lactucae__Lettuce         183 TATCGACAAGCGAACCATCGAGAAGTTCGAGAAGGTTAGT 222
35.MAFF726924_F.oxysporumSchlechtendalf.sp.lagenariae_Whitefloweredgourd 183 TATCGACAAGCGAACCATCGAGAAGTTCGAGAAGGTTAGT 222
36.MAFF744003_F.oxysporumSchlechtendalf.sp.lagenariae_Squash)        183 TATCGACAAGCGAACCATCGAGAAGTTCGAGAAGGTTAGT 222
37.MAFF305122_F.oxysporumSchlechtendalf.sp.melonis__Melon            183 TATCGACAAGCGAACCATCGAGAAGTTCGAGAAGGTTAGT 222
38.MAFF306714_F.oxysporumSchlechtendalf.sp.momordicae_Balsampear     183 TATCGACAAGCGAACCATCGAGAAGTTCGAGAAGGTTAGT 222
39.MAFF238905_F.oxysporumSchlechtendalf.sp.radicis-lycopersici_Tomato 183 TATCGACAAGCGAACCATCGAGAAGTTCGAGAAGGTTAGT 222
40.MAFF150004_F.oxysporumSchlechtendalf.sp.spinaciae_Spinach         183 TATCGACAAGCGAACCATCGAGAAGTTCGAGAAGGTTAGT 222
41.MAFF247034_F.oxysporumSchlechtendal__Goldenchain                  183 TATCGACAAGCGAACCATCGAGAAGTTCGAGAAGGTTAGT 222
42.MAFF245747_F.oxysporumSchlechtendalf.sp.callistephi__Chinaaster   183 TATCGACAAGCGAACCATCGAGAAGTTCGAGAAGGTTAGT 222
43.MAFF305115_FoxysporumSchlechtendalf.sp.batatas__Sweatpotato       188 TATCGACAAGCGAACCATCGAGAAGTTCGAGAAGGTTGGT 227
44.MAFF150126_F.asiaticum_Seed                                       162 TATCGACAAGCGAACCATCGAGAAGTTCGAGAAGGTTGGT 201
45.MAFF246738_F.solani_Angelica                                      188 TATCGACAAGCGAACCATCGAGAAGTTCGAGAAGGTTGGT 227
46.MAFF246664_F.cugenangense_Perilla                                 183 TATCGACAAGCGAACCATCGAGAAGTTCGAGAAGGTTAGT 222
47.MH582420.1F.solanistrainMRC256                                    177 TATCGACAAGCGAACCATCGAGAAGTTCGAGAAGGTTGGT 216
48.MAFF240361_F.babinda_Soil                                         185 TATCGACAAGCGAACCATCGAGAAGTTCGAGAAGGTTAGT 224
49.MAFF242368_F.azukicola_Azukibean                                  189 TATCGACAAGCGAACCATCGAGAAGTTCGAGAAGGTTGGT 228
50.MAFF241312_F.asiaticum_Soil,welshonionfield                       162 TATCGACAAGCGAACCATCGAGAAGTTCGAGAAGGTTGGT 201
51.LT548416.1_F.culmorumpartialtef1a                                 158 TATCGACAAGCGAACCATCGAGAAGTTCGAGAAGGTTGGT 197
52.MAFF150124_F.asiaticum__Wheat                                     162 TATCGACAAGCGAACCATCGAGAAGTTCGAGAAGGTTGGT 201
53.MAFF238806_F.begoniae_Oncidiumsp                                  199 TATCGACAAGCGAACCATCGAGAAGTTCGAGAAGGTTAGT 238
54.MW594399.1_FusariumincarnatumisolateUD01C                         190 TATCGACAAGCGAACCATCGAGAAGTTCGAGAAGGTTGGT 229
55.OP414923.1Pucciniagraminisf.sp.triticiisolateSHZPgt19             285 ---------------------------------------- 285
56.MT027094.1_BipolarisoryzaestrainOrL-2                               0 ---------------------------------------- 0
57.ON734360.1_AlternariaalternataisolateH126                           0 ---------------------------------------- 0
58.LC333578.1_StemphyliumlycopersiciSOasp2                             0 ---------------------------------------- 0
59.HQ718583.1_Colletotrichumgloeosporioidesisolateq-1                567 ---------------------------------------- 567
60.JN241603.1_AtheliarolfsiiisolateSR1                                 0 ---------------------------------------- 0
61.KJ866474.1_RhizoctoniasolanistrainMHL-1                             0 ---------------------------------------- 0
62.JQ672424.1AlternariatriticinaisolateEGS17-061                       0 ---------------------------------------- 0
63.LT707559.1_P.capsicipartialtef1agene                                0 ---------------------------------------- 0
64.MW090051.1_CurvularialunatastrainCls-3                              0 ---------------------------------------- 0
65.DQ400892.1_Aspergillusterreus                                       0 ---------------------------------------- 0
66.DQ911416.1_Pythiumsp.quercumstrainPy292                             0 ---------------------------------------- 0
67.EU797495.1_Phytophthorasp.oaksoilPoland                             0 ---------------------------------------- 0
68.HM148321.1_Cladosporiumcucumerinum                                  0 ---------------------------------------- 0
```

```
69.AF398888.1_SclerotiniasclerotiorumisolateSS1                    0 ---------------------------------------- 0
70.AF398888.1_S.sclerotiorumisolateSS1                             0 ---------------------------------------- 0
71.HPAB545908.1_Verticilliumnonalfalfaeisolate                     0 ---------------------------------------- 0
72.EF433315.1_CeratocystisfimbriatavoucherCMW15052               522 ---------------------------------------- 522
73.MN159912.1_Botrytiscinerea                                      0 ---------------------------------------- 0
74.MF034741.1_PeltasterfructicolaisolateSRB92                      0 ---------------------------------------- 0
75.LC440360.1_CercosporaasparagiCOasp2                             0 ---------------------------------------- 0
76.AY944105.1_MagnaportheoryzaeisolateSAG00T3()                    0 ---------------------------------------- 0
77.JX266586.1_CochliobolusmiyabeanusvoucherMFLUCC10-0733           0 ---------------------------------------- 0
78.MN393253.1_CorynesporacassiicolaisolateQHD001(MN393253.1UNVERIFIED) 0 ---------------------------------------- 0
79.MF375218.1_AgroatheliarolfsiiisolateBJB24                       0 ---------------------------------------- 0
80.MN106270.1_AgroatheliarolfsiistrainJ-12                         0 ---------------------------------------- 0
81.OQ732628.1_AgroatheliarolfsiiisolateBTCBSr3                     0 ---------------------------------------- 0
82.KY196185.1_ColletotrichumtruncatumstrainPAK53                 468 ---------------------------------------- 468
83.GU935835.1_ColletotrichumcoccodesisolateC96002                132 TATCGACAAGCGTACCATCGAGAAGTTCGAGAAGGAGGGT 171
84.MK085963.1_AlternariatenuissimaisolateSCCZ06                    0 ---------------------------------------- 0
85.MT548042.1_AlternarialongipesstrainKY_2019_012                  0 ---------------------------------------- 0
86.MN356465.1CalonectriamontanaisolateHSP4                         0 ---------------------------------------- 0
87.OL694224.1_CalonectriacanadianastrainF099                       0 ---------------------------------------- 0
88.MK803351.1_NeoscytalidiumdimidiatumstrainKale4-C                0 ---------------------------------------- 0
89.ON376993.1_CurvulariachiangmaiensisisolateND00J7                0 ---------------------------------------- 0
90.OQ383346.1_NeoscytalidiumdimidiatumisolateGKH-2                 0 ---------------------------------------- 0
91.MF662595.1_NeoscytalidiumnovaehollandiaeisolateNeNo1            0 ---------------------------------------- 0
92.EF560588.1Melampsoralini                                      380 ---------------------------------------- 380
93.LC590862.1_NeoscytalidiumdimidiatumPSU-HP01TEF1                 0 ---------------------------------------- 0
94.KX278106.1_BotryosphaeriaqingyuanensisstrainCERC2947            0 ---------------------------------------- 0
95.AJ578763.1_Blumeriagraminisf.sp.hordeicyp51                     0 ---------------------------------------- 0
96.MF490858.1_CurvulariadactylocteniicolastrainCPC28810            0 ---------------------------------------- 0
97.KT287115.1_Bipolariscactivoraisolate3.8.6                       0 ---------------------------------------- 0
98.MT560940.1_CurvulariacactivorastrainHLGH0118                    0 ---------------------------------------- 0
99.OM714565.1_CurvulariaplantarumstrainM0134                       0 ---------------------------------------- 0
100.MN159911.1_BotrytiscinereaSICAUCC19-0003                       0 ---------------------------------------- 0
102.GU294713.1LasiodiplodiatheobromaestrainUCD2430TX               0 ---------------------------------------- 0
103.KX868094.1_Mycosphaerellasp.isolateCRM20.1                     0 ---------------------------------------- 0
104.LC599478.1Pseudocercosporapini-densifloraeMUCC534              0 ---------------------------------------- 0
105.N584698.1BipolarissetariaestrainKBS4-2                         0 ---------------------------------------- 0

2.OM160859.1_F.buharicum                                         195 TA--TTTCCCTTCGATCGCGC--CCTTATGCC-CATCGAT 229
1.LC727524.1_F.buharicum_OKI-1_Okura                             226 TA--TTTCCCTTCGATCGCGC--CCTTATGCC-CATCGAT 260
3.KX302919.1_F.sublunatum                                        192 TA--TTTCCCTCCGATCA-GCGCCCTTTTGCC-CTTCGAT 227
4.LT996094.1_F.convolutans                                       192 TA--TTTCCCTTCGATC--GCGCCCTTTTGCC-CATCGAT 226
5.OM160861.1_F.abutilonis                                        194 TA--TTTCCCTTCGATCGCGC--CC-------------AT 216
6.OM160874.1_F.guadeloupense                                     193 CA--TTTTCCTTCGATCGCGC--CCTTCTGTC-CATCGAT 227
7.MH392475.1_F.graminearum                                       147 CTC-ATTTTCCTCGATCGCGCGCCCTTTTCCC-TTTCGAA 184
8.MH582420.1_F.solani                                            197 GACATCTGCCCCCGATCGCGCCTTGATATTCCACATCGAA 236
9.MAFF244605_F.oxysporum                                         193 CAC-TTTCCCTTCGATCGCGCGTCCTCT-GCC-CATCGAT 229
```

10.MAFF237278_F.contaminatum_Hylocereus	240	CAC-TTTCCCTTCGATCGCGCGTCCTTT-GCC-CATCGAT	276
11.MAFF237649_F.concentricum__Riceroot	233	C-----TCCCTTCGATCGCGCGTCCTTT-GCC-CATCGAT	265
12.MAFF237650_F.concentricum__Wheat	233	C-----TCCCTTCGATCGCGCGTCCTTT-GCC-CATCGAT	265
13.MAFF239869_F.mangiferae__Ryukyupine	223	C-----TCCCTTCGATCGCGCGTCCTTT-GCC-CGTCGAT	255
14.MAFF240460_F.fujikuroi_Passionfruit	223	CAC-TTTCCCTTCGATCGCGCGTCCTTT-GCC-CACCGAT	259
15.MAFF241317_F.graminearum_Wheat	202	CTC-ATTTTCCTCGATCGCGCGCCCTTT-TCCCTTTCGAA	239
16.MAFF242670_F.ipomoeae_Wheat	225	TTCCATTTTCCTCGATCGCACGCCCTCT-GCC-CATCGAT	262
17.MAFF245129_F.concentricum_Fraxinus	223	C-----TCCCTTCGATCGCGCGTCCTTT-GCC-CATCGAT	255
18.MAFF245395_F.cugenangense_Rhubarb	223	CAC-TTTCCCTTCAATCGCGCGTCCTTT-GCC-CATCGAT	259
19.MAFF246637_F.nirenbergiae_Strawberry	223	CAC-TTTCCCTTCGATCGCGCGTCCTTT-GCC-CATCGAT	259
20.MAFF246672_F.nirenbergiae_ChinesePeony	223	CAC-TTTCCCTTCGATCGCGCGTCCTTT-GCC-CATCGAT	259
21.MAFF246697_F.commune_Urallicoriceroot	224	CAC-TTTCCCTTCGATCGCGCGTCCTCT-GCC-CATCGAT	260
22.MAFF246729_F.falciforme_Angelica	228	GACATCT-CCCCCGATCGCGCCTTGCTATTCCACAACGAA	266
23.MAFF247220_F.duplospermum__Euwallaceasp	228	GACATCC-CCCCCGATCGCGCCTTGATATCCCACATCGAA	266
24.MAFF410760_F.odoratissimum_alpha	223	CAC-TTTCCCTTCGATCGCGCGTCCTTT-GCC-CATCGAC	259
25.MAFF244605_FusariumoxysporumSchlechtendal_MAFF244605_Tomato	224	CAC-TTTCCCTTCGATCGCGCGTCCTCT-GCC-CATCGAT	260
26.MAFF241326_F.asiaticum_Wheat	205	CTC-ATTTTCCTCGATCGCGCGCCCTTT-TCC-TTTCGAA	241
27.MAFF245014_F.asiaticum_Wildsoybean	202	CTC-ATTTTCCTCGATCGCGCGCCCTTT-TCC-TTTCGAA	238
28.MAFF150124_F.asiaticum__Wheat	202	CTC-ATTTTCCTCGATCGCGCGCCCTTT-TCC-TTTCGAA	238
29.OM135603.1F.algeriense	230	CAC-TATCCCTTCGCTCCCACGTCCTTT-GCC-CACGGAA	266
30.MAFF237465_F.penzigii_Aloe	233	CATCTTTTCCTCGATTCCTGAGCCTTTCACTACGCGATCG	272
31.MAFF103054_F.oxysporumSchlechtendalf.sp.cucumerinum_Cucumber	223	CAC-TTTCCCTTCAATCGCGCGTCCTTT-GCC-CATCGAT	259
32.MAFF712246_F.oxysporumSchlechtendalf.sp.dianthi__Carnation	223	CAC-TTTCCCTTCGATCGCGCGTCCTTT-GCC-CATCGAT	259
33.MAFF305558_F.oxysporumSchlechtendalf.sp.fragariae__Watermelon	223	CAC-TTTCCCTTCAATCGCGCGTCCTTT-GCC-CATCGAT	259
34.MAFF744087_F.oxysporumSchlechtendalf.sp.lactucae__Lettuce	223	CAC-TTTCCCTTCAATCGCGCGTCCTTT-GCC-CATCGAT	259
35.MAFF726924_F.oxysporumSchlechtendalf.sp.lagenariae_Whitefloweredgourd	223	CAC-TTTCCCTTCAATCGCGCGTCCTTT-GCC-CATCGAT	259
36.MAFF744003_F.oxysporumSchlechtendalf.sp.lagenariae_Squash)	223	CAC-TTTCCCTTCAATCGCGCGTCCTTT-GCC-CATCGAT	259
37.MAFF305122_F.oxysporumSchlechtendalf.sp.melonis__Melon	223	CAC-TTTCCCTTCAATCGCGCGTCCTTT-GCC-CATCGAT	259
38.MAFF306714_F.oxysporumSchlechtendalf.sp.momordicae_Balsampear	223	CAC-TTTCCCTTCAATCGCGCGTCCTTT-GCC-CATCGAT	259
39.MAFF238905_F.oxysporumSchlechtendalf.sp.radicis-lycopersici_Tomato	223	CAC-TTTCCCTTCGATCGCGCGTCCTTT-GCC-CATCGAT	259
40.MAFF150004_F.oxysporumSchlechtendalf.sp.spinaciae_Spinach	223	CAC-TTTCCCTTCAATCGCGCGTCCTTT-GCC-CATCGAT	259
41.MAFF247034_F.oxysporumSchlechtendal__Goldenchain	223	CAC-TTTCCCTTCAATCGCGCGTCCTTT-GCC-CATCGAT	259
42.MAFF245747_F.oxysporumSchlechtendalf.sp.callistephi__Chinaaster	223	CAC-TTTCCCTTCAATCGCGCGTCCTTT-GCC-CATCGAT	259
43.MAFF305115_FoxysporumSchlechtendalf.sp.batatas__Sweatpotato	228	GACATCT-CCCCCGATCGCGCCTTGCTATTCCACATCGAA	266
44.MAFF150126_F.asiaticum_Seed	202	CTC-ATTTTCCTCGATCGCGCGCCCTTT-TCC-TTTCGAA	238
45.MAFF246738_F.solani_Angelica	228	GACATCT-CCCCCGATCGCGCCTTGCTATTCCACATCGAA	266
46.MAFF246664_F.cugenangense_Perilla	223	CAC-TTTCCCTTCAATCGCGCGTCCTTT-GCC-CATCGAT	259
47.MH582420.1F.solanistrainMRC256	217	GACATCTGCCCCCGATCGCGCCTTGATATTCCACATCGAA	256
48.MAFF240361_F.babinda_Soil	225	CAC-TTGCCCTTCGATCGCGCGCCCTTTTGCC-CGTCGAG	262
49.MAFF242368_F.azukicola_Azukibean	229	GACATCT-CCCCCGAGCGCGCCTTGCTATTCTCCATCGAA	267
50.MAFF241312_F.asiaticum_Soil,welshonionfield	202	CTC-ATTTTCCTCGATCGCGCGCCCTTT-TCC-TTTCGAA	238
51.LT548416.1_F.culmorumpartialtef1a	198	CTC-ATTTTCCTCGATCGCGCGCCCTTT-TCCCTTTCGAA	235
52.MAFF150124_F.asiaticum__Wheat	202	CTC-ATTTTCCTCGATCGCGCGCCCTTT-TCC-TTTCGAA	238
53.MAFF238806_F.begoniae_Oncidiumsp	239	TAC-TTTCCCTTCGATCGCGCGTCCTTT-GCC-CATCGAT	275
54.MW594399.1_FusariumincarnatumisolateUD01C	230	TTC-CATTTCCCCGATCGCACGCCCTCT-ACC-CACCGAT	266
55.OP414923.1Pucciniagraminisf.sp.triticiisolateSHZPgt19	285	--	285

```
56.MT027094.1_BipolarisoryzaestrainOrL-2                                0 ---------------------------------------- 0
57.ON734360.1_AlternariaalternataisolateH126                             0 ---------------------------------------- 0
58.LC333578.1_StemphyliumlycopersiciSOasp2                               0 ---------------------------------------- 0
59.HQ718583.1_Colletotrichumgloeosporioidesisolateq-1                  567 ---------------------------------------- 567
60.JN241603.1_AtheliarolfsiiisolateSR1                                   0 ---------------------------------------- 0
61.KJ866474.1_RhizoctoniasolanistrainMHL-1                               0 ---------------------------------------- 0
62.JQ672424.1AlternariatriticinaisolateEGS17-061                         0 ---------------------------------------- 0
63.LT707559.1_P.capsicipartialtef1agene                                  0 ---------------------------------------- 0
64.MW090051.1_CurvularialunatastrainCls-3                                0 ---------------------------------------- 0
65.DQ400892.1_Aspergillusterreus                                         0 ---------------------------------------- 0
66.DQ911416.1_Pythiumsp.quercumstrainPy292                               0 ---------------------------------------- 0
67.EU797495.1_Phytophthorasp.oaksoilPoland                               0 ---------------------------------------- 0
68.HM148321.1_Cladosporiumcucumerinum                                    0 ---------------------------------------- 0
69.AF398888.1_SclerotiniasclerotiorumisolateSS1                          0 ---------------------------------------- 0
70.AF398888.1_S.sclerotiorumisolateSS1                                   0 ---------------------------------------- 0
71.HPAB545908.1_Verticilliumnonalfalfaeisolate                           0 ---------------------------------------- 0
72.EF433315.1_CeratocystisfimbriatavoucherCMW15052                     522 ---------------------------------------- 522
73.MN159912.1_Botrytiscinerea                                            0 ---------------------------------------- 0
74.MF034741.1_PeltasterfructicolaisolateSRB92                            0 ---------------------------------------- 0
75.LC440360.1_CercosporaasparagiCOasp2                                   0 ---------------------------------------- 0
76.AY944105.1_MagnaportheoryzaeisolateSAG00T3()                          1 -------------------------------TAAGCACTA 9
77.JX266586.1_CochliobolusmiyabeanusvoucherMFLUCC10-0733                 0 ---------------------------------------- 0
78.MN393253.1_CorynesporacassiicolaisolateQHD001(MN393253.1UNVERIFIED)   0 ---------------------------------------- 0
79.MF375218.1_AgroatheliarolfsiiisolateBJB24                             0 ---------------------------------------- 0
80.MN106270.1_AgroatheliarolfsiistrainJ-12                               0 ---------------------------------------- 0
81.OQ732628.1_AgroatheliarolfsiiisolateBTCBSr3                           0 ---------------------------------------- 0
82.KY196185.1_ColletotrichumtruncatumstrainPAK53                       468 ---------------------------------------- 468
83.GU935835.1_ColletotrichumcoccodesisolateC96002                      172 ACGTACATTACATTTTTCACGCAGGAGCGATCGGGTCTGG 211
84.MK085963.1_AlternariatenuissimaisolateSCCZ06                          0 ---------------------------------------- 0
85.MT548042.1_AlternarialongipesstrainKY_2019_012                        0 ---------------------------------------- 0
86.MN356465.1CalonectriamontanaisolateHSP4                               1 ---GAGAAGGTTGGTGACATTTCCTCGATTCCCCCATCGC 37
87.OL694224.1_CalonectriacanadianastrainF099                             1 -TCGAGAAGGTTGGTGACATTTCCTCGATTCCCCCATCGC 39
88.MK803351.1_NeoscytalidiumdimidiatumstrainKale4-C                      0 ---------------------------------------- 0
89.ON376993.1_CurvulariachiangmaiensisisolateND00J7                      0 ---------------------------------------- 0
90.OQ383346.1_NeoscytalidiumdimidiatumisolateGKH-2                       0 ---------------------------------------- 0
91.MF662595.1_NeoscytalidiumnovaehollandiaeisolateNeNo1                  0 ---------------------------------------- 0
92.EF560588.1Melampsoralini                                            380 ---------------------------------------- 380
93.LC590862.1_NeoscytalidiumdimidiatumPSU-HP01TEF1                       0 ---------------------------------------- 0
94.KX278106.1_BotryosphaeriaqingyuanensisstrainCERC2947                  0 ---------------------------------------- 0
95.AJ578763.1_Blumeriagraminisf.sp.hordeicyp51                           0 ---------------------------------------- 0
96.MF490858.1_CurvulariadactylocteniicolastrainCPC28810                  0 ---------------------------------------- 0
97.KT287115.1_Bipolariscactivoraisolate3.8.6                             0 ---------------------------------------- 0
98.MT560940.1_CurvulariacactivorastrainHLGH0118                          0 ---------------------------------------- 0
99.OM714565.1_CurvulariaplantarumstrainM0134                             0 ---------------------------------------- 0
100.MN159911.1_BotrytiscinereaSICAUCC19-0003                             0 ---------------------------------------- 0
102.GU294713.1LasiodiplodiatheobromaestrainUCD2430TX                     0 ---------------------------------------- 0
```

```
103.KX868094.1_Mycosphaerellasp.isolateCRM20.1                        1 -----------------------------CAGTCACACCC 11
104.LC599478.1Pseudocercosporapini-densifloraeMUCC534                 0 ---------------------------------------- 0
105.N584698.1BipolarissetariaestrainKBS4-2                            0 ---------------------------------------- 0

2.OM160859.1_F.buharicum                                            230 TTGCCCGTCGAATCGCTCCC--TCCGCGACTCGCAACGCG 267
1.LC727524.1_F.buharicum_OKI-1_Okura                                261 TTGCCCGTCGAATCGCTCCC--TCCGCGACTCGCAACGCG 298
3.KX302919.1_F.sublunatum                                           228 TTGCCCGTCGAATCGCTCCC--TTCACGATTTCCAACACG 265
4.LT996094.1_F.convolutans                                          227 TTGCCCGTCGAATCGCTCCC--TTCACGATATGCAACACG 264
5.OM160861.1_F.abutilonis                                           217 TTGTTCATCGAATCGTTCCC--TTCACGACTCGCAACGCG 254
6.OM160874.1_F.guadeloupense                                        228 TTGCCCGTCGAATCGCTCCC--TTCACGACTCGCAACACG 265
7.MH392475.1_F.graminearum                                          185 ATATCATTCGAATCGCCCTCACACGACGACTCGATACGCG 224
8.MH582420.1_F.solani                                               237 TTCCCCGTCGAATTCCCTCC--ATCGCGATACGCTCTGCG 274
9.MAFF244605_F.oxysporum                                            230 TT-CC--------------C---CTACGACTCGAAACCTG 251
10.MAFF237278_F.contaminatum_Hylocereus                             277 TT-CC--------------C---CTACGACTCGAAACGTA 298
11.MAFF237649_F.concentricum__Riceroot                              266 TTTCC--------------C---CTACGACTCGAAACGTG 288
12.MAFF237650_F.concentricum__Wheat                                 266 TTTCC--------------C---CTACGACTCGAAACGTG 288
13.MAFF239869_F.mangiferae__Ryukyupine                              256 TTTCC--------------C----TACGATTCGAAACGTG 277
14.MAFF240460_F.fujikuroi_Passionfruit                              260 TT-CC--------------C---TTACGATTCGAAACGTG 281
15.MAFF241317_F.graminearum_Wheat                                   240 ATATCATTCGAATCGCCCTCACACGACGACTCGATACGCG 279
16.MAFF242670_F.ipomoeae_Wheat                                      263 CCATCACCCGAATC--AGTCTC--GACGACTGAACATGCG 298
17.MAFF245129_F.concentricum_Fraxinus                               256 TTTCC--------------C---CTACGACTCGAAACGTG 278
18.MAFF245395_F.cugenangense_Rhubarb                                260 TT-CC--------------C---CTACGACTCGAAACGTG 281
19.MAFF246637_F.nirenbergiae_Strawberry                             260 TT-CC--------------C---CTACGACTCGAAACGTG 281
20.MAFF246672_F.nirenbergiae_ChinesePeony                           260 TT-CC--------------C---CTACGACTCGAAACGTG 281
21.MAFF246697_F.commune_Urallicoriceroot                            261 TT-CC--------------C---CTACGACTCGAAACCTG 282
22.MAFF246729_F.falciforme_Angelica                                 267 TTCCC------------TCC--CTCGCGATACGCTCTGCG 292
23.MAFF247220_F.duplospermum__Euwallaceasp                          267 TTCCC------------TCC--CTCACGATCCGCGCTGCG 292
24.MAFF410760_F.odoratissimum_alpha                                 260 TT-CC--------------C---CTACGACTCGAAACGTG 281
25.MAFF244605_FusariumoxysporumSchlechtendal_MAFF244605_Tomato      261 TT-CC--------------C---CTACGACTCGAAACCTG 282
26.MAFF241326_F.asiaticum_Wheat                                     242 ATATCATTCGAATCGCACTCACACGACGACTCGATACGCG 281
27.MAFF245014_F.asiaticum_Wildsoybean                               239 ATATCATTCGAATCGCACTCACACGACGACTCGATACGCG 278
28.MAFF150124_F.asiaticum__Wheat                                    239 ATATCATTCGAATCGCACTCACACGACGACTCGATACGCG 278
29.OM135603.1F.algeriense                                           267 TTTCCTCTCGACTCGCTCCC---ATACGACTCGAAACGCG 303
30.MAFF237465_F.penzigii_Aloe                                       273 ATTCGCATCACGTCCATTACCCCGCCCAATACCGATGATA 312
31.MAFF103054_F.oxysporumSchlechtendalf.sp.cucumerinum_Cucumber     260 TT-CC--------------C---CTACGACTCGAAACGTG 281
32.MAFF712246_F.oxysporumSchlechtendalf.sp.dianthi__Carnation       260 TT-CC--------------C---CTACGACTCGAAACGTG 281
33.MAFF305558_F.oxysporumSchlechtendalf.sp.fragariae__Watermelon    260 TT-CC--------------C---CTACGACTCGAAACGTG 281
34.MAFF744087_F.oxysporumSchlechtendalf.sp.lactucae__Lettuce        260 TT-CC--------------C---CTACGACTCGAAACGTG 281
35.MAFF726924_F.oxysporumSchlechtendalf.sp.lagenariae_Whitefloweredgourd 260 TT-CC--------------C---CTACGACTCGAAACGTG 281
36.MAFF744003_F.oxysporumSchlechtendalf.sp.lagenariae_Squash)       260 TT-CC--------------C---CTACGACTCGAAACGTG 281
37.MAFF305122_F.oxysporumSchlechtendalf.sp.melonis__Melon           260 TT-CC--------------C---CTACGACTCGAAACGTG 281
38.MAFF306714_F.oxysporumSchlechtendalf.sp.momordicae_Balsampear    260 TT-CC--------------C---CTACGACTCGAAACGTG 281
39.MAFF238905_F.oxysporumSchlechtendalf.sp.radicis-lycopersici_Tomato 260 TT-CA--------------C---CTACGACTCGAAACGTG 281
40.MAFF150004_F.oxysporumSchlechtendalf.sp.spinaciae_Spinach        260 TT-CC--------------C---CTACGACTCGAAACGTG 281
41.MAFF247034_F.oxysporumSchlechtendal__Goldenchain                 260 TT-CC--------------C---CTACGACTCGAAACGTG 281
42.MAFF245747_F.oxysporumSchlechtendalf.sp.callistephi__Chinaaster  260 TT-CC--------------C---CTACGACTCGAAACGTG 281
```

```
43.MAFF305115_FoxysporumSchlechtendalf.sp.batatas__Sweatpotato        267 TTCCCCGTCGAATTCCCTCC--CTCGCGATACGCTCTGCG 304
44.MAFF150126_F.asiaticum_Seed                                        239 ATATCATTCGAATCGCACTCACACGACGACTCGATACGCG 278
45.MAFF246738_F.solani_Angelica                                       267 TTCCCCGTCGAATTCCCTCC--CTCGCGATACGCTCTGCG 304
46.MAFF246664_F.cugenangense_Perilla                                  260 TT-CC--------------C---CTACGACTCGAAACGTG 281
47.MH582420.1F.solanistrainMRC256                                     257 TTCCCCGTCGAATTCCCTCC--ATCGCGATACGCTCTGCG 294
48.MAFF240361_F.babinda_Soil                                          263 TTCCCTTTCGAATCACTCCC---ATACGACTCGATCAGCG 299
49.MAFF242368_F.azukicola_Azukibean                                   268 TTCCCCGTCGAATTCCCTCC--CTCGCGATCCGCTCTGCG 305
50.MAFF241312_F.asiaticum_Soil,welshonionfield                        239 ATATCATTCGAATCGCACTCACACGACGACTCGATACGCG 278
51.LT548416.1_F.culmorumpartialtef1a                                  236 ACATCATTCGAATCGCCCTCACACGACGACTCGATACGCG 275
52.MAFF150124_F.asiaticum__Wheat                                      239 ATATCATTCGAATCGCACTCACACGACGACTCGATACGCG 278
53.MAFF238806_F.begoniae_Oncidiumsp                                   276 TC-TC--------------C---ATACGACTCGAAACGTG 297
54.MW594399.1_FusariumincarnatumisolateUD01C                          267 CCATC-AGTCGAATCAGTTA---CGACGATTGAATATGCG 302
55.OP414923.1Pucciniagraminisf.sp.triticiisolateSHZPgt19              285 ---------------------------------------- 285
56.MT027094.1_BipolarisoryzaestrainOrL-2                                0 ---------------------------------------- 0
57.ON734360.1_AlternariaalternataisolateH126                            0 ---------------------------------------- 0
58.LC333578.1_StemphyliumlycopersiciSOasp2                              0 ---------------------------------------- 0
59.HQ718583.1_Colletotrichumgloeosporioidesisolateq-1                 567 ---------------------------------------- 567
60.JN241603.1_AtheliarolfsiiisolateSR1                                  0 ---------------------------------------- 0
61.KJ866474.1_RhizoctoniasolanistrainMHL-1                              0 ---------------------------------------- 0
62.JQ672424.1AlternariatriticinaisolateEGS17-061                        0 ---------------------------------------- 0
63.LT707559.1_P.capsicipartialtef1agene                                 0 ---------------------------------------- 0
64.MW090051.1_CurvularialunatastrainCls-3                               0 ---------------------------------------- 0
65.DQ400892.1_Aspergillusterreus                                        0 ---------------------------------------- 0
66.DQ911416.1_Pythiumsp.quercumstrainPy292                              0 ---------------------------------------- 0
67.EU797495.1_Phytophthorasp.oaksoilPoland                              0 ---------------------------------------- 0
68.HM148321.1_Cladosporiumcucumerinum                                   1 ---------------------CATCGAGAAGTTCGAGAAG 19
69.AF398888.1_SclerotiniasclerotiorumisolateSS1                         0 ---------------------------------------- 0
70.AF398888.1_S.sclerotiorumisolateSS1                                  0 ---------------------------------------- 0
71.HPAB545908.1_Verticilliumnonalfalfaeisolate                          0 ---------------------------------------- 0
72.EF433315.1_CeratocystisfimbriatavoucherCMW15052                    522 ---------------------------------------- 522
73.MN159912.1_Botrytiscinerea                                           0 ---------------------------------------- 0
74.MF034741.1_PeltasterfructicolaisolateSRB92                           0 ---------------------------------------- 0
75.LC440360.1_CercosporaasparagiCOasp2                                  0 ---------------------------------------- 0
76.AY944105.1_MagnaportheoryzaeisolateSAG00T3()                        10 TCCTTTTCCCTCTATTCAATCGCTCTTTACAAATGCCCAA 49
77.JX266586.1_CochliobolusmiyabeanusvoucherMFLUCC10-0733                0 ---------------------------------------- 0
78.MN393253.1_CorynesporacassiicolaisolateQHD001(MN393253.1UNVERIFIED)  0 ---------------------------------------- 0
79.MF375218.1_AgroatheliarolfsiiisolateBJB24                            0 ---------------------------------------- 0
80.MN106270.1_AgroatheliarolfsiistrainJ-12                              0 ---------------------------------------- 0
81.OQ732628.1_AgroatheliarolfsiiisolateBTCBSr3                          0 ---------------------------------------- 0
82.KY196185.1_ColletotrichumtruncatumstrainPAK53                      468 ---------------------------------------- 468
83.GU935835.1_ColletotrichumcoccodesisolateC96002                     212 GCAGTCGAAATGCCTGCGAGGGCAATTAGACTCATCCCAC 251
84.MK085963.1_AlternariatenuissimaisolateSCCZ06                         0 ---------------------------------------- 0
85.MT548042.1_AlternarialongipesstrainKY_2019_012                       0 ---------------------------------------- 0
86.MN356465.1CalonectriamontanaisolateHSP4                             38 CCGTCGATTCGCGCGTCGCCGTGTCTGCTCCACCCGAAAC 77
87.OL694224.1_CalonectriacanadianastrainF099                           40 CCGTCGATTCGCGCGTCGCCGTGTCTGCTCCACCCGAAAC 79
88.MK803351.1_NeoscytalidiumdimidiatumstrainKale4-C                     0 ---------------------------------------- 0
```

```
89.ON376993.1_CurvulariachiangmaiensisisolateND00J7          0 ---------------------------------------- 0
90.OQ383346.1_NeoscytalidiumdimidiatumisolateGKH-2           0 ---------------------------------------- 0
91.MF662595.1_NeoscytalidiumnovaehollandiaeisolateNeNo1      0 ---------------------------------------- 0
92.EF560588.1Melampsoralini                                380 ---------------------------------------- 380
93.LC590862.1_NeoscytalidiumdimidiatumPSU-HP01TEF1           0 ---------------------------------------- 0
94.KX278106.1_BotryosphaeriaqingyuanensisstrainCERC2947      0 ---------------------------------------- 0
95.AJ578763.1_Blumeriagraminisf.sp.hordeicyp51               0 ---------------------------------------- 0
96.MF490858.1_CurvulariadactylocteniicolastrainCPC28810      0 ---------------------------------------- 0
97.KT287115.1_Bipolariscactivoraisolate3.8.6                 0 ---------------------------------------- 0
98.MT560940.1_CurvulariacactivorastrainHLGH0118              0 ---------------------------------------- 0
99.OM714565.1_CurvulariaplantarumstrainM0134                 0 ---------------------------------------- 0
100.MN159911.1_BotrytiscinereaSICAUCC19-0003                 0 ---------------------------------------- 0
102.GU294713.1LasiodiplodiatheobromaestrainUCD2430TX         0 ---------------------------------------- 0
103.KX868094.1_Mycosphaerellasp.isolateCRM20.1              12 AACAACACCATTCGCACACATTTTCGCCGCCTTATCACAT 51
104.LC599478.1Pseudocercosporapini-densifloraeMUCC534        0 ---------------------------------------- 0
105.N584698.1BipolarissetariaestrainKBS4-2                   0 ---------------------------------------- 0

2.OM160859.1_F.buharicum                                   268 CC-GTTACCCCGCTCGAGCACAAAAATTTT-GCGGTGCGA 305
1.LC727524.1_F.buharicum_OKI-1_Okura                       299 CC-GTTACCCCGCTCGAGCACAAAAATTTT-GCGGTGCGA 336
3.KX302919.1_F.sublunatum                                  266 CC-GTTACCCCGCTCGAGCACAAAATTTTG--CGGCGCGA 302
4.LT996094.1_F.convolutans                                 265 CC-GTTACCCCGCTCGAGCACAAAAATTTG--CGGTGCGA 301
5.OM160861.1_F.abutilonis                                  255 CC-GTTACCCCGCTCGAGCACAAAATTTTT-GCGGTGCGA 292
6.OM160874.1_F.guadeloupense                               266 CC-TTTACCCCGCTCGAGCTCAAAAATTTT-GCGGTGCGA 303
7.MH392475.1_F.graminearum                                 225 CCTGTTACCCCGCTCGAGGTCAAAAATTTT-GCGGCTTTG 263
8.MH582420.1_F.solani                                      275 CCCGCTTC---TC-CGAGTCCCAAAATTTTTGCGGTCCGA 310
9.MAFF244605_F.oxysporum                                   252 CCCGCTACCCCGCTCGAGACCAAAAATTTT-GCGATATGA 290
10.MAFF237278_F.contaminatum_Hylocereus                    299 CCCGCTACCCCGCTCGAGACCAAAAATTTT-GCAATATGA 337
11.MAFF237649_F.concentricum__Riceroot                     289 CCCGCTACCCCGCTCGAGACCAAAAATTTT-GCGATATGA 327
12.MAFF237650_F.concentricum__Wheat                        289 CCCGCTACCCCGCTCGAGACCAAAAATTTT-GCGATATGA 327
13.MAFF239869_F.mangiferae__Ryukyupine                     278 CCCGCTACCCCGCTCGAGACCAAAAATTTT-GCGATATGA 316
14.MAFF240460_F.fujikuroi_Passionfruit                     282 CCTGCTACCCCGCTCGAGACCAAAAATTTT-GCGATATGA 320
15.MAFF241317_F.graminearum_Wheat                          280 CCTGTTACCCCGCTCGAGGTCAAAAATTTT-GCGGCTTTG 318
16.MAFF242670_F.ipomoeae_Wheat                             299 CCTGTTACCCCGCTCGAGTACAAAA-TTTT-GCGGTTCAA 336
17.MAFF245129_F.concentricum_Fraxinus                      279 CCCGCTACCCCGCTCGAGACCAAAAATTTT-GCGATATGA 317
18.MAFF245395_F.cugenangense_Rhubarb                       282 CCCGCTACCCCGCTCGAGACCAAAAATTTT-GCAATATGA 320
19.MAFF246637_F.nirenbergiae_Strawberry                    282 CCCGCTACCCCGCTCGAGACCAAAAATTTT-GCAATATGA 320
20.MAFF246672_F.nirenbergiae_ChinesePeony                  282 CCCGCTACCCCGCTCGAGACCAAAAATTTT-GCAATATGA 320
21.MAFF246697_F.commune_Urallicoriceroot                   283 CCCGCTACCCCGCTCGAGACCAAAAATTTT-GCGATATGA 321
22.MAFF246729_F.falciforme_Angelica                        293 CCCGCTTC--TCC-CGAGTCCCAAATTTTTTGCGGTCCGA 329
23.MAFF247220_F.duplospermum__Euwallaceasp                 293 CCCGCTTC--TTC-CGAGTCCCAAAATTTTTGCGGTGCGA 329
24.MAFF410760_F.odoratissimum_alpha                        282 CCCGCTACCCCGCTCGAGACCAAAAATTTT-GCAATATGA 320
25.MAFF244605_FusariumoxysporumSchlechtendal_MAFF244605_Tomato 283 CCCGCTACCCCGCTCGAGACCAAAAATTTT-GCGATATGA 321
26.MAFF241326_F.asiaticum_Wheat                            282 CCTGTTACCCCGCTCGAGGTCAAAAATTTT-GCGGCTTTG 320
27.MAFF245014_F.asiaticum_Wildsoybean                      279 CCTGTTACCCCGCTCGAGGTCAAAAATTTT-GCGGCTTTG 317
28.MAFF150124_F.asiaticum__Wheat                           279 CCTGTTACCCCGCTCGAGGTCAAAAATTTT-GCGGCTTTG 317
29.OM135603.1F.algeriense                                  304 CCCGCTACCCCGCTCGAGAACAAAAATTTT-GCGGTGCGA 342
```

```
30.MAFF237465_F.penzigii_Aloe                                         313 TTTTTTTGCGTGGCCTTTTCATGCTTTGGCGGAGGTTCTT 352
31.MAFF103054_F.oxysporumSchlechtendalf.sp.cucumerinum_Cucumber       282 CCCGCTACCCCGCTCGAGACCAAAAATTTT-GCAATATGA 320
32.MAFF712246_F.oxysporumSchlechtendalf.sp.dianthi__Carnation         282 CCCGCTACCCCGCTCGAGACCAAAAATTTT-GCAATATGA 320
33.MAFF305558_F.oxysporumSchlechtendalf.sp.fragariae__Watermelon      282 CCCGCTACCCCGCTCGAGACCAAAAATTTT-GCAATATGA 320
34.MAFF744087_F.oxysporumSchlechtendalf.sp.lactucae__Lettuce          282 CCCGCTACCCCGCTCGAGACCAAAAATTTT-GCAATATGA 320
35.MAFF726924_F.oxysporumSchlechtendalf.sp.lagenariae_Whitefloweredgourd 282 CCCGCTACCCCGCTCGAGACCAAAAATTTT-GCAATATGA 320
36.MAFF744003_F.oxysporumSchlechtendalf.sp.lagenariae_Squash)         282 CCCGTTACCCCGCTCGAGACCAAAAATTTT-GCAATATGA 320
37.MAFF305122_F.oxysporumSchlechtendalf.sp.melonis__Melon             282 CCCGCTACCCCGCTCGAGACCAAAAATTTT-GCAATATGA 320
38.MAFF306714_F.oxysporumSchlechtendalf.sp.momordicae_Balsampear      282 CCCGCTACCCCGCTCGAGACCAAAAATTTT-GCAATATGA 320
39.MAFF238905_F.oxysporumSchlechtendalf.sp.radicis-lycopersici_Tomato 282 CCCGCTACCCCGCTCGAGACCAAAAATTTT-GCAATATGA 320
40.MAFF150004_F.oxysporumSchlechtendalf.sp.spinaciae_Spinach          282 CCCGCTACCCCGCTCGAGACCAAAAATTTT-GCAATATGA 320
41.MAFF247034_F.oxysporumSchlechtendal__Goldenchain                   282 CCCGCTACCCCGCTCGAGACCAAAAATTTT-GCAATATGA 320
42.MAFF245747_F.oxysporumSchlechtendalf.sp.callistephi__Chinaaster    282 CCCGCTACCCCGCTCGAGACCAAAAATTTT-GCAATATGA 320
43.MAFF305115_FoxysporumSchlechtendalf.sp.batatas__Sweatpotato        305 CCCGCTTC--TCC-CGAGTCCCAAAATTTTTGCGGTCCGA 341
44.MAFF150126_F.asiaticum_Seed                                        279 CCTGTTACCCCGCTCGAGGTCAAAAATTTT-GCGGCTTTG 317
45.MAFF246738_F.solani_Angelica                                       305 CCCGCTTC--TCC-CGAGTCCCAAAATTTTTGCGGTCCGA 341
46.MAFF246664_F.cugenangense_Perilla                                  282 CCCGCTACCCCGCTCGAGACCAAAAATTTT-GCAATATGA 320
47.MH582420.1F.solanistrainMRC256                                     295 CCCGCTTC--TCC--GAGTCCCAAAATTTTTGCGGTCCGA 330
48.MAFF240361_F.babinda_Soil                                          300 CCGGATACCCCGCTTGAGTCCAAAATTTTT-GCGGTGCGA 338
49.MAFF242368_F.azukicola_Azukibean                                   306 CCCGCTCCCCTCC-CGAGTCACAAAATTTT-GCGGCGCGA 343
50.MAFF241312_F.asiaticum_Soil,welshonionfield                        279 CCTGTTACCCCGCTCGAGGTCAAAAATTTT-GCGGCTTTG 317
51.LT548416.1_F.culmorumpartialtef1a                                  276 CCTGTTACCCCGCTCGAGGTCAAAAATTTT-GCGGCTTTG 314
52.MAFF150124_F.asiaticum__Wheat                                      279 CCTGTTACCCCGCTCGAGGTCAAAAATTTT-GCGGCTTTG 317
53.MAFF238806_F.begoniae_Oncidiumsp                                   298 CCCGCTACCCCGCTCGAGACCAAAAATTTT-GCGATATGA 336
54.MW594399.1_FusariumincarnatumisolateUD01C                          303 CCTGTTACCCCGCTCGAGTACAAAA-TTTT-GCGGTTCAA 340
55.OP414923.1Pucciniagraminisf.sp.triticiisolateSHZPgt19              285 ---------------------------------------- 285
56.MT027094.1_BipolarisoryzaestrainOrL-2                                0 ---------------------------------------- 0
57.ON734360.1_AlternariaalternataisolateH126                            0 ---------------------------------------- 0
58.LC333578.1_StemphyliumlycopersiciSOasp2                              0 ---------------------------------------- 0
59.HQ718583.1_Colletotrichumgloeosporioidesisolateq-1                 567 ---------------------------------------- 567
60.JN241603.1_AtheliarolfsiiisolateSR1                                  1 --------------------CAAATATGAGTGCGTGTTCT 20
61.KJ866474.1_RhizoctoniasolanistrainMHL-1                              0 ---------------------------------------- 0
62.JQ672424.1AlternariatriticinaisolateEGS17-061                        0 ---------------------------------------- 0
63.LT707559.1_P.capsicipartialtef1agene                                 0 ---------------------------------------- 0
64.MW090051.1_CurvularialunatastrainCls-3                               0 ---------------------------------------- 0
65.DQ400892.1_Aspergillusterreus                                        0 ---------------------------------------- 0
66.DQ911416.1_Pythiumsp.quercumstrainPy292                              0 ---------------------------------------- 0
67.EU797495.1_Phytophthorasp.oaksoilPoland                              0 ---------------------------------------- 0
68.HM148321.1_Cladosporiumcucumerinum                                  20 GTGAGCATCACTCGGGCACAATGTCGCCTCGTCGCGATGG 59
69.AF398888.1_SclerotiniasclerotiorumisolateSS1                         0 ---------------------------------------- 0
70.AF398888.1_S.sclerotiorumisolateSS1                                  0 ---------------------------------------- 0
71.HPAB545908.1_Verticilliumnonalfalfaeisolate                          0 ---------------------------------------- 0
72.EF433315.1_CeratocystisfimbriatavoucherCMW15052                    522 ---------------------------------------- 522
73.MN159912.1_Botrytiscinerea                                           0 ---------------------------------------- 0
74.MF034741.1_PeltasterfructicolaisolateSRB92                           0 ---------------------------------------- 0
75.LC440360.1_CercosporaasparagiCOasp2                                  0 ---------------------------------------- 0
```

76.AY944105.1_MagnaportheoryzaeisolateSAG00T3()	50	TGAGTACATAAAAACTCGCGTTTTTTTCGCGGCGCATACT	89
77.JX266586.1_CochliobolusmiyabeanusvoucherMFLUCC10-0733	0	--	0
78.MN393253.1_CorynesporacassiicolaisolateQHD001(MN393253.1UNVERIFIED)	0	--	0
79.MF375218.1_AgroatheliarolfsiiisolateBJB24	0	--	0
80.MN106270.1_AgroatheliarolfsiistrainJ-12	0	--	0
81.OQ732628.1_AgroatheliarolfsiiisolateBTCBSr3	0	--	0
82.KY196185.1_ColletotrichumtruncatumstrainPAK53	468	--	468
83.GU935835.1_ColletotrichumcoccodesisolateC96002	252	CCGCGCCATGTTCCCATTCGCGAACCATATTCCATCAGTA	291
84.MK085963.1_AlternariatenuissimaisolateSCCZ06	0	--	0
85.MT548042.1_AlternarialongipesstrainKY_2019_012	0	--	0
86.MN356465.1CalonectriamontanaisolateHSP4	78	ACCTCCCCCTCGCCACCCCTCTTTGTCGATCGAAAAATTT	117
87.OL694224.1_CalonectriacanadianastrainF099	80	ACCTCCCCCTCGCCACCCCTCTTTGTCGATCGAAAAATTT	119
88.MK803351.1_NeoscytalidiumdimidiatumstrainKale4-C	0	--	0
89.ON376993.1_CurvulariachiangmaiensisisolateND00J7	0	--	0
90.OQ383346.1_NeoscytalidiumdimidiatumisolateGKH-2	0	--	0
91.MF662595.1_NeoscytalidiumnovaehollandiaeisolateNeNo1	0	--	0
92.EF560588.1Melampsoralini	380	--	380
93.LC590862.1_NeoscytalidiumdimidiatumPSU-HP01TEF1	0	--	0
94.KX278106.1_BotryosphaeriaqingyuanensisstrainCERC2947	0	--	0
95.AJ578763.1_Blumeriagraminisf.sp.hordeicyp51	0	--	0
96.MF490858.1_CurvulariadactylocteniicolastrainCPC28810	0	--	0
97.KT287115.1_Bipolariscactivoraisolate3.8.6	0	--	0
98.MT560940.1_CurvulariacactivorastrainHLGH0118	0	--	0
99.OM714565.1_CurvulariaplantarumstrainM0134	0	--	0
100.MN159911.1_BotrytiscinereaSICAUCC19-0003	0	--	0
102.GU294713.1LasiodiplodiatheobromaestrainUCD2430TX	0	--	0
103.KX868094.1_Mycosphaerellasp.isolateCRM20.1	52	CTTTTGGTGAGAGGGGCAAAATTTGGTGGGGTGCGAGAAT	91
104.LC599478.1Pseudocercosporapini-densifloraeMUCC534	0	--	0
105.N584698.1BipolarissetariaestrainKBS4-2	0	--	0

2.OM160859.1_F.buharicum	306	CCGT-AATTTTTTT----GGTGGGGCATTTT-ACCCCGCC	339
1.LC727524.1_F.buharicum_OKI-1_Okura	337	CCGT-AATTTTTTT----GGTGGGGCATTTT-ACCCCGCC	370
3.KX302919.1_F.sublunatum	303	CCGT-AATTTTTTT----GGTGGGGCATTT-TACCCCGCC	336
4.LT996094.1_F.convolutans	302	CCGT-AATTTTTTT----GGTGGGGCATAT-TACCCCGCC	335
5.OM160861.1_F.abutilonis	293	CCGT-AATTTTTTT-TTTGGTGGGGTATTTTTACCCCGCC	330
6.OM160874.1_F.guadeloupense	304	CCGT-AATTTTTTT----GGTGGGGCACTT--ACCCCGCC	336
7.MH392475.1_F.graminearum	264	TCGT-AATTTTTTT-CCCGGTGGGGCTCAT--ACCCCGCC	299
8.MH582420.1_F.solani	311	CCGT-AATTTTTTT----GGTGGGGCATTT--ACCCCGCC	343
9.MAFF244605_F.oxysporum	291	CCGT-AATTTTTTT----GGTGGGGCATTT--ACCCCGCC	323
10.MAFF237278_F.contaminatum_Hylocereus	338	CCGT-AATTTTTTT----GGTGGGGCACTT--ACCCCGCC	370
11.MAFF237649_F.concentricum__Riceroot	328	CCGT-AATTTTTTT----GGTGGGGCATTT--ACCCCGCC	360
12.MAFF237650_F.concentricum__Wheat	328	CCGT-AATTTTTTT---TGGTGGGGCATTT--ACCCCGCC	361
13.MAFF239869_F.mangiferae__Ryukyupine	317	CCGT-AATTTTTTT----GGTGGGGCATTT--ACCCCGCC	349
14.MAFF240460_F.fujikuroi_Passionfruit	321	CCGT-AATTTTTTT----GGTGGGGCATTT--ACCCCGCC	353
15.MAFF241317_F.graminearum_Wheat	319	TCGT-AATTTTTTTCCC-GGTGGGGCTCAT--ACCCCGCC	354
16.MAFF242670_F.ipomoeae_Wheat	337	CCGT-AATTTTT-----TGGTGGGGCTTAT--ACCCCGCT	368

```
17.MAFF245129_F.concentricum_Fraxinus                                  318 CCGT-AATTTTTTT---TGGTGGGGCATTT--ACCCCGCC 351
18.MAFF245395_F.cugenangense_Rhubarb                                   321 CCGT-AATTTTTTT----GGTGGGGCACTT--ACCCCGCC 353
19.MAFF246637_F.nirenbergiae_Strawberry                                321 CTGT-AATTTTTTT---TGGTGGGGCACTT--ACCCCGCC 354
20.MAFF246672_F.nirenbergiae_ChinesePeony                              321 CTGT-AATTTTTTT---TGGTGGGGCACTT--ACCCCGCC 354
21.MAFF246697_F.commune_Urallicoriceroot                               322 CCGT-AATTTTTTT---TGGTGGGGCATTT--ACCCCGCC 355
22.MAFF246729_F.falciforme_Angelica                                    330 CCGT-AATTTTTTT---TGGTGGGGCATTT--ACCCCGCC 363
23.MAFF247220_F.duplospermum__Euwallaceasp                             330 CCGTGAATTTTTTT---TGGTGGGGCATTT-TACCCCGCC 365
24.MAFF410760_F.odoratissimum_alpha                                    321 CCGT-AATTTTTTT----GGTGGGGCATTT--ACCCCGCC 353
25.MAFF244605_FusariumoxysporumSchlechtendal_MAFF244605_Tomato          322 CCGT-AATTTTTTT----GGTGGGGCATTT--ACCCCGCC 354
26.MAFF241326_F.asiaticum_Wheat                                        321 TCGT-AATTTTTTTCCCTGGTGGGGCTCAT--ACCCCGCC 357
27.MAFF245014_F.asiaticum_Wildsoybean                                  318 TCGT-AATTTTTTTCCCTGGTGGGGCTCAT--ACCCCGCC 354
28.MAFF150124_F.asiaticum__Wheat                                       318 TCGT-AATTTTTTTTCCTGGTGGGGCTCAT--ACCCCGCC 354
29.OM135603.1F.algeriense                                              343 CCGT-AATTTTTTT----GGTGGGGCATTT--ACCCCGCC 375
30.MAFF237465_F.penzigii_Aloe                                          353 ACCCCGCCGAACATGCGAAGTTGGCATTTTGCCCCACCAC 392
31.MAFF103054_F.oxysporumSchlechtendalf.sp.cucumerinum_Cucumber        321 CCGT-AATTTTTTT----GGTGGGGCACTT--ACCCCGCC 353
32.MAFF712246_F.oxysporumSchlechtendalf.sp.dianthi__Carnation          321 CCGT-AATTTTTTTT--TGGTGGGGCACTT--ACCCCGCC 355
33.MAFF305558_F.oxysporumSchlechtendalf.sp.fragariae__Watermelon       321 CCGT-AATTTTTTT----GGTGGGGCACTT--ACCCCGCC 353
34.MAFF744087_F.oxysporumSchlechtendalf.sp.lactucae__Lettuce           321 CCGT-AATTTTTTT----GGTGGGGCACTT--ACCCCGCC 353
35.MAFF726924_F.oxysporumSchlechtendalf.sp.lagenariae_Whiteflanderedgourd 321 CCGT-AATTTTTTT----GGTGGGGCACTT--ACCCCGCC 353
36.MAFF744003_F.oxysporumSchlechtendalf.sp.lagenariae_Squash)          321 CCGT-AATTTTTTTT---GGTGGGGCACTT--ACCCCGCC 354
37.MAFF305122_F.oxysporumSchlechtendalf.sp.melonis__Melon              321 CCGT-AATTTTTTT----GGTGGGGCACTT--ACCCCGCC 353
38.MAFF306714_F.oxysporumSchlechtendalf.sp.momordicae_Balsampear       321 CCGT-AATTTTTTT----GGTGGGGCACTT--ACCCCGCC 353
39.MAFF238905_F.oxysporumSchlechtendalf.sp.radicis-lycopersici_Tomato  321 CCGT-AATTTTTTTT--TGGTGGGGCACTT--ACCCCGCC 355
40.MAFF150004_F.oxysporumSchlechtendalf.sp.spinaciae_Spinach           321 CCGT-AATTTTTTT----GGTGGGGCACTT--ACCCCGCC 353
41.MAFF247034_F.oxysporumSchlechtendal__Goldenchain                    321 CCGT-AATTTTTTT----GGTGGGGCACTT--ACCCCGCC 353
42.MAFF245747_F.oxysporumSchlechtendalf.sp.callistephi__Chinaaster     321 CCGT-AATTTTTTT----GGTGGGGCACTT--ACCCCGCC 353
43.MAFF305115_FoxysporumSchlechtendalf.sp.batatas__Sweatpotato         342 CCGT-AATTTTTTT---TGGTGGGGCATTT--ACCCCGCC 375
44.MAFF150126_F.asiaticum_Seed                                         318 TCGT-AATTTTTTTCCCTGGTGGGGCTCAT--ACCCCGCC 354
45.MAFF246738_F.solani_Angelica                                        342 CCGT-AATTTTTTT---TGGTGGGGCATTT--ACCCCGCC 375
46.MAFF246664_F.cugenangense_Perilla                                   321 CCGT-AATTTTTTT----GGTGGGGCACTT--ACCCCGCC 353
47.MH582420.1F.solanistrainMRC256                                      331 CCGT-AATTTTTTT----GGTGGGGCATTT--ACCCCGCC 363
48.MAFF240361_F.babinda_Soil                                           339 CCGT-TAATTTTTT----GGTGGGGTATCT--ACCCCGCC 371
49.MAFF242368_F.azukicola_Azukibean                                    344 CCGC-AATTTTTTT---TGGTGGGGCATTT--ACCCCGCC 377
50.MAFF241312_F.asiaticum_Soil,welshonionfield                         318 TCGT-AATTTTTTTCCCTGGTGGGGCTCAT--ACCCCGCC 354
51.LT548416.1_F.culmorumpartialtef1a                                   315 TCGT-AATTTTT----CTGGTGGGGCTCAT--ACCCCGCC 347
52.MAFF150124_F.asiaticum__Wheat                                       318 TCGT-AATTTTTTTTCCTGGTGGGGCTCAT--ACCCCGCC 354
53.MAFF238806_F.begoniae_Oncidiumsp                                    337 CCGT-AATTTTTTT---TGGTGGGGCATTT--ACCCCGCC 370
54.MW594399.1_FusariumincarnatumisolateUD01C                           341 CCGT-AATTTTTTT---TGGTGGGGTTTCA--ACCCCGCT 374
55.OP414923.1Pucciniagraminisf.sp.triticiisolateSHZPgt19               285 ---------------------------------------- 285
56.MT027094.1_BipolarisoryzaestrainOrL-2                                 0 ---------------------------------------- 0
57.ON734360.1_AlternariaalternataisolateH126                             0 ---------------------------------------- 0
58.LC333578.1_StemphyliumlycopersiciSOasp2                               0 ---------------------------------------- 0
59.HQ718583.1_Colletotrichumgloeosporioidesisolateq-1                  567 ---------------------------------------- 567
60.JN241603.1_AtheliarolfsiiisolateSR1                                  21 CCCTAGAAAATTTTCTTGCGTAGATCAATCGTTTATTAT- 59
61.KJ866474.1_RhizoctoniasolanistrainMHL-1                               0 ---------------------------------------- 0
62.JQ672424.1AlternariatriticinaisolateEGS17-061                         0 ---------------------------------------- 0
```

```
63.LT707559.1_P.capsicipartialtef1agene                                0 ---------------------------------------- 0
64.MW090051.1_CurvularialunatastrainCls-3                              0 ---------------------------------------- 0
65.DQ400892.1_Aspergillusterreus                                       0 ---------------------------------------- 0
66.DQ911416.1_Pythiumsp.quercumstrainPy292                             0 ---------------------------------------- 0
67.EU797495.1_Phytophthorasp.oaksoilPoland                             0 ---------------------------------------- 0
68.HM148321.1_Cladosporiumcucumerinum                                 60 CATATCCCTCTGCCCCGCCACAACACCCCGCCTCGTCGCA 99
69.AF398888.1_SclerotiniasclerotiorumisolateSS1                        0 ---------------------------------------- 0
70.AF398888.1_S.sclerotiorumisolateSS1                                 0 ---------------------------------------- 0
71.HPAB545908.1_Verticilliumnonalfalfaeisolate                         0 ---------------------------------------- 0
72.EF433315.1_CeratocystisfimbriatavoucherCMW15052                   522 ---------------------------------------- 522
73.MN159912.1_Botrytiscinerea                                          0 ---------------------------------------- 0
74.MF034741.1_PeltasterfructicolaisolateSRB92                          0 ---------------------------------------- 0
75.LC440360.1_CercosporaasparagiCOasp2                                 0 ---------------------------------------- 0
76.AY944105.1_MagnaportheoryzaeisolateSAG00T3()                       90 TTTTTTCAGCCTGCCCCTCACTTGGTGGGGGATTGAGGGT 129
77.JX266586.1_CochliobolusmiyabeanusvoucherMFLUCC10-0733               0 ---------------------------------------- 0
78.MN393253.1_CorynesporacassiicolaisolateQHD001(MN393253.1UNVERIFIED) 0 ---------------------------------------- 0
79.MF375218.1_AgroatheliarolfsiiisolateBJB24                           0 ---------------------------------------- 0
80.MN106270.1_AgroatheliarolfsiistrainJ-12                             0 ---------------------------------------- 0
81.OQ732628.1_AgroatheliarolfsiiisolateBTCBSr3                         0 ---------------------------------------- 0
82.KY196185.1_ColletotrichumtruncatumstrainPAK53                     468 ---------------------------------------- 468
83.GU935835.1_ColletotrichumcoccodesisolateC96002                    292 GCTAACGTTCATCTTAGCCGCCGAGTTGGGCAAGGGTTC- 330
84.MK085963.1_AlternariatenuissimaisolateSCCZ06                        0 ---------------------------------------- 0
85.MT548042.1_AlternarialongipesstrainKY_2019_012                      0 ---------------------------------------- 0
86.MN356465.1CalonectriamontanaisolateHSP4                           118 TCTGCACATCCTCTGGGTTTGTGGTGGGGCATTTACCCCG 157
87.OL694224.1_CalonectriacanadianastrainF099                         120 TCTGCACATCCTCTGGGTTTGTGGTGGGGCATTTACCCCG 159
88.MK803351.1_NeoscytalidiumdimidiatumstrainKale4-C                    0 ---------------------------------------- 0
89.ON376993.1_CurvulariachiangmaiensisisolateND00J7                    0 ---------------------------------------- 0
90.OQ383346.1_NeoscytalidiumdimidiatumisolateGKH-2                      0 ---------------------------------------- 0
91.MF662595.1_NeoscytalidiumnovaehollandiaeisolateNeNo1                0 ---------------------------------------- 0
92.EF560588.1Melampsoralini                                          380 ---------------------------------------- 380
93.LC590862.1_NeoscytalidiumdimidiatumPSU-HP01TEF1                     0 ---------------------------------------- 0
94.KX278106.1_BotryosphaeriaqingyuanensisstrainCERC2947                0 ---------------------------------------- 0
95.AJ578763.1_Blumeriagraminisf.sp.hordeicyp51                         0 ---------------------------------------- 0
96.MF490858.1_CurvulariadactylocteniicolastrainCPC28810                0 ---------------------------------------- 0
97.KT287115.1_Bipolariscactivoraisolate3.8.6                           0 ---------------------------------------- 0
98.MT560940.1_CurvulariacactivorastrainHLGH0118                        0 ---------------------------------------- 0
99.OM714565.1_CurvulariaplantarumstrainM0134                           0 ---------------------------------------- 0
100.MN159911.1_BotrytiscinereaSICAUCC19-0003                           0 ---------------------------------------- 0
102.GU294713.1LasiodiplodiatheobromaestrainUCD2430TX                   0 ---------------------------------------- 0
103.KX868094.1_Mycosphaerellasp.isolateCRM20.1                        92 TTTGGCGCCCACTTTTCTGGGGTCAAACGCCATGATCTCA 131
104.LC599478.1Pseudocercosporapini-densifloraeMUCC534                  0 ---------------------------------------- 0
105.N584698.1BipolarissetariaestrainKBS4-2                             0 ---------------------------------------- 0

2.OM160859.1_F.buharicum                                             340 ACTCGAGC--TT-----GGCCGCGCAATGCCCTGTT--CC 370
1.LC727524.1_F.buharicum_OKI-1_Okura                                 371 ACTCGAGC--TT-----GGCCGCGCAATGCGCTGTT--CC 401
3.KX302919.1_F.sublunatum                                            337 ACTCGAGCTGGGACACGCAAGCCCTGTTCCCTGCACACAA 376
```

4.LT996094.1_F.convolutans	336	ACTCGAGTCTGGACACGCAAGCCCTGTTCCCTGCACACAA	375
5.OM160861.1_F.abutilonis	331	ACTCGAGC--TT-----GGCTGCGAAATGCCCTGTT--CT	361
6.OM160874.1_F.guadeloupense	337	ACTCGAGC--TG-----GGCCACGCAATGCCCTGAT--CC	367
7.MH392475.1_F.graminearum	300	ACTCGAGCGACAGGCGTCTGCCCTCTTCCCACAAACCATT	339
8.MH582420.1_F.solani	344	ACTCGGGCGACGTTGGACAAAGCCCTGATCCCTGCACACA	383
9.MAFF244605_F.oxysporum	324	ACTCGAGCGACG-----GGC-GCGTT-TGCCCTCCTC-CC	355
10.MAFF237278_F.contaminatum_Hylocereus	371	ACTTGAGCGACG-----GGA-GCGTT-TGCCCTCTTAACC	403
11.MAFF237649_F.concentricum__Riceroot	361	ACTCGAGCGATG-----GGC-GCGTT-TGCCCTC------	387
12.MAFF237650_F.concentricum__Wheat	362	ACTCGAGCGATG-----GGC-GCGTT-TGCCCTC------	388
13.MAFF239869_F.mangiferae__Ryukyupine	350	ACTCGAGCGATG-----GGC-GCGTT-TGCCCTC------	376
14.MAFF240460_F.fujikuroi_Passionfruit	354	ACTCGAGTGATG-----GGC-GCGTT-TTGCC-CTTT-CC	384
15.MAFF241317_F.graminearum_Wheat	355	ACTCGAGCGACA-----GG---CGTC-TGCCCTCTT---C	382
16.MAFF242670_F.ipomoeae_Wheat	369	ACTCGAGTGACA-----GG---CGCT-TGCCCTCTT---C	396
17.MAFF245129_F.concentricum_Fraxinus	352	ACTCGAGCGATG-----GGC-GCGTT-TGCCCTC------	378
18.MAFF245395_F.cugenangense_Rhubarb	354	ACTTGAGCGACG-----GGA-GCGTT-TGCCCTCTTA-CC	385
19.MAFF246637_F.nirenbergiae_Strawberry	355	ACTTGAGCGACG-----GGA-GCGTT-TGCCCTCTTAACC	387
20.MAFF246672_F.nirenbergiae_ChinesePeony	355	ACTTGAGCGACG-----GGA-GCGTT-TGCCCTCTTAACC	387
21.MAFF246697_F.commune_Urallicoriceroot	356	ACTCGAGCGACG-----GGC-GCGTT-TGCCCTCCTC-CC	387
22.MAFF246729_F.falciforme_Angelica	364	ACTCGGGCGACGTTGGACAAAGCCCTGATCCCTGCACACA	403
23.MAFF247220_F.duplospermum__Euwallaceasp	366	ACTCGGGCGACGTTGGACAAAGCCCTGATCCCTGCACACA	405
24.MAFF410760_F.odoratissimum_alpha	354	ACTTGAGCGACG-----GGGCGCGTT-TGCCCTCTTA-CC	386
25.MAFF244605_FusariumoxysporumSchlechtendal_MAFF244605_Tomato	355	ACTCGAGCGACG-----GGC-GCGTT-TGCCCTCCTC-CC	386
26.MAFF241326_F.asiaticum_Wheat	358	ACTCGAGCGACA-----GG---CGTT-TGCCCTCTT---C	385
27.MAFF245014_F.asiaticum_Wildsoybean	355	ACTCGAGCGACA-----GG---CGTT-TGCCCTCTT---C	382
28.MAFF150124_F.asiaticum__Wheat	355	ACTCGAGCGACA-----GG---CGTT-TGCCCTCTT---C	382
29.OM135603.1F.algeriense	376	ACTCGAGCGTTG-----GGC-GCGCT-TGCCCTTCT--CC	406
30.MAFF237465_F.penzigii_Aloe	393	AAAAAATTTCTCACATTCTCAATTCATGGCTCGTCACAAG	432
31.MAFF103054_F.oxysporumSchlechtendalf.sp.cucumerinum_Cucumber	354	ACTTGAGCGACG-----GGA-GCGTT-TGCCCTCTTA-CC	385
32.MAFF712246_F.oxysporumSchlechtendalf.sp.dianthi__Carnation	356	ACTTGAGCGACG-----GGA-GCGTT-TGCCCTCTTA-CC	387
33.MAFF305558_F.oxysporumSchlechtendalf.sp.fragariae__Watermelon	354	ACTTGAGCGACG-----GGA-GCGTT-TGCCCTCTTA-CC	385
34.MAFF744087_F.oxysporumSchlechtendalf.sp.lactucae__Lettuce	354	ACTTGAGCGACG-----GGA-GCGTT-TGCCCTCTTA-CC	385
35.MAFF726924_F.oxysporumSchlechtendalf.sp.lagenariae_Whitefloweredgourd	354	ACTTGAGCGACG-----GGA-GCGTT-TGCCCTCTTA-CC	385
36.MAFF744003_F.oxysporumSchlechtendalf.sp.lagenariae_Squash)	355	ACTTGAGCGACG-----GGA-GCGTT-TGCCCTCTTA-CC	386
37.MAFF305122_F.oxysporumSchlechtendalf.sp.melonis__Melon	354	ACTTGAGCGACG-----GGA-GCGTT-TGCCTTCTTA-CC	385
38.MAFF306714_F.oxysporumSchlechtendalf.sp.momordicae_Balsampear	354	ACTTGAGCGACG-----GGA-GCGTT-TGCCCTCTTA-CC	385
39.MAFF238905_F.oxysporumSchlechtendalf.sp.radicis-lycopersici_Tomato	356	ACTTGAGCGACG-----GGA-GCGTT-TGCCCTCTTAACC	388
40.MAFF150004_F.oxysporumSchlechtendalf.sp.spinaciae_Spinach	354	ACTTGAGCGACG-----GGA-GCGTT-TGCCCTCTTA-CC	385
41.MAFF247034_F.oxysporumSchlechtendal__Goldenchain	354	ACTTGAGCGACG-----GGA-GCGTT-TGCCCTCTTA-CC	385
42.MAFF245747_F.oxysporumSchlechtendalf.sp.callistephi__Chinaaster	354	ACTTGAGCGAAG-----GGA-GCGTT-TGCCCTCTTA-CC	385
43.MAFF305115_FoxysporumSchlechtendalf.sp.batatas__Sweatpotato	376	ACTCGGGCGACGTTGGACAAAGCCCTGATCCCTGCACACA	415
44.MAFF150126_F.asiaticum_Seed	355	ACTCGAGCGACA-----GG---CGTT-TGCCCTCTT---C	382
45.MAFF246738_F.solani_Angelica	376	ACTCGGGCGACGTTGGACAAAGCCCTGATCCCTGCACACA	415
46.MAFF246664_F.cugenangense_Perilla	354	ACTTGAGCGACG-----GGA-GCGTT-TGCCCTCTTA-CC	385
47.MH582420.1F.solanistrainMRC256	364	ACTCGGGCGACGTTGGACAAAGCCCTGATCCCTGCACACA	403
48.MAFF240361_F.babinda_Soil	372	ACTCGAGTGACG-----GGC-GCGCT-TGCCCTGTT--CC	402
49.MAFF242368_F.azukicola_Azukibean	378	GCTCGCGAGACGTCGGACAAAGCCCTGATCCCTGCACACA	417

50.MAFF241312_F.asiaticum_Soil,welshonionfield	355	ACTCGAGCGACA-----GG---CGTT-TGCCCTCTT---C	382
51.LT548416.1_F.culmorumpartialtef1a	348	ACTCGAGCGACA-----GG---CGCT-TGCCCTCTT---C	375
52.MAFF150124_F.asiaticum__Wheat	355	ACTCGAGCGACA-----GG---CGTT-TGCCCTCTT---C	382
53.MAFF238806_F.begoniae_Oncidiumsp	371	ACTCGAGCGATG-----CGC-GTTTC-TGCCCTC----TC	399
54.MW594399.1_FusariumincarnatumisolateUD01C	375	ACTCGAGCGACA-----GAC---GTT-TGCCCTC------	399
55.OP414923.1Pucciniagraminisf.sp.triticiisolateSHZPgt19	285	--	285
56.MT027094.1_BipolarisoryzaestrainOrL-2	0	--	0
57.ON734360.1_AlternariaalternataisolateH126	0	--	0
58.LC333578.1_StemphyliumlycopersiciSOasp2	0	--	0
59.HQ718583.1_Colletotrichumgloeosporioidesisolateq-1	567	--	567
60.JN241603.1_AtheliarolfsiiisolateSR1	59	--	59
61.KJ866474.1_RhizoctoniasolanistrainMHL-1	0	--	0
62.JQ672424.1AlternariatriticinaisolateEGS17-061	0	--	0
63.LT707559.1_P.capsicipartialtef1agene	0	--	0
64.MW090051.1_CurvularialunatastrainCls-3	0	--	0
65.DQ400892.1_Aspergillusterreus	0	--	0
66.DQ911416.1_Pythiumsp.quercumstrainPy292	0	--	0
67.EU797495.1_Phytophthorasp.oaksoilPoland	0	--	0
68.HM148321.1_Cladosporiumcucumerinum	100	AATTTGCGATAAGGATGTGGGCCGCCCTGGCTTGGCACGG	139
69.AF398888.1_SclerotiniasclerotiorumisolateSS1	0	--	0
70.AF398888.1_S.sclerotiorumisolateSS1	0	--	0
71.HPAB545908.1_Verticilliumnonalfalfaeisolate	0	--	0
72.EF433315.1_CeratocystisfimbriatavoucherCMW15052	522	--	522
73.MN159912.1_Botrytiscinerea	0	--	0
74.MF034741.1_PeltasterfructicolaisolateSRB92	0	--	0
75.LC440360.1_CercosporaasparagiCOasp2	0	--	0
76.AY944105.1_MagnaportheoryzaeisolateSAG00T3()	130	CTCACTTTTTCAACCCTTGTTCTTTTCGTCATACACACAA	169
77.JX266586.1_CochliobolusmiyabeanusvoucherMFLUCC10-0733	0	--	0
78.MN393253.1_CorynesporacassiicolaisolateQHD001(MN393253.1UNVERIFIED)	0	--	0
79.MF375218.1_AgroatheliarolfsiiisolateBJB24	0	--	0
80.MN106270.1_AgroatheliarolfsiistrainJ-12	0	--	0
81.OQ732628.1_AgroatheliarolfsiiisolateBTCBSr3	0	--	0
82.KY196185.1_ColletotrichumtruncatumstrainPAK53	468	--	468
83.GU935835.1_ColletotrichumcoccodesisolateC96002	330	--	330
84.MK085963.1_AlternariatenuissimaisolateSCCZ06	0	--	0
85.MT548042.1_AlternarialongipesstrainKY_2019_012	0	--	0
86.MN356465.1CalonectriamontanaisolateHSP4	158	CCGCCCACAGGAGGTCGTCAAACCTGCACTGCCCCACTTC	197
87.OL694224.1_CalonectriacanadianastrainF099	160	CCGCCCACAGGAGGTCGTCAAACCTGCACTGCCCCACTTC	199
88.MK803351.1_NeoscytalidiumdimidiatumstrainKale4-C	0	--	0
89.ON376993.1_CurvulariachiangmaiensisisolateND00J7	0	--	0
90.OQ383346.1_NeoscytalidiumdimidiatumisolateGKH-2	0	--	0
91.MF662595.1_NeoscytalidiumnovaehollandiaeisolateNeNo1	0	--	0
92.EF560588.1Melampsoralini	380	--	380
93.LC590862.1_NeoscytalidiumdimidiatumPSU-HP01TEF1	0	--	0
94.KX278106.1_BotryosphaeriaqingyuanensisstrainCERC2947	0	--	0
95.AJ578763.1_Blumeriagraminisf.sp.hordeicyp51	0	--	0

```
96.MF490858.1_CurvulariadactylocteniicolastrainCPC28810              0 ---------------------------------------- 0
97.KT287115.1_Bipolariscactivoraisolate3.8.6                         0 ---------------------------------------- 0
98.MT560940.1_CurvulariacactivorastrainHLGH0118                      0 ---------------------------------------- 0
99.OM714565.1_CurvulariaplantarumstrainM0134                         0 ---------------------------------------- 0
100.MN159911.1_BotrytiscinereaSICAUCC19-0003                         0 ---------------------------------------- 0
102.GU294713.1LasiodiplodiatheobromaestrainUCD2430TX                 0 ---------------------------------------- 0
103.KX868094.1_Mycosphaerellasp.isolateCRM20.1                     132 TCCATCACCGCCATATGCCTTCTCAGCAGCTCTCGAGCAC 171
104.LC599478.1Pseudocercosporapini-densifloraeMUCC534                0 ---------------------------------------- 0
105.N584698.1BipolarissetariaestrainKBS4-2                           0 ---------------------------------------- 0

2.OM160859.1_F.buharicum                                           371 CTGC--ACACATAATCACTTAG-GCGC-TCGTCA--TG-- 402
1.LC727524.1_F.buharicum_OKI-1_Okura                               402 CTGC--ACACATAATCACTTAG-GCGC-TCGTCA--TG-- 433
3.KX302919.1_F.sublunatum                                          377 TCATCGTCCGGGCGCGATCATGATGCATGAAACAGTTGCT 416
4.LT996094.1_F.convolutans                                         376 TCATCATTCGGGTGCGATCATGATGTGTCAATCAGTTGCT 415
5.OM160861.1_F.abutilonis                                          362 CTGC--ACACACAATCACTTGG-GCTC-TTATCA--TG-- 393
6.OM160874.1_F.guadeloupense                                       368 CTGC--ACACA-AACAATTCTG-GCGCGTCATTACCTG-- 401
7.MH392475.1_F.graminearum                                         340 CCCTGGGCGCTCATCATCACGTGTCAACCAGTCACTAACC 379
8.MH582420.1_F.solani                                              384 AAAA--CACCAAACCCTCTTGGCGCGCATCATCACGTGGT 421
9.MAFF244605_F.oxysporum                                           356 ATTT--CCACAACCTCAATGAGCGCA--TCGTCACGTG-- 389
10.MAFF237278_F.contaminatum_Hylocereus                            404 ATTC--TCACAACCTCAATGAGTGCG--TCGTCACGTG-- 437
11.MAFF237649_F.concentricum__Riceroot                             388 --TC---CACAATCTCAATGAGCGCA--TCGTCACGTG-- 418
12.MAFF237650_F.concentricum__Wheat                                389 --TC---CACAATCTCAATGAGCGCA--TCGTCACGTG-- 419
13.MAFF239869_F.mangiferae__Ryukyupine                             377 --TC---CACAACCTCAATGAGCGCA--TCGTCACGTG-- 407
14.MAFF240460_F.fujikuroi_Passionfruit                             385 TGTC---CACAACCTCAATGAGCGCA--TTGTCACGTG-- 417
15.MAFF241317_F.graminearum_Wheat                                  383 CCAC--AAACCAT-TCCCTGGGCGCTCATCATCACGTG-- 417
16.MAFF242670_F.ipomoeae_Wheat                                     397 CCAC--AAA---A-TCACTTG------CGCATCACGTG-- 422
17.MAFF245129_F.concentricum_Fraxinus                              379 --TC---CACAATCTCAATGAGCGCA--TCGTCACGTG-- 409
18.MAFF245395_F.cugenangense_Rhubarb                               386 ATTC--TCAGAACCTCAATGAGTGCG--TCGTCACGTG-- 419
19.MAFF246637_F.nirenbergiae_Strawberry                            388 ATTC--TCACAACCTCAATGAGTGCG--TCGTCACGTG-- 421
20.MAFF246672_F.nirenbergiae_ChinesePeony                          388 ATTC--TCACAACCTCAATGAGTGCG--TCGTCACGTG-- 421
21.MAFF246697_F.commune_Urallicoriceroot                           388 ATTT--CCACAACCTCAATGAGCGCA--TCGTCACGTG-- 421
22.MAFF246729_F.falciforme_Angelica                                404 AAAA--CACCAAACCCTCTTGGCGCGCATCATCACGTGGT 441
23.MAFF247220_F.duplospermum__Euwallaceasp                         406 AAAAA-CACCAAACCCTCTTGGCGCGCATCATCACGTGTT 444
24.MAFF410760_F.odoratissimum_alpha                                387 ATT-----ACAACCTCAATGAGTGCG--TCGTCACGTG-- 417
25.MAFF244605_FusariumoxysporumSchlechtendal_MAFF244605_Tomato      387 ATTT--CCACAACCTCAATGAGCGCA--TCGTCACGTG-- 420
26.MAFF241326_F.asiaticum_Wheat                                    386 CCAC--AAACCAT-TCCCTGGGCGCTCATCATCACGTG-- 420
27.MAFF245014_F.asiaticum_Wildsoybean                              383 CCAC--AAACCAT-TCCCTGGGCGCTCATCATCACGTG-- 417
28.MAFF150124_F.asiaticum__Wheat                                   383 CCAC--AAACCAT-TCCCTGGGCGCTCATCATCACGTG-- 417
29.OM135603.1F.algeriense                                          407 CACA--TAA-----CTCATGAGCGCACATCATCACGTG-- 437
30.MAFF237465_F.penzigii_Aloe                                      433 CAATCCAAACATGATGCTAACAA-CTCACCAATAGGAAGC 471
31.MAFF103054_F.oxysporumSchlechtendalf.sp.cucumerinum_Cucumber    386 ATTC--TCAGAACCTCAATGAGTGCG--TCGTCACGTG-- 419
32.MAFF712246_F.oxysporumSchlechtendalf.sp.dianthi__Carnation      388 ATTC--TCACAACCTCAATGAGTGCG--TCGTCACGTG-- 421
33.MAFF305558_F.oxysporumSchlechtendalf.sp.fragariae__Watermelon   386 ATTC--TCAGAACCTCAATGAGTGCG--TCGTCACGTG-- 419
34.MAFF744087_F.oxysporumSchlechtendalf.sp.lactucae__Lettuce       386 ATTC--TCAGAACCTCAATGAGTGCG--TCGTCACGTG-- 419
35.MAFF726924_F.oxysporumSchlechtendalf.sp.lagenariae_Whitefloweredgourd 386 ATTC--TCAGAACCTCAATGAGTGCG--TCGTCACGTG-- 419
36.MAFF744003_F.oxysporumSchlechtendalf.sp.lagenariae_Squash)      387 ATTC--TCACAACCTCAATGAGTGCG--TCGTCACGTG-- 420
```

```
37.MAFF305122_F.oxysporumSchlechtendalf.sp.melonis__Melon              386 ATTC--TCAGAACCTCAATGAGTGCG--TCGTCACGTG-- 419
38.MAFF306714_F.oxysporumSchlechtendalf.sp.momordicae_Balsampear       386 ATTC--TCAGAACCTCAATGAGTGCG--TCGTCACGTG-- 419
39.MAFF238905_F.oxysporumSchlechtendalf.sp.radicis-lycopersici_Tomato  389 ATTC--TCACAACCTCAATCAGTGCG--TCGTCACGTG-- 422
40.MAFF150004_F.oxysporumSchlechtendalf.sp.spinaciae_Spinach           386 ATTC--TCAGAACCTCAATGAGTGCG--TCGTCACGTG-- 419
41.MAFF247034_F.oxysporumSchlechtendal__Goldenchain                    386 ATTC--TCAGAACCTCAATGAGTGCG--TCGTCACGTG-- 419
42.MAFF245747_F.oxysporumSchlechtendalf.sp.callistephi__Chinaaster     386 ATTC--TCACAACCTCAATGAGTGCG--TCGTCACGTG-- 419
43.MAFF305115_FoxysporumSchlechtendalf.sp.batatas__Sweatpotato         416 AAAA--CACCAAATCCTCTTGGCGCGCATCATCACGTGGT 453
44.MAFF150126_F.asiaticum_Seed                                         383 CCAC--AAACCAT-TCCCTGGGCGCTCATCATCACGTG-- 417
45.MAFF246738_F.solani_Angelica                                        416 AAAA--CACCAAATCCTCTTGGCGCGCATCATCACGTGGT 453
46.MAFF246664_F.cugenangense_Perilla                                   386 ATTC--TCAGAACCTCAATGAGTGCG--TCGTCACGTG-- 419
47.MH582420.1F.solanistrainMRC256                                      404 AAAA--CACCAAACCCTCTTGGCGCGCATCATCACGTGGT 441
48.MAFF240361_F.babinda_Soil                                           403 CACA--AAATCATCATAATGGGCGCGCATCATCACGTG-- 438
49.MAFF242368_F.azukicola_Azukibean                                    418 AAAAAACACCAAACCCTCTTGGCGCGCATCGTCACGTGGT 457
50.MAFF241312_F.asiaticum_Soil,welshonionfield                         383 CCAC--AAACCAT-TCCCTGGGCGCTCATCATCACGTG-- 417
51.LT548416.1_F.culmorumpartialtef1a                                   376 CCAC--AAACCAT-TCCCTAGGCGCGCACCATCACGTG-- 410
52.MAFF150124_F.asiaticum__Wheat                                       383 CCAC--AAACCAT-TCCCTGGGCGCTCATCATCACGTG-- 417
53.MAFF238806_F.begoniae_Oncidiumsp                                    400 ATTG--CCACAACCTTC-TGAGCGCA--TCGTCACGTG-- 432
54.MW594399.1_FusariumincarnatumisolateUD01C                           400 -TTC--CCACAAACTCATGTCTTGTG---CATCACGTG-- 431
55.OP414923.1Pucciniagraminisf.sp.triticiisolateSHZPgt19               285 ---------------------------------------- 285
56.MT027094.1_BipolarisoryzaestrainOrL-2                                 0 ---------------------------------------- 0
57.ON734360.1_AlternariaalternataisolateH126                             0 ---------------------------------------- 0
58.LC333578.1_StemphyliumlycopersiciSOasp2                               0 ---------------------------------------- 0
59.HQ718583.1_Colletotrichumgloeosporioidesisolateq-1                  567 ---------------------------------------- 567
60.JN241603.1_AtheliarolfsiiisolateSR1                                  59 ---------------------------------------- 59
61.KJ866474.1_RhizoctoniasolanistrainMHL-1                               0 ---------------------------------------- 0
62.JQ672424.1AlternariatriticinaisolateEGS17-061                         0 ---------------------------------------- 0
63.LT707559.1_P.capsicipartialtef1agene                                  0 ---------------------------------------- 0
64.MW090051.1_CurvularialunatastrainCls-3                                0 ---------------------------------------- 0
65.DQ400892.1_Aspergillusterreus                                         0 ---------------------------------------- 0
66.DQ911416.1_Pythiumsp.quercumstrainPy292                               0 ---------------------------------------- 0
67.EU797495.1_Phytophthorasp.oaksoilPoland                               0 ---------------------------------------- 0
68.HM148321.1_Cladosporiumcucumerinum                                  140 ACGTTCATCACGACAAGACAGCATTGCCACCCTCCACCAC 179
69.AF398888.1_SclerotiniasclerotiorumisolateSS1                          0 ---------------------------------------- 0
70.AF398888.1_S.sclerotiorumisolateSS1                                   0 ---------------------------------------- 0
71.HPAB545908.1_Verticilliumnonalfalfaeisolate                           0 ---------------------------------------- 0
72.EF433315.1_CeratocystisfimbriatavoucherCMW15052                     522 ---------------------------------------- 522
73.MN159912.1_Botrytiscinerea                                            0 ---------------------------------------- 0
74.MF034741.1_PeltasterfructicolaisolateSRB92                            0 ---------------------------------------- 0
75.LC440360.1_CercosporaasparagiCOasp2                                   0 ---------------------------------------- 0
76.AY944105.1_MagnaportheoryzaeisolateSAG00T3()                        170 AATTTCCATTAGTGGCCCGAGCGCCACCACTTGCAAGATT 209
77.JX266586.1_CochliobolusmiyabeanusvoucherMFLUCC10-0733                 0 ---------------------------------------- 0
78.MN393253.1_CorynesporacassiicolaisolateQHD001(MN393253.1UNVERIFIED)   0 ---------------------------------------- 0
79.MF375218.1_AgroatheliarolfsiiisolateBJB24                             0 ---------------------------------------- 0
80.MN106270.1_AgroatheliarolfsiistrainJ-12                               0 ---------------------------------------- 0
81.OQ732628.1_AgroatheliarolfsiiisolateBTCBSr3                           0 ---------------------------------------- 0
82.KY196185.1_ColletotrichumtruncatumstrainPAK53                       468 ---------------------------------------- 468
```

```
83.GU935835.1_ColletotrichumcoccodesisolateC96002          330 ---------------------------------------- 330
84.MK085963.1_AlternariatenuissimaisolateSCCZ06              0 ---------------------------------------- 0
85.MT548042.1_AlternarialongipesstrainKY_2019_012            0 ---------------------------------------- 0
86.MN356465.1CalonectriamontanaisolateHSP4                 198 GGCTTTCACACCAAATCACATTTGGCATATCGCCACACAT 237
87.OL694224.1_CalonectriacanadianastrainF099               200 GGCTTTCACACCAAATCACATTTGGCATATCGCCACACAT 239
88.MK803351.1_NeoscytalidiumdimidiatumstrainKale4-C          0 ---------------------------------------- 0
89.ON376993.1_CurvulariachiangmaiensisisolateND00J7          0 ---------------------------------------- 0
90.OQ383346.1_NeoscytalidiumdimidiatumisolateGKH-2           0 ---------------------------------------- 0
91.MF662595.1_NeoscytalidiumnovaehollandiaeisolateNeNo1      0 ---------------------------------------- 0
92.EF560588.1Melampsoralini                                380 ---------------------------------------- 380
93.LC590862.1_NeoscytalidiumdimidiatumPSU-HP01TEF1           0 ---------------------------------------- 0
94.KX278106.1_BotryosphaeriaqingyuanensisstrainCERC2947      0 ---------------------------------------- 0
95.AJ578763.1_Blumeriagraminisf.sp.hordeicyp51               0 ---------------------------------------- 0
96.MF490858.1_CurvulariadactylocteniicolastrainCPC28810      0 ---------------------------------------- 0
97.KT287115.1_Bipolariscactivoraisolate3.8.6                 0 ---------------------------------------- 0
98.MT560940.1_CurvulariacactivorastrainHLGH0118              0 ---------------------------------------- 0
99.OM714565.1_CurvulariaplantarumstrainM0134                 0 ---------------------------------------- 0
100.MN159911.1_BotrytiscinereaSICAUCC19-0003                 0 ---------------------------------------- 0
102.GU294713.1LasiodiplodiatheobromaestrainUCD2430TX         0 ---------------------------------------- 0
103.KX868094.1_Mycosphaerellasp.isolateCRM20.1             172 AACACCGTACACCAAGCGCACAAACACATAACATCACGCA 211
104.LC599478.1Pseudocercosporapini-densifloraeMUCC534        0 ---------------------------------------- 0
105.N584698.1BipolarissetariaestrainKBS4-2                   0 ---------------------------------------- 0

2.OM160859.1_F.buharicum                                   403 TGAAA-CAGTTGCTGACCACCTCGACAATAGGAAGCCGCC 441
1.LC727524.1_F.buharicum_OKI-1_Okura                       434 TGA---CAGTTGCTGACCACCTCGACAATAGGAAGCCGCC 470
3.KX302919.1_F.sublunatum                                  417 AACCACCTTGACAATAGGAAGCCGCCGAGCTCGGTAA--- 453
4.LT996094.1_F.convolutans                                 416 AACCACCTCGATAACAGGAAGCCGCCGAGCTCGGTAA--- 452
5.OM160861.1_F.abutilonis                                  394 TGAAA-CACTTGCTAACCACTTCGACAATAGGAAGCCGCC 432
6.OM160874.1_F.guadeloupense                               402 TGAGA-CAGTTGCTAACCACTTGGACAATAGGAAGCCGCC 440
7.MH392475.1_F.graminearum                                 380 ACCTGTCAATAGGAAGCCGCCGAGCTCGGTAAGGGTTCCT 419
8.MH582420.1_F.solani                                      422 TCACGAC-GACGCTAACCGGTCCAACAATAGGAAGCCGCT 460
9.MAFF244605_F.oxysporum                                   390 TCAAG-CAGTCACTAACCA-TTCAATAATAGGAAGCCGCT 427
10.MAFF237278_F.contaminatum_Hylocereus                    438 TCAAG-CAGTCACTAACCA-TTCAACAATAGGAAGCCGCT 475
11.MAFF237649_F.concentricum__Riceroot                     419 CCAAG-CAGTCACTAACCA-TCCGACAATAGGAAGCCGCT 456
12.MAFF237650_F.concentricum__Wheat                        420 CCAAG-CAGTCACTAACCA-TCCGACAATAGGAAGCCGCT 457
13.MAFF239869_F.mangiferae__Ryukyupine                     408 TCAAG-CAGTCACTAACCG-TCCGACAATAGGAAGCCGCT 445
14.MAFF240460_F.fujikuroi_Passionfruit                     418 TCAAA-C------TAACCA-TTCGACAATAGGAAGCCGCT 449
15.MAFF241317_F.graminearum_Wheat                          418 TCAAC-CAGTCACTAACCA-CCTGTCAATAGGAAGCCGCC 455
16.MAFF242670_F.ipomoeae_Wheat                             423 TCAAT-CAGTCACTAACCA-CTCGACAATAGGAAGCCGCC 460
17.MAFF245129_F.concentricum_Fraxinus                      410 CCAAG-CAGTCACTAACCA-TCCGACAATAGGAAGCCGCT 447
18.MAFF245395_F.cugenangense_Rhubarb                       420 TCAAG-CAGTCACTAACCA-TTCAACAATAGGAAGCCGCT 457
19.MAFF246637_F.nirenbergiae_Strawberry                    422 TCAAG-CAGTCACTAACCA-TTCAACAATAGGAAGCCGCT 459
20.MAFF246672_F.nirenbergiae_ChinesePeony                  422 TCAAG-CAGTCACTAACCA-TTCAACAATAGGAAGCCGCT 459
21.MAFF246697_F.commune_Urallicoriceroot                   422 TCACG-CAGTCACTAACCA-TTCAATAATAGGAAGCCGCT 459
22.MAFF246729_F.falciforme_Angelica                        442 TCACAACAAACGCTAACCGGTCCAACAATAGGAAGCCGCT 481
23.MAFF247220_F.duplospermum__Euwallaceasp                 445 TGACCACAGACGCTAACCGACTCAACAACAGGAAGCCGCT 484
```

```
24.MAFF410760_F.odoratissimum_alpha                                    418 TCAAG-CAGTCACTAACCA-TTCAATAATAGGAAGCCGCT 455
25.MAFF244605_FusariumoxysporumSchlechtendal_MAFF244605_Tomato         421 TCAAG-CAGTCACTAACCA-TTCAATAATAGGAAGCCGCT 458
26.MAFF241326_F.asiaticum_Wheat                                        421 TCAAC-CAGTCACTAACCA-CCTGTCAATAGGAAGCCGCC 458
27.MAFF245014_F.asiaticum_Wildsoybean                                  418 TCAAC-CAGTCACTAACCA-CCTGTCAATAGGAAGCCGCC 455
28.MAFF150124_F.asiaticum__Wheat                                       418 TCAAC-CAGTCACTAACCA-CCTGTCAATAGGAAGCCGCC 455
29.OM135603.1F.algeriense                                              438 TCAAG-CAGTAACTAACCA-CTGGACAATAGGAAGCCGCT 475
30.MAFF237465_F.penzigii_Aloe                                          472 CGCCGAGCTCGGTAA------------------------- 486
31.MAFF103054_F.oxysporumSchlechtendalf.sp.cucumerinum_Cucumber        420 TCAAG-CAGTCACTAACCA-TTCAACAATAGGAAGCCGCT 457
32.MAFF712246_F.oxysporumSchlechtendalf.sp.dianthi__Carnation          422 TCAAG-CAGTCACTAACCA-TTCAACAATAGGAAGCCGCT 459
33.MAFF305558_F.oxysporumSchlechtendalf.sp.fragariae__Watermelon       420 TCAAG-CAGTCACTAACCA-TTCAACAATAGGAAGCCGCT 457
34.MAFF744087_F.oxysporumSchlechtendalf.sp.lactucae__Lettuce           420 TCAAG-CAGTCACTAACCA-TTCAACAATAGGAAGCCGCT 457
35.MAFF726924_F.oxysporumSchlechtendalf.sp.lagenariae_Whitefloweredgourd 420 TCAAG-CAGTCACTAACCA-TTCAACAATAGGAAGCCGCT 457
36.MAFF744003_F.oxysporumSchlechtendalf.sp.lagenariae_Squash)          421 TCAAG-CAGTCACTAACCA-TTCAACAATAGGAAGCCGCT 458
37.MAFF305122_F.oxysporumSchlechtendalf.sp.melonis__Melon              420 TCAAG-CAGTCACTAACCA-TTCAACAATAGGAAGCCGCT 457
38.MAFF306714_F.oxysporumSchlechtendalf.sp.momordicae_Balsampear       420 TCAAG-CAGTCACTAACCA-TTCAACAATAGGAAGCCGCT 457
39.MAFF238905_F.oxysporumSchlechtendalf.sp.radicis-lycopersici_Tomato  423 TCAAG-CAGTCACTAACCA-TTCAACAATAGGAAGCCGCT 460
40.MAFF150004_F.oxysporumSchlechtendalf.sp.spinaciae_Spinach           420 TCAAG-CAGTCACTAACCA-TTCAACAATAGGAAGCCGCT 457
41.MAFF247034_F.oxysporumSchlechtendal__Goldenchain                    420 TCAAG-CAGTCACTAACCA-TTCAACAATAGGAAGCCGCT 457
42.MAFF245747_F.oxysporumSchlechtendalf.sp.callistephi__Chinaaster     420 TGAAG-CAGTCACTAACCA-TTCAACAATAGGAAGCCGCT 457
43.MAFF305115_FoxysporumSchlechtendalf.sp.batatas__Sweatpotato         454 TCACGACAGACGCTAACCGGTCCAACAATAGGAAGCCGCT 493
44.MAFF150126_F.asiaticum_Seed                                         418 TCAAC-CAGTCACTAACCA-CCTGTCAATAGGAAGCCGCC 455
45.MAFF246738_F.solani_Angelica                                        454 TCACGACAGACGCTAACCGGTCCAACAATAGGAAGCCGCT 493
46.MAFF246664_F.cugenangense_Perilla                                   420 TCAAG-CAGTCACTAACCA-TTCAACAATAGGAAGCCGCT 457
47.MH582420.1F.solanistrainMRC256                                      442 TCACGAC-GACGCTAACCGGTCCAACAATAGGAAGCCGCT 480
48.MAFF240361_F.babinda_Soil                                           439 TCAAT-CAGTCACTAACCA-TTTGATAATAGGAAGCCGCT 476
49.MAFF242368_F.azukicola_Azukibean                                    458 CCGCGACAGAAGCTAACCGACTCAACAATAGGAAGCCGCT 497
50.MAFF241312_F.asiaticum_Soil,welshonionfield                         418 TCAAC-CAGTCACTAACCA-CCTGTCAATAGGAAGCCGCC 455
51.LT548416.1_F.culmorumpartialtef1a                                   411 TCAAT-CAGTTACTAACCA-CCTGTCAATAGGAAGCCGCC 448
52.MAFF150124_F.asiaticum__Wheat                                       418 TCAAC-CAGTCACTAACCA-CCTGTCAATAGGAAGCCGCC 455
53.MAFF238806_F.begoniae_Oncidiumsp                                    433 TTAAG-CAGTCACTAACCA-TTCGACAATAGGAAGCCGCT 470
54.MW594399.1_FusariumincarnatumisolateUD01C                           432 TCCAT-CAGCCACTAACCA-CCCGACAATAGGAAGCCGCC 469
55.OP414923.1Pucciniagraminisf.sp.triticiisolateSHZPgt19               285 ---------------------------------------- 285
56.MT027094.1_BipolarisoryzaestrainOrL-2                                 0 ---------------------------------------- 0
57.ON734360.1_AlternariaalternataisolateH126                             0 ---------------------------------------- 0
58.LC333578.1_StemphyliumlycopersiciSOasp2                               0 ---------------------------------------- 0
59.HQ718583.1_Colletotrichumgloeosporioidesisolateq-1                  567 ---------------------------------------- 567
60.JN241603.1_AtheliarolfsiiisolateSR1                                  59 ---------------------------------------- 59
61.KJ866474.1_RhizoctoniasolanistrainMHL-1                               0 ---------------------------------------- 0
62.JQ672424.1AlternariatriticinaisolateEGS17-061                         0 ---------------------------------------- 0
63.LT707559.1_P.capsicipartialtef1agene                                  0 ---------------------------------------- 0
64.MW090051.1_CurvularialunatastrainCls-3                                0 ---------------------------------------- 0
65.DQ400892.1_Aspergillusterreus                                         0 ---------------------------------------- 0
66.DQ911416.1_Pythiumsp.quercumstrainPy292                               0 ---------------------------------------- 0
67.EU797495.1_Phytophthorasp.oaksoilPoland                               0 ---------------------------------------- 0
68.HM148321.1_Cladosporiumcucumerinum                                  180 ACGCTGGACAGATTACTGACAATTACTACAGGAAGCCGCC 219
69.AF398888.1_SclerotiniasclerotiorumisolateSS1                          0 ---------------------------------------- 0
```

70.AF398888.1_S.sclerotiorumisolateSS1	0	--	0
71.HPAB545908.1_Verticilliumnonalfalfaeisolate	0	--	0
72.EF433315.1_CeratocystisfimbriatavoucherCMW15052	522	--	522
73.MN159912.1_Botrytiscinerea	0	--	0
74.MF034741.1_PeltasterfructicolaisolateSRB92	0	--	0
75.LC440360.1_CercosporaasparagiCOasp2	0	--	0
76.AY944105.1_MagnaportheoryzaeisolateSAG00T3()	210	GACCTTTGGACTGTTGCTAACAACCTCACAGGAAGCCGCT	249
77.JX266586.1_CochliobolusmiyabeanusvoucherMFLUCC10-0733	0	--	0
78.MN393253.1_CorynesporacassiicolaisolateQHD001(MN393253.1UNVERIFIED)	0	--	0
79.MF375218.1_AgroatheliarolfsiiisolateBJB24	0	--	0
80.MN106270.1_AgroatheliarolfsiistrainJ-12	0	--	0
81.OQ732628.1_AgroatheliarolfsiiisolateBTCBSr3	0	--	0
82.KY196185.1_ColletotrichumtruncatumstrainPAK53	468	--	468
83.GU935835.1_ColletotrichumcoccodesisolateC96002	330	--	330
84.MK085963.1_AlternariatenuissimaisolateSCCZ06	0	--	0
85.MT548042.1_AlternarialongipesstrainKY_2019_012	0	--	0
86.MN356465.1CalonectriamontanaisolateHSP4	238	ATTGATTGACACTGTGCTAACTCACAAACAGGAAGCCGCT	277
87.OL694224.1_CalonectriacanadianastrainF099	240	ATTGATTGACACTGTGCTAACTCACAAACAGGAAGCCGCT	279
88.MK803351.1_NeoscytalidiumdimidiatumstrainKale4-C	0	--	0
89.ON376993.1_CurvulariachiangmaiensisisolateND00J7	0	--	0
90.OQ383346.1_NeoscytalidiumdimidiatumisolateGKH-2	0	--	0
91.MF662595.1_NeoscytalidiumnovaehollandiaeisolateNeNo1	0	--	0
92.EF560588.1Melampsoralini	380	--	380
93.LC590862.1_NeoscytalidiumdimidiatumPSU-HP01TEF1	0	--	0
94.KX278106.1_BotryosphaeriaqingyuanensisstrainCERC2947	0	--	0
95.AJ578763.1_Blumeriagraminisf.sp.hordeicyp51	0	--	0
96.MF490858.1_CurvulariadactylocteniicolastrainCPC28810	0	--	0
97.KT287115.1_Bipolariscactivoraisolate3.8.6	0	--	0
98.MT560940.1_CurvulariacactivorastrainHLGH0118	0	--	0
99.OM714565.1_CurvulariaplantarumstrainM0134	0	--	0
100.MN159911.1_BotrytiscinereaSICAUCC19-0003	0	--	0
102.GU294713.1LasiodiplodiatheobromaestrainUCD2430TX	0	--	0
103.KX868094.1_Mycosphaerellasp.isolateCRM20.1	212	TCAAGGACATGACACTGACAATTTGCTACAGGAAGCCGCC	251
104.LC599478.1Pseudocercosporapini-densifloraeMUCC534	0	--	0
105.N584698.1BipolarissetariaestrainKBS4-2	0	--	0
2.OM160859.1_F.buharicum	442	GAGCTCGGTAAGGGTTCCTTCAAGTACGCCTGGGTCCTTG	481
1.LC727524.1_F.buharicum_OKI-1_Okura	471	GAGCTCGGTAAGGGTTCCTTCAAGTACGCCTGGGTCCTTG	510
3.KX302919.1_F.sublunatum	454	-----------GGGTTCCTTCAAGTACGCTTGGGTTCTTG	482
4.LT996094.1_F.convolutans	453	-----------GGGTTCCTTCAAGTACGCCTGGGTTCTTG	481
5.OM160861.1_F.abutilonis	433	GAGCTCGGTAAGGGTTCCTTCAAGTACGCCTGGGTCCTTG	472
6.OM160874.1_F.guadeloupense	441	GAGCTCGGTAAGGGTTCCTTCAAGTACGCCTGGGTCCTTG	480
7.MH392475.1_F.graminearum	420	TCAAGTACGCC-----------------------------	430
8.MH582420.1_F.solani	461	GAGCTCGGTAAGGGTTCCTTCAAGTACGCCTGGGTCCTTG	500
9.MAFF244605_F.oxysporum	428	GAGCTCGGTAAGGGTTCCTTCAAGTACGCCTGGGTTCTTG	467
10.MAFF237278_F.contaminatum_Hylocereus	476	GAGCTCGGTAAGGGTTCCTTCAAGTACGCCTGGGTTCTTG	515

```
11.MAFF237649_F.concentricum__Riceroot                                457 GAGCTCGGTAAGGGTTCCTTCAAGTACGCCTGGGTTCTTG 496
12.MAFF237650_F.concentricum__Wheat                                   458 GAGCTCGGTAAGGGTTCCTTCAAGTACGCCTGGGTTCTTG 497
13.MAFF239869_F.mangiferae__Ryukyupine                                446 GAGCTCGGTAAGGGTTCCTTCAAGTACGCCTGGGTTCTTG 485
14.MAFF240460_F.fujikuroi_Passionfruit                                450 GAGCTCGGTAAGGGTTCCTTCAAGTACGCCTGGGTTCTTG 489
15.MAFF241317_F.graminearum_Wheat                                     456 GAGCTCGGTAAGGGTTCCTTCAAGTACGCCTGGGTTCTTG 495
16.MAFF242670_F.ipomoeae_Wheat                                        461 GAGCTCGGTAAGGGTTCCTTCAAGTACGCCTGGGTTCTTG 500
17.MAFF245129_F.concentricum_Fraxinus                                 448 GAGCTCGGTAAGGGTTCCTTCAAGTACGCCTGGGTTCTTG 487
18.MAFF245395_F.cugenangense_Rhubarb                                  458 GAGCTCGGTAAGGGTTCCTTCAAGTACGCCTGGGTTCTTG 497
19.MAFF246637_F.nirenbergiae_Strawberry                               460 GAGCTCGGTAAGGGTTCCTTCAAGTACGCCTGGGTTCTTG 499
20.MAFF246672_F.nirenbergiae_ChinesePeony                             460 GAGCTCGGTAAGGGTTCCTTCAAGTACGCCTGGGTTCTTG 499
21.MAFF246697_F.commune_Urallicoriceroot                              460 GAGCTCGGTAAGGGTTCCTTCAAGTACGCCTGGGTTCTTG 499
22.MAFF246729_F.falciforme_Angelica                                   482 GAGCTCGGTAAGGGTTCCTTCAAGTACGCCTGGGTCCTTG 521
23.MAFF247220_F.duplospermum__Euwallaceasp                            485 GAGCTCGGTAAGGGTTCCTTCAAGTACGCCTGGGTCCTTG 524
24.MAFF410760_F.odoratissimum_alpha                                   456 GAGCTCGGTAAGGGTTCCTTCAAGTACGCCTGGGTTCTTG 495
25.MAFF244605_FusariumoxysporumSchlechtendal_MAFF244605_Tomato         459 GAGCTCGGTAAGGGTTCCTTCAAGTACGCCTGGGTTCTTG 498
26.MAFF241326_F.asiaticum_Wheat                                       459 GAGCTCGGTAAGGGTTCCTTCAAGTACGCCTGGGTTCTTG 498
27.MAFF245014_F.asiaticum_Wildsoybean                                 456 GAGCTCGGTAAGGGTTCCTTCAAGTACGCCTGGGTTCTTG 495
28.MAFF150124_F.asiaticum__Wheat                                      456 GAGCTCGGTAAGGGCTCCTTCAAGTACGCCTGGGTTCTTG 495
29.OM135603.1F.algeriense                                             476 GAGCTCGGTAAGGGTTCCTTCAAGTACGCCTGGGTCCTTG 515
30.MAFF237465_F.penzigii_Aloe                                         487 -----------GGGTTCCTTCAAGTATGCCTGGGTTCTTG 515
31.MAFF103054_F.oxysporumSchlechtendalf.sp.cucumerinum_Cucumber       458 GAGCTCGGTAAGGGTTCCTTCAAGTACGCCTGGGTTCTTG 497
32.MAFF712246_F.oxysporumSchlechtendalf.sp.dianthi__Carnation         460 GAGCTCGGTAAGGGTTCCTTCAAGTACGCCTGGGTTCTTG 499
33.MAFF305558_F.oxysporumSchlechtendalf.sp.fragariae__Watermelon      458 GAGCTCGGTAAGGGTTCCTTCAAGTACGCCTGGGTTCTTG 497
34.MAFF744087_F.oxysporumSchlechtendalf.sp.lactucae__Lettuce          458 GAGCTCGGTAAGGGTTCCTTCAAGTACGCCTGGGTTCTTG 497
35.MAFF726924_F.oxysporumSchlechtendalf.sp.lagenariae_Whiteflberedgourd 458 GAGCTCGGTAAGGGTTCCTTCAAGTACGCCTGGGTTCTTG 497
36.MAFF744003_F.oxysporumSchlechtendalf.sp.lagenariae_Squash)         459 GAGCTCGGTAAGGGTTCCTTCAAGTACGCCTGGGTTCTTG 498
37.MAFF305122_F.oxysporumSchlechtendalf.sp.melonis__Melon             458 GAGCTCGGTAAGGGTTCCTTCAAGTACGCCTGGGTTCTTG 497
38.MAFF306714_F.oxysporumSchlechtendalf.sp.momordicae_Balsampear      458 GAGCTCGGTAAGGGTTCCTTCAAGTACGCCTGGGTTCTTG 497
39.MAFF238905_F.oxysporumSchlechtendalf.sp.radicis-lycopersici_Tomato 461 GAGCTCGGTAAGGGTTCCTTCAAGTACGCCTGGGTTCTTG 500
40.MAFF150004_F.oxysporumSchlechtendalf.sp.spinaciae_Spinach          458 GAGCTCGGTAAGGGTTCCTTCAAGTACGCCTGGGTTCTTG 497
41.MAFF247034_F.oxysporumSchlechtendal__Goldenchain                   458 GAGCTCGGTAAGGGTTCCTTCAAGTACGCCTGGGTTCTTG 497
42.MAFF245747_F.oxysporumSchlechtendalf.sp.callistephi__Chinaaster    458 GAGCTCGGTAAGGGTTCCTTCAAGTACGCCTGGGTTCTTG 497
43.MAFF305115_FoxysporumSchlechtendalf.sp.batatas__Sweatpotato        494 GAGCTCGGTAAGGGTTCCTTCAAGTACGCCTGGGTCCTTG 533
44.MAFF150126_F.asiaticum_Seed                                        456 GAGCTCGGTAAGGGTTCCTTCAAGTACGCCTGGGTTCTTG 495
45.MAFF246738_F.solani_Angelica                                       494 GAGCTCGGTAAGGGTTCCTTCAAGTACGCCTGGGTCCTTG 533
46.MAFF246664_F.cugenangense_Perilla                                  458 GAGCTCGGTAAGGGTTCCTTCAAGTACGCCTGGGTTCTTG 497
47.MH582420.1F.solanistrainMRC256                                     481 GAGCTCGGTAAGGGTTCCTTCAAGTACGCCTGGGTCCTTG 520
48.MAFF240361_F.babinda_Soil                                          477 GAGCTCGGTAAGGGTTCCTTCAAGTACGCTTGGGTTCTTG 516
49.MAFF242368_F.azukicola_Azukibean                                   498 GAGCTCGGCAAGGGTTCCTTCAAGTACGCCTGGGTCCTTG 537
50.MAFF241312_F.asiaticum_Soil,welshonionfield                        456 GAGCTCGGTAAGGGTTCCTTCAAGTACGCCTGGGTTCTTG 495
51.LT548416.1_F.culmorumpartialtef1a                                  449 GAGCTCGGTAAGGGTTCCTTCAAGTACGCCTGGGTTCTTG 488
52.MAFF150124_F.asiaticum__Wheat                                      456 GAGCTCGGTAAGGGCTCCTTCAAGTACGCCTGGGTTCTTG 495
53.MAFF238806_F.begoniae_Oncidiumsp                                   471 GAGCTCGGTAAGGGTTCCTTCAAGTATGCCTGGGTTCTTG 510
54.MW594399.1_FusariumincarnatumisolateUD01C                          470 GAGCTCGGTAAGGGTTCCTTCAAGTACGCCTGGGTTCTTG 509
55.OP414923.1Pucciniagraminisf.sp.triticiisolateSHZPgt19              285 ---------------------------------------- 285
56.MT027094.1_BipolarisoryzaestrainOrL-2                                0 ---------------------------------------- 0
```

```
57.ON734360.1_AlternariaalternataisolateH126                              0 ---------------------------------------- 0
58.LC333578.1_StemphyliumlycopersiciSOasp2                                0 ---------------------------------------- 0
59.HQ718583.1_Colletotrichumgloeosporioidesisolateq-1                   567 ---------------------------------------- 567
60.JN241603.1_AtheliarolfsiiisolateSR1                                   60 -----------------CTTCAA-TA-GCCTGGGTGCTCG 80
61.KJ866474.1_RhizoctoniasolanistrainMHL-1                                0 ---------------------------------------- 0
62.JQ672424.1AlternariatriticinaisolateEGS17-061                          0 ---------------------------------------- 0
63.LT707559.1_P.capsicipartialtef1agene                                   0 ---------------------------------------- 0
64.MW090051.1_CurvularialunatastrainCls-3                                 0 ---------------------------------------- 0
65.DQ400892.1_Aspergillusterreus                                          0 ---------------------------------------- 0
66.DQ911416.1_Pythiumsp.quercumstrainPy292                                0 ---------------------------------------- 0
67.EU797495.1_Phytophthorasp.oaksoilPoland                                0 ---------------------------------------- 0
68.HM148321.1_Cladosporiumcucumerinum                                   220 GAACTCGGCAAGGGTTCCTTCAAGTACGCATGGGTCCTCG 259
69.AF398888.1_SclerotiniasclerotiorumisolateSS1                           0 ---------------------------------------- 0
70.AF398888.1_S.sclerotiorumisolateSS1                                    0 ---------------------------------------- 0
71.HPAB545908.1_Verticilliumnonalfalfaeisolate                            0 ---------------------------------------- 0
72.EF433315.1_CeratocystisfimbriatavoucherCMW15052                      522 ---------------------------------------- 522
73.MN159912.1_Botrytiscinerea                                             0 ---------------------------------------- 0
74.MF034741.1_PeltasterfructicolaisolateSRB92                             0 ---------------------------------------- 0
75.LC440360.1_CercosporaasparagiCOasp2                                    0 ---------------------------------------- 0
76.AY944105.1_MagnaportheoryzaeisolateSAG00T3()                         250 GAGTTG---------------------------------- 255
77.JX266586.1_CochliobolusmiyabeanusvoucherMFLUCC10-0733                  0 ---------------------------------------- 0
78.MN393253.1_CorynesporacassiicolaisolateQHD001(MN393253.1UNVERIFIED)    0 ---------------------------------------- 0
79.MF375218.1_AgroatheliarolfsiiisolateBJB24                              0 ---------------------------------------- 0
80.MN106270.1_AgroatheliarolfsiistrainJ-12                                0 ---------------------------------------- 0
81.OQ732628.1_AgroatheliarolfsiiisolateBTCBSr3                            0 ---------------------------------------- 0
82.KY196185.1_ColletotrichumtruncatumstrainPAK53                        468 ---------------------------------------- 468
83.GU935835.1_ColletotrichumcoccodesisolateC96002                       331 -----------------CTTCAAGTACGCGTGGGTTCTTG 353
84.MK085963.1_AlternariatenuissimaisolateSCCZ06                           0 ---------------------------------------- 0
85.MT548042.1_AlternarialongipesstrainKY_2019_012                         0 ---------------------------------------- 0
86.MN356465.1CalonectriamontanaisolateHSP4                              278 GAACTCGGCAAGGGTTCCTTCAAGTACGCCTGGGTTCTTG 317
87.OL694224.1_CalonectriacanadianastrainF099                            280 GAACTCGGCAAGGGTTCCTTCAAGTACGCCTGGGTTCTTG 319
88.MK803351.1_NeoscytalidiumdimidiatumstrainKale4-C                       0 ---------------------------------------- 0
89.ON376993.1_CurvulariachiangmaiensisisolateND00J7                       0 ---------------------------------------- 0
90.OQ383346.1_NeoscytalidiumdimidiatumisolateGKH-2                        0 ---------------------------------------- 0
91.MF662595.1_NeoscytalidiumnovaehollandiaeisolateNeNo1                   0 ---------------------------------------- 0
92.EF560588.1Melampsoralini                                             380 ---------------------------------------- 380
93.LC590862.1_NeoscytalidiumdimidiatumPSU-HP01TEF1                        0 ---------------------------------------- 0
94.KX278106.1_BotryosphaeriaqingyuanensisstrainCERC2947                   0 ---------------------------------------- 0
95.AJ578763.1_Blumeriagraminisf.sp.hordeicyp51                            0 ---------------------------------------- 0
96.MF490858.1_CurvulariadactylocteniicolastrainCPC28810                   0 ---------------------------------------- 0
97.KT287115.1_Bipolariscactivoraisolate3.8.6                              0 ---------------------------------------- 0
98.MT560940.1_CurvulariacactivorastrainHLGH0118                           0 ---------------------------------------- 0
99.OM714565.1_CurvulariaplantarumstrainM0134                              0 ---------------------------------------- 0
100.MN159911.1_BotrytiscinereaSICAUCC19-0003                              0 ---------------------------------------- 0
102.GU294713.1LasiodiplodiatheobromaestrainUCD2430TX                      0 ---------------------------------------- 0
103.KX868094.1_Mycosphaerellasp.isolateCRM20.1                          252 GAGTTGGGCAAGGGCTCCTTCAAGTACGCATGGGTGCTCG 291
```

```
104.LC599478.1Pseudocercosporapini-densifloraeMUCC534                        0 ---------------------------------------- 0
105.N584698.1BipolarissetariaestrainKBS4-2                                   0 ---------------------------------------- 0

2.OM160859.1_F.buharicum                                                   482 ACAAGCTCAAGGCCGAGCGTGAGCGTGGTATCACCATCGA 521
1.LC727524.1_F.buharicum_OKI-1_Okura                                       511 ACAAGCTCAAGGCCGAGCGTGAGCGTGGTATCACCATCGA 550
3.KX302919.1_F.sublunatum                                                  483 ACAAGCTCAAAGCCGAGCGTGAGCGTGGTATCACCATCG- 521
4.LT996094.1_F.convolutans                                                 482 ACAAGCTCAAGGCCGAGCGTGAGCGTGGTATCACC----- 516
5.OM160861.1_F.abutilonis                                                  473 ACAAGCTCAAGGCCGAGCGTGAGCGTGGTATCACCATCGA 512
6.OM160874.1_F.guadeloupense                                               481 ACAAGCTCAAGGCCGAGCGTGAGCGTGGTATCACCATCGA 520
7.MH392475.1_F.graminearum                                                 430 ---------------------------------------- 430
8.MH582420.1_F.solani                                                      501 ACAAGCTCAAGGCCGAGCGTGAGCGTGGTATCACCATCGA 540
9.MAFF244605_F.oxysporum                                                   468 ACAAGCTCAAGGCCGAGCGTGAGCGTGGTATCACCATCGA 507
10.MAFF237278_F.contaminatum_Hylocereus                                    516 ACAAGCTCAAGGCCGAGCGTGAGCGTGGTATCACCATCGA 555
11.MAFF237649_F.concentricum__Riceroot                                     497 ACAAGCTCAAGGCCGAGCGTGAGCGTGGTATCACCATCGA 536
12.MAFF237650_F.concentricum__Wheat                                        498 ACAAGCTCAAGGCCGAGCGTGAGCGTGGTATCACCATCGA 537
13.MAFF239869_F.mangiferae__Ryukyupine                                     486 ACAAGCTCAAGGCCGAGCGTGAGCGTGGTATCACCATCGA 525
14.MAFF240460_F.fujikuroi_Passionfruit                                     490 ACAAGCTCAAGGCCGAGCGTGAGCGTGGTATCACCATCGA 529
15.MAFF241317_F.graminearum_Wheat                                          496 ACAAGCTCAAAGCCGAGCGTGAGCGTGGTATCACCATTGA 535
16.MAFF242670_F.ipomoeae_Wheat                                             501 ACAAGCTCAAGGCCGAGCGTGAGCGTGGTATCACCATCGA 540
17.MAFF245129_F.concentricum_Fraxinus                                      488 ACAAGCTCAAGGCCGAGCGTGAGCGTGGTATCACCATCGA 527
18.MAFF245395_F.cugenangense_Rhubarb                                       498 ACAAGCTCAAGGCCGAGCGTGAGCGTGGTATCACCATCGA 537
19.MAFF246637_F.nirenbergiae_Strawberry                                    500 ACAAGCTCAAGGCCGAGCGTGAGCGTGGTATCACCATCGA 539
20.MAFF246672_F.nirenbergiae_ChinesePeony                                  500 ACAAGCTCAAGGCCGAGCGTGAGCGTGGTATCACCATCGA 539
21.MAFF246697_F.commune_Urallicoriceroot                                   500 ACAAGCTCAAGGCCGAGCGTGAGCGTGGTATCACCATCGA 539
22.MAFF246729_F.falciforme_Angelica                                        522 ACAAGCTCAAGGCCGAGCGTGAGCGTGGTATCACCATCGA 561
23.MAFF247220_F.duplospermum__Euwallaceasp                                 525 ACAAGCTCAAGGCCGAGCGTGAGCGTGGTATCACCATCGA 564
24.MAFF410760_F.odoratissimum_alpha                                        496 ACAAGCTCAAGGCCGAGCGTGAGCGTGGTATCACCATCGA 535
25.MAFF244605_FusariumoxysporumSchlechtendal_MAFF244605_Tomato              499 ACAAGCTCAAGGCCGAGCGTGAGCGTGGTATCACCATCGA 538
26.MAFF241326_F.asiaticum_Wheat                                            499 ACAAGCTCAAAGCCGAGCGTGAGCGTGGTATCACCATTGA 538
27.MAFF245014_F.asiaticum_Wildsoybean                                      496 ACAAGCTCAAAGCCGAGCGTGAGCGTGGTATCACCATTGA 535
28.MAFF150124_F.asiaticum__Wheat                                           496 ACAAGCTCAAAGCCGAGCGTGAGCGTGGTATCACCATTGA 535
29.OM135603.1F.algeriense                                                  516 ACAAGCTCAAGGCCGAGCGTGAGCGTGGTATCACCATCGA 555
30.MAFF237465_F.penzigii_Aloe                                              516 ACAAGCTCAAGGCCGAGCGTGAGCGTGGTATCACCATCGA 555
31.MAFF103054_F.oxysporumSchlechtendalf.sp.cucumerinum_Cucumber            498 ACAAGCTCAAGGCCGAGCGTGAGCGTGGTATCACCATCGA 537
32.MAFF712246_F.oxysporumSchlechtendalf.sp.dianthi__Carnation              500 ACAAGCTCAAGGCCGAGCGTGAGCGTGGTATCACCATCGA 539
33.MAFF305558_F.oxysporumSchlechtendalf.sp.fragariae__Watermelon           498 ACAAGCTCAAGGCCGAGCGTGAGCGTGGTATCACCATCGA 537
34.MAFF744087_F.oxysporumSchlechtendalf.sp.lactucae__Lettuce               498 ACAAGCTCAAGGCCGAGCGTGAGCGTGGTATCACCATCGA 537
35.MAFF726924_F.oxysporumSchlechtendalf.sp.lagenariae_Whitefloweredgourd   498 ACAAGCTCAAGGCCGAGCGTGAGCGTGGTATCACCATCGA 537
36.MAFF744003_F.oxysporumSchlechtendalf.sp.lagenariae_Squash)              499 ACAAGCTCAAGGCCGAGCGTGAGCGTGGTATCACCATCGA 538
37.MAFF305122_F.oxysporumSchlechtendalf.sp.melonis__Melon                  498 ACAAGCTCAAGGCCGAGCGTGAGCGTGGTATCACCATCGA 537
38.MAFF306714_F.oxysporumSchlechtendalf.sp.momordicae_Balsampear           498 ACAAGCTCAAGGCCGAGCGTGAGCGTGGTATCACCATCGA 537
39.MAFF238905_F.oxysporumSchlechtendalf.sp.radicis-lycopersici_Tomato      501 ACAAGCTCAAGGCCGAGCGTGAGCGTGGTATCACCATCGA 540
40.MAFF150004_F.oxysporumSchlechtendalf.sp.spinaciae_Spinach               498 ACAAGCTCAAGGCCGAGCGTGAGCGTGGTATCACCATCGA 537
41.MAFF247034_F.oxysporumSchlechtendal__Goldenchain                        498 ACAAGCTCAAGGCCGAGCGTGAGCGTGGTATCACCATCGA 537
42.MAFF245747_F.oxysporumSchlechtendalf.sp.callistephi__Chinaaster         498 ACAAGCTCAAGGCCGAGCGTGAGCGTGGTATCACCATCGA 537
43.MAFF305115_FoxysporumSchlechtendalf.sp.batatas__Sweetpotato             534 ACAAGCTCAAGGCCGAGCGTGAGCGTGGTATCACCATCGA 573
```

```
44.MAFF150126_F.asiaticum_Seed                                         496 ACAAGCTCAAAGCCGAGCGTGAGCGTGGTATCACCATTGA 535
45.MAFF246738_F.solani_Angelica                                        534 ACAAGCTCAAGGCCGAGCGTGAGCGTGGTATCACCATCGA 573
46.MAFF246664_F.cugenangense_Perilla                                   498 ACAAGCTCAAGGCCGAGCGTGAGCGTGGTATCACCATCGA 537
47.MH582420.1F.solanistrainMRC256                                      521 ACAAGCTCAAGGCCGAGCGTGAGCGTGGTATCACCATCGA 560
48.MAFF240361_F.babinda_Soil                                           517 ACAAGCTCAAAGCCGAGCGTGAGCGTGGTATCACCATCGA 556
49.MAFF242368_F.azukicola_Azukibean                                    538 ACAAGCTCAAGGCCGAGCGTGAGCGTGGTATCACCATCGA 577
50.MAFF241312_F.asiaticum_Soil,welshonionfield                         496 ACAAGCTCAAAGCCGAGCGTGAGCGTGGTATCACCATTGA 535
51.LT548416.1_F.culmorumpartialtef1a                                   489 ACAAGCTCAAAGCCGAGCGTGAGCGTGGTATCACCATTGA 528
52.MAFF150124_F.asiaticum__Wheat                                       496 ACAAGCTCAAAGCCGAGCGTGAGCGTGGTATCACCATTGA 535
53.MAFF238806_F.begoniae_Oncidiumsp                                    511 ACAAGCTCAAGGCCGAGCGTGAGCGTGGTATCACCATCGA 550
54.MW594399.1_FusariumincarnatumisolateUD01C                           510 ACAAGCTCAAGGCTGAGCGTGAGCGTGGTATCACCATCGA 549
55.OP414923.1Pucciniagraminisf.sp.triticiisolateSHZPgt19               285 ---------------------------------------- 285
56.MT027094.1_BipolarisoryzaestrainOrL-2                                 0 ---------------------------------------- 0
57.ON734360.1_AlternariaalternataisolateH126                             0 ---------------------------------------- 0
58.LC333578.1_StemphyliumlycopersiciSOasp2                               0 ---------------------------------------- 0
59.HQ718583.1_Colletotrichumgloeosporioidesisolateq-1                  567 ---------------------------------------- 567
60.JN241603.1_AtheliarolfsiiisolateSR1                                  81 ACAAACTGAAGGCCGAGCGTGAGCGTGGTATCACCATTGA 120
61.KJ866474.1_RhizoctoniasolanistrainMHL-1                               0 ---------------------------------------- 0
62.JQ672424.1AlternariatriticinaisolateEGS17-061                         1 -------------CGAGCGTGAGCGTGGTATCACCATCGA 27
63.LT707559.1_P.capsicipartialtef1agene                                  1 -----------------------------CCCACGATCGA 11
64.MW090051.1_CurvularialunatastrainCls-3                                0 ---------------------------------------- 0
65.DQ400892.1_Aspergillusterreus                                         0 ---------------------------------------- 0
66.DQ911416.1_Pythiumsp.quercumstrainPy292                               0 ---------------------------------------- 0
67.EU797495.1_Phytophthorasp.oaksoilPoland                               0 ---------------------------------------- 0
68.HM148321.1_Cladosporiumcucumerinum                                  260 ACAAGCTGAAGTCCGAGCGTGAGCGTGGTATCACCATCGA 299
69.AF398888.1_SclerotiniasclerotiorumisolateSS1                          0 ---------------------------------------- 0
70.AF398888.1_S.sclerotiorumisolateSS1                                   0 ---------------------------------------- 0
71.HPAB545908.1_Verticilliumnonalfalfaeisolate                           0 ---------------------------------------- 0
72.EF433315.1_CeratocystisfimbriatavoucherCMW15052                     522 ---------------------------------------- 522
73.MN159912.1_Botrytiscinerea                                            0 ---------------------------------------- 0
74.MF034741.1_PeltasterfructicolaisolateSRB92                            0 ---------------------------------------- 0
75.LC440360.1_CercosporaasparagiCOasp2                                   0 ---------------------------------------- 0
76.AY944105.1_MagnaportheoryzaeisolateSAG00T3()                        255 ---------------------------------------- 255
77.JX266586.1_CochliobolusmiyabeanusvoucherMFLUCC10-0733                 0 ---------------------------------------- 0
78.MN393253.1_CorynesporacassiicolaisolateQHD001(MN393253.1UNVERIFIED)   0 ---------------------------------------- 0
79.MF375218.1_AgroatheliarolfsiiisolateBJB24                             0 ---------------------------------------- 0
80.MN106270.1_AgroatheliarolfsiistrainJ-12                               0 ---------------------------------------- 0
81.OQ732628.1_AgroatheliarolfsiiisolateBTCBSr3                           0 ---------------------------------------- 0
82.KY196185.1_ColletotrichumtruncatumstrainPAK53                       468 ---------------------------------------- 468
83.GU935835.1_ColletotrichumcoccodesisolateC96002                      354 ACAAGCTCAAGGCCGAGCGTGAGCGTGGTATCACCATCGA 393
84.MK085963.1_AlternariatenuissimaisolateSCCZ06                          0 ---------------------------------------- 0
85.MT548042.1_AlternarialongipesstrainKY_2019_012                        0 ---------------------------------------- 0
86.MN356465.1CalonectriamontanaisolateHSP4                             318 ACAAGCTCAAGGCCGAGCGTGAGCGTGGTATCACCATCGA 357
87.OL694224.1_CalonectriacanadianastrainF099                           320 ACAAGCTCAAGGCCGAGCGTGAGCGTGGTATCACCATCGA 359
88.MK803351.1_NeoscytalidiumdimidiatumstrainKale4-C                      0 ---------------------------------------- 0
89.ON376993.1_CurvulariachiangmaiensisisolateND00J7                      0 ---------------------------------------- 0
```

```
90.OQ383346.1_NeoscytalidiumdimidiatumisolateGKH-2                0 ---------------------------------------- 0
91.MF662595.1_NeoscytalidiumnovaehollandiaeisolateNeNo1           0 ---------------------------------------- 0
92.EF560588.1Melampsoralini                                     380 ---------------------------------------- 380
93.LC590862.1_NeoscytalidiumdimidiatumPSU-HP01TEF1                0 ---------------------------------------- 0
94.KX278106.1_BotryosphaeriaqingyuanensisstrainCERC2947           0 ---------------------------------------- 0
95.AJ578763.1_Blumeriagraminisf.sp.hordeicyp51                    0 ---------------------------------------- 0
96.MF490858.1_CurvulariadactylocteniicolastrainCPC28810           0 ---------------------------------------- 0
97.KT287115.1_Bipolariscactivoraisolate3.8.6                      0 ---------------------------------------- 0
98.MT560940.1_CurvulariacactivorastrainHLGH0118                   0 ---------------------------------------- 0
99.OM714565.1_CurvulariaplantarumstrainM0134                      0 ---------------------------------------- 0
100.MN159911.1_BotrytiscinereaSICAUCC19-0003                      0 ---------------------------------------- 0
102.GU294713.1LasiodiplodiatheobromaestrainUCD2430TX              0 ---------------------------------------- 0
103.KX868094.1_Mycosphaerellasp.isolateCRM20.1                  292 ACAAGCTGAAGGCCGAGCGTGAGCGTGGTATCACTATCGA 331
104.LC599478.1Pseudocercosporapini-densifloraeMUCC534             0 ---------------------------------------- 0
105.N584698.1BipolarissetariaestrainKBS4-2                        0 ---------------------------------------- 0

2.OM160859.1_F.buharicum                                        522 CATTGCTCTCTGGAAGTTCGAGACTCCTCGCTACTATGT- 560
1.LC727524.1_F.buharicum_OKI-1_Okura                            551 CATTGCTCTCTGGAAGTTCGAGACTCCTCGCTACTATGTC 590
3.KX302919.1_F.sublunatum                                       521 ---------------------------------------- 521
4.LT996094.1_F.convolutans                                      516 ---------------------------------------- 516
5.OM160861.1_F.abutilonis                                       513 CATTGCTCTCTGGAAGTTCGAGACTCCCCGCTACTATGTC 552
6.OM160874.1_F.guadeloupense                                    521 CATTGCTCTCTGGAAGTTCGAGACTCCCCGCTACTATGTC 560
7.MH392475.1_F.graminearum                                      430 ---------------------------------------- 430
8.MH582420.1_F.solani                                           540 ---------------------------------------- 540
9.MAFF244605_F.oxysporum                                        508 TATTGCTCTCTGGAAGTTCGAGACTCCTCGCTACTAT--- 544
10.MAFF237278_F.contaminatum_Hylocereus                         556 TATTGCTCTCTGGAAGTTCGAGACTCCTCGCTACTATGTC 595
11.MAFF237649_F.concentricum__Riceroot                          537 TATCGCTCTCTGGAAGTTCGAGACTCCTCGCTACTATGTC 576
12.MAFF237650_F.concentricum__Wheat                             538 TATCGCTCTCTGGAAGTTCGAGACTCCTCGCTACTATGTC 577
13.MAFF239869_F.mangiferae__Ryukyupine                          526 TATTGCTCTCTGGAAGTTCGAGACTCCTCGCTACTATGTC 565
14.MAFF240460_F.fujikuroi_Passionfruit                          530 TATTGCTCTCTGGAAGTTCGAGACTCCTCGCTACTATGTC 569
15.MAFF241317_F.graminearum_Wheat                               536 TATCGCCCTCTGGAAGTTCGAGACTCCTCGCTACTATGTC 575
16.MAFF242670_F.ipomoeae_Wheat                                  541 TATCGCTCTCTGGAAGTTCGAGACTCCTCGCTACTATGTC 580
17.MAFF245129_F.concentricum_Fraxinus                           528 TATCGCTCTCTGGAAGTTCGAGACTCCTCGCTACTATGTC 567
18.MAFF245395_F.cugenangense_Rhubarb                            538 TATTGCTCTCTGGAAGTTCGAGACTCCTCGCTACTATGTC 577
19.MAFF246637_F.nirenbergiae_Strawberry                         540 TATTGCTCTCTGGAAGTTCGAGACTCCTCGCTACTATGTC 579
20.MAFF246672_F.nirenbergiae_ChinesePeony                       540 TATTGCTCTCTGGAAGTTCGAGACTCCTCGCTACTATGTC 579
21.MAFF246697_F.commune_Urallicoriceroot                        540 TATTGCTCTCTGGAAGTTCGAGACTCCTCGCTACTATGTC 579
22.MAFF246729_F.falciforme_Angelica                             562 CATTGCCCTCTGGAAGTTCGAGACTCCCCGCTACTATGTC 601
23.MAFF247220_F.duplospermum__Euwallaceasp                      565 CATTGCCCTCTGGAAGTTCGAGACTCCCCGCTACTATGTC 604
24.MAFF410760_F.odoratissimum_alpha                             536 TATTGCTCTCTGGAAGTTCGAGACTCCTCGCTACTATGTC 575
25.MAFF244605_FusariumoxysporumSchlechtendal_MAFF244605_Tomato  539 TATTGCTCTCTGGAAGTTCGAGACTCCTCGCTACTATGTC 578
26.MAFF241326_F.asiaticum_Wheat                                 539 TATCGCCCTCTGGAAGTTCGAGACTCCTCGCTACTATGTC 578
27.MAFF245014_F.asiaticum_Wildsoybean                           536 TATCGCCCTCTGGAAGTTCGAGACTCCTCGCTACTATGTC 575
28.MAFF150124_F.asiaticum__Wheat                                536 TATCGCCCTCTGGAAGTTCGAGACTCCTCGCTACTATGTC 575
29.OM135603.1F.algeriense                                       556 CATTGCTCTCTGGAAGTTCGAGACTCCCCGTTACTATGTC 595
30.MAFF237465_F.penzigii_Aloe                                   556 TATCGCTCTCTGGAAGTTCGAGACTCCCCGCTACTATGTC 595
```

31.MAFF103054_F.oxysporumSchlechtendalf.sp.cucumerinum_Cucumber	538	TATTGCTCTCTGGAAGTTCGAGACTCCTCGCTACTATGTC	577
32.MAFF712246_F.oxysporumSchlechtendalf.sp.dianthi__Carnation	540	TATTGCTCTCTGGAAGTTCGAGACTCCTCGCTACTATGTC	579
33.MAFF305558_F.oxysporumSchlechtendalf.sp.fragariae__Watermelon	538	TATTGCTCTCTGGAAGTTCGAGACTCCTCGCTACTATGTC	577
34.MAFF744087_F.oxysporumSchlechtendalf.sp.lactucae__Lettuce	538	TATTGCTCTCTGGAAGTTCGAGACTCCTCGCTACTATGTC	577
35.MAFF726924_F.oxysporumSchlechtendalf.sp.lagenariae_Whitefloweredgourd	538	TATTGCTCTCTGGAAGTTCGAGACTCCTCGCTACTATGTC	577
36.MAFF744003_F.oxysporumSchlechtendalf.sp.lagenariae_Squash)	539	TATTGCTCTCTGGAAGTTCGAGACTCCTCGCTACTATGTC	578
37.MAFF305122_F.oxysporumSchlechtendalf.sp.melonis__Melon	538	TATTGCTCTCTGGAAGTTCGAGACTCCTCGCTACTATGTC	577
38.MAFF306714_F.oxysporumSchlechtendalf.sp.momordicae_Balsampear	538	TATTGCTCTCTGGAAGTTCGAGACTCCTCGCTACTATGTC	577
39.MAFF238905_F.oxysporumSchlechtendalf.sp.radicis-lycopersici_Tomato	541	TATTGCTCTCTGGAAGTTCGAGACTCCTCGCTACTATGTC	580
40.MAFF150004_F.oxysporumSchlechtendalf.sp.spinaciae_Spinach	538	TATTGCTCTCTGGAAGTTCGAGACTCCTCGCTACTATGTC	577
41.MAFF247034_F.oxysporumSchlechtendal__Goldenchain	538	TATTGCTCTCTGGAAGTTCGAGACTCCTCGCTACTATGTC	577
42.MAFF245747_F.oxysporumSchlechtendalf.sp.callistephi__Chinaaster	538	TATTGCTCTCTGGAAGTTCGAGACTCCTCGCTACTATGTC	577
43.MAFF305115_FoxysporumSchlechtendalf.sp.batatas__Sweatpotato	574	CATTGCCCTCTGGAAGTTCGAGACTCCCCGCTACTATGTC	613
44.MAFF150126_F.asiaticum_Seed	536	TATCGCCCTCTGGAAGTTCGAGACTCCTCGCTACTATGTC	575
45.MAFF246738_F.solani_Angelica	574	CATTGCCCTCTGGAAGTTCGAGACTCCCCGCTACTATGTC	613
46.MAFF246664_F.cugenangense_Perilla	538	TATTGCTCTCTGGAAGTTCGAGACTCCTCGCTACTATGTC	577
47.MH582420.1F.solanistrainMRC256	561	CATTGCCCTCTGGAAGTTCGAGACTCCCCGCTACTATGTC	600
48.MAFF240361_F.babinda_Soil	557	TATTGCTCTCTGGAAGTTCGAGACTCCTCGCTACTATGTC	596
49.MAFF242368_F.azukicola_Azukibean	578	CATTGCCCTCTGGAAGTTCGAGACTCCCCGCTACTATGTC	617
50.MAFF241312_F.asiaticum_Soil,welshonionfield	536	TATCGCCCTCTGGAAGTTCGAGACTCCTCGCTACTATGTC	575
51.LT548416.1_F.culmorumpartialtef1a	529	TATCGCTCTCTGGAAGTTCGAGACTCCTCGCTACTATGTC	568
52.MAFF150124_F.asiaticum__Wheat	536	TATCGCCCTCTGGAAGTTCGAGACTCCTCGCTACTATGTC	575
53.MAFF238806_F.begoniae_Oncidiumsp	551	TATTGCTCTCTGGAAGTTCGAGACTCCTCGCTACTATGTC	590
54.MW594399.1_FusariumincarnatumisolateUD01C	550	TATCGCCCTCTGGAAGTTCGAGACTCCTCGCTACTATGTC	589
55.OP414923.1Pucciniagraminisf.sp.triticiisolateSHZPgt19	285	--	285
56.MT027094.1_BipolarisoryzaestrainOrL-2	0	--	0
57.ON734360.1_AlternariaalternataisolateH126	0	--	0
58.LC333578.1_StemphyliumlycopersiciSOasp2	0	--	0
59.HQ718583.1_Colletotrichumgloeosporioidesisolateq-1	567	--	567
60.JN241603.1_AtheliarolfsiiisolateSR1	121	TATCGCTCTCTGGAAGTTCGAGACCCCCAAATACATGGTT	160
61.KJ866474.1_RhizoctoniasolanistrainMHL-1	0	--	0
62.JQ672424.1AlternariatriticinaisolateEGS17-061	28	TATCGCTCTCTGGAAGTTCGAGACTCCCAAGTACGTATTC	67
63.LT707559.1_P.capsicipartialtef1agene	12	CATTGCCCTGTGGAAGTTCGAGTCCCCCAAGTACTTCTTC	51
64.MW090051.1_CurvularialunatastrainCls-3	0	--	0
65.DQ400892.1_Aspergillusterreus	0	--	0
66.DQ911416.1_Pythiumsp.quercumstrainPy292	1	----------TGGAAGTTCGAGTCGCCGAAGTACTTCTTC	30
67.EU797495.1_Phytophthorasp.oaksoilPoland	1	----------TGGAAGTTCGAGTCCCCCAAGTACTTCTTC	30
68.HM148321.1_Cladosporiumcucumerinum	300	TATCGCCCTCTGGAAGTTCGAGACTCCCAAGTACAACGTC	339
69.AF398888.1_SclerotiniasclerotiorumisolateSS1	0	--	0
70.AF398888.1_S.sclerotiorumisolateSS1	0	--	0
71.HPAB545908.1_Verticilliumnonalfalfaeisolate	0	--	0
72.EF433315.1_CeratocystisfimbriatavoucherCMW15052	522	--	522
73.MN159912.1_Botrytiscinerea	0	--	0
74.MF034741.1_PeltasterfructicolaisolateSRB92	0	--	0
75.LC440360.1_CercosporaasparagiCOasp2	0	--	0
76.AY944105.1_MagnaportheoryzaeisolateSAG00T3()	255	--	255

77.JX266586.1_CochliobolusmiyabeanusvoucherMFLUCC10-0733	0	--	0
78.MN393253.1_CorynesporacassiicolaisolateQHD001(MN393253.1UNVERIFIED)	0	--	0
79.MF375218.1_AgroatheliarolfsiiisolateBJB24	0	--	0
80.MN106270.1_AgroatheliarolfsiistrainJ-12	0	--	0
81.OQ732628.1_AgroatheliarolfsiiisolateBTCBSr3	0	--	0
82.KY196185.1_ColletotrichumtruncatumstrainPAK53	468	--	468
83.GU935835.1_ColletotrichumcoccodesisolateC96002	394	CATTGCCCTCTGGAAGTTCGAGACTCCCAAGTACTATGTC	433
84.MK085963.1_AlternariatenuissimaisolateSCCZ06	0	--	0
85.MT548042.1_AlternarialongipesstrainKY_2019_012	0	--	0
86.MN356465.1CalonectriamontanaisolateHSP4	358	TATTGCTCTCTGGAAGTTCGAGACTCCCAAGTACGATGTC	397
87.OL694224.1_CalonectriacanadianastrainF099	360	TATTGCTCTCTGGAAGTTCGAGACTCCCAAGTACGATGTC	399
88.MK803351.1_NeoscytalidiumdimidiatumstrainKale4-C	0	--	0
89.ON376993.1_CurvulariachiangmaiensisisolateND00J7	0	--	0
90.OQ383346.1_NeoscytalidiumdimidiatumisolateGKH-2	0	--	0
91.MF662595.1_NeoscytalidiumnovaehollandiaeisolateNeNo1	0	--	0
92.EF560588.1Melampsoralini	380	--	380
93.LC590862.1_NeoscytalidiumdimidiatumPSU-HP01TEF1	0	--	0
94.KX278106.1_BotryosphaeriaqingyuanensisstrainCERC2947	0	--	0
95.AJ578763.1_Blumeriagraminisf.sp.hordeicyp51	0	--	0
96.MF490858.1_CurvulariadactylocteniicolastrainCPC28810	0	--	0
97.KT287115.1_Bipolariscactivoraisolate3.8.6	0	--	0
98.MT560940.1_CurvulariacactivorastrainHLGH0118	0	--	0
99.OM714565.1_CurvulariaplantarumstrainM0134	0	--	0
100.MN159911.1_BotrytiscinereaSICAUCC19-0003	0	--	0
102.GU294713.1LasiodiplodiatheobromaestrainUCD2430TX	0	--	0
103.KX868094.1_Mycosphaerellasp.isolateCRM20.1	332	TATCGCACTCTGGAAGTTCGAGACTCCAAAGTACTACGT-	370
104.LC599478.1Pseudocercosporapini-densifloraeMUCC534	0	--	0
105.N584698.1BipolarissetariaestrainKBS4-2	0	--	0

2.OM160859.1_F.buharicum	560	--	560
1.LC727524.1_F.buharicum_OKI-1_Okura	591	ACCGTCATTGGTATGTTGTCAC----CTTCA-GCTCATCT	625
3.KX302919.1_F.sublunatum	521	--	521
4.LT996094.1_F.convolutans	516	--	516
5.OM160861.1_F.abutilonis	553	ACCGTCATTGGTATGTCATCGC----CTTCA-CCTCACCG	587
6.OM160874.1_F.guadeloupense	560	--	560
7.MH392475.1_F.graminearum	430	--	430
8.MH582420.1_F.solani	540	--	540
9.MAFF244605_F.oxysporum	544	--	544
10.MAFF237278_F.contaminatum_Hylocereus	596	ACCGTCATTGGTATGTTGTCGCTC--ATGCT-TCATTCTA	632
11.MAFF237649_F.concentricum__Riceroot	577	ACCGTCATTGGTATGTTGTCGCCC--ATGCT-TCATTCTT	613
12.MAFF237650_F.concentricum__Wheat	578	ACCGTCATTGGTATGTTGTCGCCC--ATGCT-ACATTCTT	614
13.MAFF239869_F.mangiferae__Ryukyupine	566	ACCGTCATTGGTATGTTGTCGCTC--ATGCT-TCATTCTT	602
14.MAFF240460_F.fujikuroi_Passionfruit	570	ACCGTCATTGGTATGTTGTCGCTC--ATGCT-TCATTCTA	606
15.MAFF241317_F.graminearum_Wheat	576	ACCGTCATTGGTATGTTGTCAC-----CACT-GCTGTCAT	609
16.MAFF242670_F.ipomoeae_Wheat	581	ACCGTCATTGGTATGTTGTCAC-----CACTTGCACTCAT	615
17.MAFF245129_F.concentricum_Fraxinus	568	ACCGTCATTGGTATGTTGTCGCCC--ATGCT-ACATTCTT	604

```
18.MAFF245395_F.cugenangense_Rhubarb                                   578 ACCGTCATTGGTATGTTGTCGCTC--ATGCT-TCATTCTA 614
19.MAFF246637_F.nirenbergiae_Strawberry                                580 ACCGTCATTGGTATGTTGTCGCTC--ATGCT-TCATTCTA 616
20.MAFF246672_F.nirenbergiae_ChinesePeony                              580 ACCGTCATTGGTATGTTGTCGCTC--ATGCT-TCATTCTA 616
21.MAFF246697_F.commune_Urallicoriceroot                               580 ACCGTCATTGGTATGTTGTCGCTC--ATGCT-TCATTCTA 616
22.MAFF246729_F.falciforme_Angelica                                    602 ACCGTCATTGGTATGTTGCTGTCA----CCTCTG-TCACA 636
23.MAFF247220_F.duplospermum__Euwallaceasp                             605 ACCGTCATTGGTATGTCGCTGTCG----TCTCTC-TCAAT 639
24.MAFF410760_F.odoratissimum_alpha                                    576 ACCGTCATTGGTATGTTGTCGCTC--ATGCT-TCATTCTA 612
25.MAFF244605_FusariumoxysporumSchlechtendal_MAFF244605_Tomato         579 ACCGTCATTGGTATGTTGTCGCTC--ATGCT-TCATTCTA 615
26.MAFF241326_F.asiaticum_Wheat                                        579 ACCGTCATTGGTATGTTGTCAC-----CACT-GCTGTCAT 612
27.MAFF245014_F.asiaticum_Wildsoybean                                  576 ACCGTCATTGGTATGTTGTCAC-----CACT-GCTGTCAT 609
28.MAFF150124_F.asiaticum__Wheat                                       576 ACCGTCATTGGTATGTTGTCAC-----CACT-GCTGTCAT 609
29.OM135603.1F.algeriense                                              596 ACCGTCATTGGTATGTTGTCACTCTGATGCTCGATTTATA 635
30.MAFF237465_F.penzigii_Aloe                                          596 ACCGTCATTGGTAAGTCTTGACTGACTCACGCACGTCATC 635
31.MAFF103054_F.oxysporumSchlechtendalf.sp.cucumerinum_Cucumber        578 ACCGTCATTGGTATGTTGTCGCTC--ATGCT-TCATTCTA 614
32.MAFF712246_F.oxysporumSchlechtendalf.sp.dianthi__Carnation          580 ACCGTCATTGGTATGTTGTCGCTC--ATGCT-TCATTCTA 616
33.MAFF305558_F.oxysporumSchlechtendalf.sp.fragariae__Watermelon       578 ACCGTCATTGGTATGTTGTCGCTC--ATGCT-TCATTCTA 614
34.MAFF744087_F.oxysporumSchlechtendalf.sp.lactucae__Lettuce           578 ACCGTCATTGGTATGTTGTCGCTC--ATGCT-TCATTCTA 614
35.MAFF726924_F.oxysporumSchlechtendalf.sp.lagenariae_Whitefloweredgourd 578 ACCGTCATTGGTATGTTGTCGCTC--ATGCT-TCATTCTA 614
36.MAFF744003_F.oxysporumSchlechtendalf.sp.lagenariae_Squash)          579 ACCGTCATTGGTATGTTGTCGCTC--ATGCT-TCATTCTA 615
37.MAFF305122_F.oxysporumSchlechtendalf.sp.melonis__Melon              578 ACCGTCATTGGTATGTTGTCGCTC--ATGCT-TCATTCTA 614
38.MAFF306714_F.oxysporumSchlechtendalf.sp.momordicae_Balsampear       578 ACCGTCATTGGTATGTTGTCGCTC--ATGCT-TCATTCTA 614
39.MAFF238905_F.oxysporumSchlechtendalf.sp.radicis-lycopersici_Tomato  581 ACCGTCATTGGTATGTTGTCTCTC--ATGCT-TCATTCTA 617
40.MAFF150004_F.oxysporumSchlechtendalf.sp.spinaciae_Spinach           578 ACCGTCATTGGTATGTTGTCGCTC--ATGCT-TCATTCTA 614
41.MAFF247034_F.oxysporumSchlechtendal__Goldenchain                    578 ACCGTCATTGGTATGTTGTCGCTC--ATGCT-TCATTCTA 614
42.MAFF245747_F.oxysporumSchlechtendalf.sp.callistephi__Chinaaster     578 ACCGTCATTGGTATGTTGTCGCTC--ATGCT-TCATTCTA 614
43.MAFF305115_FoxysporumSchlechtendalf.sp.batatas__Sweatpotato         614 ACCGTCATTGGTATGTTGCTGTCA----CCTCTC-TCACA 648
44.MAFF150126_F.asiaticum_Seed                                         576 ACCGTCATTGGTATGTTGTCAC-----CACT-GCTGTCAT 609
45.MAFF246738_F.solani_Angelica                                        614 ACCGTCATTGGTATGTTGCTGTCA----CCTCTC-TCACA 648
46.MAFF246664_F.cugenangense_Perilla                                   578 ACCGTCATTGGTATGTTGTCGCTC--ATGCT-TCATTCTA 614
47.MH582420.1F.solanistrainMRC256                                      601 ACCGTCATTGGTATGTTGCTGTCA----CCTCTC-TCACA 635
48.MAFF240361_F.babinda_Soil                                           597 ACCGTCATTGGTATGTTGCCACTGTTACTGTCACCTTAGT 636
49.MAFF242368_F.azukicola_Azukibean                                    618 ACCGTCATTGGTATGTCGCTGCTG----CCTCTCATCGCT 653
50.MAFF241312_F.asiaticum_Soil,welshonionfield                         576 ACCGTCATTGGTATGTTGTCAC-----CACT-GCTGTCAT 609
51.LT548416.1_F.culmorumpartialtef1a                                   569 ACCGTCATTGGTATGTTGTCAC-----TACT-GCTGTCAT 602
52.MAFF150124_F.asiaticum__Wheat                                       576 ACCGTCATTGGTATGTTGTCAC-----CACT-GCTGTCAT 609
53.MAFF238806_F.begoniae_Oncidiumsp                                    591 ACCGTCATTGGTATGTTGTCGCTC--ATGCC-TCGTTCTC 627
54.MW594399.1_FusariumincarnatumisolateUD01C                           590 ACCGTCATTGGTACGTTATCA-TC----ACTTACACTCAA 624
55.OP414923.1Pucciniagraminisf.sp.triticiisolateSHZPgt19               285 ---------------------------------------- 285
56.MT027094.1_BipolarisoryzaestrainOrL-2                                 0 ---------------------------------------- 0
57.ON734360.1_AlternariaalternataisolateH126                             0 ---------------------------------------- 0
58.LC333578.1_StemphyliumlycopersiciSOasp2                               0 ---------------------------------------- 0
59.HQ718583.1_Colletotrichumgloeosporioidesisolateq-1                  567 ---------------------------------------- 567
60.JN241603.1_AtheliarolfsiiisolateSR1                                 161 ACCGTGAGTAGCTCATGATATTTGATTGTGGTACTTAGCA 200
61.KJ866474.1_RhizoctoniasolanistrainMHL-1                               0 ---------------------------------------- 0
62.JQ672424.1AlternariatriticinaisolateEGS17-061                        68 ACCCACCACTACTATGCGAGACATCTC-TAACCAGCAACA 106
63.LT707559.1_P.capsicipartialtef1agene                                 52 ACGGTCA--------------------------------- 58
```

```
64.MW090051.1_CurvularialunatastrainCls-3                               0 ---------------------------------------- 0
65.DQ400892.1_Aspergillusterreus                                        0 ---------------------------------------- 0
66.DQ911416.1_Pythiumsp.quercumstrainPy292                             31 ACGGTGA--------------------------------- 37
67.EU797495.1_Phytophthorasp.oaksoilPoland                             31 ACGGTCA--------------------------------- 37
68.HM148321.1_Cladosporiumcucumerinum                                 340 ACCGTCATTGACGCCCCCGGTCACCGTGATTTCATCAAGA 379
69.AF398888.1_SclerotiniasclerotiorumisolateSS1                         0 ---------------------------------------- 0
70.AF398888.1_S.sclerotiorumisolateSS1                                  0 ---------------------------------------- 0
71.HPAB545908.1_Verticilliumnonalfalfaeisolate                          0 ---------------------------------------- 0
72.EF433315.1_CeratocystisfimbriatavoucherCMW15052                    522 ---------------------------------------- 522
73.MN159912.1_Botrytiscinerea                                           0 ---------------------------------------- 0
74.MF034741.1_PeltasterfructicolaisolateSRB92                           0 ---------------------------------------- 0
75.LC440360.1_CercosporaasparagiCOasp2                                  0 ---------------------------------------- 0
76.AY944105.1_MagnaportheoryzaeisolateSAG00T3()                       255 ---------------------------------------- 255
77.JX266586.1_CochliobolusmiyabeanusvoucherMFLUCC10-0733                0 ---------------------------------------- 0
78.MN393253.1_CorynesporacassiicolaisolateQHD001(MN393253.1UNVERIFIED)  0 ---------------------------------------- 0
79.MF375218.1_AgroatheliarolfsiiisolateBJB24                            0 ---------------------------------------- 0
80.MN106270.1_AgroatheliarolfsiistrainJ-12                              0 ---------------------------------------- 0
81.OQ732628.1_AgroatheliarolfsiiisolateBTCBSr3                          0 ---------------------------------------- 0
82.KY196185.1_ColletotrichumtruncatumstrainPAK53                      468 ---------------------------------------- 468
83.GU935835.1_ColletotrichumcoccodesisolateC96002                     434 ACCGTCATTGGTAAGTTTTGACATCCCCTAGCTCAGATGA 473
84.MK085963.1_AlternariatenuissimaisolateSCCZ06                         0 ---------------------------------------- 0
85.MT548042.1_AlternarialongipesstrainKY_2019_012                       0 ---------------------------------------- 0
86.MN356465.1CalonectriamontanaisolateHSP4                            398 ACCGTCATTGGTAAGCTTTGATTCCATCCGACTCTTGCCA 437
87.OL694224.1_CalonectriacanadianastrainF099                          400 ACCGTCATTGGTAAGCTTTGATTCCATCCGACTCTTGCCA 439
88.MK803351.1_NeoscytalidiumdimidiatumstrainKale4-C                     0 ---------------------------------------- 0
89.ON376993.1_CurvulariachiangmaiensisisolateND00J7                     0 ---------------------------------------- 0
90.OQ383346.1_NeoscytalidiumdimidiatumisolateGKH-2                      0 ---------------------------------------- 0
91.MF662595.1_NeoscytalidiumnovaehollandiaeisolateNeNo1                 0 ---------------------------------------- 0
92.EF560588.1Melampsoralini                                           380 ---------------------------------------- 380
93.LC590862.1_NeoscytalidiumdimidiatumPSU-HP01TEF1                      0 ---------------------------------------- 0
94.KX278106.1_BotryosphaeriaqingyuanensisstrainCERC2947                 0 ---------------------------------------- 0
95.AJ578763.1_Blumeriagraminisf.sp.hordeicyp51                          0 ---------------------------------------- 0
96.MF490858.1_CurvulariadactylocteniicolastrainCPC28810                 0 ---------------------------------------- 0
97.KT287115.1_Bipolariscactivoraisolate3.8.6                            0 ---------------------------------------- 0
98.MT560940.1_CurvulariacactivorastrainHLGH0118                         0 ---------------------------------------- 0
99.OM714565.1_CurvulariaplantarumstrainM0134                            0 ---------------------------------------- 0
100.MN159911.1_BotrytiscinereaSICAUCC19-0003                            0 ---------------------------------------- 0
102.GU294713.1LasiodiplodiatheobromaestrainUCD2430TX                    0 ---------------------------------------- 0
103.KX868094.1_Mycosphaerellasp.isolateCRM20.1                        371 -ACGTATTTCCTGACCCGAACC----ATTTATGTATGGTG 405
104.LC599478.1Pseudocercosporapini-densifloraeMUCC534                   0 ---------------------------------------- 0
105.N584698.1BipolarissetariaestrainKBS4-2                              0 ---------------------------------------- 0

2.OM160859.1_F.buharicum                                              560 ---------------------------------------- 560
1.LC727524.1_F.buharicum_OKI-1_Okura                                  626 CGCACACTCACTCTAACAATGTCCATA--GACGCTCCCGG 663
3.KX302919.1_F.sublunatum                                             521 ---------------------------------------- 521
4.LT996094.1_F.convolutans                                            516 ---------------------------------------- 516
```

5.OM160861.1_F.abutilonis	588	CACATGATTGTTCTAACAATGTATTCA--GACGCTCCCGG	625
6.OM160874.1_F.guadeloupense	560	--	560
7.MH392475.1_F.graminearum	430	--	430
8.MH582420.1_F.solani	540	--	540
9.MAFF244605_F.oxysporum	544	--	544
10.MAFF237278_F.contaminatum_Hylocereus	633	CTTCTCTTCGTACTAACATATCACTCA--GACGCTCCCGG	670
11.MAFF237649_F.concentricum__Riceroot	614	CTTC--CTCGTACTAACATATCACTCA--GACGCTCCCGG	649
12.MAFF237650_F.concentricum__Wheat	615	CTTC--CTCGTACTAACATATCACTCA--GACGCTCCCGG	650
13.MAFF239869_F.mangiferae__Ryukyupine	603	CTTC--CTCGTACTAACATGTCATCCA--GACGCTCCCGG	638
14.MAFF240460_F.fujikuroi_Passionfruit	607	CTTA--TTCATACTAACATATCATTCA--GACGCTCCCGG	642
15.MAFF241317_F.graminearum_Wheat	610	CACATTCTCATACTAACATGGCTATCA--GACGCTCCCGG	647
16.MAFF242670_F.ipomoeae_Wheat	616	TACCTTCTCATGCTAACATGTATTCCA--GACGCTCCCGG	653
17.MAFF245129_F.concentricum_Fraxinus	605	CTTC--CTCGTACTAACATATCACTCA--GACGCTCCCGG	640
18.MAFF245395_F.cugenangense_Rhubarb	615	CTTCTCTTCGTACTAACATATCACTCA--GACGCTCCCGG	652
19.MAFF246637_F.nirenbergiae_Strawberry	617	CTTCTCTTCGTACTAACAT--CACTCA--GACGCTCCCGG	652
20.MAFF246672_F.nirenbergiae_ChinesePeony	617	CTTCTCTTCGTACTAACAT--CACTCA--GACGCTCCCGG	652
21.MAFF246697_F.commune_Urallicoriceroot	617	CTTCTCTTCGTACTGACATATCACTCA--GACGCTCCCGG	654
22.MAFF246729_F.falciforme_Angelica	637	CATGTCTCACCACTAACA-ATCAA-CA--GACGCCCCCGG	672
23.MAFF247220_F.duplospermum__Euwallaceasp	640	CATGTCTCACCACTAACA-ATCAA-CA--GACGCCCCCGG	675
24.MAFF410760_F.odoratissimum_alpha	613	CGTCTCTTCGTACTAATATATCACTCA--GACGCTCCCGG	650
25.MAFF244605_FusariumoxysporumSchlechtendal_MAFF244605_Tomato	616	CTTCTCTTCGTACTGACATATCACTCA--GACGCTCCCGG	653
26.MAFF241326_F.asiaticum_Wheat	613	CACATTCTCATACTAACATGGCTATCA--GACGCTCCCGG	650
27.MAFF245014_F.asiaticum_Wildsoybean	610	CACATTCTCATACTAACATGGCTATCA--GACGCTCCCGG	647
28.MAFF150124_F.asiaticum__Wheat	610	CACATTCTCATACTAACATGGCTACCA--GACGCTCCCGG	647
29.OM135603.1F.algeriense	636	CT------CATGCTAACATGACATTTA--GACGCTCCCGG	667
30.MAFF237465_F.penzigii_Aloe	636	GTCCTCCAGTCACTAACATGGCCATCACAGACGCTCCCGG	675
31.MAFF103054_F.oxysporumSchlechtendalf.sp.cucumerinum_Cucumber	615	CTTCTCTTCGTACTAACATATCACTCA--GACGCTCCCGG	652
32.MAFF712246_F.oxysporumSchlechtendalf.sp.dianthi__Carnation	617	CTTCTCTTCGTACTAACATATCACTCA--GACGCTCCCGG	654
33.MAFF305558_F.oxysporumSchlechtendalf.sp.fragariae__Watermelon	615	CTTCTCTTCGTACTAACATATCACTCA--AACGCTCCCGG	652
34.MAFF744087_F.oxysporumSchlechtendalf.sp.lactucae__Lettuce	615	CTTCTCTTCGTACTAACATATCACTCA--GACGCTCCCGG	652
35.MAFF726924_F.oxysporumSchlechtendalf.sp.lagenariae_Whitefloweredgourd	615	CTTCTCTTCGTACTAACATATCACTCA--GACGCTCCCGG	652
36.MAFF744003_F.oxysporumSchlechtendalf.sp.lagenariae_Squash)	616	CTTCTCTTCGTACTAACATATCACTCA--GACGCTCCCGG	653
37.MAFF305122_F.oxysporumSchlechtendalf.sp.melonis__Melon	615	CTTCTCTTCGTACTAACATATCACTCA--GACGCTCCCGG	652
38.MAFF306714_F.oxysporumSchlechtendalf.sp.momordicae_Balsampear	615	CTTCTCTTCGTACTAACATATCACTCA--GACGCTCCCGG	652
39.MAFF238905_F.oxysporumSchlechtendalf.sp.radicis-lycopersici_Tomato	618	CTTCTCTTCGTACTAACATATCACTCA--GACGCTCCCGG	655
40.MAFF150004_F.oxysporumSchlechtendalf.sp.spinaciae_Spinach	615	CTTCTCTTCGTACTAACATATCACTCA--GACGCTCCCGG	652
41.MAFF247034_F.oxysporumSchlechtendal__Goldenchain	615	CTTCTCTTCGTACTAACATATCACTCA--GACGCTCCCGG	652
42.MAFF245747_F.oxysporumSchlechtendalf.sp.callistephi__Chinaaster	615	CTTCTCTTCGTACTAACATATCACTCA--GACGCTCCCGG	652
43.MAFF305115_FoxysporumSchlechtendalf.sp.batatas__Sweatpotato	649	CATGTCTCACCACTAACA-ATCAA-CA--GACGCCCCCGG	684
44.MAFF150126_F.asiaticum_Seed	610	CACATTCTCATACTAACATGGCTATCA--GACGCTCCCGG	647
45.MAFF246738_F.solani_Angelica	649	CATGTCTCACCACTAACA-ATCAA-CA--GACGCCCCCGG	684
46.MAFF246664_F.cugenangense_Perilla	615	CTTCTCTTCGTACTAACATATCACTCA--GACGCTCCCGG	652
47.MH582420.1F.solanistrainMRC256	636	CATGTCTCACCACTAACA-ATCAA-CA--GACGCCCCCGG	671
48.MAFF240361_F.babinda_Soil	637	CTTGATCTCATGCTAACATCTCATTCA--GACGCTCCCGG	674
49.MAFF242368_F.azukicola_Azukibean	654	CATGTCTCACCACTAACA-ATCGA-CA--GACGCTCCGGG	689
50.MAFF241312_F.asiaticum_Soil,welshonionfield	610	CACATTCTCATACTAACATGGCTATCA--GACGCTCCCGG	647

```
51.LT548416.1_F.culmorumpartialtef1a                                  603 CACATTCTCATACTAACACGACTATCA--GACGCTCCCGG 640
52.MAFF150124_F.asiaticum__Wheat                                      610 CACATTCTCATACTAACATGGCTACCA--GACGCTCCCGG 647
53.MAFF238806_F.begoniae_Oncidiumsp                                   628 CCTTTATTCATACTAACATATCACTCA--GACGCTCCCGG 665
54.MW594399.1_FusariumincarnatumisolateUD01C                          625 TACTTTCTCATGCTAACATGTACTTCA--GACGCTCCCGG 662
55.OP414923.1Pucciniagraminisf.sp.triticiisolateSHZPgt19              285 ---------------------------------------- 285
56.MT027094.1_BipolarisoryzaestrainOrL-2                                0 ---------------------------------------- 0
57.ON734360.1_AlternariaalternataisolateH126                            0 ---------------------------------------- 0
58.LC333578.1_StemphyliumlycopersiciSOasp2                              0 ---------------------------------------- 0
59.HQ718583.1_Colletotrichumgloeosporioidesisolateq-1                 567 ---------------------------------------- 567
60.JN241603.1_AtheliarolfsiiisolateSR1                                201 GGGGATCTACAATGTAAATCTCGTCCAGGATCATCGATAG 240
61.KJ866474.1_RhizoctoniasolanistrainMHL-1                              0 ---------------------------------------- 0
62.JQ672424.1AlternariatriticinaisolateEGS17-061                      107 GAT-ACTACGTCACCGTCAGTATGTGCTATCAACGGAACC 145
63.LT707559.1_P.capsicipartialtef1agene                                58 ---------------------------------------- 58
64.MW090051.1_CurvularialunatastrainCls-3                               0 ---------------------------------------- 0
65.DQ400892.1_Aspergillusterreus                                        0 ---------------------------------------- 0
66.DQ911416.1_Pythiumsp.quercumstrainPy292                             37 ---------------------------------------- 37
67.EU797495.1_Phytophthorasp.oaksoilPoland                             37 ---------------------------------------- 37
68.HM148321.1_Cladosporiumcucumerinum                                 380 ACATGA---------------------------------- 385
69.AF398888.1_SclerotiniasclerotiorumisolateSS1                         0 ---------------------------------------- 0
70.AF398888.1_S.sclerotiorumisolateSS1                                  0 ---------------------------------------- 0
71.HPAB545908.1_Verticilliumnonalfalfaeisolate                          0 ---------------------------------------- 0
72.EF433315.1_CeratocystisfimbriatavoucherCMW15052                    522 ---------------------------------------- 522
73.MN159912.1_Botrytiscinerea                                           0 ---------------------------------------- 0
74.MF034741.1_PeltasterfructicolaisolateSRB92                           0 ---------------------------------------- 0
75.LC440360.1_CercosporaasparagiCOasp2                                  0 ---------------------------------------- 0
76.AY944105.1_MagnaportheoryzaeisolateSAG00T3()                       255 ---------------------------------------- 255
77.JX266586.1_CochliobolusmiyabeanusvoucherMFLUCC10-0733                0 ---------------------------------------- 0
78.MN393253.1_CorynesporacassiicolaisolateQHD001(MN393253.1UNVERIFIED)  0 ---------------------------------------- 0
79.MF375218.1_AgroatheliarolfsiiisolateBJB24                            0 ---------------------------------------- 0
80.MN106270.1_AgroatheliarolfsiistrainJ-12                              0 ---------------------------------------- 0
81.OQ732628.1_AgroatheliarolfsiiisolateBTCBSr3                          0 ---------------------------------------- 0
82.KY196185.1_ColletotrichumtruncatumstrainPAK53                      468 ---------------------------------------- 468
83.GU935835.1_ColletotrichumcoccodesisolateC96002                     474 GATTAGCACTTGCCAATCTTGGGGCGCAAAATCTTGATAC 513
84.MK085963.1_AlternariatenuissimaisolateSCCZ06                         0 ---------------------------------------- 0
85.MT548042.1_AlternarialongipesstrainKY_2019_012                       0 ---------------------------------------- 0
86.MN356465.1CalonectriamontanaisolateHSP4                            438 TCTAGCATCTAACCAAC-TCTGAA-CA--GATGCCCCCGG 473
87.OL694224.1_CalonectriacanadianastrainF099                          440 TCTAGCATCTAACCAAC-TCTGAA-CA--GATGCCCCCGG 475
88.MK803351.1_NeoscytalidiumdimidiatumstrainKale4-C                     0 ---------------------------------------- 0
89.ON376993.1_CurvulariachiangmaiensisisolateND00J7                     0 ---------------------------------------- 0
90.OQ383346.1_NeoscytalidiumdimidiatumisolateGKH-2                      0 ---------------------------------------- 0
91.MF662595.1_NeoscytalidiumnovaehollandiaeisolateNeNo1                 0 ---------------------------------------- 0
92.EF560588.1Melampsoralini                                           380 ---------------------------------------- 380
93.LC590862.1_NeoscytalidiumdimidiatumPSU-HP01TEF1                      0 ---------------------------------------- 0
94.KX278106.1_BotryosphaeriaqingyuanensisstrainCERC2947                 0 ---------------------------------------- 0
95.AJ578763.1_Blumeriagraminisf.sp.hordeicyp51                          0 ---------------------------------------- 0
96.MF490858.1_CurvulariadactylocteniicolastrainCPC28810                 0 ---------------------------------------- 0
```

```
97.KT287115.1_Bipolariscactivoraisolate3.8.6                          0 ---------------------------------------- 0
98.MT560940.1_CurvulariacactivorastrainHLGH0118                       0 ---------------------------------------- 0
99.OM714565.1_CurvulariaplantarumstrainM0134                          0 ---------------------------------------- 0
100.MN159911.1_BotrytiscinereaSICAUCC19-0003                          0 ---------------------------------------- 0
102.GU294713.1LasiodiplodiatheobromaestrainUCD2430TX                  0 ---------------------------------------- 0
103.KX868094.1_Mycosphaerellasp.isolateCRM20.1                      406 TACTAACGTCTGTTAAGGTCACCGTCATCGACGCCCCAGG 445
104.LC599478.1Pseudocercosporapini-densifloraeMUCC534                 0 ---------------------------------------- 0
105.N584698.1BipolarissetariaestrainKBS4-2                            0 ---------------------------------------- 0

2.OM160859.1_F.buharicum                                            560 ---------------------------------------- 560
1.LC727524.1_F.buharicum_OKI-1_Okura                                664 TCACCGTGATTTCATCAAG--------------------- 682
3.KX302919.1_F.sublunatum                                           521 ---------------------------------------- 521
4.LT996094.1_F.convolutans                                          516 ---------------------------------------- 516
5.OM160861.1_F.abutilonis                                           626 TCACC----------------------------------- 630
6.OM160874.1_F.guadeloupense                                        560 ---------------------------------------- 560
7.MH392475.1_F.graminearum                                          430 ---------------------------------------- 430
8.MH582420.1_F.solani                                               540 ---------------------------------------- 540
9.MAFF244605_F.oxysporum                                            544 ---------------------------------------- 544
10.MAFF237278_F.contaminatum_Hylocereus                             671 TCACCGTGATTTCATCAAGAACATGATGACTGGGTACCTC 710
11.MAFF237649_F.concentricum__Riceroot                              650 TCACCGTGATTTCATCAAGAACATGATCA----------- 678
12.MAFF237650_F.concentricum__Wheat                                 651 TCACCGTGATTTCATCAAGAACATGATCA----------- 679
13.MAFF239869_F.mangiferae__Ryukyupine                              639 TCACCGTGATTTCATCAAG--------------------- 657
14.MAFF240460_F.fujikuroi_Passionfruit                              643 TCACCGTGATTTCATCAAG--------------------- 661
15.MAFF241317_F.graminearum_Wheat                                   648 TCACCGTGATTTCATCAAG--------------------- 666
16.MAFF242670_F.ipomoeae_Wheat                                      654 TCACCGTGATTTCATCAAG--------------------- 672
17.MAFF245129_F.concentricum_Fraxinus                               641 TCACCGTGATTTCATCAAG--------------------- 659
18.MAFF245395_F.cugenangense_Rhubarb                                653 TCACCGTGATTTCATCAAG--------------------- 671
19.MAFF246637_F.nirenbergiae_Strawberry                             653 TCACCGTGATTTCATCAAG--------------------- 671
20.MAFF246672_F.nirenbergiae_ChinesePeony                           653 TCACCGTGATTTCATCAAG--------------------- 671
21.MAFF246697_F.commune_Urallicoriceroot                            655 TCACCGTGATTTCATCAAG--------------------- 673
22.MAFF246729_F.falciforme_Angelica                                 673 CCACCGTGATTTCATCAAG--------------------- 691
23.MAFF247220_F.duplospermum__Euwallaceasp                          676 CCACCGTGACTTCATCAAG--------------------- 694
24.MAFF410760_F.odoratissimum_alpha                                 651 TCACCGTGATTTCATCAAG--------------------- 669
25.MAFF244605_FusariumoxysporumSchlechtendal_MAFF244605_Tomato      654 TCACCGTGATTTCATCAAG--------------------- 672
26.MAFF241326_F.asiaticum_Wheat                                     651 TCACCGTGATTTCATCAAG--------------------- 669
27.MAFF245014_F.asiaticum_Wildsoybean                               648 TCACCGTGATTTCATCAAG--------------------- 666
28.MAFF150124_F.asiaticum__Wheat                                    648 TCACCGTGATTTCATCAAG--------------------- 666
29.OM135603.1F.algeriense                                           668 TCACCGTGATTTCATCAAGAACATGATCACTG-------- 699
30.MAFF237465_F.penzigii_Aloe                                       676 TCACCGTGATTTCATCAAGAACATGATCA----------- 704
31.MAFF103054_F.oxysporumSchlechtendalf.sp.cucumerinum_Cucumber     653 TCACCGTGATTTCATCAAG--------------------- 671
32.MAFF712246_F.oxysporumSchlechtendalf.sp.dianthi__Carnation       655 TCACCGTGATTTCATCAAG--------------------- 673
33.MAFF305558_F.oxysporumSchlechtendalf.sp.fragariae__Watermelon    653 TCACCGTGATTTCATCAAG--------------------- 671
34.MAFF744087_F.oxysporumSchlechtendalf.sp.lactucae__Lettuce        653 TCACCGTGATTTCATCAAG--------------------- 671
35.MAFF726924_F.oxysporumSchlechtendalf.sp.lagenariae_Whiteflorwredgourd 653 TCACCGTGATTTCATCAAG--------------------- 671
36.MAFF744003_F.oxysporumSchlechtendalf.sp.lagenariae_Squash)       654 TCACCGTGATTTCATCAAG--------------------- 672
37.MAFF305122_F.oxysporumSchlechtendalf.sp.melonis__Melon           653 TCACCGTGATTTCATCAAG--------------------- 671
```

38.MAFF306714_F.oxysporumSchlechtendalf.sp.momordicae_Balsampear	653	TCACCGTGATTTCATCAAG---------------------	671
39.MAFF238905_F.oxysporumSchlechtendalf.sp.radicis-lycopersici_Tomato	656	TCACCGTGATTTCATCAAG---------------------	674
40.MAFF150004_F.oxysporumSchlechtendalf.sp.spinaciae_Spinach	653	TCACCGTGATTTCATCAAG---------------------	671
41.MAFF247034_F.oxysporumSchlechtendal__Goldenchain	653	TCACCGTGATTTCATCAAG---------------------	671
42.MAFF245747_F.oxysporumSchlechtendalf.sp.callistephi__Chinaaster	653	TCACCGTGATTTCATCAAG---------------------	671
43.MAFF305115_FoxysporumSchlechtendalf.sp.batatas__Sweatpotato	685	CCACCGTGACTTCATCAAG---------------------	703
44.MAFF150126_F.asiaticum_Seed	648	TCACCGTGATTTCATCAAG---------------------	666
45.MAFF246738_F.solani_Angelica	685	CCACCGTGACTTCATCAAG---------------------	703
46.MAFF246664_F.cugenangense_Perilla	653	TCACCGTGATTTCATCAAG---------------------	671
47.MH582420.1F.solanistrainMRC256	672	CCACCGTG--------------------------------	679
48.MAFF240361_F.babinda_Soil	675	TCATCGTGACTTCATCAAG---------------------	693
49.MAFF242368_F.azukicola_Azukibean	690	CCACCGTGACTTCATCAAG---------------------	708
50.MAFF241312_F.asiaticum_Soil,welshonionfield	648	TCACCGTGATTTCATCAAG---------------------	666
51.LT548416.1_F.culmorumpartialtef1a	641	TCACCG----------------------------------	646
52.MAFF150124_F.asiaticum__Wheat	648	TCACCGTGATTTCATCAAG---------------------	666
53.MAFF238806_F.begoniae_Oncidiumsp	666	CCATCGTGACTTCATCAAGAACATGATGACTGG-TACTTC	704
54.MW594399.1_FusariumincarnatumisolateUD01C	663	TCACCGTGATTTCATCAAGAACATGAT-------------	689
55.OP414923.1Pucciniagraminisf.sp.triticiisolateSHZPgt19	285	--	285
56.MT027094.1_BipolarisoryzaestrainOrL-2	0	--	0
57.ON734360.1_AlternariaalternataisolateH126	0	--	0
58.LC333578.1_StemphyliumlycopersiciSOasp2	0	--	0
59.HQ718583.1_Colletotrichumgloeosporioidesisolateq-1	567	--	567
60.JN241603.1_AtheliarolfsiiisolateSR1	241	CCCCTGGCTCACCTA-------------------------	255
61.KJ866474.1_RhizoctoniasolanistrainMHL-1	1	--------------C-------------------------	1
62.JQ672424.1AlternariatriticinaisolateEGS17-061	146	TCCTTATCACGACCTTCAATCGCTGACAATTTACTTAGTT	185
63.LT707559.1_P.capsicipartialtef1agene	59	--------------------------------------TT	60
64.MW090051.1_CurvularialunatastrainCls-3	0	--	0
65.DQ400892.1_Aspergillusterreus	0	--	0
66.DQ911416.1_Pythiumsp.quercumstrainPy292	38	--------------------------------------TT	39
67.EU797495.1_Phytophthorasp.oaksoilPoland	38	--------------------------------------TT	39
68.HM148321.1_Cladosporiumcucumerinum	385	--	385
69.AF398888.1_SclerotiniasclerotiorumisolateSS1	0	--	0
70.AF398888.1_S.sclerotiorumisolateSS1	0	--	0
71.HPAB545908.1_Verticilliumnonalfalfaeisolate	0	--	0
72.EF433315.1_CeratocystisfimbriatavoucherCMW15052	522	--	522
73.MN159912.1_Botrytiscinerea	0	--	0
74.MF034741.1_PeltasterfructicolaisolateSRB92	0	--	0
75.LC440360.1_CercosporaasparagiCOasp2	0	--	0
76.AY944105.1_MagnaportheoryzaeisolateSAG00T3()	255	--	255
77.JX266586.1_CochliobolusmiyabeanusvoucherMFLUCC10-0733	0	--	0
78.MN393253.1_CorynesporacassiicolaisolateQHD001(MN393253.1UNVERIFIED)	0	--	0
79.MF375218.1_AgroatheliarolfsiiisolateBJB24	0	--	0
80.MN106270.1_AgroatheliarolfsiistrainJ-12	0	--	0
81.OQ732628.1_AgroatheliarolfsiiisolateBTCBSr3	0	--	0
82.KY196185.1_ColletotrichumtruncatumstrainPAK53	468	--	468
83.GU935835.1_ColletotrichumcoccodesisolateC96002	514	AGATTACTAACATGTTCCA---------------------	532

```
84.MK085963.1_AlternariatenuissimaisolateSCCZ06                0 ---------------------------------------- 0
85.MT548042.1_Alternarialongipesstrain KY_2019_012             0 ---------------------------------------- 0
86.MN356465.1CalonectriamontanaisolateHSP4                   474 TCACCGTGACTTCATC------------------------ 489
87.OL694224.1_CalonectriacanadianastrainF099                 476 TCACCGTGACTTCATCAAGAACATGATCA----------- 504
88.MK803351.1_NeoscytalidiumdimidiatumstrainKale4-C            0 ---------------------------------------- 0
89.ON376993.1_CurvulariachiangmaiensisisolateND00J7            0 ---------------------------------------- 0
90.OQ383346.1_NeoscytalidiumdimidiatumisolateGKH-2             0 ---------------------------------------- 0
91.MF662595.1_NeoscytalidiumnovaehollandiaeisolateNeNo1        0 ---------------------------------------- 0
92.EF560588.1Melampsoralini                                  380 ---------------------------------------- 380
93.LC590862.1_NeoscytalidiumdimidiatumPSU-HP01TEF1             0 ---------------------------------------- 0
94.KX278106.1_BotryosphaeriaqingyuanensisstrainCERC2947        0 ---------------------------------------- 0
95.AJ578763.1_Blumeriagraminisf.sp.hordeicyp51                 0 ---------------------------------------- 0
96.MF490858.1_CurvulariadactylocteniicolastrainCPC28810        0 ---------------------------------------- 0
97.KT287115.1_Bipolariscactivoraisolate3.8.6                   0 ---------------------------------------- 0
98.MT560940.1_CurvulariacactivorastrainHLGH0118                0 ---------------------------------------- 0
99.OM714565.1_CurvulariaplantarumstrainM0134                   0 ---------------------------------------- 0
100.MN159911.1_BotrytiscinereaSICAUCC19-0003                   0 ---------------------------------------- 0
102.GU294713.1LasiodiplodiatheobromaestrainUCD2430TX           0 ---------------------------------------- 0
103.KX868094.1_Mycosphaerellasp.isolateCRM20.1               446 TCACCGTGA-TTCATCAAGAACATGATCACT--------- 475
104.LC599478.1Pseudocercosporapini-densifloraeMUCC534          0 ---------------------------------------- 0
105.N584698.1BipolarissetariaestrainKBS4-2                     0 ---------------------------------------- 0

2.OM160859.1_F.buharicum                                     560 ---------------------------------------- 560
1.LC727524.1_F.buharicum_OKI-1_Okura                         682 ---------------------------------------- 682
3.KX302919.1_F.sublunatum                                    521 ---------------------------------------- 521
4.LT996094.1_F.convolutans                                   516 ---------------------------------------- 516
5.OM160861.1_F.abutilonis                                    630 ---------------------------------------- 630
6.OM160874.1_F.guadeloupense                                 560 ---------------------------------------- 560
7.MH392475.1_F.graminearum                                   430 ---------------------------------------- 430
8.MH582420.1_F.solani                                        540 ---------------------------------------- 540
9.MAFF244605_F.oxysporum                                     544 ---------------------------------------- 544
10.MAFF237278_F.contaminatum_Hylocereus                      711 C--------------------------------------- 711
11.MAFF237649_F.concentricum__Riceroot                       678 ---------------------------------------- 678
12.MAFF237650_F.concentricum__Wheat                          679 ---------------------------------------- 679
13.MAFF239869_F.mangiferae__Ryukyupine                       657 ---------------------------------------- 657
14.MAFF240460_F.fujikuroi_Passionfruit                       661 ---------------------------------------- 661
15.MAFF241317_F.graminearum_Wheat                            666 ---------------------------------------- 666
16.MAFF242670_F.ipomoeae_Wheat                               672 ---------------------------------------- 672
17.MAFF245129_F.concentricum_Fraxinus                        659 ---------------------------------------- 659
18.MAFF245395_F.cugenangense_Rhubarb                         671 ---------------------------------------- 671
19.MAFF246637_F.nirenbergiae_Strawberry                      671 ---------------------------------------- 671
20.MAFF246672_F.nirenbergiae_ChinesePeony                    671 ---------------------------------------- 671
21.MAFF246697_F.commune_Urallicoriceroot                     673 ---------------------------------------- 673
22.MAFF246729_F.falciforme_Angelica                          691 ---------------------------------------- 691
23.MAFF247220_F.duplospermum__Euwallaceasp                   694 ---------------------------------------- 694
24.MAFF410760_F.odoratissimum_alpha                          669 ---------------------------------------- 669
```

```
25.MAFF244605_FusariumoxysporumSchlechtendal_MAFF244605_Tomato        672 ---------------------------------------- 672
26.MAFF241326_F.asiaticum_Wheat                                       669 ---------------------------------------- 669
27.MAFF245014_F.asiaticum_Wildsoybean                                 666 ---------------------------------------- 666
28.MAFF150124_F.asiaticum__Wheat                                      666 ---------------------------------------- 666
29.OM135603.1F.algeriense                                             699 ---------------------------------------- 699
30.MAFF237465_F.penzigii_Aloe                                         704 ---------------------------------------- 704
31.MAFF103054_F.oxysporumSchlechtendalf.sp.cucumerinum_Cucumber       671 ---------------------------------------- 671
32.MAFF712246_F.oxysporumSchlechtendalf.sp.dianthi__Carnation         673 ---------------------------------------- 673
33.MAFF305558_F.oxysporumSchlechtendalf.sp.fragariae__Watermelon      671 ---------------------------------------- 671
34.MAFF744087_F.oxysporumSchlechtendalf.sp.lactucae__Lettuce          671 ---------------------------------------- 671
35.MAFF726924_F.oxysporumSchlechtendalf.sp.lagenariae_Whitefloweredgourd 671 ---------------------------------------- 671
36.MAFF744003_F.oxysporumSchlechtendalf.sp.lagenariae_Squash)         672 ---------------------------------------- 672
37.MAFF305122_F.oxysporumSchlechtendalf.sp.melonis__Melon             671 ---------------------------------------- 671
38.MAFF306714_F.oxysporumSchlechtendalf.sp.momordicae_Balsampear      671 ---------------------------------------- 671
39.MAFF238905_F.oxysporumSchlechtendalf.sp.radicis-lycopersici_Tomato 674 ---------------------------------------- 674
40.MAFF150004_F.oxysporumSchlechtendalf.sp.spinaciae_Spinach          671 ---------------------------------------- 671
41.MAFF247034_F.oxysporumSchlechtendal__Goldenchain                   671 ---------------------------------------- 671
42.MAFF245747_F.oxysporumSchlechtendalf.sp.callistephi__Chinaaster    671 ---------------------------------------- 671
43.MAFF305115_FoxysporumSchlechtendalf.sp.batatas__Sweatpotato        703 ---------------------------------------- 703
44.MAFF150126_F.asiaticum_Seed                                        666 ---------------------------------------- 666
45.MAFF246738_F.solani_Angelica                                       703 ---------------------------------------- 703
46.MAFF246664_F.cugenangense_Perilla                                  671 ---------------------------------------- 671
47.MH582420.1F.solanistrainMRC256                                     679 ---------------------------------------- 679
48.MAFF240361_F.babinda_Soil                                          693 ---------------------------------------- 693
49.MAFF242368_F.azukicola_Azukibean                                   708 ---------------------------------------- 708
50.MAFF241312_F.asiaticum_Soil,welshonionfield                        666 ---------------------------------------- 666
51.LT548416.1_F.culmorumpartialtef1a                                  646 ---------------------------------------- 646
52.MAFF150124_F.asiaticum__Wheat                                      666 ---------------------------------------- 666
53.MAFF238806_F.begoniae_Oncidiumsp                                   705 C--------------------------------------- 705
54.MW594399.1_FusariumincarnatumisolateUD01C                          689 ---------------------------------------- 689
55.OP414923.1Pucciniagraminisf.sp.triticiisolateSHZPgt19              286 ----------------GTGATTTCA-TCAAAAA-CATGAT 307
56.MT027094.1_BipolarisoryzaestrainOrL-2                                0 ---------------------------------------- 0
57.ON734360.1_AlternariaalternataisolateH126                            0 ---------------------------------------- 0
58.LC333578.1_StemphyliumlycopersiciSOasp2                              0 ---------------------------------------- 0
59.HQ718583.1_Colletotrichumgloeosporioidesisolateq-1                 567 ---------------------------------------- 567
60.JN241603.1_AtheliarolfsiiisolateSR1                                256 ----------------GTGACTTCAATCAAGAATCATGAT 279
61.KJ866474.1_RhizoctoniasolanistrainMHL-1                              2 ----------------GTGATTTCA-TCAAGAA-CATGAT 23
62.JQ672424.1AlternariatriticinaisolateEGS17-061                      186 GACGCCCCCGGTCACCGTGATTTCA-TCAAGAA-CATGAT 223
63.LT707559.1_P.capsicipartialtef1agene                                61 GACGCCCCTGGTCACCGTGACTTCA-TCAAGAA-CATGAT 98
64.MW090051.1_CurvularialunatastrainCls-3                               0 ---------------------------------------- 0
65.DQ400892.1_Aspergillusterreus                                        1 -------------------------------------GAT 3
66.DQ911416.1_Pythiumsp.quercumstrainPy292                             40 GACGCTCCCGGCCGCCGTGACTTCA-TCAAGAA-CATGAT 77
67.EU797495.1_Phytophthorasp.oaksoilPoland                             40 GACGCCCCTGGTCACCGTGACTTCA-TCAAGAA-CATGAT 77
68.HM148321.1_Cladosporiumcucumerinum                                 385 ---------------------------------------- 385
69.AF398888.1_SclerotiniasclerotiorumisolateSS1                         0 ---------------------------------------- 0
70.AF398888.1_S.sclerotiorumisolateSS1                                  0 ---------------------------------------- 0
```

```
71.HPAB545908.1_Verticilliumnonalfalfaeisolate                      0 ---------------------------------------- 0
72.EF433315.1_CeratocystisfimbriatavoucherCMW15052                522 ---------------------------------------- 522
73.MN159912.1_Botrytiscinerea                                       0 ---------------------------------------- 0
74.MF034741.1_PeltasterfructicolaisolateSRB92                       0 ---------------------------------------- 0
75.LC440360.1_CercosporaasparagiCOasp2                              0 ---------------------------------------- 0
76.AY944105.1_MagnaportheoryzaeisolateSAG00T3()                   255 ---------------------------------------- 255
77.JX266586.1_CochliobolusmiyabeanusvoucherMFLUCC10-0733            0 ---------------------------------------- 0
78.MN393253.1_CorynesporacassiicolaisolateQHD001(MN393253.1UNVERIFIED) 0 ---------------------------------------- 0
79.MF375218.1_AgroatheliarolfsiiisolateBJB24                        1 -------------------------------------GAT 3
80.MN106270.1_AgroatheliarolfsiistrainJ-12                          1 -----------------------------AGAA-CATGAT 10
81.OQ732628.1_AgroatheliarolfsiiisolateBTCBSr3                      0 ---------------------------------------- 0
82.KY196185.1_ColletotrichumtruncatumstrainPAK53                  468 ---------------------------------------- 468
83.GU935835.1_ColletotrichumcoccodesisolateC96002                 533 GACGCTCCCGGTCACCGTGACTTCA-TCAAGAA-CATGAT 570
84.MK085963.1_AlternariatenuissimaisolateSCCZ06                     0 ---------------------------------------- 0
85.MT548042.1_AlternarialongipesstrainKY_2019_012                   0 ---------------------------------------- 0
86.MN356465.1CalonectriamontanaisolateHSP4                        489 ---------------------------------------- 489
87.OL694224.1_CalonectriacanadianastrainF099                      504 ---------------------------------------- 504
88.MK803351.1_NeoscytalidiumdimidiatumstrainKale4-C                 0 ---------------------------------------- 0
89.ON376993.1_CurvulariachiangmaiensisisolateND00J7                 0 ---------------------------------------- 0
90.OQ383346.1_NeoscytalidiumdimidiatumisolateGKH-2                  0 ---------------------------------------- 0
91.MF662595.1_NeoscytalidiumnovaehollandiaeisolateNeNo1             0 ---------------------------------------- 0
92.EF560588.1Melampsoralini                                       380 ---------------------------------------- 380
93.LC590862.1_NeoscytalidiumdimidiatumPSU-HP01TEF1                  0 ---------------------------------------- 0
94.KX278106.1_BotryosphaeriaqingyuanensisstrainCERC2947             0 ---------------------------------------- 0
95.AJ578763.1_Blumeriagraminisf.sp.hordeicyp51                      0 ---------------------------------------- 0
96.MF490858.1_CurvulariadactylocteniicolastrainCPC28810             0 ---------------------------------------- 0
97.KT287115.1_Bipolariscactivoraisolate3.8.6                        1 --------------TCGTGATTTTA-TCAAGAA-CATGAT 24
98.MT560940.1_CurvulariacactivorastrainHLGH0118                     0 ---------------------------------------- 0
99.OM714565.1_CurvulariaplantarumstrainM0134                        1 --------------------------------A-CATGAT 7
100.MN159911.1_BotrytiscinereaSICAUCC19-0003                        0 ---------------------------------------- 0
102.GU294713.1LasiodiplodiatheobromaestrainUCD2430TX                0 ---------------------------------------- 0
103.KX868094.1_Mycosphaerellasp.isolateCRM20.1                    475 ---------------------------------------- 475
104.LC599478.1Pseudocercosporapini-densifloraeMUCC534               0 ---------------------------------------- 0
105.N584698.1BipolarissetariaestrainKBS4-2                          1 ---GCTCCTGGACACCGTGACTTTA-TCAAGAA-CATGAT 35

2.OM160859.1_F.buharicum                                          560 ---------------------------------------- 560
1.LC727524.1_F.buharicum_OKI-1_Okura                              682 ---------------------------------------- 682
3.KX302919.1_F.sublunatum                                         521 ---------------------------------------- 521
4.LT996094.1_F.convolutans                                        516 ---------------------------------------- 516
5.OM160861.1_F.abutilonis                                         630 ---------------------------------------- 630
6.OM160874.1_F.guadeloupense                                      560 ---------------------------------------- 560
7.MH392475.1_F.graminearum                                        430 ---------------------------------------- 430
8.MH582420.1_F.solani                                             540 ---------------------------------------- 540
9.MAFF244605_F.oxysporum                                          544 ---------------------------------------- 544
10.MAFF237278_F.contaminatum_Hylocereus                           711 ---------------------------------------- 711
11.MAFF237649_F.concentricum__Riceroot                            678 ---------------------------------------- 678
```

```
12.MAFF237650_F.concentricum__Wheat                                       679 ---------------------------------------- 679
13.MAFF239869_F.mangiferae__Ryukyupine                                    657 ---------------------------------------- 657
14.MAFF240460_F.fujikuroi_Passionfruit                                    661 ---------------------------------------- 661
15.MAFF241317_F.graminearum_Wheat                                         666 ---------------------------------------- 666
16.MAFF242670_F.ipomoeae_Wheat                                            672 ---------------------------------------- 672
17.MAFF245129_F.concentricum_Fraxinus                                     659 ---------------------------------------- 659
18.MAFF245395_F.cugenangense_Rhubarb                                      671 ---------------------------------------- 671
19.MAFF246637_F.nirenbergiae_Strawberry                                   671 ---------------------------------------- 671
20.MAFF246672_F.nirenbergiae_ChinesePeony                                 671 ---------------------------------------- 671
21.MAFF246697_F.commune_Urallicoriceroot                                  673 ---------------------------------------- 673
22.MAFF246729_F.falciforme_Angelica                                       691 ---------------------------------------- 691
23.MAFF247220_F.duplospermum__Euwallaceasp                                694 ---------------------------------------- 694
24.MAFF410760_F.odoratissimum_alpha                                       669 ---------------------------------------- 669
25.MAFF244605_FusariumoxysporumSchlechtendal_MAFF244605_Tomato            672 ---------------------------------------- 672
26.MAFF241326_F.asiaticum_Wheat                                           669 ---------------------------------------- 669
27.MAFF245014_F.asiaticum_Wildsoybean                                     666 ---------------------------------------- 666
28.MAFF150124_F.asiaticum__Wheat                                          666 ---------------------------------------- 666
29.OM135603.1F.algeriense                                                 699 ---------------------------------------- 699
30.MAFF237465_F.penzigii_Aloe                                             704 ---------------------------------------- 704
31.MAFF103054_F.oxysporumSchlechtendalf.sp.cucumerinum_Cucumber           671 ---------------------------------------- 671
32.MAFF712246_F.oxysporumSchlechtendalf.sp.dianthi__Carnation             673 ---------------------------------------- 673
33.MAFF305558_F.oxysporumSchlechtendalf.sp.fragariae__Watermelon          671 ---------------------------------------- 671
34.MAFF744087_F.oxysporumSchlechtendalf.sp.lactucae__Lettuce              671 ---------------------------------------- 671
35.MAFF726924_F.oxysporumSchlechtendalf.sp.lagenariae_Whiteflsoweredgourd 671 ---------------------------------------- 671
36.MAFF744003_F.oxysporumSchlechtendalf.sp.lagenariae_Squash)             672 ---------------------------------------- 672
37.MAFF305122_F.oxysporumSchlechtendalf.sp.melonis__Melon                 671 ---------------------------------------- 671
38.MAFF306714_F.oxysporumSchlechtendalf.sp.momordicae_Balsampear          671 ---------------------------------------- 671
39.MAFF238905_F.oxysporumSchlechtendalf.sp.radicis-lycopersici_Tomato     674 ---------------------------------------- 674
40.MAFF150004_F.oxysporumSchlechtendalf.sp.spinaciae_Spinach              671 ---------------------------------------- 671
41.MAFF247034_F.oxysporumSchlechtendal__Goldenchain                       671 ---------------------------------------- 671
42.MAFF245747_F.oxysporumSchlechtendalf.sp.callistephi__Chinaaster        671 ---------------------------------------- 671
43.MAFF305115_FoxysporumSchlechtendalf.sp.batatas__Sweatpotato            703 ---------------------------------------- 703
44.MAFF150126_F.asiaticum_Seed                                            666 ---------------------------------------- 666
45.MAFF246738_F.solani_Angelica                                           703 ---------------------------------------- 703
46.MAFF246664_F.cugenangense_Perilla                                      671 ---------------------------------------- 671
47.MH582420.1F.solanistrainMRC256                                         679 ---------------------------------------- 679
48.MAFF240361_F.babinda_Soil                                              693 ---------------------------------------- 693
49.MAFF242368_F.azukicola_Azukibean                                       708 ---------------------------------------- 708
50.MAFF241312_F.asiaticum_Soil,welshonionfield                            666 ---------------------------------------- 666
51.LT548416.1_F.culmorumpartialtef1a                                      646 ---------------------------------------- 646
52.MAFF150124_F.asiaticum__Wheat                                          666 ---------------------------------------- 666
53.MAFF238806_F.begoniae_Oncidiumsp                                       705 ---------------------------------------- 705
54.MW594399.1_FusariumincarnatumisolateUD01C                              689 ---------------------------------------- 689
55.OP414923.1Pucciniagraminisf.sp.triticiisolateSHZPgt19                  308 CACT-GGTACCTC-CCAAGCCGATTGTGCT-ATCCTC-AT 343
56.MT027094.1_BipolarisoryzaestrainOrL-2                                    1 --------------------------------TCCTC-AT 7
57.ON734360.1_AlternariaalternataisolateH126                                0 ---------------------------------------- 0
```

```
58.LC333578.1_StemphyliumlycopersiciSOasp2                               0 ---------------------------------------- 0
59.HQ718583.1_Colletotrichumgloeosporioidesisolateq-1                  567 ---------------------------------------- 567
60.JN241603.1_AtheliarolfsiiisolateSR1                                 280 CACTAGGTACTTCACCAGGCCGATTGCGCTCATCCTCTAT 319
61.KJ866474.1_RhizoctoniasolanistrainMHL-1                              24 CACT-GGTACCTC-CCAGGCTGA-TGCGCT-ATTCTC-AT 58
62.JQ672424.1AlternariatriticinaisolateEGS17-061                       224 CACT-GGTACCTC-CCAGGCCGACTGCGCT-ATTCTC-AT 259
63.LT707559.1_P.capsicipartialtef1agene                                 99 TACG-GGTACCTC-GCAGGCCGATTGCGCC-ATTCTG-GT 134
64.MW090051.1_CurvularialunatastrainCls-3                                1 ---------------------------------------T 1
65.DQ400892.1_Aspergillusterreus                                         4 CACT-GGTACCTC-CCAGGCTGACTGCGCT-ATCCTC-AT 39
66.DQ911416.1_Pythiumsp.quercumstrainPy292                              78 CACG-GGCACGTC-GCAGGCCGACTGCGCC-ATCCTT-GT 113
67.EU797495.1_Phytophthorasp.oaksoilPoland                              78 CACG-GGGACCTC-GCAGGCTGACTGCGCC-ATTCTG-GT 113
68.HM148321.1_Cladosporiumcucumerinum                                  385 ---------------------------------------- 385
69.AF398888.1_SclerotiniasclerotiorumisolateSS1                          0 ---------------------------------------- 0
70.AF398888.1_S.sclerotiorumisolateSS1                                   0 ---------------------------------------- 0
71.HPAB545908.1_Verticilliumnonalfalfaeisolate                           1 -----------------GGCCGATTGCGCC-ATTCTC-AT 21
72.EF433315.1_CeratocystisfimbriatavoucherCMW15052                     522 ---------------------------------------- 522
73.MN159912.1_Botrytiscinerea                                            1 ------------------GCTGATTGTGCC-GTTCTC-AT 20
74.MF034741.1_PeltasterfructicolaisolateSRB92                            0 ---------------------------------------- 0
75.LC440360.1_CercosporaasparagiCOasp2                                   0 ---------------------------------------- 0
76.AY944105.1_MagnaportheoryzaeisolateSAG00T3()                        255 ---------------------------------------- 255
77.JX266586.1_CochliobolusmiyabeanusvoucherMFLUCC10-0733                 1 ----------------------ACTGCGCT-ATCCTC-AT 16
78.MN393253.1_CorynesporacassiicolaisolateQHD001(MN393253.1UNVERIFIED)   0 ---------------------------------------- 0
79.MF375218.1_AgroatheliarolfsiiisolateBJB24                             4 CACT-GGTACTTC-CCAGGCCGATTGCGCT-ATCCTC-AT 39
80.MN106270.1_AgroatheliarolfsiistrainJ-12                              11 CACT-GGTACTTC-CCAGGCCGACTGCGCT-ATTCTC-AT 46
81.OQ732628.1_AgroatheliarolfsiiisolateBTCBSr3                           1 --------------CCAGGCCGATTGCGCT-ATCCTC-AT 24
82.KY196185.1_ColletotrichumtruncatumstrainPAK53                       468 ---------------------------------------- 468
83.GU935835.1_ColletotrichumcoccodesisolateC96002                      571 CACT-GGTACCTC-CCAGGCTGACTGCGCT-ATTCTC-AT 606
84.MK085963.1_AlternariatenuissimaisolateSCCZ06                          0 ---------------------------------------- 0
85.MT548042.1_AlternarialongipesstrainKY_2019_012                        0 ---------------------------------------- 0
86.MN356465.1CalonectriamontanaisolateHSP4                             489 ---------------------------------------- 489
87.OL694224.1_CalonectriacanadianastrainF099                           504 ---------------------------------------- 504
88.MK803351.1_NeoscytalidiumdimidiatumstrainKale4-C                      0 ---------------------------------------- 0
89.ON376993.1_CurvulariachiangmaiensisisolateND00J7                      1 --------------------TGACTGCGCC-ATTCTC-AT 18
90.OQ383346.1_NeoscytalidiumdimidiatumisolateGKH-2                       0 ---------------------------------------- 0
91.MF662595.1_NeoscytalidiumnovaehollandiaeisolateNeNo1                  0 ---------------------------------------- 0
92.EF560588.1Melampsoralini                                            380 ---------------------------------------- 380
93.LC590862.1_NeoscytalidiumdimidiatumPSU-HP01TEF1                       0 ---------------------------------------- 0
94.KX278106.1_BotryosphaeriaqingyuanensisstrainCERC2947                  0 ---------------------------------------- 0
95.AJ578763.1_Blumeriagraminisf.sp.hordeicyp51                           0 ---------------------------------------- 0
96.MF490858.1_CurvulariadactylocteniicolastrainCPC28810                  1 ---------------------------------TCTC-AT 6
97.KT287115.1_Bipolariscactivoraisolate3.8.6                            25 CACT-GGTACCTC-GCAGGCTGACTGTGCC-ATTCTC-AT 60
98.MT560940.1_CurvulariacactivorastrainHLGH0118                          1 ---------------------GACTGTGCC-ATTCTC-AT 17
99.OM714565.1_CurvulariaplantarumstrainM0134                             8 CACT-GGTACCTC-CCAGGCTGACTGCGCC-ATTCTC-AT 43
100.MN159911.1_BotrytiscinereaSICAUCC19-0003                             1 ------------------GCTGATTGTGCC-GTTCTC-AT 20
102.GU294713.1LasiodiplodiatheobromaestrainUCD2430TX                     0 ---------------------------------------- 0
103.KX868094.1_Mycosphaerellasp.isolateCRM20.1                         475 ---------------------------------------- 475
104.LC599478.1Pseudocercosporapini-densifloraeMUCC534                    0 ---------------------------------------- 0
```

```
105.N584698.1BipolarissetariaestrainKBS4-2                          36 CACT-GGTACCTC-CCAGGCTGACTGCGCT-ATCCTC-AT 71

2.OM160859.1_F.buharicum                                           560 ---------------------------------------- 560
1.LC727524.1_F.buharicum_OKI-1_Okura                               682 ---------------------------------------- 682
3.KX302919.1_F.sublunatum                                          521 ---------------------------------------- 521
4.LT996094.1_F.convolutans                                         516 ---------------------------------------- 516
5.OM160861.1_F.abutilonis                                          630 ---------------------------------------- 630
6.OM160874.1_F.guadeloupense                                       560 ---------------------------------------- 560
7.MH392475.1_F.graminearum                                         430 ---------------------------------------- 430
8.MH582420.1_F.solani                                              540 ---------------------------------------- 540
9.MAFF244605_F.oxysporum                                           544 ---------------------------------------- 544
10.MAFF237278_F.contaminatum_Hylocereus                            711 ---------------------------------------- 711
11.MAFF237649_F.concentricum__Riceroot                             678 ---------------------------------------- 678
12.MAFF237650_F.concentricum__Wheat                                679 ---------------------------------------- 679
13.MAFF239869_F.mangiferae__Ryukyupine                             657 ---------------------------------------- 657
14.MAFF240460_F.fujikuroi_Passionfruit                             661 ---------------------------------------- 661
15.MAFF241317_F.graminearum_Wheat                                  666 ---------------------------------------- 666
16.MAFF242670_F.ipomoeae_Wheat                                     672 ---------------------------------------- 672
17.MAFF245129_F.concentricum_Fraxinus                              659 ---------------------------------------- 659
18.MAFF245395_F.cugenangense_Rhubarb                               671 ---------------------------------------- 671
19.MAFF246637_F.nirenbergiae_Strawberry                            671 ---------------------------------------- 671
20.MAFF246672_F.nirenbergiae_ChinesePeony                          671 ---------------------------------------- 671
21.MAFF246697_F.commune_Urallicoriceroot                           673 ---------------------------------------- 673
22.MAFF246729_F.falciforme_Angelica                                691 ---------------------------------------- 691
23.MAFF247220_F.duplospermum__Euwallaceasp                         694 ---------------------------------------- 694
24.MAFF410760_F.odoratissimum_alpha                                669 ---------------------------------------- 669
25.MAFF244605_FusariumoxysporumSchlechtendal_MAFF244605_Tomato     672 ---------------------------------------- 672
26.MAFF241326_F.asiaticum_Wheat                                    669 ---------------------------------------- 669
27.MAFF245014_F.asiaticum_Wildsoybean                              666 ---------------------------------------- 666
28.MAFF150124_F.asiaticum__Wheat                                   666 ---------------------------------------- 666
29.OM135603.1F.algeriense                                          699 ---------------------------------------- 699
30.MAFF237465_F.penzigii_Aloe                                      704 ---------------------------------------- 704
31.MAFF103054_F.oxysporumSchlechtendalf.sp.cucumerinum_Cucumber    671 ---------------------------------------- 671
32.MAFF712246_F.oxysporumSchlechtendalf.sp.dianthi__Carnation      673 ---------------------------------------- 673
33.MAFF305558_F.oxysporumSchlechtendalf.sp.fragariae__Watermelon   671 ---------------------------------------- 671
34.MAFF744087_F.oxysporumSchlechtendalf.sp.lactucae__Lettuce       671 ---------------------------------------- 671
35.MAFF726924_F.oxysporumSchlechtendalf.sp.lagenariae_Whitefloweredgourd 671 ---------------------------------------- 671
36.MAFF744003_F.oxysporumSchlechtendalf.sp.lagenariae_Squash)      672 ---------------------------------------- 672
37.MAFF305122_F.oxysporumSchlechtendalf.sp.melonis__Melon          671 ---------------------------------------- 671
38.MAFF306714_F.oxysporumSchlechtendalf.sp.momordicae_Balsampear   671 ---------------------------------------- 671
39.MAFF238905_F.oxysporumSchlechtendalf.sp.radicis-lycopersici_Tomato 674 ---------------------------------------- 674
40.MAFF150004_F.oxysporumSchlechtendalf.sp.spinaciae_Spinach       671 ---------------------------------------- 671
41.MAFF247034_F.oxysporumSchlechtendal__Goldenchain                671 ---------------------------------------- 671
42.MAFF245747_F.oxysporumSchlechtendalf.sp.callistephi__Chinaaster 671 ---------------------------------------- 671
43.MAFF305115_FoxysporumSchlechtendalf.sp.batatas__Sweatpotato     703 ---------------------------------------- 703
44.MAFF150126_F.asiaticum_Seed                                     666 ---------------------------------------- 666
```

```
45.MAFF246738_F.solani_Angelica                                      703 ---------------------------------------- 703
46.MAFF246664_F.cugenangense_Perilla                                 671 ---------------------------------------- 671
47.MH582420.1F.solanistrainMRC256                                    679 ---------------------------------------- 679
48.MAFF240361_F.babinda_Soil                                         693 ---------------------------------------- 693
49.MAFF242368_F.azukicola_Azukibean                                  708 ---------------------------------------- 708
50.MAFF241312_F.asiaticum_Soil,welshonionfield                       666 ---------------------------------------- 666
51.LT548416.1_F.culmorumpartialtef1a                                 646 ---------------------------------------- 646
52.MAFF150124_F.asiaticum__Wheat                                     666 ---------------------------------------- 666
53.MAFF238806_F.begoniae_Oncidiumsp                                  705 ---------------------------------------- 705
54.MW594399.1_FusariumincarnatumisolateUD01C                         689 ---------------------------------------- 689
55.OP414923.1Pucciniagraminisf.sp.triticiisolateSHZPgt19             344 -CATCGCC-GCCGGTACTGG-TGAATTCGAAGCTGG---T 377
56.MT027094.1_BipolarisoryzaestrainOrL-2                               8 -TATCGCT-GCCGGTACTGG-TGAGTTCGAGGCTGG---T 41
57.ON734360.1_AlternariaalternataisolateH126                           0 ---------------------------------------- 0
58.LC333578.1_StemphyliumlycopersiciSOasp2                             0 ---------------------------------------- 0
59.HQ718583.1_Colletotrichumgloeosporioidesisolateq-1                567 ---------------------------------------- 567
60.JN241603.1_AtheliarolfsiiisolateSR1                               320 ACATCGCCAGGTGGTACTGGGTGAATTCGAGGCTGGGACT 359
61.KJ866474.1_RhizoctoniasolanistrainMHL-1                            59 -CATTGCT-GGCGGTACTGG-TGAATTCGAAGCCGG---T 92
62.JQ672424.1AlternariatriticinaisolateEGS17-061                      260 -CATTGCC-GCCGGTACTGG-TGAGTTCGAGGCTGG---T 293
63.LT707559.1_P.capsicipartialtef1agene                              135 -GGTTGCT-TCGGGTGTGGG-TGAGTTCGAGGCTGG---T 168
64.MW090051.1_CurvularialunatastrainCls-3                              2 -CATTGCC-GCCGGTACTGG-TGAGTTCGAGGCTGG---T 35
65.DQ400892.1_Aspergillusterreus                                      40 -CATTGCC-TCCGGTACTGG-TGAGTTCGAGGCTGG---T 73
66.DQ911416.1_Pythiumsp.quercumstrainPy292                           114 -CGTCGCG-TCGGGCGTCGG-CGAGTTCGAGGCTGG---T 147
67.EU797495.1_Phytophthorasp.oaksoilPoland                           114 -GGTCGCC-TCGGGTGTGGG-CGAGTTCGAGGCTGG---T 147
68.HM148321.1_Cladosporiumcucumerinum                                385 ---------------------------------------- 385
69.AF398888.1_SclerotiniasclerotiorumisolateSS1                        0 ---------------------------------------- 0
70.AF398888.1_S.sclerotiorumisolateSS1                                 0 ---------------------------------------- 0
71.HPAB545908.1_Verticilliumnonalfalfaeisolate                        22 -CATTGCC-GCCGGCACTGG-TGAGTTCGAGGCTGG---T 55
72.EF433315.1_CeratocystisfimbriatavoucherCMW15052                   523 ----------------------GAGTTCGAGGCCGG---T 537
73.MN159912.1_Botrytiscinerea                                         21 -CATTGCC-GCTGGTACTGG-TGAGTTCGAGGCTGG---T 54
74.MF034741.1_PeltasterfructicolaisolateSRB92                          0 ---------------------------------------- 0
75.LC440360.1_CercosporaasparagiCOasp2                                 0 ---------------------------------------- 0
76.AY944105.1_MagnaportheoryzaeisolateSAG00T3()                      255 ---------------------------------------- 255
77.JX266586.1_CochliobolusmiyabeanusvoucherMFLUCC10-0733              17 -TATCGCT-GCCGGTACTGG-TGAGTTCGAGGCTGG---T 50
78.MN393253.1_CorynesporacassiicolaisolateQHD001(MN393253.1UNVERIFIED) 0 ---------------------------------------- 0
79.MF375218.1_AgroatheliarolfsiiisolateBJB24                          40 -CATCGCC-GGTGGTACTGG-TGAATTCGAGGCTGG---T 73
80.MN106270.1_AgroatheliarolfsiistrainJ-12                            47 -CATCGCC-GGTGGTACTGG-TGAATTCGAGGCCGG---T 80
81.OQ732628.1_AgroatheliarolfsiiisolateBTCBSr3                        25 -CATCGCC-GGTGGTACTGG-TGAATTCGAGGCTGG---T 58
82.KY196185.1_ColletotrichumtruncatumstrainPAK53                     468 ---------------------------------------- 468
83.GU935835.1_ColletotrichumcoccodesisolateC96002                    607 -TATCGCT-GCCGGCACTGG-TGAGTTCGAGGCTGG---T 640
84.MK085963.1_AlternariatenuissimaisolateSCCZ06                        0 ---------------------------------------- 0
85.MT548042.1_AlternarialongipesstrainKY_2019_012                      0 ---------------------------------------- 0
86.MN356465.1CalonectriamontanaisolateHSP4                           489 ---------------------------------------- 489
87.OL694224.1_CalonectriacanadianastrainF099                         504 ---------------------------------------- 504
88.MK803351.1_NeoscytalidiumdimidiatumstrainKale4-C                    0 ---------------------------------------- 0
89.ON376993.1_CurvulariachiangmaiensisisolateND00J7                   19 -CATTGCC-GCCGGTACTGG-TGAGTTCGAGGCTGG---T 52
90.OQ383346.1_NeoscytalidiumdimidiatumisolateGKH-2                      0 ---------------------------------------- 0
```

```
91.MF662595.1_NeoscytalidiumnovaehollandiaeisolateNeNo1          0 ---------------------------------------- 0
92.EF560588.1Melampsoralini                                    381 ----------------------GAATTCGAAGCCGG---T 395
93.LC590862.1_NeoscytalidiumdimidiatumPSU-HP01TEF1               0 ---------------------------------------- 0
94.KX278106.1_BotryosphaeriaqingyuanensisstrainCERC2947          0 ---------------------------------------- 0
95.AJ578763.1_Blumeriagraminisf.sp.hordeicyp51                   0 ---------------------------------------- 0
96.MF490858.1_CurvulariadactylocteniicolastrainCPC28810          7 -CATTGCC-GCCGGTACTGG-TGAGTTCGAGGCTGG---T 40
97.KT287115.1_Bipolariscactivoraisolate3.8.6                    61 -CATTGCC-GCCGGTACTGG-TGAGTTCGAGGCTGG---T 94
98.MT560940.1_CurvulariacactivorastrainHLGH0118                 18 -CATTGCC-GCCGGTACTGG-TGAGTTCGAGGCTGG---T 51
99.OM714565.1_CurvulariaplantarumstrainM0134                    44 -CATTGCC-GCCGGTACTGG-TGAGTTCGAGGCTGG---T 77
100.MN159911.1_BotrytiscinereaSICAUCC19-0003                    21 -CATTGCC-GCTGGTACTGG-TGAGTTCGAGGCTGG---T 54
102.GU294713.1LasiodiplodiatheobromaestrainUCD2430TX             0 ---------------------------------------- 0
103.KX868094.1_Mycosphaerellasp.isolateCRM20.1                 475 ---------------------------------------- 475
104.LC599478.1Pseudocercosporapini-densifloraeMUCC534            0 ---------------------------------------- 0
105.N584698.1BipolarissetariaestrainKBS4-2                      72 -TATCGCT-GCCGGTACTGG-TGAGTTCGAGGCTGG---T 105

2.OM160859.1_F.buharicum                                       560 ---------------------------------------- 560
1.LC727524.1_F.buharicum_OKI-1_Okura                           682 ---------------------------------------- 682
3.KX302919.1_F.sublunatum                                      521 ---------------------------------------- 521
4.LT996094.1_F.convolutans                                     516 ---------------------------------------- 516
5.OM160861.1_F.abutilonis                                      630 ---------------------------------------- 630
6.OM160874.1_F.guadeloupense                                   560 ---------------------------------------- 560
7.MH392475.1_F.graminearum                                     430 ---------------------------------------- 430
8.MH582420.1_F.solani                                          540 ---------------------------------------- 540
9.MAFF244605_F.oxysporum                                       544 ---------------------------------------- 544
10.MAFF237278_F.contaminatum_Hylocereus                        711 ---------------------------------------- 711
11.MAFF237649_F.concentricum__Riceroot                         678 ---------------------------------------- 678
12.MAFF237650_F.concentricum__Wheat                            679 ---------------------------------------- 679
13.MAFF239869_F.mangiferae__Ryukyupine                         657 ---------------------------------------- 657
14.MAFF240460_F.fujikuroi_Passionfruit                         661 ---------------------------------------- 661
15.MAFF241317_F.graminearum_Wheat                              666 ---------------------------------------- 666
16.MAFF242670_F.ipomoeae_Wheat                                 672 ---------------------------------------- 672
17.MAFF245129_F.concentricum_Fraxinus                          659 ---------------------------------------- 659
18.MAFF245395_F.cugenangense_Rhubarb                           671 ---------------------------------------- 671
19.MAFF246637_F.nirenbergiae_Strawberry                        671 ---------------------------------------- 671
20.MAFF246672_F.nirenbergiae_ChinesePeony                      671 ---------------------------------------- 671
21.MAFF246697_F.commune_Urallicoriceroot                       673 ---------------------------------------- 673
22.MAFF246729_F.falciforme_Angelica                            691 ---------------------------------------- 691
23.MAFF247220_F.duplospermum__Euwallaceasp                     694 ---------------------------------------- 694
24.MAFF410760_F.odoratissimum_alpha                            669 ---------------------------------------- 669
25.MAFF244605_FusariumoxysporumSchlechtendal_MAFF244605_Tomato 672 ---------------------------------------- 672
26.MAFF241326_F.asiaticum_Wheat                                669 ---------------------------------------- 669
27.MAFF245014_F.asiaticum_Wildsoybean                          666 ---------------------------------------- 666
28.MAFF150124_F.asiaticum__Wheat                               666 ---------------------------------------- 666
29.OM135603.1F.algeriense                                      699 ---------------------------------------- 699
30.MAFF237465_F.penzigii_Aloe                                  704 ---------------------------------------- 704
31.MAFF103054_F.oxysporumSchlechtendalf.sp.cucumerinum_Cucumber 671 ---------------------------------------- 671
```

```
32.MAFF712246_F.oxysporumSchlechtendalf.sp.dianthi__Carnation            673 ---------------------------------------- 673
33.MAFF305558_F.oxysporumSchlechtendalf.sp.fragariae__Watermelon         671 ---------------------------------------- 671
34.MAFF744087_F.oxysporumSchlechtendalf.sp.lactucae__Lettuce             671 ---------------------------------------- 671
35.MAFF726924_F.oxysporumSchlechtendalf.sp.lagenariae_Whitefloweredgourd 671 ---------------------------------------- 671
36.MAFF744003_F.oxysporumSchlechtendalf.sp.lagenariae_Squash)            672 ---------------------------------------- 672
37.MAFF305122_F.oxysporumSchlechtendalf.sp.melonis__Melon                671 ---------------------------------------- 671
38.MAFF306714_F.oxysporumSchlechtendalf.sp.momordicae_Balsampear         671 ---------------------------------------- 671
39.MAFF238905_F.oxysporumSchlechtendalf.sp.radicis-lycopersici_Tomato    674 ---------------------------------------- 674
40.MAFF150004_F.oxysporumSchlechtendalf.sp.spinaciae_Spinach             671 ---------------------------------------- 671
41.MAFF247034_F.oxysporumSchlechtendal__Goldenchain                      671 ---------------------------------------- 671
42.MAFF245747_F.oxysporumSchlechtendalf.sp.callistephi__Chinaaster       671 ---------------------------------------- 671
43.MAFF305115_FoxysporumSchlechtendalf.sp.batatas__Sweatpotato           703 ---------------------------------------- 703
44.MAFF150126_F.asiaticum_Seed                                           666 ---------------------------------------- 666
45.MAFF246738_F.solani_Angelica                                          703 ---------------------------------------- 703
46.MAFF246664_F.cugenangense_Perilla                                     671 ---------------------------------------- 671
47.MH582420.1F.solanistrainMRC256                                        679 ---------------------------------------- 679
48.MAFF240361_F.babinda_Soil                                             693 ---------------------------------------- 693
49.MAFF242368_F.azukicola_Azukibean                                      708 ---------------------------------------- 708
50.MAFF241312_F.asiaticum_Soil,welshonionfield                           666 ---------------------------------------- 666
51.LT548416.1_F.culmorumpartialtef1a                                     646 ---------------------------------------- 646
52.MAFF150124_F.asiaticum__Wheat                                         666 ---------------------------------------- 666
53.MAFF238806_F.begoniae_Oncidiumsp                                      705 ---------------------------------------- 705
54.MW594399.1_FusariumincarnatumisolateUD01C                             689 ---------------------------------------- 689
55.OP414923.1Pucciniagraminisf.sp.triticiisolateSHZPgt19                 378 ATCTCCAAGGATGGCC-AGACTCGTG-AACACGCCCTCCT 415
56.MT027094.1_BipolarisoryzaestrainOrL-2                                  42 ATCTCCAAGGATGGCC-AGACTCGTG-AGCACGCCCTTCT 79
57.ON734360.1_AlternariaalternataisolateH126                                0 ---------------------------------------- 0
58.LC333578.1_StemphyliumlycopersiciSOasp2                                 0 ---------------------------------------- 0
59.HQ718583.1_Colletotrichumgloeosporioidesisolateq-1                    567 ---------------------------------------- 567
60.JN241603.1_AtheliarolfsiiisolateSR1                                   360 ATCTCCAAGGATGGCCCAGACTCGCGGAGCACGCTCTTTT 399
61.KJ866474.1_RhizoctoniasolanistrainMHL-1                                93 ATCTCCAAGGATGGAC-AGACCCGTG-AACACGCTCTCCT 130
62.JQ672424.1AlternariatriticinaisolateEGS17-061                         294 ATCTCCAAGGATGGCC-AGACTCGTG-AGCACGCCCTTCT 331
63.LT707559.1_P.capsicipartialtef1agene                                  169 ATCTCCAAGGAGGGCC-AGACCCGTG-AGCACGCTCTGCT 206
64.MW090051.1_CurvularialunatastrainCls-3                                 36 ATCTCCAAGGATGGTC-AGACTCGTG-AGCACGCTCTGCT 73
65.DQ400892.1_Aspergillusterreus                                          74 ATCTCCAAGGATGGCC-AGACTCGTG-AGCACGCTCTGCT 111
66.DQ911416.1_Pythiumsp.quercumstrainPy292                               148 ATCTCGAAGGAGGGCC-AGACGCGCG-AGCACGCTCTGCT 185
67.EU797495.1_Phytophthorasp.oaksoilPoland                               148 ATCTCCAAGGAGGGCC-AGACGCGCG-AGCACGCTCTGCT 185
68.HM148321.1_Cladosporiumcucumerinum                                    385 ---------------------------------------- 385
69.AF398888.1_SclerotiniasclerotiorumisolateSS1                            0 ---------------------------------------- 0
70.AF398888.1_S.sclerotiorumisolateSS1                                     0 ---------------------------------------- 0
71.HPAB545908.1_Verticilliumnonalfalfaeisolate                            56 ATCTCCAAGGATGGCC-AGACTCGTG-AGCACGCCCTGCT 93
72.EF433315.1_CeratocystisfimbriatavoucherCMW15052                       538 ATCTCCAAGGATGGCC-AGACCCGTG-AGCACGCTCTGCT 575
73.MN159912.1_Botrytiscinerea                                             55 ATCTCCAAGGATGGCC-AAACTCGTG-AGCACGCTCTTCT 92
74.MF034741.1_PeltasterfructicolaisolateSRB92                              0 ---------------------------------------- 0
75.LC440360.1_CercosporaasparagiCOasp2                                     0 ---------------------------------------- 0
76.AY944105.1_MagnaportheoryzaeisolateSAG00T3()                          255 ---------------------------------------- 255
77.JX266586.1_CochliobolusmiyabeanusvoucherMFLUCC10-0733                  51 ATCTCCAAGGATGGCC-AGACTCGTG-AGCACGCCCTTCT 88
```

```
78.MN393253.1_CorynesporacassiicolaisolateQHD001(MN393253.1UNVERIFIED)      0 ---------------------------------------- 0
79.MF375218.1_AgroatheliarolfsiiisolateBJB24                               74 ATCTCCAAGGATGGCC-AGACTCGCG-AGCACGCTCTTTT 111
80.MN106270.1_AgroatheliarolfsiistrainJ-12                                 81 ATCTCCAAGGATGGCC-AGACTCGCG-AGCACGCTCTTTT 118
81.OQ732628.1_AgroatheliarolfsiiisolateBTCBSr3                             59 ATCTCCAAGGATGGCC-AGACTCGCG-AGCACGCTCTTTT 96
82.KY196185.1_ColletotrichumtruncatumstrainPAK53                          468 ---------------------------------------- 468
83.GU935835.1_ColletotrichumcoccodesisolateC96002                         641 ATCTCCAAGGATGGCC-AGACCCGTG-AGCACGCTCTGCT 678
84.MK085963.1_AlternariatenuissimaisolateSCCZ06                             0 ---------------------------------------- 0
85.MT548042.1_AlternarialongipesstrainKY_2019_012                           0 ---------------------------------------- 0
86.MN356465.1CalonectriamontanaisolateHSP4                                489 ---------------------------------------- 489
87.OL694224.1_CalonectriacanadianastrainF099                              504 ---------------------------------------- 504
88.MK803351.1_NeoscytalidiumdimidiatumstrainKale4-C                         0 ---------------------------------------- 0
89.ON376993.1_CurvulariachiangmaiensisisolateND00J7                        53 ATCTCCAAGGATGGTC-AGACTCGTG-AGCACGCTCTGCT 90
90.OQ383346.1_NeoscytalidiumdimidiatumisolateGKH-2                          0 ---------------------------------------- 0
91.MF662595.1_NeoscytalidiumnovaehollandiaeisolateNeNo1                     0 ---------------------------------------- 0
92.EF560588.1Melampsoralini                                               396 ATCTCCAAGGATGGTC-AAACTCGTG-AACATGCTCTTCT 433
93.LC590862.1_NeoscytalidiumdimidiatumPSU-HP01TEF1                          0 ---------------------------------------- 0
94.KX278106.1_BotryosphaeriaqingyuanensisstrainCERC2947                     0 ---------------------------------------- 0
95.AJ578763.1_Blumeriagraminisf.sp.hordeicyp51                              0 ---------------------------------------- 0
96.MF490858.1_CurvulariadactylocteniicolastrainCPC28810                    41 ATCTCCAAGGATGGTC-AGACTCGTG-AGCACGCTCTGCT 78
97.KT287115.1_Bipolariscactivoraisolate3.8.6                               95 ATCTCCAAGGATGGTC-AGACCCGTG-AGCACGCTCTGCT 132
98.MT560940.1_CurvulariacactivorastrainHLGH0118                            52 ATCTCCAAGGATGGTC-AGACCCGTG-AGCACGCTCTGCT 89
99.OM714565.1_CurvulariaplantarumstrainM0134                               78 ATCTCCAAGGATGGTC-AGACTCGTG-AGCACGCTCTGCT 115
100.MN159911.1_BotrytiscinereaSICAUCC19-0003                               55 ATCTCCAAGGATGGCC-AAACTCGTG-AGCACGCTCTTCT 92
102.GU294713.1LasiodiplodiatheobromaestrainUCD2430TX                         0 ---------------------------------------- 0
103.KX868094.1_Mycosphaerellasp.isolateCRM20.1                            475 ---------------------------------------- 475
104.LC599478.1Pseudocercosporapini-densifloraeMUCC534                       0 ---------------------------------------- 0
105.N584698.1BipolarissetariaestrainKBS4-2                                106 ATCTCCAAGGATGGCC-AGACTCGTG-AGCACGCCCTTCT 143

2.OM160859.1_F.buharicum                                                  560 ---------------------------------------- 560
1.LC727524.1_F.buharicum_OKI-1_Okura                                      682 ---------------------------------------- 682
3.KX302919.1_F.sublunatum                                                 521 ---------------------------------------- 521
4.LT996094.1_F.convolutans                                                516 ---------------------------------------- 516
5.OM160861.1_F.abutilonis                                                 630 ---------------------------------------- 630
6.OM160874.1_F.guadeloupense                                              560 ---------------------------------------- 560
7.MH392475.1_F.graminearum                                                430 ---------------------------------------- 430
8.MH582420.1_F.solani                                                     540 ---------------------------------------- 540
9.MAFF244605_F.oxysporum                                                  544 ---------------------------------------- 544
10.MAFF237278_F.contaminatum_Hylocereus                                   711 ---------------------------------------- 711
11.MAFF237649_F.concentricum__Riceroot                                    678 ---------------------------------------- 678
12.MAFF237650_F.concentricum__Wheat                                       679 ---------------------------------------- 679
13.MAFF239869_F.mangiferae__Ryukyupine                                    657 ---------------------------------------- 657
14.MAFF240460_F.fujikuroi_Passionfruit                                    661 ---------------------------------------- 661
15.MAFF241317_F.graminearum_Wheat                                         666 ---------------------------------------- 666
16.MAFF242670_F.ipomoeae_Wheat                                            672 ---------------------------------------- 672
17.MAFF245129_F.concentricum_Fraxinus                                     659 ---------------------------------------- 659
18.MAFF245395_F.cugenangense_Rhubarb                                      671 ---------------------------------------- 671
```

```
19.MAFF246637_F.nirenbergiae_Strawberry                            671 ---------------------------------------- 671
20.MAFF246672_F.nirenbergiae_ChinesePeony                          671 ---------------------------------------- 671
21.MAFF246697_F.commune_Urallicoriceroot                           673 ---------------------------------------- 673
22.MAFF246729_F.falciforme_Angelica                                691 ---------------------------------------- 691
23.MAFF247220_F.duplospermum__Euwallaceasp                         694 ---------------------------------------- 694
24.MAFF410760_F.odoratissimum_alpha                                669 ---------------------------------------- 669
25.MAFF244605_FusariumoxysporumSchlechtendal_MAFF244605_Tomato     672 ---------------------------------------- 672
26.MAFF241326_F.asiaticum_Wheat                                    669 ---------------------------------------- 669
27.MAFF245014_F.asiaticum_Wildsoybean                              666 ---------------------------------------- 666
28.MAFF150124_F.asiaticum__Wheat                                   666 ---------------------------------------- 666
29.OM135603.1F.algeriense                                          699 ---------------------------------------- 699
30.MAFF237465_F.penzigii_Aloe                                      704 ---------------------------------------- 704
31.MAFF103054_F.oxysporumSchlechtendalf.sp.cucumerinum_Cucumber    671 ---------------------------------------- 671
32.MAFF712246_F.oxysporumSchlechtendalf.sp.dianthi__Carnation      673 ---------------------------------------- 673
33.MAFF305558_F.oxysporumSchlechtendalf.sp.fragariae__Watermelon   671 ---------------------------------------- 671
34.MAFF744087_F.oxysporumSchlechtendalf.sp.lactucae__Lettuce       671 ---------------------------------------- 671
35.MAFF726924_F.oxysporumSchlechtendalf.sp.lagenariae_Whiteflower  671 ---------------------------------------- 671
edgourd
36.MAFF744003_F.oxysporumSchlechtendalf.sp.lagenariae_Squash)      672 ---------------------------------------- 672
37.MAFF305122_F.oxysporumSchlechtendalf.sp.melonis__Melon          671 ---------------------------------------- 671
38.MAFF306714_F.oxysporumSchlechtendalf.sp.momordicae_Balsampear   671 ---------------------------------------- 671
39.MAFF238905_F.oxysporumSchlechtendalf.sp.radicis-lycopersici_Tomato 674 ---------------------------------------- 674
40.MAFF150004_F.oxysporumSchlechtendalf.sp.spinaciae_Spinach       671 ---------------------------------------- 671
41.MAFF247034_F.oxysporumSchlechtendal__Goldenchain                671 ---------------------------------------- 671
42.MAFF245747_F.oxysporumSchlechtendalf.sp.callistephi__Chinaaster 671 ---------------------------------------- 671
43.MAFF305115_FoxysporumSchlechtendalf.sp.batatas__Sweatpotato     703 ---------------------------------------- 703
44.MAFF150126_F.asiaticum_Seed                                     666 ---------------------------------------- 666
45.MAFF246738_F.solani_Angelica                                    703 ---------------------------------------- 703
46.MAFF246664_F.cugenangense_Perilla                               671 ---------------------------------------- 671
47.MH582420.1F.solanistrainMRC256                                  679 ---------------------------------------- 679
48.MAFF240361_F.babinda_Soil                                       693 ---------------------------------------- 693
49.MAFF242368_F.azukicola_Azukibean                                708 ---------------------------------------- 708
50.MAFF241312_F.asiaticum_Soil,welshonionfield                     666 ---------------------------------------- 666
51.LT548416.1_F.culmorumpartialtef1a                               646 ---------------------------------------- 646
52.MAFF150124_F.asiaticum__Wheat                                   666 ---------------------------------------- 666
53.MAFF238806_F.begoniae_Oncidiumsp                                705 ---------------------------------------- 705
54.MW594399.1_FusariumincarnatumisolateUD01C                       689 ---------------------------------------- 689
55.OP414923.1Pucciniagraminisf.sp.triticiisolateSHZPgt19           416 AGCCTTCACCCTTGGTGTCCGACAACTCATCGTTGCCATC 455
56.MT027094.1_BipolarisoryzaestrainOrL-2                            80 CGCCTACACCCTTGGTGTCAAGCAGCTCATCGTTGCCATC 119
57.ON734360.1_AlternariaalternataisolateH126                         0 ---------------------------------------- 0
58.LC333578.1_StemphyliumlycopersiciSOasp2                           0 ---------------------------------------- 0
59.HQ718583.1_Colletotrichumgloeosporioidesisolateq-1              567 ---------------------------------------- 567
60.JN241603.1_AtheliarolfsiiisolateSR1                             400 GGCTTTCACCCTCGGTGTCCGTCAGCTCATCGTCGCTGTC 439
61.KJ866474.1_RhizoctoniasolanistrainMHL-1                         131 TGCCTTCACCCTTGGTGTGCGTCAGCTCATTGTCGCCGTC 170
62.JQ672424.1AlternariatriticinaisolateEGS17-061                    332 CGCCTACACCCTCGGTGTCAAGCAGCTCATCGTTGCCATC 371
63.LT707559.1_P.capsicipartialtef1agene                            207 CGCTTTCACCCTGGGTGTGAAGCAGATGATCGTTGCCATC 246
64.MW090051.1_CurvularialunatastrainCls-3                           74 CGCCTACACCCTCGGTGTCAAGCAGCTCATCGTCGCCATC 113
```

65.DQ400892.1_Aspergillusterreus	112	TGCCTTCACCCTCGGTGTCCGTCAGCTCATTGTTGCCCTC	151
66.DQ911416.1_Pythiumsp.quercumstrainPy292	186	TGCGTTCACGCTTGGCGTGAAGCAGATGATCGTGGCCATC	225
67.EU797495.1_Phytophthorasp.oaksoilPoland	186	TGCCTTCACCCTGGGTGTGAAGCAGATGATCGTCGCCATC	225
68.HM148321.1_Cladosporiumcucumerinum	385	--	385
69.AF398888.1_SclerotiniasclerotiorumisolateSS1	0	--	0
70.AF398888.1_S.sclerotiorumisolateSS1	0	--	0
71.HPAB545908.1_Verticilliumnonalfalfaeisolate	94	CGCCTACACCCTTGGTGTCAAGCAGCTCATCGTCGCCATC	133
72.EF433315.1_CeratocystisfimbriatavoucherCMW15052	576	GGCTTTCACCCTCGGTGTCAAGCAGCTGATTGTTGCCATC	615
73.MN159912.1_Botrytiscinerea	93	TGCCTACACCCTTGGTGTCAAGCAACTCATTGTTGCCATC	132
74.MF034741.1_PeltasterfructicolaisolateSRB92	0	--	0
75.LC440360.1_CercosporaasparagiCOasp2	0	--	0
76.AY944105.1_MagnaportheoryzaeisolateSAG00T3()	255	--	255
77.JX266586.1_CochliobolusmiyabeanusvoucherMFLUCC10-0733	89	CGCCTACACCCTTGGTGTCAAGCAGCTCATCGTTGCCATC	128
78.MN393253.1_CorynesporacassiicolaisolateQHD001(MN393253.1UNVERIFIED)	0	--	0
79.MF375218.1_AgroatheliarolfsiiisolateBJB24	112	GGCTTTCACCCTCGGTGTCCGTCAGCTCATCGTCGCTGTC	151
80.MN106270.1_AgroatheliarolfsiistrainJ-12	119	GGCTTTCACCCTCGGTGTCCGTCAGCTCATCGTCGCTGTC	158
81.OQ732628.1_AgroatheliarolfsiiisolateBTCBSr3	97	GGCTTTCACCCTCGGTGTCCGTCAGCTCATCGTCGCTGTC	136
82.KY196185.1_ColletotrichumtruncatumstrainPAK53	468	--	468
83.GU935835.1_ColletotrichumcoccodesisolateC96002	679	CGCCTACACCCTCGGTGTCAAGCAGCTCATTGTTGCCATC	718
84.MK085963.1_AlternariatenuissimaisolateSCCZ06	0	--	0
85.MT548042.1_AlternarialongipesstrainKY_2019_012	0	--	0
86.MN356465.1CalonectriamontanaisolateHSP4	489	--	489
87.OL694224.1_CalonectriacanadianastrainF099	504	--	504
88.MK803351.1_NeoscytalidiumdimidiatumstrainKale4-C	0	--	0
89.ON376993.1_CurvulariachiangmaiensisisolateND00J7	91	CGCCTACACCCTCGGTGTCAAGCAGCTCATCGTCGCCATC	130
90.OQ383346.1_NeoscytalidiumdimidiatumisolateGKH-2	0	--	0
91.MF662595.1_NeoscytalidiumnovaehollandiaeisolateNeNo1	0	--	0
92.EF560588.1Melampsoralini	434	TGCCTTTACTCTCGGTGTCCGTCAACTCATCGTGGCCATC	473
93.LC590862.1_NeoscytalidiumdimidiatumPSU-HP01TEF1	0	--	0
94.KX278106.1_BotryosphaeriaqingyuanensisstrainCERC2947	0	--	0
95.AJ578763.1_Blumeriagraminisf.sp.hordeicyp51	0	--	0
96.MF490858.1_CurvulariadactylocteniicolastrainCPC28810	79	CGCCTACACCCTCGGTGTCAAGCAGCTCATCGTCGCCATC	118
97.KT287115.1_Bipolariscactivoraisolate3.8.6	133	CGCCTACACCCTCGGTGTCAAGCAGCTCATCGTTGCCATC	172
98.MT560940.1_CurvulariacactivorastrainHLGH0118	90	CGCCTACACCCTCGGTGTCAAGCAGCTCATCGTTGCCATC	129
99.OM714565.1_CurvulariaplantarumstrainM0134	116	CGCCTACACCCTCGGTGTCAAGCAGCTCATCGTCGCCATC	155
100.MN159911.1_BotrytiscinereaSICAUCC19-0003	93	TGCCTACACCCTTGGTGTCAAGCAACTCATTGTTGCCATC	132
102.GU294713.1LasiodiplodiatheobromaestrainUCD2430TX	0	--	0
103.KX868094.1_Mycosphaerellasp.isolateCRM20.1	475	--	475
104.LC599478.1Pseudocercosporapini-densifloraeMUCC534	0	--	0
105.N584698.1BipolarissetariaestrainKBS4-2	144	CGCCTACACCCTTGGTGTCAAGCAGCTCATCGTTGCCATC	183
2.OM160859.1_F.buharicum	560	--	560
1.LC727524.1_F.buharicum_OKI-1_Okura	682	--	682
3.KX302919.1_F.sublunatum	521	--	521
4.LT996094.1_F.convolutans	516	--	516
5.OM160861.1_F.abutilonis	630	--	630

```
6.OM160874.1_F.guadeloupense                                          560 ---------------------------------------- 560
7.MH392475.1_F.graminearum                                            430 ---------------------------------------- 430
8.MH582420.1_F.solani                                                 540 ---------------------------------------- 540
9.MAFF244605_F.oxysporum                                              544 ---------------------------------------- 544
10.MAFF237278_F.contaminatum_Hylocereus                               711 ---------------------------------------- 711
11.MAFF237649_F.concentricum__Riceroot                                678 ---------------------------------------- 678
12.MAFF237650_F.concentricum__Wheat                                   679 ---------------------------------------- 679
13.MAFF239869_F.mangiferae__Ryukyupine                                657 ---------------------------------------- 657
14.MAFF240460_F.fujikuroi_Passionfruit                                661 ---------------------------------------- 661
15.MAFF241317_F.graminearum_Wheat                                     666 ---------------------------------------- 666
16.MAFF242670_F.ipomoeae_Wheat                                        672 ---------------------------------------- 672
17.MAFF245129_F.concentricum_Fraxinus                                 659 ---------------------------------------- 659
18.MAFF245395_F.cugenangense_Rhubarb                                  671 ---------------------------------------- 671
19.MAFF246637_F.nirenbergiae_Strawberry                               671 ---------------------------------------- 671
20.MAFF246672_F.nirenbergiae_ChinesePeony                             671 ---------------------------------------- 671
21.MAFF246697_F.commune_Urallicoriceroot                              673 ---------------------------------------- 673
22.MAFF246729_F.falciforme_Angelica                                   691 ---------------------------------------- 691
23.MAFF247220_F.duplospermum__Euwallaceasp                            694 ---------------------------------------- 694
24.MAFF410760_F.odoratissimum_alpha                                   669 ---------------------------------------- 669
25.MAFF244605_FusariumoxysporumSchlechtendal_MAFF244605_Tomato        672 ---------------------------------------- 672
26.MAFF241326_F.asiaticum_Wheat                                       669 ---------------------------------------- 669
27.MAFF245014_F.asiaticum_Wildsoybean                                 666 ---------------------------------------- 666
28.MAFF150124_F.asiaticum__Wheat                                      666 ---------------------------------------- 666
29.OM135603.1F.algeriense                                             699 ---------------------------------------- 699
30.MAFF237465_F.penzigii_Aloe                                         704 ---------------------------------------- 704
31.MAFF103054_F.oxysporumSchlechtendalf.sp.cucumerinum_Cucumber       671 ---------------------------------------- 671
32.MAFF712246_F.oxysporumSchlechtendalf.sp.dianthi__Carnation         673 ---------------------------------------- 673
33.MAFF305558_F.oxysporumSchlechtendalf.sp.fragariae__Watermelon      671 ---------------------------------------- 671
34.MAFF744087_F.oxysporumSchlechtendalf.sp.lactucae__Lettuce          671 ---------------------------------------- 671
35.MAFF726924_F.oxysporumSchlechtendalf.sp.lagenariae_Whitefloweredgourd 671 ---------------------------------------- 671
36.MAFF744003_F.oxysporumSchlechtendalf.sp.lagenariae_Squash)         672 ---------------------------------------- 672
37.MAFF305122_F.oxysporumSchlechtendalf.sp.melonis__Melon             671 ---------------------------------------- 671
38.MAFF306714_F.oxysporumSchlechtendalf.sp.momordicae_Balsampear      671 ---------------------------------------- 671
39.MAFF238905_F.oxysporumSchlechtendalf.sp.radicis-lycopersici_Tomato 674 ---------------------------------------- 674
40.MAFF150004_F.oxysporumSchlechtendalf.sp.spinaciae_Spinach          671 ---------------------------------------- 671
41.MAFF247034_F.oxysporumSchlechtendal__Goldenchain                   671 ---------------------------------------- 671
42.MAFF245747_F.oxysporumSchlechtendalf.sp.callistephi__Chinaaster    671 ---------------------------------------- 671
43.MAFF305115_FoxysporumSchlechtendalf.sp.batatas__Sweatpotato        703 ---------------------------------------- 703
44.MAFF150126_F.asiaticum_Seed                                        666 ---------------------------------------- 666
45.MAFF246738_F.solani_Angelica                                       703 ---------------------------------------- 703
46.MAFF246664_F.cugenangense_Perilla                                  671 ---------------------------------------- 671
47.MH582420.1F.solanistrainMRC256                                     679 ---------------------------------------- 679
48.MAFF240361_F.babinda_Soil                                          693 ---------------------------------------- 693
49.MAFF242368_F.azukicola_Azukibean                                   708 ---------------------------------------- 708
50.MAFF241312_F.asiaticum_Soil,welshonionfield                        666 ---------------------------------------- 666
51.LT548416.1_F.culmorumpartialtef1a                                  646 ---------------------------------------- 646
```

```
52.MAFF150124_F.asiaticum__Wheat                                     666 ---------------------------------------- 666
53.MAFF238806_F.begoniae_Oncidiumsp                                  705 ---------------------------------------- 705
54.MW594399.1_FusariumincarnatumisolateUD01C                         689 ---------------------------------------- 689
55.OP414923.1Pucciniagraminisf.sp.triticiisolateSHZPgt19             456 AAC-AAGATGGAC------ACCACCAAATGGTCCGAGCAG 488
56.MT027094.1_BipolarisoryzaestrainOrL-2                             120 AAC-AAGATGGAC------ACCACCAAGTGGTCTGAGGAC 152
57.ON734360.1_AlternariaalternataisolateH126                           1 -------ATGGAC------ACCACCAAGTGGTCCGAGGAG 27
58.LC333578.1_StemphyliumlycopersiciSOasp2                             0 ---------------------------------------- 0
59.HQ718583.1_Colletotrichumgloeosporioidesisolateq-1                567 ---------------------------------------- 567
60.JN241603.1_AtheliarolfsiiisolateSR1                               440 AACGAAGATGGACACCACCTA------------------- 460
61.KJ866474.1_RhizoctoniasolanistrainMHL-1                           171 AAC-AAGATGGACACCACC-AAGGTATGCTATTTGCCGTT 208
62.JQ672424.1AlternariatriticinaisolateEGS17-061                     372 AAC-AAGATGGAC------ACTACCAAGTGGTCCGAGGAG 404
63.LT707559.1_P.capsicipartialtef1agene                              247 AAC-AAGATGGACGACTCGTCTGTCATGTACGGCCAGGCC 285
64.MW090051.1_CurvularialunatastrainCls-3                            114 AAC-AAGATGGAC------ACCACCAAGTGGTCTGAGGAG 146
65.DQ400892.1_Aspergillusterreus                                     152 AAC-AAGATGGAC------ACCTGCAAGTGGTCCGAGGAC 184
66.DQ911416.1_Pythiumsp.quercumstrainPy292                           226 AAC-AAGATGGACGACTCGTCGGTGATGTACGGCGAGGGC 264
67.EU797495.1_Phytophthorasp.oaksoilPoland                           226 AAC-AAGATGGACGACTCGTCTGTCATGTACGGCCAGGCC 264
68.HM148321.1_Cladosporiumcucumerinum                                385 ---------------------------------------- 385
69.AF398888.1_SclerotiniasclerotiorumisolateSS1                        0 ---------------------------------------- 0
70.AF398888.1_S.sclerotiorumisolateSS1                                 0 ---------------------------------------- 0
71.HPAB545908.1_Verticilliumnonalfalfaeisolate                       134 AAC-AAGATGGAC------ACCACCAAGTGGTCCGAGGAG 166
72.EF433315.1_CeratocystisfimbriatavoucherCMW15052                   616 AAC-AAGATGGAC------ACCGCCAAGTGGGCTGAGGCT 648
73.MN159912.1_Botrytiscinerea                                        133 AAC-AAGATGGAC------ACCACCAAGTGGTCCGAGGAT 165
74.MF034741.1_PeltasterfructicolaisolateSRB92                          0 ---------------------------------------- 0
75.LC440360.1_CercosporaasparagiCOasp2                                 0 ---------------------------------------- 0
76.AY944105.1_MagnaportheoryzaeisolateSAG00T3()                      255 ---------------------------------------- 255
77.JX266586.1_CochliobolusmiyabeanusvoucherMFLUCC10-0733             129 AAC-AAGATGGAC------ACCACCAAGTGGTCTGAGGAC 161
78.MN393253.1_CorynesporacassiicolaisolateQHD001(MN393253.1UNVERIFIED)  0 ---------------------------------------- 0
79.MF375218.1_AgroatheliarolfsiiisolateBJB24                         152 AAC-AAGATGGACACCACC-A------------------- 170
80.MN106270.1_AgroatheliarolfsiistrainJ-12                           159 AAC-AAGATGGACACCACC-A------------------- 177
81.OQ732628.1_AgroatheliarolfsiiisolateBTCBSr3                       137 AAC-AAGATGGACACCACC-A------------------- 155
82.KY196185.1_ColletotrichumtruncatumstrainPAK53                     468 ---------------------------------------- 468
83.GU935835.1_ColletotrichumcoccodesisolateC96002                    719 AAC-AAGATGGAC------ACCACCAAGTGGTCCGAGGCC 751
84.MK085963.1_AlternariatenuissimaisolateSCCZ06                        0 ---------------------------------------- 0
85.MT548042.1_AlternarialongipesstrainKY_2019_012                      0 ---------------------------------------- 0
86.MN356465.1CalonectriamontanaisolateHSP4                           489 ---------------------------------------- 489
87.OL694224.1_CalonectriacanadianastrainF099                         504 ---------------------------------------- 504
88.MK803351.1_NeoscytalidiumdimidiatumstrainKale4-C                    0 ---------------------------------------- 0
89.ON376993.1_CurvulariachiangmaiensisisolateND00J7                  131 AAC-AAGATGGAC------ACCACCAAGTGGTCTGAGGAG 163
90.OQ383346.1_NeoscytalidiumdimidiatumisolateGKH-2                     0 ---------------------------------------- 0
91.MF662595.1_NeoscytalidiumnovaehollandiaeisolateNeNo1                0 ---------------------------------------- 0
92.EF560588.1Melampsoralini                                          474 AAC-AAGATGGAC------ACCTGCAAGTGGTCCGAGCAG 506
93.LC590862.1_NeoscytalidiumdimidiatumPSU-HP01TEF1                     0 ---------------------------------------- 0
94.KX278106.1_BotryosphaeriaqingyuanensisstrainCERC2947                0 ---------------------------------------- 0
95.AJ578763.1_Blumeriagraminisf.sp.hordeicyp51                         0 ---------------------------------------- 0
96.MF490858.1_CurvulariadactylocteniicolastrainCPC28810              119 AAC-AAGATGGAC------ACCACCAAGTGGTCTGAGGAG 151
97.KT287115.1_Bipolariscactivoraisolate3.8.6                         173 AAC-AAGATGGAC------ACCACCAAGTGGTCCGAGGAC 205
```

```
98.MT560940.1_CurvulariacactivorastrainHLGH0118                130 AAC-AAGATGGAC------ACCACCAAGTGGTCCGAGGAC 162
99.OM714565.1_CurvulariaplantarumstrainM0134                   156 AAC-AAGATGGAC------ACCACCAAGTGGTCTGAGGAC 188
100.MN159911.1_BotrytiscinereaSICAUCC19-0003                   133 AAC-AAGATGGAC------ACCACCAAGTGGTCCGAGGAT 165
102.GU294713.1LasiodiplodiatheobromaestrainUCD2430TX             0 ---------------------------------------- 0
103.KX868094.1_Mycosphaerellasp.isolateCRM20.1                 475 ---------------------------------------- 475
104.LC599478.1Pseudocercosporapini-densifloraeMUCC534            0 ---------------------------------------- 0
105.N584698.1BipolarissetariaestrainKBS4-2                     184 AAC-AAGATGGAC------ACCACCAAGTGGTCTGAGGAC 216

2.OM160859.1_F.buharicum                                       560 ---------------------------------------- 560
1.LC727524.1_F.buharicum_OKI-1_Okura                           682 ---------------------------------------- 682
3.KX302919.1_F.sublunatum                                      521 ---------------------------------------- 521
4.LT996094.1_F.convolutans                                     516 ---------------------------------------- 516
5.OM160861.1_F.abutilonis                                      630 ---------------------------------------- 630
6.OM160874.1_F.guadeloupense                                   560 ---------------------------------------- 560
7.MH392475.1_F.graminearum                                     430 ---------------------------------------- 430
8.MH582420.1_F.solani                                          540 ---------------------------------------- 540
9.MAFF244605_F.oxysporum                                       544 ---------------------------------------- 544
10.MAFF237278_F.contaminatum_Hylocereus                        711 ---------------------------------------- 711
11.MAFF237649_F.concentricum__Riceroot                         678 ---------------------------------------- 678
12.MAFF237650_F.concentricum__Wheat                            679 ---------------------------------------- 679
13.MAFF239869_F.mangiferae__Ryukyupine                         657 ---------------------------------------- 657
14.MAFF240460_F.fujikuroi_Passionfruit                         661 ---------------------------------------- 661
15.MAFF241317_F.graminearum_Wheat                              666 ---------------------------------------- 666
16.MAFF242670_F.ipomoeae_Wheat                                 672 ---------------------------------------- 672
17.MAFF245129_F.concentricum_Fraxinus                          659 ---------------------------------------- 659
18.MAFF245395_F.cugenangense_Rhubarb                           671 ---------------------------------------- 671
19.MAFF246637_F.nirenbergiae_Strawberry                        671 ---------------------------------------- 671
20.MAFF246672_F.nirenbergiae_ChinesePeony                      671 ---------------------------------------- 671
21.MAFF246697_F.commune_Urallicoriceroot                       673 ---------------------------------------- 673
22.MAFF246729_F.falciforme_Angelica                            691 ---------------------------------------- 691
23.MAFF247220_F.duplospermum__Euwallaceasp                     694 ---------------------------------------- 694
24.MAFF410760_F.odoratissimum_alpha                            669 ---------------------------------------- 669
25.MAFF244605_FusariumoxysporumSchlechtendal_MAFF244605_Tomato 672 ---------------------------------------- 672
26.MAFF241326_F.asiaticum_Wheat                                669 ---------------------------------------- 669
27.MAFF245014_F.asiaticum_Wildsoybean                          666 ---------------------------------------- 666
28.MAFF150124_F.asiaticum__Wheat                               666 ---------------------------------------- 666
29.OM135603.1F.algeriense                                      699 ---------------------------------------- 699
30.MAFF237465_F.penzigii_Aloe                                  704 ---------------------------------------- 704
31.MAFF103054_F.oxysporumSchlechtendalf.sp.cucumerinum_Cucumber 671 ---------------------------------------- 671
32.MAFF712246_F.oxysporumSchlechtendalf.sp.dianthi__Carnation  673 ---------------------------------------- 673
33.MAFF305558_F.oxysporumSchlechtendalf.sp.fragariae__Watermelon 671 ---------------------------------------- 671
34.MAFF744087_F.oxysporumSchlechtendalf.sp.lactucae__Lettuce   671 ---------------------------------------- 671
35.MAFF726924_F.oxysporumSchlechtendalf.sp.lagenariae_Whitefloweredgourd 671 ---------------------------------------- 671
36.MAFF744003_F.oxysporumSchlechtendalf.sp.lagenariae_Squash)  672 ---------------------------------------- 672
37.MAFF305122_F.oxysporumSchlechtendalf.sp.melonis__Melon      671 ---------------------------------------- 671
38.MAFF306714_F.oxysporumSchlechtendalf.sp.momordicae_Balsampear 671 ---------------------------------------- 671
```

```
39.MAFF238905_F.oxysporumSchlechtendalf.sp.radicis-lycopersici_Tomato     674 ---------------------------------------- 674
40.MAFF150004_F.oxysporumSchlechtendalf.sp.spinaciae_Spinach              671 ---------------------------------------- 671
41.MAFF247034_F.oxysporumSchlechtendal__Goldenchain                       671 ---------------------------------------- 671
42.MAFF245747_F.oxysporumSchlechtendalf.sp.callistephi__Chinaaster        671 ---------------------------------------- 671
43.MAFF305115_FoxysporumSchlechtendalf.sp.batatas__Sweatpotato            703 ---------------------------------------- 703
44.MAFF150126_F.asiaticum_Seed                                            666 ---------------------------------------- 666
45.MAFF246738_F.solani_Angelica                                           703 ---------------------------------------- 703
46.MAFF246664_F.cugenangense_Perilla                                      671 ---------------------------------------- 671
47.MH582420.1F.solanistrainMRC256                                         679 ---------------------------------------- 679
48.MAFF240361_F.babinda_Soil                                              693 ---------------------------------------- 693
49.MAFF242368_F.azukicola_Azukibean                                       708 ---------------------------------------- 708
50.MAFF241312_F.asiaticum_Soil,welshonionfield                            666 ---------------------------------------- 666
51.LT548416.1_F.culmorumpartialtef1a                                      646 ---------------------------------------- 646
52.MAFF150124_F.asiaticum__Wheat                                          666 ---------------------------------------- 666
53.MAFF238806_F.begoniae_Oncidiumsp                                       705 ---------------------------------------- 705
54.MW594399.1_FusariumincarnatumisolateUD01C                              689 ---------------------------------------- 689
55.OP414923.1Pucciniagraminisf.sp.triticiisolateSHZPgt19                  489 AGGTTTGTTTTTTTATCCCCTCTTCACTCCTTGCCCCTCT 528
56.MT027094.1_BipolarisoryzaestrainOrL-2                                  153 CGTTACCAGGAGATCATCAAGGAGACCTCCAACTTCATCA 192
57.ON734360.1_AlternariaalternataisolateH126                               28 CGTTACCAGGAGATCATCAAGGAGACCTCCAACTTCATCA 67
58.LC333578.1_StemphyliumlycopersiciSOasp2                                  0 ---------------------------------------- 0
59.HQ718583.1_Colletotrichumgloeosporioidesisolateq-1                     567 ---------------------------------------- 567
60.JN241603.1_AtheliarolfsiiisolateSR1                                    461 ----------------------------------AGTGGA 466
61.KJ866474.1_RhizoctoniasolanistrainMHL-1                                209 TATGGAAGTGGAGTTGATACTGATGGTGTTATATAGTGGA 248
62.JQ672424.1AlternariatriticinaisolateEGS17-061                          405 CGTTACCAGGAGATCATCAAGGAGACCTCCAACTTCATCA 444
63.LT707559.1_P.capsicipartialtef1agene                                   286 CGTTACGAGGAGATCAAGTCTGAGGTGACTACCTACCTGA 325
64.MW090051.1_CurvularialunatastrainCls-3                                 147 CGTTACCAGGAAATCATCAAGGAGACCTCCAACTTCATCA 186
65.DQ400892.1_Aspergillusterreus                                          185 CGTTACAACGAAATCGTGAAGGAGACCTCCAACTTCATCA 224
66.DQ911416.1_Pythiumsp.quercumstrainPy292                                265 CGCTACACGGAGATCAAGAACGAGGTGACGGCGTACCTGA 304
67.EU797495.1_Phytophthorasp.oaksoilPoland                                265 CGTTACGAGGAGATCAAGGCTGAGGTCACTACCTACCTGA 304
68.HM148321.1_Cladosporiumcucumerinum                                     385 ---------------------------------------- 385
69.AF398888.1_SclerotiniasclerotiorumisolateSS1                             0 ---------------------------------------- 0
70.AF398888.1_S.sclerotiorumisolateSS1                                      0 ---------------------------------------- 0
71.HPAB545908.1_Verticilliumnonalfalfaeisolate                            167 CGTTTCACTGAGATCATCAAGGAGACCACCAACTTCATCA 206
72.EF433315.1_CeratocystisfimbriatavoucherCMW15052                        649 CGTTACAACGAGATTATCAAGGCAGACTCCACCTTCATCA 688
73.MN159912.1_Botrytiscinerea                                             166 CGTTACCAAGAAATTATCAAGGAGACCTCCAACTTCATCA 205
74.MF034741.1_PeltasterfructicolaisolateSRB92                               0 ---------------------------------------- 0
75.LC440360.1_CercosporaasparagiCOasp2                                      0 ---------------------------------------- 0
76.AY944105.1_MagnaportheoryzaeisolateSAG00T3()                           255 ---------------------------------------- 255
77.JX266586.1_CochliobolusmiyabeanusvoucherMFLUCC10-0733                  162 CGTTACCAGGAGATCATCAAGGAGACCTCCAACTTCATCA 201
78.MN393253.1_CorynesporacassiicolaisolateQHD001(MN393253.1UNVERIFIED)     0 ---------------------------------------- 0
79.MF375218.1_AgroatheliarolfsiiisolateBJB24                              171 ----------------------------------AGTGGA 176
80.MN106270.1_AgroatheliarolfsiistrainJ-12                                178 ----------------------------------AGTGGA 183
81.OQ732628.1_AgroatheliarolfsiiisolateBTCBSr3                            156 ----------------------------------AGTGGA 161
82.KY196185.1_ColletotrichumtruncatumstrainPAK53                          468 ---------------------------------------- 468
83.GU935835.1_ColletotrichumcoccodesisolateC96002                         752 CGTTTCGAGGAGATCATCAAGGAGACCTCCAACTTCATCA 791
84.MK085963.1_AlternariatenuissimaisolateSCCZ06                             0 ---------------------------------------- 0
```

```
85.MT548042.1_AlternarialongipesstrainKY_2019_012                        0 ---------------------------------------- 0
86.MN356465.1CalonectriamontanaisolateHSP4                             489 ---------------------------------------- 489
87.OL694224.1_CalonectriacanadianastrainF099                           504 ---------------------------------------- 504
88.MK803351.1_NeoscytalidiumdimidiatumstrainKale4-C                      0 ---------------------------------------- 0
89.ON376993.1_CurvulariachiangmaiensisisolateND00J7                    164 CGTTACCAGGAAATCATCAAGGAGACCTCCAACTTCATCA 203
90.OQ383346.1_NeoscytalidiumdimidiatumisolateGKH-2                       0 ---------------------------------------- 0
91.MF662595.1_NeoscytalidiumnovaehollandiaeisolateNeNo1                  0 ---------------------------------------- 0
92.EF560588.1Melampsoralini                                            507 AGGTACGTGCAAAGATTCTC-GATCCCACTTCCTTCCATC 545
93.LC590862.1_NeoscytalidiumdimidiatumPSU-HP01TEF1                       0 ---------------------------------------- 0
94.KX278106.1_BotryosphaeriaqingyuanensisstrainCERC2947                  0 ---------------------------------------- 0
95.AJ578763.1_Blumeriagraminisf.sp.hordeicyp51                           0 ---------------------------------------- 0
96.MF490858.1_CurvulariadactylocteniicolastrainCPC28810                152 CGTTACCAGGAAATCATCAAGGAGACCTCCAACTTCATCA 191
97.KT287115.1_Bipolariscactivoraisolate3.8.6                           206 CGTTACCAGGAGATCATCAAGGAGACCTCCAACTTCATCA 245
98.MT560940.1_CurvulariacactivorastrainHLGH0118                        163 CGTTACCAGGAGATCATCAAGGAGACCTCCAACTTCATCA 202
99.OM714565.1_CurvulariaplantarumstrainM0134                           189 CGTTACCAGGAAATCATCAAGGAGACCTCCAACTTCATCA 228
100.MN159911.1_BotrytiscinereaSICAUCC19-0003                           166 CGTTACCAAGAAATTATCAAGGAGACCTCCAACTTCATCA 205
102.GU294713.1LasiodiplodiatheobromaestrainUCD2430TX                     0 ---------------------------------------- 0
103.KX868094.1_Mycosphaerellasp.isolateCRM20.1                         475 ---------------------------------------- 475
104.LC599478.1Pseudocercosporapini-densifloraeMUCC534                    0 ---------------------------------------- 0
105.N584698.1BipolarissetariaestrainKBS4-2                             217 CGTTACCAGGAGATCATCAAGGAGACCTCCAACTTCATCA 256

2.OM160859.1_F.buharicum                                               560 ---------------------------------------- 560
1.LC727524.1_F.buharicum_OKI-1_Okura                                   682 ---------------------------------------- 682
3.KX302919.1_F.sublunatum                                              521 ---------------------------------------- 521
4.LT996094.1_F.convolutans                                             516 ---------------------------------------- 516
5.OM160861.1_F.abutilonis                                              630 ---------------------------------------- 630
6.OM160874.1_F.guadeloupense                                           560 ---------------------------------------- 560
7.MH392475.1_F.graminearum                                             430 ---------------------------------------- 430
8.MH582420.1_F.solani                                                  540 ---------------------------------------- 540
9.MAFF244605_F.oxysporum                                               544 ---------------------------------------- 544
10.MAFF237278_F.contaminatum_Hylocereus                                711 ---------------------------------------- 711
11.MAFF237649_F.concentricum__Riceroot                                 678 ---------------------------------------- 678
12.MAFF237650_F.concentricum__Wheat                                    679 ---------------------------------------- 679
13.MAFF239869_F.mangiferae__Ryukyupine                                 657 ---------------------------------------- 657
14.MAFF240460_F.fujikuroi_Passionfruit                                 661 ---------------------------------------- 661
15.MAFF241317_F.graminearum_Wheat                                      666 ---------------------------------------- 666
16.MAFF242670_F.ipomoeae_Wheat                                         672 ---------------------------------------- 672
17.MAFF245129_F.concentricum_Fraxinus                                  659 ---------------------------------------- 659
18.MAFF245395_F.cugenangense_Rhubarb                                   671 ---------------------------------------- 671
19.MAFF246637_F.nirenbergiae_Strawberry                                671 ---------------------------------------- 671
20.MAFF246672_F.nirenbergiae_ChinesePeony                              671 ---------------------------------------- 671
21.MAFF246697_F.commune_Urallicoriceroot                               673 ---------------------------------------- 673
22.MAFF246729_F.falciforme_Angelica                                    691 ---------------------------------------- 691
23.MAFF247220_F.duplospermum__Euwallaceasp                             694 ---------------------------------------- 694
24.MAFF410760_F.odoratissimum_alpha                                    669 ---------------------------------------- 669
25.MAFF244605_FusariumoxysporumSchlechtendal_MAFF244605_Tomato         672 ---------------------------------------- 672
```

```
26.MAFF241326_F.asiaticum_Wheat                                         669 ---------------------------------------- 669
27.MAFF245014_F.asiaticum_Wildsoybean                                   666 ---------------------------------------- 666
28.MAFF150124_F.asiaticum__Wheat                                        666 ---------------------------------------- 666
29.OM135603.1F.algeriense                                               699 ---------------------------------------- 699
30.MAFF237465_F.penzigii_Aloe                                           704 ---------------------------------------- 704
31.MAFF103054_F.oxysporumSchlechtendalf.sp.cucumerinum_Cucumber         671 ---------------------------------------- 671
32.MAFF712246_F.oxysporumSchlechtendalf.sp.dianthi__Carnation           673 ---------------------------------------- 673
33.MAFF305558_F.oxysporumSchlechtendalf.sp.fragariae__Watermelon        671 ---------------------------------------- 671
34.MAFF744087_F.oxysporumSchlechtendalf.sp.lactucae__Lettuce            671 ---------------------------------------- 671
35.MAFF726924_F.oxysporumSchlechtendalf.sp.lagenariae_Whiteflowered gourd 671 ---------------------------------------- 671
36.MAFF744003_F.oxysporumSchlechtendalf.sp.lagenariae_Squash)           672 ---------------------------------------- 672
37.MAFF305122_F.oxysporumSchlechtendalf.sp.melonis__Melon               671 ---------------------------------------- 671
38.MAFF306714_F.oxysporumSchlechtendalf.sp.momordicae_Balsampear        671 ---------------------------------------- 671
39.MAFF238905_F.oxysporumSchlechtendalf.sp.radicis-lycopersici_Tomato   674 ---------------------------------------- 674
40.MAFF150004_F.oxysporumSchlechtendalf.sp.spinaciae_Spinach            671 ---------------------------------------- 671
41.MAFF247034_F.oxysporumSchlechtendal__Goldenchain                     671 ---------------------------------------- 671
42.MAFF245747_F.oxysporumSchlechtendalf.sp.callistephi__Chinaaster      671 ---------------------------------------- 671
43.MAFF305115_FoxysporumSchlechtendalf.sp.batatas__Sweatpotato          703 ---------------------------------------- 703
44.MAFF150126_F.asiaticum_Seed                                          666 ---------------------------------------- 666
45.MAFF246738_F.solani_Angelica                                         703 ---------------------------------------- 703
46.MAFF246664_F.cugenangense_Perilla                                    671 ---------------------------------------- 671
47.MH582420.1F.solanistrainMRC256                                       679 ---------------------------------------- 679
48.MAFF240361_F.babinda_Soil                                            693 ---------------------------------------- 693
49.MAFF242368_F.azukicola_Azukibean                                     708 ---------------------------------------- 708
50.MAFF241312_F.asiaticum_Soil,welshonionfield                          666 ---------------------------------------- 666
51.LT548416.1_F.culmorumpartialtef1a                                    646 ---------------------------------------- 646
52.MAFF150124_F.asiaticum__Wheat                                        666 ---------------------------------------- 666
53.MAFF238806_F.begoniae_Oncidiumsp                                     705 ---------------------------------------- 705
54.MW594399.1_FusariumincarnatumisolateUD01C                            689 ---------------------------------------- 689
55.OP414923.1Pucciniagraminisf.sp.triticiisolateSHZPgt19                529 CATCTAACTTATCTTCTGCCACCAGATACGAGGAAATCGT 568
56.MT027094.1_BipolarisoryzaestrainOrL-2                                193 AGAAGGTCGGATACAACCCCAAGCACGTTCCCTTCGTGCC 232
57.ON734360.1_AlternariaalternataisolateH126                             68 AGAAGGTCGGCTACAACCCCAAGCACGTTCCCTTCGTCCC 107
58.LC333578.1_StemphyliumlycopersiciSOasp2                                0 ---------------------------------------- 0
59.HQ718583.1_Colletotrichumgloeosporioidesisolateq-1                   567 ---------------------------------------- 567
60.JN241603.1_AtheliarolfsiiisolateSR1                                  467 GTGAGGACCCGTTTCAACGAAATCGTCAAGGAAACCTGCC 506
61.KJ866474.1_RhizoctoniasolanistrainMHL-1                              249 GCGAGGACC-GTTTCAACGAAATTATCAAGGAAACCT-CC 286
62.JQ672424.1AlternariatriticinaisolateEGS17-061                         445 AGAAGGTCGGCTACAACCCCAAGCACGTTCCCTTCGTCCC 484
63.LT707559.1_P.capsicipartialtef1agene                                 326 AGAAGGTGGGSTACAAGCCGGCTAAGATCCCGTTCGTGCC 365
64.MW090051.1_CurvularialunatastrainCls-3                               187 AGAAGGTCGGCTACAACCCCAAGCACGTTCCCTTCGTCCC 226
65.DQ400892.1_Aspergillusterreus                                        225 AGAAGGTCGGCTACAACCCCAAGGCCGTTCCCTTCGTCCC 264
66.DQ911416.1_Pythiumsp.quercumstrainPy292                              305 AGAAGGTGGGCTACAAGCCGGCGAAGATCCCGTTCGTGCC 344
67.EU797495.1_Phytophthorasp.oaksoilPoland                              305 AGAAGGTCGGCTACAAGCCCGCCAAGATCCCGTTCGTGCC 344
68.HM148321.1_Cladosporiumcucumerinum                                   385 ---------------------------------------- 385
69.AF398888.1_SclerotiniasclerotiorumisolateSS1                           0 ---------------------------------------- 0
70.AF398888.1_S.sclerotiorumisolateSS1                                    0 ---------------------------------------- 0
71.HPAB545908.1_Verticilliumnonalfalfaeisolate                          207 AGAAGGTCGGCTACAACCCCAAGACTGTCGCCTTCGTCCC 246
```

72.EF433315.1_CeratocystisfimbriatavoucherCMW15052	689	AGAAGGTCGGTTACAACCCCAAGACCGTTGCCTTTGTCCC	728
73.MN159912.1_Botrytiscinerea	206	AGAAGGTCGGATACAACCCAAAGACCGTTCCTTTCGTTCC	245
74.MF034741.1_PeltasterfructicolaisolateSRB92	0	--	0
75.LC440360.1_CercosporaasparagiCOasp2	0	--	0
76.AY944105.1_MagnaportheoryzaeisolateSAG00T3()	255	--	255
77.JX266586.1_CochliobolusmiyabeanusvoucherMFLUCC10-0733	202	AGAAGGTCGGATACAACCCCAAGCACGTTCCCTTCGTGCC	241
78.MN393253.1_CorynesporacassiicolaisolateQHD001(MN393253.1UNVERIFIED)	0	--	0
79.MF375218.1_AgroatheliarolfsiiisolateBJB24	177	GTGAGGACC-GTTTCAACGAAATCGTCAAGGAAACCT-CC	214
80.MN106270.1_AgroatheliarolfsiistrainJ-12	184	GTGAGGACC-GTTTCAACGAAATCGTCAAGGAAACCT-CC	221
81.OQ732628.1_AgroatheliarolfsiiisolateBTCBSr3	162	GTGAGGACC-GTTTCAACGAAATCGTCAAGGAAACCT-CC	199
82.KY196185.1_ColletotrichumtruncatumstrainPAK53	468	--	468
83.GU935835.1_ColletotrichumcoccodesisolateC96002	792	AGAAGGTCGGCTACAACCCCAAGACTGTTGCCTTCGTTCC	831
84.MK085963.1_AlternariatenuissimaisolateSCCZ06	0	--	0
85.MT548042.1_AlternarialongipesstrainKY_2019_012	0	--	0
86.MN356465.1CalonectriamontanaisolateHSP4	489	--	489
87.OL694224.1_CalonectriacanadianastrainF099	504	--	504
88.MK803351.1_NeoscytalidiumdimidiatumstrainKale4-C	0	--	0
89.ON376993.1_CurvulariachiangmaiensisisolateND00J7	204	AGAAGGTCGGCTACAACCCCAAGCACGTTCCCTTCGTCCC	243
90.OQ383346.1_NeoscytalidiumdimidiatumisolateGKH-2	0	--	0
91.MF662595.1_NeoscytalidiumnovaehollandiaeisolateNeNo1	0	--	0
92.EF560588.1Melampsoralini	546	CCGATTTCTTACCATATTGATGTATTTGTTCTTTTGTTTT	585
93.LC590862.1_NeoscytalidiumdimidiatumPSU-HP01TEF1	0	--	0
94.KX278106.1_BotryosphaeriaqingyuanensisstrainCERC2947	0	--	0
95.AJ578763.1_Blumeriagraminisf.sp.hordeicyp51	0	--	0
96.MF490858.1_CurvulariadactylocteniicolastrainCPC28810	192	AGAAGGTCGGCTACAACCCCAAGCACGTTCCCTTCGTCCC	231
97.KT287115.1_Bipolariscactivoraisolate3.8.6	246	AGAAGGTCGGCTACAACCCCAAGCACGTTCCCTTCGTCCC	285
98.MT560940.1_CurvulariacactivorastrainHLGH0118	203	AGAAGGTCGGCTACAACCCCAAGCACGTTCCCTTCGTCCC	242
99.OM714565.1_CurvulariaplantarumstrainM0134	229	AGAAGGTCGGCTACAACCCCAAGCACGTTCCCTTCGTCCC	268
100.MN159911.1_BotrytiscinereaSICAUCC19-0003	206	AGAAGGTCGGATACAACCCAAAGACCGTTCCTTTCGTTCC	245
102.GU294713.1LasiodiplodiatheobromaestrainUCD2430TX	0	--	0
103.KX868094.1_Mycosphaerellasp.isolateCRM20.1	475	--	475
104.LC599478.1Pseudocercosporapini-densifloraeMUCC534	0	--	0
105.N584698.1BipolarissetariaestrainKBS4-2	257	AGAAGGTCGGCTACAACCCCAAGCACGTTCCCTTCGTGCC	296
2.OM160859.1_F.buharicum	560	--	560
1.LC727524.1_F.buharicum_OKI-1_Okura	682	--	682
3.KX302919.1_F.sublunatum	521	--	521
4.LT996094.1_F.convolutans	516	--	516
5.OM160861.1_F.abutilonis	630	--	630
6.OM160874.1_F.guadeloupense	560	--	560
7.MH392475.1_F.graminearum	430	--	430
8.MH582420.1_F.solani	540	--	540
9.MAFF244605_F.oxysporum	544	--	544
10.MAFF237278_F.contaminatum_Hylocereus	711	--	711
11.MAFF237649_F.concentricum__Riceroot	678	--	678
12.MAFF237650_F.concentricum__Wheat	679	--	679

```
13.MAFF239869_F.mangiferae__Ryukyupine                                657 ---------------------------------------- 657
14.MAFF240460_F.fujikuroi_Passionfruit                                661 ---------------------------------------- 661
15.MAFF241317_F.graminearum_Wheat                                     666 ---------------------------------------- 666
16.MAFF242670_F.ipomoeae_Wheat                                        672 ---------------------------------------- 672
17.MAFF245129_F.concentricum_Fraxinus                                 659 ---------------------------------------- 659
18.MAFF245395_F.cugenangense_Rhubarb                                  671 ---------------------------------------- 671
19.MAFF246637_F.nirenbergiae_Strawberry                               671 ---------------------------------------- 671
20.MAFF246672_F.nirenbergiae_ChinesePeony                             671 ---------------------------------------- 671
21.MAFF246697_F.commune_Urallicoriceroot                              673 ---------------------------------------- 673
22.MAFF246729_F.falciforme_Angelica                                   691 ---------------------------------------- 691
23.MAFF247220_F.duplospermum__Euwallaceasp                            694 ---------------------------------------- 694
24.MAFF410760_F.odoratissimum_alpha                                   669 ---------------------------------------- 669
25.MAFF244605_FusariumoxysporumSchlechtendal_MAFF244605_Tomato        672 ---------------------------------------- 672
26.MAFF241326_F.asiaticum_Wheat                                       669 ---------------------------------------- 669
27.MAFF245014_F.asiaticum_Wildsoybean                                 666 ---------------------------------------- 666
28.MAFF150124_F.asiaticum__Wheat                                      666 ---------------------------------------- 666
29.OM135603.1F.algeriense                                             699 ---------------------------------------- 699
30.MAFF237465_F.penzigii_Aloe                                         704 ---------------------------------------- 704
31.MAFF103054_F.oxysporumSchlechtendalf.sp.cucumerinum_Cucumber       671 ---------------------------------------- 671
32.MAFF712246_F.oxysporumSchlechtendalf.sp.dianthi__Carnation         673 ---------------------------------------- 673
33.MAFF305558_F.oxysporumSchlechtendalf.sp.fragariae__Watermelon      671 ---------------------------------------- 671
34.MAFF744087_F.oxysporumSchlechtendalf.sp.lactucae__Lettuce          671 ---------------------------------------- 671
35.MAFF726924_F.oxysporumSchlechtendalf.sp.lagenariae_Whitefloweredgourd 671 ---------------------------------------- 671
36.MAFF744003_F.oxysporumSchlechtendalf.sp.lagenariae_Squash)         672 ---------------------------------------- 672
37.MAFF305122_F.oxysporumSchlechtendalf.sp.melonis__Melon             671 ---------------------------------------- 671
38.MAFF306714_F.oxysporumSchlechtendalf.sp.momordicae_Balsampear      671 ---------------------------------------- 671
39.MAFF238905_F.oxysporumSchlechtendalf.sp.radicis-lycopersici_Tomato 674 ---------------------------------------- 674
40.MAFF150004_F.oxysporumSchlechtendalf.sp.spinaciae_Spinach          671 ---------------------------------------- 671
41.MAFF247034_F.oxysporumSchlechtendal__Goldenchain                   671 ---------------------------------------- 671
42.MAFF245747_F.oxysporumSchlechtendalf.sp.callistephi__Chinaaster    671 ---------------------------------------- 671
43.MAFF305115_FoxysporumSchlechtendalf.sp.batatas__Sweatpotato        703 ---------------------------------------- 703
44.MAFF150126_F.asiaticum_Seed                                        666 ---------------------------------------- 666
45.MAFF246738_F.solani_Angelica                                       703 ---------------------------------------- 703
46.MAFF246664_F.cugenangense_Perilla                                  671 ---------------------------------------- 671
47.MH582420.1F.solanistrainMRC256                                     679 ---------------------------------------- 679
48.MAFF240361_F.babinda_Soil                                          693 ---------------------------------------- 693
49.MAFF242368_F.azukicola_Azukibean                                   708 ---------------------------------------- 708
50.MAFF241312_F.asiaticum_Soil,welshonionfield                        666 ---------------------------------------- 666
51.LT548416.1_F.culmorumpartialtef1a                                  646 ---------------------------------------- 646
52.MAFF150124_F.asiaticum__Wheat                                      666 ---------------------------------------- 666
53.MAFF238806_F.begoniae_Oncidiumsp                                   705 ---------------------------------------- 705
54.MW594399.1_FusariumincarnatumisolateUD01C                          689 ---------------------------------------- 689
55.OP414923.1Pucciniagraminisf.sp.triticiisolateSHZPgt19              569 CAAGGAAACCTCCAACTTCGTCAAGAAGGTCGGGTACAAC 608
56.MT027094.1_BipolarisoryzaestrainOrL-2                              233 CATCTCCGGTTTCAACGGTGACAACATGATTGAGGCCTCC 272
57.ON734360.1_AlternariaalternataisolateH126                          108 CATCTCCGGTTTCAACGGTGACAACATGATTGAGGCCTCA 147
58.LC333578.1_StemphyliumlycopersiciSOasp2                              0 ---------------------------------------- 0
```

```
59.HQ718583.1_Colletotrichumgloeosporioidesisolateq-1         567 ---------------------------------------- 567
60.JN241603.1_AtheliarolfsiiisolateSR1                        507 AACTTCATCAAGAAGGTCGGGTTACAACCCCAAGGCCGTC 546
61.KJ866474.1_RhizoctoniasolanistrainMHL-1                    287 AATTTTATCAAGAAGGTCGG-CTACAACCCCAAGACGGTC 325
62.JQ672424.1AlternariatriticinaisolateEGS17-061              485 CATCTCCGGCTTCAACGGTGACAACATGATCGAGGCTTCC 524
63.LT707559.1_P.capsicipartialtef1agene                       366 TATTTCCGGCTGGGAGGGAGACAACATGATCGACCGTTCC 405
64.MW090051.1_CurvularialunatastrainCls-3                     227 CATCTCCGGTTTCAACGGAGACAACATGATTGAGGCTTCC 266
65.DQ400892.1_Aspergillusterreus                              265 CATCTCCGGTTTCAACGGTGACAACATGCTTGAGCCTTCC 304
66.DQ911416.1_Pythiumsp.quercumstrainPy292                    345 TATCTCTGGCTGGGAGGGCGACAACATGATTGAGAAGTCG 384
67.EU797495.1_Phytophthorasp.oaksoilPoland                    345 CATCTCGGGCTGGGAGGGTGACAACATGATCGAACGTTCG 384
68.HM148321.1_Cladosporiumcucumerinum                         385 ---------------------------------------- 385
69.AF398888.1_SclerotiniasclerotiorumisolateSS1                 0 ---------------------------------------- 0
70.AF398888.1_S.sclerotiorumisolateSS1                          0 ---------------------------------------- 0
71.HPAB545908.1_Verticilliumnonalfalfaeisolate                247 CATCTCCGGCTTCAACGGCGACAACATGCTCCAGGCCTCC 286
72.EF433315.1_CeratocystisfimbriatavoucherCMW15052             729 CATCTCTGGGTTCACGGGC--------------------- 747
73.MN159912.1_Botrytiscinerea                                 246 TATCTCCGGTTTCAACGGTGATAACATGATCGACAACTCC 285
74.MF034741.1_PeltasterfructicolaisolateSRB92                   0 ---------------------------------------- 0
75.LC440360.1_CercosporaasparagiCOasp2                          0 ---------------------------------------- 0
76.AY944105.1_MagnaportheoryzaeisolateSAG00T3()               255 ---------------------------------------- 255
77.JX266586.1_CochliobolusmiyabeanusvoucherMFLUCC10-0733       242 CATCTCCGGTTTCAACGGTGACAACATGATTGAGGCCTCC 281
78.MN393253.1_CorynesporacassiicolaisolateQHD001(MN393253.1UNVERIFIED)   0 ---------------------------------------- 0
79.MF375218.1_AgroatheliarolfsiiisolateBJB24                  215 AACTTCATCAAGAAGGTCGG-TTACAACCCCAAGGCCGTC 253
80.MN106270.1_AgroatheliarolfsiistrainJ-12                    222 AACTTCATCAAGAAGGTCGG-TTACAACCCCAAGGCCGTC 260
81.OQ732628.1_AgroatheliarolfsiiisolateBTCBSr3                200 AACTTCATCAAGAAGGTCGG-TTACAACCC-AAGGCCGTC 237
82.KY196185.1_ColletotrichumtruncatumstrainPAK53               468 ---------------------------------------- 468
83.GU935835.1_ColletotrichumcoccodesisolateC96002             832 CATCTCCGGTTTCCACGGCGACAACATGCTTGCCCCCACC 871
84.MK085963.1_AlternariatenuissimaisolateSCCZ06                 0 ---------------------------------------- 0
85.MT548042.1_AlternarialongipesstrainKY_2019_012               0 ---------------------------------------- 0
86.MN356465.1CalonectriamontanaisolateHSP4                    489 ---------------------------------------- 489
87.OL694224.1_CalonectriacanadianastrainF099                  504 ---------------------------------------- 504
88.MK803351.1_NeoscytalidiumdimidiatumstrainKale4-C             0 ---------------------------------------- 0
89.ON376993.1_CurvulariachiangmaiensisisolateND00J7           244 CATCTCCGGTTTCAACGGAGACAACATGATTGAGGCTTCC 283
90.OQ383346.1_NeoscytalidiumdimidiatumisolateGKH-2              0 ---------------------------------------- 0
91.MF662595.1_NeoscytalidiumnovaehollandiaeisolateNeNo1         0 ---------------------------------------- 0
92.EF560588.1Melampsoralini                                   586 ACCCAGATACGAGGAAATCGTCAAGGAAACCTCCAACTTC 625
93.LC590862.1_NeoscytalidiumdimidiatumPSU-HP01TEF1              0 ---------------------------------------- 0
94.KX278106.1_BotryosphaeriaqingyuanensisstrainCERC2947         0 ---------------------------------------- 0
95.AJ578763.1_Blumeriagraminisf.sp.hordeicyp51                  0 ---------------------------------------- 0
96.MF490858.1_CurvulariadactylocteniicolastrainCPC28810       232 CATCTCCGGTTTCAACGGAGACAACATGATTGAGGCTTCC 271
97.KT287115.1_Bipolariscactivoraisolate3.8.6                  286 CATCTCTGGTTTCAACGGAGACAACATGATTGAGGCCTCC 325
98.MT560940.1_CurvulariacactivorastrainHLGH0118               243 CATCTCTGGTTTCAACGGAGACAACATGATTGAGGCCTCC 282
99.OM714565.1_CurvulariaplantarumstrainM0134                  269 CATCTCCGGTTTCAACGGAGACAACATGATTGAGGCCTCC 308
100.MN159911.1_BotrytiscinereaSICAUCC19-0003                  246 TATCTCCGGTTTCAACGGTGATAACATGATCGACAACTCC 285
102.GU294713.1LasiodiplodiatheobromaestrainUCD2430TX            0 ---------------------------------------- 0
103.KX868094.1_Mycosphaerellasp.isolateCRM20.1                475 ---------------------------------------- 475
104.LC599478.1Pseudocercosporapini-densifloraeMUCC534           0 ---------------------------------------- 0
105.N584698.1BipolarissetariaestrainKBS4-2                    297 CATCTCCGGTTTCAACGGTGACAACATGATTGAGGCCTCC 336
```

```
2.OM160859.1_F.buharicum                                            560 ---------------------------------------- 560
1.LC727524.1_F.buharicum_OKI-1_Okura                                682 ---------------------------------------- 682
3.KX302919.1_F.sublunatum                                           521 ---------------------------------------- 521
4.LT996094.1_F.convolutans                                          516 ---------------------------------------- 516
5.OM160861.1_F.abutilonis                                           630 ---------------------------------------- 630
6.OM160874.1_F.guadeloupense                                        560 ---------------------------------------- 560
7.MH392475.1_F.graminearum                                          430 ---------------------------------------- 430
8.MH582420.1_F.solani                                               540 ---------------------------------------- 540
9.MAFF244605_F.oxysporum                                            544 ---------------------------------------- 544
10.MAFF237278_F.contaminatum_Hylocereus                             711 ---------------------------------------- 711
11.MAFF237649_F.concentricum__Riceroot                              678 ---------------------------------------- 678
12.MAFF237650_F.concentricum__Wheat                                 679 ---------------------------------------- 679
13.MAFF239869_F.mangiferae__Ryukyupine                              657 ---------------------------------------- 657
14.MAFF240460_F.fujikuroi_Passionfruit                              661 ---------------------------------------- 661
15.MAFF241317_F.graminearum_Wheat                                   666 ---------------------------------------- 666
16.MAFF242670_F.ipomoeae_Wheat                                      672 ---------------------------------------- 672
17.MAFF245129_F.concentricum_Fraxinus                               659 ---------------------------------------- 659
18.MAFF245395_F.cugenangense_Rhubarb                                671 ---------------------------------------- 671
19.MAFF246637_F.nirenbergiae_Strawberry                             671 ---------------------------------------- 671
20.MAFF246672_F.nirenbergiae_ChinesePeony                           671 ---------------------------------------- 671
21.MAFF246697_F.commune_Urallicoriceroot                            673 ---------------------------------------- 673
22.MAFF246729_F.falciforme_Angelica                                 691 ---------------------------------------- 691
23.MAFF247220_F.duplospermum__Euwallaceasp                          694 ---------------------------------------- 694
24.MAFF410760_F.odoratissimum_alpha                                 669 ---------------------------------------- 669
25.MAFF244605_FusariumoxysporumSchlechtendal_MAFF244605_Tomato      672 ---------------------------------------- 672
26.MAFF241326_F.asiaticum_Wheat                                     669 ---------------------------------------- 669
27.MAFF245014_F.asiaticum_Wildsoybean                               666 ---------------------------------------- 666
28.MAFF150124_F.asiaticum__Wheat                                    666 ---------------------------------------- 666
29.OM135603.1F.algeriense                                           699 ---------------------------------------- 699
30.MAFF237465_F.penzigii_Aloe                                       704 ---------------------------------------- 704
31.MAFF103054_F.oxysporumSchlechtendalf.sp.cucumerinum_Cucumber     671 ---------------------------------------- 671
32.MAFF712246_F.oxysporumSchlechtendalf.sp.dianthi__Carnation       673 ---------------------------------------- 673
33.MAFF305558_F.oxysporumSchlechtendalf.sp.fragariae__Watermelon    671 ---------------------------------------- 671
34.MAFF744087_F.oxysporumSchlechtendalf.sp.lactucae__Lettuce        671 ---------------------------------------- 671
35.MAFF726924_F.oxysporumSchlechtendalf.sp.lagenariae_Whitefloweredgourd 671 ---------------------------------------- 671
36.MAFF744003_F.oxysporumSchlechtendalf.sp.lagenariae_Squash)       672 ---------------------------------------- 672
37.MAFF305122_F.oxysporumSchlechtendalf.sp.melonis__Melon           671 ---------------------------------------- 671
38.MAFF306714_F.oxysporumSchlechtendalf.sp.momordicae_Balsampear    671 ---------------------------------------- 671
39.MAFF238905_F.oxysporumSchlechtendalf.sp.radicis-lycopersici_Tomato 674 ---------------------------------------- 674
40.MAFF150004_F.oxysporumSchlechtendalf.sp.spinaciae_Spinach        671 ---------------------------------------- 671
41.MAFF247034_F.oxysporumSchlechtendal__Goldenchain                 671 ---------------------------------------- 671
42.MAFF245747_F.oxysporumSchlechtendalf.sp.callistephi__Chinaaster  671 ---------------------------------------- 671
43.MAFF305115_FoxysporumSchlechtendalf.sp.batatas__Sweatpotato      703 ---------------------------------------- 703
44.MAFF150126_F.asiaticum_Seed                                      666 ---------------------------------------- 666
45.MAFF246738_F.solani_Angelica                                     703 ---------------------------------------- 703
46.MAFF246664_F.cugenangense_Perilla                                671 ---------------------------------------- 671
```

47.MH582420.1F.solanistrainMRC256	679	--	679
48.MAFF240361_F.babinda_Soil	693	--	693
49.MAFF242368_F.azukicola_Azukibean	708	--	708
50.MAFF241312_F.asiaticum_Soil,welshonionfield	666	--	666
51.LT548416.1_F.culmorumpartialtef1a	646	--	646
52.MAFF150124_F.asiaticum__Wheat	666	--	666
53.MAFF238806_F.begoniae_Oncidiumsp	705	--	705
54.MW594399.1_FusariumincarnatumisolateUD01C	689	--	689
55.OP414923.1Pucciniagraminisf.sp.triticiisolateSHZPgt19	609	CCCAAATCTATCGCC-------------------------	623
56.MT027094.1_BipolarisoryzaestrainOrL-2	273	ACCAACTGCCCCTGGTACAAGGGTTGGGAGAAGGAGACCA	312
57.ON734360.1_AlternariaalternataisolateH126	148	TCCAACTGCCCCTGGTACAAGGGTTGGGAGAAGGAGACCA	187
58.LC333578.1_StemphyliumlycopersiciSOasp2	0	--	0
59.HQ718583.1_Colletotrichumgloeosporioidesisolateq-1	567	--	567
60.JN241603.1_AtheliarolfsiiisolateSR1	547	GCCTTCCGTCCCCCATCTCCGGATGGCACGGTGACAACAT	586
61.KJ866474.1_RhizoctoniasolanistrainMHL-1	326	GCCTTT-GTCCCT-ATCTCTGGATGGCACGGTGACAACAT	363
62.JQ672424.1AlternariatriticinaisolateEGS17-061	525	TCCAACTGCCCCTGGTACAAGGGTTGGGAGAAGGAGACCA	564
63.LT707559.1_P.capsicipartialtef1agene	406	ACCAACATGCCGTGGTACAAGGGACCTTAC----------	435
64.MW090051.1_CurvularialunatastrainCls-3	267	ACCAACTGCCCCTGGTACAAGGGTTGGGAGAAGGAGACCA	306
65.DQ400892.1_Aspergillusterreus	305	CCCAACTGC-------------------------------	313
66.DQ911416.1_Pythiumsp.quercumstrainPy292	385	TCGAACATGCCGTGGTACAAGGGCCCGTAC----------	414
67.EU797495.1_Phytophthorasp.oaksoilPoland	385	TCGAACATGCCGTGGTACAAGGGACCCTTC----------	414
68.HM148321.1_Cladosporiumcucumerinum	385	--	385
69.AF398888.1_SclerotiniasclerotiorumisolateSS1	0	--	0
70.AF398888.1_S.sclerotiorumisolateSS1	0	--	0
71.HPAB545908.1_Verticilliumnonalfalfaeisolate	287	ACCAACTGC-------------------------------	295
72.EF433315.1_CeratocystisfimbriatavoucherCMW15052	747	--	747
73.MN159912.1_Botrytiscinerea	286	ACCAACTGCCCATGGTACAAGGGTTGGGAGAAGGAAGCCA	325
74.MF034741.1_PeltasterfructicolaisolateSRB92	0	--	0
75.LC440360.1_CercosporaasparagiCOasp2	0	--	0
76.AY944105.1_MagnaportheoryzaeisolateSAG00T3()	255	--	255
77.JX266586.1_CochliobolusmiyabeanusvoucherMFLUCC10-0733	282	ACCAACTGCCCCTGGTACAAGGGTTGGGAGAAGGAGACCA	321
78.MN393253.1_CorynesporacassiicolaisolateQHD001(MN393253.1UNVERIFIED)	0	--	0
79.MF375218.1_AgroatheliarolfsiiisolateBJB24	254	GCCTTC-GTCCCC-ATCTCCGGATGGCACGGTGACAACAT	291
80.MN106270.1_AgroatheliarolfsiistrainJ-12	261	GCCTTC-GTCCCC-ATCTCCGGATGGCACGGTGACAACAT	298
81.OQ732628.1_AgroatheliarolfsiiisolateBTCBSr3	238	GCCTTC-GTCCCC-ATCTCCGGATGGCACGGTGACAACAT	275
82.KY196185.1_ColletotrichumtruncatumstrainPAK53	468	--	468
83.GU935835.1_ColletotrichumcoccodesisolateC96002	872	ACCAACGCCCCTTGGTACAAGGGTTGGGAGAAGGAGACCA	911
84.MK085963.1_AlternariatenuissimaisolateSCCZ06	0	--	0
85.MT548042.1_AlternarialongipesstrainKY_2019_012	0	--	0
86.MN356465.1CalonectriamontanaisolateHSP4	489	--	489
87.OL694224.1_CalonectriacanadianastrainF099	504	--	504
88.MK803351.1_NeoscytalidiumdimidiatumstrainKale4-C	0	--	0
89.ON376993.1_CurvulariachiangmaiensisisolateND00J7	284	ACCAACTGCCCCTGGTACAAGGGTTGGGAGAAGGAGACCA	323
90.OQ383346.1_NeoscytalidiumdimidiatumisolateGKH-2	0	--	0
91.MF662595.1_NeoscytalidiumnovaehollandiaeisolateNeNo1	0	--	0
92.EF560588.1Melampsoralini	626	GTCAAGAAGGTTGGATTCAACCCCAAGACAA---------	656

93.LC590862.1_NeoscytalidiumdimidiatumPSU-HP01TEF1	0	--	0
94.KX278106.1_BotryosphaeriaqingyuanensisstrainCERC2947	0	--	0
95.AJ578763.1_Blumeriagraminisf.sp.hordeicyp51	0	--	0
96.MF490858.1_CurvulariadactylocteniicolastrainCPC28810	272	ACCAACTGCCCCTGGTACAAGGGTTGGGAGAAGGAGACCA	311
97.KT287115.1_Bipolariscactivoraisolate3.8.6	326	ACCAACTGCCCCTGGTACAAGGGTTGGGAGAAGGAGACCA	365
98.MT560940.1_CurvulariacactivorastrainHLGH0118	283	ACCAACTGCCCCTGGTACAAGGGTTGGGAGAAGGAGACCA	322
99.OM714565.1_CurvulariaplantarumstrainM0134	309	ACCAACTGCCCCTGGTACAAGGGTTGGGAGAAGGAGACCA	348
100.MN159911.1_BotrytiscinereaSICAUCC19-0003	286	ACCAACTGCCCATGGTACAAGGGTTGGGAGAAGGAAGCCA	325
102.GU294713.1LasiodiplodiatheobromaestrainUCD2430TX	0	--	0
103.KX868094.1_Mycosphaerellasp.isolateCRM20.1	475	--	475
104.LC599478.1Pseudocercosporapini-densifloraeMUCC534	0	--	0
105.N584698.1BipolarissetariaestrainKBS4-2	337	ACCAACTGCCCCTGGTACAAGGGTTGGGAGAAGGAGACCA	376
2.OM160859.1_F.buharicum	560	--	560
1.LC727524.1_F.buharicum_OKI-1_Okura	682	--	682
3.KX302919.1_F.sublunatum	521	--	521
4.LT996094.1_F.convolutans	516	--	516
5.OM160861.1_F.abutilonis	630	--	630
6.OM160874.1_F.guadeloupense	560	--	560
7.MH392475.1_F.graminearum	430	--	430
8.MH582420.1_F.solani	540	--	540
9.MAFF244605_F.oxysporum	544	--	544
10.MAFF237278_F.contaminatum_Hylocereus	711	--	711
11.MAFF237649_F.concentricum__Riceroot	678	--	678
12.MAFF237650_F.concentricum__Wheat	679	--	679
13.MAFF239869_F.mangiferae__Ryukyupine	657	--	657
14.MAFF240460_F.fujikuroi_Passionfruit	661	--	661
15.MAFF241317_F.graminearum_Wheat	666	--	666
16.MAFF242670_F.ipomoeae_Wheat	672	--	672
17.MAFF245129_F.concentricum_Fraxinus	659	--	659
18.MAFF245395_F.cugenangense_Rhubarb	671	--	671
19.MAFF246637_F.nirenbergiae_Strawberry	671	--	671
20.MAFF246672_F.nirenbergiae_ChinesePeony	671	--	671
21.MAFF246697_F.commune_Urallicoriceroot	673	--	673
22.MAFF246729_F.falciforme_Angelica	691	--	691
23.MAFF247220_F.duplospermum__Euwallaceasp	694	--	694
24.MAFF410760_F.odoratissimum_alpha	669	--	669
25.MAFF244605_FusariumoxysporumSchlechtendal_MAFF244605_Tomato	672	--	672
26.MAFF241326_F.asiaticum_Wheat	669	--	669
27.MAFF245014_F.asiaticum_Wildsoybean	666	--	666
28.MAFF150124_F.asiaticum__Wheat	666	--	666
29.OM135603.1F.algeriense	699	--	699
30.MAFF237465_F.penzigii_Aloe	704	--	704
31.MAFF103054_F.oxysporumSchlechtendalf.sp.cucumerinum_Cucumber	671	--	671
32.MAFF712246_F.oxysporumSchlechtendalf.sp.dianthi__Carnation	673	--	673
33.MAFF305558_F.oxysporumSchlechtendalf.sp.fragariae__Watermelon	671	--	671

```
34.MAFF744087_F.oxysporumSchlechtendalf.sp.lactucae__Lettuce              671 ---------------------------------------- 671
35.MAFF726924_F.oxysporumSchlechtendalf.sp.lagenariae_Whitefloweredgourd  671 ---------------------------------------- 671
36.MAFF744003_F.oxysporumSchlechtendalf.sp.lagenariae_Squash)             672 ---------------------------------------- 672
37.MAFF305122_F.oxysporumSchlechtendalf.sp.melonis__Melon                 671 ---------------------------------------- 671
38.MAFF306714_F.oxysporumSchlechtendalf.sp.momordicae_Balsampear          671 ---------------------------------------- 671
39.MAFF238905_F.oxysporumSchlechtendalf.sp.radicis-lycopersici_Tomato     674 ---------------------------------------- 674
40.MAFF150004_F.oxysporumSchlechtendalf.sp.spinaciae_Spinach              671 ---------------------------------------- 671
41.MAFF247034_F.oxysporumSchlechtendal__Goldenchain                       671 ---------------------------------------- 671
42.MAFF245747_F.oxysporumSchlechtendalf.sp.callistephi__Chinaaster        671 ---------------------------------------- 671
43.MAFF305115_FoxysporumSchlechtendalf.sp.batatas__Sweatpotato            703 ---------------------------------------- 703
44.MAFF150126_F.asiaticum_Seed                                            666 ---------------------------------------- 666
45.MAFF246738_F.solani_Angelica                                           703 ---------------------------------------- 703
46.MAFF246664_F.cugenangense_Perilla                                      671 ---------------------------------------- 671
47.MH582420.1F.solanistrainMRC256                                         679 ---------------------------------------- 679
48.MAFF240361_F.babinda_Soil                                              693 ---------------------------------------- 693
49.MAFF242368_F.azukicola_Azukibean                                       708 ---------------------------------------- 708
50.MAFF241312_F.asiaticum_Soil,welshonionfield                            666 ---------------------------------------- 666
51.LT548416.1_F.culmorumpartialtef1a                                      646 ---------------------------------------- 646
52.MAFF150124_F.asiaticum__Wheat                                          666 ---------------------------------------- 666
53.MAFF238806_F.begoniae_Oncidiumsp                                       705 ---------------------------------------- 705
54.MW594399.1_FusariumincarnatumisolateUD01C                              689 ---------------------------------------- 689
55.OP414923.1Pucciniagraminisf.sp.triticiisolateSHZPgt19                  623 ---------------------------------------- 623
56.MT027094.1_BipolarisoryzaestrainOrL-2                                  313 AGT------CCAAGTCCACCGGTAAGACCCTCCTCGAGGC 346
57.ON734360.1_AlternariaalternataisolateH126                              188 AGG------CCAAGGCCACTGGTAAGACCCTCCTCGAGGC 221
58.LC333578.1_StemphyliumlycopersiciSOasp2                                  0 ---------------------------------------- 0
59.HQ718583.1_Colletotrichumgloeosporioidesisolateq-1                     567 ---------------------------------------- 567
60.JN241603.1_AtheliarolfsiiisolateSR1                                    587 GTTGGAGGAGTCCACCAAGTAAGCCGTCGATTGTCCGTGC 626
61.KJ866474.1_RhizoctoniasolanistrainMHL-1                                364 GTTGGAGGAGTCCCCCAAGTA---CGTATACTGTC-GCAC 399
62.JQ672424.1AlternariatriticinaisolateEGS17-061                          565 AGG------CCAAGGCCACTGGTAAGACCCTCCTCGAGGC 598
63.LT707559.1_P.capsicipartialtef1agene                                   436 -----------------------------CTCCTTGAGGC 446
64.MW090051.1_CurvularialunatastrainCls-3                                 307 AGG------CCAAGGCCACTGGTAAGACCCTCCTCGAGGC 340
65.DQ400892.1_Aspergillusterreus                                          313 ---------------------------------------- 313
66.DQ911416.1_Pythiumsp.quercumstrainPy292                                415 -----------------------------CTCCTCGAGGC 425
67.EU797495.1_Phytophthorasp.oaksoilPoland                                415 -----------------------------CTCCTTGAGGC 425
68.HM148321.1_Cladosporiumcucumerinum                                     385 ---------------------------------------- 385
69.AF398888.1_SclerotiniasclerotiorumisolateSS1                             0 ---------------------------------------- 0
70.AF398888.1_S.sclerotiorumisolateSS1                                      0 ---------------------------------------- 0
71.HPAB545908.1_Verticilliumnonalfalfaeisolate                            295 ---------------------------------------- 295
72.EF433315.1_CeratocystisfimbriatavoucherCMW15052                        747 ---------------------------------------- 747
73.MN159912.1_Botrytiscinerea                                             326 AGGGAGGAGCCAAGTCCACCGGAAAGACTCTCCTCGAGGC 365
74.MF034741.1_PeltasterfructicolaisolateSRB92                               0 ---------------------------------------- 0
75.LC440360.1_CercosporaasparagiCOasp2                                      0 ---------------------------------------- 0
76.AY944105.1_MagnaportheoryzaeisolateSAG00T3()                           255 ---------------------------------------- 255
77.JX266586.1_CochliobolusmiyabeanusvoucherMFLUCC10-0733                  322 AGT------CCAAGTCCACCGGTAAGACCCTCCTCGAGGC 355
78.MN393253.1_CorynesporacassiicolaisolateQHD001(MN393253.1UNVERIFIED)      0 ---------------------------------------- 0
79.MF375218.1_AgroatheliarolfsiiisolateBJB24                              292 GTTGGAGGAGTCCACCAAGTAAGCCGTCGATTGTCCGTGC 331
```

```
80.MN106270.1_AgroatheliarolfsiistrainJ-12                 299 GTTGGAGGAGTCCACCAAGTAAGCCGTCGATTGTCCGTGC 338
81.OQ732628.1_AgroatheliarolfsiiisolateBTCBSr3             276 GTTGGAGGAGTCCACCAAGTAAGCCGTCGATTGTCCGTGC 315
82.KY196185.1_ColletotrichumtruncatumstrainPAK53           468 ---------------------------------------- 468
83.GU935835.1_ColletotrichumcoccodesisolateC96002          912 AGG---CCGGCAAGTCCACCGGTAAGACTCTCCTTGAGGC 948
84.MK085963.1_AlternariatenuissimaisolateSCCZ06              0 ---------------------------------------- 0
85.MT548042.1_AlternarialongipesstrainKY_2019_012            0 ---------------------------------------- 0
86.MN356465.1CalonectriamontanaisolateHSP4                 489 ---------------------------------------- 489
87.OL694224.1_CalonectriacanadianastrainF099               504 ---------------------------------------- 504
88.MK803351.1_NeoscytalidiumdimidiatumstrainKale4-C          0 ---------------------------------------- 0
89.ON376993.1_CurvulariachiangmaiensisisolateND00J7        324 AGG------CCAAGGCCACTGGTAAGACCCTCCTCGAGGC 357
90.OQ383346.1_NeoscytalidiumdimidiatumisolateGKH-2           0 ---------------------------------------- 0
91.MF662595.1_NeoscytalidiumnovaehollandiaeisolateNeNo1      0 ---------------------------------------- 0
92.EF560588.1Melampsoralini                                656 ---------------------------------------- 656
93.LC590862.1_NeoscytalidiumdimidiatumPSU-HP01TEF1           0 ---------------------------------------- 0
94.KX278106.1_BotryosphaeriaqingyuanensisstrainCERC2947      0 ---------------------------------------- 0
95.AJ578763.1_Blumeriagraminisf.sp.hordeicyp51               0 ---------------------------------------- 0
96.MF490858.1_CurvulariadactylocteniicolastrainCPC28810    312 AGG------CCAAGGCCACTGGTAAGACCCTCCTCGAGGC 345
97.KT287115.1_Bipolariscactivoraisolate3.8.6               366 AGG------CCAAGGCCACTGGTAAGACCCTCCTTGAGGC 399
98.MT560940.1_CurvulariacactivorastrainHLGH0118            323 AGG------CCAAGGCCACTGGTAAGACCCTCCTTGAGGC 356
99.OM714565.1_CurvulariaplantarumstrainM0134               349 AGG------CCAAGGCCACTGGTAAGACCCTCCTTGAGGC 382
100.MN159911.1_BotrytiscinereaSICAUCC19-0003               326 AGGGAGGAGCCAAGTCCACCGGAAAGACTCTCCTCGAGGC 365
102.GU294713.1LasiodiplodiatheobromaestrainUCD2430TX         0 ---------------------------------------- 0
103.KX868094.1_Mycosphaerellasp.isolateCRM20.1             475 ---------------------------------------- 475
104.LC599478.1Pseudocercosporapini-densifloraeMUCC534        0 ---------------------------------------- 0
105.N584698.1BipolarissetariaestrainKBS4-2                 377 AGT------CCAAGGCCACCGGTAAGACCCTCCTCGAGGC 410

2.OM160859.1_F.buharicum                                   560 ---------------------------------------- 560
1.LC727524.1_F.buharicum_OKI-1_Okura                       682 ---------------------------------------- 682
3.KX302919.1_F.sublunatum                                  521 ---------------------------------------- 521
4.LT996094.1_F.convolutans                                 516 ---------------------------------------- 516
5.OM160861.1_F.abutilonis                                  630 ---------------------------------------- 630
6.OM160874.1_F.guadeloupense                               560 ---------------------------------------- 560
7.MH392475.1_F.graminearum                                 430 ---------------------------------------- 430
8.MH582420.1_F.solani                                      540 ---------------------------------------- 540
9.MAFF244605_F.oxysporum                                   544 ---------------------------------------- 544
10.MAFF237278_F.contaminatum_Hylocereus                    711 ---------------------------------------- 711
11.MAFF237649_F.concentricum__Riceroot                     678 ---------------------------------------- 678
12.MAFF237650_F.concentricum__Wheat                        679 ---------------------------------------- 679
13.MAFF239869_F.mangiferae__Ryukyupine                     657 ---------------------------------------- 657
14.MAFF240460_F.fujikuroi_Passionfruit                     661 ---------------------------------------- 661
15.MAFF241317_F.graminearum_Wheat                          666 ---------------------------------------- 666
16.MAFF242670_F.ipomoeae_Wheat                             672 ---------------------------------------- 672
17.MAFF245129_F.concentricum_Fraxinus                      659 ---------------------------------------- 659
18.MAFF245395_F.cugenangense_Rhubarb                       671 ---------------------------------------- 671
19.MAFF246637_F.nirenbergiae_Strawberry                    671 ---------------------------------------- 671
20.MAFF246672_F.nirenbergiae_ChinesePeony                  671 ---------------------------------------- 671
```

```
21.MAFF246697_F.commune_Urallicoriceroot                          673 ---------------------------------------- 673
22.MAFF246729_F.falciforme_Angelica                               691 ---------------------------------------- 691
23.MAFF247220_F.duplospermum__Euwallaceasp                        694 ---------------------------------------- 694
24.MAFF410760_F.odoratissimum_alpha                               669 ---------------------------------------- 669
25.MAFF244605_FusariumoxysporumSchlechtendal_MAFF244605_Tomato    672 ---------------------------------------- 672
26.MAFF241326_F.asiaticum_Wheat                                   669 ---------------------------------------- 669
27.MAFF245014_F.asiaticum_Wildsoybean                             666 ---------------------------------------- 666
28.MAFF150124_F.asiaticum__Wheat                                  666 ---------------------------------------- 666
29.OM135603.1F.algeriense                                         699 ---------------------------------------- 699
30.MAFF237465_F.penzigii_Aloe                                     704 ---------------------------------------- 704
31.MAFF103054_F.oxysporumSchlechtendalf.sp.cucumerinum_Cucumber   671 ---------------------------------------- 671
32.MAFF712246_F.oxysporumSchlechtendalf.sp.dianthi__Carnation     673 ---------------------------------------- 673
33.MAFF305558_F.oxysporumSchlechtendalf.sp.fragariae__Watermelon  671 ---------------------------------------- 671
34.MAFF744087_F.oxysporumSchlechtendalf.sp.lactucae__Lettuce      671 ---------------------------------------- 671
35.MAFF726924_F.oxysporumSchlechtendalf.sp.lagenariae_Whiteflоweredgourd 671 ---------------------------------------- 671
36.MAFF744003_F.oxysporumSchlechtendalf.sp.lagenariae_Squash)     672 ---------------------------------------- 672
37.MAFF305122_F.oxysporumSchlechtendalf.sp.melonis__Melon         671 ---------------------------------------- 671
38.MAFF306714_F.oxysporumSchlechtendalf.sp.momordicae_Balsampear  671 ---------------------------------------- 671
39.MAFF238905_F.oxysporumSchlechtendalf.sp.radicis-lycopersici_Tomato 674 ---------------------------------------- 674
40.MAFF150004_F.oxysporumSchlechtendalf.sp.spinaciae_Spinach      671 ---------------------------------------- 671
41.MAFF247034_F.oxysporumSchlechtendal__Goldenchain               671 ---------------------------------------- 671
42.MAFF245747_F.oxysporumSchlechtendalf.sp.callistephi__Chinaaster 671 ---------------------------------------- 671
43.MAFF305115_FoxysporumSchlechtendalf.sp.batatas__Sweatpotato    703 ---------------------------------------- 703
44.MAFF150126_F.asiaticum_Seed                                    666 ---------------------------------------- 666
45.MAFF246738_F.solani_Angelica                                   703 ---------------------------------------- 703
46.MAFF246664_F.cugenangense_Perilla                              671 ---------------------------------------- 671
47.MH582420.1F.solanistrainMRC256                                 679 ---------------------------------------- 679
48.MAFF240361_F.babinda_Soil                                      693 ---------------------------------------- 693
49.MAFF242368_F.azukicola_Azukibean                               708 ---------------------------------------- 708
50.MAFF241312_F.asiaticum_Soil,welshonionfield                    666 ---------------------------------------- 666
51.LT548416.1_F.culmorumpartialtef1a                              646 ---------------------------------------- 646
52.MAFF150124_F.asiaticum__Wheat                                  666 ---------------------------------------- 666
53.MAFF238806_F.begoniae_Oncidiumsp                               705 ---------------------------------------- 705
54.MW594399.1_FusariumincarnatumisolateUD01C                      689 ---------------------------------------- 689
55.OP414923.1Pucciniagraminisf.sp.triticiisolateSHZPgt19          623 ---------------------------------------- 623
56.MT027094.1_BipolarisoryzaestrainOrL-2                          347 CATCGATGCCATCGACCCTCCTAGC--------------- 371
57.ON734360.1_AlternariaalternataisolateH126                      222 CATCGACGCCATCGACCCTCCCAGC--------------- 246
58.LC333578.1_StemphyliumlycopersiciSOasp2                          0 ---------------------------------------- 0
59.HQ718583.1_Colletotrichumgloeosporioidesisolateq-1             567 ---------------------------------------- 567
60.JN241603.1_AtheliarolfsiiisolateSR1                            627 CGATTTGATATGGCGTCTCATCATCTTTTGCAGCATGCCA 666
61.KJ866474.1_RhizoctoniasolanistrainMHL-1                        400 CTAGGTT-TACGCTTTGACTGACTTGACCCCAGCATGCCA 438
62.JQ672424.1AlternariatriticinaisolateEGS17-061                  599 CATTGACGCTATCGACCCTCCCAGC--------------- 623
63.LT707559.1_P.capsicipartialtef1agene                           447 TCTTGACAACCTGAACGCCCCCAAG--------------- 471
64.MW090051.1_CurvularialunatastrainCls-3                         341 CATCGACGCCATCGACCCCCCTGTC--------------- 365
65.DQ400892.1_Aspergillusterreus                                  314 -------------------------------------CCC 316
66.DQ911416.1_Pythiumsp.quercumstrainPy292                        426 GCTTGACCAGCTGAACGCCCCCAAG--------------- 450
```

```
67.EU797495.1_Phytophthorasp.oaksoilPoland                                426 TCTTGACCTGCTGAACGCCCCCAAG--------------- 450
68.HM148321.1_Cladosporiumcucumerinum                                     385 ---------------------------------------- 385
69.AF398888.1_SclerotiniasclerotiorumisolateSS1                             0 ---------------------------------------- 0
70.AF398888.1_S.sclerotiorumisolateSS1                                      0 ---------------------------------------- 0
71.HPAB545908.1_Verticilliumnonalfalfaeisolate                            296 -------------------------------------CCC 298
72.EF433315.1_CeratocystisfimbriatavoucherCMW15052                        747 ---------------------------------------- 747
73.MN159912.1_Botrytiscinerea                                             366 CATCGATGCTATCGACCCTCCTTCC--------------- 390
74.MF034741.1_PeltasterfructicolaisolateSRB92                               0 ---------------------------------------- 0
75.LC440360.1_CercosporaasparagiCOasp2                                      0 ---------------------------------------- 0
76.AY944105.1_MagnaportheoryzaeisolateSAG00T3()                           255 ---------------------------------------- 255
77.JX266586.1_CochliobolusmiyabeanusvoucherMFLUCC10-0733                  356 CATCGATGCCATCGACCCTCCTAGC--------------- 380
78.MN393253.1_CorynesporacassiicolaisolateQHD001(MN393253.1UNVERIFIED)      0 ---------------------------------------- 0
79.MF375218.1_AgroatheliarolfsiiisolateBJB24                              332 CGATTTGATATGGCGTCTCATCATCTTTTGCAGCATGCCA 371
80.MN106270.1_AgroatheliarolfsiistrainJ-12                                339 CGATTTAATATGACGTCTCATCATCTTTTCCAGCATGCCA 378
81.OQ732628.1_AgroatheliarolfsiiisolateBTCBSr3                            316 CGATTTGATATGGCGTCTCATCATCTTTTGCAGCATGCCA 355
82.KY196185.1_ColletotrichumtruncatumstrainPAK53                          468 ---------------------------------------- 468
83.GU935835.1_ColletotrichumcoccodesisolateC96002                         949 CATCGACTCCATCGAGCAGCCCAAG--------------- 973
84.MK085963.1_AlternariatenuissimaisolateSCCZ06                             0 ---------------------------------------- 0
85.MT548042.1_AlternarialongipesstrainKY_2019_012                           0 ---------------------------------------- 0
86.MN356465.1CalonectriamontanaisolateHSP4                                489 ---------------------------------------- 489
87.OL694224.1_CalonectriacanadianastrainF099                              504 ---------------------------------------- 504
88.MK803351.1_NeoscytalidiumdimidiatumstrainKale4-C                         0 ---------------------------------------- 0
89.ON376993.1_CurvulariachiangmaiensisisolateND00J7                       358 CATCGACGCCATCGACCCCCCTGTC--------------- 382
90.OQ383346.1_NeoscytalidiumdimidiatumisolateGKH-2                          0 ---------------------------------------- 0
91.MF662595.1_NeoscytalidiumnovaehollandiaeisolateNeNo1                     0 ---------------------------------------- 0
92.EF560588.1Melampsoralini                                               656 ---------------------------------------- 656
93.LC590862.1_NeoscytalidiumdimidiatumPSU-HP01TEF1                          0 ---------------------------------------- 0
94.KX278106.1_BotryosphaeriaqingyuanensisstrainCERC2947                     0 ---------------------------------------- 0
95.AJ578763.1_Blumeriagraminisf.sp.hordeicyp51                              0 ---------------------------------------- 0
96.MF490858.1_CurvulariadactylocteniicolastrainCPC28810                   346 CATCGACGCCATCGACCCCCCTGTC--------------- 370
97.KT287115.1_Bipolariscactivoraisolate3.8.6                              400 CATCGACGCCATCGACCCTCCTGCC--------------- 424
98.MT560940.1_CurvulariacactivorastrainHLGH0118                           357 CATCGACGCCATCGACCCTCCTGCC--------------- 381
99.OM714565.1_CurvulariaplantarumstrainM0134                              383 CATCGACGCCATCGACCCTCCTGTC--------------- 407
100.MN159911.1_BotrytiscinereaSICAUCC19-0003                              366 CATCGATGCTATCGACCCTCCTTCC--------------- 390
102.GU294713.1LasiodiplodiatheobromaestrainUCD2430TX                         0 ---------------------------------------- 0
103.KX868094.1_Mycosphaerellasp.isolateCRM20.1                            475 ---------------------------------------- 475
104.LC599478.1Pseudocercosporapini-densifloraeMUCC534                       0 ---------------------------------------- 0
105.N584698.1BipolarissetariaestrainKBS4-2                                411 CATCGATGCCATCGACCCTCCCAGC--------------- 435

2.OM160859.1_F.buharicum                                                  560 ---------------------------------------- 560
1.LC727524.1_F.buharicum_OKI-1_Okura                                      682 ---------------------------------------- 682
3.KX302919.1_F.sublunatum                                                 521 ---------------------------------------- 521
4.LT996094.1_F.convolutans                                                516 ---------------------------------------- 516
5.OM160861.1_F.abutilonis                                                 630 ---------------------------------------- 630
6.OM160874.1_F.guadeloupense                                              560 ---------------------------------------- 560
7.MH392475.1_F.graminearum                                                430 ---------------------------------------- 430
```

8.MH582420.1_F.solani	540	--	540
9.MAFF244605_F.oxysporum	544	--	544
10.MAFF237278_F.contaminatum_Hylocereus	711	--	711
11.MAFF237649_F.concentricum__Riceroot	678	--	678
12.MAFF237650_F.concentricum__Wheat	679	--	679
13.MAFF239869_F.mangiferae__Ryukyupine	657	--	657
14.MAFF240460_F.fujikuroi_Passionfruit	661	--	661
15.MAFF241317_F.graminearum_Wheat	666	--	666
16.MAFF242670_F.ipomoeae_Wheat	672	--	672
17.MAFF245129_F.concentricum_Fraxinus	659	--	659
18.MAFF245395_F.cugenangense_Rhubarb	671	--	671
19.MAFF246637_F.nirenbergiae_Strawberry	671	--	671
20.MAFF246672_F.nirenbergiae_ChinesePeony	671	--	671
21.MAFF246697_F.commune_Urallicoriceroot	673	--	673
22.MAFF246729_F.falciforme_Angelica	691	--	691
23.MAFF247220_F.duplospermum__Euwallaceasp	694	--	694
24.MAFF410760_F.odoratissimum_alpha	669	--	669
25.MAFF244605_FusariumoxysporumSchlechtendal_MAFF244605_Tomato	672	--	672
26.MAFF241326_F.asiaticum_Wheat	669	--	669
27.MAFF245014_F.asiaticum_Wildsoybean	666	--	666
28.MAFF150124_F.asiaticum__Wheat	666	--	666
29.OM135603.1F.algeriense	699	--	699
30.MAFF237465_F.penzigii_Aloe	704	--	704
31.MAFF103054_F.oxysporumSchlechtendalf.sp.cucumerinum_Cucumber	671	--	671
32.MAFF712246_F.oxysporumSchlechtendalf.sp.dianthi__Carnation	673	--	673
33.MAFF305558_F.oxysporumSchlechtendalf.sp.fragariae__Watermelon	671	--	671
34.MAFF744087_F.oxysporumSchlechtendalf.sp.lactucae__Lettuce	671	--	671
35.MAFF726924_F.oxysporumSchlechtendalf.sp.lagenariae_Whitefloweredgourd	671	--	671
36.MAFF744003_F.oxysporumSchlechtendalf.sp.lagenariae_Squash)	672	--	672
37.MAFF305122_F.oxysporumSchlechtendalf.sp.melonis__Melon	671	--	671
38.MAFF306714_F.oxysporumSchlechtendalf.sp.momordicae_Balsampear	671	--	671
39.MAFF238905_F.oxysporumSchlechtendalf.sp.radicis-lycopersici_Tomato	674	--	674
40.MAFF150004_F.oxysporumSchlechtendalf.sp.spinaciae_Spinach	671	--	671
41.MAFF247034_F.oxysporumSchlechtendal__Goldenchain	671	--	671
42.MAFF245747_F.oxysporumSchlechtendalf.sp.callistephi__Chinaaster	671	--	671
43.MAFF305115_FoxysporumSchlechtendalf.sp.batatas__Sweatpotato	703	--	703
44.MAFF150126_F.asiaticum_Seed	666	--	666
45.MAFF246738_F.solani_Angelica	703	--	703
46.MAFF246664_F.cugenangense_Perilla	671	--	671
47.MH582420.1F.solanistrainMRC256	679	--	679
48.MAFF240361_F.babinda_Soil	693	--	693
49.MAFF242368_F.azukicola_Azukibean	708	--	708
50.MAFF241312_F.asiaticum_Soil,welshonionfield	666	--	666
51.LT548416.1_F.culmorumpartialtef1a	646	--	646
52.MAFF150124_F.asiaticum__Wheat	666	--	666
53.MAFF238806_F.begoniae_Oncidiumsp	705	--	705

```
54.MW594399.1_FusariumincarnatumisolateUD01C                        689 ---------------------------------------- 689
55.OP414923.1Pucciniagraminisf.sp.triticiisolateSHZPgt19            623 ---------------------------------------- 623
56.MT027094.1_BipolarisoryzaestrainOrL-2                            371 ---------------------------------------- 371
57.ON734360.1_AlternariaalternataisolateH126                        246 ---------------------------------------- 246
58.LC333578.1_StemphyliumlycopersiciSOasp2                            0 ---------------------------------------- 0
59.HQ718583.1_Colletotrichumgloeosporioidesisolateq-1               567 ---------------------------------------- 567
60.JN241603.1_AtheliarolfsiiisolateSR1                              667 TGGTTCAAGGGCTGGACTAAGGAGA---CCAAGGCTGGTG 703
61.KJ866474.1_RhizoctoniasolanistrainMHL-1                          439 TGGTACAAGGGCTGGACCAAGGAGA---CCAAGGCTGGTG 475
62.JQ672424.1AlternariatriticinaisolateEGS17-061                    623 ---------------------------------------- 623
63.LT707559.1_P.capsicipartialtef1agene                             471 ---------------------------------------- 471
64.MW090051.1_CurvularialunatastrainCls-3                           365 ---------------------------------------- 365
65.DQ400892.1_Aspergillusterreus                                    317 TGGTACAAGGGTTGGGAGAAGGAGG---GCAAGTCCGGCA 353
66.DQ911416.1_Pythiumsp.quercumstrainPy292                          450 ---------------------------------------- 450
67.EU797495.1_Phytophthorasp.oaksoilPoland                          450 ---------------------------------------- 450
68.HM148321.1_Cladosporiumcucumerinum                               385 ---------------------------------------- 385
69.AF398888.1_SclerotiniasclerotiorumisolateSS1                       0 ---------------------------------------- 0
70.AF398888.1_S.sclerotiorumisolateSS1                                0 ---------------------------------------- 0
71.HPAB545908.1_Verticilliumnonalfalfaeisolate                      299 TGGTACAAGGGCTGGGAGAAGGAGGGCGCCAAGGGTGCCA 338
72.EF433315.1_CeratocystisfimbriatavoucherCMW15052                  747 ---------------------------------------- 747
73.MN159912.1_Botrytiscinerea                                       390 ---------------------------------------- 390
74.MF034741.1_PeltasterfructicolaisolateSRB92                         0 ---------------------------------------- 0
75.LC440360.1_CercosporaasparagiCOasp2                                0 ---------------------------------------- 0
76.AY944105.1_MagnaportheoryzaeisolateSAG00T3()                     255 ---------------------------------------- 255
77.JX266586.1_CochliobolusmiyabeanusvoucherMFLUCC10-0733            380 ---------------------------------------- 380
78.MN393253.1_CorynesporacassiicolaisolateQHD001(MN393253.1UNVERIFIED)  0 ---------------------------------------- 0
79.MF375218.1_AgroatheliarolfsiiisolateBJB24                        372 TGGTTCAAGGGCTGGACTAAGGAGA---CCAAGGCTGGTG 408
80.MN106270.1_AgroatheliarolfsiistrainJ-12                          379 TGGTTCAAGGGCTGGACTAAGGAGA---CCAAGGCTGGTG 415
81.OQ732628.1_AgroatheliarolfsiiisolateBTCBSr3                      356 TGGTTCAAGGGCTGGACTAAGGAGA---CCAAGGCTGGTG 392
82.KY196185.1_ColletotrichumtruncatumstrainPAK53                    468 ---------------------------------------- 468
83.GU935835.1_ColletotrichumcoccodesisolateC96002                   973 ---------------------------------------- 973
84.MK085963.1_AlternariatenuissimaisolateSCCZ06                       0 ---------------------------------------- 0
85.MT548042.1_AlternarialongipesstrainKY_2019_012                     0 ---------------------------------------- 0
86.MN356465.1CalonectriamontanaisolateHSP4                          489 ---------------------------------------- 489
87.OL694224.1_CalonectriacanadianastrainF099                        504 ---------------------------------------- 504
88.MK803351.1_NeoscytalidiumdimidiatumstrainKale4-C                   0 ---------------------------------------- 0
89.ON376993.1_CurvulariachiangmaiensisisolateND00J7                 382 ---------------------------------------- 382
90.OQ383346.1_NeoscytalidiumdimidiatumisolateGKH-2                    0 ---------------------------------------- 0
91.MF662595.1_NeoscytalidiumnovaehollandiaeisolateNeNo1               0 ---------------------------------------- 0
92.EF560588.1Melampsoralini                                         656 ---------------------------------------- 656
93.LC590862.1_NeoscytalidiumdimidiatumPSU-HP01TEF1                    0 ---------------------------------------- 0
94.KX278106.1_BotryosphaeriaqingyuanensisstrainCERC2947               0 ---------------------------------------- 0
95.AJ578763.1_Blumeriagraminisf.sp.hordeicyp51                        0 ---------------------------------------- 0
96.MF490858.1_CurvulariadactylocteniicolastrainCPC28810             370 ---------------------------------------- 370
97.KT287115.1_Bipolariscactivoraisolate3.8.6                        424 ---------------------------------------- 424
98.MT560940.1_CurvulariacactivorastrainHLGH0118                     381 ---------------------------------------- 381
99.OM714565.1_CurvulariaplantarumstrainM0134                        407 ---------------------------------------- 407
```

```
100.MN159911.1_BotrytiscinereaSICAUCC19-0003                             390 ---------------------------------------- 390
102.GU294713.1LasiodiplodiatheobromaestrainUCD2430TX                       0 ---------------------------------------- 0
103.KX868094.1_Mycosphaerellasp.isolateCRM20.1                           475 ---------------------------------------- 475
104.LC599478.1Pseudocercosporapini-densifloraeMUCC534                      0 ---------------------------------------- 0
105.N584698.1BipolarissetariaestrainKBS4-2                               435 ---------------------------------------- 435

2.OM160859.1_F.buharicum                                                 560 ---------------------------------------- 560
1.LC727524.1_F.buharicum_OKI-1_Okura                                     682 ---------------------------------------- 682
3.KX302919.1_F.sublunatum                                                521 ---------------------------------------- 521
4.LT996094.1_F.convolutans                                               516 ---------------------------------------- 516
5.OM160861.1_F.abutilonis                                                630 ---------------------------------------- 630
6.OM160874.1_F.guadeloupense                                             560 ---------------------------------------- 560
7.MH392475.1_F.graminearum                                               430 ---------------------------------------- 430
8.MH582420.1_F.solani                                                    540 ---------------------------------------- 540
9.MAFF244605_F.oxysporum                                                 544 ---------------------------------------- 544
10.MAFF237278_F.contaminatum_Hylocereus                                  711 ---------------------------------------- 711
11.MAFF237649_F.concentricum__Riceroot                                   678 ---------------------------------------- 678
12.MAFF237650_F.concentricum__Wheat                                      679 ---------------------------------------- 679
13.MAFF239869_F.mangiferae__Ryukyupine                                   657 ---------------------------------------- 657
14.MAFF240460_F.fujikuroi_Passionfruit                                   661 ---------------------------------------- 661
15.MAFF241317_F.graminearum_Wheat                                        666 ---------------------------------------- 666
16.MAFF242670_F.ipomoeae_Wheat                                           672 ---------------------------------------- 672
17.MAFF245129_F.concentricum_Fraxinus                                    659 ---------------------------------------- 659
18.MAFF245395_F.cugenangense_Rhubarb                                     671 ---------------------------------------- 671
19.MAFF246637_F.nirenbergiae_Strawberry                                  671 ---------------------------------------- 671
20.MAFF246672_F.nirenbergiae_ChinesePeony                                671 ---------------------------------------- 671
21.MAFF246697_F.commune_Urallicoriceroot                                 673 ---------------------------------------- 673
22.MAFF246729_F.falciforme_Angelica                                      691 ---------------------------------------- 691
23.MAFF247220_F.duplospermum__Euwallaceasp                               694 ---------------------------------------- 694
24.MAFF410760_F.odoratissimum_alpha                                      669 ---------------------------------------- 669
25.MAFF244605_FusariumoxysporumSchlechtendal_MAFF244605_Tomato           672 ---------------------------------------- 672
26.MAFF241326_F.asiaticum_Wheat                                          669 ---------------------------------------- 669
27.MAFF245014_F.asiaticum_Wildsoybean                                    666 ---------------------------------------- 666
28.MAFF150124_F.asiaticum__Wheat                                         666 ---------------------------------------- 666
29.OM135603.1F.algeriense                                                699 ---------------------------------------- 699
30.MAFF237465_F.penzigii_Aloe                                            704 ---------------------------------------- 704
31.MAFF103054_F.oxysporumSchlechtendalf.sp.cucumerinum_Cucumber          671 ---------------------------------------- 671
32.MAFF712246_F.oxysporumSchlechtendalf.sp.dianthi__Carnation            673 ---------------------------------------- 673
33.MAFF305558_F.oxysporumSchlechtendalf.sp.fragariae__Watermelon         671 ---------------------------------------- 671
34.MAFF744087_F.oxysporumSchlechtendalf.sp.lactucae__Lettuce             671 ---------------------------------------- 671
35.MAFF726924_F.oxysporumSchlechtendalf.sp.lagenariae_Whitefloweredgourd 671 ---------------------------------------- 671
36.MAFF744003_F.oxysporumSchlechtendalf.sp.lagenariae_Squash)            672 ---------------------------------------- 672
37.MAFF305122_F.oxysporumSchlechtendalf.sp.melonis__Melon                671 ---------------------------------------- 671
38.MAFF306714_F.oxysporumSchlechtendalf.sp.momordicae_Balsampear         671 ---------------------------------------- 671
39.MAFF238905_F.oxysporumSchlechtendalf.sp.radicis-lycopersici_Tomato    674 ---------------------------------------- 674
40.MAFF150004_F.oxysporumSchlechtendalf.sp.spinaciae_Spinach             671 ---------------------------------------- 671
```

```
41.MAFF247034_F.oxysporumSchlechtendal__Goldenchain                     671 ---------------------------------------- 671
42.MAFF245747_F.oxysporumSchlechtendalf.sp.callistephi__Chinaaster      671 ---------------------------------------- 671
43.MAFF305115_FoxysporumSchlechtendalf.sp.batatas__Sweatpotato          703 ---------------------------------------- 703
44.MAFF150126_F.asiaticum_Seed                                          666 ---------------------------------------- 666
45.MAFF246738_F.solani_Angelica                                         703 ---------------------------------------- 703
46.MAFF246664_F.cugenangense_Perilla                                    671 ---------------------------------------- 671
47.MH582420.1F.solanistrainMRC256                                       679 ---------------------------------------- 679
48.MAFF240361_F.babinda_Soil                                            693 ---------------------------------------- 693
49.MAFF242368_F.azukicola_Azukibean                                     708 ---------------------------------------- 708
50.MAFF241312_F.asiaticum_Soil,welshonionfield                          666 ---------------------------------------- 666
51.LT548416.1_F.culmorumpartialtef1a                                    646 ---------------------------------------- 646
52.MAFF150124_F.asiaticum__Wheat                                        666 ---------------------------------------- 666
53.MAFF238806_F.begoniae_Oncidiumsp                                     705 ---------------------------------------- 705
54.MW594399.1_FusariumincarnatumisolateUD01C                            689 ---------------------------------------- 689
55.OP414923.1Pucciniagraminisf.sp.triticiisolateSHZPgt19                623 ---------------------------------------- 623
56.MT027094.1_BipolarisoryzaestrainOrL-2                                371 ---------------------------------------- 371
57.ON734360.1_AlternariaalternataisolateH126                            246 ---------------------------------------- 246
58.LC333578.1_StemphyliumlycopersiciSOasp2                                0 ---------------------------------------- 0
59.HQ718583.1_Colletotrichumgloeosporioidesisolateq-1                   567 ---------------------------------------- 567
60.JN241603.1_AtheliarolfsiiisolateSR1                                  704 TCGTTAAGGGCAAGACCCTCCTCGATGC-ATCGATGCCAT 742
61.KJ866474.1_RhizoctoniasolanistrainMHL-1                              476 TCACCAAGGGTAAGACCCTCCTCGACGCCATCGATGCTAT 515
62.JQ672424.1AlternariatriticinaisolateEGS17-061                         623 ---------------------------------------- 623
63.LT707559.1_P.capsicipartialtef1agene                                 471 ---------------------------------------- 471
64.MW090051.1_CurvularialunatastrainCls-3                               365 ---------------------------------------- 365
65.DQ400892.1_Aspergillusterreus                                        354 AGGTCACCGGTAAGACTCTCCTCGAGGCCATCGATGCCAT 393
66.DQ911416.1_Pythiumsp.quercumstrainPy292                              450 ---------------------------------------- 450
67.EU797495.1_Phytophthorasp.oaksoilPoland                              450 ---------------------------------------- 450
68.HM148321.1_Cladosporiumcucumerinum                                   385 ---------------------------------------- 385
69.AF398888.1_SclerotiniasclerotiorumisolateSS1                           0 ---------------------------------------- 0
70.AF398888.1_S.sclerotiorumisolateSS1                                    0 ---------------------------------------- 0
71.HPAB545908.1_Verticilliumnonalfalfaeisolate                          339 AGTCCACCGGCAAGACTCTTCTCGAGGCCATTGACGCCAT 378
72.EF433315.1_CeratocystisfimbriatavoucherCMW15052                      747 ---------------------------------------- 747
73.MN159912.1_Botrytiscinerea                                           390 ---------------------------------------- 390
74.MF034741.1_PeltasterfructicolaisolateSRB92                             0 ---------------------------------------- 0
75.LC440360.1_CercosporaasparagiCOasp2                                    1 ------------TTCGAGAAGGTGAGCAACTGCCAGCACA 28
76.AY944105.1_MagnaportheoryzaeisolateSAG00T3()                         255 ---------------------------------------- 255
77.JX266586.1_CochliobolusmiyabeanusvoucherMFLUCC10-0733                 380 ---------------------------------------- 380
78.MN393253.1_CorynesporacassiicolaisolateQHD001(MN393253.1UNVERIFIED)    0 ---------------------------------------- 0
79.MF375218.1_AgroatheliarolfsiiisolateBJB24                            409 TCGTTAAGGGCAAGACCCTCCTCGATGCCATCGATGCCAT 448
80.MN106270.1_AgroatheliarolfsiistrainJ-12                              416 TCGTTAAGGGCAAGACCCTCCTCGATGCCATCGATGCCAT 455
81.OQ732628.1_AgroatheliarolfsiiisolateBTCBSr3                          393 TCGTTAAGGGCAAGACCCTCCTCGATGCCATCGATGCCAT 432
82.KY196185.1_ColletotrichumtruncatumstrainPAK53                        468 ---------------------------------------- 468
83.GU935835.1_ColletotrichumcoccodesisolateC96002                       973 ---------------------------------------- 973
84.MK085963.1_AlternariatenuissimaisolateSCCZ06                           0 ---------------------------------------- 0
85.MT548042.1_AlternarialongipesstrainKY_2019_012                         0 ---------------------------------------- 0
86.MN356465.1CalonectriamontanaisolateHSP4                              489 ---------------------------------------- 489
```

```
87.OL694224.1_CalonectriacanadianastrainF099                    504 ---------------------------------------- 504
88.MK803351.1_NeoscytalidiumdimidiatumstrainKale4-C               0 ---------------------------------------- 0
89.ON376993.1_CurvulariachiangmaiensisisolateND00J7             382 ---------------------------------------- 382
90.OQ383346.1_NeoscytalidiumdimidiatumisolateGKH-2                0 ---------------------------------------- 0
91.MF662595.1_NeoscytalidiumnovaehollandiaeisolateNeNo1           0 ---------------------------------------- 0
92.EF560588.1Melampsoralini                                     656 ---------------------------------------- 656
93.LC590862.1_NeoscytalidiumdimidiatumPSU-HP01TEF1                0 ---------------------------------------- 0
94.KX278106.1_BotryosphaeriaqingyuanensisstrainCERC2947           0 ---------------------------------------- 0
95.AJ578763.1_Blumeriagraminisf.sp.hordeicyp51                    0 ---------------------------------------- 0
96.MF490858.1_CurvulariadactylocteniicolastrainCPC28810         370 ---------------------------------------- 370
97.KT287115.1_Bipolariscactivoraisolate3.8.6                    424 ---------------------------------------- 424
98.MT560940.1_CurvulariacactivorastrainHLGH0118                 381 ---------------------------------------- 381
99.OM714565.1_CurvulariaplantarumstrainM0134                    407 ---------------------------------------- 407
100.MN159911.1_BotrytiscinereaSICAUCC19-0003                    390 ---------------------------------------- 390
102.GU294713.1LasiodiplodiatheobromaestrainUCD2430TX              0 ---------------------------------------- 0
103.KX868094.1_Mycosphaerellasp.isolateCRM20.1                  475 ---------------------------------------- 475
104.LC599478.1Pseudocercosporapini-densifloraeMUCC534             0 ---------------------------------------- 0
105.N584698.1BipolarissetariaestrainKBS4-2                      435 ---------------------------------------- 435

2.OM160859.1_F.buharicum                                        560 ---------------------------------------- 560
1.LC727524.1_F.buharicum_OKI-1_Okura                            682 ---------------------------------------- 682
3.KX302919.1_F.sublunatum                                       521 ---------------------------------------- 521
4.LT996094.1_F.convolutans                                      516 ---------------------------------------- 516
5.OM160861.1_F.abutilonis                                       630 ---------------------------------------- 630
6.OM160874.1_F.guadeloupense                                    560 ---------------------------------------- 560
7.MH392475.1_F.graminearum                                      430 ---------------------------------------- 430
8.MH582420.1_F.solani                                           540 ---------------------------------------- 540
9.MAFF244605_F.oxysporum                                        544 ---------------------------------------- 544
10.MAFF237278_F.contaminatum_Hylocereus                         711 ---------------------------------------- 711
11.MAFF237649_F.concentricum__Riceroot                          678 ---------------------------------------- 678
12.MAFF237650_F.concentricum__Wheat                             679 ---------------------------------------- 679
13.MAFF239869_F.mangiferae__Ryukyupine                          657 ---------------------------------------- 657
14.MAFF240460_F.fujikuroi_Passionfruit                          661 ---------------------------------------- 661
15.MAFF241317_F.graminearum_Wheat                               666 ---------------------------------------- 666
16.MAFF242670_F.ipomoeae_Wheat                                  672 ---------------------------------------- 672
17.MAFF245129_F.concentricum_Fraxinus                           659 ---------------------------------------- 659
18.MAFF245395_F.cugenangense_Rhubarb                            671 ---------------------------------------- 671
19.MAFF246637_F.nirenbergiae_Strawberry                         671 ---------------------------------------- 671
20.MAFF246672_F.nirenbergiae_ChinesePeony                       671 ---------------------------------------- 671
21.MAFF246697_F.commune_Urallicoriceroot                        673 ---------------------------------------- 673
22.MAFF246729_F.falciforme_Angelica                             691 ---------------------------------------- 691
23.MAFF247220_F.duplospermum__Euwallaceasp                      694 ---------------------------------------- 694
24.MAFF410760_F.odoratissimum_alpha                             669 ---------------------------------------- 669
25.MAFF244605_FusariumoxysporumSchlechtendal_MAFF244605_Tomato  672 ---------------------------------------- 672
26.MAFF241326_F.asiaticum_Wheat                                 669 ---------------------------------------- 669
27.MAFF245014_F.asiaticum_Wildsoybean                           666 ---------------------------------------- 666
```

```
28.MAFF150124_F.asiaticum__Wheat                                    666 ---------------------------------------- 666
29.OM135603.1F.algeriense                                           699 ---------------------------------------- 699
30.MAFF237465_F.penzigii_Aloe                                       704 ---------------------------------------- 704
31.MAFF103054_F.oxysporumSchlechtendalf.sp.cucumerinum_Cucumber     671 ---------------------------------------- 671
32.MAFF712246_F.oxysporumSchlechtendalf.sp.dianthi__Carnation       673 ---------------------------------------- 673
33.MAFF305558_F.oxysporumSchlechtendalf.sp.fragariae__Watermelon    671 ---------------------------------------- 671
34.MAFF744087_F.oxysporumSchlechtendalf.sp.lactucae__Lettuce        671 ---------------------------------------- 671
35.MAFF726924_F.oxysporumSchlechtendalf.sp.lagenariae_Whiteflowered gourd 671 ---------------------------------------- 671
36.MAFF744003_F.oxysporumSchlechtendalf.sp.lagenariae_Squash)       672 ---------------------------------------- 672
37.MAFF305122_F.oxysporumSchlechtendalf.sp.melonis__Melon           671 ---------------------------------------- 671
38.MAFF306714_F.oxysporumSchlechtendalf.sp.momordicae_Balsampear    671 ---------------------------------------- 671
39.MAFF238905_F.oxysporumSchlechtendalf.sp.radicis-lycopersici_Tomato 674 ---------------------------------------- 674
40.MAFF150004_F.oxysporumSchlechtendalf.sp.spinaciae_Spinach        671 ---------------------------------------- 671
41.MAFF247034_F.oxysporumSchlechtendal__Goldenchain                 671 ---------------------------------------- 671
42.MAFF245747_F.oxysporumSchlechtendalf.sp.callistephi__Chinaaster  671 ---------------------------------------- 671
43.MAFF305115_FoxysporumSchlechtendalf.sp.batatas__Sweatpotato      703 ---------------------------------------- 703
44.MAFF150126_F.asiaticum_Seed                                      666 ---------------------------------------- 666
45.MAFF246738_F.solani_Angelica                                     703 ---------------------------------------- 703
46.MAFF246664_F.cugenangense_Perilla                                671 ---------------------------------------- 671
47.MH582420.1F.solanistrainMRC256                                   679 ---------------------------------------- 679
48.MAFF240361_F.babinda_Soil                                        693 ---------------------------------------- 693
49.MAFF242368_F.azukicola_Azukibean                                 708 ---------------------------------------- 708
50.MAFF241312_F.asiaticum_Soil,welshonionfield                      666 ---------------------------------------- 666
51.LT548416.1_F.culmorumpartialtef1a                                646 ---------------------------------------- 646
52.MAFF150124_F.asiaticum__Wheat                                    666 ---------------------------------------- 666
53.MAFF238806_F.begoniae_Oncidiumsp                                 705 ---------------------------------------- 705
54.MW594399.1_FusariumincarnatumisolateUD01C                        689 ---------------------------------------- 689
55.OP414923.1Pucciniagraminisf.sp.triticiisolateSHZPgt19            623 ---------------------------------------- 623
56.MT027094.1_BipolarisoryzaestrainOrL-2                            372 -------------CGTCCTACCGACAAGCCTCTC--CGTC 396
57.ON734360.1_AlternariaalternataisolateH126                        247 -------------CGTCCCACCGACAAGCCCCTC--CGTC 271
58.LC333578.1_StemphyliumlycopersiciSOasp2                            0 ---------------------------------------- 0
59.HQ718583.1_Colletotrichumgloeosporioidesisolateq-1               567 ---------------------------------------- 567
60.JN241603.1_AtheliarolfsiiisolateSR1                              743 CGAGCCCCCCGTCCGTCCCTCCGACAAGCCCCTC--CGTC 780
61.KJ866474.1_RhizoctoniasolanistrainMHL-1                          516 CGAGCCCCCCGTCCGTCCCTCCGACAAGCCTCTC--CGTC 553
62.JQ672424.1AlternariatriticinaisolateEGS17-061                    624 -------------CGTCCCACCGACAAGCCCCTC--CGCC 648
63.LT707559.1_P.capsicipartialtef1agene                             472 -------------CGTCCGTCTGACAAGCCCCTT--CGTC 496
64.MW090051.1_CurvularialunatastrainCls-3                           366 -------------CGTCCTACCGACAAGCCCCTC--CGCC 390
65.DQ400892.1_Aspergillusterreus                                    394 CGAGCCCCCCGTCCGTCCTGCCAACAAGCCCCTC--CGTC 431
66.DQ911416.1_Pythiumsp.quercumstrainPy292                          451 -------------CGTCCGTCGGACAAGCCGCTT--CGTC 475
67.EU797495.1_Phytophthorasp.oaksoilPoland                          451 -------------CGTCCCTCGGACAAGCCTCTG--CGTC 475
68.HM148321.1_Cladosporiumcucumerinum                               385 ---------------------------------------- 385
69.AF398888.1_SclerotiniasclerotiorumisolateSS1                       0 ---------------------------------------- 0
70.AF398888.1_S.sclerotiorumisolateSS1                                0 ---------------------------------------- 0
71.HPAB545908.1_Verticilliumnonalfalfaeisolate                      379 TGAGCAGCCCAAGCGTCCCACCGACAAGCCCCTC--CGTC 416
72.EF433315.1_CeratocystisfimbriatavoucherCMW15052                  747 ---------------------------------------- 747
73.MN159912.1_Botrytiscinerea                                       391 -------------CGCCCAACTGACAAGCCCCTC--CGTC 415
```

74.MF034741.1_PeltasterfructicolaisolateSRB92	0	--	0
75.LC440360.1_CercosporaasparagiCOasp2	29	CAAATGCATTTTGTCGCACAAATTTTCGCCGCTTGTCGCC	68
76.AY944105.1_MagnaportheoryzaeisolateSAG00T3()	255	--	255
77.JX266586.1_CochliobolusmiyabeanusvoucherMFLUCC10-0733	381	-------------CGTCCTACCGACAAGCCTCTC--CGTC	405
78.MN393253.1_CorynesporacassiicolaisolateQHD001(MN393253.1UNVERIFIED)	0	--	0
79.MF375218.1_AgroatheliarolfsiiisolateBJB24	449	CGAGCCCCCCGTCCGTCCCTCCGACAAGCCCCTC--CGTC	486
80.MN106270.1_AgroatheliarolfsiistrainJ-12	456	CGAGCCCCCCGTCCGTCCCTCCGACAAGCCCCTC--CGTC	493
81.OQ732628.1_AgroatheliarolfsiiisolateBTCBSr3	433	CGAGCCCCCCGTCCGTCCCTCCGACAAGCCCCTC--CGTC	470
82.KY196185.1_ColletotrichumtruncatumstrainPAK53	468	--	468
83.GU935835.1_ColletotrichumcoccodesisolateC96002	974	-------------CGCCCGACAGACAAGCCCCTC--CGTC	998
84.MK085963.1_AlternariatenuissimaisolateSCCZ06	0	--	0
85.MT548042.1_AlternarialongipesstrainKY_2019_012	0	--	0
86.MN356465.1CalonectriamontanaisolateHSP4	489	--	489
87.OL694224.1_CalonectriacanadianastrainF099	504	--	504
88.MK803351.1_NeoscytalidiumdimidiatumstrainKale4-C	0	--	0
89.ON376993.1_CurvulariachiangmaiensisisolateND00J7	383	-------------CGTCCTACCGACAAGCCCCTC--CGCC	407
90.OQ383346.1_NeoscytalidiumdimidiatumisolateGKH-2	0	--	0
91.MF662595.1_NeoscytalidiumnovaehollandiaeisolateNeNo1	0	--	0
92.EF560588.1Melampsoralini	656	--	656
93.LC590862.1_NeoscytalidiumdimidiatumPSU-HP01TEF1	0	--	0
94.KX278106.1_BotryosphaeriaqingyuanensisstrainCERC2947	0	--	0
95.AJ578763.1_Blumeriagraminisf.sp.hordeicyp51	0	--	0
96.MF490858.1_CurvulariadactylocteniicolastrainCPC28810	371	-------------CGTCCTACCGACAAGCCCCTC--CGCC	395
97.KT287115.1_Bipolariscactivoraisolate3.8.6	425	-------------CGTCCTACCGACAAGCCCCTC--CGTC	449
98.MT560940.1_CurvulariacactivorastrainHLGH0118	382	-------------CGTCCTACCGACAAGCCCCTC--CGTC	406
99.OM714565.1_CurvulariaplantarumstrainM0134	408	-------------CGTCCTACCGACAAGCCCCTC--CGCC	432
100.MN159911.1_BotrytiscinereaSICAUCC19-0003	391	-------------CGCCCAACTGACAAGCCCCTC--CGTC	415
102.GU294713.1LasiodiplodiatheobromaestrainUCD2430TX	0	--	0
103.KX868094.1_Mycosphaerellasp.isolateCRM20.1	475	--	475
104.LC599478.1Pseudocercosporapini-densifloraeMUCC534	0	--	0
105.N584698.1BipolarissetariaestrainKBS4-2	436	-------------CGTCCTACCGACAAGCCCCTC--CGTC	460
2.OM160859.1_F.buharicum	560	--	560
1.LC727524.1_F.buharicum_OKI-1_Okura	682	--	682
3.KX302919.1_F.sublunatum	521	--	521
4.LT996094.1_F.convolutans	516	--	516
5.OM160861.1_F.abutilonis	630	--	630
6.OM160874.1_F.guadeloupense	560	--	560
7.MH392475.1_F.graminearum	430	--	430
8.MH582420.1_F.solani	540	--	540
9.MAFF244605_F.oxysporum	544	--	544
10.MAFF237278_F.contaminatum_Hylocereus	711	--	711
11.MAFF237649_F.concentricum__Riceroot	678	--	678
12.MAFF237650_F.concentricum__Wheat	679	--	679
13.MAFF239869_F.mangiferae__Ryukyupine	657	--	657
14.MAFF240460_F.fujikuroi_Passionfruit	661	--	661

15.MAFF241317_F.graminearum_Wheat	666	--	666
16.MAFF242670_F.ipomoeae_Wheat	672	--	672
17.MAFF245129_F.concentricum_Fraxinus	659	--	659
18.MAFF245395_F.cugenangense_Rhubarb	671	--	671
19.MAFF246637_F.nirenbergiae_Strawberry	671	--	671
20.MAFF246672_F.nirenbergiae_ChinesePeony	671	--	671
21.MAFF246697_F.commune_Urallicoriceroot	673	--	673
22.MAFF246729_F.falciforme_Angelica	691	--	691
23.MAFF247220_F.duplospermum__Euwallaceasp	694	--	694
24.MAFF410760_F.odoratissimum_alpha	669	--	669
25.MAFF244605_FusariumoxysporumSchlechtendal_MAFF244605_Tomato	672	--	672
26.MAFF241326_F.asiaticum_Wheat	669	--	669
27.MAFF245014_F.asiaticum_Wildsoybean	666	--	666
28.MAFF150124_F.asiaticum__Wheat	666	--	666
29.OM135603.1F.algeriense	699	--	699
30.MAFF237465_F.penzigii_Aloe	704	--	704
31.MAFF103054_F.oxysporumSchlechtendalf.sp.cucumerinum_Cucumber	671	--	671
32.MAFF712246_F.oxysporumSchlechtendalf.sp.dianthi__Carnation	673	--	673
33.MAFF305558_F.oxysporumSchlechtendalf.sp.fragariae__Watermelon	671	--	671
34.MAFF744087_F.oxysporumSchlechtendalf.sp.lactucae__Lettuce	671	--	671
35.MAFF726924_F.oxysporumSchlechtendalf.sp.lagenariae_Whitefloweredgourd	671	--	671
36.MAFF744003_F.oxysporumSchlechtendalf.sp.lagenariae_Squash)	672	--	672
37.MAFF305122_F.oxysporumSchlechtendalf.sp.melonis__Melon	671	--	671
38.MAFF306714_F.oxysporumSchlechtendalf.sp.momordicae_Balsampear	671	--	671
39.MAFF238905_F.oxysporumSchlechtendalf.sp.radicis-lycopersici_Tomato	674	--	674
40.MAFF150004_F.oxysporumSchlechtendalf.sp.spinaciae_Spinach	671	--	671
41.MAFF247034_F.oxysporumSchlechtendal__Goldenchain	671	--	671
42.MAFF245747_F.oxysporumSchlechtendalf.sp.callistephi__Chinaaster	671	--	671
43.MAFF305115_FoxysporumSchlechtendalf.sp.batatas__Sweatpotato	703	--	703
44.MAFF150126_F.asiaticum_Seed	666	--	666
45.MAFF246738_F.solani_Angelica	703	--	703
46.MAFF246664_F.cugenangense_Perilla	671	--	671
47.MH582420.1F.solanistrainMRC256	679	--	679
48.MAFF240361_F.babinda_Soil	693	--	693
49.MAFF242368_F.azukicola_Azukibean	708	--	708
50.MAFF241312_F.asiaticum_Soil,welshonionfield	666	--	666
51.LT548416.1_F.culmorumpartialtef1a	646	--	646
52.MAFF150124_F.asiaticum__Wheat	666	--	666
53.MAFF238806_F.begoniae_Oncidiumsp	705	--	705
54.MW594399.1_FusariumincarnatumisolateUD01C	689	--	689
55.OP414923.1Pucciniagraminisf.sp.triticiisolateSHZPgt19	623	--	623
56.MT027094.1_BipolarisoryzaestrainOrL-2	397	TTCCCCTCCAGGATGTGTACAAGATCGGTGGTATTGGCAC	436
57.ON734360.1_AlternariaalternataisolateH126	272	TTCCCCTCCAGGATGTTTACAAGATTGGTGGTATTGGCAC	311
58.LC333578.1_StemphyliumlycopersiciSOasp2	0	--	0
59.HQ718583.1_Colletotrichumgloeosporioidesisolateq-1	567	--	567
60.JN241603.1_AtheliarolfsiiisolateSR1	781	TCCCTCTCCAGGATGTCTACAAGATCGGTGGTATCGGTAC	820

61.KJ866474.1_RhizoctoniasolanistrainMHL-1	554	TCCCACTCCAGGACGTCTACAAGATCGGTGGTATCGGAAC	593
62.JQ672424.1AlternariatriticinaisolateEGS17-061	649	TTCCCCTCCAGGATGTCTACAAGATTGGTGGTATTGGCAC	688
63.LT707559.1_P.capsicipartialtef1agene	497	TGCCCCTTCAGGATGTGTACAAGATCGGCGGTATCGGCAC	536
64.MW090051.1_CurvularialunatastrainCls-3	391	TTCCCCTCCAGGATGTCTACAAGATTGGTGGTATTGGCAC	430
65.DQ400892.1_Aspergillusterreus	432	TTCCCCTCCAGGATGTCTACA-------------------	452
66.DQ911416.1_Pythiumsp.quercumstrainPy292	476	TTCCTCTCCAGGACGTGTACAAGATCGGCGGTATTGGCAC	515
67.EU797495.1_Phytophthorasp.oaksoilPoland	476	TGCCCCTCCAGGACGTGTACAAGATCGGCGGTATCGGCAC	515
68.HM148321.1_Cladosporiumcucumerinum	385	--	385
69.AF398888.1_SclerotiniasclerotiorumisolateSS1	0	--	0
70.AF398888.1_S.sclerotiorumisolateSS1	0	--	0
71.HPAB545908.1_Verticilliumnonalfalfaeisolate	417	TTCCCCTCCAGGATGTCTACAAGATC--------------	442
72.EF433315.1_CeratocystisfimbriatavoucherCMW15052	747	--	747
73.MN159912.1_Botrytiscinerea	416	TCCCACTCCAAGATGTTTACAAGATTGGTGGTATTGGAAC	455
74.MF034741.1_PeltasterfructicolaisolateSRB92	0	--	0
75.LC440360.1_CercosporaasparagiCOasp2	69	T-CTGCGCTGGTGCCCCTCCAAAACTGGTGGGGTGCGAGA	107
76.AY944105.1_MagnaportheoryzaeisolateSAG00T3()	255	--	255
77.JX266586.1_CochliobolusmiyabeanusvoucherMFLUCC10-0733	406	TTCCCCTCCAGGATGTGTACAAGATCGGTGGTATTGGCAC	445
78.MN393253.1_CorynesporacassiicolaisolateQHD001(MN393253.1UNVERIFIED)	0	--	0
79.MF375218.1_AgroatheliarolfsiiisolateBJB24	487	TCCCTCTCCAGGATGTCTACAAGATCGGTGGTATCGGTAC	526
80.MN106270.1_AgroatheliarolfsiistrainJ-12	494	TCCCTCTCCAGGACGT------------------------	509
81.OQ732628.1_AgroatheliarolfsiiisolateBTCBSr3	471	TCCCTCTCCAGGATGTCTACAAGATCGGT-----------	499
82.KY196185.1_ColletotrichumtruncatumstrainPAK53	468	--	468
83.GU935835.1_ColletotrichumcoccodesisolateC96002	999	TTCCCCTCCAGGATGTCTACAAGATCGGTGGTATCGGCAC	1038
84.MK085963.1_AlternariatenuissimaisolateSCCZ06	0	--	0
85.MT548042.1_AlternarialongipesstrainKY_2019_012	0	--	0
86.MN356465.1CalonectriamontanaisolateHSP4	489	--	489
87.OL694224.1_CalonectriacanadianastrainF099	504	--	504
88.MK803351.1_NeoscytalidiumdimidiatumstrainKale4-C	0	--	0
89.ON376993.1_CurvulariachiangmaiensisisolateND00J7	408	TTCCCCTCCAGGATGTCTACAAGATTGGTGGTATTGGCAC	447
90.OQ383346.1_NeoscytalidiumdimidiatumisolateGKH-2	0	--	0
91.MF662595.1_NeoscytalidiumnovaehollandiaeisolateNeNo1	0	--	0
92.EF560588.1Melampsoralini	656	--	656
93.LC590862.1_NeoscytalidiumdimidiatumPSU-HP01TEF1	0	--	0
94.KX278106.1_BotryosphaeriaqingyuanensisstrainCERC2947	0	--	0
95.AJ578763.1_Blumeriagraminisf.sp.hordeicyp51	0	--	0
96.MF490858.1_CurvulariadactylocteniicolastrainCPC28810	396	TTCCCCTCCAGGATGTCTACAAGATTGGTGGTATTGGCAC	435
97.KT287115.1_Bipolariscactivoraisolate3.8.6	450	TTCCCCTCCAGGATGTGTACAAGATCGGTGGTATTGGCAC	489
98.MT560940.1_CurvulariacactivorastrainHLGH0118	407	TTCCCCTCCAGGATGTGTACAAGATCGGTGGTATTGGCAC	446
99.OM714565.1_CurvulariaplantarumstrainM0134	433	TTCCCCTCCAGGATGTCTACAAGATTGGTGGTATTGGCAC	472
100.MN159911.1_BotrytiscinereaSICAUCC19-0003	416	TCCCACTCCAAGATGTTTACAAGATTGGTGGTATTGGAAC	455
102.GU294713.1LasiodiplodiatheobromaestrainUCD2430TX	0	--	0
103.KX868094.1_Mycosphaerellasp.isolateCRM20.1	475	--	475
104.LC599478.1Pseudocercosporapini-densifloraeMUCC534	0	--	0
105.N584698.1BipolarissetariaestrainKBS4-2	461	TTCCCCTCCAGGATGTGTACAAGATCGGTGGTATTGGCAC	500
2.OM160859.1_F.buharicum	560	--	560

1.LC727524.1_F.buharicum_OKI-1_Okura	682	--	682
3.KX302919.1_F.sublunatum	521	--	521
4.LT996094.1_F.convolutans	516	--	516
5.OM160861.1_F.abutilonis	630	--	630
6.OM160874.1_F.guadeloupense	560	--	560
7.MH392475.1_F.graminearum	430	--	430
8.MH582420.1_F.solani	540	--	540
9.MAFF244605_F.oxysporum	544	--	544
10.MAFF237278_F.contaminatum_Hylocereus	711	--	711
11.MAFF237649_F.concentricum__Riceroot	678	--	678
12.MAFF237650_F.concentricum__Wheat	679	--	679
13.MAFF239869_F.mangiferae__Ryukyupine	657	--	657
14.MAFF240460_F.fujikuroi_Passionfruit	661	--	661
15.MAFF241317_F.graminearum_Wheat	666	--	666
16.MAFF242670_F.ipomoeae_Wheat	672	--	672
17.MAFF245129_F.concentricum_Fraxinus	659	--	659
18.MAFF245395_F.cugenangense_Rhubarb	671	--	671
19.MAFF246637_F.nirenbergiae_Strawberry	671	--	671
20.MAFF246672_F.nirenbergiae_ChinesePeony	671	--	671
21.MAFF246697_F.commune_Urallicoriceroot	673	--	673
22.MAFF246729_F.falciforme_Angelica	691	--	691
23.MAFF247220_F.duplospermum__Euwallaceasp	694	--	694
24.MAFF410760_F.odoratissimum_alpha	669	--	669
25.MAFF244605_FusariumoxysporumSchlechtendal_MAFF244605_Tomato	672	--	672
26.MAFF241326_F.asiaticum_Wheat	669	--	669
27.MAFF245014_F.asiaticum_Wildsoybean	666	--	666
28.MAFF150124_F.asiaticum__Wheat	666	--	666
29.OM135603.1F.algeriense	699	--	699
30.MAFF237465_F.penzigii_Aloe	704	--	704
31.MAFF103054_F.oxysporumSchlechtendalf.sp.cucumerinum_Cucumber	671	--	671
32.MAFF712246_F.oxysporumSchlechtendalf.sp.dianthi__Carnation	673	--	673
33.MAFF305558_F.oxysporumSchlechtendalf.sp.fragariae__Watermelon	671	--	671
34.MAFF744087_F.oxysporumSchlechtendalf.sp.lactucae__Lettuce	671	--	671
35.MAFF726924_F.oxysporumSchlechtendalf.sp.lagenariae_Whitefloweredgourd	671	--	671
36.MAFF744003_F.oxysporumSchlechtendalf.sp.lagenariae_Squash)	672	--	672
37.MAFF305122_F.oxysporumSchlechtendalf.sp.melonis__Melon	671	--	671
38.MAFF306714_F.oxysporumSchlechtendalf.sp.momordicae_Balsampear	671	--	671
39.MAFF238905_F.oxysporumSchlechtendalf.sp.radicis-lycopersici_Tomato	674	--	674
40.MAFF150004_F.oxysporumSchlechtendalf.sp.spinaciae_Spinach	671	--	671
41.MAFF247034_F.oxysporumSchlechtendal__Goldenchain	671	--	671
42.MAFF245747_F.oxysporumSchlechtendalf.sp.callistephi__Chinaaster	671	--	671
43.MAFF305115_FoxysporumSchlechtendalf.sp.batatas__Sweatpotato	703	--	703
44.MAFF150126_F.asiaticum_Seed	666	--	666
45.MAFF246738_F.solani_Angelica	703	--	703
46.MAFF246664_F.cugenangense_Perilla	671	--	671
47.MH582420.1F.solanistrainMRC256	679	--	679

```
48.MAFF240361_F.babinda_Soil                                    693 ---------------------------------------- 693
49.MAFF242368_F.azukicola_Azukibean                             708 ---------------------------------------- 708
50.MAFF241312_F.asiaticum_Soil,welshonionfield                  666 ---------------------------------------- 666
51.LT548416.1_F.culmorumpartialtef1a                            646 ---------------------------------------- 646
52.MAFF150124_F.asiaticum__Wheat                                666 ---------------------------------------- 666
53.MAFF238806_F.begoniae_Oncidiumsp                             705 ---------------------------------------- 705
54.MW594399.1_FusariumincarnatumisolateUD01C                    689 ---------------------------------------- 689
55.OP414923.1Pucciniagraminisf.sp.triticiisolateSHZPgt19        623 ---------------------------------------- 623
56.MT027094.1_BipolarisoryzaestrainOrL-2                        437 GGTTC----CCGTCGGTCGTGTCGAGACCGGTATCATCAA 472
57.ON734360.1_AlternariaalternataisolateH126                    312 GGTGC----CCGTCGGTCGTGTCGAGACCGGTATCATCAA 347
58.LC333578.1_StemphyliumlycopersiciSOasp2                        0 ---------------------------------------- 0
59.HQ718583.1_Colletotrichumgloeosporioidesisolateq-1           567 ---------------------------------------- 567
60.JN241603.1_AtheliarolfsiiisolateSR1                          821 GGTGC----CCGTCGGTCGTGTTGAGACTGGTGTCATCAA 856
61.KJ866474.1_RhizoctoniasolanistrainMHL-1                      594 AGTGC----CCGTCGGTCGTGTCGAGACTGGTGTCATCAA 629
62.JQ672424.1AlternariatriticinaisolateEGS17-061                689 GGTGC----CCGTCGGTCGTGTCGAGACCGGTATCATCAA 724
63.LT707559.1_P.capsicipartialtef1agene                         537 GGTAC----CTGTCGGCCGTGTGGAGACCGGTGTCATCAA 572
64.MW090051.1_CurvularialunatastrainCls-3                       431 GGTCC----CCGTCGGTCGTGTCGAGACCGGTATCATCAA 466
65.DQ400892.1_Aspergillusterreus                                452 ---------------------------------------- 452
66.DQ911416.1_Pythiumsp.quercumstrainPy292                      516 GGTTC----CGGTGGGCCGCGTCGAGACGGGTGTGCTCAA 551
67.EU797495.1_Phytophthorasp.oaksoilPoland                      516 GGTAC----CGGTCGGCCGTGTTGAGACGGGTGTCATCAA 551
68.HM148321.1_Cladosporiumcucumerinum                           385 ---------------------------------------- 385
69.AF398888.1_SclerotiniasclerotiorumisolateSS1                   0 ---------------------------------------- 0
70.AF398888.1_S.sclerotiorumisolateSS1                            0 ---------------------------------------- 0
71.HPAB545908.1_Verticilliumnonalfalfaeisolate                  442 ---------------------------------------- 442
72.EF433315.1_CeratocystisfimbriatavoucherCMW15052              747 ---------------------------------------- 747
73.MN159912.1_Botrytiscinerea                                   456 GGTGC----CAGTCGGTCGTGTTGAGACCGGTATCATCAA 491
74.MF034741.1_PeltasterfructicolaisolateSRB92                     0 ---------------------------------------- 0
75.LC440360.1_CercosporaasparagiCOasp2                          108 ATTTCGGCGCTTTGGGCTCTGCCGCTTGCGATGACTTCAT 147
76.AY944105.1_MagnaportheoryzaeisolateSAG00T3()                 255 ---------------------------------------- 255
77.JX266586.1_CochliobolusmiyabeanusvoucherMFLUCC10-0733        446 GGTTC----CCGTCGGTCGTGTCGAGACCGGTATCATCAA 481
78.MN393253.1_CorynesporacassiicolaisolateQHD001(MN393253.1UNVERIFIED)  0 ---------------------------------------- 0
79.MF375218.1_AgroatheliarolfsiiisolateBJB24                    527 GGTGC----CCGTCGGTCGTGT------------------ 544
80.MN106270.1_AgroatheliarolfsiistrainJ-12                      509 ---------------------------------------- 509
81.OQ732628.1_AgroatheliarolfsiiisolateBTCBSr3                  499 ---------------------------------------- 499
82.KY196185.1_ColletotrichumtruncatumstrainPAK53                468 ---------------------------------------- 468
83.GU935835.1_ColletotrichumcoccodesisolateC96002              1039 AGT------------------------------------- 1041
84.MK085963.1_AlternariatenuissimaisolateSCCZ06                   0 ---------------------------------------- 0
85.MT548042.1_AlternarialongipesstrainKY_2019_012                 0 ---------------------------------------- 0
86.MN356465.1CalonectriamontanaisolateHSP4                      489 ---------------------------------------- 489
87.OL694224.1_CalonectriacanadianastrainF099                    504 ---------------------------------------- 504
88.MK803351.1_NeoscytalidiumdimidiatumstrainKale4-C               0 ---------------------------------------- 0
89.ON376993.1_CurvulariachiangmaiensisisolateND00J7             448 GGTCC----CCGTCGGTCGTGTCGAGACCGGTATCATCAA 483
90.OQ383346.1_NeoscytalidiumdimidiatumisolateGKH-2                0 ---------------------------------------- 0
91.MF662595.1_NeoscytalidiumnovaehollandiaeisolateNeNo1           0 ---------------------------------------- 0
92.EF560588.1Melampsoralini                                     656 ---------------------------------------- 656
93.LC590862.1_NeoscytalidiumdimidiatumPSU-HP01TEF1                0 ---------------------------------------- 0
```

```
94.KX278106.1_BotryosphaeriaqingyuanensisstrainCERC2947          0 ---------------------------------------- 0
95.AJ578763.1_Blumeriagraminisf.sp.hordeicyp51                   0 ---------------------------------------- 0
96.MF490858.1_CurvulariadactylocteniicolastrainCPC28810        436 GGTCC----CCGTCGGTCGTGTCGAGACCGGTATCATCAA 471
97.KT287115.1_Bipolariscactivoraisolate3.8.6                   490 GGTCC----CCGTCGGTCGTGTCGAGACTGGTATCATCAA 525
98.MT560940.1_CurvulariacactivorastrainHLGH0118                447 GGTCC----CCGTCGGTCGTGTCGAGACTGGTATCATCAA 482
99.OM714565.1_CurvulariaplantarumstrainM0134                   473 GGTCC----CCGTCGGTCGTGTCGAGACCGGTATCATCAA 508
100.MN159911.1_BotrytiscinereaSICAUCC19-0003                   456 GGTGC----CAGTCGGTCGTGTTGAGACCGGTATCATCAA 491
102.GU294713.1LasiodiplodiatheobromaestrainUCD2430TX             0 ---------------------------------------- 0
103.KX868094.1_Mycosphaerellasp.isolateCRM20.1                 475 ---------------------------------------- 475
104.LC599478.1Pseudocercosporapini-densifloraeMUCC534            0 ---------------------------------------- 0
105.N584698.1BipolarissetariaestrainKBS4-2                     501 GGTTC----CCGTCGGTCGTGTCGAGACCGGTATCATCAA 536

2.OM160859.1_F.buharicum                                       560 ---------------------------------------- 560
1.LC727524.1_F.buharicum_OKI-1_Okura                           682 ---------------------------------------- 682
3.KX302919.1_F.sublunatum                                      521 ---------------------------------------- 521
4.LT996094.1_F.convolutans                                     516 ---------------------------------------- 516
5.OM160861.1_F.abutilonis                                      630 ---------------------------------------- 630
6.OM160874.1_F.guadeloupense                                   560 ---------------------------------------- 560
7.MH392475.1_F.graminearum                                     430 ---------------------------------------- 430
8.MH582420.1_F.solani                                          540 ---------------------------------------- 540
9.MAFF244605_F.oxysporum                                       544 ---------------------------------------- 544
10.MAFF237278_F.contaminatum_Hylocereus                        711 ---------------------------------------- 711
11.MAFF237649_F.concentricum__Riceroot                         678 ---------------------------------------- 678
12.MAFF237650_F.concentricum__Wheat                            679 ---------------------------------------- 679
13.MAFF239869_F.mangiferae__Ryukyupine                         657 ---------------------------------------- 657
14.MAFF240460_F.fujikuroi_Passionfruit                         661 ---------------------------------------- 661
15.MAFF241317_F.graminearum_Wheat                              666 ---------------------------------------- 666
16.MAFF242670_F.ipomoeae_Wheat                                 672 ---------------------------------------- 672
17.MAFF245129_F.concentricum_Fraxinus                          659 ---------------------------------------- 659
18.MAFF245395_F.cugenangense_Rhubarb                           671 ---------------------------------------- 671
19.MAFF246637_F.nirenbergiae_Strawberry                        671 ---------------------------------------- 671
20.MAFF246672_F.nirenbergiae_ChinesePeony                      671 ---------------------------------------- 671
21.MAFF246697_F.commune_Urallicoriceroot                       673 ---------------------------------------- 673
22.MAFF246729_F.falciforme_Angelica                            691 ---------------------------------------- 691
23.MAFF247220_F.duplospermum__Euwallaceasp                     694 ---------------------------------------- 694
24.MAFF410760_F.odoratissimum_alpha                            669 ---------------------------------------- 669
25.MAFF244605_FusariumoxysporumSchlechtendal_MAFF244605_Tomato 672 ---------------------------------------- 672
26.MAFF241326_F.asiaticum_Wheat                                669 ---------------------------------------- 669
27.MAFF245014_F.asiaticum_Wildsoybean                          666 ---------------------------------------- 666
28.MAFF150124_F.asiaticum__Wheat                               666 ---------------------------------------- 666
29.OM135603.1F.algeriense                                      699 ---------------------------------------- 699
30.MAFF237465_F.penzigii_Aloe                                  704 ---------------------------------------- 704
31.MAFF103054_F.oxysporumSchlechtendalf.sp.cucumerinum_Cucumber 671 ---------------------------------------- 671
32.MAFF712246_F.oxysporumSchlechtendalf.sp.dianthi__Carnation  673 ---------------------------------------- 673
33.MAFF305558_F.oxysporumSchlechtendalf.sp.fragariae__Watermelon 671 ---------------------------------------- 671
34.MAFF744087_F.oxysporumSchlechtendalf.sp.lactucae__Lettuce   671 ---------------------------------------- 671
```

```
35.MAFF726924_F.oxysporumSchlechtendalf.sp.lagenariae_Whitefloweredgourd 671 ---------------------------------------- 671
36.MAFF744003_F.oxysporumSchlechtendalf.sp.lagenariae_Squash) 672 ---------------------------------------- 672
37.MAFF305122_F.oxysporumSchlechtendalf.sp.melonis__Melon 671 ---------------------------------------- 671
38.MAFF306714_F.oxysporumSchlechtendalf.sp.momordicae_Balsampear 671 ---------------------------------------- 671
39.MAFF238905_F.oxysporumSchlechtendalf.sp.radicis-lycopersici_Tomato 674 ---------------------------------------- 674
40.MAFF150004_F.oxysporumSchlechtendalf.sp.spinaciae_Spinach 671 ---------------------------------------- 671
41.MAFF247034_F.oxysporumSchlechtendal__Goldenchain 671 ---------------------------------------- 671
42.MAFF245747_F.oxysporumSchlechtendalf.sp.callistephi__Chinaaster 671 ---------------------------------------- 671
43.MAFF305115_FoxysporumSchlechtendalf.sp.batatas__Sweatpotato 703 ---------------------------------------- 703
44.MAFF150126_F.asiaticum_Seed 666 ---------------------------------------- 666
45.MAFF246738_F.solani_Angelica 703 ---------------------------------------- 703
46.MAFF246664_F.cugenangense_Perilla 671 ---------------------------------------- 671
47.MH582420.1F.solanistrainMRC256 679 ---------------------------------------- 679
48.MAFF240361_F.babinda_Soil 693 ---------------------------------------- 693
49.MAFF242368_F.azukicola_Azukibean 708 ---------------------------------------- 708
50.MAFF241312_F.asiaticum_Soil,welshonionfield 666 ---------------------------------------- 666
51.LT548416.1_F.culmorumpartialtef1a 646 ---------------------------------------- 646
52.MAFF150124_F.asiaticum__Wheat 666 ---------------------------------------- 666
53.MAFF238806_F.begoniae_Oncidiumsp 705 ---------------------------------------- 705
54.MW594399.1_FusariumincarnatumisolateUD01C 689 ---------------------------------------- 689
55.OP414923.1Pucciniagraminisf.sp.triticiisolateSHZPgt19 623 ---------------------------------------- 623
56.MT027094.1_BipolarisoryzaestrainOrL-2 473 -GGCCGGTATGG-TCGTCACCTTCGCCCC-CGCTGGTGTC 509
57.ON734360.1_AlternariaalternataisolateH126 348 -GGCCGGTATGG-TCGTCACCTTCGCCCC-CGCTGGTGTC 384
58.LC333578.1_StemphyliumlycopersiciSOasp2 0 ---------------------------------------- 0
59.HQ718583.1_Colletotrichumgloeosporioidesisolateq-1 567 ---------------------------------------- 567
60.JN241603.1_AtheliarolfsiiisolateSR1 857 AGGCCGGTATGGGTCGTCACCTTCGCTCCCTTCCAACGTC 896
61.KJ866474.1_RhizoctoniasolanistrainMHL-1 630 -GGCTGGAATGA-TCGTTTCCTTTGCTCC-TACCAACGTG 666
62.JQ672424.1AlternariatriticinaisolateEGS17-061 725 -GGCCGGTATGG-TCGTCACCTTCGCCCC-CGCTGGTGTC 761
63.LT707559.1_P.capsicipartialtef1agene 573 -GCCTGGCATGG-TCGCCACTTTCGGCCC-CGTTGGTCTG 609
64.MW090051.1_CurvularialunatastrainCls-3 467 -GCCCGGTATGG-TCGTCACCTTCGCCCC-CGCTGGTGTC 503
65.DQ400892.1_Aspergillusterreus 452 ---------------------------------------- 452
66.DQ911416.1_Pythiumsp.quercumstrainPy292 552 -GCCGGGCATGG-TCGCGACGTTCGGCCC-TGTGGGTCTG 588
67.EU797495.1_Phytophthorasp.oaksoilPoland 552 -GCCCGGCATGG-TTGCCACCTTCGGCCC-CGTNGGTCTG 588
68.HM148321.1_Cladosporiumcucumerinum 385 ---------------------------------------- 385
69.AF398888.1_SclerotiniasclerotiorumisolateSS1 0 ---------------------------------------- 0
70.AF398888.1_S.sclerotiorumisolateSS1 0 ---------------------------------------- 0
71.HPAB545908.1_Verticilliumnonalfalfaeisolate 442 ---------------------------------------- 442
72.EF433315.1_CeratocystisfimbriatavoucherCMW15052 747 ---------------------------------------- 747
73.MN159912.1_Botrytiscinerea 492 -GGCCGGTATGG-TCGTTACCTTCGCCCC-AGCTGGTGTC 528
74.MF034741.1_PeltasterfructicolaisolateSRB92 0 ---------------------------------------- 0
75.LC440360.1_CercosporaasparagiCOasp2 148 ---CCGCTATGACTCCTCGCCCACCGCTCAACGCATTGGG 184
76.AY944105.1_MagnaportheoryzaeisolateSAG00T3() 255 ---------------------------------------- 255
77.JX266586.1_CochliobolusmiyabeanusvoucherMFLUCC10-0733 482 -GGCCGGTATGG-TCGTCACCTTCGCCCC-CGCTGGTGTC 518
78.MN393253.1_CorynesporacassiicolaisolateQHD001(MN393253.1UNVERIFIED) 0 ---------------------------------------- 0
79.MF375218.1_AgroatheliarolfsiiisolateBJB24 544 ---------------------------------------- 544
80.MN106270.1_AgroatheliarolfsiistrainJ-12 509 ---------------------------------------- 509
```

```
81.OQ732628.1_AgroatheliarolfsiiisolateBTCBSr3              499 ---------------------------------------- 499
82.KY196185.1_ColletotrichumtruncatumstrainPAK53            468 ---------------------------------------- 468
83.GU935835.1_ColletotrichumcoccodesisolateC96002          1041 ---------------------------------------- 1041
84.MK085963.1_AlternariatenuissimaisolateSCCZ06               0 ---------------------------------------- 0
85.MT548042.1_AlternarialongipesstrainKY_2019_012             0 ---------------------------------------- 0
86.MN356465.1CalonectriamontanaisolateHSP4                  489 ---------------------------------------- 489
87.OL694224.1_CalonectriacanadianastrainF099                504 ---------------------------------------- 504
88.MK803351.1_NeoscytalidiumdimidiatumstrainKale4-C           0 ---------------------------------------- 0
89.ON376993.1_CurvulariachiangmaiensisisolateND00J7         484 -GCCCGGTATGG-TCGTCACCTTCGCCCC-CGCTGGTGTC 520
90.OQ383346.1_NeoscytalidiumdimidiatumisolateGKH-2            0 ---------------------------------------- 0
91.MF662595.1_NeoscytalidiumnovaehollandiaeisolateNeNo1       0 ---------------------------------------- 0
92.EF560588.1Melampsoralini                                 656 ---------------------------------------- 656
93.LC590862.1_NeoscytalidiumdimidiatumPSU-HP01TEF1            0 ---------------------------------------- 0
94.KX278106.1_BotryosphaeriaqingyuanensisstrainCERC2947       0 ---------------------------------------- 0
95.AJ578763.1_Blumeriagraminisf.sp.hordeicyp51                0 ---------------------------------------- 0
96.MF490858.1_CurvulariadactylocteniicolastrainCPC28810     472 -GCCCGGTATGG-TCGTCACCTTCGCCCC-CGCTGGTGTC 508
97.KT287115.1_Bipolariscactivoraisolate3.8.6                526 -GGCCGGTATGG-TCGTCACCTTCGCCCC-CGCTGGTGTC 562
98.MT560940.1_CurvulariacactivorastrainHLGH0118             483 -GGCCGGTATGG-TCGTCACCTTCGCCCC-CGCTGGTGTC 519
99.OM714565.1_CurvulariaplantarumstrainM0134                509 -GCCCGGTATGG-TCGTCACCTTCGCCCC-CGCTGGTGTC 545
100.MN159911.1_BotrytiscinereaSICAUCC19-0003                492 -GGCCGGTATGG-TCGTTACCTTCGCCCC-AGCTGGTGTC 528
102.GU294713.1LasiodiplodiatheobromaestrainUCD2430TX          0 ---------------------------------------- 0
103.KX868094.1_Mycosphaerellasp.isolateCRM20.1              475 ---------------------------------------- 475
104.LC599478.1Pseudocercosporapini-densifloraeMUCC534         0 ---------------------------------------- 0
105.N584698.1BipolarissetariaestrainKBS4-2                  537 -GGCCGGTATGG-TCGTCACCTTCGCCCC-CGCTGGTGTC 573

2.OM160859.1_F.buharicum                                    560 ---------------------------------------- 560
1.LC727524.1_F.buharicum_OKI-1_Okura                        682 ---------------------------------------- 682
3.KX302919.1_F.sublunatum                                   521 ---------------------------------------- 521
4.LT996094.1_F.convolutans                                  516 ---------------------------------------- 516
5.OM160861.1_F.abutilonis                                   630 ---------------------------------------- 630
6.OM160874.1_F.guadeloupense                                560 ---------------------------------------- 560
7.MH392475.1_F.graminearum                                  430 ---------------------------------------- 430
8.MH582420.1_F.solani                                       540 ---------------------------------------- 540
9.MAFF244605_F.oxysporum                                    544 ---------------------------------------- 544
10.MAFF237278_F.contaminatum_Hylocereus                     711 ---------------------------------------- 711
11.MAFF237649_F.concentricum__Riceroot                      678 ---------------------------------------- 678
12.MAFF237650_F.concentricum__Wheat                         679 ---------------------------------------- 679
13.MAFF239869_F.mangiferae__Ryukyupine                      657 ---------------------------------------- 657
14.MAFF240460_F.fujikuroi_Passionfruit                      661 ---------------------------------------- 661
15.MAFF241317_F.graminearum_Wheat                           666 ---------------------------------------- 666
16.MAFF242670_F.ipomoeae_Wheat                              672 ---------------------------------------- 672
17.MAFF245129_F.concentricum_Fraxinus                       659 ---------------------------------------- 659
18.MAFF245395_F.cugenangense_Rhubarb                        671 ---------------------------------------- 671
19.MAFF246637_F.nirenbergiae_Strawberry                     671 ---------------------------------------- 671
20.MAFF246672_F.nirenbergiae_ChinesePeony                   671 ---------------------------------------- 671
21.MAFF246697_F.commune_Urallicoriceroot                    673 ---------------------------------------- 673
```

```
22.MAFF246729_F.falciforme_Angelica                                   691 ---------------------------------------- 691
23.MAFF247220_F.duplospermum__Euwallaceasp                            694 ---------------------------------------- 694
24.MAFF410760_F.odoratissimum_alpha                                   669 ---------------------------------------- 669
25.MAFF244605_FusariumoxysporumSchlechtendal_MAFF244605_Tomato        672 ---------------------------------------- 672
26.MAFF241326_F.asiaticum_Wheat                                       669 ---------------------------------------- 669
27.MAFF245014_F.asiaticum_Wildsoybean                                 666 ---------------------------------------- 666
28.MAFF150124_F.asiaticum__Wheat                                      666 ---------------------------------------- 666
29.OM135603.1F.algeriense                                             699 ---------------------------------------- 699
30.MAFF237465_F.penzigii_Aloe                                         704 ---------------------------------------- 704
31.MAFF103054_F.oxysporumSchlechtendalf.sp.cucumerinum_Cucumber       671 ---------------------------------------- 671
32.MAFF712246_F.oxysporumSchlechtendalf.sp.dianthi__Carnation         673 ---------------------------------------- 673
33.MAFF305558_F.oxysporumSchlechtendalf.sp.fragariae__Watermelon      671 ---------------------------------------- 671
34.MAFF744087_F.oxysporumSchlechtendalf.sp.lactucae__Lettuce          671 ---------------------------------------- 671
35.MAFF726924_F.oxysporumSchlechtendalf.sp.lagenariae_Whitefloweredgourd 671 ---------------------------------------- 671
36.MAFF744003_F.oxysporumSchlechtendalf.sp.lagenariae_Squash)         672 ---------------------------------------- 672
37.MAFF305122_F.oxysporumSchlechtendalf.sp.melonis__Melon             671 ---------------------------------------- 671
38.MAFF306714_F.oxysporumSchlechtendalf.sp.momordicae_Balsampear      671 ---------------------------------------- 671
39.MAFF238905_F.oxysporumSchlechtendalf.sp.radicis-lycopersici_Tomato 674 ---------------------------------------- 674
40.MAFF150004_F.oxysporumSchlechtendalf.sp.spinaciae_Spinach          671 ---------------------------------------- 671
41.MAFF247034_F.oxysporumSchlechtendal__Goldenchain                   671 ---------------------------------------- 671
42.MAFF245747_F.oxysporumSchlechtendalf.sp.callistephi__Chinaaster    671 ---------------------------------------- 671
43.MAFF305115_FoxysporumSchlechtendalf.sp.batatas__Sweatpotato        703 ---------------------------------------- 703
44.MAFF150126_F.asiaticum_Seed                                        666 ---------------------------------------- 666
45.MAFF246738_F.solani_Angelica                                       703 ---------------------------------------- 703
46.MAFF246664_F.cugenangense_Perilla                                  671 ---------------------------------------- 671
47.MH582420.1F.solanistrainMRC256                                     679 ---------------------------------------- 679
48.MAFF240361_F.babinda_Soil                                          693 ---------------------------------------- 693
49.MAFF242368_F.azukicola_Azukibean                                   708 ---------------------------------------- 708
50.MAFF241312_F.asiaticum_Soil,welshonionfield                        666 ---------------------------------------- 666
51.LT548416.1_F.culmorumpartialtef1a                                  646 ---------------------------------------- 646
52.MAFF150124_F.asiaticum__Wheat                                      666 ---------------------------------------- 666
53.MAFF238806_F.begoniae_Oncidiumsp                                   705 ---------------------------------------- 705
54.MW594399.1_FusariumincarnatumisolateUD01C                          689 ---------------------------------------- 689
55.OP414923.1Pucciniagraminisf.sp.triticiisolateSHZPgt19              623 ---------------------------------------- 623
56.MT027094.1_BipolarisoryzaestrainOrL-2                              510 ACCACTGAGGTCAAGTCCGTCGAGATGCACCACGAGCAGC 549
57.ON734360.1_AlternariaalternataisolateH126                          385 ACCACTGAAGTCAAGTCCGTCGAGATGCACCACGAGCAGC 424
58.LC333578.1_StemphyliumlycopersiciSOasp2                              0 ---------------------------------------- 0
59.HQ718583.1_Colletotrichumgloeosporioidesisolateq-1                 567 ---------------------------------------- 567
60.JN241603.1_AtheliarolfsiiisolateSR1                                897 ACCACCGAAGTCAAGTCCGTCGAAATGCACCACGAGCAGC 936
61.KJ866474.1_RhizoctoniasolanistrainMHL-1                            667 ACCACTGAAGTCAAGTC----------------------- 683
62.JQ672424.1AlternariatriticinaisolateEGS17-061                      762 ACCACTGAGGTCAAGTCCGTCGAGATGCACCACGAGCAGC 801
63.LT707559.1_P.capsicipartialtef1agene                               610 TCGACTGAAGTTAAGTCCGTGGAGATGCACCACGAGTCCC 649
64.MW090051.1_CurvularialunatastrainCls-3                             504 ACCACTGAAGTCAAGTCCGTCGAGATGCACCACGAGCAGC 543
65.DQ400892.1_Aspergillusterreus                                      452 ---------------------------------------- 452
66.DQ911416.1_Pythiumsp.quercumstrainPy292                            589 TCGACGGAAGTCAAGTCGGTGGAGATGCACCACGAGTCGC 628
67.EU797495.1_Phytophthorasp.oaksoilPoland                            589 TCGACGGAAGTCAAGTCCGTTGAGATGCACCACGAGTCCC 628
```

```
68.HM148321.1_Cladosporiumcucumerinum                                   385 ---------------------------------------- 385
69.AF398888.1_SclerotiniasclerotiorumisolateSS1                           0 ---------------------------------------- 0
70.AF398888.1_S.sclerotiorumisolateSS1                                    0 ---------------------------------------- 0
71.HPAB545908.1_Verticilliumnonalfalfaeisolate                          442 ---------------------------------------- 442
72.EF433315.1_CeratocystisfimbriatavoucherCMW15052                      747 ---------------------------------------- 747
73.MN159912.1_Botrytiscinerea                                           529 ACCACTGAAGTCAAGTCCGTCGAGATGCACCACGAACAAC 568
74.MF034741.1_PeltasterfructicolaisolateSRB92                             0 ---------------------------------------- 0
75.LC440360.1_CercosporaasparagiCOasp2                                  185 CATACCGCCAGCACA-CCACACATCTGCACGACCACACTT 223
76.AY944105.1_MagnaportheoryzaeisolateSAG00T3()                         255 ---------------------------------------- 255
77.JX266586.1_CochliobolusmiyabeanusvoucherMFLUCC10-0733                519 ACCACTGAGGTCAAGTCCGTCGAGATGCACCACGAGCAGC 558
78.MN393253.1_CorynesporacassiicolaisolateQHD001(MN393253.1UNVERIFIED)    0 ---------------------------------------- 0
79.MF375218.1_AgroatheliarolfsiiisolateBJB24                            544 ---------------------------------------- 544
80.MN106270.1_AgroatheliarolfsiistrainJ-12                              509 ---------------------------------------- 509
81.OQ732628.1_AgroatheliarolfsiiisolateBTCBSr3                          499 ---------------------------------------- 499
82.KY196185.1_ColletotrichumtruncatumstrainPAK53                        468 ---------------------------------------- 468
83.GU935835.1_ColletotrichumcoccodesisolateC96002                      1041 ---------------------------------------- 1041
84.MK085963.1_AlternariatenuissimaisolateSCCZ06                           0 ---------------------------------------- 0
85.MT548042.1_AlternarialongipesstrainKY_2019_012                         0 ---------------------------------------- 0
86.MN356465.1CalonectriamontanaisolateHSP4                              489 ---------------------------------------- 489
87.OL694224.1_CalonectriacanadianastrainF099                            504 ---------------------------------------- 504
88.MK803351.1_NeoscytalidiumdimidiatumstrainKale4-C                       0 ---------------------------------------- 0
89.ON376993.1_CurvulariachiangmaiensisisolateND00J7                     521 ACCACTGAAGTCAAGTCCGTCGAGATGCACCACGAGCAGC 560
90.OQ383346.1_NeoscytalidiumdimidiatumisolateGKH-2                        0 ---------------------------------------- 0
91.MF662595.1_NeoscytalidiumnovaehollandiaeisolateNeNo1                   0 ---------------------------------------- 0
92.EF560588.1Melampsoralini                                             656 ---------------------------------------- 656
93.LC590862.1_NeoscytalidiumdimidiatumPSU-HP01TEF1                        0 ---------------------------------------- 0
94.KX278106.1_BotryosphaeriaqingyuanensisstrainCERC2947                   0 ---------------------------------------- 0
95.AJ578763.1_Blumeriagraminisf.sp.hordeicyp51                            0 ---------------------------------------- 0
96.MF490858.1_CurvulariadactylocteniicolastrainCPC28810                 509 ACCACTGAAGTCAAGTCCGTCGAGATGCACCACGAGCAGC 548
97.KT287115.1_Bipolariscactivoraisolate3.8.6                            563 ACCACTGAGGTCAAGTCCGTCGAGATGCACCACGAGCAGC 602
98.MT560940.1_CurvulariacactivorastrainHLGH0118                         520 ACCACTGAGGTCAAGTCCGTCGAGATGCACCACGAGCAGC 559
99.OM714565.1_CurvulariaplantarumstrainM0134                            546 ACCACCGAAGTCAAGTCCGTCGAGATGCACCACGAGCAGC 585
100.MN159911.1_BotrytiscinereaSICAUCC19-0003                            529 ACCACTGAAGTCAAGTCCGTCGAGATGCACCACGAACAAC 568
102.GU294713.1LasiodiplodiatheobromaestrainUCD2430TX                      0 ---------------------------------------- 0
103.KX868094.1_Mycosphaerellasp.isolateCRM20.1                          475 ---------------------------------------- 475
104.LC599478.1Pseudocercosporapini-densifloraeMUCC534                     0 ---------------------------------------- 0
105.N584698.1BipolarissetariaestrainKBS4-2                              574 ACCACTGAAGTCAAGTCCGTCGAGATGCACCACGAGCAGC 613

2.OM160859.1_F.buharicum                                                560 ---------------------------------------- 560
1.LC727524.1_F.buharicum_OKI-1_Okura                                    682 ---------------------------------------- 682
3.KX302919.1_F.sublunatum                                               521 ---------------------------------------- 521
4.LT996094.1_F.convolutans                                              516 ---------------------------------------- 516
5.OM160861.1_F.abutilonis                                               630 ---------------------------------------- 630
6.OM160874.1_F.guadeloupense                                            560 ---------------------------------------- 560
7.MH392475.1_F.graminearum                                              430 ---------------------------------------- 430
8.MH582420.1_F.solani                                                   540 ---------------------------------------- 540
```

```
9.MAFF244605_F.oxysporum                                             544 ---------------------------------------- 544
10.MAFF237278_F.contaminatum_Hylocereus                              711 ---------------------------------------- 711
11.MAFF237649_F.concentricum__Riceroot                               678 ---------------------------------------- 678
12.MAFF237650_F.concentricum__Wheat                                  679 ---------------------------------------- 679
13.MAFF239869_F.mangiferae__Ryukyupine                               657 ---------------------------------------- 657
14.MAFF240460_F.fujikuroi_Passionfruit                               661 ---------------------------------------- 661
15.MAFF241317_F.graminearum_Wheat                                    666 ---------------------------------------- 666
16.MAFF242670_F.ipomoeae_Wheat                                       672 ---------------------------------------- 672
17.MAFF245129_F.concentricum_Fraxinus                                659 ---------------------------------------- 659
18.MAFF245395_F.cugenangense_Rhubarb                                 671 ---------------------------------------- 671
19.MAFF246637_F.nirenbergiae_Strawberry                              671 ---------------------------------------- 671
20.MAFF246672_F.nirenbergiae_ChinesePeony                            671 ---------------------------------------- 671
21.MAFF246697_F.commune_Urallicoriceroot                             673 ---------------------------------------- 673
22.MAFF246729_F.falciforme_Angelica                                  691 ---------------------------------------- 691
23.MAFF247220_F.duplospermum__Euwallaceasp                           694 ---------------------------------------- 694
24.MAFF410760_F.odoratissimum_alpha                                  669 ---------------------------------------- 669
25.MAFF244605_FusariumoxysporumSchlechtendal_MAFF244605_Tomato       672 ---------------------------------------- 672
26.MAFF241326_F.asiaticum_Wheat                                      669 ---------------------------------------- 669
27.MAFF245014_F.asiaticum_Wildsoybean                                666 ---------------------------------------- 666
28.MAFF150124_F.asiaticum__Wheat                                     666 ---------------------------------------- 666
29.OM135603.1F.algeriense                                            699 ---------------------------------------- 699
30.MAFF237465_F.penzigii_Aloe                                        704 ---------------------------------------- 704
31.MAFF103054_F.oxysporumSchlechtendalf.sp.cucumerinum_Cucumber      671 ---------------------------------------- 671
32.MAFF712246_F.oxysporumSchlechtendalf.sp.dianthi__Carnation        673 ---------------------------------------- 673
33.MAFF305558_F.oxysporumSchlechtendalf.sp.fragariae__Watermelon     671 ---------------------------------------- 671
34.MAFF744087_F.oxysporumSchlechtendalf.sp.lactucae__Lettuce         671 ---------------------------------------- 671
35.MAFF726924_F.oxysporumSchlechtendalf.sp.lagenariae_Whiteflowered gourd 671 ---------------------------------------- 671
36.MAFF744003_F.oxysporumSchlechtendalf.sp.lagenariae_Squash)        672 ---------------------------------------- 672
37.MAFF305122_F.oxysporumSchlechtendalf.sp.melonis__Melon            671 ---------------------------------------- 671
38.MAFF306714_F.oxysporumSchlechtendalf.sp.momordicae_Balsampear     671 ---------------------------------------- 671
39.MAFF238905_F.oxysporumSchlechtendalf.sp.radicis-lycopersici_Tomato 674 ---------------------------------------- 674
40.MAFF150004_F.oxysporumSchlechtendalf.sp.spinaciae_Spinach         671 ---------------------------------------- 671
41.MAFF247034_F.oxysporumSchlechtendal__Goldenchain                  671 ---------------------------------------- 671
42.MAFF245747_F.oxysporumSchlechtendalf.sp.callistephi__Chinaaster   671 ---------------------------------------- 671
43.MAFF305115_FoxysporumSchlechtendalf.sp.batatas__Sweatpotato       703 ---------------------------------------- 703
44.MAFF150126_F.asiaticum_Seed                                       666 ---------------------------------------- 666
45.MAFF246738_F.solani_Angelica                                      703 ---------------------------------------- 703
46.MAFF246664_F.cugenangense_Perilla                                 671 ---------------------------------------- 671
47.MH582420.1F.solanistrainMRC256                                    679 ---------------------------------------- 679
48.MAFF240361_F.babinda_Soil                                         693 ---------------------------------------- 693
49.MAFF242368_F.azukicola_Azukibean                                  708 ---------------------------------------- 708
50.MAFF241312_F.asiaticum_Soil,welshonionfield                       666 ---------------------------------------- 666
51.LT548416.1_F.culmorumpartialtef1a                                 646 ---------------------------------------- 646
52.MAFF150124_F.asiaticum__Wheat                                     666 ---------------------------------------- 666
53.MAFF238806_F.begoniae_Oncidiumsp                                  705 ---------------------------------------- 705
54.MW594399.1_FusariumincarnatumisolateUD01C                         689 ---------------------------------------- 689
```

```
55.OP414923.1Pucciniagraminisf.sp.triticiisolateSHZPgt19                  623 ---------------------------------------- 623
56.MT027094.1_BipolarisoryzaestrainOrL-2                                  550 TGACCGAGGGTGTCCCCGGTGACAACGTCGG-TTTCAACG 588
57.ON734360.1_AlternariaalternataisolateH126                              425 TCACCGAGGGTGTCCCCGGTGACAACGTCGG-CTTCAACG 463
58.LC333578.1_StemphyliumlycopersiciSOasp2                                  0 ---------------------------------------- 0
59.HQ718583.1_Colletotrichumgloeosporioidesisolateq-1                     567 ---------------------------------------- 567
60.JN241603.1_AtheliarolfsiiisolateSR1                                    937 TCGTTGAGGGTGTTCCCGGTGACAACGTCGGGTTTCAACG 976
61.KJ866474.1_RhizoctoniasolanistrainMHL-1                                683 ---------------------------------------- 683
62.JQ672424.1AlternariatriticinaisolateEGS17-061                          802 TTGTCGAGGGTGTCCCCGGTGACA---------------- 825
63.LT707559.1_P.capsicipartialtef1agene                                   650 TGCCGGAGGCTGTCCCTGGTGACAACGTCGG-CTTCAACG 688
64.MW090051.1_CurvularialunatastrainCls-3                                 544 TCACCGAGGGTGTCCCCGGTGACAACGTCGG-CTTCAACG 582
65.DQ400892.1_Aspergillusterreus                                          452 ---------------------------------------- 452
66.DQ911416.1_Pythiumsp.quercumstrainPy292                                629 TGCCGGAGGCTGTCCCGGGTGACAACGTTGG-CTTCAACG 667
67.EU797495.1_Phytophthorasp.oaksoilPoland                                629 TGGCGGAGGCTGTCCCCGGTGACAACGTTGG-CTTCAACG 667
68.HM148321.1_Cladosporiumcucumerinum                                     385 ---------------------------------------- 385
69.AF398888.1_SclerotiniasclerotiorumisolateSS1                             0 ---------------------------------------- 0
70.AF398888.1_S.sclerotiorumisolateSS1                                      0 ---------------------------------------- 0
71.HPAB545908.1_Verticilliumnonalfalfaeisolate                            442 ---------------------------------------- 442
72.EF433315.1_CeratocystisfimbriatavoucherCMW15052                        747 ---------------------------------------- 747
73.MN159912.1_Botrytiscinerea                                             569 TCGTTGAGGGTGTTCCAGGTGACAACGTCGG-TTTCAACG 607
74.MF034741.1_PeltasterfructicolaisolateSRB92                               0 ---------------------------------------- 0
75.LC440360.1_CercosporaasparagiCOasp2                                    224 CGCATCAAGAATAGACTGCTGACAATGGCTCCTCACAGGA 263
76.AY944105.1_MagnaportheoryzaeisolateSAG00T3()                           255 ---------------------------------------- 255
77.JX266586.1_CochliobolusmiyabeanusvoucherMFLUCC10-0733                  559 TGACCGAGGGTGTCCCCGGTGACAACGTCGG-TTTCAACG 597
78.MN393253.1_CorynesporacassiicolaisolateQHD001(MN393253.1UNVERIFIED)      0 ---------------------------------------- 0
79.MF375218.1_AgroatheliarolfsiiisolateBJB24                              544 ---------------------------------------- 544
80.MN106270.1_AgroatheliarolfsiistrainJ-12                                509 ---------------------------------------- 509
81.OQ732628.1_AgroatheliarolfsiiisolateBTCBSr3                            499 ---------------------------------------- 499
82.KY196185.1_ColletotrichumtruncatumstrainPAK53                          468 ---------------------------------------- 468
83.GU935835.1_ColletotrichumcoccodesisolateC96002                        1041 ---------------------------------------- 1041
84.MK085963.1_AlternariatenuissimaisolateSCCZ06                             0 ---------------------------------------- 0
85.MT548042.1_AlternarialongipesstrainKY_2019_012                           0 ---------------------------------------- 0
86.MN356465.1CalonectriamontanaisolateHSP4                                489 ---------------------------------------- 489
87.OL694224.1_CalonectriacanadianastrainF099                              504 ---------------------------------------- 504
88.MK803351.1_NeoscytalidiumdimidiatumstrainKale4-C                         0 ---------------------------------------- 0
89.ON376993.1_CurvulariachiangmaiensisisolateND00J7                       561 TCACCGAGGGTGTCCCCGGTGACAACGTCGG-CTTCAACG 599
90.OQ383346.1_NeoscytalidiumdimidiatumisolateGKH-2                          0 ---------------------------------------- 0
91.MF662595.1_NeoscytalidiumnovaehollandiaeisolateNeNo1                     0 ---------------------------------------- 0
92.EF560588.1Melampsoralini                                               656 ---------------------------------------- 656
93.LC590862.1_NeoscytalidiumdimidiatumPSU-HP01TEF1                          0 ---------------------------------------- 0
94.KX278106.1_BotryosphaeriaqingyuanensisstrainCERC2947                     0 ---------------------------------------- 0
95.AJ578763.1_Blumeriagraminisf.sp.hordeicyp51                              0 ---------------------------------------- 0
96.MF490858.1_CurvulariadactylocteniicolastrainCPC28810                   549 TCACCGAGGGTGTCCCCGGTGACAACGTCGG-CTTCAACG 587
97.KT287115.1_Bipolariscactivoraisolate3.8.6                              603 TCGTCGAGGGTGTCCCCGGTGACAACGTCGG-CTTCAACG 641
98.MT560940.1_CurvulariacactivorastrainHLGH0118                           560 TCGTCGAGGGTGTCCCCGGTGACAACGTCGG-CTTCAACG 598
99.OM714565.1_CurvulariaplantarumstrainM0134                              586 TTACTGAGGGTGTCCCCGGTGACAACGTCGG-CTTCAACG 624
100.MN159911.1_BotrytiscinereaSICAUCC19-0003                              569 TCGTTGAGGGTGTTCCAGGTGACAACGTCGG-TTTCAACG 607
```

```
102.GU294713.1Lasiodiplodiatheobromaestrain UCD2430TX                       0 ---------------------------------------- 0
```

```
102.GU294713.1LasiodiplodiatheobromaestrainUCD2430TX                           0 ---------------------------------------- 0
103.KX868094.1_Mycosphaerellasp.isolateCRM20.1                               475 ---------------------------------------- 475
104.LC599478.1Pseudocercosporapini-densifloraeMUCC534                          0 ---------------------------------------- 0
105.N584698.1BipolarissetariaestrainKBS4-2                                   614 TGACCGAGGGTGTCCCCGGTGACAACGTCGG-CTTCAACG 652

2.OM160859.1_F.buharicum                                                     560 ---------------------------------------- 560
1.LC727524.1_F.buharicum_OKI-1_Okura                                         682 ---------------------------------------- 682
3.KX302919.1_F.sublunatum                                                    521 ---------------------------------------- 521
4.LT996094.1_F.convolutans                                                   516 ---------------------------------------- 516
5.OM160861.1_F.abutilonis                                                    630 ---------------------------------------- 630
6.OM160874.1_F.guadeloupense                                                 560 ---------------------------------------- 560
7.MH392475.1_F.graminearum                                                   430 ---------------------------------------- 430
8.MH582420.1_F.solani                                                        540 ---------------------------------------- 540
9.MAFF244605_F.oxysporum                                                     544 ---------------------------------------- 544
10.MAFF237278_F.contaminatum_Hylocereus                                      711 ---------------------------------------- 711
11.MAFF237649_F.concentricum__Riceroot                                       678 ---------------------------------------- 678
12.MAFF237650_F.concentricum__Wheat                                          679 ---------------------------------------- 679
13.MAFF239869_F.mangiferae__Ryukyupine                                       657 ---------------------------------------- 657
14.MAFF240460_F.fujikuroi_Passionfruit                                       661 ---------------------------------------- 661
15.MAFF241317_F.graminearum_Wheat                                            666 ---------------------------------------- 666
16.MAFF242670_F.ipomoeae_Wheat                                               672 ---------------------------------------- 672
17.MAFF245129_F.concentricum_Fraxinus                                        659 ---------------------------------------- 659
18.MAFF245395_F.cugenangense_Rhubarb                                         671 ---------------------------------------- 671
19.MAFF246637_F.nirenbergiae_Strawberry                                      671 ---------------------------------------- 671
20.MAFF246672_F.nirenbergiae_ChinesePeony                                    671 ---------------------------------------- 671
21.MAFF246697_F.commune_Urallicoriceroot                                     673 ---------------------------------------- 673
22.MAFF246729_F.falciforme_Angelica                                          691 ---------------------------------------- 691
23.MAFF247220_F.duplospermum__Euwallaceasp                                   694 ---------------------------------------- 694
24.MAFF410760_F.odoratissimum_alpha                                          669 ---------------------------------------- 669
25.MAFF244605_FusariumoxysporumSchlechtendal_MAFF244605_Tomato               672 ---------------------------------------- 672
26.MAFF241326_F.asiaticum_Wheat                                              669 ---------------------------------------- 669
27.MAFF245014_F.asiaticum_Wildsoybean                                        666 ---------------------------------------- 666
28.MAFF150124_F.asiaticum__Wheat                                             666 ---------------------------------------- 666
29.OM135603.1F.algeriense                                                    699 ---------------------------------------- 699
30.MAFF237465_F.penzigii_Aloe                                                704 ---------------------------------------- 704
31.MAFF103054_F.oxysporumSchlechtendalf.sp.cucumerinum_Cucumber              671 ---------------------------------------- 671
32.MAFF712246_F.oxysporumSchlechtendalf.sp.dianthi__Carnation                673 ---------------------------------------- 673
33.MAFF305558_F.oxysporumSchlechtendalf.sp.fragariae__Watermelon             671 ---------------------------------------- 671
34.MAFF744087_F.oxysporumSchlechtendalf.sp.lactucae__Lettuce                 671 ---------------------------------------- 671
35.MAFF726924_F.oxysporumSchlechtendalf.sp.lagenariae_Whitefloweredgourd     671 ---------------------------------------- 671
36.MAFF744003_F.oxysporumSchlechtendalf.sp.lagenariae_Squash)                672 ---------------------------------------- 672
37.MAFF305122_F.oxysporumSchlechtendalf.sp.melonis__Melon                    671 ---------------------------------------- 671
38.MAFF306714_F.oxysporumSchlechtendalf.sp.momordicae_Balsampear             671 ---------------------------------------- 671
39.MAFF238905_F.oxysporumSchlechtendalf.sp.radicis-lycopersici_Tomato        674 ---------------------------------------- 674
40.MAFF150004_F.oxysporumSchlechtendalf.sp.spinaciae_Spinach                 671 ---------------------------------------- 671
41.MAFF247034_F.oxysporumSchlechtendal__Goldenchain                          671 ---------------------------------------- 671
```

```
42.MAFF245747_F.oxysporumSchlechtendalf.sp.callistephi__Chinaaster          671 ---------------------------------------- 671
43.MAFF305115_FoxysporumSchlechtendalf.sp.batatas__Sweatpotato              703 ---------------------------------------- 703
44.MAFF150126_F.asiaticum_Seed                                              666 ---------------------------------------- 666
45.MAFF246738_F.solani_Angelica                                             703 ---------------------------------------- 703
46.MAFF246664_F.cugenangense_Perilla                                        671 ---------------------------------------- 671
47.MH582420.1F.solanistrainMRC256                                           679 ---------------------------------------- 679
48.MAFF240361_F.babinda_Soil                                                693 ---------------------------------------- 693
49.MAFF242368_F.azukicola_Azukibean                                         708 ---------------------------------------- 708
50.MAFF241312_F.asiaticum_Soil,welshonionfield                              666 ---------------------------------------- 666
51.LT548416.1_F.culmorumpartialtef1a                                        646 ---------------------------------------- 646
52.MAFF150124_F.asiaticum__Wheat                                            666 ---------------------------------------- 666
53.MAFF238806_F.begoniae_Oncidiumsp                                         705 ---------------------------------------- 705
54.MW594399.1_FusariumincarnatumisolateUD01C                                689 ---------------------------------------- 689
55.OP414923.1Pucciniagraminisf.sp.triticiisolateSHZPgt19                    623 ---------------------------------------- 623
56.MT027094.1_BipolarisoryzaestrainOrL-2                                    589 TCAAGAACGTCTCCGTCAAGGAGATCCGTCGTGGCAACGT 628
57.ON734360.1_AlternariaalternataisolateH126                                464 TCAAGAACGTCTCCGTCAAGGAGATCCGTCGTGGTAACGT 503
58.LC333578.1_StemphyliumlycopersiciSOasp2                                    0 ---------------------------------------- 0
59.HQ718583.1_Colletotrichumgloeosporioidesisolateq-1                       567 ---------------------------------------- 567
60.JN241603.1_AtheliarolfsiiisolateSR1                                      977 TCAAGTAAGTCAAGTTGATACATCATTTATTTAACAT--- 1013
61.KJ866474.1_RhizoctoniasolanistrainMHL-1                                  683 ---------------------------------------- 683
62.JQ672424.1AlternariatriticinaisolateEGS17-061                            825 ---------------------------------------- 825
63.LT707559.1_P.capsicipartialtef1agene                                     689 TCAAGAACGTGTCGGTCAAGGAGCTGCGTCGTGGTTTCGT 728
64.MW090051.1_CurvularialunatastrainCls-3                                   583 TCAAGAACGTCTCCGTCAAGGAGATCCGTCGTGGTAACGT 622
65.DQ400892.1_Aspergillusterreus                                            452 ---------------------------------------- 452
66.DQ911416.1_Pythiumsp.quercumstrainPy292                                  668 TGAAGAACGTGTCGGTGAAGGAGCTGCGTCGCGGCTTCGT 707
67.EU797495.1_Phytophthorasp.oaksoilPoland                                  668 TCAAGAACGTGTCGGTCAAGGAGCTGCGTCGTGGATTCGT 707
68.HM148321.1_Cladosporiumcucumerinum                                       385 ---------------------------------------- 385
69.AF398888.1_SclerotiniasclerotiorumisolateSS1                               0 ---------------------------------------- 0
70.AF398888.1_S.sclerotiorumisolateSS1                                        0 ---------------------------------------- 0
71.HPAB545908.1_Verticilliumnonalfalfaeisolate                              442 ---------------------------------------- 442
72.EF433315.1_CeratocystisfimbriatavoucherCMW15052                          747 ---------------------------------------- 747
73.MN159912.1_Botrytiscinerea                                               608 TCAAGAACGTTTCCGTTAAGGAAATTAGACGTGGTAACGT 647
74.MF034741.1_PeltasterfructicolaisolateSRB92                                 0 ---------------------------------------- 0
75.LC440360.1_CercosporaasparagiCOasp2                                      264 AGCAGCTGAACTCGGTAAGGGTTCCTTCAAGTAA------ 297
76.AY944105.1_MagnaportheoryzaeisolateSAG00T3()                             255 ---------------------------------------- 255
77.JX266586.1_CochliobolusmiyabeanusvoucherMFLUCC10-0733                    598 TCAAGAACGTCTCCGTCAAGGAGATCCGTCGTGGCAACGT 637
78.MN393253.1_CorynesporacassiicolaisolateQHD001(MN393253.1UNVERIFIED)        0 ---------------------------------------- 0
79.MF375218.1_AgroatheliarolfsiiisolateBJB24                                544 ---------------------------------------- 544
80.MN106270.1_AgroatheliarolfsiistrainJ-12                                  509 ---------------------------------------- 509
81.OQ732628.1_AgroatheliarolfsiiisolateBTCBSr3                              499 ---------------------------------------- 499
82.KY196185.1_ColletotrichumtruncatumstrainPAK53                            468 ---------------------------------------- 468
83.GU935835.1_ColletotrichumcoccodesisolateC96002                          1041 ---------------------------------------- 1041
84.MK085963.1_AlternariatenuissimaisolateSCCZ06                               0 ---------------------------------------- 0
85.MT548042.1_AlternarialongipesstrainKY_2019_012                             0 ---------------------------------------- 0
86.MN356465.1CalonectriamontanaisolateHSP4                                  489 ---------------------------------------- 489
87.OL694224.1_CalonectriacanadianastrainF099                                504 ---------------------------------------- 504
```

```
88.MK803351.1_NeoscytalidiumdimidiatumstrainKale4-C              0 ---------------------------------------- 0
89.ON376993.1_CurvulariachiangmaiensisisolateND00J7            600 TCAAGAACGTCTCCGTCAAGGAGATCCGTCGTGGTAACGT 639
90.OQ383346.1_NeoscytalidiumdimidiatumisolateGKH-2               0 ---------------------------------------- 0
91.MF662595.1_NeoscytalidiumnovaehollandiaeisolateNeNo1          0 ---------------------------------------- 0
92.EF560588.1Melampsoralini                                    656 ---------------------------------------- 656
93.LC590862.1_NeoscytalidiumdimidiatumPSU-HP01TEF1               0 ---------------------------------------- 0
94.KX278106.1_BotryosphaeriaqingyuanensisstrainCERC2947          0 ---------------------------------------- 0
95.AJ578763.1_Blumeriagraminisf.sp.hordeicyp51                   0 ---------------------------------------- 0
96.MF490858.1_CurvulariadactylocteniicolastrainCPC28810        588 TCAAGAACGTCTCCGTCAAGGAGATCCGTCGTGGTAACGT 627
97.KT287115.1_Bipolariscactivoraisolate3.8.6                   642 TCAAGAACGTCTCCGTCAAGGAGATCCGCCGTGGTAACGT 681
98.MT560940.1_CurvulariacactivorastrainHLGH0118                599 TCAAGAACGTCTCCGTCAAGGAGATCCGCCGTGGTAACGT 638
99.OM714565.1_CurvulariaplantarumstrainM0134                   625 TCAAGAACGTCTCCGTCAAGGAGATCCGTCGTGGTAACGT 664
100.MN159911.1_BotrytiscinereaSICAUCC19-0003                   608 TCAAGAACGTTTCCGTTAAGGAAATTAGACGTGGTAACGT 647
102.GU294713.1LasiodiplodiatheobromaestrainUCD2430TX             0 ---------------------------------------- 0
103.KX868094.1_Mycosphaerellasp.isolateCRM20.1                 475 ---------------------------------------- 475
104.LC599478.1Pseudocercosporapini-densifloraeMUCC534            0 ---------------------------------------- 0
105.N584698.1BipolarissetariaestrainKBS4-2                     653 TCAAGAACGTCTCCGTCAAGGAGATCCGTCGTGGTAACGT 692

2.OM160859.1_F.buharicum                                       560 ---------------------------------------- 560
1.LC727524.1_F.buharicum_OKI-1_Okura                           682 ---------------------------------------- 682
3.KX302919.1_F.sublunatum                                      521 ---------------------------------------- 521
4.LT996094.1_F.convolutans                                     516 ---------------------------------------- 516
5.OM160861.1_F.abutilonis                                      630 ---------------------------------------- 630
6.OM160874.1_F.guadeloupense                                   560 ---------------------------------------- 560
7.MH392475.1_F.graminearum                                     430 ---------------------------------------- 430
8.MH582420.1_F.solani                                          540 ---------------------------------------- 540
9.MAFF244605_F.oxysporum                                       544 ---------------------------------------- 544
10.MAFF237278_F.contaminatum_Hylocereus                        711 ---------------------------------------- 711
11.MAFF237649_F.concentricum__Riceroot                         678 ---------------------------------------- 678
12.MAFF237650_F.concentricum__Wheat                            679 ---------------------------------------- 679
13.MAFF239869_F.mangiferae__Ryukyupine                         657 ---------------------------------------- 657
14.MAFF240460_F.fujikuroi_Passionfruit                         661 ---------------------------------------- 661
15.MAFF241317_F.graminearum_Wheat                              666 ---------------------------------------- 666
16.MAFF242670_F.ipomoeae_Wheat                                 672 ---------------------------------------- 672
17.MAFF245129_F.concentricum_Fraxinus                          659 ---------------------------------------- 659
18.MAFF245395_F.cugenangense_Rhubarb                           671 ---------------------------------------- 671
19.MAFF246637_F.nirenbergiae_Strawberry                        671 ---------------------------------------- 671
20.MAFF246672_F.nirenbergiae_ChinesePeony                      671 ---------------------------------------- 671
21.MAFF246697_F.commune_Urallicoriceroot                       673 ---------------------------------------- 673
22.MAFF246729_F.falciforme_Angelica                            691 ---------------------------------------- 691
23.MAFF247220_F.duplospermum__Euwallaceasp                     694 ---------------------------------------- 694
24.MAFF410760_F.odoratissimum_alpha                            669 ---------------------------------------- 669
25.MAFF244605_FusariumoxysporumSchlechtendal_MAFF244605_Tomato 672 ---------------------------------------- 672
26.MAFF241326_F.asiaticum_Wheat                                669 ---------------------------------------- 669
27.MAFF245014_F.asiaticum_Wildsoybean                          666 ---------------------------------------- 666
28.MAFF150124_F.asiaticum__Wheat                               666 ---------------------------------------- 666
```

```
29.OM135603.1F.algeriense                                     699 ---------------------------------------- 699
30.MAFF237465_F.penzigii_Aloe                                 704 ---------------------------------------- 704
31.MAFF103054_F.oxysporumSchlechtendalf.sp.cucumerinum_Cucumber 671 ---------------------------------------- 671
32.MAFF712246_F.oxysporumSchlechtendalf.sp.dianthi__Carnation 673 ---------------------------------------- 673
33.MAFF305558_F.oxysporumSchlechtendalf.sp.fragariae__Watermelon 671 ---------------------------------------- 671
34.MAFF744087_F.oxysporumSchlechtendalf.sp.lactucae__Lettuce  671 ---------------------------------------- 671
35.MAFF726924_F.oxysporumSchlechtendalf.sp.lagenariae_WhiteflOweredgourd 671 ---------------------------------------- 671
36.MAFF744003_F.oxysporumSchlechtendalf.sp.lagenariae_Squash) 672 ---------------------------------------- 672
37.MAFF305122_F.oxysporumSchlechtendalf.sp.melonis__Melon     671 ---------------------------------------- 671
38.MAFF306714_F.oxysporumSchlechtendalf.sp.momordicae_Balsampear 671 ---------------------------------------- 671
39.MAFF238905_F.oxysporumSchlechtendalf.sp.radicis-lycopersici_Tomato 674 ---------------------------------------- 674
40.MAFF150004_F.oxysporumSchlechtendalf.sp.spinaciae_Spinach  671 ---------------------------------------- 671
41.MAFF247034_F.oxysporumSchlechtendal__Goldenchain           671 ---------------------------------------- 671
42.MAFF245747_F.oxysporumSchlechtendalf.sp.callistephi__Chinaaster 671 ---------------------------------------- 671
43.MAFF305115_FoxysporumSchlechtendalf.sp.batatas__Sweatpotato 703 ---------------------------------------- 703
44.MAFF150126_F.asiaticum_Seed                                666 ---------------------------------------- 666
45.MAFF246738_F.solani_Angelica                               703 ---------------------------------------- 703
46.MAFF246664_F.cugenangense_Perilla                          671 ---------------------------------------- 671
47.MH582420.1F.solanistrainMRC256                             679 ---------------------------------------- 679
48.MAFF240361_F.babinda_Soil                                  693 ---------------------------------------- 693
49.MAFF242368_F.azukicola_Azukibean                           708 ---------------------------------------- 708
50.MAFF241312_F.asiaticum_Soil,welshonionfield                666 ---------------------------------------- 666
51.LT548416.1_F.culmorumpartialtef1a                          646 ---------------------------------------- 646
52.MAFF150124_F.asiaticum__Wheat                              666 ---------------------------------------- 666
53.MAFF238806_F.begoniae_Oncidiumsp                           705 ---------------------------------------- 705
54.MW594399.1_FusariumincarnatumisolateUD01C                  689 ---------------------------------------- 689
55.OP414923.1Pucciniagraminisf.sp.triticiisolateSHZPgt19      623 ---------------------------------------- 623
56.MT027094.1_BipolarisoryzaestrainOrL-2                      629 TGCCGGTGACTCCAAGAACGACCCCCCCAAGGCTTCCGAG 668
57.ON734360.1_AlternariaalternataisolateH126                  504 TGCCGGTGACTCCAAGAACGACCCCCCCAAGGGTGCCGAG 543
58.LC333578.1_StemphyliumlycopersiciSOasp2                      0 ---------------------------------------- 0
59.HQ718583.1_Colletotrichumgloeosporioidesisolateq-1         567 ---------------------------------------- 567
60.JN241603.1_AtheliarolfsiiisolateSR1                       1013 ---------------------------------------- 1013
61.KJ866474.1_RhizoctoniasolanistrainMHL-1                    683 ---------------------------------------- 683
62.JQ672424.1AlternariatriticinaisolateEGS17-061              825 ---------------------------------------- 825
63.LT707559.1_P.capsicipartialtef1agene                       729 CGCTTCGGACTCCAAGAACGACCCCGCTAAGGGCACCCAG 768
64.MW090051.1_CurvularialunatastrainCls-3                     623 TGCCGGTGACTCCAAGAACGACCCCCCCAAGGGTTGCGAG 662
65.DQ400892.1_Aspergillusterreus                              452 ---------------------------------------- 452
66.DQ911416.1_Pythiumsp.quercumstrainPy292                    708 GGCTTCGGACTCGAAGAACGACCCGGCGAAGGCTACGGAG 747
67.EU797495.1_Phytophthorasp.oaksoilPoland                    708 CGCCTCGGACTCCAAGAACGACCCGGCCAAGGCCACCCAG 747
68.HM148321.1_Cladosporiumcucumerinum                         385 ---------------------------------------- 385
69.AF398888.1_SclerotiniasclerotiorumisolateSS1                 0 ---------------------------------------- 0
70.AF398888.1_S.sclerotiorumisolateSS1                          0 ---------------------------------------- 0
71.HPAB545908.1_Verticilliumnonalfalfaeisolate                442 ---------------------------------------- 442
72.EF433315.1_CeratocystisfimbriatavoucherCMW15052            747 ---------------------------------------- 747
73.MN159912.1_Botrytiscinerea                                 648 TGCTGGTGACTCCAAGCAAGACCCCCCCAAGGGTGCCGAG 687
74.MF034741.1_PeltasterfructicolaisolateSRB92                   0 ---------------------------------------- 0
```

```
75.LC440360.1_CercosporaasparagiCOasp2                                  297 ---------------------------------------- 297
76.AY944105.1_MagnaportheoryzaeisolateSAG00T3()                         255 ---------------------------------------- 255
77.JX266586.1_CochliobolusmiyabeanusvoucherMFLUCC10-0733                638 TGCCGGTGACTCCAAGAACGACCCCCCCAAGGCTTCCGAG 677
78.MN393253.1_CorynesporacassiicolaisolateQHD001(MN393253.1UNVERIFIED)    0 ---------------------------------------- 0
79.MF375218.1_AgroatheliarolfsiiisolateBJB24                            544 ---------------------------------------- 544
80.MN106270.1_AgroatheliarolfsiistrainJ-12                              509 ---------------------------------------- 509
81.OQ732628.1_AgroatheliarolfsiiisolateBTCBSr3                          499 ---------------------------------------- 499
82.KY196185.1_ColletotrichumtruncatumstrainPAK53                        468 ---------------------------------------- 468
83.GU935835.1_ColletotrichumcoccodesisolateC96002                      1041 ---------------------------------------- 1041
84.MK085963.1_AlternariatenuissimaisolateSCCZ06                           0 ---------------------------------------- 0
85.MT548042.1_AlternarialongipesstrainKY_2019_012                         0 ---------------------------------------- 0
86.MN356465.1CalonectriamontanaisolateHSP4                              489 ---------------------------------------- 489
87.OL694224.1_CalonectriacanadianastrainF099                            504 ---------------------------------------- 504
88.MK803351.1_NeoscytalidiumdimidiatumstrainKale4-C                       0 ---------------------------------------- 0
89.ON376993.1_CurvulariachiangmaiensisisolateND00J7                     640 TGCCGGTGACTCCAAGAACGACCCCCCCAAGGGTTGCGAG 679
90.OQ383346.1_NeoscytalidiumdimidiatumisolateGKH-2                        0 ---------------------------------------- 0
91.MF662595.1_NeoscytalidiumnovaehollandiaeisolateNeNo1                   0 ---------------------------------------- 0
92.EF560588.1Melampsoralini                                             656 ---------------------------------------- 656
93.LC590862.1_NeoscytalidiumdimidiatumPSU-HP01TEF1                        0 ---------------------------------------- 0
94.KX278106.1_BotryosphaeriaqingyuanensisstrainCERC2947                   0 ---------------------------------------- 0
95.AJ578763.1_Blumeriagraminisf.sp.hordeicyp51                            1 -----------------------ATGGGAATATCAGAAAG 17
96.MF490858.1_CurvulariadactylocteniicolastrainCPC28810                 628 TGCCGGTGACTCCAAGAACGACCCCCCCAAGGGTTGCGAG 667
97.KT287115.1_Bipolariscactivoraisolate3.8.6                            682 TGCCGGTGACTCGAAGAACGACCCTCCCAAGGGCTGCGAG 721
98.MT560940.1_CurvulariacactivorastrainHLGH0118                         639 TGCCGGTGACTCGAAGAACGACCCTCCCAAGGGCTGCGAG 678
99.OM714565.1_CurvulariaplantarumstrainM0134                            665 TGCCGGTGACTCCAAGAACGACCCCCCCAAGGGTTGCGAG 704
100.MN159911.1_BotrytiscinereaSICAUCC19-0003                            648 TGCTGGTGACTCCAAGCAAGACCCCCCCAAGGGTGCCGAG 687
102.GU294713.1LasiodiplodiatheobromaestrainUCD2430TX                      1 ----------------------TATGCACACAATTTCCCC 18
103.KX868094.1_Mycosphaerellasp.isolateCRM20.1                          475 ---------------------------------------- 475
104.LC599478.1Pseudocercosporapini-densifloraeMUCC534                     0 ---------------------------------------- 0
105.N584698.1BipolarissetariaestrainKBS4-2                              693 TGCTGGTGACTCCAAGAACGACCCCCCCAAGGCTTCCGAG 732

2.OM160859.1_F.buharicum                                                560 ---------------------------------------- 560
1.LC727524.1_F.buharicum_OKI-1_Okura                                    682 ---------------------------------------- 682
3.KX302919.1_F.sublunatum                                               521 ---------------------------------------- 521
4.LT996094.1_F.convolutans                                              516 ---------------------------------------- 516
5.OM160861.1_F.abutilonis                                               630 ---------------------------------------- 630
6.OM160874.1_F.guadeloupense                                            560 ---------------------------------------- 560
7.MH392475.1_F.graminearum                                              430 ---------------------------------------- 430
8.MH582420.1_F.solani                                                   540 ---------------------------------------- 540
9.MAFF244605_F.oxysporum                                                544 ---------------------------------------- 544
10.MAFF237278_F.contaminatum_Hylocereus                                 711 ---------------------------------------- 711
11.MAFF237649_F.concentricum__Riceroot                                  678 ---------------------------------------- 678
12.MAFF237650_F.concentricum__Wheat                                     679 ---------------------------------------- 679
13.MAFF239869_F.mangiferae__Ryukyupine                                  657 ---------------------------------------- 657
14.MAFF240460_F.fujikuroi_Passionfruit                                  661 ---------------------------------------- 661
15.MAFF241317_F.graminearum_Wheat                                       666 ---------------------------------------- 666
```

```
16.MAFF242670_F.ipomoeae_Wheat                                        672 ---------------------------------------- 672
17.MAFF245129_F.concentricum_Fraxinus                                 659 ---------------------------------------- 659
18.MAFF245395_F.cugenangense_Rhubarb                                  671 ---------------------------------------- 671
19.MAFF246637_F.nirenbergiae_Strawberry                               671 ---------------------------------------- 671
20.MAFF246672_F.nirenbergiae_ChinesePeony                             671 ---------------------------------------- 671
21.MAFF246697_F.commune_Urallicoriceroot                              673 ---------------------------------------- 673
22.MAFF246729_F.falciforme_Angelica                                   691 ---------------------------------------- 691
23.MAFF247220_F.duplospermum__Euwallaceasp                            694 ---------------------------------------- 694
24.MAFF410760_F.odoratissimum_alpha                                   669 ---------------------------------------- 669
25.MAFF244605_FusariumoxysporumSchlechtendal_MAFF244605_Tomato        672 ---------------------------------------- 672
26.MAFF241326_F.asiaticum_Wheat                                       669 ---------------------------------------- 669
27.MAFF245014_F.asiaticum_Wildsoybean                                 666 ---------------------------------------- 666
28.MAFF150124_F.asiaticum__Wheat                                      666 ---------------------------------------- 666
29.OM135603.1F.algeriense                                             699 ---------------------------------------- 699
30.MAFF237465_F.penzigii_Aloe                                         704 ---------------------------------------- 704
31.MAFF103054_F.oxysporumSchlechtendalf.sp.cucumerinum_Cucumber       671 ---------------------------------------- 671
32.MAFF712246_F.oxysporumSchlechtendalf.sp.dianthi__Carnation         673 ---------------------------------------- 673
33.MAFF305558_F.oxysporumSchlechtendalf.sp.fragariae__Watermelon      671 ---------------------------------------- 671
34.MAFF744087_F.oxysporumSchlechtendalf.sp.lactucae__Lettuce          671 ---------------------------------------- 671
35.MAFF726924_F.oxysporumSchlechtendalf.sp.lagenariae_Whitefloweredgourd 671 ---------------------------------------- 671
36.MAFF744003_F.oxysporumSchlechtendalf.sp.lagenariae_Squash)         672 ---------------------------------------- 672
37.MAFF305122_F.oxysporumSchlechtendalf.sp.melonis__Melon             671 ---------------------------------------- 671
38.MAFF306714_F.oxysporumSchlechtendalf.sp.momordicae_Balsampear      671 ---------------------------------------- 671
39.MAFF238905_F.oxysporumSchlechtendalf.sp.radicis-lycopersici_Tomato 674 ---------------------------------------- 674
40.MAFF150004_F.oxysporumSchlechtendalf.sp.spinaciae_Spinach          671 ---------------------------------------- 671
41.MAFF247034_F.oxysporumSchlechtendal__Goldenchain                   671 ---------------------------------------- 671
42.MAFF245747_F.oxysporumSchlechtendalf.sp.callistephi__Chinaaster    671 ---------------------------------------- 671
43.MAFF305115_FoxysporumSchlechtendalf.sp.batatas__Sweatpotato        703 ---------------------------------------- 703
44.MAFF150126_F.asiaticum_Seed                                        666 ---------------------------------------- 666
45.MAFF246738_F.solani_Angelica                                       703 ---------------------------------------- 703
46.MAFF246664_F.cugenangense_Perilla                                  671 ---------------------------------------- 671
47.MH582420.1F.solanistrainMRC256                                     679 ---------------------------------------- 679
48.MAFF240361_F.babinda_Soil                                          693 ---------------------------------------- 693
49.MAFF242368_F.azukicola_Azukibean                                   708 ---------------------------------------- 708
50.MAFF241312_F.asiaticum_Soil,welshonionfield                        666 ---------------------------------------- 666
51.LT548416.1_F.culmorumpartialtef1a                                  646 ---------------------------------------- 646
52.MAFF150124_F.asiaticum__Wheat                                      666 ---------------------------------------- 666
53.MAFF238806_F.begoniae_Oncidiumsp                                   705 ---------------------------------------- 705
54.MW594399.1_FusariumincarnatumisolateUD01C                          689 ---------------------------------------- 689
55.OP414923.1Pucciniagraminisf.sp.triticiisolateSHZPgt19              623 ---------------------------------------- 623
56.MT027094.1_BipolarisoryzaestrainOrL-2                              669 TCCTTCAACGCCCAGGTCATCGTCCTCAACCACCCCGGTC 708
57.ON734360.1_AlternariaalternataisolateH126                          544 TCCTTCAACGCCCAGGTCATCGTCCTCAACCACCCTGGTC 583
58.LC333578.1_StemphyliumlycopersiciSOasp2                              0 ---------------------------------------- 0
59.HQ718583.1_Colletotrichumgloeosporioidesisolateq-1                 567 ---------------------------------------- 567
60.JN241603.1_AtheliarolfsiiisolateSR1                               1013 ---------------------------------------- 1013
61.KJ866474.1_RhizoctoniasolanistrainMHL-1                            683 ---------------------------------------- 683
```

```
62.JQ672424.1AlternariatriticinaisolateEGS17-061                       825 ---------------------------------------- 825
63.LT707559.1_P.capsicipartialtef1agene                                769 GACTTCACCGCTCAGGTGATTGTGCTGAACCACCCTGGCC 808
64.MW090051.1_CurvularialunatastrainCls-3                              663 TCCTTCAACGCCCAGGTCATCGTCCTCAACCACCCCGGTC 702
65.DQ400892.1_Aspergillusterreus                                       452 ---------------------------------------- 452
66.DQ911416.1_Pythiumsp.quercumstrainPy292                             748 GACTTCACGGCCCAGGTGATCGTGCTGAACCACCCCGGCC 787
67.EU797495.1_Phytophthorasp.oaksoilPoland                             748 GACTTCACCGCCCAGGTGATCGTGCTGAACCACCCCGGTC 787
68.HM148321.1_Cladosporiumcucumerinum                                  385 ---------------------------------------- 385
69.AF398888.1_SclerotiniasclerotiorumisolateSS1                          0 ---------------------------------------- 0
70.AF398888.1_S.sclerotiorumisolateSS1                                   0 ---------------------------------------- 0
71.HPAB545908.1_Verticilliumnonalfalfaeisolate                         442 ---------------------------------------- 442
72.EF433315.1_CeratocystisfimbriatavoucherCMW15052                     747 ---------------------------------------- 747
73.MN159912.1_Botrytiscinerea                                          688 TCCTTCAACGCTCAAGTCATTGTTCTTAACCACCCTGGTC 727
74.MF034741.1_PeltasterfructicolaisolateSRB92                            1 --------------------------TCATCGAGAAGTTC 14
75.LC440360.1_CercosporaasparagiCOasp2                                 297 ---------------------------------------- 297
76.AY944105.1_MagnaportheoryzaeisolateSAG00T3()                        255 ---------------------------------------- 255
77.JX266586.1_CochliobolusmiyabeanusvoucherMFLUCC10-0733               678 TCCTTCAACGCCCAGGTCATCGTCCTCAACCACCCCGGTC 717
78.MN393253.1_CorynesporacassiicolaisolateQHD001(MN393253.1UNVERIFIED)   0 ---------------------------------------- 0
79.MF375218.1_AgroatheliarolfsiiisolateBJB24                           544 ---------------------------------------- 544
80.MN106270.1_AgroatheliarolfsiistrainJ-12                             509 ---------------------------------------- 509
81.OQ732628.1_AgroatheliarolfsiiisolateBTCBSr3                         499 ---------------------------------------- 499
82.KY196185.1_ColletotrichumtruncatumstrainPAK53                       468 ---------------------------------------- 468
83.GU935835.1_ColletotrichumcoccodesisolateC96002                     1041 ---------------------------------------- 1041
84.MK085963.1_AlternariatenuissimaisolateSCCZ06                          0 ---------------------------------------- 0
85.MT548042.1_AlternarialongipesstrainKY_2019_012                        0 ---------------------------------------- 0
86.MN356465.1CalonectriamontanaisolateHSP4                             489 ---------------------------------------- 489
87.OL694224.1_CalonectriacanadianastrainF099                           504 ---------------------------------------- 504
88.MK803351.1_NeoscytalidiumdimidiatumstrainKale4-C                      1 --------------------------------------CA 2
89.ON376993.1_CurvulariachiangmaiensisisolateND00J7                    680 TCCTTCAACGCCCAGGTCATCGTCCTCAACCACCCCGGTC 719
90.OQ383346.1_NeoscytalidiumdimidiatumisolateGKH-2                       0 ---------------------------------------- 0
91.MF662595.1_NeoscytalidiumnovaehollandiaeisolateNeNo1                  0 ---------------------------------------- 0
92.EF560588.1Melampsoralini                                            656 ---------------------------------------- 656
93.LC590862.1_NeoscytalidiumdimidiatumPSU-HP01TEF1                       0 ---------------------------------------- 0
94.KX278106.1_BotryosphaeriaqingyuanensisstrainCERC2947                  0 ---------------------------------------- 0
95.AJ578763.1_Blumeriagraminisf.sp.hordeicyp51                          18 CTTTATGTTTCCATACTTGCAGCCTTTGCTCCAACTCGGT 57
96.MF490858.1_CurvulariadactylocteniicolastrainCPC28810                668 TCCTTCAACGCCCAGGTCATCGTCCTCAACCACCCCGGTC 707
97.KT287115.1_Bipolariscactivoraisolate3.8.6                           722 TCCTTCAACGCCCAGGTCATTGTCCTCAACCACCCTGGTC 761
98.MT560940.1_CurvulariacactivorastrainHLGH0118                        679 TCCTTCAACGCCCAGGTCATTGTCCTCAACCACCCTGGTC 718
99.OM714565.1_CurvulariaplantarumstrainM0134                           705 TCCTTCAACGCCCAGGTCATCGTCCTCAACCACCCTGGTC 744
100.MN159911.1_BotrytiscinereaSICAUCC19-0003                           688 TCCTTCAACGCTCAAGTCATTGTTCTTAACCACCCTGGTC 727
102.GU294713.1LasiodiplodiatheobromaestrainUCD2430TX                     19 ATTTGCGGCGCTGGCATTTGATTGCGCTCTCGCTGCGTTA 58
103.KX868094.1_Mycosphaerellasp.isolateCRM20.1                          475 ---------------------------------------- 475
104.LC599478.1Pseudocercosporapini-densifloraeMUCC534                    1 ---------------------------GAGAAGGTAAGCT 13
105.N584698.1BipolarissetariaestrainKBS4-2                             733 TCCTTCAACGCCCAGGTCATCGTCCTCAACCACCCCGGTC 772

2.OM160859.1_F.buharicum                                               560 ---------------------------------------- 560
1.LC727524.1_F.buharicum_OKI-1_Okura                                   682 ---------------------------------------- 682
```

3.KX302919.1_F.sublunatum	521	--	521
4.LT996094.1_F.convolutans	516	--	516
5.OM160861.1_F.abutilonis	630	--	630
6.OM160874.1_F.guadeloupense	560	--	560
7.MH392475.1_F.graminearum	430	--	430
8.MH582420.1_F.solani	540	--	540
9.MAFF244605_F.oxysporum	544	--	544
10.MAFF237278_F.contaminatum_Hylocereus	711	--	711
11.MAFF237649_F.concentricum__Riceroot	678	--	678
12.MAFF237650_F.concentricum__Wheat	679	--	679
13.MAFF239869_F.mangiferae__Ryukyupine	657	--	657
14.MAFF240460_F.fujikuroi_Passionfruit	661	--	661
15.MAFF241317_F.graminearum_Wheat	666	--	666
16.MAFF242670_F.ipomoeae_Wheat	672	--	672
17.MAFF245129_F.concentricum_Fraxinus	659	--	659
18.MAFF245395_F.cugenangense_Rhubarb	671	--	671
19.MAFF246637_F.nirenbergiae_Strawberry	671	--	671
20.MAFF246672_F.nirenbergiae_ChinesePeony	671	--	671
21.MAFF246697_F.commune_Urallicoriceroot	673	--	673
22.MAFF246729_F.falciforme_Angelica	691	--	691
23.MAFF247220_F.duplospermum__Euwallaceasp	694	--	694
24.MAFF410760_F.odoratissimum_alpha	669	--	669
25.MAFF244605_FusariumoxysporumSchlechtendal_MAFF244605_Tomato	672	--	672
26.MAFF241326_F.asiaticum_Wheat	669	--	669
27.MAFF245014_F.asiaticum_Wildsoybean	666	--	666
28.MAFF150124_F.asiaticum__Wheat	666	--	666
29.OM135603.1F.algeriense	699	--	699
30.MAFF237465_F.penzigii_Aloe	704	--	704
31.MAFF103054_F.oxysporumSchlechtendalf.sp.cucumerinum_Cucumber	671	--	671
32.MAFF712246_F.oxysporumSchlechtendalf.sp.dianthi__Carnation	673	--	673
33.MAFF305558_F.oxysporumSchlechtendalf.sp.fragariae__Watermelon	671	--	671
34.MAFF744087_F.oxysporumSchlechtendalf.sp.lactucae__Lettuce	671	--	671
35.MAFF726924_F.oxysporumSchlechtendalf.sp.lagenariae_Whitefloweredgourd	671	--	671
36.MAFF744003_F.oxysporumSchlechtendalf.sp.lagenariae_Squash)	672	--	672
37.MAFF305122_F.oxysporumSchlechtendalf.sp.melonis__Melon	671	--	671
38.MAFF306714_F.oxysporumSchlechtendalf.sp.momordicae_Balsampear	671	--	671
39.MAFF238905_F.oxysporumSchlechtendalf.sp.radicis-lycopersici_Tomato	674	--	674
40.MAFF150004_F.oxysporumSchlechtendalf.sp.spinaciae_Spinach	671	--	671
41.MAFF247034_F.oxysporumSchlechtendal__Goldenchain	671	--	671
42.MAFF245747_F.oxysporumSchlechtendalf.sp.callistephi__Chinaaster	671	--	671
43.MAFF305115_FoxysporumSchlechtendalf.sp.batatas__Sweatpotato	703	--	703
44.MAFF150126_F.asiaticum_Seed	666	--	666
45.MAFF246738_F.solani_Angelica	703	--	703
46.MAFF246664_F.cugenangense_Perilla	671	--	671
47.MH582420.1F.solanistrainMRC256	679	--	679
48.MAFF240361_F.babinda_Soil	693	--	693

```
49.MAFF242368_F.azukicola_Azukibean                                     708 ---------------------------------------- 708
50.MAFF241312_F.asiaticum_Soil,welshonionfield                          666 ---------------------------------------- 666
51.LT548416.1_F.culmorumpartialtef1a                                    646 ---------------------------------------- 646
52.MAFF150124_F.asiaticum__Wheat                                        666 ---------------------------------------- 666
53.MAFF238806_F.begoniae_Oncidiumsp                                     705 ---------------------------------------- 705
54.MW594399.1_FusariumincarnatumisolateUD01C                            689 ---------------------------------------- 689
55.OP414923.1Pucciniagraminisf.sp.triticiisolateSHZPgt19                623 ---------------------------------------- 623
56.MT027094.1_BipolarisoryzaestrainOrL-2                                709 AGGTCGGTGCCGGTTACGCACCAGTC-CTCGACTGCCACA 747
57.ON734360.1_AlternariaalternataisolateH126                            584 AGGTCGGTGCTGGTTACGCCCCAGTC-CTCGACTGCCACA 622
58.LC333578.1_StemphyliumlycopersiciSOasp2                                0 ---------------------------------------- 0
59.HQ718583.1_Colletotrichumgloeosporioidesisolateq-1                   567 ---------------------------------------- 567
60.JN241603.1_AtheliarolfsiiisolateSR1                                 1013 ---------------------------------------- 1013
61.KJ866474.1_RhizoctoniasolanistrainMHL-1                              683 ---------------------------------------- 683
62.JQ672424.1AlternariatriticinaisolateEGS17-061                        825 ---------------------------------------- 825
63.LT707559.1_P.capsicipartialtef1agene                                 809 AGATCGGCAACGGTTACTCGCCGGTG-CTTGATTGCCACA 847
64.MW090051.1_CurvularialunatastrainCls-3                               703 AGGTCGGTGCCGGTTACGCACCAGTC-CTTGACTGCCACA 741
65.DQ400892.1_Aspergillusterreus                                        452 ---------------------------------------- 452
66.DQ911416.1_Pythiumsp.quercumstrainPy292                              788 AGATCGGCAACGGCTACTCGCCGGTG-CTTGACTGCCACA 826
67.EU797495.1_Phytophthorasp.oaksoilPoland                              788 AGATCGGCAACGGCTACTCGCCGGTG-CTCGACTGCCACA 826
68.HM148321.1_Cladosporiumcucumerinum                                   385 ---------------------------------------- 385
69.AF398888.1_SclerotiniasclerotiorumisolateSS1                           0 ---------------------------------------- 0
70.AF398888.1_S.sclerotiorumisolateSS1                                    0 ---------------------------------------- 0
71.HPAB545908.1_Verticilliumnonalfalfaeisolate                          442 ---------------------------------------- 442
72.EF433315.1_CeratocystisfimbriatavoucherCMW15052                      747 ---------------------------------------- 747
73.MN159912.1_Botrytiscinerea                                           728 AAGTCGGTGCTGGTTACGCTCCAGTT-TTGGATTGCCACA 766
74.MF034741.1_PeltasterfructicolaisolateSRB92                            15 GAGAAGGTAAGACTCTTCCACAAACATTCCTAGCACCGCT 54
75.LC440360.1_CercosporaasparagiCOasp2                                  297 ---------------------------------------- 297
76.AY944105.1_MagnaportheoryzaeisolateSAG00T3()                         255 ---------------------------------------- 255
77.JX266586.1_CochliobolusmiyabeanusvoucherMFLUCC10-0733                718 AGGTCGGTGCCGGTTACGCACCAGTC-CTCGACTGCCACA 756
78.MN393253.1_CorynesporacassiicolaisolateQHD001(MN393253.1UNVERIFIED)    0 ---------------------------------------- 0
79.MF375218.1_AgroatheliarolfsiiisolateBJB24                            544 ---------------------------------------- 544
80.MN106270.1_AgroatheliarolfsiistrainJ-12                              509 ---------------------------------------- 509
81.OQ732628.1_AgroatheliarolfsiiisolateBTCBSr3                          499 ---------------------------------------- 499
82.KY196185.1_ColletotrichumtruncatumstrainPAK53                        468 ---------------------------------------- 468
83.GU935835.1_ColletotrichumcoccodesisolateC96002                      1041 ---------------------------------------- 1041
84.MK085963.1_AlternariatenuissimaisolateSCCZ06                           0 ---------------------------------------- 0
85.MT548042.1_AlternarialongipesstrainKY_2019_012                         0 ---------------------------------------- 0
86.MN356465.1CalonectriamontanaisolateHSP4                              489 ---------------------------------------- 489
87.OL694224.1_CalonectriacanadianastrainF099                            504 ---------------------------------------- 504
88.MK803351.1_NeoscytalidiumdimidiatumstrainKale4-C                       3 TCGAGAAGTTCGAGAAGGTAAGCAAACTTTTTTCCGCCAC 42
89.ON376993.1_CurvulariachiangmaiensisisolateND00J7                     720 AGGTCGGTGCCGGTTACGCACCAGTC-CTTGACTGCCACA 758
90.OQ383346.1_NeoscytalidiumdimidiatumisolateGKH-2                        1 -----------------------GCCGGCTATCAGAGCAC 17
91.MF662595.1_NeoscytalidiumnovaehollandiaeisolateNeNo1                   1 ----GAAGTTCGAGAAGGTAAGCAAACTTTTTTCCGCCAC 36
92.EF560588.1Melampsoralini                                             656 ---------------------------------------- 656
93.LC590862.1_NeoscytalidiumdimidiatumPSU-HP01TEF1                        1 ------------------------------AATCTTCGCA 10
94.KX278106.1_BotryosphaeriaqingyuanensisstrainCERC2947                   1 --GAGAAGTTCGAGAAGGTAAGCACGCTTT-TTCTATG-- 35
```

```
95.AJ578763.1_Blumeriagraminisf.sp.hordeicyp51                  58 TTTGGCATTGCGTTGGCTAGTGGAATTATAAGTTTATTAT 97
96.MF490858.1_CurvulariadactylocteniicolastrainCPC28810        708 AGGTCGGTGCCGGTTACGCACCAGTC-CTTGACTGCCACA 746
97.KT287115.1_Bipolariscactivoraisolate3.8.6                   762 AGGTCGGTGCCGGTTACGCGCCAGTC-CTCGACTGCCACA 800
98.MT560940.1_CurvulariacactivorastrainHLGH0118                719 AGGTCGGTGCCGGTTACGCGCCAGTC-CTCGACTGCCACA 757
99.OM714565.1_CurvulariaplantarumstrainM0134                   745 AGGTCGGTGCCGGTTACGCCCCAGTC-CTTGACTGCCACA 783
100.MN159911.1_BotrytiscinereaSICAUCC19-0003                   728 AAGTCGGTGCTGGTTACGCTCCAGTT-TTGGATTGCCACA 766
102.GU294713.1LasiodiplodiatheobromaestrainUCD2430TX             59 TCGAGAAGTTCGAGAAGGGA-------GTGATTCC-CGAC 90
103.KX868094.1_Mycosphaerellasp.isolateCRM20.1                 475 ---------------------------------------- 475
104.LC599478.1Pseudocercosporapini-densifloraeMUCC534           14 ATCGCCATCACTTTCTCGCACCCGCCGCTCGACTACAACA 53
105.N584698.1BipolarissetariaestrainKBS4-2                     773 AGGTCGGTGCCGGTTACGCACCAGTC-CTCGACTGCCACA 811

2.OM160859.1_F.buharicum                                       560 ---------------------------------------- 560
1.LC727524.1_F.buharicum_OKI-1_Okura                           682 ---------------------------------------- 682
3.KX302919.1_F.sublunatum                                      521 ---------------------------------------- 521
4.LT996094.1_F.convolutans                                     516 ---------------------------------------- 516
5.OM160861.1_F.abutilonis                                      630 ---------------------------------------- 630
6.OM160874.1_F.guadeloupense                                   560 ---------------------------------------- 560
7.MH392475.1_F.graminearum                                     430 ---------------------------------------- 430
8.MH582420.1_F.solani                                          540 ---------------------------------------- 540
9.MAFF244605_F.oxysporum                                       544 ---------------------------------------- 544
10.MAFF237278_F.contaminatum_Hylocereus                        711 ---------------------------------------- 711
11.MAFF237649_F.concentricum__Riceroot                         678 ---------------------------------------- 678
12.MAFF237650_F.concentricum__Wheat                            679 ---------------------------------------- 679
13.MAFF239869_F.mangiferae__Ryukyupine                         657 ---------------------------------------- 657
14.MAFF240460_F.fujikuroi_Passionfruit                         661 ---------------------------------------- 661
15.MAFF241317_F.graminearum_Wheat                              666 ---------------------------------------- 666
16.MAFF242670_F.ipomoeae_Wheat                                 672 ---------------------------------------- 672
17.MAFF245129_F.concentricum_Fraxinus                          659 ---------------------------------------- 659
18.MAFF245395_F.cugenangense_Rhubarb                           671 ---------------------------------------- 671
19.MAFF246637_F.nirenbergiae_Strawberry                        671 ---------------------------------------- 671
20.MAFF246672_F.nirenbergiae_ChinesePeony                      671 ---------------------------------------- 671
21.MAFF246697_F.commune_Urallicoriceroot                       673 ---------------------------------------- 673
22.MAFF246729_F.falciforme_Angelica                            691 ---------------------------------------- 691
23.MAFF247220_F.duplospermum__Euwallaceasp                     694 ---------------------------------------- 694
24.MAFF410760_F.odoratissimum_alpha                            669 ---------------------------------------- 669
25.MAFF244605_FusariumoxysporumSchlechtendal_MAFF244605_Tomato 672 ---------------------------------------- 672
26.MAFF241326_F.asiaticum_Wheat                                669 ---------------------------------------- 669
27.MAFF245014_F.asiaticum_Wildsoybean                          666 ---------------------------------------- 666
28.MAFF150124_F.asiaticum__Wheat                               666 ---------------------------------------- 666
29.OM135603.1F.algeriense                                      699 ---------------------------------------- 699
30.MAFF237465_F.penzigii_Aloe                                  704 ---------------------------------------- 704
31.MAFF103054_F.oxysporumSchlechtendalf.sp.cucumerinum_Cucumber 671 ---------------------------------------- 671
32.MAFF712246_F.oxysporumSchlechtendalf.sp.dianthi__Carnation  673 ---------------------------------------- 673
33.MAFF305558_F.oxysporumSchlechtendalf.sp.fragariae__Watermelon 671 ---------------------------------------- 671
34.MAFF744087_F.oxysporumSchlechtendalf.sp.lactucae__Lettuce   671 ---------------------------------------- 671
35.MAFF726924_F.oxysporumSchlechtendalf.sp.lagenariae_Whitefloweredgourd 671 ---------------------------------------- 671
```

```
36.MAFF744003_F.oxysporumSchlechtendalf.sp.lagenariae_Squash)       672 ---------------------------------------- 672
37.MAFF305122_F.oxysporumSchlechtendalf.sp.melonis__Melon           671 ---------------------------------------- 671
38.MAFF306714_F.oxysporumSchlechtendalf.sp.momordicae_Balsampear    671 ---------------------------------------- 671
39.MAFF238905_F.oxysporumSchlechtendalf.sp.radicis-lycopersici_Tomato 674 ---------------------------------------- 674
40.MAFF150004_F.oxysporumSchlechtendalf.sp.spinaciae_Spinach        671 ---------------------------------------- 671
41.MAFF247034_F.oxysporumSchlechtendal__Goldenchain                 671 ---------------------------------------- 671
42.MAFF245747_F.oxysporumSchlechtendalf.sp.callistephi__Chinaaster  671 ---------------------------------------- 671
43.MAFF305115_FoxysporumSchlechtendalf.sp.batatas__Sweatpotato      703 ---------------------------------------- 703
44.MAFF150126_F.asiaticum_Seed                                      666 ---------------------------------------- 666
45.MAFF246738_F.solani_Angelica                                     703 ---------------------------------------- 703
46.MAFF246664_F.cugenangense_Perilla                                671 ---------------------------------------- 671
47.MH582420.1F.solanistrainMRC256                                   679 ---------------------------------------- 679
48.MAFF240361_F.babinda_Soil                                        693 ---------------------------------------- 693
49.MAFF242368_F.azukicola_Azukibean                                 708 ---------------------------------------- 708
50.MAFF241312_F.asiaticum_Soil,welshonionfield                      666 ---------------------------------------- 666
51.LT548416.1_F.culmorumpartialtef1a                                646 ---------------------------------------- 646
52.MAFF150124_F.asiaticum__Wheat                                    666 ---------------------------------------- 666
53.MAFF238806_F.begoniae_Oncidiumsp                                 705 ---------------------------------------- 705
54.MW594399.1_FusariumincarnatumisolateUD01C                        689 ---------------------------------------- 689
55.OP414923.1Pucciniagraminisf.sp.triticiisolateSHZPgt19            623 ---------------------------------------- 623
56.MT027094.1_BipolarisoryzaestrainOrL-2                            748 CTGCCCACATTGCCTGCAAGTTCTCTGAGCT--CCTCGAG 785
57.ON734360.1_AlternariaalternataisolateH126                        623 CCGCCCACATTGCTTGCAAGTTCTCTGAGCT--CCTCGAG 660
58.LC333578.1_StemphyliumlycopersiciSOasp2                            0 ---------------------------------------- 0
59.HQ718583.1_Colletotrichumgloeosporioidesisolateq-1               567 ---------------------------------------- 567
60.JN241603.1_AtheliarolfsiiisolateSR1                             1013 ---------------------------------------- 1013
61.KJ866474.1_RhizoctoniasolanistrainMHL-1                          683 ---------------------------------------- 683
62.JQ672424.1AlternariatriticinaisolateEGS17-061                    825 ---------------------------------------- 825
63.LT707559.1_P.capsicipartialtef1agene                             848 CGGCCCACGTTGCCTGCAAGTTCAAAGAGAT--CACGGAG 885
64.MW090051.1_CurvularialunatastrainCls-3                           742 CTGCCCACATTGCTTGCAAGTTCTCCGAGCT--CCTCGAG 779
65.DQ400892.1_Aspergillusterreus                                    452 ---------------------------------------- 452
66.DQ911416.1_Pythiumsp.quercumstrainPy292                          827 CGGCCCACGTTGCGTGCAAGTTCAAGGAGAT--CACGGAG 864
67.EU797495.1_Phytophthorasp.oaksoilPoland                          827 CGGCCCACGTTGCCTGCAAGTTCAAAGAGAT--CATCGAG 864
68.HM148321.1_Cladosporiumcucumerinum                               385 ---------------------------------------- 385
69.AF398888.1_SclerotiniasclerotiorumisolateSS1                       0 ---------------------------------------- 0
70.AF398888.1_S.sclerotiorumisolateSS1                                0 ---------------------------------------- 0
71.HPAB545908.1_Verticilliumnonalfalfaeisolate                      442 ---------------------------------------- 442
72.EF433315.1_CeratocystisfimbriatavoucherCMW15052                  747 ---------------------------------------- 747
73.MN159912.1_Botrytiscinerea                                       767 CTGCCCACATTGCTTGCAAGTTCTCTGAGCT--CCTCCAA 804
74.MF034741.1_PeltasterfructicolaisolateSRB92                        55 TCAGCTCGCTGCGCGAAAATTTTCCTTCTTTCGCCATCTG 94
75.LC440360.1_CercosporaasparagiCOasp2                              297 ---------------------------------------- 297
76.AY944105.1_MagnaportheoryzaeisolateSAG00T3()                     255 ---------------------------------------- 255
77.JX266586.1_CochliobolusmiyabeanusvoucherMFLUCC10-0733            757 CTGCCCACATTGCCTGCAAGTTCTCTGAGCT--CCTCGAG 794
78.MN393253.1_CorynesporacassiicolaisolateQHD001(MN393253.1UNVERIFIED) 0 ---------------------------------------- 0
79.MF375218.1_AgroatheliarolfsiiisolateBJB24                        544 ---------------------------------------- 544
80.MN106270.1_AgroatheliarolfsiistrainJ-12                          509 ---------------------------------------- 509
81.OQ732628.1_AgroatheliarolfsiiisolateBTCBSr3                      499 ---------------------------------------- 499
```

```
82.KY196185.1_ColletotrichumtruncatumstrainPAK53          468 ---------------------------------------- 468
83.GU935835.1_ColletotrichumcoccodesisolateC96002        1041 ---------------------------------------- 1041
84.MK085963.1_AlternariatenuissimaisolateSCCZ06             0 ---------------------------------------- 0
85.MT548042.1_AlternarialongipesstrainKY_2019_012           0 ---------------------------------------- 0
86.MN356465.1CalonectriamontanaisolateHSP4                489 ---------------------------------------- 489
87.OL694224.1_CalonectriacanadianastrainF099              504 ---------------------------------------- 504
88.MK803351.1_NeoscytalidiumdimidiatumstrainKale4-C        43 CACTGCACGTGTGCTGGGTTCCTGCGCCGAATTTGCCTTA 82
89.ON376993.1_CurvulariachiangmaiensisisolateND00J7       759 CTGCCCACATTGCTTGCAAGTTCTCCGAGCT--CCTCGAG 796
90.OQ383346.1_NeoscytalidiumdimidiatumisolateGKH-2         18 CACTGCACGTGTGCTGGGTTCCTGCGCCGAATTTGCCTTA 57
91.MF662595.1_NeoscytalidiumnovaehollandiaeisolateNeNo1    37 CACTGCACGTGTGCTGGGTTCCTGCGCCGAATTTGCCTTA 76
92.EF560588.1Melampsoralini                                656 ---------------------------------------- 656
93.LC590862.1_NeoscytalidiumdimidiatumPSU-HP01TEF1         11 CACTGCACGTGTGCTGGGTTCCTGCGCCGAATTTGCCTTA 50
94.KX278106.1_BotryosphaeriaqingyuanensisstrainCERC2947    36 -CCTGCACGTGTGCTGGGTTCCTGCGCCGAATTTGCCTTA 74
95.AJ578763.1_Blumeriagraminisf.sp.hordeicyp51             98 TACTGTTAACCTTCTTGAACGTATTGAAGCAGTTACTTTT 137
96.MF490858.1_CurvulariadactylocteniicolastrainCPC28810   747 CTGCCCACATTGCTTGCAAGTTCTCCGAGCT--CCTCGAG 784
97.KT287115.1_Bipolariscactivoraisolate3.8.6             801 CCGCCCACATTGCCTGCAAGTTCTCTGAGCT--CCTCGAG 838
98.MT560940.1_CurvulariacactivorastrainHLGH0118           758 CCGCCCACATTGCCTGCAAGTTCTCTGAGCT--CCTCGAG 795
99.OM714565.1_CurvulariaplantarumstrainM0134              784 CTGCCCACATTGCCTGCAAGTTCTCTGAGCT--CCTCGAG 821
100.MN159911.1_BotrytiscinereaSICAUCC19-0003              767 CTGCCCACATTGCTTGCAAGTTCTCTGAGCT--CCTCCAA 804
102.GU294713.1LasiodiplodiatheobromaestrainUCD2430TX        91 CCCTCC--CTCAGCAGCGCTGCGGCGCACAGTCCGCCTTA 128
103.KX868094.1_Mycosphaerellasp.isolateCRM20.1            475 ---------------------------------------- 475
104.LC599478.1Pseudocercosporapini-densifloraeMUCC534      54 CCATTTTT-TCGCTCTTATCATCGTTGCGCTGGCGACGAG 92
105.N584698.1BipolarissetariaestrainKBS4-2                812 CTGCCCACATTGCCTGCAAGTTCTCTGAGCT--CCTCGAG 849

2.OM160859.1_F.buharicum                                  560 ---------------------------------------- 560
1.LC727524.1_F.buharicum_OKI-1_Okura                      682 ---------------------------------------- 682
3.KX302919.1_F.sublunatum                                 521 ---------------------------------------- 521
4.LT996094.1_F.convolutans                                516 ---------------------------------------- 516
5.OM160861.1_F.abutilonis                                 630 ---------------------------------------- 630
6.OM160874.1_F.guadeloupense                              560 ---------------------------------------- 560
7.MH392475.1_F.graminearum                                430 ---------------------------------------- 430
8.MH582420.1_F.solani                                     540 ---------------------------------------- 540
9.MAFF244605_F.oxysporum                                  544 ---------------------------------------- 544
10.MAFF237278_F.contaminatum_Hylocereus                   711 ---------------------------------------- 711
11.MAFF237649_F.concentricum__Riceroot                    678 ---------------------------------------- 678
12.MAFF237650_F.concentricum__Wheat                       679 ---------------------------------------- 679
13.MAFF239869_F.mangiferae__Ryukyupine                    657 ---------------------------------------- 657
14.MAFF240460_F.fujikuroi_Passionfruit                    661 ---------------------------------------- 661
15.MAFF241317_F.graminearum_Wheat                         666 ---------------------------------------- 666
16.MAFF242670_F.ipomoeae_Wheat                            672 ---------------------------------------- 672
17.MAFF245129_F.concentricum_Fraxinus                     659 ---------------------------------------- 659
18.MAFF245395_F.cugenangense_Rhubarb                      671 ---------------------------------------- 671
19.MAFF246637_F.nirenbergiae_Strawberry                   671 ---------------------------------------- 671
20.MAFF246672_F.nirenbergiae_ChinesePeony                 671 ---------------------------------------- 671
21.MAFF246697_F.commune_Urallicoriceroot                  673 ---------------------------------------- 673
22.MAFF246729_F.falciforme_Angelica                       691 ---------------------------------------- 691
```

```
23.MAFF247220_F.duplospermum__Euwallaceasp 694 ---------------------------------------- 694
24.MAFF410760_F.odoratissimum_alpha 669 ---------------------------------------- 669
25.MAFF244605_FusariumoxysporumSchlechtendal_MAFF244605_Tomato 672 ---------------------------------------- 672
26.MAFF241326_F.asiaticum_Wheat 669 ---------------------------------------- 669
27.MAFF245014_F.asiaticum_Wildsoybean 666 ---------------------------------------- 666
28.MAFF150124_F.asiaticum__Wheat 666 ---------------------------------------- 666
29.OM135603.1F.algeriense 699 ---------------------------------------- 699
30.MAFF237465_F.penzigii_Aloe 704 ---------------------------------------- 704
31.MAFF103054_F.oxysporumSchlechtendalf.sp.cucumerinum_Cucumber 671 ---------------------------------------- 671
32.MAFF712246_F.oxysporumSchlechtendalf.sp.dianthi__Carnation 673 ---------------------------------------- 673
33.MAFF305558_F.oxysporumSchlechtendalf.sp.fragariae__Watermelon 671 ---------------------------------------- 671
34.MAFF744087_F.oxysporumSchlechtendalf.sp.lactucae__Lettuce 671 ---------------------------------------- 671
35.MAFF726924_F.oxysporumSchlechtendalf.sp.lagenariae_Whitefl000eredgourd 671 ---------------------------------------- 671
36.MAFF744003_F.oxysporumSchlechtendalf.sp.lagenariae_Squash) 672 ---------------------------------------- 672
37.MAFF305122_F.oxysporumSchlechtendalf.sp.melonis__Melon 671 ---------------------------------------- 671
38.MAFF306714_F.oxysporumSchlechtendalf.sp.momordicae_Balsampear 671 ---------------------------------------- 671
39.MAFF238905_F.oxysporumSchlechtendalf.sp.radicis-lycopersici_Tomato 674 ---------------------------------------- 674
40.MAFF150004_F.oxysporumSchlechtendalf.sp.spinaciae_Spinach 671 ---------------------------------------- 671
41.MAFF247034_F.oxysporumSchlechtendal__Goldenchain 671 ---------------------------------------- 671
42.MAFF245747_F.oxysporumSchlechtendalf.sp.callistephi__Chinaaster 671 ---------------------------------------- 671
43.MAFF305115_FoxysporumSchlechtendalf.sp.batatas__Sweatpotato 703 ---------------------------------------- 703
44.MAFF150126_F.asiaticum_Seed 666 ---------------------------------------- 666
45.MAFF246738_F.solani_Angelica 703 ---------------------------------------- 703
46.MAFF246664_F.cugenangense_Perilla 671 ---------------------------------------- 671
47.MH582420.1F.solanistrainMRC256 679 ---------------------------------------- 679
48.MAFF240361_F.babinda_Soil 693 ---------------------------------------- 693
49.MAFF242368_F.azukicola_Azukibean 708 ---------------------------------------- 708
50.MAFF241312_F.asiaticum_Soil,welshonionfield 666 ---------------------------------------- 666
51.LT548416.1_F.culmorumpartialtef1a 646 ---------------------------------------- 646
52.MAFF150124_F.asiaticum__Wheat 666 ---------------------------------------- 666
53.MAFF238806_F.begoniae_Oncidiumsp 705 ---------------------------------------- 705
54.MW594399.1_FusariumincarnatumisolateUD01C 689 ---------------------------------------- 689
55.OP414923.1Pucciniagraminisf.sp.triticiisolateSHZPgt19 623 ---------------------------------------- 623
56.MT027094.1_BipolarisoryzaestrainOrL-2 786 ----AAGATTGACCGCCGTACCGGAAAGTCTGTT-GAGAA 820
57.ON734360.1_AlternariaalternataisolateH126 661 ----AAGATTGACCGCCGTACCGGAAAGTCTGTT-GAGAA 695
58.LC333578.1_StemphyliumlycopersiciSOasp2 0 ---------------------------------------- 0
59.HQ718583.1_Colletotrichumgloeosporioidesisolateq-1 567 ---------------------------------------- 567
60.JN241603.1_AtheliarolfsiiisolateSR1 1013 ---------------------------------------- 1013
61.KJ866474.1_RhizoctoniasolanistrainMHL-1 683 ---------------------------------------- 683
62.JQ672424.1AlternariatriticinaisolateEGS17-061 825 ---------------------------------------- 825
63.LT707559.1_P.capsicipartialtef1agene 886 ----AAGATGGACCGTCGTTCGGGCAAGGTGCTC-GAGAC 920
64.MW090051.1_CurvularialunatastrainCls-3 780 ----AAGATCGACCGCCGTACCGGAAAGTCTGTT-GAGAA 814
65.DQ400892.1_Aspergillusterreus 452 ---------------------------------------- 452
66.DQ911416.1_Pythiumsp.quercumstrainPy292 865 ----AAGATGGACCGTCGTTCGGGCAAGGTGCTC-GAGGC 899
67.EU797495.1_Phytophthorasp.oaksoilPoland 865 ----AAGATGGACCGTCGTTCGGGCAAGGTNCCG-GAGAC 899
68.HM148321.1_Cladosporiumcucumerinum 385 ---------------------------------------- 385
```

```
69.AF398888.1_SclerotiniasclerotiorumisolateSS1                        0 ---------------------------------------- 0
70.AF398888.1_S.sclerotiorumisolateSS1                                 0 ---------------------------------------- 0
71.HPAB545908.1_Verticilliumnonalfalfaeisolate                       442 ---------------------------------------- 442
72.EF433315.1_CeratocystisfimbriatavoucherCMW15052                   747 ---------------------------------------- 747
73.MN159912.1_Botrytiscinerea                                        805 ----AAGATTGATCGTCGTACCGGTAAATCCATG-GAAGA 839
74.MF034741.1_PeltasterfructicolaisolateSRB92                         95 TTATGATGATGGAGGGGAATTTTCTCAGGCGGCGGGGCAT 134
75.LC440360.1_CercosporaasparagiCOasp2                               297 ---------------------------------------- 297
76.AY944105.1_MagnaportheoryzaeisolateSAG00T3()                      255 ---------------------------------------- 255
77.JX266586.1_CochliobolusmiyabeanusvoucherMFLUCC10-0733             795 ----AAGATTGACCGCCGTACCGGAAAGTCTGTT-GAGAA 829
78.MN393253.1_CorynesporacassiicolaisolateQHD001(MN393253.1UNVERIFIED)  0 ---------------------------------------- 0
79.MF375218.1_AgroatheliarolfsiiisolateBJB24                         544 ---------------------------------------- 544
80.MN106270.1_AgroatheliarolfsiistrainJ-12                           509 ---------------------------------------- 509
81.OQ732628.1_AgroatheliarolfsiiisolateBTCBSr3                       499 ---------------------------------------- 499
82.KY196185.1_ColletotrichumtruncatumstrainPAK53                     468 ---------------------------------------- 468
83.GU935835.1_ColletotrichumcoccodesisolateC96002                   1041 ---------------------------------------- 1041
84.MK085963.1_AlternariatenuissimaisolateSCCZ06                        0 ---------------------------------------- 0
85.MT548042.1_AlternarialongipesstrainKY_2019_012                      0 ---------------------------------------- 0
86.MN356465.1CalonectriamontanaisolateHSP4                           489 ---------------------------------------- 489
87.OL694224.1_CalonectriacanadianastrainF099                         504 ---------------------------------------- 504
88.MK803351.1_NeoscytalidiumdimidiatumstrainKale4-C                   83 TCGCT--TGGGTGAGGGGCATTTT-GGTGGTGGGGTT-GG 118
89.ON376993.1_CurvulariachiangmaiensisisolateND00J7                  797 ----AAGATCGACCGCCGTACCGGAAAGTCTGTT-GAGAA 831
90.OQ383346.1_NeoscytalidiumdimidiatumisolateGKH-2                    58 TCGCT--TGGGTGAGGGGCATTTT-GGTGGTGGGGTT-GG 93
91.MF662595.1_NeoscytalidiumnovaehollandiaeisolateNeNo1               77 TCGCT--TGGGTGAGGGGCATTTT-GGTGGTGGGGTT-GG 112
92.EF560588.1Melampsoralini                                          656 ---------------------------------------- 656
93.LC590862.1_NeoscytalidiumdimidiatumPSU-HP01TEF1                    51 TCGCT--TGGGTGAGGGGCATTTT-GGTGGTGGGGTT-GG 86
94.KX278106.1_BotryosphaeriaqingyuanensisstrainCERC2947               75 TCACTCTCTGGTGAGGGGCAATTT-CTTGGTGGGGCT-GG 112
95.AJ578763.1_Blumeriagraminisf.sp.hordeicyp51                       138 CAAGAATCCAAATGAGCCACCGATCGTGTTTCATTGGATT 177
96.MF490858.1_CurvulariadactylocteniicolastrainCPC28810              785 ----AAGATCGACCGCCGTACCGGAAAGTCTGTT-GAGAA 819
97.KT287115.1_Bipolariscactivoraisolate3.8.6                         839 ----AAGATTGACCGCCGTACCGGAAAGTCTGTT-GAGAA 873
98.MT560940.1_CurvulariacactivorastrainHLGH0118                      796 ----AAGATTGACCGCCGTACCGGAAAGTCTGTT-GAGAA 830
99.OM714565.1_CurvulariaplantarumstrainM0134                         822 ----AAGATCGACCGCCGTACCGGAAAGTCTGTT-GAGAA 856
100.MN159911.1_BotrytiscinereaSICAUCC19-0003                         805 ----AAGATTGATCGTCGTACCGGTAAATCCATG-GAAGA 839
102.GU294713.1LasiodiplodiatheobromaestrainUCD2430TX                  129 TCGCT--TTGATGAGGGGCATTTTTCGTGGTGGGGTTTGG 166
103.KX868094.1_Mycosphaerellasp.isolateCRM20.1                       475 ---------------------------------------- 475
104.LC599478.1Pseudocercosporapini-densifloraeMUCC534                  93 GGGCAAAATTTGGTGGGGTGCGAGAATTTCGCTTCGGCTC 132
105.N584698.1BipolarissetariaestrainKBS4-2                            850 ----AAGATTGACCGCCGTACCGGAAAGTCTGTT-GAGAA 884

2.OM160859.1_F.buharicum                                             560 ---------------------------------------- 560
1.LC727524.1_F.buharicum_OKI-1_Okura                                 682 ---------------------------------------- 682
3.KX302919.1_F.sublunatum                                            521 ---------------------------------------- 521
4.LT996094.1_F.convolutans                                           516 ---------------------------------------- 516
5.OM160861.1_F.abutilonis                                            630 ---------------------------------------- 630
6.OM160874.1_F.guadeloupense                                         560 ---------------------------------------- 560
7.MH392475.1_F.graminearum                                           430 ---------------------------------------- 430
8.MH582420.1_F.solani                                                540 ---------------------------------------- 540
9.MAFF244605_F.oxysporum                                             544 ---------------------------------------- 544
```

```
10.MAFF237278_F.contaminatum_Hylocereus                               711 ---------------------------------------- 711
11.MAFF237649_F.concentricum__Riceroot                                678 ---------------------------------------- 678
12.MAFF237650_F.concentricum__Wheat                                   679 ---------------------------------------- 679
13.MAFF239869_F.mangiferae__Ryukyupine                                657 ---------------------------------------- 657
14.MAFF240460_F.fujikuroi_Passionfruit                                661 ---------------------------------------- 661
15.MAFF241317_F.graminearum_Wheat                                     666 ---------------------------------------- 666
16.MAFF242670_F.ipomoeae_Wheat                                        672 ---------------------------------------- 672
17.MAFF245129_F.concentricum_Fraxinus                                 659 ---------------------------------------- 659
18.MAFF245395_F.cugenangense_Rhubarb                                  671 ---------------------------------------- 671
19.MAFF246637_F.nirenbergiae_Strawberry                               671 ---------------------------------------- 671
20.MAFF246672_F.nirenbergiae_ChinesePeony                             671 ---------------------------------------- 671
21.MAFF246697_F.commune_Urallicoriceroot                              673 ---------------------------------------- 673
22.MAFF246729_F.falciforme_Angelica                                   691 ---------------------------------------- 691
23.MAFF247220_F.duplospermum__Euwallaceasp                            694 ---------------------------------------- 694
24.MAFF410760_F.odoratissimum_alpha                                   669 ---------------------------------------- 669
25.MAFF244605_FusariumoxysporumSchlechtendal_MAFF244605_Tomato        672 ---------------------------------------- 672
26.MAFF241326_F.asiaticum_Wheat                                       669 ---------------------------------------- 669
27.MAFF245014_F.asiaticum_Wildsoybean                                 666 ---------------------------------------- 666
28.MAFF150124_F.asiaticum__Wheat                                      666 ---------------------------------------- 666
29.OM135603.1F.algeriense                                             699 ---------------------------------------- 699
30.MAFF237465_F.penzigii_Aloe                                         704 ---------------------------------------- 704
31.MAFF103054_F.oxysporumSchlechtendalf.sp.cucumerinum_Cucumber       671 ---------------------------------------- 671
32.MAFF712246_F.oxysporumSchlechtendalf.sp.dianthi__Carnation         673 ---------------------------------------- 673
33.MAFF305558_F.oxysporumSchlechtendalf.sp.fragariae__Watermelon      671 ---------------------------------------- 671
34.MAFF744087_F.oxysporumSchlechtendalf.sp.lactucae__Lettuce          671 ---------------------------------------- 671
35.MAFF726924_F.oxysporumSchlechtendalf.sp.lagenariae_Whitefloweredgourd 671 ---------------------------------------- 671
36.MAFF744003_F.oxysporumSchlechtendalf.sp.lagenariae_Squash)         672 ---------------------------------------- 672
37.MAFF305122_F.oxysporumSchlechtendalf.sp.melonis__Melon             671 ---------------------------------------- 671
38.MAFF306714_F.oxysporumSchlechtendalf.sp.momordicae_Balsampear      671 ---------------------------------------- 671
39.MAFF238905_F.oxysporumSchlechtendalf.sp.radicis-lycopersici_Tomato 674 ---------------------------------------- 674
40.MAFF150004_F.oxysporumSchlechtendalf.sp.spinaciae_Spinach          671 ---------------------------------------- 671
41.MAFF247034_F.oxysporumSchlechtendal__Goldenchain                   671 ---------------------------------------- 671
42.MAFF245747_F.oxysporumSchlechtendalf.sp.callistephi__Chinaaster    671 ---------------------------------------- 671
43.MAFF305115_FoxysporumSchlechtendalf.sp.batatas__Sweatpotato        703 ---------------------------------------- 703
44.MAFF150126_F.asiaticum_Seed                                        666 ---------------------------------------- 666
45.MAFF246738_F.solani_Angelica                                       703 ---------------------------------------- 703
46.MAFF246664_F.cugenangense_Perilla                                  671 ---------------------------------------- 671
47.MH582420.1F.solanistrainMRC256                                     679 ---------------------------------------- 679
48.MAFF240361_F.babinda_Soil                                          693 ---------------------------------------- 693
49.MAFF242368_F.azukicola_Azukibean                                   708 ---------------------------------------- 708
50.MAFF241312_F.asiaticum_Soil,welshonionfield                        666 ---------------------------------------- 666
51.LT548416.1_F.culmorumpartialtef1a                                  646 ---------------------------------------- 646
52.MAFF150124_F.asiaticum__Wheat                                      666 ---------------------------------------- 666
53.MAFF238806_F.begoniae_Oncidiumsp                                   705 ---------------------------------------- 705
54.MW594399.1_FusariumincarnatumisolateUD01C                          689 ---------------------------------------- 689
55.OP414923.1Pucciniagraminisf.sp.triticiisolateSHZPgt19              623 ---------------------------------------- 623
```

```
56.MT027094.1_BipolarisoryzaestrainOrL-2                               821 CTCCCCCAA---GTTCATC--AAGTCTGGTGACGC-CGCC 854
57.ON734360.1_AlternariaalternataisolateH126                           696 CTCTCCCAA---GTTCATC--AAGTCCGGTGACGC-CGCC 729
58.LC333578.1_StemphyliumlycopersiciSOasp2                               0 ---------------------------------------- 0
59.HQ718583.1_Colletotrichumgloeosporioidesisolateq-1                  567 ---------------------------------------- 567
60.JN241603.1_AtheliarolfsiiisolateSR1                                1013 ---------------------------------------- 1013
61.KJ866474.1_RhizoctoniasolanistrainMHL-1                             683 ---------------------------------------- 683
62.JQ672424.1AlternariatriticinaisolateEGS17-061                       825 ---------------------------------------- 825
63.LT707559.1_P.capsicipartialtef1agene                                921 TGCCCCCAA---GTTCGTG--AAGTCGGGTGATGC-CTGC 954
64.MW090051.1_CurvularialunatastrainCls-3                              815 CTCCCCCAA---GTTCATC--AAGTCCGGTGACGC-TGCC 848
65.DQ400892.1_Aspergillusterreus                                       452 ---------------------------------------- 452
66.DQ911416.1_Pythiumsp.quercumstrainPy292                             900 GAACCCGAA---GTTCGTC--AAGTCGGGTGACGC-CTG- 932
67.EU797495.1_Phytophthorasp.oaksoilPoland                             900 NGCCCCCAA---GTTCGTC--AAGTCGGGCGATGC-C--- 930
68.HM148321.1_Cladosporiumcucumerinum                                  385 ---------------------------------------- 385
69.AF398888.1_SclerotiniasclerotiorumisolateSS1                          0 ---------------------------------------- 0
70.AF398888.1_S.sclerotiorumisolateSS1                                   0 ---------------------------------------- 0
71.HPAB545908.1_Verticilliumnonalfalfaeisolate                         442 ---------------------------------------- 442
72.EF433315.1_CeratocystisfimbriatavoucherCMW15052                     747 ---------------------------------------- 747
73.MN159912.1_Botrytiscinerea                                          840 CTCTCCAAA---ATTCATC--AAGTCTGGTGATGC-TGCC 873
74.MF034741.1_PeltasterfructicolaisolateSRB92                          135 TATCTCTATCACGCTTGCCCGTTTGGGACACCTACACTCA 174
75.LC440360.1_CercosporaasparagiCOasp2                                 297 ---------------------------------------- 297
76.AY944105.1_MagnaportheoryzaeisolateSAG00T3()                        255 ---------------------------------------- 255
77.JX266586.1_CochliobolusmiyabeanusvoucherMFLUCC10-0733               830 CTCCCCCAA---GTTCATC--AAGTCTGGTGACGC-CGCC 863
78.MN393253.1_CorynesporacassiicolaisolateQHD001(MN393253.1UNVERIFIED)   0 ---------------------------------------- 0
79.MF375218.1_AgroatheliarolfsiiisolateBJB24                           544 ---------------------------------------- 544
80.MN106270.1_AgroatheliarolfsiistrainJ-12                             509 ---------------------------------------- 509
81.OQ732628.1_AgroatheliarolfsiiisolateBTCBSr3                         499 ---------------------------------------- 499
82.KY196185.1_ColletotrichumtruncatumstrainPAK53                       468 ---------------------------------------- 468
83.GU935835.1_ColletotrichumcoccodesisolateC96002                     1041 ---------------------------------------- 1041
84.MK085963.1_AlternariatenuissimaisolateSCCZ06                          0 ---------------------------------------- 0
85.MT548042.1_AlternarialongipesstrainKY_2019_012                        0 ---------------------------------------- 0
86.MN356465.1CalonectriamontanaisolateHSP4                             489 ---------------------------------------- 489
87.OL694224.1_CalonectriacanadianastrainF099                           504 ---------------------------------------- 504
88.MK803351.1_NeoscytalidiumdimidiatumstrainKale4-C                    119 CCCGCGCTAGGCCTCGTTCGGTCTTCGGCAAAATCTCCGC 158
89.ON376993.1_CurvulariachiangmaiensisisolateND00J7                    832 CTCCCCCAA---GTTCATC--AAGTCCGGTGACGC-TGCC 865
90.OQ383346.1_NeoscytalidiumdimidiatumisolateGKH-2                      94 CCCGCGCTAGGCCTCGTTCGGTCTTCGGCAAAATCTCCGC 133
91.MF662595.1_NeoscytalidiumnovaehollandiaeisolateNeNo1                113 CCCGCGCTAGGCCTCGTTCGGTCCTCGGCAAAATCTCCGC 152
92.EF560588.1Melampsoralini                                             656 ---------------------------------------- 656
93.LC590862.1_NeoscytalidiumdimidiatumPSU-HP01TEF1                      87 CCCGCGCTAGGCCTCGTTCGGTCTTCGGCAAAATCTCCGC 126
94.KX278106.1_BotryosphaeriaqingyuanensisstrainCERC2947                113 CCCGCGCTAAGCCTCGTTTGGTCTTCGGCAAAATCTCCGC 152
95.AJ578763.1_Blumeriagraminisf.sp.hordeicyp51                         178 CCTATCATTGGAAGTACAATTTCATATGGAATGAATCCCT 217
96.MF490858.1_CurvulariadactylocteniicolastrainCPC28810                820 CTCCCCCAA---GTTCATC--AAGTCCGGTGACGC-TGCC 853
97.KT287115.1_Bipolariscactivoraisolate3.8.6                           874 CTCTCCCAA---GTTCATC--AAGTCCGGTGACGC-CGCC 907
98.MT560940.1_CurvulariacactivorastrainHLGH0118                        831 CTCTCCCAA---GTTCATC--AAGTCCGGTGACGC-CGCC 864
99.OM714565.1_CurvulariaplantarumstrainM0134                           857 CTCCCCCAA---GTTCATC--AAGTCCGGTGACGC-CGCC 890
100.MN159911.1_BotrytiscinereaSICAUCC19-0003                           840 CTCTCCAAA---ATTCATC--AAGTCTGGTGATGC-TGCC 873
102.GU294713.1LasiodiplodiatheobromaestrainUCD2430TX                    167 CCCGCGCTA-GCCTCGTCTGGG-TTCGGCAAAATCACCGC 204
```

```
103.KX868094.1_Mycosphaerellasp.isolateCRM20.1                    475 ---------------------------------------- 475
104.LC599478.1Pseudocercosporapini-densifloraeMUCC534             133 CACAGCCAATGACTTCATCTCAAGCCTCAGCACACATTCC 172
105.N584698.1BipolarissetariaestrainKBS4-2                        885 CTCCCCCAA---GTTCATC--AAGTCCGGTGACGC-CGCC 918

2.OM160859.1_F.buharicum                                          560 ---------------------------------------- 560
1.LC727524.1_F.buharicum_OKI-1_Okura                              682 ---------------------------------------- 682
3.KX302919.1_F.sublunatum                                         521 ---------------------------------------- 521
4.LT996094.1_F.convolutans                                        516 ---------------------------------------- 516
5.OM160861.1_F.abutilonis                                         630 ---------------------------------------- 630
6.OM160874.1_F.guadeloupense                                      560 ---------------------------------------- 560
7.MH392475.1_F.graminearum                                        430 ---------------------------------------- 430
8.MH582420.1_F.solani                                             540 ---------------------------------------- 540
9.MAFF244605_F.oxysporum                                          544 ---------------------------------------- 544
10.MAFF237278_F.contaminatum_Hylocereus                           711 ---------------------------------------- 711
11.MAFF237649_F.concentricum__Riceroot                            678 ---------------------------------------- 678
12.MAFF237650_F.concentricum__Wheat                               679 ---------------------------------------- 679
13.MAFF239869_F.mangiferae__Ryukyupine                            657 ---------------------------------------- 657
14.MAFF240460_F.fujikuroi_Passionfruit                            661 ---------------------------------------- 661
15.MAFF241317_F.graminearum_Wheat                                 666 ---------------------------------------- 666
16.MAFF242670_F.ipomoeae_Wheat                                    672 ---------------------------------------- 672
17.MAFF245129_F.concentricum_Fraxinus                             659 ---------------------------------------- 659
18.MAFF245395_F.cugenangense_Rhubarb                              671 ---------------------------------------- 671
19.MAFF246637_F.nirenbergiae_Strawberry                           671 ---------------------------------------- 671
20.MAFF246672_F.nirenbergiae_ChinesePeony                         671 ---------------------------------------- 671
21.MAFF246697_F.commune_Urallicoriceroot                          673 ---------------------------------------- 673
22.MAFF246729_F.falciforme_Angelica                               691 ---------------------------------------- 691
23.MAFF247220_F.duplospermum__Euwallaceasp                        694 ---------------------------------------- 694
24.MAFF410760_F.odoratissimum_alpha                               669 ---------------------------------------- 669
25.MAFF244605_FusariumoxysporumSchlechtendal_MAFF244605_Tomato    672 ---------------------------------------- 672
26.MAFF241326_F.asiaticum_Wheat                                   669 ---------------------------------------- 669
27.MAFF245014_F.asiaticum_Wildsoybean                             666 ---------------------------------------- 666
28.MAFF150124_F.asiaticum__Wheat                                  666 ---------------------------------------- 666
29.OM135603.1F.algeriense                                         699 ---------------------------------------- 699
30.MAFF237465_F.penzigii_Aloe                                     704 ---------------------------------------- 704
31.MAFF103054_F.oxysporumSchlechtendalf.sp.cucumerinum_Cucumber   671 ---------------------------------------- 671
32.MAFF712246_F.oxysporumSchlechtendalf.sp.dianthi__Carnation     673 ---------------------------------------- 673
33.MAFF305558_F.oxysporumSchlechtendalf.sp.fragariae__Watermelon  671 ---------------------------------------- 671
34.MAFF744087_F.oxysporumSchlechtendalf.sp.lactucae__Lettuce      671 ---------------------------------------- 671
35.MAFF726924_F.oxysporumSchlechtendalf.sp.lagenariae_Whitefloweredgourd 671 ---------------------------------------- 671
36.MAFF744003_F.oxysporumSchlechtendalf.sp.lagenariae_Squash)     672 ---------------------------------------- 672
37.MAFF305122_F.oxysporumSchlechtendalf.sp.melonis__Melon         671 ---------------------------------------- 671
38.MAFF306714_F.oxysporumSchlechtendalf.sp.momordicae_Balsampear  671 ---------------------------------------- 671
39.MAFF238905_F.oxysporumSchlechtendalf.sp.radicis-lycopersici_Tomato 674 ---------------------------------------- 674
40.MAFF150004_F.oxysporumSchlechtendalf.sp.spinaciae_Spinach      671 ---------------------------------------- 671
41.MAFF247034_F.oxysporumSchlechtendal__Goldenchain               671 ---------------------------------------- 671
42.MAFF245747_F.oxysporumSchlechtendalf.sp.callistephi__Chinaaster 671 ---------------------------------------- 671
```

43.MAFF305115_FoxysporumSchlechtendalf.sp.batatas__Sweatpotato	703	--	703
44.MAFF150126_F.asiaticum_Seed	666	--	666
45.MAFF246738_F.solani_Angelica	703	--	703
46.MAFF246664_F.cugenangense_Perilla	671	--	671
47.MH582420.1F.solanistrainMRC256	679	--	679
48.MAFF240361_F.babinda_Soil	693	--	693
49.MAFF242368_F.azukicola_Azukibean	708	--	708
50.MAFF241312_F.asiaticum_Soil,welshonionfield	666	--	666
51.LT548416.1_F.culmorumpartialtef1a	646	--	646
52.MAFF150124_F.asiaticum__Wheat	666	--	666
53.MAFF238806_F.begoniae_Oncidiumsp	705	--	705
54.MW594399.1_FusariumincarnatumisolateUD01C	689	--	689
55.OP414923.1Pucciniagraminisf.sp.triticiisolateSHZPgt19	623	--	623
56.MT027094.1_BipolarisoryzaestrainOrL-2	855	ATCGTCAAGATGGTTCCCTCCAAGCCCATGTGCGTTGAGG	894
57.ON734360.1_AlternariaalternataisolateH126	730	ATCGTCAAGATGGTTCCCTCCAAGCCCATGTGCGTTGAGG	769
58.LC333578.1_StemphyliumlycopersiciSOasp2	0	--	0
59.HQ718583.1_Colletotrichumgloeosporioidesisolateq-1	567	--	567
60.JN241603.1_AtheliarolfsiiisolateSR1	1013	--	1013
61.KJ866474.1_RhizoctoniasolanistrainMHL-1	683	--	683
62.JQ672424.1AlternariatriticinaisolateEGS17-061	825	--	825
63.LT707559.1_P.capsicipartialtef1agene	955	ATGGTCATCC------------------------------	964
64.MW090051.1_CurvularialunatastrainCls-3	849	ATCGTCAAGATGGTTCCCTCCAAGCCCATGTGCGTTGAGG	888
65.DQ400892.1_Aspergillusterreus	452	--	452
66.DQ911416.1_Pythiumsp.quercumstrainPy292	932	--	932
67.EU797495.1_Phytophthorasp.oaksoilPoland	930	--	930
68.HM148321.1_Cladosporiumcucumerinum	385	--	385
69.AF398888.1_SclerotiniasclerotiorumisolateSS1	0	--	0
70.AF398888.1_S.sclerotiorumisolateSS1	0	--	0
71.HPAB545908.1_Verticilliumnonalfalfaeisolate	442	--	442
72.EF433315.1_CeratocystisfimbriatavoucherCMW15052	747	--	747
73.MN159912.1_Botrytiscinerea	874	ATCGTCAAGATGGTTCCATCCAAGCCTATGTGTGTTGAGG	913
74.MF034741.1_PeltasterfructicolaisolateSRB92	175	ACACACCACATGAATGACTCGA-GAACATGAACA-CGAGT	212
75.LC440360.1_CercosporaasparagiCOasp2	297	--	297
76.AY944105.1_MagnaportheoryzaeisolateSAG00T3()	255	--	255
77.JX266586.1_CochliobolusmiyabeanusvoucherMFLUCC10-0733	864	ATCGTCAAGATGGTTCCCTCCAAGCCCATGTGCGTTGAGG	903
78.MN393253.1_CorynesporacassiicolaisolateQHD001(MN393253.1UNVERIFIED)	0	--	0
79.MF375218.1_AgroatheliarolfsiiisolateBJB24	544	--	544
80.MN106270.1_AgroatheliarolfsiistrainJ-12	509	--	509
81.OQ732628.1_AgroatheliarolfsiiisolateBTCBSr3	499	--	499
82.KY196185.1_ColletotrichumtruncatumstrainPAK53	468	--	468
83.GU935835.1_ColletotrichumcoccodesisolateC96002	1041	--	1041
84.MK085963.1_AlternariatenuissimaisolateSCCZ06	0	--	0
85.MT548042.1_AlternarialongipesstrainKY_2019_012	0	--	0
86.MN356465.1CalonectriamontanaisolateHSP4	489	--	489
87.OL694224.1_CalonectriacanadianastrainF099	504	--	504
88.MK803351.1_NeoscytalidiumdimidiatumstrainKale4-C	159	ATCAGGATTTTTTGCGACCGGCGTGCGACCGACACG--CG	196

```
89.ON376993.1_CurvulariachiangmaiensisisolateND00J7            866 ATCGTCAAGATGGTTCCCTCCAAGCCCATGTGCGTTGAGG 905
90.OQ383346.1_NeoscytalidiumdimidiatumisolateGKH-2             134 ATCAGGATTTTTTGCGACCGGCGTGCGACCGACACG--CG 171
91.MF662595.1_NeoscytalidiumnovaehollandiaeisolateNeNo1        153 ATCAGGATTTTTTGCGACCGGCGTGCGACCGACACG--CG 190
92.EF560588.1Melampsoralini                                     656 ---------------------------------------- 656
93.LC590862.1_NeoscytalidiumdimidiatumPSU-HP01TEF1             127 ATCAGGATTTTTTGCGACCGGCGTGCGACCGACACG--CG 164
94.KX278106.1_BotryosphaeriaqingyuanensisstrainCERC2947         153 ATCTGGATTTTTTGTGACCGGCGTGCGACCGACGCGAACA 192
95.AJ578763.1_Blumeriagraminisf.sp.hordeicyp51                  218 ACAAATTCTTTCATGAATCCCAAGCCAAGGTCAGTTGCCG 257
96.MF490858.1_CurvulariadactylocteniicolastrainCPC28810         854 ATCGTCAAGATGGTTCCCTCCAAGCCCATGTGCGTTGAGG 893
97.KT287115.1_Bipolariscactivoraisolate3.8.6                    908 ATCGTCAAGATGGTTCCCTCCAAGCCCATGTGCGTTGAGG 947
98.MT560940.1_CurvulariacactivorastrainHLGH0118                 865 ATCGTCAAGATGGTTCCCTCCAAGCCCATGTGGGTTGAGG 904
99.OM714565.1_CurvulariaplantarumstrainM0134                    891 ATCGTCAAGATGGTTCCCTCCAAGCCCATGTGCGTTGAGG 930
100.MN159911.1_BotrytiscinereaSICAUCC19-0003                    874 ATCGTCAAGATGGTTCCATCCAAGCCTATGTGTGTTGAGG 913
102.GU294713.1LasiodiplodiatheobromaestrainUCD2430TX            205 ACTTGGTTTTTTTGCGACCGGCGTCTGGCCGACGCGCCTC 244
103.KX868094.1_Mycosphaerellasp.isolateCRM20.1                  475 ---------------------------------------- 475
104.LC599478.1Pseudocercosporapini-densifloraeMUCC534           173 TTCTCCCACCCTTTGGCATCGACAGCGACGTCCCTCTTCG 212
105.N584698.1BipolarissetariaestrainKBS4-2                      919 ATCGTCAAGATGGTTCCCTCCAAGCCCATGTGCGTTGAGG 958

2.OM160859.1_F.buharicum                                        560 ---------------------------------------- 560
1.LC727524.1_F.buharicum_OKI-1_Okura                            682 ---------------------------------------- 682
3.KX302919.1_F.sublunatum                                       521 ---------------------------------------- 521
4.LT996094.1_F.convolutans                                      516 ---------------------------------------- 516
5.OM160861.1_F.abutilonis                                       630 ---------------------------------------- 630
6.OM160874.1_F.guadeloupense                                    560 ---------------------------------------- 560
7.MH392475.1_F.graminearum                                      430 ---------------------------------------- 430
8.MH582420.1_F.solani                                           540 ---------------------------------------- 540
9.MAFF244605_F.oxysporum                                        544 ---------------------------------------- 544
10.MAFF237278_F.contaminatum_Hylocereus                         711 ---------------------------------------- 711
11.MAFF237649_F.concentricum__Riceroot                          678 ---------------------------------------- 678
12.MAFF237650_F.concentricum__Wheat                             679 ---------------------------------------- 679
13.MAFF239869_F.mangiferae__Ryukyupine                          657 ---------------------------------------- 657
14.MAFF240460_F.fujikuroi_Passionfruit                          661 ---------------------------------------- 661
15.MAFF241317_F.graminearum_Wheat                               666 ---------------------------------------- 666
16.MAFF242670_F.ipomoeae_Wheat                                  672 ---------------------------------------- 672
17.MAFF245129_F.concentricum_Fraxinus                           659 ---------------------------------------- 659
18.MAFF245395_F.cugenangense_Rhubarb                            671 ---------------------------------------- 671
19.MAFF246637_F.nirenbergiae_Strawberry                         671 ---------------------------------------- 671
20.MAFF246672_F.nirenbergiae_ChinesePeony                       671 ---------------------------------------- 671
21.MAFF246697_F.commune_Urallicoriceroot                        673 ---------------------------------------- 673
22.MAFF246729_F.falciforme_Angelica                             691 ---------------------------------------- 691
23.MAFF247220_F.duplospermum__Euwallaceasp                      694 ---------------------------------------- 694
24.MAFF410760_F.odoratissimum_alpha                             669 ---------------------------------------- 669
25.MAFF244605_FusariumoxysporumSchlechtendal_MAFF244605_Tomato  672 ---------------------------------------- 672
26.MAFF241326_F.asiaticum_Wheat                                 669 ---------------------------------------- 669
27.MAFF245014_F.asiaticum_Wildsoybean                           666 ---------------------------------------- 666
28.MAFF150124_F.asiaticum__Wheat                                666 ---------------------------------------- 666
29.OM135603.1F.algeriense                                       699 ---------------------------------------- 699
```

```
30.MAFF237465_F.penzigii_Aloe                                 704 ---------------------------------------- 704
31.MAFF103054_F.oxysporumSchlechtendalf.sp.cucumerinum_Cucumber 671 ---------------------------------------- 671
32.MAFF712246_F.oxysporumSchlechtendalf.sp.dianthi__Carnation  673 ---------------------------------------- 673
33.MAFF305558_F.oxysporumSchlechtendalf.sp.fragariae__Watermelon 671 ---------------------------------------- 671
34.MAFF744087_F.oxysporumSchlechtendalf.sp.lactucae__Lettuce   671 ---------------------------------------- 671
35.MAFF726924_F.oxysporumSchlechtendalf.sp.lagenariae_Whitefloweredgourd 671 ---------------------------------------- 671
36.MAFF744003_F.oxysporumSchlechtendalf.sp.lagenariae_Squash)  672 ---------------------------------------- 672
37.MAFF305122_F.oxysporumSchlechtendalf.sp.melonis__Melon      671 ---------------------------------------- 671
38.MAFF306714_F.oxysporumSchlechtendalf.sp.momordicae_Balsampear 671 ---------------------------------------- 671
39.MAFF238905_F.oxysporumSchlechtendalf.sp.radicis-lycopersici_Tomato 674 ---------------------------------------- 674
40.MAFF150004_F.oxysporumSchlechtendalf.sp.spinaciae_Spinach   671 ---------------------------------------- 671
41.MAFF247034_F.oxysporumSchlechtendal__Goldenchain            671 ---------------------------------------- 671
42.MAFF245747_F.oxysporumSchlechtendalf.sp.callistephi__Chinaaster 671 ---------------------------------------- 671
43.MAFF305115_FoxysporumSchlechtendalf.sp.batatas__Sweatpotato 703 ---------------------------------------- 703
44.MAFF150126_F.asiaticum_Seed                                666 ---------------------------------------- 666
45.MAFF246738_F.solani_Angelica                               703 ---------------------------------------- 703
46.MAFF246664_F.cugenangense_Perilla                          671 ---------------------------------------- 671
47.MH582420.1F.solanistrainMRC256                             679 ---------------------------------------- 679
48.MAFF240361_F.babinda_Soil                                  693 ---------------------------------------- 693
49.MAFF242368_F.azukicola_Azukibean                           708 ---------------------------------------- 708
50.MAFF241312_F.asiaticum_Soil,welshonionfield                666 ---------------------------------------- 666
51.LT548416.1_F.culmorumpartialtef1a                          646 ---------------------------------------- 646
52.MAFF150124_F.asiaticum__Wheat                              666 ---------------------------------------- 666
53.MAFF238806_F.begoniae_Oncidiumsp                           705 ---------------------------------------- 705
54.MW594399.1_FusariumincarnatumisolateUD01C                  689 ---------------------------------------- 689
55.OP414923.1Pucciniagraminisf.sp.triticiisolateSHZPgt19      623 ---------------------------------------- 623
56.MT027094.1_BipolarisoryzaestrainOrL-2                      895 C--------------------------------------- 895
57.ON734360.1_AlternariaalternataisolateH126                  770 CTTTCACTGACTACCCTCCTCTCGGTCGTTTCGCTGTCCG 809
58.LC333578.1_StemphyliumlycopersiciSOasp2                      0 ---------------------------------------- 0
59.HQ718583.1_Colletotrichumgloeosporioidesisolateq-1         567 ---------------------------------------- 567
60.JN241603.1_AtheliarolfsiiisolateSR1                       1013 ---------------------------------------- 1013
61.KJ866474.1_RhizoctoniasolanistrainMHL-1                    683 ---------------------------------------- 683
62.JQ672424.1AlternariatriticinaisolateEGS17-061              825 ---------------------------------------- 825
63.LT707559.1_P.capsicipartialtef1agene                       964 ---------------------------------------- 964
64.MW090051.1_CurvularialunatastrainCls-3                     889 CTTTCACTGACTACCCTCCT-------------------- 908
65.DQ400892.1_Aspergillusterreus                              452 ---------------------------------------- 452
66.DQ911416.1_Pythiumsp.quercumstrainPy292                    932 ---------------------------------------- 932
67.EU797495.1_Phytophthorasp.oaksoilPoland                    930 ---------------------------------------- 930
68.HM148321.1_Cladosporiumcucumerinum                         385 ---------------------------------------- 385
69.AF398888.1_SclerotiniasclerotiorumisolateSS1                 0 ---------------------------------------- 0
70.AF398888.1_S.sclerotiorumisolateSS1                          0 ---------------------------------------- 0
71.HPAB545908.1_Verticilliumnonalfalfaeisolate                442 ---------------------------------------- 442
72.EF433315.1_CeratocystisfimbriatavoucherCMW15052            747 ---------------------------------------- 747
73.MN159912.1_Botrytiscinerea                                 914 CCTTCACTGAGTACCCAC---------------------- 931
74.MF034741.1_PeltasterfructicolaisolateSRB92                 213 CGCTCA-TGGATACC-TCTCCTCGACACTCATCACGTCAA 250
75.LC440360.1_CercosporaasparagiCOasp2                        297 ---------------------------------------- 297
```

```
76.AY944105.1_MagnaportheoryzaeisolateSAG00T3()                        255 ---------------------------------------- 255
77.JX266586.1_CochliobolusmiyabeanusvoucherMFLUCC10-0733                904 CTTTCACTGACTACC------------------------- 918
78.MN393253.1_CorynesporacassiicolaisolateQHD001(MN393253.1UNVERIFIED)    0 ---------------------------------------- 0
79.MF375218.1_AgroatheliarolfsiiisolateBJB24                            544 ---------------------------------------- 544
80.MN106270.1_AgroatheliarolfsiistrainJ-12                              509 ---------------------------------------- 509
81.OQ732628.1_AgroatheliarolfsiiisolateBTCBSr3                          499 ---------------------------------------- 499
82.KY196185.1_ColletotrichumtruncatumstrainPAK53                        468 ---------------------------------------- 468
83.GU935835.1_ColletotrichumcoccodesisolateC96002                      1041 ---------------------------------------- 1041
84.MK085963.1_AlternariatenuissimaisolateSCCZ06                           0 ---------------------------------------- 0
85.MT548042.1_AlternarialongipesstrainKY_2019_012                         0 ---------------------------------------- 0
86.MN356465.1CalonectriamontanaisolateHSP4                              489 ---------------------------------------- 489
87.OL694224.1_CalonectriacanadianastrainF099                            504 ---------------------------------------- 504
88.MK803351.1_NeoscytalidiumdimidiatumstrainKale4-C                     197 CCTCCTAGCGTCGCTTGCA---GCCACTCACGTCCGCCCG 233
89.ON376993.1_CurvulariachiangmaiensisisolateND00J7                     906 CTTTCACTGACTACCCTCCTCTCGGTCGTTTCG------- 938
90.OQ383346.1_NeoscytalidiumdimidiatumisolateGKH-2                      172 CCTCCTAGCGTCGCTTGCA---GCCACTCACGTCCGCCCG 208
91.MF662595.1_NeoscytalidiumnovaehollandiaeisolateNeNo1                191 CCTCCTAGCGTCGCTTGCA---GCCACTCACGTTCGCCCG 227
92.EF560588.1Melampsoralini                                             656 ---------------------------------------- 656
93.LC590862.1_NeoscytalidiumdimidiatumPSU-HP01TEF1                      165 CCTCCTAGCGTCGCTTGCA---GCCACTCACGTCCGCCCG 201
94.KX278106.1_BotryosphaeriaqingyuanensisstrainCERC2947                 193 CCCCTCACCAACGCTTCCA---GCCACTCACGTTCGTCTA 229
95.AJ578763.1_Blumeriagraminisf.sp.hordeicyp51                          258 CTTACACTCTCTGAGGAATACGATATTAATTGAGGTAAAG 297
96.MF490858.1_CurvulariadactylocteniicolastrainCPC28810                 894 CTTTCACTGACTACCCTCCTCTCGGTCGTTTCGCCGT--- 930
97.KT287115.1_Bipolariscactivoraisolate3.8.6                           948 CCTTCACTGACTACCCTCCTCTTGGTCGTTTCGCCGTCCG 987
98.MT560940.1_CurvulariacactivorastrainHLGH0118                         905 CCTTCACTGACTACCCTCCTCTTGGTCGTTTCGCCGTCCG 944
99.OM714565.1_CurvulariaplantarumstrainM0134                            931 CCTTCACTGACTACCCTCCTCTCGGTCGTTTCGCCGTCCG 970
100.MN159911.1_BotrytiscinereaSICAUCC19-0003                            914 CCTTCACTGAGTACCCAC---------------------- 931
102.GU294713.1LasiodiplodiatheobromaestrainUCD2430TX                     245 CCCACTAGCGAAAAATGCTCTGACCACTCATGTACCGTCG 284
103.KX868094.1_Mycosphaerellasp.isolateCRM20.1                          475 ---------------------------------------- 475
104.LC599478.1Pseudocercosporapini-densifloraeMUCC534                   213 AA--CACTGGC-ATTGACTGCGATGTCCTCT-GATGTCCT 248
105.N584698.1BipolarissetariaestrainKBS4-2                              959 CTTTCACTGACTACCCTCCTCTTGGTCGTTTCGCCGTCCG 998

2.OM160859.1_F.buharicum                                                560 ---------------------------------------- 560
1.LC727524.1_F.buharicum_OKI-1_Okura                                    682 ---------------------------------------- 682
3.KX302919.1_F.sublunatum                                               521 ---------------------------------------- 521
4.LT996094.1_F.convolutans                                              516 ---------------------------------------- 516
5.OM160861.1_F.abutilonis                                               630 ---------------------------------------- 630
6.OM160874.1_F.guadeloupense                                            560 ---------------------------------------- 560
7.MH392475.1_F.graminearum                                              430 ---------------------------------------- 430
8.MH582420.1_F.solani                                                   540 ---------------------------------------- 540
9.MAFF244605_F.oxysporum                                                544 ---------------------------------------- 544
10.MAFF237278_F.contaminatum_Hylocereus                                 711 ---------------------------------------- 711
11.MAFF237649_F.concentricum__Riceroot                                  678 ---------------------------------------- 678
12.MAFF237650_F.concentricum__Wheat                                     679 ---------------------------------------- 679
13.MAFF239869_F.mangiferae__Ryukyupine                                  657 ---------------------------------------- 657
14.MAFF240460_F.fujikuroi_Passionfruit                                  661 ---------------------------------------- 661
15.MAFF241317_F.graminearum_Wheat                                       666 ---------------------------------------- 666
16.MAFF242670_F.ipomoeae_Wheat                                          672 ---------------------------------------- 672
```

17.MAFF245129_F.concentricum_Fraxinus	659	--	659
18.MAFF245395_F.cugenangense_Rhubarb	671	--	671
19.MAFF246637_F.nirenbergiae_Strawberry	671	--	671
20.MAFF246672_F.nirenbergiae_ChinesePeony	671	--	671
21.MAFF246697_F.commune_Urallicoriceroot	673	--	673
22.MAFF246729_F.falciforme_Angelica	691	--	691
23.MAFF247220_F.duplospermum__Euwallaceasp	694	--	694
24.MAFF410760_F.odoratissimum_alpha	669	--	669
25.MAFF244605_FusariumoxysporumSchlechtendal_MAFF244605_Tomato	672	--	672
26.MAFF241326_F.asiaticum_Wheat	669	--	669
27.MAFF245014_F.asiaticum_Wildsoybean	666	--	666
28.MAFF150124_F.asiaticum__Wheat	666	--	666
29.OM135603.1F.algeriense	699	--	699
30.MAFF237465_F.penzigii_Aloe	704	--	704
31.MAFF103054_F.oxysporumSchlechtendalf.sp.cucumerinum_Cucumber	671	--	671
32.MAFF712246_F.oxysporumSchlechtendalf.sp.dianthi__Carnation	673	--	673
33.MAFF305558_F.oxysporumSchlechtendalf.sp.fragariae__Watermelon	671	--	671
34.MAFF744087_F.oxysporumSchlechtendalf.sp.lactucae__Lettuce	671	--	671
35.MAFF726924_F.oxysporumSchlechtendalf.sp.lagenariae_Whiteflrweredgourd	671	--	671
36.MAFF744003_F.oxysporumSchlechtendalf.sp.lagenariae_Squash)	672	--	672
37.MAFF305122_F.oxysporumSchlechtendalf.sp.melonis__Melon	671	--	671
38.MAFF306714_F.oxysporumSchlechtendalf.sp.momordicae_Balsampear	671	--	671
39.MAFF238905_F.oxysporumSchlechtendalf.sp.radicis-lycopersici_Tomato	674	--	674
40.MAFF150004_F.oxysporumSchlechtendalf.sp.spinaciae_Spinach	671	--	671
41.MAFF247034_F.oxysporumSchlechtendal__Goldenchain	671	--	671
42.MAFF245747_F.oxysporumSchlechtendalf.sp.callistephi__Chinaaster	671	--	671
43.MAFF305115_FoxysporumSchlechtendalf.sp.batatas__Sweatpotato	703	--	703
44.MAFF150126_F.asiaticum_Seed	666	--	666
45.MAFF246738_F.solani_Angelica	703	--	703
46.MAFF246664_F.cugenangense_Perilla	671	--	671
47.MH582420.1F.solanistrainMRC256	679	--	679
48.MAFF240361_F.babinda_Soil	693	--	693
49.MAFF242368_F.azukicola_Azukibean	708	--	708
50.MAFF241312_F.asiaticum_Soil,welshonionfield	666	--	666
51.LT548416.1_F.culmorumpartialtef1a	646	--	646
52.MAFF150124_F.asiaticum__Wheat	666	--	666
53.MAFF238806_F.begoniae_Oncidiumsp	705	--	705
54.MW594399.1_FusariumincarnatumisolateUD01C	689	--	689
55.OP414923.1Pucciniagraminisf.sp.triticiisolateSHZPgt19	623	--	623
56.MT027094.1_BipolarisoryzaestrainOrL-2	895	--	895
57.ON734360.1_AlternariaalternataisolateH126	810	TGACATG--CGTCAGACCGTCGCG----------------	831
58.LC333578.1_StemphyliumlycopersiciSOasp2	0	--	0
59.HQ718583.1_Colletotrichumgloeosporioidesisolateq-1	567	--	567
60.JN241603.1_AtheliarolfsiiisolateSR1	1013	--	1013
61.KJ866474.1_RhizoctoniasolanistrainMHL-1	683	--	683
62.JQ672424.1AlternariatriticinaisolateEGS17-061	825	--	825

```
63.LT707559.1_P.capsicipartialtef1agene                                  964 ---------------------------------------- 964
64.MW090051.1_CurvularialunatastrainCls-3                                908 ---------------------------------------- 908
65.DQ400892.1_Aspergillusterreus                                         452 ---------------------------------------- 452
66.DQ911416.1_Pythiumsp.quercumstrainPy292                               932 ---------------------------------------- 932
67.EU797495.1_Phytophthorasp.oaksoilPoland                               930 ---------------------------------------- 930
68.HM148321.1_Cladosporiumcucumerinum                                    385 ---------------------------------------- 385
69.AF398888.1_SclerotiniasclerotiorumisolateSS1                            0 ---------------------------------------- 0
70.AF398888.1_S.sclerotiorumisolateSS1                                     0 ---------------------------------------- 0
71.HPAB545908.1_Verticilliumnonalfalfaeisolate                           442 ---------------------------------------- 442
72.EF433315.1_CeratocystisfimbriatavoucherCMW15052                       747 ---------------------------------------- 747
73.MN159912.1_Botrytiscinerea                                            931 ---------------------------------------- 931
74.MF034741.1_PeltasterfructicolaisolateSRB92                            251 CACCATGGACATGAGCTGACAGTGTTGATAGG-AAGCCGC 289
75.LC440360.1_CercosporaasparagiCOasp2                                   297 ---------------------------------------- 297
76.AY944105.1_MagnaportheoryzaeisolateSAG00T3()                          255 ---------------------------------------- 255
77.JX266586.1_CochliobolusmiyabeanusvoucherMFLUCC10-0733                 918 ---------------------------------------- 918
78.MN393253.1_CorynesporacassiicolaisolateQHD001(MN393253.1UNVERIFIED)     0 ---------------------------------------- 0
79.MF375218.1_AgroatheliarolfsiiisolateBJB24                             544 ---------------------------------------- 544
80.MN106270.1_AgroatheliarolfsiistrainJ-12                               509 ---------------------------------------- 509
81.OQ732628.1_AgroatheliarolfsiiisolateBTCBSr3                           499 ---------------------------------------- 499
82.KY196185.1_ColletotrichumtruncatumstrainPAK53                         468 ---------------------------------------- 468
83.GU935835.1_ColletotrichumcoccodesisolateC96002                       1041 ---------------------------------------- 1041
84.MK085963.1_AlternariatenuissimaisolateSCCZ06                            0 ---------------------------------------- 0
85.MT548042.1_AlternarialongipesstrainKY_2019_012                          0 ---------------------------------------- 0
86.MN356465.1CalonectriamontanaisolateHSP4                               489 ---------------------------------------- 489
87.OL694224.1_CalonectriacanadianastrainF099                             504 ---------------------------------------- 504
88.MK803351.1_NeoscytalidiumdimidiatumstrainKale4-C                      234 CGCGATCAGATGCTAACCGCCACCATAACAGG-AAGCCGC 272
89.ON376993.1_CurvulariachiangmaiensisisolateND00J7                      938 ---------------------------------------- 938
90.OQ383346.1_NeoscytalidiumdimidiatumisolateGKH-2                       209 CGCGATCAGATGCTAACCGCCACCATAACAGG-AAGCCGC 247
91.MF662595.1_NeoscytalidiumnovaehollandiaeisolateNeNo1                  228 CGCGATCAGATGCTAACCGCCACCATAACAGG-AAGCCGC 266
92.EF560588.1Melampsoralini                                              656 ---------------------------------------- 656
93.LC590862.1_NeoscytalidiumdimidiatumPSU-HP01TEF1                       202 CGCGATCAGATGCTAAC-GCCACCATAACAGG-AAGCCGC 239
94.KX278106.1_BotryosphaeriaqingyuanensisstrainCERC2947                  230 TGCGACCATATGCTAACCACCGCCACAACAGG-AAGCCGC 268
95.AJ578763.1_Blumeriagraminisf.sp.hordeicyp51                           298 TACGGAAATATCTTCACTTTCATATTACTGGGTAAGAAGA 337
96.MF490858.1_CurvulariadactylocteniicolastrainCPC28810                  930 ---------------------------------------- 930
97.KT287115.1_Bipolariscactivoraisolate3.8.6                             988 TGACATG--CGTCAGACCGTC------------------- 1006
98.MT560940.1_CurvulariacactivorastrainHLGH0118                          945 TGACATG--CGTCAAACTGGT------------------- 963
99.OM714565.1_CurvulariaplantarumstrainM0134                             971 TGACATG--CGTCAA------------------------- 983
100.MN159911.1_BotrytiscinereaSICAUCC19-0003                             931 ---------------------------------------- 931
102.GU294713.1LasiodiplodiatheobromaestrainUCD2430TX                     285 TACGAGCAAAGGCTAACCGCC-CCACTACAGGGAAGCCGC 323
103.KX868094.1_Mycosphaerellasp.isolateCRM20.1                           475 ---------------------------------------- 475
104.LC599478.1Pseudocercosporapini-densifloraeMUCC534                    249 GACCATG--TCACTGACGATATGCCTCACAGG-AAGCCGC 285
105.N584698.1BipolarissetariaestrainKBS4-2                               999 TGACATG--CGTCAGACCGTCGCTGTTGGTGT-CAT---- 1031

2.OM160859.1_F.buharicum                                                 560 ---------------------------------------- 560
1.LC727524.1_F.buharicum_OKI-1_Okura                                     682 ---------------------------------------- 682
3.KX302919.1_F.sublunatum                                                521 ---------------------------------------- 521
```

4.LT996094.1_F.convolutans	516	--	516
5.OM160861.1_F.abutilonis	630	--	630
6.OM160874.1_F.guadeloupense	560	--	560
7.MH392475.1_F.graminearum	430	--	430
8.MH582420.1_F.solani	540	--	540
9.MAFF244605_F.oxysporum	544	--	544
10.MAFF237278_F.contaminatum_Hylocereus	711	--	711
11.MAFF237649_F.concentricum__Riceroot	678	--	678
12.MAFF237650_F.concentricum__Wheat	679	--	679
13.MAFF239869_F.mangiferae__Ryukyupine	657	--	657
14.MAFF240460_F.fujikuroi_Passionfruit	661	--	661
15.MAFF241317_F.graminearum_Wheat	666	--	666
16.MAFF242670_F.ipomoeae_Wheat	672	--	672
17.MAFF245129_F.concentricum_Fraxinus	659	--	659
18.MAFF245395_F.cugenangense_Rhubarb	671	--	671
19.MAFF246637_F.nirenbergiae_Strawberry	671	--	671
20.MAFF246672_F.nirenbergiae_ChinesePeony	671	--	671
21.MAFF246697_F.commune_Urallicoriceroot	673	--	673
22.MAFF246729_F.falciforme_Angelica	691	--	691
23.MAFF247220_F.duplospermum__Euwallaceasp	694	--	694
24.MAFF410760_F.odoratissimum_alpha	669	--	669
25.MAFF244605_FusariumoxysporumSchlechtendal_MAFF244605_Tomato	672	--	672
26.MAFF241326_F.asiaticum_Wheat	669	--	669
27.MAFF245014_F.asiaticum_Wildsoybean	666	--	666
28.MAFF150124_F.asiaticum__Wheat	666	--	666
29.OM135603.1F.algeriense	699	--	699
30.MAFF237465_F.penzigii_Aloe	704	--	704
31.MAFF103054_F.oxysporumSchlechtendalf.sp.cucumerinum_Cucumber	671	--	671
32.MAFF712246_F.oxysporumSchlechtendalf.sp.dianthi__Carnation	673	--	673
33.MAFF305558_F.oxysporumSchlechtendalf.sp.fragariae__Watermelon	671	--	671
34.MAFF744087_F.oxysporumSchlechtendalf.sp.lactucae__Lettuce	671	--	671
35.MAFF726924_F.oxysporumSchlechtendalf.sp.lagenariae_Whitefloweredgourd	671	--	671
36.MAFF744003_F.oxysporumSchlechtendalf.sp.lagenariae_Squash)	672	--	672
37.MAFF305122_F.oxysporumSchlechtendalf.sp.melonis__Melon	671	--	671
38.MAFF306714_F.oxysporumSchlechtendalf.sp.momordicae_Balsampear	671	--	671
39.MAFF238905_F.oxysporumSchlechtendalf.sp.radicis-lycopersici_Tomato	674	--	674
40.MAFF150004_F.oxysporumSchlechtendalf.sp.spinaciae_Spinach	671	--	671
41.MAFF247034_F.oxysporumSchlechtendal__Goldenchain	671	--	671
42.MAFF245747_F.oxysporumSchlechtendalf.sp.callistephi__Chinaaster	671	--	671
43.MAFF305115_FoxysporumSchlechtendalf.sp.batatas__Sweatpotato	703	--	703
44.MAFF150126_F.asiaticum_Seed	666	--	666
45.MAFF246738_F.solani_Angelica	703	--	703
46.MAFF246664_F.cugenangense_Perilla	671	--	671
47.MH582420.1F.solanistrainMRC256	679	--	679
48.MAFF240361_F.babinda_Soil	693	--	693
49.MAFF242368_F.azukicola_Azukibean	708	--	708

```
50.MAFF241312_F.asiaticum_Soil,welshonionfield                         666 ---------------------------------------- 666
51.LT548416.1_F.culmorumpartialtef1a                                   646 ---------------------------------------- 646
52.MAFF150124_F.asiaticum__Wheat                                       666 ---------------------------------------- 666
53.MAFF238806_F.begoniae_Oncidiumsp                                    705 ---------------------------------------- 705
54.MW594399.1_FusariumincarnatumisolateUD01C                           689 ---------------------------------------- 689
55.OP414923.1Pucciniagraminisf.sp.triticiisolateSHZPgt19               623 ---------------------------------------- 623
56.MT027094.1_BipolarisoryzaestrainOrL-2                               895 ---------------------------------------- 895
57.ON734360.1_AlternariaalternataisolateH126                           831 ---------------------------------------- 831
58.LC333578.1_StemphyliumlycopersiciSOasp2                               1 -----------------------------TCGAGAAGTTC 11
59.HQ718583.1_Colletotrichumgloeosporioidesisolateq-1                  567 ---------------------------------------- 567
60.JN241603.1_AtheliarolfsiiisolateSR1                                1013 ---------------------------------------- 1013
61.KJ866474.1_RhizoctoniasolanistrainMHL-1                             683 ---------------------------------------- 683
62.JQ672424.1AlternariatriticinaisolateEGS17-061                       825 ---------------------------------------- 825
63.LT707559.1_P.capsicipartialtef1agene                                964 ---------------------------------------- 964
64.MW090051.1_CurvularialunatastrainCls-3                              908 ---------------------------------------- 908
65.DQ400892.1_Aspergillusterreus                                       452 ---------------------------------------- 452
66.DQ911416.1_Pythiumsp.quercumstrainPy292                             932 ---------------------------------------- 932
67.EU797495.1_Phytophthorasp.oaksoilPoland                             930 ---------------------------------------- 930
68.HM148321.1_Cladosporiumcucumerinum                                  385 ---------------------------------------- 385
69.AF398888.1_SclerotiniasclerotiorumisolateSS1                          1 ----------------------CATCGAGAAGTTCGAGAA 18
70.AF398888.1_S.sclerotiorumisolateSS1                                   1 ----------------------CATCGAGAAGTTCGAGAA 18
71.HPAB545908.1_Verticilliumnonalfalfaeisolate                         442 ---------------------------------------- 442
72.EF433315.1_CeratocystisfimbriatavoucherCMW15052                     747 ---------------------------------------- 747
73.MN159912.1_Botrytiscinerea                                          931 ---------------------------------------- 931
74.MF034741.1_PeltasterfructicolaisolateSRB92                          290 TGAGCTCGGTAAGGGTTCCTTCAAGTAA------------ 317
75.LC440360.1_CercosporaasparagiCOasp2                                 297 ---------------------------------------- 297
76.AY944105.1_MagnaportheoryzaeisolateSAG00T3()                        255 ---------------------------------------- 255
77.JX266586.1_CochliobolusmiyabeanusvoucherMFLUCC10-0733               918 ---------------------------------------- 918
78.MN393253.1_CorynesporacassiicolaisolateQHD001(MN393253.1UNVERIFIED)   0 ---------------------------------------- 0
79.MF375218.1_AgroatheliarolfsiiisolateBJB24                           544 ---------------------------------------- 544
80.MN106270.1_AgroatheliarolfsiistrainJ-12                             509 ---------------------------------------- 509
81.OQ732628.1_AgroatheliarolfsiiisolateBTCBSr3                         499 ---------------------------------------- 499
82.KY196185.1_ColletotrichumtruncatumstrainPAK53                       468 ---------------------------------------- 468
83.GU935835.1_ColletotrichumcoccodesisolateC96002                     1041 ---------------------------------------- 1041
84.MK085963.1_AlternariatenuissimaisolateSCCZ06                          0 ---------------------------------------- 0
85.MT548042.1_AlternarialongipesstrainKY_2019_012                        1 ---------------------------CATCGAGAAGTTC 13
86.MN356465.1CalonectriamontanaisolateHSP4                             489 ---------------------------------------- 489
87.OL694224.1_CalonectriacanadianastrainF099                           504 ---------------------------------------- 504
88.MK803351.1_NeoscytalidiumdimidiatumstrainKale4-C                    273 TGAACTCGGTAAGGGTTCCTTCAAGTA------------- 299
89.ON376993.1_CurvulariachiangmaiensisisolateND00J7                    938 ---------------------------------------- 938
90.OQ383346.1_NeoscytalidiumdimidiatumisolateGKH-2                     248 TGAACTCGGTAAGGGTTCCTTCAAGTAACTTCTTAAGTTC 287
91.MF662595.1_NeoscytalidiumnovaehollandiaeisolateNeNo1                267 TGAACTCGGTAAGGGTTC---------------------- 284
92.EF560588.1Melampsoralini                                            656 ---------------------------------------- 656
93.LC590862.1_NeoscytalidiumdimidiatumPSU-HP01TEF1                     240 TGAACTCGGTAAGGGTTCCTTCAAGTAA------------ 267
94.KX278106.1_BotryosphaeriaqingyuanensisstrainCERC2947                269 TGAGCTCGGCAAGGGT------------------------ 284
95.AJ578763.1_Blumeriagraminisf.sp.hordeicyp51                         338 CGACGGTATATCTAGGTCGACAGGGAAATAATTTTATTCT 377
```

```
96.MF490858.1_CurvulariadactylocteniicolastrainCPC28810              930 ---------------------------------------- 930
97.KT287115.1_Bipolariscactivoraisolate3.8.6                        1006 ---------------------------------------- 1006
98.MT560940.1_CurvulariacactivorastrainHLGH0118                      963 ---------------------------------------- 963
99.OM714565.1_CurvulariaplantarumstrainM0134                         983 ---------------------------------------- 983
100.MN159911.1_BotrytiscinereaSICAUCC19-0003                         931 ---------------------------------------- 931
102.GU294713.1LasiodiplodiatheobromaestrainUCD2430TX                 324 TGAGCTCGGTAAGGGTTCCTTCAAGTAACACCATTTTTTC 363
103.KX868094.1_Mycosphaerellasp.isolateCRM20.1                       475 ---------------------------------------- 475
104.LC599478.1Pseudocercosporapini-densifloraeMUCC534                286 CGAACTCGGTAAGGGTTCCTTCAAG--------------- 310
105.N584698.1BipolarissetariaestrainKBS4-2                          1031 ---------------------------------------- 1031

2.OM160859.1_F.buharicum                                             560 ---------------------------------------- 560
1.LC727524.1_F.buharicum_OKI-1_Okura                                 682 ---------------------------------------- 682
3.KX302919.1_F.sublunatum                                            521 ---------------------------------------- 521
4.LT996094.1_F.convolutans                                           516 ---------------------------------------- 516
5.OM160861.1_F.abutilonis                                            630 ---------------------------------------- 630
6.OM160874.1_F.guadeloupense                                         560 ---------------------------------------- 560
7.MH392475.1_F.graminearum                                           430 ---------------------------------------- 430
8.MH582420.1_F.solani                                                540 ---------------------------------------- 540
9.MAFF244605_F.oxysporum                                             544 ---------------------------------------- 544
10.MAFF237278_F.contaminatum_Hylocereus                              711 ---------------------------------------- 711
11.MAFF237649_F.concentricum__Riceroot                               678 ---------------------------------------- 678
12.MAFF237650_F.concentricum__Wheat                                  679 ---------------------------------------- 679
13.MAFF239869_F.mangiferae__Ryukyupine                               657 ---------------------------------------- 657
14.MAFF240460_F.fujikuroi_Passionfruit                               661 ---------------------------------------- 661
15.MAFF241317_F.graminearum_Wheat                                    666 ---------------------------------------- 666
16.MAFF242670_F.ipomoeae_Wheat                                       672 ---------------------------------------- 672
17.MAFF245129_F.concentricum_Fraxinus                                659 ---------------------------------------- 659
18.MAFF245395_F.cugenangense_Rhubarb                                 671 ---------------------------------------- 671
19.MAFF246637_F.nirenbergiae_Strawberry                              671 ---------------------------------------- 671
20.MAFF246672_F.nirenbergiae_ChinesePeony                            671 ---------------------------------------- 671
21.MAFF246697_F.commune_Urallicoriceroot                             673 ---------------------------------------- 673
22.MAFF246729_F.falciforme_Angelica                                  691 ---------------------------------------- 691
23.MAFF247220_F.duplospermum__Euwallaceasp                           694 ---------------------------------------- 694
24.MAFF410760_F.odoratissimum_alpha                                  669 ---------------------------------------- 669
25.MAFF244605_FusariumoxysporumSchlechtendal_MAFF244605_Tomato        672 ---------------------------------------- 672
26.MAFF241326_F.asiaticum_Wheat                                      669 ---------------------------------------- 669
27.MAFF245014_F.asiaticum_Wildsoybean                                666 ---------------------------------------- 666
28.MAFF150124_F.asiaticum__Wheat                                     666 ---------------------------------------- 666
29.OM135603.1F.algeriense                                            699 ---------------------------------------- 699
30.MAFF237465_F.penzigii_Aloe                                        704 ---------------------------------------- 704
31.MAFF103054_F.oxysporumSchlechtendalf.sp.cucumerinum_Cucumber      671 ---------------------------------------- 671
32.MAFF712246_F.oxysporumSchlechtendalf.sp.dianthi__Carnation        673 ---------------------------------------- 673
33.MAFF305558_F.oxysporumSchlechtendalf.sp.fragariae__Watermelon     671 ---------------------------------------- 671
34.MAFF744087_F.oxysporumSchlechtendalf.sp.lactucae__Lettuce         671 ---------------------------------------- 671
35.MAFF726924_F.oxysporumSchlechtendalf.sp.lagenariae_Whiteflorweredgourd 671 ---------------------------------------- 671
36.MAFF744003_F.oxysporumSchlechtendalf.sp.lagenariae_Squash)        672 ---------------------------------------- 672
```

```
37.MAFF305122_F.oxysporumSchlechtendalf.sp.melonis__Melon              671 ---------------------------------------- 671
38.MAFF306714_F.oxysporumSchlechtendalf.sp.momordicae_Balsampear       671 ---------------------------------------- 671
39.MAFF238905_F.oxysporumSchlechtendalf.sp.radicis-lycopersici_Tomato  674 ---------------------------------------- 674
40.MAFF150004_F.oxysporumSchlechtendalf.sp.spinaciae_Spinach           671 ---------------------------------------- 671
41.MAFF247034_F.oxysporumSchlechtendal__Goldenchain                    671 ---------------------------------------- 671
42.MAFF245747_F.oxysporumSchlechtendalf.sp.callistephi__Chinaaster     671 ---------------------------------------- 671
43.MAFF305115_FoxysporumSchlechtendalf.sp.batatas__Sweatpotato         703 ---------------------------------------- 703
44.MAFF150126_F.asiaticum_Seed                                         666 ---------------------------------------- 666
45.MAFF246738_F.solani_Angelica                                        703 ---------------------------------------- 703
46.MAFF246664_F.cugenangense_Perilla                                   671 ---------------------------------------- 671
47.MH582420.1F.solanistrainMRC256                                      679 ---------------------------------------- 679
48.MAFF240361_F.babinda_Soil                                           693 ---------------------------------------- 693
49.MAFF242368_F.azukicola_Azukibean                                    708 ---------------------------------------- 708
50.MAFF241312_F.asiaticum_Soil,welshonionfield                         666 ---------------------------------------- 666
51.LT548416.1_F.culmorumpartialtef1a                                   646 ---------------------------------------- 646
52.MAFF150124_F.asiaticum__Wheat                                       666 ---------------------------------------- 666
53.MAFF238806_F.begoniae_Oncidiumsp                                    705 ---------------------------------------- 705
54.MW594399.1_FusariumincarnatumisolateUD01C                           689 ---------------------------------------- 689
55.OP414923.1Pucciniagraminisf.sp.triticiisolateSHZPgt19               623 ---------------------------------------- 623
56.MT027094.1_BipolarisoryzaestrainOrL-2                               895 ---------------------------------------- 895
57.ON734360.1_AlternariaalternataisolateH126                           831 ---------------------------------------- 831
58.LC333578.1_StemphyliumlycopersiciSOasp2                              12 GAGAAGGTAGGGCATCACTTTTTTTACACCTGCAAAGTCG 51
59.HQ718583.1_Colletotrichumgloeosporioidesisolateq-1                  567 ---------------------------------------- 567
60.JN241603.1_AtheliarolfsiiisolateSR1                                1013 ---------------------------------------- 1013
61.KJ866474.1_RhizoctoniasolanistrainMHL-1                             683 ---------------------------------------- 683
62.JQ672424.1AlternariatriticinaisolateEGS17-061                       825 ---------------------------------------- 825
63.LT707559.1_P.capsicipartialtef1agene                                964 ---------------------------------------- 964
64.MW090051.1_CurvularialunatastrainCls-3                              908 ---------------------------------------- 908
65.DQ400892.1_Aspergillusterreus                                       452 ---------------------------------------- 452
66.DQ911416.1_Pythiumsp.quercumstrainPy292                             932 ---------------------------------------- 932
67.EU797495.1_Phytophthorasp.oaksoilPoland                             930 ---------------------------------------- 930
68.HM148321.1_Cladosporiumcucumerinum                                  385 ---------------------------------------- 385
69.AF398888.1_SclerotiniasclerotiorumisolateSS1                         19 GGTGAGATTTTCCCCTCTTATCCTACATTTTTTCTTTCGA 58
70.AF398888.1_S.sclerotiorumisolateSS1                                  19 GGTGAGATTTTCCCCTCTTATCCTACATTTTTTCTTTCGA 58
71.HPAB545908.1_Verticilliumnonalfalfaeisolate                         442 ---------------------------------------- 442
72.EF433315.1_CeratocystisfimbriatavoucherCMW15052                     747 ---------------------------------------- 747
73.MN159912.1_Botrytiscinerea                                          931 ---------------------------------------- 931
74.MF034741.1_PeltasterfructicolaisolateSRB92                          317 ---------------------------------------- 317
75.LC440360.1_CercosporaasparagiCOasp2                                 297 ---------------------------------------- 297
76.AY944105.1_MagnaportheoryzaeisolateSAG00T3()                        255 ---------------------------------------- 255
77.JX266586.1_CochliobolusmiyabeanusvoucherMFLUCC10-0733               918 ---------------------------------------- 918
78.MN393253.1_CorynesporacassiicolaisolateQHD001(MN393253.1UNVERIFIED)   1 ---GATTTTTCGCTTTCGACCGGCGATTCTGGGCCTGG-G 36
79.MF375218.1_AgroatheliarolfsiiisolateBJB24                           544 ---------------------------------------- 544
80.MN106270.1_AgroatheliarolfsiistrainJ-12                             509 ---------------------------------------- 509
81.OQ732628.1_AgroatheliarolfsiiisolateBTCBSr3                         499 ---------------------------------------- 499
82.KY196185.1_ColletotrichumtruncatumstrainPAK53                       468 ---------------------------------------- 468
```

```
83.GU935835.1_ColletotrichumcoccodesisolateC96002         1041 ---------------------------------------- 1041
84.MK085963.1_AlternariatenuissimaisolateSCCZ06              1 -AGAAGGTATGGCATCACTTTTCTTTCACGCGGCCTGTTG 39
85.MT548042.1_AlternarialongipesstrainKY_2019_012           14 GAGAAGGTATGGCATCACTTTTCTTTCACGCGGCCTGTTG 53
86.MN356465.1CalonectriamontanaisolateHSP4                 489 ---------------------------------------- 489
87.OL694224.1_CalonectriacanadianastrainF099              504 ---------------------------------------- 504
88.MK803351.1_NeoscytalidiumdimidiatumstrainKale4-C       299 ---------------------------------------- 299
89.ON376993.1_CurvulariachiangmaiensisisolateND00J7       938 ---------------------------------------- 938
90.OQ383346.1_NeoscytalidiumdimidiatumisolateGKH-2        288 TTGGTAATCT------------------------------ 297
91.MF662595.1_NeoscytalidiumnovaehollandiaeisolateNeNo1   284 ---------------------------------------- 284
92.EF560588.1Melampsoralini                               656 ---------------------------------------- 656
93.LC590862.1_NeoscytalidiumdimidiatumPSU-HP01TEF1        267 ---------------------------------------- 267
94.KX278106.1_BotryosphaeriaqingyuanensisstrainCERC2947   284 ---------------------------------------- 284
95.AJ578763.1_Blumeriagraminisf.sp.hordeicyp51            378 TAATGGAAAACTCAGAGACGTTAATGCCGAAGAAATTTAT 417
96.MF490858.1_CurvulariadactylocteniicolastrainCPC28810   930 ---------------------------------------- 930
97.KT287115.1_Bipolariscactivoraisolate3.8.6             1006 ---------------------------------------- 1006
98.MT560940.1_CurvulariacactivorastrainHLGH0118           963 ---------------------------------------- 963
99.OM714565.1_CurvulariaplantarumstrainM0134              983 ---------------------------------------- 983
100.MN159911.1_BotrytiscinereaSICAUCC19-0003              931 ---------------------------------------- 931
102.GU294713.1LasiodiplodiatheobromaestrainUCD2430TX      364 AAGATTCAGATGACTGACTTTCCTTACAGGAAGCTGCCGA 403
103.KX868094.1_Mycosphaerellasp.isolateCRM20.1            475 ---------------------------------------- 475
104.LC599478.1Pseudocercosporapini-densifloraeMUCC534     310 ---------------------------------------- 310
105.N584698.1BipolarissetariaestrainKBS4-2               1031 ---------------------------------------- 1031

2.OM160859.1_F.buharicum                                  560 ---------------------------------------- 560
1.LC727524.1_F.buharicum_OKI-1_Okura                      682 ---------------------------------------- 682
3.KX302919.1_F.sublunatum                                 521 ---------------------------------------- 521
4.LT996094.1_F.convolutans                                516 ---------------------------------------- 516
5.OM160861.1_F.abutilonis                                 630 ---------------------------------------- 630
6.OM160874.1_F.guadeloupense                              560 ---------------------------------------- 560
7.MH392475.1_F.graminearum                                430 ---------------------------------------- 430
8.MH582420.1_F.solani                                     540 ---------------------------------------- 540
9.MAFF244605_F.oxysporum                                  544 ---------------------------------------- 544
10.MAFF237278_F.contaminatum_Hylocereus                   711 ---------------------------------------- 711
11.MAFF237649_F.concentricum__Riceroot                    678 ---------------------------------------- 678
12.MAFF237650_F.concentricum__Wheat                       679 ---------------------------------------- 679
13.MAFF239869_F.mangiferae__Ryukyupine                    657 ---------------------------------------- 657
14.MAFF240460_F.fujikuroi_Passionfruit                    661 ---------------------------------------- 661
15.MAFF241317_F.graminearum_Wheat                         666 ---------------------------------------- 666
16.MAFF242670_F.ipomoeae_Wheat                            672 ---------------------------------------- 672
17.MAFF245129_F.concentricum_Fraxinus                     659 ---------------------------------------- 659
18.MAFF245395_F.cugenangense_Rhubarb                      671 ---------------------------------------- 671
19.MAFF246637_F.nirenbergiae_Strawberry                   671 ---------------------------------------- 671
20.MAFF246672_F.nirenbergiae_ChinesePeony                 671 ---------------------------------------- 671
21.MAFF246697_F.commune_Urallicoriceroot                  673 ---------------------------------------- 673
22.MAFF246729_F.falciforme_Angelica                       691 ---------------------------------------- 691
23.MAFF247220_F.duplospermum__Euwallaceasp                694 ---------------------------------------- 694
```

```
24.MAFF410760_F.odoratissimum_alpha                                 669 ---------------------------------------- 669
25.MAFF244605_FusariumoxysporumSchlechtendal_MAFF244605_Tomato      672 ---------------------------------------- 672
26.MAFF241326_F.asiaticum_Wheat                                     669 ---------------------------------------- 669
27.MAFF245014_F.asiaticum_Wildsoybean                               666 ---------------------------------------- 666
28.MAFF150124_F.asiaticum__Wheat                                    666 ---------------------------------------- 666
29.OM135603.1F.algeriense                                           699 ---------------------------------------- 699
30.MAFF237465_F.penzigii_Aloe                                       704 ---------------------------------------- 704
31.MAFF103054_F.oxysporumSchlechtendalf.sp.cucumerinum_Cucumber     671 ---------------------------------------- 671
32.MAFF712246_F.oxysporumSchlechtendalf.sp.dianthi__Carnation       673 ---------------------------------------- 673
33.MAFF305558_F.oxysporumSchlechtendalf.sp.fragariae__Watermelon    671 ---------------------------------------- 671
34.MAFF744087_F.oxysporumSchlechtendalf.sp.lactucae__Lettuce        671 ---------------------------------------- 671
35.MAFF726924_F.oxysporumSchlechtendalf.sp.lagenariae_Whiteflowered gourd 671 ---------------------------------------- 671
36.MAFF744003_F.oxysporumSchlechtendalf.sp.lagenariae_Squash)       672 ---------------------------------------- 672
37.MAFF305122_F.oxysporumSchlechtendalf.sp.melonis__Melon           671 ---------------------------------------- 671
38.MAFF306714_F.oxysporumSchlechtendalf.sp.momordicae_Balsampear    671 ---------------------------------------- 671
39.MAFF238905_F.oxysporumSchlechtendalf.sp.radicis-lycopersici_Tomato 674 ---------------------------------------- 674
40.MAFF150004_F.oxysporumSchlechtendalf.sp.spinaciae_Spinach        671 ---------------------------------------- 671
41.MAFF247034_F.oxysporumSchlechtendal__Goldenchain                 671 ---------------------------------------- 671
42.MAFF245747_F.oxysporumSchlechtendalf.sp.callistephi__Chinaaster  671 ---------------------------------------- 671
43.MAFF305115_FoxysporumSchlechtendalf.sp.batatas__Sweatpotato      703 ---------------------------------------- 703
44.MAFF150126_F.asiaticum_Seed                                      666 ---------------------------------------- 666
45.MAFF246738_F.solani_Angelica                                     703 ---------------------------------------- 703
46.MAFF246664_F.cugenangense_Perilla                                671 ---------------------------------------- 671
47.MH582420.1F.solanistrainMRC256                                   679 ---------------------------------------- 679
48.MAFF240361_F.babinda_Soil                                        693 ---------------------------------------- 693
49.MAFF242368_F.azukicola_Azukibean                                 708 ---------------------------------------- 708
50.MAFF241312_F.asiaticum_Soil,welshonionfield                      666 ---------------------------------------- 666
51.LT548416.1_F.culmorumpartialtef1a                                646 ---------------------------------------- 646
52.MAFF150124_F.asiaticum__Wheat                                    666 ---------------------------------------- 666
53.MAFF238806_F.begoniae_Oncidiumsp                                 705 ---------------------------------------- 705
54.MW594399.1_FusariumincarnatumisolateUD01C                        689 ---------------------------------------- 689
55.OP414923.1Pucciniagraminisf.sp.triticiisolateSHZPgt19            623 ---------------------------------------- 623
56.MT027094.1_BipolarisoryzaestrainOrL-2                            895 ---------------------------------------- 895
57.ON734360.1_AlternariaalternataisolateH126                        831 ---------------------------------------- 831
58.LC333578.1_StemphyliumlycopersiciSOasp2                           52 CTGCCACCTGGTGCATCTCCTGAGCGCGCAGCCAT-TTTC 90
59.HQ718583.1_Colletotrichumgloeosporioidesisolateq-1               567 ---------------------------------------- 567
60.JN241603.1_AtheliarolfsiiisolateSR1                             1013 ---------------------------------------- 1013
61.KJ866474.1_RhizoctoniasolanistrainMHL-1                          683 ---------------------------------------- 683
62.JQ672424.1AlternariatriticinaisolateEGS17-061                    825 ---------------------------------------- 825
63.LT707559.1_P.capsicipartialtef1agene                             964 ---------------------------------------- 964
64.MW090051.1_CurvularialunatastrainCls-3                           908 ---------------------------------------- 908
65.DQ400892.1_Aspergillusterreus                                    452 ---------------------------------------- 452
66.DQ911416.1_Pythiumsp.quercumstrainPy292                          932 ---------------------------------------- 932
67.EU797495.1_Phytophthorasp.oaksoilPoland                          930 ---------------------------------------- 930
68.HM148321.1_Cladosporiumcucumerinum                               385 ---------------------------------------- 385
69.AF398888.1_SclerotiniasclerotiorumisolateSS1                      59 CACAGTCTTTGTCCCTGTGCGGATTGAGTGCTCAATGCCT 98
```

```
70.AF398888.1_S.sclerotiorumisolateSS1                                  59 CACAGTCTTTGTCCCTGTGCGGATTGAGTGCTCAATGCCT 98
71.HPAB545908.1_Verticilliumnonalfalfaeisolate                         442 ---------------------------------------- 442
72.EF433315.1_CeratocystisfimbriatavoucherCMW15052                     747 ---------------------------------------- 747
73.MN159912.1_Botrytiscinerea                                          931 ---------------------------------------- 931
74.MF034741.1_PeltasterfructicolaisolateSRB92                          317 ---------------------------------------- 317
75.LC440360.1_CercosporaasparagiCOasp2                                 297 ---------------------------------------- 297
76.AY944105.1_MagnaportheoryzaeisolateSAG00T3()                        255 ---------------------------------------- 255
77.JX266586.1_CochliobolusmiyabeanusvoucherMFLUCC10-0733               918 ---------------------------------------- 918
78.MN393253.1_CorynesporacassiicolaisolateQHD001(MN393253.1UNVERIFIED)  37 CTGGCTTCCGGCGCGCCCGCTGTGCG--TAGCCGTTATTT 74
79.MF375218.1_AgroatheliarolfsiiisolateBJB24                           544 ---------------------------------------- 544
80.MN106270.1_AgroatheliarolfsiistrainJ-12                             509 ---------------------------------------- 509
81.OQ732628.1_AgroatheliarolfsiiisolateBTCBSr3                         499 ---------------------------------------- 499
82.KY196185.1_ColletotrichumtruncatumstrainPAK53                       468 ---------------------------------------- 468
83.GU935835.1_ColletotrichumcoccodesisolateC96002                     1041 ---------------------------------------- 1041
84.MK085963.1_AlternariatenuissimaisolateSCCZ06                         40 CGCCCACCCGGTGCTTTCTCTGAGCGCGTAGCCAA-AGCC 78
85.MT548042.1_AlternarialongipesstrainKY_2019_012                       54 CGCCCACCCGGTGCTTTCTCTGAGCGCGTAGCCAA-AGCC 92
86.MN356465.1CalonectriamontanaisolateHSP4                             489 ---------------------------------------- 489
87.OL694224.1_CalonectriacanadianastrainF099                           504 ---------------------------------------- 504
88.MK803351.1_NeoscytalidiumdimidiatumstrainKale4-C                    299 ---------------------------------------- 299
89.ON376993.1_CurvulariachiangmaiensisisolateND00J7                    938 ---------------------------------------- 938
90.OQ383346.1_NeoscytalidiumdimidiatumisolateGKH-2                     297 ---------------------------------------- 297
91.MF662595.1_NeoscytalidiumnovaehollandiaeisolateNeNo1                284 ---------------------------------------- 284
92.EF560588.1Melampsoralini                                            656 ---------------------------------------- 656
93.LC590862.1_NeoscytalidiumdimidiatumPSU-HP01TEF1                     267 ---------------------------------------- 267
94.KX278106.1_BotryosphaeriaqingyuanensisstrainCERC2947                284 ---------------------------------------- 284
95.AJ578763.1_Blumeriagraminisf.sp.hordeicyp51                         418 ACGGTCTTGACGACTCCTGTCTTCGGGACTGATGTAGTGT 457
96.MF490858.1_CurvulariadactylocteniicolastrainCPC28810                930 ---------------------------------------- 930
97.KT287115.1_Bipolariscactivoraisolate3.8.6                          1006 ---------------------------------------- 1006
98.MT560940.1_CurvulariacactivorastrainHLGH0118                        963 ---------------------------------------- 963
99.OM714565.1_CurvulariaplantarumstrainM0134                           983 ---------------------------------------- 983
100.MN159911.1_BotrytiscinereaSICAUCC19-0003                           931 ---------------------------------------- 931
102.GU294713.1LasiodiplodiatheobromaestrainUCD2430TX                   404 ACTT------------------------------------ 407
103.KX868094.1_Mycosphaerellasp.isolateCRM20.1                         475 ---------------------------------------- 475
104.LC599478.1Pseudocercosporapini-densifloraeMUCC534                  310 ---------------------------------------- 310
105.N584698.1BipolarissetariaestrainKBS4-2                            1031 ---------------------------------------- 1031

2.OM160859.1_F.buharicum                                               560 ---------------------------------------- 560
1.LC727524.1_F.buharicum_OKI-1_Okura                                   682 ---------------------------------------- 682
3.KX302919.1_F.sublunatum                                              521 ---------------------------------------- 521
4.LT996094.1_F.convolutans                                             516 ---------------------------------------- 516
5.OM160861.1_F.abutilonis                                              630 ---------------------------------------- 630
6.OM160874.1_F.guadeloupense                                           560 ---------------------------------------- 560
7.MH392475.1_F.graminearum                                             430 ---------------------------------------- 430
8.MH582420.1_F.solani                                                  540 ---------------------------------------- 540
9.MAFF244605_F.oxysporum                                               544 ---------------------------------------- 544
10.MAFF237278_F.contaminatum_Hylocereus                                711 ---------------------------------------- 711
```

```
11.MAFF237649_F.concentricum__Riceroot                                   678 ---------------------------------------- 678
12.MAFF237650_F.concentricum__Wheat                                      679 ---------------------------------------- 679
13.MAFF239869_F.mangiferae__Ryukyupine                                   657 ---------------------------------------- 657
14.MAFF240460_F.fujikuroi_Passionfruit                                   661 ---------------------------------------- 661
15.MAFF241317_F.graminearum_Wheat                                        666 ---------------------------------------- 666
16.MAFF242670_F.ipomoeae_Wheat                                           672 ---------------------------------------- 672
17.MAFF245129_F.concentricum_Fraxinus                                    659 ---------------------------------------- 659
18.MAFF245395_F.cugenangense_Rhubarb                                     671 ---------------------------------------- 671
19.MAFF246637_F.nirenbergiae_Strawberry                                  671 ---------------------------------------- 671
20.MAFF246672_F.nirenbergiae_ChinesePeony                                671 ---------------------------------------- 671
21.MAFF246697_F.commune_Urallicoriceroot                                 673 ---------------------------------------- 673
22.MAFF246729_F.falciforme_Angelica                                      691 ---------------------------------------- 691
23.MAFF247220_F.duplospermum__Euwallaceasp                               694 ---------------------------------------- 694
24.MAFF410760_F.odoratissimum_alpha                                      669 ---------------------------------------- 669
25.MAFF244605_FusariumoxysporumSchlechtendal_MAFF244605_Tomato           672 ---------------------------------------- 672
26.MAFF241326_F.asiaticum_Wheat                                          669 ---------------------------------------- 669
27.MAFF245014_F.asiaticum_Wildsoybean                                    666 ---------------------------------------- 666
28.MAFF150124_F.asiaticum__Wheat                                         666 ---------------------------------------- 666
29.OM135603.1F.algeriense                                                699 ---------------------------------------- 699
30.MAFF237465_F.penzigii_Aloe                                            704 ---------------------------------------- 704
31.MAFF103054_F.oxysporumSchlechtendalf.sp.cucumerinum_Cucumber          671 ---------------------------------------- 671
32.MAFF712246_F.oxysporumSchlechtendalf.sp.dianthi__Carnation            673 ---------------------------------------- 673
33.MAFF305558_F.oxysporumSchlechtendalf.sp.fragariae__Watermelon         671 ---------------------------------------- 671
34.MAFF744087_F.oxysporumSchlechtendalf.sp.lactucae__Lettuce             671 ---------------------------------------- 671
35.MAFF726924_F.oxysporumSchlechtendalf.sp.lagenariae_Whitefloweredgourd 671 ---------------------------------------- 671
36.MAFF744003_F.oxysporumSchlechtendalf.sp.lagenariae_Squash)            672 ---------------------------------------- 672
37.MAFF305122_F.oxysporumSchlechtendalf.sp.melonis__Melon                671 ---------------------------------------- 671
38.MAFF306714_F.oxysporumSchlechtendalf.sp.momordicae_Balsampear         671 ---------------------------------------- 671
39.MAFF238905_F.oxysporumSchlechtendalf.sp.radicis-lycopersici_Tomato    674 ---------------------------------------- 674
40.MAFF150004_F.oxysporumSchlechtendalf.sp.spinaciae_Spinach             671 ---------------------------------------- 671
41.MAFF247034_F.oxysporumSchlechtendal__Goldenchain                      671 ---------------------------------------- 671
42.MAFF245747_F.oxysporumSchlechtendalf.sp.callistephi__Chinaaster       671 ---------------------------------------- 671
43.MAFF305115_FoxysporumSchlechtendalf.sp.batatas__Sweatpotato           703 ---------------------------------------- 703
44.MAFF150126_F.asiaticum_Seed                                           666 ---------------------------------------- 666
45.MAFF246738_F.solani_Angelica                                          703 ---------------------------------------- 703
46.MAFF246664_F.cugenangense_Perilla                                     671 ---------------------------------------- 671
47.MH582420.1F.solanistrainMRC256                                        679 ---------------------------------------- 679
48.MAFF240361_F.babinda_Soil                                             693 ---------------------------------------- 693
49.MAFF242368_F.azukicola_Azukibean                                      708 ---------------------------------------- 708
50.MAFF241312_F.asiaticum_Soil,welshonionfield                           666 ---------------------------------------- 666
51.LT548416.1_F.culmorumpartialtef1a                                     646 ---------------------------------------- 646
52.MAFF150124_F.asiaticum__Wheat                                         666 ---------------------------------------- 666
53.MAFF238806_F.begoniae_Oncidiumsp                                      705 ---------------------------------------- 705
54.MW594399.1_FusariumincarnatumisolateUD01C                             689 ---------------------------------------- 689
55.OP414923.1Pucciniagraminisf.sp.triticiisolateSHZPgt19                 623 ---------------------------------------- 623
56.MT027094.1_BipolarisoryzaestrainOrL-2                                 895 ---------------------------------------- 895
```

57.ON734360.1_AlternariaalternataisolateH126	831	--	831
58.LC333578.1_StemphyliumlycopersiciSOasp2	91	TGGCTTATCGCGAAGAGGGGCATAAAATGGGTGGTGGGGT	130
59.HQ718583.1_Colletotrichumgloeosporioidesisolateq-1	567	--	567
60.JN241603.1_AtheliarolfsiiisolateSR1	1013	--	1013
61.KJ866474.1_RhizoctoniasolanistrainMHL-1	683	--	683
62.JQ672424.1AlternariatriticinaisolateEGS17-061	825	--	825
63.LT707559.1_P.capsicipartialtef1agene	964	--	964
64.MW090051.1_CurvularialunatastrainCls-3	908	--	908
65.DQ400892.1_Aspergillusterreus	452	--	452
66.DQ911416.1_Pythiumsp.quercumstrainPy292	932	--	932
67.EU797495.1_Phytophthorasp.oaksoilPoland	930	--	930
68.HM148321.1_Cladosporiumcucumerinum	385	--	385
69.AF398888.1_SclerotiniasclerotiorumisolateSS1	99	AATCTCATCGCGCTGTGCCCCTCGGTGGGGTAAAGATTTT	138
70.AF398888.1_S.sclerotiorumisolateSS1	99	AATCTCATCGCGCTGTGCCCCTCGGTGGGGTAAAGATTTT	138
71.HPAB545908.1_Verticilliumnonalfalfaeisolate	442	--	442
72.EF433315.1_CeratocystisfimbriatavoucherCMW15052	747	--	747
73.MN159912.1_Botrytiscinerea	931	--	931
74.MF034741.1_PeltasterfructicolaisolateSRB92	317	--	317
75.LC440360.1_CercosporaasparagiCOasp2	297	--	297
76.AY944105.1_MagnaportheoryzaeisolateSAG00T3()	255	--	255
77.JX266586.1_CochliobolusmiyabeanusvoucherMFLUCC10-0733	918	--	918
78.MN393253.1_CorynesporacassiicolaisolateQHD001(MN393253.1UNVERIFIED)	75	TGGCTTATCGCACTGAGGGGCAATTT--GGATGGTGGGGT	112
79.MF375218.1_AgroatheliarolfsiiisolateBJB24	544	--	544
80.MN106270.1_AgroatheliarolfsiistrainJ-12	509	--	509
81.OQ732628.1_AgroatheliarolfsiiisolateBTCBSr3	499	--	499
82.KY196185.1_ColletotrichumtruncatumstrainPAK53	468	--	468
83.GU935835.1_ColletotrichumcoccodesisolateC96002	1041	--	1041
84.MK085963.1_AlternariatenuissimaisolateSCCZ06	79	TGGCTTATCGCGATGAGGGGCATTTT-TGGGTGGTGGGGA	117
85.MT548042.1_AlternarialongipesstrainKY_2019_012	93	TGGCTTATCGCGATGAGGGGCATTTT-TGGGTGGTGGGGA	131
86.MN356465.1CalonectriamontanaisolateHSP4	489	--	489
87.OL694224.1_CalonectriacanadianastrainF099	504	--	504
88.MK803351.1_NeoscytalidiumdimidiatumstrainKale4-C	299	--	299
89.ON376993.1_CurvulariachiangmaiensisisolateND00J7	938	--	938
90.OQ383346.1_NeoscytalidiumdimidiatumisolateGKH-2	297	--	297
91.MF662595.1_NeoscytalidiumnovaehollandiaeisolateNeNo1	284	--	284
92.EF560588.1Melampsoralini	656	--	656
93.LC590862.1_NeoscytalidiumdimidiatumPSU-HP01TEF1	267	--	267
94.KX278106.1_BotryosphaeriaqingyuanensisstrainCERC2947	284	--	284
95.AJ578763.1_Blumeriagraminisf.sp.hordeicyp51	458	TTGACTGTCCTAATTCAAAATTAATGGAACAAAAGAAGGT	497
96.MF490858.1_CurvulariadactylocteniicolastrainCPC28810	930	--	930
97.KT287115.1_Bipolariscactivoraisolate3.8.6	1006	--	1006
98.MT560940.1_CurvulariacactivorastrainHLGH0118	963	--	963
99.OM714565.1_CurvulariaplantarumstrainM0134	983	--	983
100.MN159911.1_BotrytiscinereaSICAUCC19-0003	931	--	931
102.GU294713.1LasiodiplodiatheobromaestrainUCD2430TX	407	--	407
103.KX868094.1_Mycosphaerellasp.isolateCRM20.1	475	--	475

```
104.LC599478.1Pseudocercosporapini-densifloraeMUCC534              310 ---------------------------------------- 310
105.N584698.1BipolarissetariaestrainKBS4-2                        1031 ---------------------------------------- 1031

2.OM160859.1_F.buharicum                                           560 ---------------------------------------- 560
1.LC727524.1_F.buharicum_OKI-1_Okura                               682 ---------------------------------------- 682
3.KX302919.1_F.sublunatum                                          521 ---------------------------------------- 521
4.LT996094.1_F.convolutans                                         516 ---------------------------------------- 516
5.OM160861.1_F.abutilonis                                          630 ---------------------------------------- 630
6.OM160874.1_F.guadeloupense                                       560 ---------------------------------------- 560
7.MH392475.1_F.graminearum                                         430 ---------------------------------------- 430
8.MH582420.1_F.solani                                              540 ---------------------------------------- 540
9.MAFF244605_F.oxysporum                                           544 ---------------------------------------- 544
10.MAFF237278_F.contaminatum_Hylocereus                            711 ---------------------------------------- 711
11.MAFF237649_F.concentricum__Riceroot                             678 ---------------------------------------- 678
12.MAFF237650_F.concentricum__Wheat                                679 ---------------------------------------- 679
13.MAFF239869_F.mangiferae__Ryukyupine                             657 ---------------------------------------- 657
14.MAFF240460_F.fujikuroi_Passionfruit                             661 ---------------------------------------- 661
15.MAFF241317_F.graminearum_Wheat                                  666 ---------------------------------------- 666
16.MAFF242670_F.ipomoeae_Wheat                                     672 ---------------------------------------- 672
17.MAFF245129_F.concentricum_Fraxinus                              659 ---------------------------------------- 659
18.MAFF245395_F.cugenangense_Rhubarb                               671 ---------------------------------------- 671
19.MAFF246637_F.nirenbergiae_Strawberry                            671 ---------------------------------------- 671
20.MAFF246672_F.nirenbergiae_ChinesePeony                          671 ---------------------------------------- 671
21.MAFF246697_F.commune_Urallicoriceroot                           673 ---------------------------------------- 673
22.MAFF246729_F.falciforme_Angelica                                691 ---------------------------------------- 691
23.MAFF247220_F.duplospermum__Euwallaceasp                         694 ---------------------------------------- 694
24.MAFF410760_F.odoratissimum_alpha                                669 ---------------------------------------- 669
25.MAFF244605_FusariumoxysporumSchlechtendal_MAFF244605_Tomato     672 ---------------------------------------- 672
26.MAFF241326_F.asiaticum_Wheat                                    669 ---------------------------------------- 669
27.MAFF245014_F.asiaticum_Wildsoybean                              666 ---------------------------------------- 666
28.MAFF150124_F.asiaticum__Wheat                                   666 ---------------------------------------- 666
29.OM135603.1F.algeriense                                          699 ---------------------------------------- 699
30.MAFF237465_F.penzigii_Aloe                                      704 ---------------------------------------- 704
31.MAFF103054_F.oxysporumSchlechtendalf.sp.cucumerinum_Cucumber    671 ---------------------------------------- 671
32.MAFF712246_F.oxysporumSchlechtendalf.sp.dianthi__Carnation      673 ---------------------------------------- 673
33.MAFF305558_F.oxysporumSchlechtendalf.sp.fragariae__Watermelon   671 ---------------------------------------- 671
34.MAFF744087_F.oxysporumSchlechtendalf.sp.lactucae__Lettuce       671 ---------------------------------------- 671
35.MAFF726924_F.oxysporumSchlechtendalf.sp.lagenariae_Whitefloweredgourd 671 ---------------------------------------- 671
36.MAFF744003_F.oxysporumSchlechtendalf.sp.lagenariae_Squash)      672 ---------------------------------------- 672
37.MAFF305122_F.oxysporumSchlechtendalf.sp.melonis__Melon          671 ---------------------------------------- 671
38.MAFF306714_F.oxysporumSchlechtendalf.sp.momordicae_Balsampear   671 ---------------------------------------- 671
39.MAFF238905_F.oxysporumSchlechtendalf.sp.radicis-lycopersici_Tomato 674 ---------------------------------------- 674
40.MAFF150004_F.oxysporumSchlechtendalf.sp.spinaciae_Spinach       671 ---------------------------------------- 671
41.MAFF247034_F.oxysporumSchlechtendal__Goldenchain                671 ---------------------------------------- 671
42.MAFF245747_F.oxysporumSchlechtendalf.sp.callistephi__Chinaaster 671 ---------------------------------------- 671
43.MAFF305115_FoxysporumSchlechtendalf.sp.batatas__Sweatpotato     703 ---------------------------------------- 703
```

```
44.MAFF150126_F.asiaticum_Seed                                       666 ---------------------------------------- 666
45.MAFF246738_F.solani_Angelica                                      703 ---------------------------------------- 703
46.MAFF246664_F.cugenangense_Perilla                                 671 ---------------------------------------- 671
47.MH582420.1F.solanistrainMRC256                                    679 ---------------------------------------- 679
48.MAFF240361_F.babinda_Soil                                         693 ---------------------------------------- 693
49.MAFF242368_F.azukicola_Azukibean                                  708 ---------------------------------------- 708
50.MAFF241312_F.asiaticum_Soil,welshonionfield                       666 ---------------------------------------- 666
51.LT548416.1_F.culmorumpartialtef1a                                 646 ---------------------------------------- 646
52.MAFF150124_F.asiaticum__Wheat                                     666 ---------------------------------------- 666
53.MAFF238806_F.begoniae_Oncidiumsp                                  705 ---------------------------------------- 705
54.MW594399.1_FusariumincarnatumisolateUD01C                         689 ---------------------------------------- 689
55.OP414923.1Pucciniagraminisf.sp.triticiisolateSHZPgt19             623 ---------------------------------------- 623
56.MT027094.1_BipolarisoryzaestrainOrL-2                             895 ---------------------------------------- 895
57.ON734360.1_AlternariaalternataisolateH126                         831 ---------------------------------------- 831
58.LC333578.1_StemphyliumlycopersiciSOasp2                           131 TGTGCGAACTTTTACGCGCTAGCGCTAGTCCGCATGCGGC 170
59.HQ718583.1_Colletotrichumgloeosporioidesisolateq-1                567 ---------------------------------------- 567
60.JN241603.1_AtheliarolfsiiisolateSR1                              1013 ---------------------------------------- 1013
61.KJ866474.1_RhizoctoniasolanistrainMHL-1                           683 ---------------------------------------- 683
62.JQ672424.1AlternariatriticinaisolateEGS17-061                     825 ---------------------------------------- 825
63.LT707559.1_P.capsicipartialtef1agene                              964 ---------------------------------------- 964
64.MW090051.1_CurvularialunatastrainCls-3                            908 ---------------------------------------- 908
65.DQ400892.1_Aspergillusterreus                                     452 ---------------------------------------- 452
66.DQ911416.1_Pythiumsp.quercumstrainPy292                           932 ---------------------------------------- 932
67.EU797495.1_Phytophthorasp.oaksoilPoland                           930 ---------------------------------------- 930
68.HM148321.1_Cladosporiumcucumerinum                                385 ---------------------------------------- 385
69.AF398888.1_SclerotiniasclerotiorumisolateSS1                      139 TCTTGTCCCACCAGCAGAATTTTTTTGCGACCGGACATTT 178
70.AF398888.1_S.sclerotiorumisolateSS1                               139 TCTTGTCCCACCAGCAGAATTTTTTTGCGACCGGACATTT 178
71.HPAB545908.1_Verticilliumnonalfalfaeisolate                       442 ---------------------------------------- 442
72.EF433315.1_CeratocystisfimbriatavoucherCMW15052                   747 ---------------------------------------- 747
73.MN159912.1_Botrytiscinerea                                        931 ---------------------------------------- 931
74.MF034741.1_PeltasterfructicolaisolateSRB92                        317 ---------------------------------------- 317
75.LC440360.1_CercosporaasparagiCOasp2                               297 ---------------------------------------- 297
76.AY944105.1_MagnaportheoryzaeisolateSAG00T3()                      255 ---------------------------------------- 255
77.JX266586.1_CochliobolusmiyabeanusvoucherMFLUCC10-0733             918 ---------------------------------------- 918
78.MN393253.1_CorynesporacassiicolaisolateQHD001(MN393253.1UNVERIFIED) 113 TGTGCGAACTTTTACGCGCTAGCGCTAGTCCCGATCCGGA 152
79.MF375218.1_AgroatheliarolfsiiisolateBJB24                         544 ---------------------------------------- 544
80.MN106270.1_AgroatheliarolfsiistrainJ-12                           509 ---------------------------------------- 509
81.OQ732628.1_AgroatheliarolfsiiisolateBTCBSr3                       499 ---------------------------------------- 499
82.KY196185.1_ColletotrichumtruncatumstrainPAK53                     468 ---------------------------------------- 468
83.GU935835.1_ColletotrichumcoccodesisolateC96002                   1041 ---------------------------------------- 1041
84.MK085963.1_AlternariatenuissimaisolateSCCZ06                      118 TGTGCGAACTTTTACGCGCTAGCGCTAGTCCGCATGCGGC 157
85.MT548042.1_AlternarialongipesstrainKY_2019_012                    132 TGTGCGAACTTTTACGCGCTAGCGCTAGTCCGCATGCGGC 171
86.MN356465.1CalonectriamontanaisolateHSP4                           489 ---------------------------------------- 489
87.OL694224.1_CalonectriacanadianastrainF099                         504 ---------------------------------------- 504
88.MK803351.1_NeoscytalidiumdimidiatumstrainKale4-C                  299 ---------------------------------------- 299
89.ON376993.1_CurvulariachiangmaiensisisolateND00J7                  938 ---------------------------------------- 938
```

```
90.OQ383346.1_NeoscytalidiumdimidiatumisolateGKH-2                   297 ---------------------------------------- 297
91.MF662595.1_NeoscytalidiumnovaehollandiaeisolateNeNo1              284 ---------------------------------------- 284
92.EF560588.1Melampsoralini                                          656 ---------------------------------------- 656
93.LC590862.1_NeoscytalidiumdimidiatumPSU-HP01TEF1                   267 ---------------------------------------- 267
94.KX278106.1_BotryosphaeriaqingyuanensisstrainCERC2947              284 ---------------------------------------- 284
95.AJ578763.1_Blumeriagraminisf.sp.hordeicyp51                       498 TTTAAAATCATATGGATAACTTTCAGAATCAGACTCTGAT 537
96.MF490858.1_CurvulariadactylocteniicolastrainCPC28810              930 ---------------------------------------- 930
97.KT287115.1_Bipolariscactivoraisolate3.8.6                        1006 ---------------------------------------- 1006
98.MT560940.1_CurvulariacactivorastrainHLGH0118                      963 ---------------------------------------- 963
99.OM714565.1_CurvulariaplantarumstrainM0134                         983 ---------------------------------------- 983
100.MN159911.1_BotrytiscinereaSICAUCC19-0003                         931 ---------------------------------------- 931
102.GU294713.1LasiodiplodiatheobromaestrainUCD2430TX                 407 ---------------------------------------- 407
103.KX868094.1_Mycosphaerellasp.isolateCRM20.1                       475 ---------------------------------------- 475
104.LC599478.1Pseudocercosporapini-densifloraeMUCC534                310 ---------------------------------------- 310
105.N584698.1BipolarissetariaestrainKBS4-2                          1031 ---------------------------------------- 1031

2.OM160859.1_F.buharicum                                             560 ---------------------------------------- 560
1.LC727524.1_F.buharicum_OKI-1_Okura                                 682 ---------------------------------------- 682
3.KX302919.1_F.sublunatum                                            521 ---------------------------------------- 521
4.LT996094.1_F.convolutans                                           516 ---------------------------------------- 516
5.OM160861.1_F.abutilonis                                            630 ---------------------------------------- 630
6.OM160874.1_F.guadeloupense                                         560 ---------------------------------------- 560
7.MH392475.1_F.graminearum                                           430 ---------------------------------------- 430
8.MH582420.1_F.solani                                                540 ---------------------------------------- 540
9.MAFF244605_F.oxysporum                                             544 ---------------------------------------- 544
10.MAFF237278_F.contaminatum_Hylocereus                              711 ---------------------------------------- 711
11.MAFF237649_F.concentricum__Riceroot                               678 ---------------------------------------- 678
12.MAFF237650_F.concentricum__Wheat                                  679 ---------------------------------------- 679
13.MAFF239869_F.mangiferae__Ryukyupine                               657 ---------------------------------------- 657
14.MAFF240460_F.fujikuroi_Passionfruit                               661 ---------------------------------------- 661
15.MAFF241317_F.graminearum_Wheat                                    666 ---------------------------------------- 666
16.MAFF242670_F.ipomoeae_Wheat                                       672 ---------------------------------------- 672
17.MAFF245129_F.concentricum_Fraxinus                                659 ---------------------------------------- 659
18.MAFF245395_F.cugenangense_Rhubarb                                 671 ---------------------------------------- 671
19.MAFF246637_F.nirenbergiae_Strawberry                              671 ---------------------------------------- 671
20.MAFF246672_F.nirenbergiae_ChinesePeony                            671 ---------------------------------------- 671
21.MAFF246697_F.commune_Urallicoriceroot                             673 ---------------------------------------- 673
22.MAFF246729_F.falciforme_Angelica                                  691 ---------------------------------------- 691
23.MAFF247220_F.duplospermum__Euwallaceasp                           694 ---------------------------------------- 694
24.MAFF410760_F.odoratissimum_alpha                                  669 ---------------------------------------- 669
25.MAFF244605_FusariumoxysporumSchlechtendal_MAFF244605_Tomato       672 ---------------------------------------- 672
26.MAFF241326_F.asiaticum_Wheat                                      669 ---------------------------------------- 669
27.MAFF245014_F.asiaticum_Wildsoybean                                666 ---------------------------------------- 666
28.MAFF150124_F.asiaticum__Wheat                                     666 ---------------------------------------- 666
29.OM135603.1F.algeriense                                            699 ---------------------------------------- 699
30.MAFF237465_F.penzigii_Aloe                                        704 ---------------------------------------- 704
```

31.MAFF103054_F.oxysporumSchlechtendalf.sp.cucumerinum_Cucumber	671	--	671
32.MAFF712246_F.oxysporumSchlechtendalf.sp.dianthi__Carnation	673	--	673
33.MAFF305558_F.oxysporumSchlechtendalf.sp.fragariae__Watermelon	671	--	671
34.MAFF744087_F.oxysporumSchlechtendalf.sp.lactucae__Lettuce	671	--	671
35.MAFF726924_F.oxysporumSchlechtendalf.sp.lagenariae_Whitefloweredgourd	671	--	671
36.MAFF744003_F.oxysporumSchlechtendalf.sp.lagenariae_Squash)	672	--	672
37.MAFF305122_F.oxysporumSchlechtendalf.sp.melonis__Melon	671	--	671
38.MAFF306714_F.oxysporumSchlechtendalf.sp.momordicae_Balsampear	671	--	671
39.MAFF238905_F.oxysporumSchlechtendalf.sp.radicis-lycopersici_Tomato	674	--	674
40.MAFF150004_F.oxysporumSchlechtendalf.sp.spinaciae_Spinach	671	--	671
41.MAFF247034_F.oxysporumSchlechtendal__Goldenchain	671	--	671
42.MAFF245747_F.oxysporumSchlechtendalf.sp.callistephi__Chinaaster	671	--	671
43.MAFF305115_FoxysporumSchlechtendalf.sp.batatas__Sweatpotato	703	--	703
44.MAFF150126_F.asiaticum_Seed	666	--	666
45.MAFF246738_F.solani_Angelica	703	--	703
46.MAFF246664_F.cugenangense_Perilla	671	--	671
47.MH582420.1F.solanistrainMRC256	679	--	679
48.MAFF240361_F.babinda_Soil	693	--	693
49.MAFF242368_F.azukicola_Azukibean	708	--	708
50.MAFF241312_F.asiaticum_Soil,welshonionfield	666	--	666
51.LT548416.1_F.culmorumpartialtef1a	646	--	646
52.MAFF150124_F.asiaticum__Wheat	666	--	666
53.MAFF238806_F.begoniae_Oncidiumsp	705	--	705
54.MW594399.1_FusariumincarnatumisolateUD01C	689	--	689
55.OP414923.1Pucciniagraminisf.sp.triticiisolateSHZPgt19	623	--	623
56.MT027094.1_BipolarisoryzaestrainOrL-2	895	--	895
57.ON734360.1_AlternariaalternataisolateH126	831	--	831
58.LC333578.1_StemphyliumlycopersiciSOasp2	171	C-TTCGCGAACCACAACACCATGAC--ATATGCAAAATCC	207
59.HQ718583.1_Colletotrichumgloeosporioidesisolateq-1	567	--	567
60.JN241603.1_AtheliarolfsiiisolateSR1	1013	--	1013
61.KJ866474.1_RhizoctoniasolanistrainMHL-1	683	--	683
62.JQ672424.1AlternariatriticinaisolateEGS17-061	825	--	825
63.LT707559.1_P.capsicipartialtef1agene	964	--	964
64.MW090051.1_CurvularialunatastrainCls-3	908	--	908
65.DQ400892.1_Aspergillusterreus	452	--	452
66.DQ911416.1_Pythiumsp.quercumstrainPy292	932	--	932
67.EU797495.1_Phytophthorasp.oaksoilPoland	930	--	930
68.HM148321.1_Cladosporiumcucumerinum	385	--	385
69.AF398888.1_SclerotiniasclerotiorumisolateSS1	179	ATTTTGTATGACTATAAATTTGTCCGGGTGCATCGTGCCG	218
70.AF398888.1_S.sclerotiorumisolateSS1	179	ATTTTGTATGACTATAAATTTGTCCGGGTGCATCGTGCCG	218
71.HPAB545908.1_Verticilliumnonalfalfaeisolate	442	--	442
72.EF433315.1_CeratocystisfimbriatavoucherCMW15052	747	--	747
73.MN159912.1_Botrytiscinerea	931	--	931
74.MF034741.1_PeltasterfructicolaisolateSRB92	317	--	317
75.LC440360.1_CercosporaasparagiCOasp2	297	--	297
76.AY944105.1_MagnaportheoryzaeisolateSAG00T3()	255	--	255

```
77.JX266586.1_CochliobolusmiyabeanusvoucherMFLUCC10-0733              918 ---------------------------------------- 918
78.MN393253.1_CorynesporacassiicolaisolateQHD001(MN393253.1UNVERIFIED) 153 CGTTCGCCAACTC-AACACCATGACGCACATCCAATTTTG 191
79.MF375218.1_AgroatheliarolfsiiisolateBJB24                          544 ---------------------------------------- 544
80.MN106270.1_AgroatheliarolfsiistrainJ-12                            509 ---------------------------------------- 509
81.OQ732628.1_AgroatheliarolfsiiisolateBTCBSr3                        499 ---------------------------------------- 499
82.KY196185.1_ColletotrichumtruncatumstrainPAK53                      468 ---------------------------------------- 468
83.GU935835.1_ColletotrichumcoccodesisolateC96002                    1041 ---------------------------------------- 1041
84.MK085963.1_AlternariatenuissimaisolateSCCZ06                       158 C-TTCGCGAACTCCAACGCAATGACGCACATGTAATTTCC 196
85.MT548042.1_AlternarialongipesstrainKY_2019_012                     172 C-TTCGCGAACTCCAACGCAATGACGCACATGTAATTTCC 210
86.MN356465.1CalonectriamontanaisolateHSP4                            489 ---------------------------------------- 489
87.OL694224.1_CalonectriacanadianastrainF099                          504 ---------------------------------------- 504
88.MK803351.1_NeoscytalidiumdimidiatumstrainKale4-C                   299 ---------------------------------------- 299
89.ON376993.1_CurvulariachiangmaiensisisolateND00J7                   938 ---------------------------------------- 938
90.OQ383346.1_NeoscytalidiumdimidiatumisolateGKH-2                    297 ---------------------------------------- 297
91.MF662595.1_NeoscytalidiumnovaehollandiaeisolateNeNo1               284 ---------------------------------------- 284
92.EF560588.1Melampsoralini                                           656 ---------------------------------------- 656
93.LC590862.1_NeoscytalidiumdimidiatumPSU-HP01TEF1                    267 ---------------------------------------- 267
94.KX278106.1_BotryosphaeriaqingyuanensisstrainCERC2947               284 ---------------------------------------- 284
95.AJ578763.1_Blumeriagraminisf.sp.hordeicyp51                        538 ATTTGCACAGTTCATGAAAGCAGCCCTTACGACTGAGGCC 577
96.MF490858.1_CurvulariadactylocteniicolastrainCPC28810               930 ---------------------------------------- 930
97.KT287115.1_Bipolariscactivoraisolate3.8.6                         1006 ---------------------------------------- 1006
98.MT560940.1_CurvulariacactivorastrainHLGH0118                       963 ---------------------------------------- 963
99.OM714565.1_CurvulariaplantarumstrainM0134                          983 ---------------------------------------- 983
100.MN159911.1_BotrytiscinereaSICAUCC19-0003                          931 ---------------------------------------- 931
102.GU294713.1LasiodiplodiatheobromaestrainUCD2430TX                  407 ---------------------------------------- 407
103.KX868094.1_Mycosphaerellasp.isolateCRM20.1                        475 ---------------------------------------- 475
104.LC599478.1Pseudocercosporapini-densifloraeMUCC534                 310 ---------------------------------------- 310
105.N584698.1BipolarissetariaestrainKBS4-2                           1031 ---------------------------------------- 1031

2.OM160859.1_F.buharicum                                              560 ---------------------------------------- 560
1.LC727524.1_F.buharicum_OKI-1_Okura                                  682 ---------------------------------------- 682
3.KX302919.1_F.sublunatum                                             521 ---------------------------------------- 521
4.LT996094.1_F.convolutans                                            516 ---------------------------------------- 516
5.OM160861.1_F.abutilonis                                             630 ---------------------------------------- 630
6.OM160874.1_F.guadeloupense                                          560 ---------------------------------------- 560
7.MH392475.1_F.graminearum                                            430 ---------------------------------------- 430
8.MH582420.1_F.solani                                                 540 ---------------------------------------- 540
9.MAFF244605_F.oxysporum                                              544 ---------------------------------------- 544
10.MAFF237278_F.contaminatum_Hylocereus                               711 ---------------------------------------- 711
11.MAFF237649_F.concentricum__Riceroot                                678 ---------------------------------------- 678
12.MAFF237650_F.concentricum__Wheat                                   679 ---------------------------------------- 679
13.MAFF239869_F.mangiferae__Ryukyupine                                657 ---------------------------------------- 657
14.MAFF240460_F.fujikuroi_Passionfruit                                661 ---------------------------------------- 661
15.MAFF241317_F.graminearum_Wheat                                     666 ---------------------------------------- 666
16.MAFF242670_F.ipomoeae_Wheat                                        672 ---------------------------------------- 672
17.MAFF245129_F.concentricum_Fraxinus                                 659 ---------------------------------------- 659
```

18.MAFF245395_F.cugenangense_Rhubarb	671	--	671
19.MAFF246637_F.nirenbergiae_Strawberry	671	--	671
20.MAFF246672_F.nirenbergiae_ChinesePeony	671	--	671
21.MAFF246697_F.commune_Urallicoriceroot	673	--	673
22.MAFF246729_F.falciforme_Angelica	691	--	691
23.MAFF247220_F.duplospermum__Euwallaceasp	694	--	694
24.MAFF410760_F.odoratissimum_alpha	669	--	669
25.MAFF244605_FusariumoxysporumSchlechtendal_MAFF244605_Tomato	672	--	672
26.MAFF241326_F.asiaticum_Wheat	669	--	669
27.MAFF245014_F.asiaticum_Wildsoybean	666	--	666
28.MAFF150124_F.asiaticum__Wheat	666	--	666
29.OM135603.1F.algeriense	699	--	699
30.MAFF237465_F.penzigii_Aloe	704	--	704
31.MAFF103054_F.oxysporumSchlechtendalf.sp.cucumerinum_Cucumber	671	--	671
32.MAFF712246_F.oxysporumSchlechtendalf.sp.dianthi__Carnation	673	--	673
33.MAFF305558_F.oxysporumSchlechtendalf.sp.fragariae__Watermelon	671	--	671
34.MAFF744087_F.oxysporumSchlechtendalf.sp.lactucae__Lettuce	671	--	671
35.MAFF726924_F.oxysporumSchlechtendalf.sp.lagenariae_Whitefloweredgourd	671	--	671
36.MAFF744003_F.oxysporumSchlechtendalf.sp.lagenariae_Squash)	672	--	672
37.MAFF305122_F.oxysporumSchlechtendalf.sp.melonis__Melon	671	--	671
38.MAFF306714_F.oxysporumSchlechtendalf.sp.momordicae_Balsampear	671	--	671
39.MAFF238905_F.oxysporumSchlechtendalf.sp.radicis-lycopersici_Tomato	674	--	674
40.MAFF150004_F.oxysporumSchlechtendalf.sp.spinaciae_Spinach	671	--	671
41.MAFF247034_F.oxysporumSchlechtendal__Goldenchain	671	--	671
42.MAFF245747_F.oxysporumSchlechtendalf.sp.callistephi__Chinaaster	671	--	671
43.MAFF305115_FoxysporumSchlechtendalf.sp.batatas__Sweatpotato	703	--	703
44.MAFF150126_F.asiaticum_Seed	666	--	666
45.MAFF246738_F.solani_Angelica	703	--	703
46.MAFF246664_F.cugenangense_Perilla	671	--	671
47.MH582420.1F.solanistrainMRC256	679	--	679
48.MAFF240361_F.babinda_Soil	693	--	693
49.MAFF242368_F.azukicola_Azukibean	708	--	708
50.MAFF241312_F.asiaticum_Soil,welshonionfield	666	--	666
51.LT548416.1_F.culmorumpartialtef1a	646	--	646
52.MAFF150124_F.asiaticum__Wheat	666	--	666
53.MAFF238806_F.begoniae_Oncidiumsp	705	--	705
54.MW594399.1_FusariumincarnatumisolateUD01C	689	--	689
55.OP414923.1Pucciniagraminisf.sp.triticiisolateSHZPgt19	623	--	623
56.MT027094.1_BipolarisoryzaestrainOrL-2	895	--	895
57.ON734360.1_AlternariaalternataisolateH126	831	--	831
58.LC333578.1_StemphyliumlycopersiciSOasp2	208	CATCTTCGGCTGCACGGTGCTAACAAGCCTCACAGGAAGC	247
59.HQ718583.1_Colletotrichumgloeosporioidesisolateq-1	567	--	567
60.JN241603.1_AtheliarolfsiiisolateSR1	1013	--	1013
61.KJ866474.1_RhizoctoniasolanistrainMHL-1	683	--	683
62.JQ672424.1AlternariatriticinaisolateEGS17-061	825	--	825
63.LT707559.1_P.capsicipartialtef1agene	964	--	964

```
64.MW090051.1_CurvularialunatastrainCls-3                            908 ---------------------------------------- 908
65.DQ400892.1_Aspergillusterreus                                     452 ---------------------------------------- 452
66.DQ911416.1_Pythiumsp.quercumstrainPy292                           932 ---------------------------------------- 932
67.EU797495.1_Phytophthorasp.oaksoilPoland                           930 ---------------------------------------- 930
68.HM148321.1_Cladosporiumcucumerinum                                385 ---------------------------------------- 385
69.AF398888.1_SclerotiniasclerotiorumisolateSS1                      219 CACGTCAAATTATCGGCTTCACATCACCAACTGCGATCAA 258
70.AF398888.1_S.sclerotiorumisolateSS1                               219 CACGTCAAATTATCGGCTTCACATCACCAACTGCGATCAA 258
71.HPAB545908.1_Verticilliumnonalfalfaeisolate                       442 ---------------------------------------- 442
72.EF433315.1_CeratocystisfimbriatavoucherCMW15052                   747 ---------------------------------------- 747
73.MN159912.1_Botrytiscinerea                                        931 ---------------------------------------- 931
74.MF034741.1_PeltasterfructicolaisolateSRB92                        317 ---------------------------------------- 317
75.LC440360.1_CercosporaasparagiCOasp2                               297 ---------------------------------------- 297
76.AY944105.1_MagnaportheoryzaeisolateSAG00T3()                      255 ---------------------------------------- 255
77.JX266586.1_CochliobolusmiyabeanusvoucherMFLUCC10-0733             918 ---------------------------------------- 918
78.MN393253.1_CorynesporacassiicolaisolateQHD001(MN393253.1UNVERIFIED) 192 CATCGCCAGCCGGCCCCCGCCCAGAACGCCGAGTTTTCTT 231
79.MF375218.1_AgroatheliarolfsiiisolateBJB24                         544 ---------------------------------------- 544
80.MN106270.1_AgroatheliarolfsiistrainJ-12                           509 ---------------------------------------- 509
81.OQ732628.1_AgroatheliarolfsiiisolateBTCBSr3                       499 ---------------------------------------- 499
82.KY196185.1_ColletotrichumtruncatumstrainPAK53                     468 ---------------------------------------- 468
83.GU935835.1_ColletotrichumcoccodesisolateC96002                   1041 ---------------------------------------- 1041
84.MK085963.1_AlternariatenuissimaisolateSCCZ06                      197 CCATT----------------------------------- 201
85.MT548042.1_AlternarialongipesstrainKY_2019_012                    211 CCATT----------------------------------- 215
86.MN356465.1CalonectriamontanaisolateHSP4                           489 ---------------------------------------- 489
87.OL694224.1_CalonectriacanadianastrainF099                         504 ---------------------------------------- 504
88.MK803351.1_NeoscytalidiumdimidiatumstrainKale4-C                  299 ---------------------------------------- 299
89.ON376993.1_CurvulariachiangmaiensisisolateND00J7                  938 ---------------------------------------- 938
90.OQ383346.1_NeoscytalidiumdimidiatumisolateGKH-2                   297 ---------------------------------------- 297
91.MF662595.1_NeoscytalidiumnovaehollandiaeisolateNeNo1              284 ---------------------------------------- 284
92.EF560588.1Melampsoralini                                          656 ---------------------------------------- 656
93.LC590862.1_NeoscytalidiumdimidiatumPSU-HP01TEF1                   267 ---------------------------------------- 267
94.KX278106.1_BotryosphaeriaqingyuanensisstrainCERC2947              284 ---------------------------------------- 284
95.AJ578763.1_Blumeriagraminisf.sp.hordeicyp51                       578 TTCCGCTCTTATGTACCTATCATCCAAAATGAAGTGAAAA 617
96.MF490858.1_CurvulariadactylocteniicolastrainCPC28810              930 ---------------------------------------- 930
97.KT287115.1_Bipolariscactivoraisolate3.8.6                        1006 ---------------------------------------- 1006
98.MT560940.1_CurvulariacactivorastrainHLGH0118                      963 ---------------------------------------- 963
99.OM714565.1_CurvulariaplantarumstrainM0134                         983 ---------------------------------------- 983
100.MN159911.1_BotrytiscinereaSICAUCC19-0003                         931 ---------------------------------------- 931
102.GU294713.1LasiodiplodiatheobromaestrainUCD2430TX                 407 ---------------------------------------- 407
103.KX868094.1_Mycosphaerellasp.isolateCRM20.1                       475 ---------------------------------------- 475
104.LC599478.1Pseudocercosporapini-densifloraeMUCC534                310 ---------------------------------------- 310
105.N584698.1BipolarissetariaestrainKBS4-2                          1031 ---------------------------------------- 1031

2.OM160859.1_F.buharicum                                             560 ---------------------------------------- 560
1.LC727524.1_F.buharicum_OKI-1_Okura                                 682 ---------------------------------------- 682
3.KX302919.1_F.sublunatum                                            521 ---------------------------------------- 521
4.LT996094.1_F.convolutans                                           516 ---------------------------------------- 516
```

```
5.OM160861.1_F.abutilonis                                            630 ---------------------------------------- 630
6.OM160874.1_F.guadeloupense                                         560 ---------------------------------------- 560
7.MH392475.1_F.graminearum                                           430 ---------------------------------------- 430
8.MH582420.1_F.solani                                                540 ---------------------------------------- 540
9.MAFF244605_F.oxysporum                                             544 ---------------------------------------- 544
10.MAFF237278_F.contaminatum_Hylocereus                              711 ---------------------------------------- 711
11.MAFF237649_F.concentricum__Riceroot                               678 ---------------------------------------- 678
12.MAFF237650_F.concentricum__Wheat                                  679 ---------------------------------------- 679
13.MAFF239869_F.mangiferae__Ryukyupine                               657 ---------------------------------------- 657
14.MAFF240460_F.fujikuroi_Passionfruit                               661 ---------------------------------------- 661
15.MAFF241317_F.graminearum_Wheat                                    666 ---------------------------------------- 666
16.MAFF242670_F.ipomoeae_Wheat                                       672 ---------------------------------------- 672
17.MAFF245129_F.concentricum_Fraxinus                                659 ---------------------------------------- 659
18.MAFF245395_F.cugenangense_Rhubarb                                 671 ---------------------------------------- 671
19.MAFF246637_F.nirenbergiae_Strawberry                              671 ---------------------------------------- 671
20.MAFF246672_F.nirenbergiae_ChinesePeony                            671 ---------------------------------------- 671
21.MAFF246697_F.commune_Urallicoriceroot                             673 ---------------------------------------- 673
22.MAFF246729_F.falciforme_Angelica                                  691 ---------------------------------------- 691
23.MAFF247220_F.duplospermum__Euwallaceasp                           694 ---------------------------------------- 694
24.MAFF410760_F.odoratissimum_alpha                                  669 ---------------------------------------- 669
25.MAFF244605_FusariumoxysporumSchlechtendal_MAFF244605_Tomato       672 ---------------------------------------- 672
26.MAFF241326_F.asiaticum_Wheat                                      669 ---------------------------------------- 669
27.MAFF245014_F.asiaticum_Wildsoybean                                666 ---------------------------------------- 666
28.MAFF150124_F.asiaticum__Wheat                                     666 ---------------------------------------- 666
29.OM135603.1F.algeriense                                            699 ---------------------------------------- 699
30.MAFF237465_F.penzigii_Aloe                                        704 ---------------------------------------- 704
31.MAFF103054_F.oxysporumSchlechtendalf.sp.cucumerinum_Cucumber      671 ---------------------------------------- 671
32.MAFF712246_F.oxysporumSchlechtendalf.sp.dianthi__Carnation        673 ---------------------------------------- 673
33.MAFF305558_F.oxysporumSchlechtendalf.sp.fragariae__Watermelon     671 ---------------------------------------- 671
34.MAFF744087_F.oxysporumSchlechtendalf.sp.lactucae__Lettuce         671 ---------------------------------------- 671
35.MAFF726924_F.oxysporumSchlechtendalf.sp.lagenariae_Whitefloweredgourd 671 ---------------------------------------- 671
36.MAFF744003_F.oxysporumSchlechtendalf.sp.lagenariae_Squash)        672 ---------------------------------------- 672
37.MAFF305122_F.oxysporumSchlechtendalf.sp.melonis__Melon            671 ---------------------------------------- 671
38.MAFF306714_F.oxysporumSchlechtendalf.sp.momordicae_Balsampear     671 ---------------------------------------- 671
39.MAFF238905_F.oxysporumSchlechtendalf.sp.radicis-lycopersici_Tomato 674 ---------------------------------------- 674
40.MAFF150004_F.oxysporumSchlechtendalf.sp.spinaciae_Spinach         671 ---------------------------------------- 671
41.MAFF247034_F.oxysporumSchlechtendal__Goldenchain                  671 ---------------------------------------- 671
42.MAFF245747_F.oxysporumSchlechtendalf.sp.callistephi__Chinaaster   671 ---------------------------------------- 671
43.MAFF305115_FoxysporumSchlechtendalf.sp.batatas__Sweatpotato       703 ---------------------------------------- 703
44.MAFF150126_F.asiaticum_Seed                                       666 ---------------------------------------- 666
45.MAFF246738_F.solani_Angelica                                      703 ---------------------------------------- 703
46.MAFF246664_F.cugenangense_Perilla                                 671 ---------------------------------------- 671
47.MH582420.1F.solanistrainMRC256                                    679 ---------------------------------------- 679
48.MAFF240361_F.babinda_Soil                                         693 ---------------------------------------- 693
49.MAFF242368_F.azukicola_Azukibean                                  708 ---------------------------------------- 708
50.MAFF241312_F.asiaticum_Soil,welshonionfield                       666 ---------------------------------------- 666
```

51.LT548416.1_F.culmorumpartialtef1a	646	--	646
52.MAFF150124_F.asiaticum__Wheat	666	--	666
53.MAFF238806_F.begoniae_Oncidiumsp	705	--	705
54.MW594399.1_FusariumincarnatumisolateUD01C	689	--	689
55.OP414923.1Pucciniagraminisf.sp.triticiisolateSHZPgt19	623	--	623
56.MT027094.1_BipolarisoryzaestrainOrL-2	895	--	895
57.ON734360.1_AlternariaalternataisolateH126	831	--	831
58.LC333578.1_StemphyliumlycopersiciSOasp2	248	CGCCGAGCTCGGTAAGGG----------------------	265
59.HQ718583.1_Colletotrichumgloeosporioidesisolateq-1	567	--	567
60.JN241603.1_AtheliarolfsiiisolateSR1	1013	--	1013
61.KJ866474.1_RhizoctoniasolanistrainMHL-1	683	--	683
62.JQ672424.1AlternariatriticinaisolateEGS17-061	825	--	825
63.LT707559.1_P.capsicipartialtef1agene	964	--	964
64.MW090051.1_CurvularialunatastrainCls-3	908	--	908
65.DQ400892.1_Aspergillusterreus	452	--	452
66.DQ911416.1_Pythiumsp.quercumstrainPy292	932	--	932
67.EU797495.1_Phytophthorasp.oaksoilPoland	930	--	930
68.HM148321.1_Cladosporiumcucumerinum	385	--	385
69.AF398888.1_SclerotiniasclerotiorumisolateSS1	259	TACCAACTATCTTTTACAATTGTCACTGACAATCATTATA	298
70.AF398888.1_S.sclerotiorumisolateSS1	259	TACCAACTATCTTTTACAATTGTCACTGACAATCATTATA	298
71.HPAB545908.1_Verticilliumnonalfalfaeisolate	442	--	442
72.EF433315.1_CeratocystisfimbriatavoucherCMW15052	747	--	747
73.MN159912.1_Botrytiscinerea	931	--	931
74.MF034741.1_PeltasterfructicolaisolateSRB92	317	--	317
75.LC440360.1_CercosporaasparagiCOasp2	297	--	297
76.AY944105.1_MagnaportheoryzaeisolateSAG00T3()	255	--	255
77.JX266586.1_CochliobolusmiyabeanusvoucherMFLUCC10-0733	918	--	918
78.MN393253.1_CorynesporacassiicolaisolateQHD001(MN393253.1UNVERIFIED)	232	TTCTTGCCTGCCTGACAACAATG--CTAACGACCCTCACA	269
79.MF375218.1_AgroatheliarolfsiiisolateBJB24	544	--	544
80.MN106270.1_AgroatheliarolfsiistrainJ-12	509	--	509
81.OQ732628.1_AgroatheliarolfsiiisolateBTCBSr3	499	--	499
82.KY196185.1_ColletotrichumtruncatumstrainPAK53	468	--	468
83.GU935835.1_ColletotrichumcoccodesisolateC96002	1041	--	1041
84.MK085963.1_AlternariatenuissimaisolateSCCZ06	202	-----------CTGGCCACAGCGAGCTAACAAGCCTCACA	230
85.MT548042.1_AlternarialongipesstrainKY_2019_012	216	-----------CTGGCCACAGCGAGCTAACAAGCCTCACA	244
86.MN356465.1CalonectriamontanaisolateHSP4	489	--	489
87.OL694224.1_CalonectriacanadianastrainF099	504	--	504
88.MK803351.1_NeoscytalidiumdimidiatumstrainKale4-C	299	--	299
89.ON376993.1_CurvulariachiangmaiensisisolateND00J7	938	--	938
90.OQ383346.1_NeoscytalidiumdimidiatumisolateGKH-2	297	--	297
91.MF662595.1_NeoscytalidiumnovaehollandiaeisolateNeNo1	284	--	284
92.EF560588.1Melampsoralini	656	--	656
93.LC590862.1_NeoscytalidiumdimidiatumPSU-HP01TEF1	267	--	267
94.KX278106.1_BotryosphaeriaqingyuanensisstrainCERC2947	284	--	284
95.AJ578763.1_Blumeriagraminisf.sp.hordeicyp51	618	GCTTTATCGAAAAATGCGACGATTTTCGAAAATCAAAAGG	657
96.MF490858.1_CurvulariadactylocteniicolastrainCPC28810	930	--	930

97.KT287115.1_Bipolariscactivoraisolate3.8.6	1006	--	1006
98.MT560940.1_CurvulariacactivorastrainHLGH0118	963	--	963
99.OM714565.1_CurvulariaplantarumstrainM0134	983	--	983
100.MN159911.1_BotrytiscinereaSICAUCC19-0003	931	--	931
102.GU294713.1LasiodiplodiatheobromaestrainUCD2430TX	407	--	407
103.KX868094.1_Mycosphaerellasp.isolateCRM20.1	475	--	475
104.LC599478.1Pseudocercosporapini-densifloraeMUCC534	310	--	310
105.N584698.1BipolarissetariaestrainKBS4-2	1031	--	1031

2.OM160859.1_F.buharicum	560	--	560
1.LC727524.1_F.buharicum_OKI-1_Okura	682	--	682
3.KX302919.1_F.sublunatum	521	--	521
4.LT996094.1_F.convolutans	516	--	516
5.OM160861.1_F.abutilonis	630	--	630
6.OM160874.1_F.guadeloupense	560	--	560
7.MH392475.1_F.graminearum	430	--	430
8.MH582420.1_F.solani	540	--	540
9.MAFF244605_F.oxysporum	544	--	544
10.MAFF237278_F.contaminatum_Hylocereus	711	--	711
11.MAFF237649_F.concentricum__Riceroot	678	--	678
12.MAFF237650_F.concentricum__Wheat	679	--	679
13.MAFF239869_F.mangiferae__Ryukyupine	657	--	657
14.MAFF240460_F.fujikuroi_Passionfruit	661	--	661
15.MAFF241317_F.graminearum_Wheat	666	--	666
16.MAFF242670_F.ipomoeae_Wheat	672	--	672
17.MAFF245129_F.concentricum_Fraxinus	659	--	659
18.MAFF245395_F.cugenangense_Rhubarb	671	--	671
19.MAFF246637_F.nirenbergiae_Strawberry	671	--	671
20.MAFF246672_F.nirenbergiae_ChinesePeony	671	--	671
21.MAFF246697_F.commune_Urallicoriceroot	673	--	673
22.MAFF246729_F.falciforme_Angelica	691	--	691
23.MAFF247220_F.duplospermum__Euwallaceasp	694	--	694
24.MAFF410760_F.odoratissimum_alpha	669	--	669
25.MAFF244605_FusariumoxysporumSchlechtendal_MAFF244605_Tomato	672	--	672
26.MAFF241326_F.asiaticum_Wheat	669	--	669
27.MAFF245014_F.asiaticum_Wildsoybean	666	--	666
28.MAFF150124_F.asiaticum__Wheat	666	--	666
29.OM135603.1F.algeriense	699	--	699
30.MAFF237465_F.penzigii_Aloe	704	--	704
31.MAFF103054_F.oxysporumSchlechtendalf.sp.cucumerinum_Cucumber	671	--	671
32.MAFF712246_F.oxysporumSchlechtendalf.sp.dianthi__Carnation	673	--	673
33.MAFF305558_F.oxysporumSchlechtendalf.sp.fragariae__Watermelon	671	--	671
34.MAFF744087_F.oxysporumSchlechtendalf.sp.lactucae__Lettuce	671	--	671
35.MAFF726924_F.oxysporumSchlechtendalf.sp.lagenariae_Whiteflroweredgourd	671	--	671
36.MAFF744003_F.oxysporumSchlechtendalf.sp.lagenariae_Squash)	672	--	672
37.MAFF305122_F.oxysporumSchlechtendalf.sp.melonis__Melon	671	--	671

```
38.MAFF306714_F.oxysporumSchlechtendalf.sp.momordicae_Balsampear         671 ---------------------------------------- 671
39.MAFF238905_F.oxysporumSchlechtendalf.sp.radicis-lycopersici_Tomato    674 ---------------------------------------- 674
40.MAFF150004_F.oxysporumSchlechtendalf.sp.spinaciae_Spinach             671 ---------------------------------------- 671
41.MAFF247034_F.oxysporumSchlechtendal__Goldenchain                      671 ---------------------------------------- 671
42.MAFF245747_F.oxysporumSchlechtendalf.sp.callistephi__Chinaaster       671 ---------------------------------------- 671
43.MAFF305115_FoxysporumSchlechtendalf.sp.batatas__Sweatpotato           703 ---------------------------------------- 703
44.MAFF150126_F.asiaticum_Seed                                           666 ---------------------------------------- 666
45.MAFF246738_F.solani_Angelica                                          703 ---------------------------------------- 703
46.MAFF246664_F.cugenangense_Perilla                                     671 ---------------------------------------- 671
47.MH582420.1F.solanistrainMRC256                                        679 ---------------------------------------- 679
48.MAFF240361_F.babinda_Soil                                             693 ---------------------------------------- 693
49.MAFF242368_F.azukicola_Azukibean                                      708 ---------------------------------------- 708
50.MAFF241312_F.asiaticum_Soil,welshonionfield                           666 ---------------------------------------- 666
51.LT548416.1_F.culmorumpartialtef1a                                     646 ---------------------------------------- 646
52.MAFF150124_F.asiaticum__Wheat                                         666 ---------------------------------------- 666
53.MAFF238806_F.begoniae_Oncidiumsp                                      705 ---------------------------------------- 705
54.MW594399.1_FusariumincarnatumisolateUD01C                             689 ---------------------------------------- 689
55.OP414923.1Pucciniagraminisf.sp.triticiisolateSHZPgt19                 623 ---------------------------------------- 623
56.MT027094.1_BipolarisoryzaestrainOrL-2                                 895 ---------------------------------------- 895
57.ON734360.1_AlternariaalternataisolateH126                             831 ---------------------------------------- 831
58.LC333578.1_StemphyliumlycopersiciSOasp2                               265 ---------------------------------------- 265
59.HQ718583.1_Colletotrichumgloeosporioidesisolateq-1                    567 ---------------------------------------- 567
60.JN241603.1_AtheliarolfsiiisolateSR1                                  1013 ---------------------------------------- 1013
61.KJ866474.1_RhizoctoniasolanistrainMHL-1                               683 ---------------------------------------- 683
62.JQ672424.1AlternariatriticinaisolateEGS17-061                         825 ---------------------------------------- 825
63.LT707559.1_P.capsicipartialtef1agene                                  964 ---------------------------------------- 964
64.MW090051.1_CurvularialunatastrainCls-3                                908 ---------------------------------------- 908
65.DQ400892.1_Aspergillusterreus                                         452 ---------------------------------------- 452
66.DQ911416.1_Pythiumsp.quercumstrainPy292                               932 ---------------------------------------- 932
67.EU797495.1_Phytophthorasp.oaksoilPoland                               930 ---------------------------------------- 930
68.HM148321.1_Cladosporiumcucumerinum                                    385 ---------------------------------------- 385
69.AF398888.1_SclerotiniasclerotiorumisolateSS1                          299 GGAAGCCGCCGAACTCGGTAAGGGTTCCTTCAAGTA---- 334
70.AF398888.1_S.sclerotiorumisolateSS1                                   299 GGAAGCCGCCGAACTCGGTAAGGGTTCCTTCAAGTA---- 334
71.HPAB545908.1_Verticilliumnonalfalfaeisolate                           442 ---------------------------------------- 442
72.EF433315.1_CeratocystisfimbriatavoucherCMW15052                       747 ---------------------------------------- 747
73.MN159912.1_Botrytiscinerea                                            931 ---------------------------------------- 931
74.MF034741.1_PeltasterfructicolaisolateSRB92                            317 ---------------------------------------- 317
75.LC440360.1_CercosporaasparagiCOasp2                                   297 ---------------------------------------- 297
76.AY944105.1_MagnaportheoryzaeisolateSAG00T3()                          255 ---------------------------------------- 255
77.JX266586.1_CochliobolusmiyabeanusvoucherMFLUCC10-0733                 918 ---------------------------------------- 918
78.MN393253.1_CorynesporacassiicolaisolateQHD001(MN393253.1UNVERIFIED)   270 GGAAGCCGCCGAACTCGGTAAGGGTTCCTTCAAGTA---- 305
79.MF375218.1_AgroatheliarolfsiiisolateBJB24                             544 ---------------------------------------- 544
80.MN106270.1_AgroatheliarolfsiistrainJ-12                               509 ---------------------------------------- 509
81.OQ732628.1_AgroatheliarolfsiiisolateBTCBSr3                           499 ---------------------------------------- 499
82.KY196185.1_ColletotrichumtruncatumstrainPAK53                         468 ---------------------------------------- 468
83.GU935835.1_ColletotrichumcoccodesisolateC96002                       1041 ---------------------------------------- 1041
```

```
84.MK085963.1_AlternariatenuissimaisolateSCCZ06            231 GGAAGCCGCCGAACTCGGTAAG------------------ 252
85.MT548042.1_AlternarialongipesstrainKY_2019_012          245 GGAAGCCGCCGAACTCGGTAAGGGTTCCTTCAAGTA---- 280
86.MN356465.1CalonectriamontanaisolateHSP4                 489 ---------------------------------------- 489
87.OL694224.1_CalonectriacanadianastrainF099               504 ---------------------------------------- 504
88.MK803351.1_NeoscytalidiumdimidiatumstrainKale4-C        299 ---------------------------------------- 299
89.ON376993.1_CurvulariachiangmaiensisisolateND00J7        938 ---------------------------------------- 938
90.OQ383346.1_NeoscytalidiumdimidiatumisolateGKH-2         297 ---------------------------------------- 297
91.MF662595.1_NeoscytalidiumnovaehollandiaeisolateNeNo1    284 ---------------------------------------- 284
92.EF560588.1Melampsoralini                                656 ---------------------------------------- 656
93.LC590862.1_NeoscytalidiumdimidiatumPSU-HP01TEF1         267 ---------------------------------------- 267
94.KX278106.1_BotryosphaeriaqingyuanensisstrainCERC2947    284 ---------------------------------------- 284
95.AJ578763.1_Blumeriagraminisf.sp.hordeicyp51             658 TATCATCAATATCGATGCAGTAATGGCTGAAATTACGATA 697
96.MF490858.1_CurvulariadactylocteniicolastrainCPC28810    930 ---------------------------------------- 930
97.KT287115.1_Bipolariscactivoraisolate3.8.6              1006 ---------------------------------------- 1006
98.MT560940.1_CurvulariacactivorastrainHLGH0118            963 ---------------------------------------- 963
99.OM714565.1_CurvulariaplantarumstrainM0134               983 ---------------------------------------- 983
100.MN159911.1_BotrytiscinereaSICAUCC19-0003               931 ---------------------------------------- 931
102.GU294713.1LasiodiplodiatheobromaestrainUCD2430TX       407 ---------------------------------------- 407
103.KX868094.1_Mycosphaerellasp.isolateCRM20.1             475 ---------------------------------------- 475
104.LC599478.1Pseudocercosporapini-densifloraeMUCC534      310 ---------------------------------------- 310
105.N584698.1BipolarissetariaestrainKBS4-2                1031 ---------------------------------------- 1031

2.OM160859.1_F.buharicum                                   560 ---------------------------------------- 560
1.LC727524.1_F.buharicum_OKI-1_Okura                       682 ---------------------------------------- 682
3.KX302919.1_F.sublunatum                                  521 ---------------------------------------- 521
4.LT996094.1_F.convolutans                                 516 ---------------------------------------- 516
5.OM160861.1_F.abutilonis                                  630 ---------------------------------------- 630
6.OM160874.1_F.guadeloupense                               560 ---------------------------------------- 560
7.MH392475.1_F.graminearum                                 430 ---------------------------------------- 430
8.MH582420.1_F.solani                                      540 ---------------------------------------- 540
9.MAFF244605_F.oxysporum                                   544 ---------------------------------------- 544
10.MAFF237278_F.contaminatum_Hylocereus                    711 ---------------------------------------- 711
11.MAFF237649_F.concentricum__Riceroot                     678 ---------------------------------------- 678
12.MAFF237650_F.concentricum__Wheat                        679 ---------------------------------------- 679
13.MAFF239869_F.mangiferae__Ryukyupine                     657 ---------------------------------------- 657
14.MAFF240460_F.fujikuroi_Passionfruit                     661 ---------------------------------------- 661
15.MAFF241317_F.graminearum_Wheat                          666 ---------------------------------------- 666
16.MAFF242670_F.ipomoeae_Wheat                             672 ---------------------------------------- 672
17.MAFF245129_F.concentricum_Fraxinus                      659 ---------------------------------------- 659
18.MAFF245395_F.cugenangense_Rhubarb                       671 ---------------------------------------- 671
19.MAFF246637_F.nirenbergiae_Strawberry                    671 ---------------------------------------- 671
20.MAFF246672_F.nirenbergiae_ChinesePeony                  671 ---------------------------------------- 671
21.MAFF246697_F.commune_Urallicoriceroot                   673 ---------------------------------------- 673
22.MAFF246729_F.falciforme_Angelica                        691 ---------------------------------------- 691
23.MAFF247220_F.duplospermum__Euwallaceasp                 694 ---------------------------------------- 694
24.MAFF410760_F.odoratissimum_alpha                        669 ---------------------------------------- 669
```

25.MAFF244605_FusariumoxysporumSchlechtendal_MAFF244605_Tomato	672	--	672
26.MAFF241326_F.asiaticum_Wheat	669	--	669
27.MAFF245014_F.asiaticum_Wildsoybean	666	--	666
28.MAFF150124_F.asiaticum__Wheat	666	--	666
29.OM135603.1F.algeriense	699	--	699
30.MAFF237465_F.penzigii_Aloe	704	--	704
31.MAFF103054_F.oxysporumSchlechtendalf.sp.cucumerinum_Cucumber	671	--	671
32.MAFF712246_F.oxysporumSchlechtendalf.sp.dianthi__Carnation	673	--	673
33.MAFF305558_F.oxysporumSchlechtendalf.sp.fragariae__Watermelon	671	--	671
34.MAFF744087_F.oxysporumSchlechtendalf.sp.lactucae__Lettuce	671	--	671
35.MAFF726924_F.oxysporumSchlechtendalf.sp.lagenariae_Whitefloweredgourd	671	--	671
36.MAFF744003_F.oxysporumSchlechtendalf.sp.lagenariae_Squash)	672	--	672
37.MAFF305122_F.oxysporumSchlechtendalf.sp.melonis__Melon	671	--	671
38.MAFF306714_F.oxysporumSchlechtendalf.sp.momordicae_Balsampear	671	--	671
39.MAFF238905_F.oxysporumSchlechtendalf.sp.radicis-lycopersici_Tomato	674	--	674
40.MAFF150004_F.oxysporumSchlechtendalf.sp.spinaciae_Spinach	671	--	671
41.MAFF247034_F.oxysporumSchlechtendal__Goldenchain	671	--	671
42.MAFF245747_F.oxysporumSchlechtendalf.sp.callistephi__Chinaaster	671	--	671
43.MAFF305115_FoxysporumSchlechtendalf.sp.batatas__Sweatpotato	703	--	703
44.MAFF150126_F.asiaticum_Seed	666	--	666
45.MAFF246738_F.solani_Angelica	703	--	703
46.MAFF246664_F.cugenangense_Perilla	671	--	671
47.MH582420.1F.solanistrainMRC256	679	--	679
48.MAFF240361_F.babinda_Soil	693	--	693
49.MAFF242368_F.azukicola_Azukibean	708	--	708
50.MAFF241312_F.asiaticum_Soil,welshonionfield	666	--	666
51.LT548416.1_F.culmorumpartialtef1a	646	--	646
52.MAFF150124_F.asiaticum__Wheat	666	--	666
53.MAFF238806_F.begoniae_Oncidiumsp	705	--	705
54.MW594399.1_FusariumincarnatumisolateUD01C	689	--	689
55.OP414923.1Pucciniagraminisf.sp.triticiisolateSHZPgt19	623	--	623
56.MT027094.1_BipolarisoryzaestrainOrL-2	895	--	895
57.ON734360.1_AlternariaalternataisolateH126	831	--	831
58.LC333578.1_StemphyliumlycopersiciSOasp2	265	--	265
59.HQ718583.1_Colletotrichumgloeosporioidesisolateq-1	567	--	567
60.JN241603.1_AtheliarolfsiiisolateSR1	1013	--	1013
61.KJ866474.1_RhizoctoniasolanistrainMHL-1	683	--	683
62.JQ672424.1AlternariatriticinaisolateEGS17-061	825	--	825
63.LT707559.1_P.capsicipartialtef1agene	964	--	964
64.MW090051.1_CurvularialunatastrainCls-3	908	--	908
65.DQ400892.1_Aspergillusterreus	452	--	452
66.DQ911416.1_Pythiumsp.quercumstrainPy292	932	--	932
67.EU797495.1_Phytophthorasp.oaksoilPoland	930	--	930
68.HM148321.1_Cladosporiumcucumerinum	385	--	385
69.AF398888.1_SclerotiniasclerotiorumisolateSS1	334	--	334
70.AF398888.1_S.sclerotiorumisolateSS1	334	--	334

```
71.HPAB545908.1_Verticilliumnonalfalfaeisolate                    442 ---------------------------------------- 442
72.EF433315.1_CeratocystisfimbriatavoucherCMW15052                747 ---------------------------------------- 747
73.MN159912.1_Botrytiscinerea                                     931 ---------------------------------------- 931
74.MF034741.1_PeltasterfructicolaisolateSRB92                     317 ---------------------------------------- 317
75.LC440360.1_CercosporaasparagiCOasp2                            297 ---------------------------------------- 297
76.AY944105.1_MagnaportheoryzaeisolateSAG00T3()                   255 ---------------------------------------- 255
77.JX266586.1_CochliobolusmiyabeanusvoucherMFLUCC10-0733          918 ---------------------------------------- 918
78.MN393253.1_CorynesporacassiicolaisolateQHD001(MN393253.1UNVERIFIED) 305 ---------------------------------------- 305
79.MF375218.1_AgroatheliarolfsiiisolateBJB24                      544 ---------------------------------------- 544
80.MN106270.1_AgroatheliarolfsiistrainJ-12                        509 ---------------------------------------- 509
81.OQ732628.1_AgroatheliarolfsiiisolateBTCBSr3                    499 ---------------------------------------- 499
82.KY196185.1_ColletotrichumtruncatumstrainPAK53                  468 ---------------------------------------- 468
83.GU935835.1_ColletotrichumcoccodesisolateC96002                1041 ---------------------------------------- 1041
84.MK085963.1_AlternariatenuissimaisolateSCCZ06                   252 ---------------------------------------- 252
85.MT548042.1_AlternarialongipesstrainKY_2019_012                 280 ---------------------------------------- 280
86.MN356465.1CalonectriamontanaisolateHSP4                        489 ---------------------------------------- 489
87.OL694224.1_CalonectriacanadianastrainF099                      504 ---------------------------------------- 504
88.MK803351.1_NeoscytalidiumdimidiatumstrainKale4-C               299 ---------------------------------------- 299
89.ON376993.1_CurvulariachiangmaiensisisolateND00J7               938 ---------------------------------------- 938
90.OQ383346.1_NeoscytalidiumdimidiatumisolateGKH-2                297 ---------------------------------------- 297
91.MF662595.1_NeoscytalidiumnovaehollandiaeisolateNeNo1           284 ---------------------------------------- 284
92.EF560588.1Melampsoralini                                       656 ---------------------------------------- 656
93.LC590862.1_NeoscytalidiumdimidiatumPSU-HP01TEF1                267 ---------------------------------------- 267
94.KX278106.1_BotryosphaeriaqingyuanensisstrainCERC2947           284 ---------------------------------------- 284
95.AJ578763.1_Blumeriagraminisf.sp.hordeicyp51                    698 TACACTGCTTCACACACCCTACAAGGGAAGGAAGTTCGCG 737
96.MF490858.1_CurvulariadactylocteniicolastrainCPC28810           930 ---------------------------------------- 930
97.KT287115.1_Bipolariscactivoraisolate3.8.6                     1006 ---------------------------------------- 1006
98.MT560940.1_CurvulariacactivorastrainHLGH0118                   963 ---------------------------------------- 963
99.OM714565.1_CurvulariaplantarumstrainM0134                      983 ---------------------------------------- 983
100.MN159911.1_BotrytiscinereaSICAUCC19-0003                      931 ---------------------------------------- 931
102.GU294713.1LasiodiplodiatheobromaestrainUCD2430TX              407 ---------------------------------------- 407
103.KX868094.1_Mycosphaerellasp.isolateCRM20.1                    475 ---------------------------------------- 475
104.LC599478.1Pseudocercosporapini-densifloraeMUCC534             310 ---------------------------------------- 310
105.N584698.1BipolarissetariaestrainKBS4-2                       1031 ---------------------------------------- 1031

2.OM160859.1_F.buharicum                                          560 ---------------------------------------- 560
1.LC727524.1_F.buharicum_OKI-1_Okura                              682 ---------------------------------------- 682
3.KX302919.1_F.sublunatum                                         521 ---------------------------------------- 521
4.LT996094.1_F.convolutans                                        516 ---------------------------------------- 516
5.OM160861.1_F.abutilonis                                         630 ---------------------------------------- 630
6.OM160874.1_F.guadeloupense                                      560 ---------------------------------------- 560
7.MH392475.1_F.graminearum                                        430 ---------------------------------------- 430
8.MH582420.1_F.solani                                             540 ---------------------------------------- 540
9.MAFF244605_F.oxysporum                                          544 ---------------------------------------- 544
10.MAFF237278_F.contaminatum_Hylocereus                           711 ---------------------------------------- 711
11.MAFF237649_F.concentricum__Riceroot                            678 ---------------------------------------- 678
```

12.MAFF237650_F.concentricum__Wheat	679	--	679
13.MAFF239869_F.mangiferae__Ryukyupine	657	--	657
14.MAFF240460_F.fujikuroi_Passionfruit	661	--	661
15.MAFF241317_F.graminearum_Wheat	666	--	666
16.MAFF242670_F.ipomoeae_Wheat	672	--	672
17.MAFF245129_F.concentricum_Fraxinus	659	--	659
18.MAFF245395_F.cugenangense_Rhubarb	671	--	671
19.MAFF246637_F.nirenbergiae_Strawberry	671	--	671
20.MAFF246672_F.nirenbergiae_ChinesePeony	671	--	671
21.MAFF246697_F.commune_Urallicoriceroot	673	--	673
22.MAFF246729_F.falciforme_Angelica	691	--	691
23.MAFF247220_F.duplospermum__Euwallaceasp	694	--	694
24.MAFF410760_F.odoratissimum_alpha	669	--	669
25.MAFF244605_FusariumoxysporumSchlechtendal_MAFF244605_Tomato	672	--	672
26.MAFF241326_F.asiaticum_Wheat	669	--	669
27.MAFF245014_F.asiaticum_Wildsoybean	666	--	666
28.MAFF150124_F.asiaticum__Wheat	666	--	666
29.OM135603.1F.algeriense	699	--	699
30.MAFF237465_F.penzigii_Aloe	704	--	704
31.MAFF103054_F.oxysporumSchlechtendalf.sp.cucumerinum_Cucumber	671	--	671
32.MAFF712246_F.oxysporumSchlechtendalf.sp.dianthi__Carnation	673	--	673
33.MAFF305558_F.oxysporumSchlechtendalf.sp.fragariae__Watermelon	671	--	671
34.MAFF744087_F.oxysporumSchlechtendalf.sp.lactucae__Lettuce	671	--	671
35.MAFF726924_F.oxysporumSchlechtendalf.sp.lagenariae_Whiteflowered gourd	671	--	671
36.MAFF744003_F.oxysporumSchlechtendalf.sp.lagenariae_Squash)	672	--	672
37.MAFF305122_F.oxysporumSchlechtendalf.sp.melonis__Melon	671	--	671
38.MAFF306714_F.oxysporumSchlechtendalf.sp.momordicae_Balsampear	671	--	671
39.MAFF238905_F.oxysporumSchlechtendalf.sp.radicis-lycopersici_Tomato	674	--	674
40.MAFF150004_F.oxysporumSchlechtendalf.sp.spinaciae_Spinach	671	--	671
41.MAFF247034_F.oxysporumSchlechtendal__Goldenchain	671	--	671
42.MAFF245747_F.oxysporumSchlechtendalf.sp.callistephi__Chinaaster	671	--	671
43.MAFF305115_FoxysporumSchlechtendalf.sp.batatas__Sweatpotato	703	--	703
44.MAFF150126_F.asiaticum_Seed	666	--	666
45.MAFF246738_F.solani_Angelica	703	--	703
46.MAFF246664_F.cugenangense_Perilla	671	--	671
47.MH582420.1F.solanistrainMRC256	679	--	679
48.MAFF240361_F.babinda_Soil	693	--	693
49.MAFF242368_F.azukicola_Azukibean	708	--	708
50.MAFF241312_F.asiaticum_Soil,welshonionfield	666	--	666
51.LT548416.1_F.culmorumpartialtef1a	646	--	646
52.MAFF150124_F.asiaticum__Wheat	666	--	666
53.MAFF238806_F.begoniae_Oncidiumsp	705	--	705
54.MW594399.1_FusariumincarnatumisolateUD01C	689	--	689
55.OP414923.1Pucciniagraminisf.sp.triticiisolateSHZPgt19	623	--	623
56.MT027094.1_BipolarisoryzaestrainOrL-2	895	--	895
57.ON734360.1_AlternariaalternataisolateH126	831	--	831

58.LC333578.1_StemphyliumlycopersiciSOasp2	265	--	265
59.HQ718583.1_Colletotrichumgloeosporioidesisolateq-1	567	--	567
60.JN241603.1_AtheliarolfsiiisolateSR1	1013	--	1013
61.KJ866474.1_RhizoctoniasolanistrainMHL-1	683	--	683
62.JQ672424.1AlternariatriticinaisolateEGS17-061	825	--	825
63.LT707559.1_P.capsicipartialtef1agene	964	--	964
64.MW090051.1_CurvularialunatastrainCls-3	908	--	908
65.DQ400892.1_Aspergillusterreus	452	--	452
66.DQ911416.1_Pythiumsp.quercumstrainPy292	932	--	932
67.EU797495.1_Phytophthorasp.oaksoilPoland	930	--	930
68.HM148321.1_Cladosporiumcucumerinum	385	--	385
69.AF398888.1_SclerotiniasclerotiorumisolateSS1	334	--	334
70.AF398888.1_S.sclerotiorumisolateSS1	334	--	334
71.HPAB545908.1_Verticilliumnonalfalfaeisolate	442	--	442
72.EF433315.1_CeratocystisfimbriatavoucherCMW15052	747	--	747
73.MN159912.1_Botrytiscinerea	931	--	931
74.MF034741.1_PeltasterfructicolaisolateSRB92	317	--	317
75.LC440360.1_CercosporaasparagiCOasp2	297	--	297
76.AY944105.1_MagnaportheoryzaeisolateSAG00T3()	255	--	255
77.JX266586.1_CochliobolusmiyabeanusvoucherMFLUCC10-0733	918	--	918
78.MN393253.1_CorynesporacassiicolaisolateQHD001(MN393253.1UNVERIFIED)	305	--	305
79.MF375218.1_AgroatheliarolfsiiisolateBJB24	544	--	544
80.MN106270.1_AgroatheliarolfsiistrainJ-12	509	--	509
81.OQ732628.1_AgroatheliarolfsiiisolateBTCBSr3	499	--	499
82.KY196185.1_ColletotrichumtruncatumstrainPAK53	468	--	468
83.GU935835.1_ColletotrichumcoccodesisolateC96002	1041	--	1041
84.MK085963.1_AlternariatenuissimaisolateSCCZ06	252	--	252
85.MT548042.1_AlternarialongipesstrainKY_2019_012	280	--	280
86.MN356465.1CalonectriamontanaisolateHSP4	489	--	489
87.OL694224.1_CalonectriacanadianastrainF099	504	--	504
88.MK803351.1_NeoscytalidiumdimidiatumstrainKale4-C	299	--	299
89.ON376993.1_CurvulariachiangmaiensisisolateND00J7	938	--	938
90.OQ383346.1_NeoscytalidiumdimidiatumisolateGKH-2	297	--	297
91.MF662595.1_NeoscytalidiumnovaehollandiaeisolateNeNo1	284	--	284
92.EF560588.1Melampsoralini	656	--	656
93.LC590862.1_NeoscytalidiumdimidiatumPSU-HP01TEF1	267	--	267
94.KX278106.1_BotryosphaeriaqingyuanensisstrainCERC2947	284	--	284
95.AJ578763.1_Blumeriagraminisf.sp.hordeicyp51	738	ATAGATTTGATTCTTCTTTGGCAGTTTTGTATCATGACCT	777
96.MF490858.1_CurvulariadactylocteniicolastrainCPC28810	930	--	930
97.KT287115.1_Bipolariscactivoraisolate3.8.6	1006	--	1006
98.MT560940.1_CurvulariacactivorastrainHLGH0118	963	--	963
99.OM714565.1_CurvulariaplantarumstrainM0134	983	--	983
100.MN159911.1_BotrytiscinereaSICAUCC19-0003	931	--	931
102.GU294713.1LasiodiplodiatheobromaestrainUCD2430TX	407	--	407
103.KX868094.1_Mycosphaerellasp.isolateCRM20.1	475	--	475
104.LC599478.1Pseudocercosporapini-densifloraeMUCC534	310	--	310

```
105.N584698.1BipolarissetariaestrainKBS4-2                              1031 ---------------------------------------- 1031

2.OM160859.1_F.buharicum                                                 560 ---------------------------------------- 560
1.LC727524.1_F.buharicum_OKI-1_Okura                                     682 ---------------------------------------- 682
3.KX302919.1_F.sublunatum                                                521 ---------------------------------------- 521
4.LT996094.1_F.convolutans                                               516 ---------------------------------------- 516
5.OM160861.1_F.abutilonis                                                630 ---------------------------------------- 630
6.OM160874.1_F.guadeloupense                                             560 ---------------------------------------- 560
7.MH392475.1_F.graminearum                                               430 ---------------------------------------- 430
8.MH582420.1_F.solani                                                    540 ---------------------------------------- 540
9.MAFF244605_F.oxysporum                                                 544 ---------------------------------------- 544
10.MAFF237278_F.contaminatum_Hylocereus                                  711 ---------------------------------------- 711
11.MAFF237649_F.concentricum__Riceroot                                   678 ---------------------------------------- 678
12.MAFF237650_F.concentricum__Wheat                                      679 ---------------------------------------- 679
13.MAFF239869_F.mangiferae__Ryukyupine                                   657 ---------------------------------------- 657
14.MAFF240460_F.fujikuroi_Passionfruit                                   661 ---------------------------------------- 661
15.MAFF241317_F.graminearum_Wheat                                        666 ---------------------------------------- 666
16.MAFF242670_F.ipomoeae_Wheat                                           672 ---------------------------------------- 672
17.MAFF245129_F.concentricum_Fraxinus                                    659 ---------------------------------------- 659
18.MAFF245395_F.cugenangense_Rhubarb                                     671 ---------------------------------------- 671
19.MAFF246637_F.nirenbergiae_Strawberry                                  671 ---------------------------------------- 671
20.MAFF246672_F.nirenbergiae_ChinesePeony                                671 ---------------------------------------- 671
21.MAFF246697_F.commune_Urallicoriceroot                                 673 ---------------------------------------- 673
22.MAFF246729_F.falciforme_Angelica                                      691 ---------------------------------------- 691
23.MAFF247220_F.duplospermum__Euwallaceasp                               694 ---------------------------------------- 694
24.MAFF410760_F.odoratissimum_alpha                                      669 ---------------------------------------- 669
25.MAFF244605_FusariumoxysporumSchlechtendal_MAFF244605_Tomato           672 ---------------------------------------- 672
26.MAFF241326_F.asiaticum_Wheat                                          669 ---------------------------------------- 669
27.MAFF245014_F.asiaticum_Wildsoybean                                    666 ---------------------------------------- 666
28.MAFF150124_F.asiaticum__Wheat                                         666 ---------------------------------------- 666
29.OM135603.1F.algeriense                                                699 ---------------------------------------- 699
30.MAFF237465_F.penzigii_Aloe                                            704 ---------------------------------------- 704
31.MAFF103054_F.oxysporumSchlechtendalf.sp.cucumerinum_Cucumber          671 ---------------------------------------- 671
32.MAFF712246_F.oxysporumSchlechtendalf.sp.dianthi__Carnation            673 ---------------------------------------- 673
33.MAFF305558_F.oxysporumSchlechtendalf.sp.fragariae__Watermelon         671 ---------------------------------------- 671
34.MAFF744087_F.oxysporumSchlechtendalf.sp.lactucae__Lettuce             671 ---------------------------------------- 671
35.MAFF726924_F.oxysporumSchlechtendalf.sp.lagenariae_Whiteflowveredgourd 671 ---------------------------------------- 671
36.MAFF744003_F.oxysporumSchlechtendalf.sp.lagenariae_Squash)            672 ---------------------------------------- 672
37.MAFF305122_F.oxysporumSchlechtendalf.sp.melonis__Melon                671 ---------------------------------------- 671
38.MAFF306714_F.oxysporumSchlechtendalf.sp.momordicae_Balsampear         671 ---------------------------------------- 671
39.MAFF238905_F.oxysporumSchlechtendalf.sp.radicis-lycopersici_Tomato    674 ---------------------------------------- 674
40.MAFF150004_F.oxysporumSchlechtendalf.sp.spinaciae_Spinach             671 ---------------------------------------- 671
41.MAFF247034_F.oxysporumSchlechtendal__Goldenchain                      671 ---------------------------------------- 671
42.MAFF245747_F.oxysporumSchlechtendalf.sp.callistephi__Chinaaster       671 ---------------------------------------- 671
43.MAFF305115_FoxysporumSchlechtendalf.sp.batatas__Sweatpotato           703 ---------------------------------------- 703
44.MAFF150126_F.asiaticum_Seed                                           666 ---------------------------------------- 666
```

45.MAFF246738_F.solani_Angelica 703 -- 703
46.MAFF246664_F.cugenangense_Perilla 671 -- 671
47.MH582420.1F.solanistrainMRC256 679 -- 679
48.MAFF240361_F.babinda_Soil 693 -- 693
49.MAFF242368_F.azukicola_Azukibean 708 -- 708
50.MAFF241312_F.asiaticum_Soil,welshonionfield 666 -- 666
51.LT548416.1_F.culmorumpartialtef1a 646 -- 646
52.MAFF150124_F.asiaticum__Wheat 666 -- 666
53.MAFF238806_F.begoniae_Oncidiumsp 705 -- 705
54.MW594399.1_FusariumincarnatumisolateUD01C 689 -- 689
55.OP414923.1Pucciniagraminisf.sp.triticiisolateSHZPgt19 623 -- 623
56.MT027094.1_BipolarisoryzaestrainOrL-2 895 -- 895
57.ON734360.1_AlternariaalternataisolateH126 831 -- 831
58.LC333578.1_StemphyliumlycopersiciSOasp2 265 -- 265
59.HQ718583.1_Colletotrichumgloeosporioidesisolateq-1 567 -- 567
60.JN241603.1_AtheliarolfsiiisolateSR1 1013 -- 1013
61.KJ866474.1_RhizoctoniasolanistrainMHL-1 683 -- 683
62.JQ672424.1AlternariatriticinaisolateEGS17-061 825 -- 825
63.LT707559.1_P.capsicipartialtef1agene 964 -- 964
64.MW090051.1_CurvularialunatastrainCls-3 908 -- 908
65.DQ400892.1_Aspergillusterreus 452 -- 452
66.DQ911416.1_Pythiumsp.quercumstrainPy292 932 -- 932
67.EU797495.1_Phytophthorasp.oaksoilPoland 930 -- 930
68.HM148321.1_Cladosporiumcucumerinum 385 -- 385
69.AF398888.1_SclerotiniasclerotiorumisolateSS1 334 -- 334
70.AF398888.1_S.sclerotiorumisolateSS1 334 -- 334
71.HPAB545908.1_Verticilliumnonalfalfaeisolate 442 -- 442
72.EF433315.1_CeratocystisfimbriatavoucherCMW15052 747 -- 747
73.MN159912.1_Botrytiscinerea 931 -- 931
74.MF034741.1_PeltasterfructicolaisolateSRB92 317 -- 317
75.LC440360.1_CercosporaasparagiCOasp2 297 -- 297
76.AY944105.1_MagnaportheoryzaeisolateSAG00T3() 255 -- 255
77.JX266586.1_CochliobolusmiyabeanusvoucherMFLUCC10-0733 918 -- 918
78.MN393253.1_CorynesporacassiicolaisolateQHD001(MN393253.1UNVERIFIED) 305 -- 305
79.MF375218.1_AgroatheliarolfsiiisolateBJB24 544 -- 544
80.MN106270.1_AgroatheliarolfsiistrainJ-12 509 -- 509
81.OQ732628.1_AgroatheliarolfsiiisolateBTCBSr3 499 -- 499
82.KY196185.1_ColletotrichumtruncatumstrainPAK53 468 -- 468
83.GU935835.1_ColletotrichumcoccodesisolateC96002 1041 -- 1041
84.MK085963.1_AlternariatenuissimaisolateSCCZ06 252 -- 252
85.MT548042.1_AlternarialongipesstrainKY_2019_012 280 -- 280
86.MN356465.1CalonectriamontanaisolateHSP4 489 -- 489
87.OL694224.1_CalonectriacanadianastrainF099 504 -- 504
88.MK803351.1_NeoscytalidiumdimidiatumstrainKale4-C 299 -- 299
89.ON376993.1_CurvulariachiangmaiensisisolateND00J7 938 -- 938
90.OQ383346.1_NeoscytalidiumdimidiatumisolateGKH-2 297 -- 297

91.MF662595.1_NeoscytalidiumnovaehollandiaeisolateNeNo1	284	--	284
92.EF560588.1Melampsoralini	656	--	656
93.LC590862.1_NeoscytalidiumdimidiatumPSU-HP01TEF1	267	--	267
94.KX278106.1_BotryosphaeriaqingyuanensisstrainCERC2947	284	--	284
95.AJ578763.1_Blumeriagraminisf.sp.hordeicyp51	778	AGATATGGGCTTCACCCCAATCAATTTCATGCTTCACTGG	817
96.MF490858.1_CurvulariadactylocteniicolastrainCPC28810	930	--	930
97.KT287115.1_Bipolariscactivoraisolate3.8.6	1006	--	1006
98.MT560940.1_CurvulariacactivorastrainHLGH0118	963	--	963
99.OM714565.1_CurvulariaplantarumstrainM0134	983	--	983
100.MN159911.1_BotrytiscinereaSICAUCC19-0003	931	--	931
102.GU294713.1LasiodiplodiatheobromaestrainUCD2430TX	407	--	407
103.KX868094.1_Mycosphaerellasp.isolateCRM20.1	475	--	475
104.LC599478.1Pseudocercosporapini-densifloraeMUCC534	310	--	310
105.N584698.1BipolarissetariaestrainKBS4-2	1031	--	1031
2.OM160859.1_F.buharicum	560	--	560
1.LC727524.1_F.buharicum_OKI-1_Okura	682	--	682
3.KX302919.1_F.sublunatum	521	--	521
4.LT996094.1_F.convolutans	516	--	516
5.OM160861.1_F.abutilonis	630	--	630
6.OM160874.1_F.guadeloupense	560	--	560
7.MH392475.1_F.graminearum	430	--	430
8.MH582420.1_F.solani	540	--	540
9.MAFF244605_F.oxysporum	544	--	544
10.MAFF237278_F.contaminatum_Hylocereus	711	--	711
11.MAFF237649_F.concentricum__Riceroot	678	--	678
12.MAFF237650_F.concentricum__Wheat	679	--	679
13.MAFF239869_F.mangiferae__Ryukyupine	657	--	657
14.MAFF240460_F.fujikuroi_Passionfruit	661	--	661
15.MAFF241317_F.graminearum_Wheat	666	--	666
16.MAFF242670_F.ipomoeae_Wheat	672	--	672
17.MAFF245129_F.concentricum_Fraxinus	659	--	659
18.MAFF245395_F.cugenangense_Rhubarb	671	--	671
19.MAFF246637_F.nirenbergiae_Strawberry	671	--	671
20.MAFF246672_F.nirenbergiae_ChinesePeony	671	--	671
21.MAFF246697_F.commune_Urallicoriceroot	673	--	673
22.MAFF246729_F.falciforme_Angelica	691	--	691
23.MAFF247220_F.duplospermum__Euwallaceasp	694	--	694
24.MAFF410760_F.odoratissimum_alpha	669	--	669
25.MAFF244605_FusariumoxysporumSchlechtendal_MAFF244605_Tomato	672	--	672
26.MAFF241326_F.asiaticum_Wheat	669	--	669
27.MAFF245014_F.asiaticum_Wildsoybean	666	--	666
28.MAFF150124_F.asiaticum__Wheat	666	--	666
29.OM135603.1F.algeriense	699	--	699
30.MAFF237465_F.penzigii_Aloe	704	--	704
31.MAFF103054_F.oxysporumSchlechtendalf.sp.cucumerinum_Cucumber	671	--	671

32.MAFF712246_F.oxysporumSchlechtendalf.sp.dianthi__Carnation	673	--	673
33.MAFF305558_F.oxysporumSchlechtendalf.sp.fragariae__Watermelon	671	--	671
34.MAFF744087_F.oxysporumSchlechtendalf.sp.lactucae__Lettuce	671	--	671
35.MAFF726924_F.oxysporumSchlechtendalf.sp.lagenariae_Whiteflowered gourd	671	--	671
36.MAFF744003_F.oxysporumSchlechtendalf.sp.lagenariae_Squash)	672	--	672
37.MAFF305122_F.oxysporumSchlechtendalf.sp.melonis__Melon	671	--	671
38.MAFF306714_F.oxysporumSchlechtendalf.sp.momordicae_Balsampear	671	--	671
39.MAFF238905_F.oxysporumSchlechtendalf.sp.radicis-lycopersici_Tomato	674	--	674
40.MAFF150004_F.oxysporumSchlechtendalf.sp.spinaciae_Spinach	671	--	671
41.MAFF247034_F.oxysporumSchlechtendal__Goldenchain	671	--	671
42.MAFF245747_F.oxysporumSchlechtendalf.sp.callistephi__Chinaaster	671	--	671
43.MAFF305115_FoxysporumSchlechtendalf.sp.batatas__Sweatpotato	703	--	703
44.MAFF150126_F.asiaticum_Seed	666	--	666
45.MAFF246738_F.solani_Angelica	703	--	703
46.MAFF246664_F.cugenangense_Perilla	671	--	671
47.MH582420.1F.solanistrainMRC256	679	--	679
48.MAFF240361_F.babinda_Soil	693	--	693
49.MAFF242368_F.azukicola_Azukibean	708	--	708
50.MAFF241312_F.asiaticum_Soil,welshonionfield	666	--	666
51.LT548416.1_F.culmorumpartialtef1a	646	--	646
52.MAFF150124_F.asiaticum__Wheat	666	--	666
53.MAFF238806_F.begoniae_Oncidiumsp	705	--	705
54.MW594399.1_FusariumincarnatumisolateUD01C	689	--	689
55.OP414923.1Pucciniagraminisf.sp.triticiisolateSHZPgt19	623	--	623
56.MT027094.1_BipolarisoryzaestrainOrL-2	895	--	895
57.ON734360.1_AlternariaalternataisolateH126	831	--	831
58.LC333578.1_StemphyliumlycopersiciSOasp2	265	--	265
59.HQ718583.1_Colletotrichumgloeosporioidesisolateq-1	567	--	567
60.JN241603.1_AtheliarolfsiiisolateSR1	1013	--	1013
61.KJ866474.1_RhizoctoniasolanistrainMHL-1	683	--	683
62.JQ672424.1AlternariatriticinaisolateEGS17-061	825	--	825
63.LT707559.1_P.capsicipartialtef1agene	964	--	964
64.MW090051.1_CurvularialunatastrainCls-3	908	--	908
65.DQ400892.1_Aspergillusterreus	452	--	452
66.DQ911416.1_Pythiumsp.quercumstrainPy292	932	--	932
67.EU797495.1_Phytophthorasp.oaksoilPoland	930	--	930
68.HM148321.1_Cladosporiumcucumerinum	385	--	385
69.AF398888.1_SclerotiniasclerotiorumisolateSS1	334	--	334
70.AF398888.1_S.sclerotiorumisolateSS1	334	--	334
71.HPAB545908.1_Verticilliumnonalfalfaeisolate	442	--	442
72.EF433315.1_CeratocystisfimbriatavoucherCMW15052	747	--	747
73.MN159912.1_Botrytiscinerea	931	--	931
74.MF034741.1_PeltasterfructicolaisolateSRB92	317	--	317
75.LC440360.1_CercosporaasparagiCOasp2	297	--	297
76.AY944105.1_MagnaportheoryzaeisolateSAG00T3()	255	--	255
77.JX266586.1_CochliobolusmiyabeanusvoucherMFLUCC10-0733	918	--	918

```
78.MN393253.1_CorynesporacassiicolaisolateQHD001(MN393253.1UNVERIFIED)   305 ---------------------------------------- 305
79.MF375218.1_AgroatheliarolfsiiisolateBJB24                             544 ---------------------------------------- 544
80.MN106270.1_AgroatheliarolfsiistrainJ-12                               509 ---------------------------------------- 509
81.OQ732628.1_AgroatheliarolfsiiisolateBTCBSr3                           499 ---------------------------------------- 499
82.KY196185.1_ColletotrichumtruncatumstrainPAK53                         468 ---------------------------------------- 468
83.GU935835.1_ColletotrichumcoccodesisolateC96002                       1041 ---------------------------------------- 1041
84.MK085963.1_AlternariatenuissimaisolateSCCZ06                          252 ---------------------------------------- 252
85.MT548042.1_AlternarialongipesstrainKY_2019_012                        280 ---------------------------------------- 280
86.MN356465.1CalonectriamontanaisolateHSP4                               489 ---------------------------------------- 489
87.OL694224.1_CalonectriacanadianastrainF099                             504 ---------------------------------------- 504
88.MK803351.1_NeoscytalidiumdimidiatumstrainKale4-C                      299 ---------------------------------------- 299
89.ON376993.1_CurvulariachiangmaiensisisolateND00J7                      938 ---------------------------------------- 938
90.OQ383346.1_NeoscytalidiumdimidiatumisolateGKH-2                       297 ---------------------------------------- 297
91.MF662595.1_NeoscytalidiumnovaehollandiaeisolateNeNo1                  284 ---------------------------------------- 284
92.EF560588.1Melampsoralini                                              656 ---------------------------------------- 656
93.LC590862.1_NeoscytalidiumdimidiatumPSU-HP01TEF1                       267 ---------------------------------------- 267
94.KX278106.1_BotryosphaeriaqingyuanensisstrainCERC2947                  284 ---------------------------------------- 284
95.AJ578763.1_Blumeriagraminisf.sp.hordeicyp51                           818 GCACCACTTCCGCACAATCGAGCTCGTGATCATGCCCAAC 857
96.MF490858.1_CurvulariadactylocteniicolastrainCPC28810                  930 ---------------------------------------- 930
97.KT287115.1_Bipolariscactivoraisolate3.8.6                            1006 ---------------------------------------- 1006
98.MT560940.1_CurvulariacactivorastrainHLGH0118                          963 ---------------------------------------- 963
99.OM714565.1_CurvulariaplantarumstrainM0134                             983 ---------------------------------------- 983
100.MN159911.1_BotrytiscinereaSICAUCC19-0003                             931 ---------------------------------------- 931
102.GU294713.1LasiodiplodiatheobromaestrainUCD2430TX                     407 ---------------------------------------- 407
103.KX868094.1_Mycosphaerellasp.isolateCRM20.1                           475 ---------------------------------------- 475
104.LC599478.1Pseudocercosporapini-densifloraeMUCC534                    310 ---------------------------------------- 310
105.N584698.1BipolarissetariaestrainKBS4-2                              1031 ---------------------------------------- 1031

2.OM160859.1_F.buharicum                                                 560 ---------------------------------------- 560
1.LC727524.1_F.buharicum_OKI-1_Okura                                     682 ---------------------------------------- 682
3.KX302919.1_F.sublunatum                                                521 ---------------------------------------- 521
4.LT996094.1_F.convolutans                                               516 ---------------------------------------- 516
5.OM160861.1_F.abutilonis                                                630 ---------------------------------------- 630
6.OM160874.1_F.guadeloupense                                             560 ---------------------------------------- 560
7.MH392475.1_F.graminearum                                               430 ---------------------------------------- 430
8.MH582420.1_F.solani                                                    540 ---------------------------------------- 540
9.MAFF244605_F.oxysporum                                                 544 ---------------------------------------- 544
10.MAFF237278_F.contaminatum_Hylocereus                                  711 ---------------------------------------- 711
11.MAFF237649_F.concentricum__Riceroot                                   678 ---------------------------------------- 678
12.MAFF237650_F.concentricum__Wheat                                      679 ---------------------------------------- 679
13.MAFF239869_F.mangiferae__Ryukyupine                                   657 ---------------------------------------- 657
14.MAFF240460_F.fujikuroi_Passionfruit                                   661 ---------------------------------------- 661
15.MAFF241317_F.graminearum_Wheat                                        666 ---------------------------------------- 666
16.MAFF242670_F.ipomoeae_Wheat                                           672 ---------------------------------------- 672
17.MAFF245129_F.concentricum_Fraxinus                                    659 ---------------------------------------- 659
18.MAFF245395_F.cugenangense_Rhubarb                                     671 ---------------------------------------- 671
```

```
19.MAFF246637_F.nirenbergiae_Strawberry                                671 ---------------------------------------- 671
20.MAFF246672_F.nirenbergiae_ChinesePeony                              671 ---------------------------------------- 671
21.MAFF246697_F.commune_Urallicoriceroot                               673 ---------------------------------------- 673
22.MAFF246729_F.falciforme_Angelica                                    691 ---------------------------------------- 691
23.MAFF247220_F.duplospermum__Euwallaceasp                             694 ---------------------------------------- 694
24.MAFF410760_F.odoratissimum_alpha                                    669 ---------------------------------------- 669
25.MAFF244605_FusariumoxysporumSchlechtendal_MAFF244605_Tomato         672 ---------------------------------------- 672
26.MAFF241326_F.asiaticum_Wheat                                        669 ---------------------------------------- 669
27.MAFF245014_F.asiaticum_Wildsoybean                                  666 ---------------------------------------- 666
28.MAFF150124_F.asiaticum__Wheat                                       666 ---------------------------------------- 666
29.OM135603.1F.algeriense                                              699 ---------------------------------------- 699
30.MAFF237465_F.penzigii_Aloe                                          704 ---------------------------------------- 704
31.MAFF103054_F.oxysporumSchlechtendalf.sp.cucumerinum_Cucumber        671 ---------------------------------------- 671
32.MAFF712246_F.oxysporumSchlechtendalf.sp.dianthi__Carnation          673 ---------------------------------------- 673
33.MAFF305558_F.oxysporumSchlechtendalf.sp.fragariae__Watermelon       671 ---------------------------------------- 671
34.MAFF744087_F.oxysporumSchlechtendalf.sp.lactucae__Lettuce           671 ---------------------------------------- 671
35.MAFF726924_F.oxysporumSchlechtendalf.sp.lagenariae_Whitefloweredgourd 671 ---------------------------------------- 671
36.MAFF744003_F.oxysporumSchlechtendalf.sp.lagenariae_Squash)          672 ---------------------------------------- 672
37.MAFF305122_F.oxysporumSchlechtendalf.sp.melonis__Melon              671 ---------------------------------------- 671
38.MAFF306714_F.oxysporumSchlechtendalf.sp.momordicae_Balsampear       671 ---------------------------------------- 671
39.MAFF238905_F.oxysporumSchlechtendalf.sp.radicis-lycopersici_Tomato  674 ---------------------------------------- 674
40.MAFF150004_F.oxysporumSchlechtendalf.sp.spinaciae_Spinach           671 ---------------------------------------- 671
41.MAFF247034_F.oxysporumSchlechtendal__Goldenchain                    671 ---------------------------------------- 671
42.MAFF245747_F.oxysporumSchlechtendalf.sp.callistephi__Chinaaster     671 ---------------------------------------- 671
43.MAFF305115_FoxysporumSchlechtendalf.sp.batatas__Sweatpotato         703 ---------------------------------------- 703
44.MAFF150126_F.asiaticum_Seed                                         666 ---------------------------------------- 666
45.MAFF246738_F.solani_Angelica                                        703 ---------------------------------------- 703
46.MAFF246664_F.cugenangense_Perilla                                   671 ---------------------------------------- 671
47.MH582420.1F.solanistrainMRC256                                      679 ---------------------------------------- 679
48.MAFF240361_F.babinda_Soil                                           693 ---------------------------------------- 693
49.MAFF242368_F.azukicola_Azukibean                                    708 ---------------------------------------- 708
50.MAFF241312_F.asiaticum_Soil,welshonionfield                         666 ---------------------------------------- 666
51.LT548416.1_F.culmorumpartialtef1a                                   646 ---------------------------------------- 646
52.MAFF150124_F.asiaticum__Wheat                                       666 ---------------------------------------- 666
53.MAFF238806_F.begoniae_Oncidiumsp                                    705 ---------------------------------------- 705
54.MW594399.1_FusariumincarnatumisolateUD01C                           689 ---------------------------------------- 689
55.OP414923.1Pucciniagraminisf.sp.triticiisolateSHZPgt19               623 ---------------------------------------- 623
56.MT027094.1_BipolarisoryzaestrainOrL-2                               895 ---------------------------------------- 895
57.ON734360.1_AlternariaalternataisolateH126                           831 ---------------------------------------- 831
58.LC333578.1_StemphyliumlycopersiciSOasp2                             265 ---------------------------------------- 265
59.HQ718583.1_Colletotrichumgloeosporioidesisolateq-1                  567 ---------------------------------------- 567
60.JN241603.1_AtheliarolfsiiisolateSR1                                1013 ---------------------------------------- 1013
61.KJ866474.1_RhizoctoniasolanistrainMHL-1                             683 ---------------------------------------- 683
62.JQ672424.1AlternariatriticinaisolateEGS17-061                       825 ---------------------------------------- 825
63.LT707559.1_P.capsicipartialtef1agene                                964 ---------------------------------------- 964
64.MW090051.1_CurvularialunatastrainCls-3                              908 ---------------------------------------- 908
```

```
65.DQ400892.1_Aspergillusterreus                                     452 ---------------------------------------- 452
66.DQ911416.1_Pythiumsp.quercumstrainPy292                           932 ---------------------------------------- 932
67.EU797495.1_Phytophthorasp.oaksoilPoland                           930 ---------------------------------------- 930
68.HM148321.1_Cladosporiumcucumerinum                                385 ---------------------------------------- 385
69.AF398888.1_SclerotiniasclerotiorumisolateSS1                      334 ---------------------------------------- 334
70.AF398888.1_S.sclerotiorumisolateSS1                               334 ---------------------------------------- 334
71.HPAB545908.1_Verticilliumnonalfalfaeisolate                       442 ---------------------------------------- 442
72.EF433315.1_CeratocystisfimbriatavoucherCMW15052                   747 ---------------------------------------- 747
73.MN159912.1_Botrytiscinerea                                        931 ---------------------------------------- 931
74.MF034741.1_PeltasterfructicolaisolateSRB92                        317 ---------------------------------------- 317
75.LC440360.1_CercosporaasparagiCOasp2                               297 ---------------------------------------- 297
76.AY944105.1_MagnaportheoryzaeisolateSAG00T3()                      255 ---------------------------------------- 255
77.JX266586.1_CochliobolusmiyabeanusvoucherMFLUCC10-0733              918 ---------------------------------------- 918
78.MN393253.1_CorynesporacassiicolaisolateQHD001(MN393253.1UNVERIFIED) 305 ---------------------------------------- 305
79.MF375218.1_AgroatheliarolfsiiisolateBJB24                         544 ---------------------------------------- 544
80.MN106270.1_AgroatheliarolfsiistrainJ-12                           509 ---------------------------------------- 509
81.OQ732628.1_AgroatheliarolfsiiisolateBTCBSr3                       499 ---------------------------------------- 499
82.KY196185.1_ColletotrichumtruncatumstrainPAK53                     468 ---------------------------------------- 468
83.GU935835.1_ColletotrichumcoccodesisolateC96002                   1041 ---------------------------------------- 1041
84.MK085963.1_AlternariatenuissimaisolateSCCZ06                      252 ---------------------------------------- 252
85.MT548042.1_AlternarialongipesstrainKY_2019_012                    280 ---------------------------------------- 280
86.MN356465.1CalonectriamontanaisolateHSP4                           489 ---------------------------------------- 489
87.OL694224.1_CalonectriacanadianastrainF099                         504 ---------------------------------------- 504
88.MK803351.1_NeoscytalidiumdimidiatumstrainKale4-C                  299 ---------------------------------------- 299
89.ON376993.1_CurvulariachiangmaiensisisolateND00J7                  938 ---------------------------------------- 938
90.OQ383346.1_NeoscytalidiumdimidiatumisolateGKH-2                   297 ---------------------------------------- 297
91.MF662595.1_NeoscytalidiumnovaehollandiaeisolateNeNo1              284 ---------------------------------------- 284
92.EF560588.1Melampsoralini                                          656 ---------------------------------------- 656
93.LC590862.1_NeoscytalidiumdimidiatumPSU-HP01TEF1                   267 ---------------------------------------- 267
94.KX278106.1_BotryosphaeriaqingyuanensisstrainCERC2947              284 ---------------------------------------- 284
95.AJ578763.1_Blumeriagraminisf.sp.hordeicyp51                       858 GGACAGTCGCAAAGATATACATGGAGATTATCAACAGCCG 897
96.MF490858.1_CurvulariadactylocteniicolastrainCPC28810              930 ---------------------------------------- 930
97.KT287115.1_Bipolariscactivoraisolate3.8.6                        1006 ---------------------------------------- 1006
98.MT560940.1_CurvulariacactivorastrainHLGH0118                      963 ---------------------------------------- 963
99.OM714565.1_CurvulariaplantarumstrainM0134                         983 ---------------------------------------- 983
100.MN159911.1_BotrytiscinereaSICAUCC19-0003                         931 ---------------------------------------- 931
102.GU294713.1LasiodiplodiatheobromaestrainUCD2430TX                 407 ---------------------------------------- 407
103.KX868094.1_Mycosphaerellasp.isolateCRM20.1                       475 ---------------------------------------- 475
104.LC599478.1Pseudocercosporapini-densifloraeMUCC534                310 ---------------------------------------- 310
105.N584698.1BipolarissetariaestrainKBS4-2                          1031 ---------------------------------------- 1031

2.OM160859.1_F.buharicum                                             560 ---------------------------------------- 560
1.LC727524.1_F.buharicum_OKI-1_Okura                                 682 ---------------------------------------- 682
3.KX302919.1_F.sublunatum                                            521 ---------------------------------------- 521
4.LT996094.1_F.convolutans                                           516 ---------------------------------------- 516
5.OM160861.1_F.abutilonis                                            630 ---------------------------------------- 630
```

```
6.OM160874.1_F.guadeloupense                                               560 ---------------------------------------- 560
7.MH392475.1_F.graminearum                                                 430 ---------------------------------------- 430
8.MH582420.1_F.solani                                                      540 ---------------------------------------- 540
9.MAFF244605_F.oxysporum                                                   544 ---------------------------------------- 544
10.MAFF237278_F.contaminatum_Hylocereus                                    711 ---------------------------------------- 711
11.MAFF237649_F.concentricum__Riceroot                                     678 ---------------------------------------- 678
12.MAFF237650_F.concentricum__Wheat                                        679 ---------------------------------------- 679
13.MAFF239869_F.mangiferae__Ryukyupine                                     657 ---------------------------------------- 657
14.MAFF240460_F.fujikuroi_Passionfruit                                     661 ---------------------------------------- 661
15.MAFF241317_F.graminearum_Wheat                                          666 ---------------------------------------- 666
16.MAFF242670_F.ipomoeae_Wheat                                             672 ---------------------------------------- 672
17.MAFF245129_F.concentricum_Fraxinus                                      659 ---------------------------------------- 659
18.MAFF245395_F.cugenangense_Rhubarb                                       671 ---------------------------------------- 671
19.MAFF246637_F.nirenbergiae_Strawberry                                    671 ---------------------------------------- 671
20.MAFF246672_F.nirenbergiae_ChinesePeony                                  671 ---------------------------------------- 671
21.MAFF246697_F.commune_Urallicoriceroot                                   673 ---------------------------------------- 673
22.MAFF246729_F.falciforme_Angelica                                        691 ---------------------------------------- 691
23.MAFF247220_F.duplospermum__Euwallaceasp                                 694 ---------------------------------------- 694
24.MAFF410760_F.odoratissimum_alpha                                        669 ---------------------------------------- 669
25.MAFF244605_FusariumoxysporumSchlechtendal_MAFF244605_Tomato             672 ---------------------------------------- 672
26.MAFF241326_F.asiaticum_Wheat                                            669 ---------------------------------------- 669
27.MAFF245014_F.asiaticum_Wildsoybean                                      666 ---------------------------------------- 666
28.MAFF150124_F.asiaticum__Wheat                                           666 ---------------------------------------- 666
29.OM135603.1F.algeriense                                                  699 ---------------------------------------- 699
30.MAFF237465_F.penzigii_Aloe                                              704 ---------------------------------------- 704
31.MAFF103054_F.oxysporumSchlechtendalf.sp.cucumerinum_Cucumber            671 ---------------------------------------- 671
32.MAFF712246_F.oxysporumSchlechtendalf.sp.dianthi__Carnation              673 ---------------------------------------- 673
33.MAFF305558_F.oxysporumSchlechtendalf.sp.fragariae__Watermelon           671 ---------------------------------------- 671
34.MAFF744087_F.oxysporumSchlechtendalf.sp.lactucae__Lettuce               671 ---------------------------------------- 671
35.MAFF726924_F.oxysporumSchlechtendalf.sp.lagenariae_Whitefloweredgourd   671 ---------------------------------------- 671
36.MAFF744003_F.oxysporumSchlechtendalf.sp.lagenariae_Squash)              672 ---------------------------------------- 672
37.MAFF305122_F.oxysporumSchlechtendalf.sp.melonis__Melon                  671 ---------------------------------------- 671
38.MAFF306714_F.oxysporumSchlechtendalf.sp.momordicae_Balsampear           671 ---------------------------------------- 671
39.MAFF238905_F.oxysporumSchlechtendalf.sp.radicis-lycopersici_Tomato      674 ---------------------------------------- 674
40.MAFF150004_F.oxysporumSchlechtendalf.sp.spinaciae_Spinach               671 ---------------------------------------- 671
41.MAFF247034_F.oxysporumSchlechtendal__Goldenchain                        671 ---------------------------------------- 671
42.MAFF245747_F.oxysporumSchlechtendalf.sp.callistephi__Chinaaster         671 ---------------------------------------- 671
43.MAFF305115_FoxysporumSchlechtendalf.sp.batatas__Sweatpotato             703 ---------------------------------------- 703
44.MAFF150126_F.asiaticum_Seed                                             666 ---------------------------------------- 666
45.MAFF246738_F.solani_Angelica                                            703 ---------------------------------------- 703
46.MAFF246664_F.cugenangense_Perilla                                       671 ---------------------------------------- 671
47.MH582420.1F.solanistrainMRC256                                          679 ---------------------------------------- 679
48.MAFF240361_F.babinda_Soil                                               693 ---------------------------------------- 693
49.MAFF242368_F.azukicola_Azukibean                                        708 ---------------------------------------- 708
50.MAFF241312_F.asiaticum_Soil,welshonionfield                             666 ---------------------------------------- 666
51.LT548416.1_F.culmorumpartialtef1a                                       646 ---------------------------------------- 646
```

52.MAFF150124_F.asiaticum__Wheat	666	--	666
53.MAFF238806_F.begoniae_Oncidiumsp	705	--	705
54.MW594399.1_FusariumincarnatumisolateUD01C	689	--	689
55.OP414923.1Pucciniagraminisf.sp.triticiisolateSHZPgt19	623	--	623
56.MT027094.1_BipolarisoryzaestrainOrL-2	895	--	895
57.ON734360.1_AlternariaalternataisolateH126	831	--	831
58.LC333578.1_StemphyliumlycopersiciSOasp2	265	--	265
59.HQ718583.1_Colletotrichumgloeosporioidesisolateq-1	567	--	567
60.JN241603.1_AtheliarolfsiiisolateSR1	1013	--	1013
61.KJ866474.1_RhizoctoniasolanistrainMHL-1	683	--	683
62.JQ672424.1AlternariatriticinaisolateEGS17-061	825	--	825
63.LT707559.1_P.capsicipartialtef1agene	964	--	964
64.MW090051.1_CurvularialunatastrainCls-3	908	--	908
65.DQ400892.1_Aspergillusterreus	452	--	452
66.DQ911416.1_Pythiumsp.quercumstrainPy292	932	--	932
67.EU797495.1_Phytophthorasp.oaksoilPoland	930	--	930
68.HM148321.1_Cladosporiumcucumerinum	385	--	385
69.AF398888.1_SclerotiniasclerotiorumisolateSS1	334	--	334
70.AF398888.1_S.sclerotiorumisolateSS1	334	--	334
71.HPAB545908.1_Verticilliumnonalfalfaeisolate	442	--	442
72.EF433315.1_CeratocystisfimbriatavoucherCMW15052	747	--	747
73.MN159912.1_Botrytiscinerea	931	--	931
74.MF034741.1_PeltasterfructicolaisolateSRB92	317	--	317
75.LC440360.1_CercosporaasparagiCOasp2	297	--	297
76.AY944105.1_MagnaportheoryzaeisolateSAG00T3()	255	--	255
77.JX266586.1_CochliobolusmiyabeanusvoucherMFLUCC10-0733	918	--	918
78.MN393253.1_CorynesporacassiicolaisolateQHD001(MN393253.1UNVERIFIED)	305	--	305
79.MF375218.1_AgroatheliarolfsiiisolateBJB24	544	--	544
80.MN106270.1_AgroatheliarolfsiistrainJ-12	509	--	509
81.OQ732628.1_AgroatheliarolfsiiisolateBTCBSr3	499	--	499
82.KY196185.1_ColletotrichumtruncatumstrainPAK53	468	--	468
83.GU935835.1_ColletotrichumcoccodesisolateC96002	1041	--	1041
84.MK085963.1_AlternariatenuissimaisolateSCCZ06	252	--	252
85.MT548042.1_AlternarialongipesstrainKY_2019_012	280	--	280
86.MN356465.1CalonectriamontanaisolateHSP4	489	--	489
87.OL694224.1_CalonectriacanadianastrainF099	504	--	504
88.MK803351.1_NeoscytalidiumdimidiatumstrainKale4-C	299	--	299
89.ON376993.1_CurvulariachiangmaiensisisolateND00J7	938	--	938
90.OQ383346.1_NeoscytalidiumdimidiatumisolateGKH-2	297	--	297
91.MF662595.1_NeoscytalidiumnovaehollandiaeisolateNeNo1	284	--	284
92.EF560588.1Melampsoralini	656	--	656
93.LC590862.1_NeoscytalidiumdimidiatumPSU-HP01TEF1	267	--	267
94.KX278106.1_BotryosphaeriaqingyuanensisstrainCERC2947	284	--	284
95.AJ578763.1_Blumeriagraminisf.sp.hordeicyp51	898	TCGGACGCAGAAAGAAACTGATGATTCCAATTTAGATATA	937
96.MF490858.1_CurvulariadactylocteniicolastrainCPC28810	930	--	930
97.KT287115.1_Bipolariscactivoraisolate3.8.6	1006	--	1006

```
98.MT560940.1_CurvulariacactivorastrainHLGH0118                  963 ---------------------------------------- 963
99.OM714565.1_CurvulariaplantarumstrainM0134                     983 ---------------------------------------- 983
100.MN159911.1_BotrytiscinereaSICAUCC19-0003                     931 ---------------------------------------- 931
102.GU294713.1LasiodiplodiatheobromaestrainUCD2430TX             407 ---------------------------------------- 407
103.KX868094.1_Mycosphaerellasp.isolateCRM20.1                   475 ---------------------------------------- 475
104.LC599478.1Pseudocercosporapini-densifloraeMUCC534            310 ---------------------------------------- 310
105.N584698.1BipolarissetariaestrainKBS4-2                      1031 ---------------------------------------- 1031

2.OM160859.1_F.buharicum                                         560 ---------------------------------------- 560
1.LC727524.1_F.buharicum_OKI-1_Okura                             682 ---------------------------------------- 682
3.KX302919.1_F.sublunatum                                        521 ---------------------------------------- 521
4.LT996094.1_F.convolutans                                       516 ---------------------------------------- 516
5.OM160861.1_F.abutilonis                                        630 ---------------------------------------- 630
6.OM160874.1_F.guadeloupense                                     560 ---------------------------------------- 560
7.MH392475.1_F.graminearum                                       430 ---------------------------------------- 430
8.MH582420.1_F.solani                                            540 ---------------------------------------- 540
9.MAFF244605_F.oxysporum                                         544 ---------------------------------------- 544
10.MAFF237278_F.contaminatum_Hylocereus                          711 ---------------------------------------- 711
11.MAFF237649_F.concentricum__Riceroot                           678 ---------------------------------------- 678
12.MAFF237650_F.concentricum__Wheat                              679 ---------------------------------------- 679
13.MAFF239869_F.mangiferae__Ryukyupine                           657 ---------------------------------------- 657
14.MAFF240460_F.fujikuroi_Passionfruit                           661 ---------------------------------------- 661
15.MAFF241317_F.graminearum_Wheat                                666 ---------------------------------------- 666
16.MAFF242670_F.ipomoeae_Wheat                                   672 ---------------------------------------- 672
17.MAFF245129_F.concentricum_Fraxinus                            659 ---------------------------------------- 659
18.MAFF245395_F.cugenangense_Rhubarb                             671 ---------------------------------------- 671
19.MAFF246637_F.nirenbergiae_Strawberry                          671 ---------------------------------------- 671
20.MAFF246672_F.nirenbergiae_ChinesePeony                        671 ---------------------------------------- 671
21.MAFF246697_F.commune_Urallicoriceroot                         673 ---------------------------------------- 673
22.MAFF246729_F.falciforme_Angelica                              691 ---------------------------------------- 691
23.MAFF247220_F.duplospermum__Euwallaceasp                       694 ---------------------------------------- 694
24.MAFF410760_F.odoratissimum_alpha                              669 ---------------------------------------- 669
25.MAFF244605_FusariumoxysporumSchlechtendal_MAFF244605_Tomato   672 ---------------------------------------- 672
26.MAFF241326_F.asiaticum_Wheat                                  669 ---------------------------------------- 669
27.MAFF245014_F.asiaticum_Wildsoybean                            666 ---------------------------------------- 666
28.MAFF150124_F.asiaticum__Wheat                                 666 ---------------------------------------- 666
29.OM135603.1F.algeriense                                        699 ---------------------------------------- 699
30.MAFF237465_F.penzigii_Aloe                                    704 ---------------------------------------- 704
31.MAFF103054_F.oxysporumSchlechtendalf.sp.cucumerinum_Cucumber  671 ---------------------------------------- 671
32.MAFF712246_F.oxysporumSchlechtendalf.sp.dianthi__Carnation    673 ---------------------------------------- 673
33.MAFF305558_F.oxysporumSchlechtendalf.sp.fragariae__Watermelon 671 ---------------------------------------- 671
34.MAFF744087_F.oxysporumSchlechtendalf.sp.lactucae__Lettuce     671 ---------------------------------------- 671
35.MAFF726924_F.oxysporumSchlechtendalf.sp.lagenariae_Whitefloweredgourd 671 ---------------------------------------- 671
36.MAFF744003_F.oxysporumSchlechtendalf.sp.lagenariae_Squash)    672 ---------------------------------------- 672
37.MAFF305122_F.oxysporumSchlechtendalf.sp.melonis__Melon        671 ---------------------------------------- 671
38.MAFF306714_F.oxysporumSchlechtendalf.sp.momordicae_Balsampear 671 ---------------------------------------- 671
```

39.MAFF238905_F.oxysporumSchlechtendalf.sp.radicis-lycopersici_Tomato	674	--	674
40.MAFF150004_F.oxysporumSchlechtendalf.sp.spinaciae_Spinach	671	--	671
41.MAFF247034_F.oxysporumSchlechtendal__Goldenchain	671	--	671
42.MAFF245747_F.oxysporumSchlechtendalf.sp.callistephi__Chinaaster	671	--	671
43.MAFF305115_FoxysporumSchlechtendalf.sp.batatas__Sweatpotato	703	--	703
44.MAFF150126_F.asiaticum_Seed	666	--	666
45.MAFF246738_F.solani_Angelica	703	--	703
46.MAFF246664_F.cugenangense_Perilla	671	--	671
47.MH582420.1F.solanistrainMRC256	679	--	679
48.MAFF240361_F.babinda_Soil	693	--	693
49.MAFF242368_F.azukicola_Azukibean	708	--	708
50.MAFF241312_F.asiaticum_Soil,welshonionfield	666	--	666
51.LT548416.1_F.culmorumpartialtef1a	646	--	646
52.MAFF150124_F.asiaticum__Wheat	666	--	666
53.MAFF238806_F.begoniae_Oncidiumsp	705	--	705
54.MW594399.1_FusariumincarnatumisolateUD01C	689	--	689
55.OP414923.1Pucciniagraminisf.sp.triticiisolateSHZPgt19	623	--	623
56.MT027094.1_BipolarisoryzaestrainOrL-2	895	--	895
57.ON734360.1_AlternariaalternataisolateH126	831	--	831
58.LC333578.1_StemphyliumlycopersiciSOasp2	265	--	265
59.HQ718583.1_Colletotrichumgloeosporioidesisolateq-1	567	--	567
60.JN241603.1_AtheliarolfsiiisolateSR1	1013	--	1013
61.KJ866474.1_RhizoctoniasolanistrainMHL-1	683	--	683
62.JQ672424.1AlternariatriticinaisolateEGS17-061	825	--	825
63.LT707559.1_P.capsicipartialtef1agene	964	--	964
64.MW090051.1_CurvularialunatastrainCls-3	908	--	908
65.DQ400892.1_Aspergillusterreus	452	--	452
66.DQ911416.1_Pythiumsp.quercumstrainPy292	932	--	932
67.EU797495.1_Phytophthorasp.oaksoilPoland	930	--	930
68.HM148321.1_Cladosporiumcucumerinum	385	--	385
69.AF398888.1_SclerotiniasclerotiorumisolateSS1	334	--	334
70.AF398888.1_S.sclerotiorumisolateSS1	334	--	334
71.HPAB545908.1_Verticilliumnonalfalfaeisolate	442	--	442
72.EF433315.1_CeratocystisfimbriatavoucherCMW15052	747	--	747
73.MN159912.1_Botrytiscinerea	931	--	931
74.MF034741.1_PeltasterfructicolaisolateSRB92	317	--	317
75.LC440360.1_CercosporaasparagiCOasp2	297	--	297
76.AY944105.1_MagnaportheoryzaeisolateSAG00T3()	255	--	255
77.JX266586.1_CochliobolusmiyabeanusvoucherMFLUCC10-0733	918	--	918
78.MN393253.1_CorynesporacassiicolaisolateQHD001(MN393253.1UNVERIFIED)	305	--	305
79.MF375218.1_AgroatheliarolfsiiisolateBJB24	544	--	544
80.MN106270.1_AgroatheliarolfsiistrainJ-12	509	--	509
81.OQ732628.1_AgroatheliarolfsiiisolateBTCBSr3	499	--	499
82.KY196185.1_ColletotrichumtruncatumstrainPAK53	468	--	468
83.GU935835.1_ColletotrichumcoccodesisolateC96002	1041	--	1041
84.MK085963.1_AlternariatenuissimaisolateSCCZ06	252	--	252

```
85.MT548042.1_AlternarialongipesstrainKY_2019_012                280 ---------------------------------------- 280
86.MN356465.1CalonectriamontanaisolateHSP4                       489 ---------------------------------------- 489
87.OL694224.1_CalonectriacanadianastrainF099                     504 ---------------------------------------- 504
88.MK803351.1_NeoscytalidiumdimidiatumstrainKale4-C              299 ---------------------------------------- 299
89.ON376993.1_CurvulariachiangmaiensisisolateND00J7              938 ---------------------------------------- 938
90.OQ383346.1_NeoscytalidiumdimidiatumisolateGKH-2               297 ---------------------------------------- 297
91.MF662595.1_NeoscytalidiumnovaehollandiaeisolateNeNo1          284 ---------------------------------------- 284
92.EF560588.1Melampsoralini                                      656 ---------------------------------------- 656
93.LC590862.1_NeoscytalidiumdimidiatumPSU-HP01TEF1               267 ---------------------------------------- 267
94.KX278106.1_BotryosphaeriaqingyuanensisstrainCERC2947          284 ---------------------------------------- 284
95.AJ578763.1_Blumeriagraminisf.sp.hordeicyp51                   938 ATGTGGCAATTAATGCGCTCTTCCTACAAAGATGGCACGC 977
96.MF490858.1_CurvulariadactyloctenIicolastrainCPC28810          930 ---------------------------------------- 930
97.KT287115.1_Bipolariscactivoraisolate3.8.6                    1006 ---------------------------------------- 1006
98.MT560940.1_CurvulariacactivorastrainHLGH0118                  963 ---------------------------------------- 963
99.OM714565.1_CurvulariaplantarumstrainM0134                     983 ---------------------------------------- 983
100.MN159911.1_BotrytiscinereaSICAUCC19-0003                     931 ---------------------------------------- 931
102.GU294713.1LasiodiplodiatheobromaestrainUCD2430TX             407 ---------------------------------------- 407
103.KX868094.1_Mycosphaerellasp.isolateCRM20.1                   475 ---------------------------------------- 475
104.LC599478.1Pseudocercosporapini-densifloraeMUCC534            310 ---------------------------------------- 310
105.N584698.1BipolarissetariaestrainKBS4-2                      1031 ---------------------------------------- 1031

2.OM160859.1_F.buharicum                                         560 ---------------------------------------- 560
1.LC727524.1_F.buharicum_OKI-1_Okura                             682 ---------------------------------------- 682
3.KX302919.1_F.sublunatum                                        521 ---------------------------------------- 521
4.LT996094.1_F.convolutans                                       516 ---------------------------------------- 516
5.OM160861.1_F.abutilonis                                        630 ---------------------------------------- 630
6.OM160874.1_F.guadeloupense                                     560 ---------------------------------------- 560
7.MH392475.1_F.graminearum                                       430 ---------------------------------------- 430
8.MH582420.1_F.solani                                            540 ---------------------------------------- 540
9.MAFF244605_F.oxysporum                                         544 ---------------------------------------- 544
10.MAFF237278_F.contaminatum_Hylocereus                          711 ---------------------------------------- 711
11.MAFF237649_F.concentricum__Riceroot                           678 ---------------------------------------- 678
12.MAFF237650_F.concentricum__Wheat                              679 ---------------------------------------- 679
13.MAFF239869_F.mangiferae__Ryukyupine                           657 ---------------------------------------- 657
14.MAFF240460_F.fujikuroi_Passionfruit                           661 ---------------------------------------- 661
15.MAFF241317_F.graminearum_Wheat                                666 ---------------------------------------- 666
16.MAFF242670_F.ipomoeae_Wheat                                   672 ---------------------------------------- 672
17.MAFF245129_F.concentricum_Fraxinus                            659 ---------------------------------------- 659
18.MAFF245395_F.cugenangense_Rhubarb                             671 ---------------------------------------- 671
19.MAFF246637_F.nirenbergiae_Strawberry                          671 ---------------------------------------- 671
20.MAFF246672_F.nirenbergiae_ChinesePeony                        671 ---------------------------------------- 671
21.MAFF246697_F.commune_Urallicoriceroot                         673 ---------------------------------------- 673
22.MAFF246729_F.falciforme_Angelica                              691 ---------------------------------------- 691
23.MAFF247220_F.duplospermum__Euwallaceasp                       694 ---------------------------------------- 694
24.MAFF410760_F.odoratissimum_alpha                              669 ---------------------------------------- 669
25.MAFF244605_FusariumoxysporumSchlechtendal_MAFF244605_Tomato   672 ---------------------------------------- 672
```

```
26.MAFF241326_F.asiaticum_Wheat                                        669 ---------------------------------------- 669
27.MAFF245014_F.asiaticum_Wildsoybean                                  666 ---------------------------------------- 666
28.MAFF150124_F.asiaticum__Wheat                                       666 ---------------------------------------- 666
29.OM135603.1F.algeriense                                              699 ---------------------------------------- 699
30.MAFF237465_F.penzigii_Aloe                                          704 ---------------------------------------- 704
31.MAFF103054_F.oxysporumSchlechtendalf.sp.cucumerinum_Cucumber        671 ---------------------------------------- 671
32.MAFF712246_F.oxysporumSchlechtendalf.sp.dianthi__Carnation          673 ---------------------------------------- 673
33.MAFF305558_F.oxysporumSchlechtendalf.sp.fragariae__Watermelon       671 ---------------------------------------- 671
34.MAFF744087_F.oxysporumSchlechtendalf.sp.lactucae__Lettuce           671 ---------------------------------------- 671
35.MAFF726924_F.oxysporumSchlechtendalf.sp.lagenariae_Whiteflorwedgourd 671 ---------------------------------------- 671
36.MAFF744003_F.oxysporumSchlechtendalf.sp.lagenariae_Squash)          672 ---------------------------------------- 672
37.MAFF305122_F.oxysporumSchlechtendalf.sp.melonis__Melon              671 ---------------------------------------- 671
38.MAFF306714_F.oxysporumSchlechtendalf.sp.momordicae_Balsampear       671 ---------------------------------------- 671
39.MAFF238905_F.oxysporumSchlechtendalf.sp.radicis-lycopersici_Tomato  674 ---------------------------------------- 674
40.MAFF150004_F.oxysporumSchlechtendalf.sp.spinaciae_Spinach           671 ---------------------------------------- 671
41.MAFF247034_F.oxysporumSchlechtendal__Goldenchain                    671 ---------------------------------------- 671
42.MAFF245747_F.oxysporumSchlechtendalf.sp.callistephi__Chinaaster     671 ---------------------------------------- 671
43.MAFF305115_FoxysporumSchlechtendalf.sp.batatas__Sweatpotato         703 ---------------------------------------- 703
44.MAFF150126_F.asiaticum_Seed                                         666 ---------------------------------------- 666
45.MAFF246738_F.solani_Angelica                                        703 ---------------------------------------- 703
46.MAFF246664_F.cugenangense_Perilla                                   671 ---------------------------------------- 671
47.MH582420.1F.solanistrainMRC256                                      679 ---------------------------------------- 679
48.MAFF240361_F.babinda_Soil                                           693 ---------------------------------------- 693
49.MAFF242368_F.azukicola_Azukibean                                    708 ---------------------------------------- 708
50.MAFF241312_F.asiaticum_Soil,welshonionfield                         666 ---------------------------------------- 666
51.LT548416.1_F.culmorumpartialtef1a                                   646 ---------------------------------------- 646
52.MAFF150124_F.asiaticum__Wheat                                       666 ---------------------------------------- 666
53.MAFF238806_F.begoniae_Oncidiumsp                                    705 ---------------------------------------- 705
54.MW594399.1_FusariumincarnatumisolateUD01C                           689 ---------------------------------------- 689
55.OP414923.1Pucciniagraminisf.sp.triticiisolateSHZPgt19               623 ---------------------------------------- 623
56.MT027094.1_BipolarisoryzaestrainOrL-2                               895 ---------------------------------------- 895
57.ON734360.1_AlternariaalternataisolateH126                           831 ---------------------------------------- 831
58.LC333578.1_StemphyliumlycopersiciSOasp2                             265 ---------------------------------------- 265
59.HQ718583.1_Colletotrichumgloeosporioidesisolateq-1                  567 ---------------------------------------- 567
60.JN241603.1_AtheliarolfsiiisolateSR1                                1013 ---------------------------------------- 1013
61.KJ866474.1_RhizoctoniasolanistrainMHL-1                             683 ---------------------------------------- 683
62.JQ672424.1AlternariatriticinaisolateEGS17-061                       825 ---------------------------------------- 825
63.LT707559.1_P.capsicipartialtef1agene                                964 ---------------------------------------- 964
64.MW090051.1_CurvularialunatastrainCls-3                              908 ---------------------------------------- 908
65.DQ400892.1_Aspergillusterreus                                       452 ---------------------------------------- 452
66.DQ911416.1_Pythiumsp.quercumstrainPy292                             932 ---------------------------------------- 932
67.EU797495.1_Phytophthorasp.oaksoilPoland                             930 ---------------------------------------- 930
68.HM148321.1_Cladosporiumcucumerinum                                  385 ---------------------------------------- 385
69.AF398888.1_SclerotiniasclerotiorumisolateSS1                        334 ---------------------------------------- 334
70.AF398888.1_S.sclerotiorumisolateSS1                                 334 ---------------------------------------- 334
71.HPAB545908.1_Verticilliumnonalfalfaeisolate                         442 ---------------------------------------- 442
```

```
72.EF433315.1_CeratocystisfimbriatavoucherCMW15052                    747 ---------------------------------------- 747
73.MN159912.1_Botrytiscinerea                                         931 ---------------------------------------- 931
74.MF034741.1_PeltasterfructicolaisolateSRB92                         317 ---------------------------------------- 317
75.LC440360.1_CercosporaasparagiCOasp2                                297 ---------------------------------------- 297
76.AY944105.1_MagnaportheoryzaeisolateSAG00T3()                       255 ---------------------------------------- 255
77.JX266586.1_CochliobolusmiyabeanusvoucherMFLUCC10-0733              918 ---------------------------------------- 918
78.MN393253.1_CorynesporacassiicolaisolateQHD001(MN393253.1UNVERIFIED) 305 ---------------------------------------- 305
79.MF375218.1_AgroatheliarolfsiiisolateBJB24                          544 ---------------------------------------- 544
80.MN106270.1_AgroatheliarolfsiistrainJ-12                            509 ---------------------------------------- 509
81.OQ732628.1_AgroatheliarolfsiiisolateBTCBSr3                        499 ---------------------------------------- 499
82.KY196185.1_ColletotrichumtruncatumstrainPAK53                      468 ---------------------------------------- 468
83.GU935835.1_ColletotrichumcoccodesisolateC96002                    1041 ---------------------------------------- 1041
84.MK085963.1_AlternariatenuissimaisolateSCCZ06                       252 ---------------------------------------- 252
85.MT548042.1_AlternarialongipesstrainKY_2019_012                     280 ---------------------------------------- 280
86.MN356465.1CalonectriamontanaisolateHSP4                            489 ---------------------------------------- 489
87.OL694224.1_CalonectriacanadianastrainF099                          504 ---------------------------------------- 504
88.MK803351.1_NeoscytalidiumdimidiatumstrainKale4-C                   299 ---------------------------------------- 299
89.ON376993.1_CurvulariachiangmaiensisisolateND00J7                   938 ---------------------------------------- 938
90.OQ383346.1_NeoscytalidiumdimidiatumisolateGKH-2                    297 ---------------------------------------- 297
91.MF662595.1_NeoscytalidiumnovaehollandiaeisolateNeNo1               284 ---------------------------------------- 284
92.EF560588.1Melampsoralini                                           656 ---------------------------------------- 656
93.LC590862.1_NeoscytalidiumdimidiatumPSU-HP01TEF1                    267 ---------------------------------------- 267
94.KX278106.1_BotryosphaeriaqingyuanensisstrainCERC2947               284 ---------------------------------------- 284
95.AJ578763.1_Blumeriagraminisf.sp.hordeicyp51                        978 CCGTACCGGATAAAGAGATTGCACACATGATGATCGCGCT 1017
96.MF490858.1_CurvulariadactylocteniicolastrainCPC28810               930 ---------------------------------------- 930
97.KT287115.1_Bipolariscactivoraisolate3.8.6                         1006 ---------------------------------------- 1006
98.MT560940.1_CurvulariacactivorastrainHLGH0118                       963 ---------------------------------------- 963
99.OM714565.1_CurvulariaplantarumstrainM0134                          983 ---------------------------------------- 983
100.MN159911.1_BotrytiscinereaSICAUCC19-0003                          931 ---------------------------------------- 931
102.GU294713.1LasiodiplodiatheobromaestrainUCD2430TX                  407 ---------------------------------------- 407
103.KX868094.1_Mycosphaerellasp.isolateCRM20.1                        475 ---------------------------------------- 475
104.LC599478.1Pseudocercosporapini-densifloraeMUCC534                 310 ---------------------------------------- 310
105.N584698.1BipolarissetariaestrainKBS4-2                           1031 ---------------------------------------- 1031

2.OM160859.1_F.buharicum                                              560 ---------------------------------------- 560
1.LC727524.1_F.buharicum_OKI-1_Okura                                  682 ---------------------------------------- 682
3.KX302919.1_F.sublunatum                                             521 ---------------------------------------- 521
4.LT996094.1_F.convolutans                                            516 ---------------------------------------- 516
5.OM160861.1_F.abutilonis                                             630 ---------------------------------------- 630
6.OM160874.1_F.guadeloupense                                          560 ---------------------------------------- 560
7.MH392475.1_F.graminearum                                            430 ---------------------------------------- 430
8.MH582420.1_F.solani                                                 540 ---------------------------------------- 540
9.MAFF244605_F.oxysporum                                              544 ---------------------------------------- 544
10.MAFF237278_F.contaminatum_Hylocereus                               711 ---------------------------------------- 711
11.MAFF237649_F.concentricum__Riceroot                                678 ---------------------------------------- 678
12.MAFF237650_F.concentricum__Wheat                                   679 ---------------------------------------- 679
```

13.MAFF239869_F.mangiferae__Ryukyupine	657	--	657
14.MAFF240460_F.fujikuroi_Passionfruit	661	--	661
15.MAFF241317_F.graminearum_Wheat	666	--	666
16.MAFF242670_F.ipomoeae_Wheat	672	--	672
17.MAFF245129_F.concentricum_Fraxinus	659	--	659
18.MAFF245395_F.cugenangense_Rhubarb	671	--	671
19.MAFF246637_F.nirenbergiae_Strawberry	671	--	671
20.MAFF246672_F.nirenbergiae_ChinesePeony	671	--	671
21.MAFF246697_F.commune_Urallicoriceroot	673	--	673
22.MAFF246729_F.falciforme_Angelica	691	--	691
23.MAFF247220_F.duplospermum__Euwallaceasp	694	--	694
24.MAFF410760_F.odoratissimum_alpha	669	--	669
25.MAFF244605_FusariumoxysporumSchlechtendal_MAFF244605_Tomato	672	--	672
26.MAFF241326_F.asiaticum_Wheat	669	--	669
27.MAFF245014_F.asiaticum_Wildsoybean	666	--	666
28.MAFF150124_F.asiaticum__Wheat	666	--	666
29.OM135603.1F.algeriense	699	--	699
30.MAFF237465_F.penzigii_Aloe	704	--	704
31.MAFF103054_F.oxysporumSchlechtendalf.sp.cucumerinum_Cucumber	671	--	671
32.MAFF712246_F.oxysporumSchlechtendalf.sp.dianthi__Carnation	673	--	673
33.MAFF305558_F.oxysporumSchlechtendalf.sp.fragariae__Watermelon	671	--	671
34.MAFF744087_F.oxysporumSchlechtendalf.sp.lactucae__Lettuce	671	--	671
35.MAFF726924_F.oxysporumSchlechtendalf.sp.lagenariae_Whitefloweredgourd	671	--	671
36.MAFF744003_F.oxysporumSchlechtendalf.sp.lagenariae_Squash)	672	--	672
37.MAFF305122_F.oxysporumSchlechtendalf.sp.melonis__Melon	671	--	671
38.MAFF306714_F.oxysporumSchlechtendalf.sp.momordicae_Balsampear	671	--	671
39.MAFF238905_F.oxysporumSchlechtendalf.sp.radicis-lycopersici_Tomato	674	--	674
40.MAFF150004_F.oxysporumSchlechtendalf.sp.spinaciae_Spinach	671	--	671
41.MAFF247034_F.oxysporumSchlechtendal__Goldenchain	671	--	671
42.MAFF245747_F.oxysporumSchlechtendalf.sp.callistephi__Chinaaster	671	--	671
43.MAFF305115_FoxysporumSchlechtendalf.sp.batatas__Sweatpotato	703	--	703
44.MAFF150126_F.asiaticum_Seed	666	--	666
45.MAFF246738_F.solani_Angelica	703	--	703
46.MAFF246664_F.cugenangense_Perilla	671	--	671
47.MH582420.1F.solanistrainMRC256	679	--	679
48.MAFF240361_F.babinda_Soil	693	--	693
49.MAFF242368_F.azukicola_Azukibean	708	--	708
50.MAFF241312_F.asiaticum_Soil,welshonionfield	666	--	666
51.LT548416.1_F.culmorumpartialtef1a	646	--	646
52.MAFF150124_F.asiaticum__Wheat	666	--	666
53.MAFF238806_F.begoniae_Oncidiumsp	705	--	705
54.MW594399.1_FusariumincarnatumisolateUD01C	689	--	689
55.OP414923.1Pucciniagraminisf.sp.triticiisolateSHZPgt19	623	--	623
56.MT027094.1_BipolarisoryzaestrainOrL-2	895	--	895
57.ON734360.1_AlternariaalternataisolateH126	831	--	831
58.LC333578.1_StemphyliumlycopersiciSOasp2	265	--	265

```
59.HQ718583.1_Colletotrichumgloeosporioidesisolateq-1                 567 ---------------------------------------- 567
60.JN241603.1_AtheliarolfsiiisolateSR1                               1013 ---------------------------------------- 1013
61.KJ866474.1_RhizoctoniasolanistrainMHL-1                            683 ---------------------------------------- 683
62.JQ672424.1AlternariatriticinaisolateEGS17-061                      825 ---------------------------------------- 825
63.LT707559.1_P.capsicipartialtef1agene                               964 ---------------------------------------- 964
64.MW090051.1_CurvularialunatastrainCls-3                             908 ---------------------------------------- 908
65.DQ400892.1_Aspergillusterreus                                      452 ---------------------------------------- 452
66.DQ911416.1_Pythiumsp.quercumstrainPy292                            932 ---------------------------------------- 932
67.EU797495.1_Phytophthorasp.oaksoilPoland                            930 ---------------------------------------- 930
68.HM148321.1_Cladosporiumcucumerinum                                 385 ---------------------------------------- 385
69.AF398888.1_SclerotiniasclerotiorumisolateSS1                       334 ---------------------------------------- 334
70.AF398888.1_S.sclerotiorumisolateSS1                                334 ---------------------------------------- 334
71.HPAB545908.1_Verticilliumnonalfalfaeisolate                        442 ---------------------------------------- 442
72.EF433315.1_CeratocystisfimbriatavoucherCMW15052                    747 ---------------------------------------- 747
73.MN159912.1_Botrytiscinerea                                         931 ---------------------------------------- 931
74.MF034741.1_PeltasterfructicolaisolateSRB92                         317 ---------------------------------------- 317
75.LC440360.1_CercosporaasparagiCOasp2                                297 ---------------------------------------- 297
76.AY944105.1_MagnaportheoryzaeisolateSAG00T3()                       255 ---------------------------------------- 255
77.JX266586.1_CochliobolusmiyabeanusvoucherMFLUCC10-0733              918 ---------------------------------------- 918
78.MN393253.1_CorynesporacassiicolaisolateQHD001(MN393253.1UNVERIFIED) 305 ---------------------------------------- 305
79.MF375218.1_AgroatheliarolfsiiisolateBJB24                          544 ---------------------------------------- 544
80.MN106270.1_AgroatheliarolfsiistrainJ-12                            509 ---------------------------------------- 509
81.OQ732628.1_AgroatheliarolfsiiisolateBTCBSr3                        499 ---------------------------------------- 499
82.KY196185.1_ColletotrichumtruncatumstrainPAK53                      468 ---------------------------------------- 468
83.GU935835.1_ColletotrichumcoccodesisolateC96002                    1041 ---------------------------------------- 1041
84.MK085963.1_AlternariatenuissimaisolateSCCZ06                       252 ---------------------------------------- 252
85.MT548042.1_AlternarialongipesstrainKY_2019_012                     280 ---------------------------------------- 280
86.MN356465.1CalonectriamontanaisolateHSP4                            489 ---------------------------------------- 489
87.OL694224.1_CalonectriacanadianastrainF099                          504 ---------------------------------------- 504
88.MK803351.1_NeoscytalidiumdimidiatumstrainKale4-C                   299 ---------------------------------------- 299
89.ON376993.1_CurvulariachiangmaiensisisolateND00J7                   938 ---------------------------------------- 938
90.OQ383346.1_NeoscytalidiumdimidiatumisolateGKH-2                    297 ---------------------------------------- 297
91.MF662595.1_NeoscytalidiumnovaehollandiaeisolateNeNo1               284 ---------------------------------------- 284
92.EF560588.1Melampsoralini                                           656 ---------------------------------------- 656
93.LC590862.1_NeoscytalidiumdimidiatumPSU-HP01TEF1                    267 ---------------------------------------- 267
94.KX278106.1_BotryosphaeriaqingyuanensisstrainCERC2947               284 ---------------------------------------- 284
95.AJ578763.1_Blumeriagraminisf.sp.hordeicyp51                       1018 CCTGATGGCTGGGCAACATTCTTCGTCGTCATCCAGCACA 1057
96.MF490858.1_CurvulariadactylocteniicolastrainCPC28810               930 ---------------------------------------- 930
97.KT287115.1_Bipolariscactivoraisolate3.8.6                         1006 ---------------------------------------- 1006
98.MT560940.1_CurvulariacactivorastrainHLGH0118                       963 ---------------------------------------- 963
99.OM714565.1_CurvulariaplantarumstrainM0134                          983 ---------------------------------------- 983
100.MN159911.1_BotrytiscinereaSICAUCC19-0003                          931 ---------------------------------------- 931
102.GU294713.1LasiodiplodiatheobromaestrainUCD2430TX                  407 ---------------------------------------- 407
103.KX868094.1_Mycosphaerellasp.isolateCRM20.1                        475 ---------------------------------------- 475
104.LC599478.1Pseudocercosporapini-densifloraeMUCC534                 310 ---------------------------------------- 310
105.N584698.1BipolarissetariaestrainKBS4-2                           1031 ---------------------------------------- 1031
```

```
2.OM160859.1_F.buharicum                                           560 ---------------------------------------- 560
1.LC727524.1_F.buharicum_OKI-1_Okura                               682 ---------------------------------------- 682
3.KX302919.1_F.sublunatum                                          521 ---------------------------------------- 521
4.LT996094.1_F.convolutans                                         516 ---------------------------------------- 516
5.OM160861.1_F.abutilonis                                          630 ---------------------------------------- 630
6.OM160874.1_F.guadeloupense                                       560 ---------------------------------------- 560
7.MH392475.1_F.graminearum                                         430 ---------------------------------------- 430
8.MH582420.1_F.solani                                              540 ---------------------------------------- 540
9.MAFF244605_F.oxysporum                                           544 ---------------------------------------- 544
10.MAFF237278_F.contaminatum_Hylocereus                            711 ---------------------------------------- 711
11.MAFF237649_F.concentricum__Riceroot                             678 ---------------------------------------- 678
12.MAFF237650_F.concentricum__Wheat                                679 ---------------------------------------- 679
13.MAFF239869_F.mangiferae__Ryukyupine                             657 ---------------------------------------- 657
14.MAFF240460_F.fujikuroi_Passionfruit                             661 ---------------------------------------- 661
15.MAFF241317_F.graminearum_Wheat                                  666 ---------------------------------------- 666
16.MAFF242670_F.ipomoeae_Wheat                                     672 ---------------------------------------- 672
17.MAFF245129_F.concentricum_Fraxinus                              659 ---------------------------------------- 659
18.MAFF245395_F.cugenangense_Rhubarb                               671 ---------------------------------------- 671
19.MAFF246637_F.nirenbergiae_Strawberry                            671 ---------------------------------------- 671
20.MAFF246672_F.nirenbergiae_ChinesePeony                          671 ---------------------------------------- 671
21.MAFF246697_F.commune_Urallicoriceroot                           673 ---------------------------------------- 673
22.MAFF246729_F.falciforme_Angelica                                691 ---------------------------------------- 691
23.MAFF247220_F.duplospermum__Euwallaceasp                         694 ---------------------------------------- 694
24.MAFF410760_F.odoratissimum_alpha                                669 ---------------------------------------- 669
25.MAFF244605_FusariumoxysporumSchlechtendal_MAFF244605_Tomato     672 ---------------------------------------- 672
26.MAFF241326_F.asiaticum_Wheat                                    669 ---------------------------------------- 669
27.MAFF245014_F.asiaticum_Wildsoybean                              666 ---------------------------------------- 666
28.MAFF150124_F.asiaticum__Wheat                                   666 ---------------------------------------- 666
29.OM135603.1F.algeriense                                          699 ---------------------------------------- 699
30.MAFF237465_F.penzigii_Aloe                                      704 ---------------------------------------- 704
31.MAFF103054_F.oxysporumSchlechtendalf.sp.cucumerinum_Cucumber    671 ---------------------------------------- 671
32.MAFF712246_F.oxysporumSchlechtendalf.sp.dianthi__Carnation      673 ---------------------------------------- 673
33.MAFF305558_F.oxysporumSchlechtendalf.sp.fragariae__Watermelon   671 ---------------------------------------- 671
34.MAFF744087_F.oxysporumSchlechtendalf.sp.lactucae__Lettuce       671 ---------------------------------------- 671
35.MAFF726924_F.oxysporumSchlechtendalf.sp.lagenariae_Whitefloweredgourd 671 ---------------------------------------- 671
36.MAFF744003_F.oxysporumSchlechtendalf.sp.lagenariae_Squash)      672 ---------------------------------------- 672
37.MAFF305122_F.oxysporumSchlechtendalf.sp.melonis__Melon          671 ---------------------------------------- 671
38.MAFF306714_F.oxysporumSchlechtendalf.sp.momordicae_Balsampear   671 ---------------------------------------- 671
39.MAFF238905_F.oxysporumSchlechtendalf.sp.radicis-lycopersici_Tomato 674 ---------------------------------------- 674
40.MAFF150004_F.oxysporumSchlechtendalf.sp.spinaciae_Spinach       671 ---------------------------------------- 671
41.MAFF247034_F.oxysporumSchlechtendal__Goldenchain                671 ---------------------------------------- 671
42.MAFF245747_F.oxysporumSchlechtendalf.sp.callistephi__Chinaaster 671 ---------------------------------------- 671
43.MAFF305115_FoxysporumSchlechtendalf.sp.batatas__Sweatpotato     703 ---------------------------------------- 703
44.MAFF150126_F.asiaticum_Seed                                     666 ---------------------------------------- 666
45.MAFF246738_F.solani_Angelica                                    703 ---------------------------------------- 703
46.MAFF246664_F.cugenangense_Perilla                               671 ---------------------------------------- 671
```

```
47.MH582420.1F.solanistrainMRC256                                        679 ---------------------------------------- 679
48.MAFF240361_F.babinda_Soil                                             693 ---------------------------------------- 693
49.MAFF242368_F.azukicola_Azukibean                                      708 ---------------------------------------- 708
50.MAFF241312_F.asiaticum_Soil,welshonionfield                           666 ---------------------------------------- 666
51.LT548416.1_F.culmorumpartialtef1a                                     646 ---------------------------------------- 646
52.MAFF150124_F.asiaticum__Wheat                                         666 ---------------------------------------- 666
53.MAFF238806_F.begoniae_Oncidiumsp                                      705 ---------------------------------------- 705
54.MW594399.1_FusariumincarnatumisolateUD01C                             689 ---------------------------------------- 689
55.OP414923.1Pucciniagraminisf.sp.triticiisolateSHZPgt19                 623 ---------------------------------------- 623
56.MT027094.1_BipolarisoryzaestrainOrL-2                                 895 ---------------------------------------- 895
57.ON734360.1_AlternariaalternataisolateH126                             831 ---------------------------------------- 831
58.LC333578.1_StemphyliumlycopersiciSOasp2                               265 ---------------------------------------- 265
59.HQ718583.1_Colletotrichumgloeosporioidesisolateq-1                    567 ---------------------------------------- 567
60.JN241603.1_AtheliarolfsiiisolateSR1                                  1013 ---------------------------------------- 1013
61.KJ866474.1_RhizoctoniasolanistrainMHL-1                               683 ---------------------------------------- 683
62.JQ672424.1AlternariatriticinaisolateEGS17-061                         825 ---------------------------------------- 825
63.LT707559.1_P.capsicipartialtef1agene                                  964 ---------------------------------------- 964
64.MW090051.1_CurvularialunatastrainCls-3                                908 ---------------------------------------- 908
65.DQ400892.1_Aspergillusterreus                                         452 ---------------------------------------- 452
66.DQ911416.1_Pythiumsp.quercumstrainPy292                               932 ---------------------------------------- 932
67.EU797495.1_Phytophthorasp.oaksoilPoland                               930 ---------------------------------------- 930
68.HM148321.1_Cladosporiumcucumerinum                                    385 ---------------------------------------- 385
69.AF398888.1_SclerotiniasclerotiorumisolateSS1                          334 ---------------------------------------- 334
70.AF398888.1_S.sclerotiorumisolateSS1                                   334 ---------------------------------------- 334
71.HPAB545908.1_Verticilliumnonalfalfaeisolate                           442 ---------------------------------------- 442
72.EF433315.1_CeratocystisfimbriatavoucherCMW15052                       747 ---------------------------------------- 747
73.MN159912.1_Botrytiscinerea                                            931 ---------------------------------------- 931
74.MF034741.1_PeltasterfructicolaisolateSRB92                            317 ---------------------------------------- 317
75.LC440360.1_CercosporaasparagiCOasp2                                   297 ---------------------------------------- 297
76.AY944105.1_MagnaportheoryzaeisolateSAG00T3()                          255 ---------------------------------------- 255
77.JX266586.1_CochliobolusmiyabeanusvoucherMFLUCC10-0733                 918 ---------------------------------------- 918
78.MN393253.1_CorynesporacassiicolaisolateQHD001(MN393253.1UNVERIFIED)   305 ---------------------------------------- 305
79.MF375218.1_AgroatheliarolfsiiisolateBJB24                             544 ---------------------------------------- 544
80.MN106270.1_AgroatheliarolfsiistrainJ-12                               509 ---------------------------------------- 509
81.OQ732628.1_AgroatheliarolfsiiisolateBTCBSr3                           499 ---------------------------------------- 499
82.KY196185.1_ColletotrichumtruncatumstrainPAK53                         468 ---------------------------------------- 468
83.GU935835.1_ColletotrichumcoccodesisolateC96002                       1041 ---------------------------------------- 1041
84.MK085963.1_AlternariatenuissimaisolateSCCZ06                          252 ---------------------------------------- 252
85.MT548042.1_AlternarialongipesstrainKY_2019_012                        280 ---------------------------------------- 280
86.MN356465.1CalonectriamontanaisolateHSP4                               489 ---------------------------------------- 489
87.OL694224.1_CalonectriacanadianastrainF099                             504 ---------------------------------------- 504
88.MK803351.1_NeoscytalidiumdimidiatumstrainKale4-C                      299 ---------------------------------------- 299
89.ON376993.1_CurvulariachiangmaiensisisolateND00J7                      938 ---------------------------------------- 938
90.OQ383346.1_NeoscytalidiumdimidiatumisolateGKH-2                       297 ---------------------------------------- 297
91.MF662595.1_NeoscytalidiumnovaehollandiaeisolateNeNo1                  284 ---------------------------------------- 284
92.EF560588.1Melampsoralini                                              656 ---------------------------------------- 656
```

```
93.LC590862.1_NeoscytalidiumdimidiatumPSU-HP01TEF1              267 ---------------------------------------- 267
94.KX278106.1_BotryosphaeriaqingyuanensisstrainCERC2947         284 ---------------------------------------- 284
95.AJ578763.1_Blumeriagraminisf.sp.hordeicyp51                 1058 TGGATCATGCTGTGGCTTGCTGCTCGACCAGACATCACTG 1097
96.MF490858.1_CurvulariadactylocteniicolastrainCPC28810         930 ---------------------------------------- 930
97.KT287115.1_Bipolariscactivoraisolate3.8.6                   1006 ---------------------------------------- 1006
98.MT560940.1_CurvulariacactivorastrainHLGH0118                 963 ---------------------------------------- 963
99.OM714565.1_CurvulariaplantarumstrainM0134                    983 ---------------------------------------- 983
100.MN159911.1_BotrytiscinereaSICAUCC19-0003                    931 ---------------------------------------- 931
102.GU294713.1LasiodiplodiatheobromaestrainUCD2430TX            407 ---------------------------------------- 407
103.KX868094.1_Mycosphaerellasp.isolateCRM20.1                  475 ---------------------------------------- 475
104.LC599478.1Pseudocercosporapini-densifloraeMUCC534           310 ---------------------------------------- 310
105.N584698.1BipolarissetariaestrainKBS4-2                     1031 ---------------------------------------- 1031

2.OM160859.1_F.buharicum                                         560 ---------------------------------------- 560
1.LC727524.1_F.buharicum_OKI-1_Okura                             682 ---------------------------------------- 682
3.KX302919.1_F.sublunatum                                        521 ---------------------------------------- 521
4.LT996094.1_F.convolutans                                       516 ---------------------------------------- 516
5.OM160861.1_F.abutilonis                                        630 ---------------------------------------- 630
6.OM160874.1_F.guadeloupense                                     560 ---------------------------------------- 560
7.MH392475.1_F.graminearum                                       430 ---------------------------------------- 430
8.MH582420.1_F.solani                                            540 ---------------------------------------- 540
9.MAFF244605_F.oxysporum                                         544 ---------------------------------------- 544
10.MAFF237278_F.contaminatum_Hylocereus                          711 ---------------------------------------- 711
11.MAFF237649_F.concentricum__Riceroot                           678 ---------------------------------------- 678
12.MAFF237650_F.concentricum__Wheat                              679 ---------------------------------------- 679
13.MAFF239869_F.mangiferae__Ryukyupine                           657 ---------------------------------------- 657
14.MAFF240460_F.fujikuroi_Passionfruit                           661 ---------------------------------------- 661
15.MAFF241317_F.graminearum_Wheat                                666 ---------------------------------------- 666
16.MAFF242670_F.ipomoeae_Wheat                                   672 ---------------------------------------- 672
17.MAFF245129_F.concentricum_Fraxinus                            659 ---------------------------------------- 659
18.MAFF245395_F.cugenangense_Rhubarb                             671 ---------------------------------------- 671
19.MAFF246637_F.nirenbergiae_Strawberry                          671 ---------------------------------------- 671
20.MAFF246672_F.nirenbergiae_ChinesePeony                        671 ---------------------------------------- 671
21.MAFF246697_F.commune_Urallicoriceroot                         673 ---------------------------------------- 673
22.MAFF246729_F.falciforme_Angelica                              691 ---------------------------------------- 691
23.MAFF247220_F.duplospermum__Euwallaceasp                       694 ---------------------------------------- 694
24.MAFF410760_F.odoratissimum_alpha                              669 ---------------------------------------- 669
25.MAFF244605_FusariumoxysporumSchlechtendal_MAFF244605_Tomato   672 ---------------------------------------- 672
26.MAFF241326_F.asiaticum_Wheat                                  669 ---------------------------------------- 669
27.MAFF245014_F.asiaticum_Wildsoybean                            666 ---------------------------------------- 666
28.MAFF150124_F.asiaticum__Wheat                                 666 ---------------------------------------- 666
29.OM135603.1F.algeriense                                        699 ---------------------------------------- 699
30.MAFF237465_F.penzigii_Aloe                                    704 ---------------------------------------- 704
31.MAFF103054_F.oxysporumSchlechtendalf.sp.cucumerinum_Cucumber  671 ---------------------------------------- 671
32.MAFF712246_F.oxysporumSchlechtendalf.sp.dianthi__Carnation    673 ---------------------------------------- 673
33.MAFF305558_F.oxysporumSchlechtendalf.sp.fragariae__Watermelon 671 ---------------------------------------- 671
```

```
34.MAFF744087_F.oxysporumSchlechtendalf.sp.lactucae__Lettuce                671 ---------------------------------------- 671
35.MAFF726924_F.oxysporumSchlechtendalf.sp.lagenariae_Whiteflowneredgourd   671 ---------------------------------------- 671
36.MAFF744003_F.oxysporumSchlechtendalf.sp.lagenariae_Squash)               672 ---------------------------------------- 672
37.MAFF305122_F.oxysporumSchlechtendalf.sp.melonis__Melon                   671 ---------------------------------------- 671
38.MAFF306714_F.oxysporumSchlechtendalf.sp.momordicae_Balsampear            671 ---------------------------------------- 671
39.MAFF238905_F.oxysporumSchlechtendalf.sp.radicis-lycopersici_Tomato       674 ---------------------------------------- 674
40.MAFF150004_F.oxysporumSchlechtendalf.sp.spinaciae_Spinach                671 ---------------------------------------- 671
41.MAFF247034_F.oxysporumSchlechtendal__Goldenchain                         671 ---------------------------------------- 671
42.MAFF245747_F.oxysporumSchlechtendalf.sp.callistephi__Chinaaster          671 ---------------------------------------- 671
43.MAFF305115_FoxysporumSchlechtendalf.sp.batatas__Sweatpotato              703 ---------------------------------------- 703
44.MAFF150126_F.asiaticum_Seed                                              666 ---------------------------------------- 666
45.MAFF246738_F.solani_Angelica                                             703 ---------------------------------------- 703
46.MAFF246664_F.cugenangense_Perilla                                        671 ---------------------------------------- 671
47.MH582420.1F.solanistrainMRC256                                           679 ---------------------------------------- 679
48.MAFF240361_F.babinda_Soil                                                693 ---------------------------------------- 693
49.MAFF242368_F.azukicola_Azukibean                                         708 ---------------------------------------- 708
50.MAFF241312_F.asiaticum_Soil,welshonionfield                              666 ---------------------------------------- 666
51.LT548416.1_F.culmorumpartialtef1a                                        646 ---------------------------------------- 646
52.MAFF150124_F.asiaticum__Wheat                                            666 ---------------------------------------- 666
53.MAFF238806_F.begoniae_Oncidiumsp                                         705 ---------------------------------------- 705
54.MW594399.1_FusariumincarnatumisolateUD01C                                689 ---------------------------------------- 689
55.OP414923.1Pucciniagraminisf.sp.triticiisolateSHZPgt19                    623 ---------------------------------------- 623
56.MT027094.1_BipolarisoryzaestrainOrL-2                                    895 ---------------------------------------- 895
57.ON734360.1_AlternariaalternataisolateH126                                831 ---------------------------------------- 831
58.LC333578.1_StemphyliumlycopersiciSOasp2                                  265 ---------------------------------------- 265
59.HQ718583.1_Colletotrichumgloeosporioidesisolateq-1                       567 ---------------------------------------- 567
60.JN241603.1_AtheliarolfsiiisolateSR1                                     1013 ---------------------------------------- 1013
61.KJ866474.1_RhizoctoniasolanistrainMHL-1                                  683 ---------------------------------------- 683
62.JQ672424.1AlternariatriticinaisolateEGS17-061                            825 ---------------------------------------- 825
63.LT707559.1_P.capsicipartialtef1agene                                     964 ---------------------------------------- 964
64.MW090051.1_CurvularialunatastrainCls-3                                   908 ---------------------------------------- 908
65.DQ400892.1_Aspergillusterreus                                            452 ---------------------------------------- 452
66.DQ911416.1_Pythiumsp.quercumstrainPy292                                  932 ---------------------------------------- 932
67.EU797495.1_Phytophthorasp.oaksoilPoland                                  930 ---------------------------------------- 930
68.HM148321.1_Cladosporiumcucumerinum                                       385 ---------------------------------------- 385
69.AF398888.1_SclerotiniasclerotiorumisolateSS1                             334 ---------------------------------------- 334
70.AF398888.1_S.sclerotiorumisolateSS1                                      334 ---------------------------------------- 334
71.HPAB545908.1_Verticilliumnonalfalfaeisolate                              442 ---------------------------------------- 442
72.EF433315.1_CeratocystisfimbriatavoucherCMW15052                          747 ---------------------------------------- 747
73.MN159912.1_Botrytiscinerea                                               931 ---------------------------------------- 931
74.MF034741.1_PeltasterfructicolaisolateSRB92                               317 ---------------------------------------- 317
75.LC440360.1_CercosporaasparagiCOasp2                                      297 ---------------------------------------- 297
76.AY944105.1_MagnaportheoryzaeisolateSAG00T3()                             255 ---------------------------------------- 255
77.JX266586.1_CochliobolusmiyabeanusvoucherMFLUCC10-0733                    918 ---------------------------------------- 918
78.MN393253.1_CorynesporacassiicolaisolateQHD001(MN393253.1UNVERIFIED)      305 ---------------------------------------- 305
79.MF375218.1_AgroatheliarolfsiiisolateBJB24                                544 ---------------------------------------- 544
```

80.MN106270.1_AgroatheliarolfsiistrainJ-12	509	--	509
81.OQ732628.1_AgroatheliarolfsiiisolateBTCBSr3	499	--	499
82.KY196185.1_ColletotrichumtruncatumstrainPAK53	468	--	468
83.GU935835.1_ColletotrichumcoccodesisolateC96002	1041	--	1041
84.MK085963.1_AlternariatenuissimaisolateSCCZ06	252	--	252
85.MT548042.1_AlternarialongipesstrainKY_2019_012	280	--	280
86.MN356465.1CalonectriamontanaisolateHSP4	489	--	489
87.OL694224.1_CalonectriacanadianastrainF099	504	--	504
88.MK803351.1_NeoscytalidiumdimidiatumstrainKale4-C	299	--	299
89.ON376993.1_CurvulariachiangmaiensisisolateND00J7	938	--	938
90.OQ383346.1_NeoscytalidiumdimidiatumisolateGKH-2	297	--	297
91.MF662595.1_NeoscytalidiumnovaehollandiaeisolateNeNo1	284	--	284
92.EF560588.1Melampsoralini	656	--	656
93.LC590862.1_NeoscytalidiumdimidiatumPSU-HP01TEF1	267	--	267
94.KX278106.1_BotryosphaeriaqingyuanensisstrainCERC2947	284	--	284
95.AJ578763.1_Blumeriagraminisf.sp.hordeicyp51	1098	AAGAACTCTACCAAGAACAATTAGAATTATTGGGCTCAGA	1137
96.MF490858.1_CurvulariadactylocteniicolastrainCPC28810	930	--	930
97.KT287115.1_Bipolariscactivoraisolate3.8.6	1006	--	1006
98.MT560940.1_CurvulariacactivorastrainHLGH0118	963	--	963
99.OM714565.1_CurvulariaplantarumstrainM0134	983	--	983
100.MN159911.1_BotrytiscinereaSICAUCC19-0003	931	--	931
102.GU294713.1LasiodiplodiatheobromaestrainUCD2430TX	407	--	407
103.KX868094.1_Mycosphaerellasp.isolateCRM20.1	475	--	475
104.LC599478.1Pseudocercosporapini-densifloraeMUCC534	310	--	310
105.N584698.1BipolarissetariaestrainKBS4-2	1031	--	1031

2.OM160859.1_F.buharicum	560	--	560
1.LC727524.1_F.buharicum_OKI-1_Okura	682	--	682
3.KX302919.1_F.sublunatum	521	--	521
4.LT996094.1_F.convolutans	516	--	516
5.OM160861.1_F.abutilonis	630	--	630
6.OM160874.1_F.guadeloupense	560	--	560
7.MH392475.1_F.graminearum	430	--	430
8.MH582420.1_F.solani	540	--	540
9.MAFF244605_F.oxysporum	544	--	544
10.MAFF237278_F.contaminatum_Hylocereus	711	--	711
11.MAFF237649_F.concentricum__Riceroot	678	--	678
12.MAFF237650_F.concentricum__Wheat	679	--	679
13.MAFF239869_F.mangiferae__Ryukyupine	657	--	657
14.MAFF240460_F.fujikuroi_Passionfruit	661	--	661
15.MAFF241317_F.graminearum_Wheat	666	--	666
16.MAFF242670_F.ipomoeae_Wheat	672	--	672
17.MAFF245129_F.concentricum_Fraxinus	659	--	659
18.MAFF245395_F.cugenangense_Rhubarb	671	--	671
19.MAFF246637_F.nirenbergiae_Strawberry	671	--	671
20.MAFF246672_F.nirenbergiae_ChinesePeony	671	--	671

21.MAFF246697_F.commune_Urallicoriceroot	673	--	673
22.MAFF246729_F.falciforme_Angelica	691	--	691
23.MAFF247220_F.duplospermum__Euwallaceasp	694	--	694
24.MAFF410760_F.odoratissimum_alpha	669	--	669
25.MAFF244605_FusariumoxysporumSchlechtendal_MAFF244605_Tomato	672	--	672
26.MAFF241326_F.asiaticum_Wheat	669	--	669
27.MAFF245014_F.asiaticum_Wildsoybean	666	--	666
28.MAFF150124_F.asiaticum__Wheat	666	--	666
29.OM135603.1F.algeriense	699	--	699
30.MAFF237465_F.penzigii_Aloe	704	--	704
31.MAFF103054_F.oxysporumSchlechtendalf.sp.cucumerinum_Cucumber	671	--	671
32.MAFF712246_F.oxysporumSchlechtendalf.sp.dianthi__Carnation	673	--	673
33.MAFF305558_F.oxysporumSchlechtendalf.sp.fragariae__Watermelon	671	--	671
34.MAFF744087_F.oxysporumSchlechtendalf.sp.lactucae__Lettuce	671	--	671
35.MAFF726924_F.oxysporumSchlechtendalf.sp.lagenariae_Whiteflowered gourd	671	--	671
36.MAFF744003_F.oxysporumSchlechtendalf.sp.lagenariae_Squash)	672	--	672
37.MAFF305122_F.oxysporumSchlechtendalf.sp.melonis__Melon	671	--	671
38.MAFF306714_F.oxysporumSchlechtendalf.sp.momordicae_Balsampear	671	--	671
39.MAFF238905_F.oxysporumSchlechtendalf.sp.radicis-lycopersici_Tomato	674	--	674
40.MAFF150004_F.oxysporumSchlechtendalf.sp.spinaciae_Spinach	671	--	671
41.MAFF247034_F.oxysporumSchlechtendal__Goldenchain	671	--	671
42.MAFF245747_F.oxysporumSchlechtendalf.sp.callistephi__Chinaaster	671	--	671
43.MAFF305115_FoxysporumSchlechtendalf.sp.batatas__Sweatpotato	703	--	703
44.MAFF150126_F.asiaticum_Seed	666	--	666
45.MAFF246738_F.solani_Angelica	703	--	703
46.MAFF246664_F.cugenangense_Perilla	671	--	671
47.MH582420.1F.solanistrainMRC256	679	--	679
48.MAFF240361_F.babinda_Soil	693	--	693
49.MAFF242368_F.azukicola_Azukibean	708	--	708
50.MAFF241312_F.asiaticum_Soil,welshonionfield	666	--	666
51.LT548416.1_F.culmorumpartialtef1a	646	--	646
52.MAFF150124_F.asiaticum__Wheat	666	--	666
53.MAFF238806_F.begoniae_Oncidiumsp	705	--	705
54.MW594399.1_FusariumincarnatumisolateUD01C	689	--	689
55.OP414923.1Pucciniagraminisf.sp.triticiisolateSHZPgt19	623	--	623
56.MT027094.1_BipolarisoryzaestrainOrL-2	895	--	895
57.ON734360.1_AlternariaalternataisolateH126	831	--	831
58.LC333578.1_StemphyliumlycopersiciSOasp2	265	--	265
59.HQ718583.1_Colletotrichumgloeosporioidesisolateq-1	567	--	567
60.JN241603.1_AtheliarolfsiiisolateSR1	1013	--	1013
61.KJ866474.1_RhizoctoniasolanistrainMHL-1	683	--	683
62.JQ672424.1AlternariatriticinaisolateEGS17-061	825	--	825
63.LT707559.1_P.capsicipartialtef1agene	964	--	964
64.MW090051.1_CurvularialunatastrainCls-3	908	--	908
65.DQ400892.1_Aspergillusterreus	452	--	452
66.DQ911416.1_Pythiumsp.quercumstrainPy292	932	--	932

```
67.EU797495.1_Phytophthorasp.oaksoilPoland                              930 ---------------------------------------- 930
68.HM148321.1_Cladosporiumcucumerinum                                   385 ---------------------------------------- 385
69.AF398888.1_SclerotiniasclerotiorumisolateSS1                         334 ---------------------------------------- 334
70.AF398888.1_S.sclerotiorumisolateSS1                                  334 ---------------------------------------- 334
71.HPAB545908.1_Verticilliumnonalfalfaeisolate                          442 ---------------------------------------- 442
72.EF433315.1_CeratocystisfimbriatavoucherCMW15052                      747 ---------------------------------------- 747
73.MN159912.1_Botrytiscinerea                                           931 ---------------------------------------- 931
74.MF034741.1_PeltasterfructicolaisolateSRB92                           317 ---------------------------------------- 317
75.LC440360.1_CercosporaasparagiCOasp2                                  297 ---------------------------------------- 297
76.AY944105.1_MagnaportheoryzaeisolateSAG00T3()                         255 ---------------------------------------- 255
77.JX266586.1_CochliobolusmiyabeanusvoucherMFLUCC10-0733                918 ---------------------------------------- 918
78.MN393253.1_CorynesporacassiicolaisolateQHD001(MN393253.1UNVERIFIED)  305 ---------------------------------------- 305
79.MF375218.1_AgroatheliarolfsiiisolateBJB24                            544 ---------------------------------------- 544
80.MN106270.1_AgroatheliarolfsiistrainJ-12                              509 ---------------------------------------- 509
81.OQ732628.1_AgroatheliarolfsiiisolateBTCBSr3                          499 ---------------------------------------- 499
82.KY196185.1_ColletotrichumtruncatumstrainPAK53                        468 ---------------------------------------- 468
83.GU935835.1_ColletotrichumcoccodesisolateC96002                      1041 ---------------------------------------- 1041
84.MK085963.1_AlternariatenuissimaisolateSCCZ06                         252 ---------------------------------------- 252
85.MT548042.1_AlternarialongipesstrainKY_2019_012                       280 ---------------------------------------- 280
86.MN356465.1CalonectriamontanaisolateHSP4                              489 ---------------------------------------- 489
87.OL694224.1_CalonectriacanadianastrainF099                            504 ---------------------------------------- 504
88.MK803351.1_NeoscytalidiumdimidiatumstrainKale4-C                     299 ---------------------------------------- 299
89.ON376993.1_CurvulariachiangmaiensisisolateND00J7                     938 ---------------------------------------- 938
90.OQ383346.1_NeoscytalidiumdimidiatumisolateGKH-2                      297 ---------------------------------------- 297
91.MF662595.1_NeoscytalidiumnovaehollandiaeisolateNeNo1                 284 ---------------------------------------- 284
92.EF560588.1Melampsoralini                                             656 ---------------------------------------- 656
93.LC590862.1_NeoscytalidiumdimidiatumPSU-HP01TEF1                      267 ---------------------------------------- 267
94.KX278106.1_BotryosphaeriaqingyuanensisstrainCERC2947                 284 ---------------------------------------- 284
95.AJ578763.1_Blumeriagraminisf.sp.hordeicyp51                         1138 ATTACCCCCTCTCAAATATGAAGATCTCTCGAAACTTTCT 1177
96.MF490858.1_CurvulariadactylocteniicolastrainCPC28810                 930 ---------------------------------------- 930
97.KT287115.1_Bipolariscactivoraisolate3.8.6                           1006 ---------------------------------------- 1006
98.MT560940.1_CurvulariacactivorastrainHLGH0118                         963 ---------------------------------------- 963
99.OM714565.1_CurvulariaplantarumstrainM0134                            983 ---------------------------------------- 983
100.MN159911.1_BotrytiscinereaSICAUCC19-0003                            931 ---------------------------------------- 931
102.GU294713.1LasiodiplodiatheobromaestrainUCD2430TX                    407 ---------------------------------------- 407
103.KX868094.1_Mycosphaerellasp.isolateCRM20.1                          475 ---------------------------------------- 475
104.LC599478.1Pseudocercosporapini-densifloraeMUCC534                   310 ---------------------------------------- 310
105.N584698.1BipolarissetariaestrainKBS4-2                             1031 ---------------------------------------- 1031

2.OM160859.1_F.buharicum                                                560 ---------------------------------------- 560
1.LC727524.1_F.buharicum_OKI-1_Okura                                    682 ---------------------------------------- 682
3.KX302919.1_F.sublunatum                                               521 ---------------------------------------- 521
4.LT996094.1_F.convolutans                                              516 ---------------------------------------- 516
5.OM160861.1_F.abutilonis                                               630 ---------------------------------------- 630
6.OM160874.1_F.guadeloupense                                            560 ---------------------------------------- 560
7.MH392475.1_F.graminearum                                              430 ---------------------------------------- 430
```

```
8.MH582420.1_F.solani                                                      540 ---------------------------------------- 540
9.MAFF244605_F.oxysporum                                                   544 ---------------------------------------- 544
10.MAFF237278_F.contaminatum_Hylocereus                                    711 ---------------------------------------- 711
11.MAFF237649_F.concentricum__Riceroot                                     678 ---------------------------------------- 678
12.MAFF237650_F.concentricum__Wheat                                        679 ---------------------------------------- 679
13.MAFF239869_F.mangiferae__Ryukyupine                                     657 ---------------------------------------- 657
14.MAFF240460_F.fujikuroi_Passionfruit                                     661 ---------------------------------------- 661
15.MAFF241317_F.graminearum_Wheat                                          666 ---------------------------------------- 666
16.MAFF242670_F.ipomoeae_Wheat                                             672 ---------------------------------------- 672
17.MAFF245129_F.concentricum_Fraxinus                                      659 ---------------------------------------- 659
18.MAFF245395_F.cugenangense_Rhubarb                                       671 ---------------------------------------- 671
19.MAFF246637_F.nirenbergiae_Strawberry                                    671 ---------------------------------------- 671
20.MAFF246672_F.nirenbergiae_ChinesePeony                                  671 ---------------------------------------- 671
21.MAFF246697_F.commune_Urallicoriceroot                                   673 ---------------------------------------- 673
22.MAFF246729_F.falciforme_Angelica                                        691 ---------------------------------------- 691
23.MAFF247220_F.duplospermum__Euwallaceasp                                 694 ---------------------------------------- 694
24.MAFF410760_F.odoratissimum_alpha                                        669 ---------------------------------------- 669
25.MAFF244605_FusariumoxysporumSchlechtendal_MAFF244605_Tomato             672 ---------------------------------------- 672
26.MAFF241326_F.asiaticum_Wheat                                            669 ---------------------------------------- 669
27.MAFF245014_F.asiaticum_Wildsoybean                                      666 ---------------------------------------- 666
28.MAFF150124_F.asiaticum__Wheat                                           666 ---------------------------------------- 666
29.OM135603.1F.algeriense                                                  699 ---------------------------------------- 699
30.MAFF237465_F.penzigii_Aloe                                              704 ---------------------------------------- 704
31.MAFF103054_F.oxysporumSchlechtendalf.sp.cucumerinum_Cucumber            671 ---------------------------------------- 671
32.MAFF712246_F.oxysporumSchlechtendalf.sp.dianthi__Carnation              673 ---------------------------------------- 673
33.MAFF305558_F.oxysporumSchlechtendalf.sp.fragariae__Watermelon           671 ---------------------------------------- 671
34.MAFF744087_F.oxysporumSchlechtendalf.sp.lactucae__Lettuce               671 ---------------------------------------- 671
35.MAFF726924_F.oxysporumSchlechtendalf.sp.lagenariae_Whitefloweredgourd   671 ---------------------------------------- 671
36.MAFF744003_F.oxysporumSchlechtendalf.sp.lagenariae_Squash)              672 ---------------------------------------- 672
37.MAFF305122_F.oxysporumSchlechtendalf.sp.melonis__Melon                  671 ---------------------------------------- 671
38.MAFF306714_F.oxysporumSchlechtendalf.sp.momordicae_Balsampear           671 ---------------------------------------- 671
39.MAFF238905_F.oxysporumSchlechtendalf.sp.radicis-lycopersici_Tomato      674 ---------------------------------------- 674
40.MAFF150004_F.oxysporumSchlechtendalf.sp.spinaciae_Spinach               671 ---------------------------------------- 671
41.MAFF247034_F.oxysporumSchlechtendal__Goldenchain                        671 ---------------------------------------- 671
42.MAFF245747_F.oxysporumSchlechtendalf.sp.callistephi__Chinaaster         671 ---------------------------------------- 671
43.MAFF305115_FoxysporumSchlechtendalf.sp.batatas__Sweatpotato             703 ---------------------------------------- 703
44.MAFF150126_F.asiaticum_Seed                                             666 ---------------------------------------- 666
45.MAFF246738_F.solani_Angelica                                            703 ---------------------------------------- 703
46.MAFF246664_F.cugenangense_Perilla                                       671 ---------------------------------------- 671
47.MH582420.1F.solanistrainMRC256                                          679 ---------------------------------------- 679
48.MAFF240361_F.babinda_Soil                                               693 ---------------------------------------- 693
49.MAFF242368_F.azukicola_Azukibean                                        708 ---------------------------------------- 708
50.MAFF241312_F.asiaticum_Soil,welshonionfield                             666 ---------------------------------------- 666
51.LT548416.1_F.culmorumpartialtef1a                                       646 ---------------------------------------- 646
52.MAFF150124_F.asiaticum__Wheat                                           666 ---------------------------------------- 666
53.MAFF238806_F.begoniae_Oncidiumsp                                        705 ---------------------------------------- 705
```

```
54.MW594399.1_FusariumincarnatumisolateUD01C                         689 ---------------------------------------- 689
55.OP414923.1Pucciniagraminisf.sp.triticiisolateSHZPgt19             623 ---------------------------------------- 623
56.MT027094.1_BipolarisoryzaestrainOrL-2                             895 ---------------------------------------- 895
57.ON734360.1_AlternariaalternataisolateH126                         831 ---------------------------------------- 831
58.LC333578.1_StemphyliumlycopersiciSOasp2                           265 ---------------------------------------- 265
59.HQ718583.1_Colletotrichumgloeosporioidesisolateq-1                567 ---------------------------------------- 567
60.JN241603.1_AtheliarolfsiiisolateSR1                              1013 ---------------------------------------- 1013
61.KJ866474.1_RhizoctoniasolanistrainMHL-1                           683 ---------------------------------------- 683
62.JQ672424.1AlternariatriticinaisolateEGS17-061                     825 ---------------------------------------- 825
63.LT707559.1_P.capsicipartialtef1agene                              964 ---------------------------------------- 964
64.MW090051.1_CurvularialunatastrainCls-3                            908 ---------------------------------------- 908
65.DQ400892.1_Aspergillusterreus                                     452 ---------------------------------------- 452
66.DQ911416.1_Pythiumsp.quercumstrainPy292                           932 ---------------------------------------- 932
67.EU797495.1_Phytophthorasp.oaksoilPoland                           930 ---------------------------------------- 930
68.HM148321.1_Cladosporiumcucumerinum                                385 ---------------------------------------- 385
69.AF398888.1_SclerotiniasclerotiorumisolateSS1                      334 ---------------------------------------- 334
70.AF398888.1_S.sclerotiorumisolateSS1                               334 ---------------------------------------- 334
71.HPAB545908.1_Verticilliumnonalfalfaeisolate                       442 ---------------------------------------- 442
72.EF433315.1_CeratocystisfimbriatavoucherCMW15052                   747 ---------------------------------------- 747
73.MN159912.1_Botrytiscinerea                                        931 ---------------------------------------- 931
74.MF034741.1_PeltasterfructicolaisolateSRB92                        317 ---------------------------------------- 317
75.LC440360.1_CercosporaasparagiCOasp2                               297 ---------------------------------------- 297
76.AY944105.1_MagnaportheoryzaeisolateSAG00T3()                      255 ---------------------------------------- 255
77.JX266586.1_CochliobolusmiyabeanusvoucherMFLUCC10-0733             918 ---------------------------------------- 918
78.MN393253.1_CorynesporacassiicolaisolateQHD001(MN393253.1UNVERIFIED) 305 ---------------------------------------- 305
79.MF375218.1_AgroatheliarolfsiiisolateBJB24                         544 ---------------------------------------- 544
80.MN106270.1_AgroatheliarolfsiistrainJ-12                           509 ---------------------------------------- 509
81.OQ732628.1_AgroatheliarolfsiiisolateBTCBSr3                       499 ---------------------------------------- 499
82.KY196185.1_ColletotrichumtruncatumstrainPAK53                     468 ---------------------------------------- 468
83.GU935835.1_ColletotrichumcoccodesisolateC96002                    1041 ---------------------------------------- 1041
84.MK085963.1_AlternariatenuissimaisolateSCCZ06                      252 ---------------------------------------- 252
85.MT548042.1_AlternarialongipesstrainKY_2019_012                    280 ---------------------------------------- 280
86.MN356465.1CalonectriamontanaisolateHSP4                           489 ---------------------------------------- 489
87.OL694224.1_CalonectriacanadianastrainF099                         504 ---------------------------------------- 504
88.MK803351.1_NeoscytalidiumdimidiatumstrainKale4-C                  299 ---------------------------------------- 299
89.ON376993.1_CurvulariachiangmaiensisisolateND00J7                  938 ---------------------------------------- 938
90.OQ383346.1_NeoscytalidiumdimidiatumisolateGKH-2                   297 ---------------------------------------- 297
91.MF662595.1_NeoscytalidiumnovaehollandiaeisolateNeNo1              284 ---------------------------------------- 284
92.EF560588.1Melampsoralini                                          656 ---------------------------------------- 656
93.LC590862.1_NeoscytalidiumdimidiatumPSU-HP01TEF1                   267 ---------------------------------------- 267
94.KX278106.1_BotryosphaeriaqingyuanensisstrainCERC2947              284 ---------------------------------------- 284
95.AJ578763.1_Blumeriagraminisf.sp.hordeicyp51                       1178 CTGCATCAAAACGTATTGAAAGAGGTTCTCCGTCTGCATG 1217
96.MF490858.1_CurvulariadactylocteniicolastrainCPC28810              930 ---------------------------------------- 930
97.KT287115.1_Bipolariscactivoraisolate3.8.6                         1006 ---------------------------------------- 1006
98.MT560940.1_CurvulariacactivorastrainHLGH0118                      963 ---------------------------------------- 963
99.OM714565.1_CurvulariaplantarumstrainM0134                         983 ---------------------------------------- 983
```

100.MN159911.1_BotrytiscinereaSICAUCC19-0003	931	--	931
102.GU294713.1LasiodiplodiatheobromaestrainUCD2430TX	407	--	407
103.KX868094.1_Mycosphaerellasp.isolateCRM20.1	475	--	475
104.LC599478.1Pseudocercosporapini-densifloraeMUCC534	310	--	310
105.N584698.1BipolarissetariaestrainKBS4-2	1031	--	1031
2.OM160859.1_F.buharicum	560	--	560
1.LC727524.1_F.buharicum_OKI-1_Okura	682	--	682
3.KX302919.1_F.sublunatum	521	--	521
4.LT996094.1_F.convolutans	516	--	516
5.OM160861.1_F.abutilonis	630	--	630
6.OM160874.1_F.guadeloupense	560	--	560
7.MH392475.1_F.graminearum	430	--	430
8.MH582420.1_F.solani	540	--	540
9.MAFF244605_F.oxysporum	544	--	544
10.MAFF237278_F.contaminatum_Hylocereus	711	--	711
11.MAFF237649_F.concentricum__Riceroot	678	--	678
12.MAFF237650_F.concentricum__Wheat	679	--	679
13.MAFF239869_F.mangiferae__Ryukyupine	657	--	657
14.MAFF240460_F.fujikuroi_Passionfruit	661	--	661
15.MAFF241317_F.graminearum_Wheat	666	--	666
16.MAFF242670_F.ipomoeae_Wheat	672	--	672
17.MAFF245129_F.concentricum_Fraxinus	659	--	659
18.MAFF245395_F.cugenangense_Rhubarb	671	--	671
19.MAFF246637_F.nirenbergiae_Strawberry	671	--	671
20.MAFF246672_F.nirenbergiae_ChinesePeony	671	--	671
21.MAFF246697_F.commune_Urallicoriceroot	673	--	673
22.MAFF246729_F.falciforme_Angelica	691	--	691
23.MAFF247220_F.duplospermum__Euwallaceasp	694	--	694
24.MAFF410760_F.odoratissimum_alpha	669	--	669
25.MAFF244605_FusariumoxysporumSchlechtendal_MAFF244605_Tomato	672	--	672
26.MAFF241326_F.asiaticum_Wheat	669	--	669
27.MAFF245014_F.asiaticum_Wildsoybean	666	--	666
28.MAFF150124_F.asiaticum__Wheat	666	--	666
29.OM135603.1F.algeriense	699	--	699
30.MAFF237465_F.penzigii_Aloe	704	--	704
31.MAFF103054_F.oxysporumSchlechtendalf.sp.cucumerinum_Cucumber	671	--	671
32.MAFF712246_F.oxysporumSchlechtendalf.sp.dianthi__Carnation	673	--	673
33.MAFF305558_F.oxysporumSchlechtendalf.sp.fragariae__Watermelon	671	--	671
34.MAFF744087_F.oxysporumSchlechtendalf.sp.lactucae__Lettuce	671	--	671
35.MAFF726924_F.oxysporumSchlechtendalf.sp.lagenariae_Whitefloweredgourd	671	--	671
36.MAFF744003_F.oxysporumSchlechtendalf.sp.lagenariae_Squash)	672	--	672
37.MAFF305122_F.oxysporumSchlechtendalf.sp.melonis__Melon	671	--	671
38.MAFF306714_F.oxysporumSchlechtendalf.sp.momordicae_Balsampear	671	--	671
39.MAFF238905_F.oxysporumSchlechtendalf.sp.radicis-lycopersici_Tomato	674	--	674
40.MAFF150004_F.oxysporumSchlechtendalf.sp.spinaciae_Spinach	671	--	671

41.MAFF247034_F.oxysporumSchlechtendal__Goldenchain	671	--	671
42.MAFF245747_F.oxysporumSchlechtendalf.sp.callistephi__Chinaaster	671	--	671
43.MAFF305115_FoxysporumSchlechtendalf.sp.batatas__Sweatpotato	703	--	703
44.MAFF150126_F.asiaticum_Seed	666	--	666
45.MAFF246738_F.solani_Angelica	703	--	703
46.MAFF246664_F.cugenangense_Perilla	671	--	671
47.MH582420.1F.solanistrainMRC256	679	--	679
48.MAFF240361_F.babinda_Soil	693	--	693
49.MAFF242368_F.azukicola_Azukibean	708	--	708
50.MAFF241312_F.asiaticum_Soil,welshonionfield	666	--	666
51.LT548416.1_F.culmorumpartialtef1a	646	--	646
52.MAFF150124_F.asiaticum__Wheat	666	--	666
53.MAFF238806_F.begoniae_Oncidiumsp	705	--	705
54.MW594399.1_FusariumincarnatumisolateUD01C	689	--	689
55.OP414923.1Pucciniagraminisf.sp.triticiisolateSHZPgt19	623	--	623
56.MT027094.1_BipolarisoryzaestrainOrL-2	895	--	895
57.ON734360.1_AlternariaalternataisolateH126	831	--	831
58.LC333578.1_StemphyliumlycopersiciSOasp2	265	--	265
59.HQ718583.1_Colletotrichumgloeosporioidesisolateq-1	567	--	567
60.JN241603.1_AtheliarolfsiiisolateSR1	1013	--	1013
61.KJ866474.1_RhizoctoniasolanistrainMHL-1	683	--	683
62.JQ672424.1AlternariatriticinaisolateEGS17-061	825	--	825
63.LT707559.1_P.capsicipartialtef1agene	964	--	964
64.MW090051.1_CurvularialunatastrainCls-3	908	--	908
65.DQ400892.1_Aspergillusterreus	452	--	452
66.DQ911416.1_Pythiumsp.quercumstrainPy292	932	--	932
67.EU797495.1_Phytophthorasp.oaksoilPoland	930	--	930
68.HM148321.1_Cladosporiumcucumerinum	385	--	385
69.AF398888.1_SclerotiniasclerotiorumisolateSS1	334	--	334
70.AF398888.1_S.sclerotiorumisolateSS1	334	--	334
71.HPAB545908.1_Verticilliumnonalfalfaeisolate	442	--	442
72.EF433315.1_CeratocystisfimbriatavoucherCMW15052	747	--	747
73.MN159912.1_Botrytiscinerea	931	--	931
74.MF034741.1_PeltasterfructicolaisolateSRB92	317	--	317
75.LC440360.1_CercosporaasparagiCOasp2	297	--	297
76.AY944105.1_MagnaportheoryzaeisolateSAG00T3()	255	--	255
77.JX266586.1_CochliobolusmiyabeanusvoucherMFLUCC10-0733	918	--	918
78.MN393253.1_CorynesporacassiicolaisolateQHD001(MN393253.1UNVERIFIED)	305	--	305
79.MF375218.1_AgroatheliarolfsiiisolateBJB24	544	--	544
80.MN106270.1_AgroatheliarolfsiistrainJ-12	509	--	509
81.OQ732628.1_AgroatheliarolfsiiisolateBTCBSr3	499	--	499
82.KY196185.1_ColletotrichumtruncatumstrainPAK53	468	--	468
83.GU935835.1_ColletotrichumcoccodesisolateC96002	1041	--	1041
84.MK085963.1_AlternariatenuissimaisolateSCCZ06	252	--	252
85.MT548042.1_AlternarialongipesstrainKY_2019_012	280	--	280
86.MN356465.1CalonectriamontanaisolateHSP4	489	--	489

87.OL694224.1_CalonectriacanadianastrainF099	504	--	504
88.MK803351.1_NeoscytalidiumdimidiatumstrainKale4-C	299	--	299
89.ON376993.1_CurvulariachiangmaiensisisolateND00J7	938	--	938
90.OQ383346.1_NeoscytalidiumdimidiatumisolateGKH-2	297	--	297
91.MF662595.1_NeoscytalidiumnovaehollandiaeisolateNeNo1	284	--	284
92.EF560588.1Melampsoralini	656	--	656
93.LC590862.1_NeoscytalidiumdimidiatumPSU-HP01TEF1	267	--	267
94.KX278106.1_BotryosphaeriaqingyuanensisstrainCERC2947	284	--	284
95.AJ578763.1_Blumeriagraminisf.sp.hordeicyp51	1218	CTCCCATACATTCGATCTTACGAAAAGTAAAGAATCCAAT	1257
96.MF490858.1_CurvulariadactylocteniicolastrainCPC28810	930	--	930
97.KT287115.1_Bipolariscactivoraisolate3.8.6	1006	--	1006
98.MT560940.1_CurvulariacactivorastrainHLGH0118	963	--	963
99.OM714565.1_CurvulariaplantarumstrainM0134	983	--	983
100.MN159911.1_BotrytiscinereaSICAUCC19-0003	931	--	931
102.GU294713.1LasiodiplodiatheobromaestrainUCD2430TX	407	--	407
103.KX868094.1_Mycosphaerellasp.isolateCRM20.1	475	--	475
104.LC599478.1Pseudocercosporapini-densifloraeMUCC534	310	--	310
105.N584698.1BipolarissetariaestrainKBS4-2	1031	--	1031

2.OM160859.1_F.buharicum	560	--	560
1.LC727524.1_F.buharicum_OKI-1_Okura	682	--	682
3.KX302919.1_F.sublunatum	521	--	521
4.LT996094.1_F.convolutans	516	--	516
5.OM160861.1_F.abutilonis	630	--	630
6.OM160874.1_F.guadeloupense	560	--	560
7.MH392475.1_F.graminearum	430	--	430
8.MH582420.1_F.solani	540	--	540
9.MAFF244605_F.oxysporum	544	--	544
10.MAFF237278_F.contaminatum_Hylocereus	711	--	711
11.MAFF237649_F.concentricum__Riceroot	678	--	678
12.MAFF237650_F.concentricum__Wheat	679	--	679
13.MAFF239869_F.mangiferae__Ryukyupine	657	--	657
14.MAFF240460_F.fujikuroi_Passionfruit	661	--	661
15.MAFF241317_F.graminearum_Wheat	666	--	666
16.MAFF242670_F.ipomoeae_Wheat	672	--	672
17.MAFF245129_F.concentricum_Fraxinus	659	--	659
18.MAFF245395_F.cugenangense_Rhubarb	671	--	671
19.MAFF246637_F.nirenbergiae_Strawberry	671	--	671
20.MAFF246672_F.nirenbergiae_ChinesePeony	671	--	671
21.MAFF246697_F.commune_Urallicoriceroot	673	--	673
22.MAFF246729_F.falciforme_Angelica	691	--	691
23.MAFF247220_F.duplospermum__Euwallaceasp	694	--	694
24.MAFF410760_F.odoratissimum_alpha	669	--	669
25.MAFF244605_FusariumoxysporumSchlechtendal_MAFF244605_Tomato	672	--	672
26.MAFF241326_F.asiaticum_Wheat	669	--	669
27.MAFF245014_F.asiaticum_Wildsoybean	666	--	666

28.MAFF150124_F.asiaticum__Wheat	666	--	666
29.OM135603.1F.algeriense	699	--	699
30.MAFF237465_F.penzigii_Aloe	704	--	704
31.MAFF103054_F.oxysporumSchlechtendalf.sp.cucumerinum_Cucumber	671	--	671
32.MAFF712246_F.oxysporumSchlechtendalf.sp.dianthi__Carnation	673	--	673
33.MAFF305558_F.oxysporumSchlechtendalf.sp.fragariae__Watermelon	671	--	671
34.MAFF744087_F.oxysporumSchlechtendalf.sp.lactucae__Lettuce	671	--	671
35.MAFF726924_F.oxysporumSchlechtendalf.sp.lagenariae_Whitefloweredgourd	671	--	671
36.MAFF744003_F.oxysporumSchlechtendalf.sp.lagenariae_Squash)	672	--	672
37.MAFF305122_F.oxysporumSchlechtendalf.sp.melonis__Melon	671	--	671
38.MAFF306714_F.oxysporumSchlechtendalf.sp.momordicae_Balsampear	671	--	671
39.MAFF238905_F.oxysporumSchlechtendalf.sp.radicis-lycopersici_Tomato	674	--	674
40.MAFF150004_F.oxysporumSchlechtendalf.sp.spinaciae_Spinach	671	--	671
41.MAFF247034_F.oxysporumSchlechtendal__Goldenchain	671	--	671
42.MAFF245747_F.oxysporumSchlechtendalf.sp.callistephi__Chinaaster	671	--	671
43.MAFF305115_FoxysporumSchlechtendalf.sp.batatas__Sweatpotato	703	--	703
44.MAFF150126_F.asiaticum_Seed	666	--	666
45.MAFF246738_F.solani_Angelica	703	--	703
46.MAFF246664_F.cugenangense_Perilla	671	--	671
47.MH582420.1F.solanistrainMRC256	679	--	679
48.MAFF240361_F.babinda_Soil	693	--	693
49.MAFF242368_F.azukicola_Azukibean	708	--	708
50.MAFF241312_F.asiaticum_Soil,welshonionfield	666	--	666
51.LT548416.1_F.culmorumpartialtef1a	646	--	646
52.MAFF150124_F.asiaticum__Wheat	666	--	666
53.MAFF238806_F.begoniae_Oncidiumsp	705	--	705
54.MW594399.1_FusariumincarnatumisolateUD01C	689	--	689
55.OP414923.1Pucciniagraminisf.sp.triticiisolateSHZPgt19	623	--	623
56.MT027094.1_BipolarisoryzaestrainOrL-2	895	--	895
57.ON734360.1_AlternariaalternataisolateH126	831	--	831
58.LC333578.1_StemphyliumlycopersiciSOasp2	265	--	265
59.HQ718583.1_Colletotrichumgloeosporioidesisolateq-1	567	--	567
60.JN241603.1_AtheliarolfsiiisolateSR1	1013	--	1013
61.KJ866474.1_RhizoctoniasolanistrainMHL-1	683	--	683
62.JQ672424.1AlternariatriticinaisolateEGS17-061	825	--	825
63.LT707559.1_P.capsicipartialtef1agene	964	--	964
64.MW090051.1_CurvularialunatastrainCls-3	908	--	908
65.DQ400892.1_Aspergillusterreus	452	--	452
66.DQ911416.1_Pythiumsp.quercumstrainPy292	932	--	932
67.EU797495.1_Phytophthorasp.oaksoilPoland	930	--	930
68.HM148321.1_Cladosporiumcucumerinum	385	--	385
69.AF398888.1_SclerotiniasclerotiorumisolateSS1	334	--	334
70.AF398888.1_S.sclerotiorumisolateSS1	334	--	334
71.HPAB545908.1_Verticilliumnonalfalfaeisolate	442	--	442
72.EF433315.1_CeratocystisfimbriatavoucherCMW15052	747	--	747
73.MN159912.1_Botrytiscinerea	931	--	931

```
74.MF034741.1_PeltasterfructicolaisolateSRB92                       317 ---------------------------------------- 317
75.LC440360.1_CercosporaasparagiCOasp2                              297 ---------------------------------------- 297
76.AY944105.1_MagnaportheoryzaeisolateSAG00T3()                     255 ---------------------------------------- 255
77.JX266586.1_CochliobolusmiyabeanusvoucherMFLUCC10-0733            918 ---------------------------------------- 918
78.MN393253.1_CorynesporacassiicolaisolateQHD001(MN393253.1UNVERIFIED) 305 ---------------------------------------- 305
79.MF375218.1_AgroatheliarolfsiiisolateBJB24                        544 ---------------------------------------- 544
80.MN106270.1_AgroatheliarolfsiistrainJ-12                          509 ---------------------------------------- 509
81.OQ732628.1_AgroatheliarolfsiiisolateBTCBSr3                      499 ---------------------------------------- 499
82.KY196185.1_ColletotrichumtruncatumstrainPAK53                    468 ---------------------------------------- 468
83.GU935835.1_ColletotrichumcoccodesisolateC96002                  1041 ---------------------------------------- 1041
84.MK085963.1_AlternariatenuissimaisolateSCCZ06                     252 ---------------------------------------- 252
85.MT548042.1_AlternarialongipesstrainKY_2019_012                   280 ---------------------------------------- 280
86.MN356465.1CalonectriamontanaisolateHSP4                          489 ---------------------------------------- 489
87.OL694224.1_CalonectriacanadianastrainF099                        504 ---------------------------------------- 504
88.MK803351.1_NeoscytalidiumdimidiatumstrainKale4-C                 299 ---------------------------------------- 299
89.ON376993.1_CurvulariachiangmaiensisisolateND00J7                 938 ---------------------------------------- 938
90.OQ383346.1_NeoscytalidiumdimidiatumisolateGKH-2                  297 ---------------------------------------- 297
91.MF662595.1_NeoscytalidiumnovaehollandiaeisolateNeNo1             284 ---------------------------------------- 284
92.EF560588.1Melampsoralini                                         656 ---------------------------------------- 656
93.LC590862.1_NeoscytalidiumdimidiatumPSU-HP01TEF1                  267 ---------------------------------------- 267
94.KX278106.1_BotryosphaeriaqingyuanensisstrainCERC2947             284 ---------------------------------------- 284
95.AJ578763.1_Blumeriagraminisf.sp.hordeicyp51                     1258 GCCCGTTCCAGGAACTAGTTATGTAATACCTAAGACCCAT 1297
96.MF490858.1_CurvulariadactylocteniicolastrainCPC28810             930 ---------------------------------------- 930
97.KT287115.1_Bipolariscactivoraisolate3.8.6                       1006 ---------------------------------------- 1006
98.MT560940.1_CurvulariacactivorastrainHLGH0118                     963 ---------------------------------------- 963
99.OM714565.1_CurvulariaplantarumstrainM0134                        983 ---------------------------------------- 983
100.MN159911.1_BotrytiscinereaSICAUCC19-0003                        931 ---------------------------------------- 931
102.GU294713.1LasiodiplodiatheobromaestrainUCD2430TX                407 ---------------------------------------- 407
103.KX868094.1_Mycosphaerellasp.isolateCRM20.1                      475 ---------------------------------------- 475
104.LC599478.1Pseudocercosporapini-densifloraeMUCC534               310 ---------------------------------------- 310
105.N584698.1BipolarissetariaestrainKBS4-2                         1031 ---------------------------------------- 1031

2.OM160859.1_F.buharicum                                            560 ---------------------------------------- 560
1.LC727524.1_F.buharicum_OKI-1_Okura                                682 ---------------------------------------- 682
3.KX302919.1_F.sublunatum                                           521 ---------------------------------------- 521
4.LT996094.1_F.convolutans                                          516 ---------------------------------------- 516
5.OM160861.1_F.abutilonis                                           630 ---------------------------------------- 630
6.OM160874.1_F.guadeloupense                                        560 ---------------------------------------- 560
7.MH392475.1_F.graminearum                                          430 ---------------------------------------- 430
8.MH582420.1_F.solani                                               540 ---------------------------------------- 540
9.MAFF244605_F.oxysporum                                            544 ---------------------------------------- 544
10.MAFF237278_F.contaminatum_Hylocereus                             711 ---------------------------------------- 711
11.MAFF237649_F.concentricum__Riceroot                              678 ---------------------------------------- 678
12.MAFF237650_F.concentricum__Wheat                                 679 ---------------------------------------- 679
13.MAFF239869_F.mangiferae__Ryukyupine                              657 ---------------------------------------- 657
14.MAFF240460_F.fujikuroi_Passionfruit                              661 ---------------------------------------- 661
```

```
15.MAFF241317_F.graminearum_Wheat                                  666 ---------------------------------------- 666
16.MAFF242670_F.ipomoeae_Wheat                                     672 ---------------------------------------- 672
17.MAFF245129_F.concentricum_Fraxinus                              659 ---------------------------------------- 659
18.MAFF245395_F.cugenangense_Rhubarb                               671 ---------------------------------------- 671
19.MAFF246637_F.nirenbergiae_Strawberry                            671 ---------------------------------------- 671
20.MAFF246672_F.nirenbergiae_ChinesePeony                          671 ---------------------------------------- 671
21.MAFF246697_F.commune_Urallicoriceroot                           673 ---------------------------------------- 673
22.MAFF246729_F.falciforme_Angelica                                691 ---------------------------------------- 691
23.MAFF247220_F.duplospermum__Euwallaceasp                         694 ---------------------------------------- 694
24.MAFF410760_F.odoratissimum_alpha                                669 ---------------------------------------- 669
25.MAFF244605_FusariumoxysporumSchlechtendal_MAFF244605_Tomato     672 ---------------------------------------- 672
26.MAFF241326_F.asiaticum_Wheat                                    669 ---------------------------------------- 669
27.MAFF245014_F.asiaticum_Wildsoybean                              666 ---------------------------------------- 666
28.MAFF150124_F.asiaticum__Wheat                                   666 ---------------------------------------- 666
29.OM135603.1F.algeriense                                          699 ---------------------------------------- 699
30.MAFF237465_F.penzigii_Aloe                                      704 ---------------------------------------- 704
31.MAFF103054_F.oxysporumSchlechtendalf.sp.cucumerinum_Cucumber    671 ---------------------------------------- 671
32.MAFF712246_F.oxysporumSchlechtendalf.sp.dianthi__Carnation      673 ---------------------------------------- 673
33.MAFF305558_F.oxysporumSchlechtendalf.sp.fragariae__Watermelon   671 ---------------------------------------- 671
34.MAFF744087_F.oxysporumSchlechtendalf.sp.lactucae__Lettuce       671 ---------------------------------------- 671
35.MAFF726924_F.oxysporumSchlechtendalf.sp.lagenariae_Whiteflower edgourd 671 ---------------------------------------- 671
36.MAFF744003_F.oxysporumSchlechtendalf.sp.lagenariae_Squash)      672 ---------------------------------------- 672
37.MAFF305122_F.oxysporumSchlechtendalf.sp.melonis__Melon          671 ---------------------------------------- 671
38.MAFF306714_F.oxysporumSchlechtendalf.sp.momordicae_Balsampear   671 ---------------------------------------- 671
39.MAFF238905_F.oxysporumSchlechtendalf.sp.radicis-lycopersici_Tomato 674 ---------------------------------------- 674
40.MAFF150004_F.oxysporumSchlechtendalf.sp.spinaciae_Spinach       671 ---------------------------------------- 671
41.MAFF247034_F.oxysporumSchlechtendal__Goldenchain                671 ---------------------------------------- 671
42.MAFF245747_F.oxysporumSchlechtendalf.sp.callistephi__Chinaaster 671 ---------------------------------------- 671
43.MAFF305115_FoxysporumSchlechtendalf.sp.batatas__Sweatpotato     703 ---------------------------------------- 703
44.MAFF150126_F.asiaticum_Seed                                     666 ---------------------------------------- 666
45.MAFF246738_F.solani_Angelica                                    703 ---------------------------------------- 703
46.MAFF246664_F.cugenangense_Perilla                               671 ---------------------------------------- 671
47.MH582420.1F.solanistrainMRC256                                  679 ---------------------------------------- 679
48.MAFF240361_F.babinda_Soil                                       693 ---------------------------------------- 693
49.MAFF242368_F.azukicola_Azukibean                                708 ---------------------------------------- 708
50.MAFF241312_F.asiaticum_Soil,welshonionfield                     666 ---------------------------------------- 666
51.LT548416.1_F.culmorumpartialtef1a                               646 ---------------------------------------- 646
52.MAFF150124_F.asiaticum__Wheat                                   666 ---------------------------------------- 666
53.MAFF238806_F.begoniae_Oncidiumsp                                705 ---------------------------------------- 705
54.MW594399.1_FusariumincarnatumisolateUD01C                       689 ---------------------------------------- 689
55.OP414923.1Pucciniagraminisf.sp.triticiisolateSHZPgt19           623 ---------------------------------------- 623
56.MT027094.1_BipolarisoryzaestrainOrL-2                           895 ---------------------------------------- 895
57.ON734360.1_AlternariaalternataisolateH126                       831 ---------------------------------------- 831
58.LC333578.1_StemphyliumlycopersiciSOasp2                         265 ---------------------------------------- 265
59.HQ718583.1_Colletotrichumgloeosporioidesisolateq-1              567 ---------------------------------------- 567
60.JN241603.1_AtheliarolfsiiisolateSR1                            1013 ---------------------------------------- 1013
```

```
61.KJ866474.1_RhizoctoniasolanistrainMHL-1  683 ---------------------------------------- 683
62.JQ672424.1AlternariatriticinaisolateEGS17-061  825 ---------------------------------------- 825
63.LT707559.1_P.capsicipartialtef1agene  964 ---------------------------------------- 964
64.MW090051.1_CurvularialunatastrainCls-3  908 ---------------------------------------- 908
65.DQ400892.1_Aspergillusterreus  452 ---------------------------------------- 452
66.DQ911416.1_Pythiumsp.quercumstrainPy292  932 ---------------------------------------- 932
67.EU797495.1_Phytophthorasp.oaksoilPoland  930 ---------------------------------------- 930
68.HM148321.1_Cladosporiumcucumerinum  385 ---------------------------------------- 385
69.AF398888.1_SclerotiniasclerotiorumisolateSS1  334 ---------------------------------------- 334
70.AF398888.1_S.sclerotiorumisolateSS1  334 ---------------------------------------- 334
71.HPAB545908.1_Verticilliumnonalfalfaeisolate  442 ---------------------------------------- 442
72.EF433315.1_CeratocystisfimbriatavoucherCMW15052  747 ---------------------------------------- 747
73.MN159912.1_Botrytiscinerea  931 ---------------------------------------- 931
74.MF034741.1_PeltasterfructicolaisolateSRB92  317 ---------------------------------------- 317
75.LC440360.1_CercosporaasparagiCOasp2  297 ---------------------------------------- 297
76.AY944105.1_MagnaportheoryzaeisolateSAG00T3()  255 ---------------------------------------- 255
77.JX266586.1_CochliobolusmiyabeanusvoucherMFLUCC10-0733  918 ---------------------------------------- 918
78.MN393253.1_CorynesporacassiicolaisolateQHD001(MN393253.1UNVERIFIED)  305 ---------------------------------------- 305
79.MF375218.1_AgroatheliarolfsiiisolateBJB24  544 ---------------------------------------- 544
80.MN106270.1_AgroatheliarolfsiistrainJ-12  509 ---------------------------------------- 509
81.OQ732628.1_AgroatheliarolfsiiisolateBTCBSr3  499 ---------------------------------------- 499
82.KY196185.1_ColletotrichumtruncatumstrainPAK53  468 ---------------------------------------- 468
83.GU935835.1_ColletotrichumcoccodesisolateC96002  1041 ---------------------------------------- 1041
84.MK085963.1_AlternariatenuissimaisolateSCCZ06  252 ---------------------------------------- 252
85.MT548042.1_AlternarialongipesstrainKY_2019_012  280 ---------------------------------------- 280
86.MN356465.1CalonectriamontanaisolateHSP4  489 ---------------------------------------- 489
87.OL694224.1_CalonectriacanadianastrainF099  504 ---------------------------------------- 504
88.MK803351.1_NeoscytalidiumdimidiatumstrainKale4-C  299 ---------------------------------------- 299
89.ON376993.1_CurvulariachiangmaiensisisolateND00J7  938 ---------------------------------------- 938
90.OQ383346.1_NeoscytalidiumdimidiatumisolateGKH-2  297 ---------------------------------------- 297
91.MF662595.1_NeoscytalidiumnovaehollandiaeisolateNeNo1  284 ---------------------------------------- 284
92.EF560588.1Melampsoralini  656 ---------------------------------------- 656
93.LC590862.1_NeoscytalidiumdimidiatumPSU-HP01TEF1  267 ---------------------------------------- 267
94.KX278106.1_BotryosphaeriaqingyuanensisstrainCERC2947  284 ---------------------------------------- 284
95.AJ578763.1_Blumeriagraminisf.sp.hordeicyp51  1298 TCCCTCTTGGCGGCCCCTGGGTGGACGAGTCGAGACGCCT 1337
96.MF490858.1_CurvulariadactylocteniicolastrainCPC28810  930 ---------------------------------------- 930
97.KT287115.1_Bipolariscactivoraisolate3.8.6  1006 ---------------------------------------- 1006
98.MT560940.1_CurvulariacactivorastrainHLGH0118  963 ---------------------------------------- 963
99.OM714565.1_CurvulariaplantarumstrainM0134  983 ---------------------------------------- 983
100.MN159911.1_BotrytiscinereaSICAUCC19-0003  931 ---------------------------------------- 931
102.GU294713.1LasiodiplodiatheobromaestrainUCD2430TX  407 ---------------------------------------- 407
103.KX868094.1_Mycosphaerellasp.isolateCRM20.1  475 ---------------------------------------- 475
104.LC599478.1Pseudocercosporapini-densifloraeMUCC534  310 ---------------------------------------- 310
105.N584698.1BipolarissetariaestrainKBS4-2  1031 ---------------------------------------- 1031

2.OM160859.1_F.buharicum  560 ---------------------------------------- 560
```

```
1.LC727524.1_F.buharicum_OKI-1_Okura                                        682 ---------------------------------------- 682
3.KX302919.1_F.sublunatum                                                   521 ---------------------------------------- 521
4.LT996094.1_F.convolutans                                                  516 ---------------------------------------- 516
5.OM160861.1_F.abutilonis                                                   630 ---------------------------------------- 630
6.OM160874.1_F.guadeloupense                                                560 ---------------------------------------- 560
7.MH392475.1_F.graminearum                                                  430 ---------------------------------------- 430
8.MH582420.1_F.solani                                                       540 ---------------------------------------- 540
9.MAFF244605_F.oxysporum                                                    544 ---------------------------------------- 544
10.MAFF237278_F.contaminatum_Hylocereus                                     711 ---------------------------------------- 711
11.MAFF237649_F.concentricum__Riceroot                                      678 ---------------------------------------- 678
12.MAFF237650_F.concentricum__Wheat                                         679 ---------------------------------------- 679
13.MAFF239869_F.mangiferae__Ryukyupine                                      657 ---------------------------------------- 657
14.MAFF240460_F.fujikuroi_Passionfruit                                      661 ---------------------------------------- 661
15.MAFF241317_F.graminearum_Wheat                                           666 ---------------------------------------- 666
16.MAFF242670_F.ipomoeae_Wheat                                              672 ---------------------------------------- 672
17.MAFF245129_F.concentricum_Fraxinus                                       659 ---------------------------------------- 659
18.MAFF245395_F.cugenangense_Rhubarb                                        671 ---------------------------------------- 671
19.MAFF246637_F.nirenbergiae_Strawberry                                     671 ---------------------------------------- 671
20.MAFF246672_F.nirenbergiae_ChinesePeony                                   671 ---------------------------------------- 671
21.MAFF246697_F.commune_Urallicoriceroot                                    673 ---------------------------------------- 673
22.MAFF246729_F.falciforme_Angelica                                         691 ---------------------------------------- 691
23.MAFF247220_F.duplospermum__Euwallaceasp                                  694 ---------------------------------------- 694
24.MAFF410760_F.odoratissimum_alpha                                         669 ---------------------------------------- 669
25.MAFF244605_FusariumoxysporumSchlechtendal_MAFF244605_Tomato              672 ---------------------------------------- 672
26.MAFF241326_F.asiaticum_Wheat                                             669 ---------------------------------------- 669
27.MAFF245014_F.asiaticum_Wildsoybean                                       666 ---------------------------------------- 666
28.MAFF150124_F.asiaticum__Wheat                                            666 ---------------------------------------- 666
29.OM135603.1F.algeriense                                                   699 ---------------------------------------- 699
30.MAFF237465_F.penzigii_Aloe                                               704 ---------------------------------------- 704
31.MAFF103054_F.oxysporumSchlechtendalf.sp.cucumerinum_Cucumber             671 ---------------------------------------- 671
32.MAFF712246_F.oxysporumSchlechtendalf.sp.dianthi__Carnation               673 ---------------------------------------- 673
33.MAFF305558_F.oxysporumSchlechtendalf.sp.fragariae__Watermelon            671 ---------------------------------------- 671
34.MAFF744087_F.oxysporumSchlechtendalf.sp.lactucae__Lettuce                671 ---------------------------------------- 671
35.MAFF726924_F.oxysporumSchlechtendalf.sp.lagenariae_Whiteflowseredgourd   671 ---------------------------------------- 671
36.MAFF744003_F.oxysporumSchlechtendalf.sp.lagenariae_Squash)               672 ---------------------------------------- 672
37.MAFF305122_F.oxysporumSchlechtendalf.sp.melonis__Melon                   671 ---------------------------------------- 671
38.MAFF306714_F.oxysporumSchlechtendalf.sp.momordicae_Balsampear            671 ---------------------------------------- 671
39.MAFF238905_F.oxysporumSchlechtendalf.sp.radicis-lycopersici_Tomato       674 ---------------------------------------- 674
40.MAFF150004_F.oxysporumSchlechtendalf.sp.spinaciae_Spinach                671 ---------------------------------------- 671
41.MAFF247034_F.oxysporumSchlechtendal__Goldenchain                         671 ---------------------------------------- 671
42.MAFF245747_F.oxysporumSchlechtendalf.sp.callistephi__Chinaaster          671 ---------------------------------------- 671
43.MAFF305115_FoxysporumSchlechtendalf.sp.batatas__Sweatpotato              703 ---------------------------------------- 703
44.MAFF150126_F.asiaticum_Seed                                              666 ---------------------------------------- 666
45.MAFF246738_F.solani_Angelica                                             703 ---------------------------------------- 703
46.MAFF246664_F.cugenangense_Perilla                                        671 ---------------------------------------- 671
47.MH582420.1F.solanistrainMRC256                                           679 ---------------------------------------- 679
```

48.MAFF240361_F.babinda_Soil	693	--	693
49.MAFF242368_F.azukicola_Azukibean	708	--	708
50.MAFF241312_F.asiaticum_Soil,welshonionfield	666	--	666
51.LT548416.1_F.culmorumpartialtef1a	646	--	646
52.MAFF150124_F.asiaticum__Wheat	666	--	666
53.MAFF238806_F.begoniae_Oncidiumsp	705	--	705
54.MW594399.1_FusariumincarnatumisolateUD01C	689	--	689
55.OP414923.1Pucciniagraminisf.sp.triticiisolateSHZPgt19	623	--	623
56.MT027094.1_BipolarisoryzaestrainOrL-2	895	--	895
57.ON734360.1_AlternariaalternataisolateH126	831	--	831
58.LC333578.1_StemphyliumlycopersiciSOasp2	265	--	265
59.HQ718583.1_Colletotrichumgloeosporioidesisolateq-1	567	--	567
60.JN241603.1_AtheliarolfsiiisolateSR1	1013	--	1013
61.KJ866474.1_RhizoctoniasolanistrainMHL-1	683	--	683
62.JQ672424.1AlternariatriticinaisolateEGS17-061	825	--	825
63.LT707559.1_P.capsicipartialtef1agene	964	--	964
64.MW090051.1_CurvularialunatastrainCls-3	908	--	908
65.DQ400892.1_Aspergillusterreus	452	--	452
66.DQ911416.1_Pythiumsp.quercumstrainPy292	932	--	932
67.EU797495.1_Phytophthorasp.oaksoilPoland	930	--	930
68.HM148321.1_Cladosporiumcucumerinum	385	--	385
69.AF398888.1_SclerotiniasclerotiorumisolateSS1	334	--	334
70.AF398888.1_S.sclerotiorumisolateSS1	334	--	334
71.HPAB545908.1_Verticilliumnonalfalfaeisolate	442	--	442
72.EF433315.1_CeratocystisfimbriatavoucherCMW15052	747	--	747
73.MN159912.1_Botrytiscinerea	931	--	931
74.MF034741.1_PeltasterfructicolaisolateSRB92	317	--	317
75.LC440360.1_CercosporaasparagiCOasp2	297	--	297
76.AY944105.1_MagnaportheoryzaeisolateSAG00T3()	255	--	255
77.JX266586.1_CochliobolusmiyabeanusvoucherMFLUCC10-0733	918	--	918
78.MN393253.1_CorynesporacassiicolaisolateQHD001(MN393253.1UNVERIFIED)	305	--	305
79.MF375218.1_AgroatheliarolfsiiisolateBJB24	544	--	544
80.MN106270.1_AgroatheliarolfsiistrainJ-12	509	--	509
81.OQ732628.1_AgroatheliarolfsiiisolateBTCBSr3	499	--	499
82.KY196185.1_ColletotrichumtruncatumstrainPAK53	468	--	468
83.GU935835.1_ColletotrichumcoccodesisolateC96002	1041	--	1041
84.MK085963.1_AlternariatenuissimaisolateSCCZ06	252	--	252
85.MT548042.1_AlternarialongipesstrainKY_2019_012	280	--	280
86.MN356465.1CalonectriamontanaisolateHSP4	489	--	489
87.OL694224.1_CalonectriacanadianastrainF099	504	--	504
88.MK803351.1_NeoscytalidiumdimidiatumstrainKale4-C	299	--	299
89.ON376993.1_CurvulariachiangmaiensisisolateND00J7	938	--	938
90.OQ383346.1_NeoscytalidiumdimidiatumisolateGKH-2	297	--	297
91.MF662595.1_NeoscytalidiumnovaehollandiaeisolateNeNo1	284	--	284
92.EF560588.1Melampsoralini	656	--	656
93.LC590862.1_NeoscytalidiumdimidiatumPSU-HP01TEF1	267	--	267

94.KX278106.1_BotryosphaeriaqingyuanensisstrainCERC2947	284	--	284
95.AJ578763.1_Blumeriagraminisf.sp.hordeicyp51	1338	CATACTTCCCCAATCCGCTTAAGTGGGATCCACATCGTTG	1377
96.MF490858.1_CurvulariadactylocteniicolastrainCPC28810	930	--	930
97.KT287115.1_Bipolariscactivoraisolate3.8.6	1006	--	1006
98.MT560940.1_CurvulariacactivorastrainHLGH0118	963	--	963
99.OM714565.1_CurvulariaplantarumstrainM0134	983	--	983
100.MN159911.1_BotrytiscinereaSICAUCC19-0003	931	--	931
102.GU294713.1LasiodiplodiatheobromaestrainUCD2430TX	407	--	407
103.KX868094.1_Mycosphaerellasp.isolateCRM20.1	475	--	475
104.LC599478.1Pseudocercosporapini-densifloraeMUCC534	310	--	310
105.N584698.1BipolarissetariaestrainKBS4-2	1031	--	1031

2.OM160859.1_F.buharicum	560	--	560
1.LC727524.1_F.buharicum_OKI-1_Okura	682	--	682
3.KX302919.1_F.sublunatum	521	--	521
4.LT996094.1_F.convolutans	516	--	516
5.OM160861.1_F.abutilonis	630	--	630
6.OM160874.1_F.guadeloupense	560	--	560
7.MH392475.1_F.graminearum	430	--	430
8.MH582420.1_F.solani	540	--	540
9.MAFF244605_F.oxysporum	544	--	544
10.MAFF237278_F.contaminatum_Hylocereus	711	--	711
11.MAFF237649_F.concentricum__Riceroot	678	--	678
12.MAFF237650_F.concentricum__Wheat	679	--	679
13.MAFF239869_F.mangiferae__Ryukyupine	657	--	657
14.MAFF240460_F.fujikuroi_Passionfruit	661	--	661
15.MAFF241317_F.graminearum_Wheat	666	--	666
16.MAFF242670_F.ipomoeae_Wheat	672	--	672
17.MAFF245129_F.concentricum_Fraxinus	659	--	659
18.MAFF245395_F.cugenangense_Rhubarb	671	--	671
19.MAFF246637_F.nirenbergiae_Strawberry	671	--	671
20.MAFF246672_F.nirenbergiae_ChinesePeony	671	--	671
21.MAFF246697_F.commune_Urallicoriceroot	673	--	673
22.MAFF246729_F.falciforme_Angelica	691	--	691
23.MAFF247220_F.duplospermum__Euwallaceasp	694	--	694
24.MAFF410760_F.odoratissimum_alpha	669	--	669
25.MAFF244605_FusariumoxysporumSchlechtendal_MAFF244605_Tomato	672	--	672
26.MAFF241326_F.asiaticum_Wheat	669	--	669
27.MAFF245014_F.asiaticum_Wildsoybean	666	--	666
28.MAFF150124_F.asiaticum__Wheat	666	--	666
29.OM135603.1F.algeriense	699	--	699
30.MAFF237465_F.penzigii_Aloe	704	--	704
31.MAFF103054_F.oxysporumSchlechtendalf.sp.cucumerinum_Cucumber	671	--	671
32.MAFF712246_F.oxysporumSchlechtendalf.sp.dianthi__Carnation	673	--	673
33.MAFF305558_F.oxysporumSchlechtendalf.sp.fragariae__Watermelon	671	--	671
34.MAFF744087_F.oxysporumSchlechtendalf.sp.lactucae__Lettuce	671	--	671

```
35.MAFF726924_F.oxysporumSchlechtendalf.sp.lagenariae_Whiteflowered gourd  671 ---------------------------------------- 671
36.MAFF744003_F.oxysporumSchlechtendalf.sp.lagenariae_Squash)             672 ---------------------------------------- 672
37.MAFF305122_F.oxysporumSchlechtendalf.sp.melonis__Melon                 671 ---------------------------------------- 671
38.MAFF306714_F.oxysporumSchlechtendalf.sp.momordicae_Balsampear          671 ---------------------------------------- 671
39.MAFF238905_F.oxysporumSchlechtendalf.sp.radicis-lycopersici_Tomato     674 ---------------------------------------- 674
40.MAFF150004_F.oxysporumSchlechtendalf.sp.spinaciae_Spinach              671 ---------------------------------------- 671
41.MAFF247034_F.oxysporumSchlechtendal__Goldenchain                       671 ---------------------------------------- 671
42.MAFF245747_F.oxysporumSchlechtendalf.sp.callistephi__Chinaaster        671 ---------------------------------------- 671
43.MAFF305115_FoxysporumSchlechtendalf.sp.batatas__Sweatpotato            703 ---------------------------------------- 703
44.MAFF150126_F.asiaticum_Seed                                            666 ---------------------------------------- 666
45.MAFF246738_F.solani_Angelica                                           703 ---------------------------------------- 703
46.MAFF246664_F.cugenangense_Perilla                                      671 ---------------------------------------- 671
47.MH582420.1F.solanistrainMRC256                                         679 ---------------------------------------- 679
48.MAFF240361_F.babinda_Soil                                              693 ---------------------------------------- 693
49.MAFF242368_F.azukicola_Azukibean                                       708 ---------------------------------------- 708
50.MAFF241312_F.asiaticum_Soil,welshonionfield                            666 ---------------------------------------- 666
51.LT548416.1_F.culmorumpartialtef1a                                      646 ---------------------------------------- 646
52.MAFF150124_F.asiaticum__Wheat                                          666 ---------------------------------------- 666
53.MAFF238806_F.begoniae_Oncidiumsp                                       705 ---------------------------------------- 705
54.MW594399.1_FusariumincarnatumisolateUD01C                              689 ---------------------------------------- 689
55.OP414923.1Pucciniagraminisf.sp.triticiisolateSHZPgt19                  623 ---------------------------------------- 623
56.MT027094.1_BipolarisoryzaestrainOrL-2                                  895 ---------------------------------------- 895
57.ON734360.1_AlternariaalternataisolateH126                              831 ---------------------------------------- 831
58.LC333578.1_StemphyliumlycopersiciSOasp2                                265 ---------------------------------------- 265
59.HQ718583.1_Colletotrichumgloeosporioidesisolateq-1                     567 ---------------------------------------- 567
60.JN241603.1_AtheliarolfsiiisolateSR1                                   1013 ---------------------------------------- 1013
61.KJ866474.1_RhizoctoniasolanistrainMHL-1                                683 ---------------------------------------- 683
62.JQ672424.1AlternariatriticinaisolateEGS17-061                          825 ---------------------------------------- 825
63.LT707559.1_P.capsicipartialtef1agene                                   964 ---------------------------------------- 964
64.MW090051.1_CurvularialunatastrainCls-3                                 908 ---------------------------------------- 908
65.DQ400892.1_Aspergillusterreus                                          452 ---------------------------------------- 452
66.DQ911416.1_Pythiumsp.quercumstrainPy292                                932 ---------------------------------------- 932
67.EU797495.1_Phytophthorasp.oaksoilPoland                                930 ---------------------------------------- 930
68.HM148321.1_Cladosporiumcucumerinum                                     385 ---------------------------------------- 385
69.AF398888.1_SclerotiniasclerotiorumisolateSS1                           334 ---------------------------------------- 334
70.AF398888.1_S.sclerotiorumisolateSS1                                    334 ---------------------------------------- 334
71.HPAB545908.1_Verticilliumnonalfalfaeisolate                            442 ---------------------------------------- 442
72.EF433315.1_CeratocystisfimbriatavoucherCMW15052                        747 ---------------------------------------- 747
73.MN159912.1_Botrytiscinerea                                             931 ---------------------------------------- 931
74.MF034741.1_PeltasterfructicolaisolateSRB92                             317 ---------------------------------------- 317
75.LC440360.1_CercosporaasparagiCOasp2                                    297 ---------------------------------------- 297
76.AY944105.1_MagnaportheoryzaeisolateSAG00T3()                           255 ---------------------------------------- 255
77.JX266586.1_CochliobolusmiyabeanusvoucherMFLUCC10-0733                  918 ---------------------------------------- 918
78.MN393253.1_CorynesporacassiicolaisolateQHD001(MN393253.1UNVERIFIED)    305 ---------------------------------------- 305
79.MF375218.1_AgroatheliarolfsiiisolateBJB24                              544 ---------------------------------------- 544
80.MN106270.1_AgroatheliarolfsiistrainJ-12                                509 ---------------------------------------- 509
```

```
81.OQ732628.1_AgroatheliarolfsiiisolateBTCBSr3                 499 ---------------------------------------- 499
82.KY196185.1_ColletotrichumtruncatumstrainPAK53               468 ---------------------------------------- 468
83.GU935835.1_ColletotrichumcoccodesisolateC96002             1041 ---------------------------------------- 1041
84.MK085963.1_AlternariatenuissimaisolateSCCZ06                252 ---------------------------------------- 252
85.MT548042.1_AlternarialongipesstrainKY_2019_012              280 ---------------------------------------- 280
86.MN356465.1CalonectriamontanaisolateHSP4                     489 ---------------------------------------- 489
87.OL694224.1_CalonectriacanadianastrainF099                   504 ---------------------------------------- 504
88.MK803351.1_NeoscytalidiumdimidiatumstrainKale4-C            299 ---------------------------------------- 299
89.ON376993.1_CurvulariachiangmaiensisisolateND00J7            938 ---------------------------------------- 938
90.OQ383346.1_NeoscytalidiumdimidiatumisolateGKH-2             297 ---------------------------------------- 297
91.MF662595.1_NeoscytalidiumnovaehollandiaeisolateNeNo1        284 ---------------------------------------- 284
92.EF560588.1Melampsoralini                                    656 ---------------------------------------- 656
93.LC590862.1_NeoscytalidiumdimidiatumPSU-HP01TEF1             267 ---------------------------------------- 267
94.KX278106.1_BotryosphaeriaqingyuanensisstrainCERC2947        284 ---------------------------------------- 284
95.AJ578763.1_Blumeriagraminisf.sp.hordeicyp51                1378 GGACACTGGATCTGGTGGCGTGATAGGCACGGATATGGAG 1417
96.MF490858.1_CurvulariadactylocteniicolastrainCPC28810        930 ---------------------------------------- 930
97.KT287115.1_Bipolariscactivoraisolate3.8.6                  1006 ---------------------------------------- 1006
98.MT560940.1_CurvulariacactivorastrainHLGH0118                963 ---------------------------------------- 963
99.OM714565.1_CurvulariaplantarumstrainM0134                   983 ---------------------------------------- 983
100.MN159911.1_BotrytiscinereaSICAUCC19-0003                   931 ---------------------------------------- 931
102.GU294713.1LasiodiplodiatheobromaestrainUCD2430TX           407 ---------------------------------------- 407
103.KX868094.1_Mycosphaerellasp.isolateCRM20.1                 475 ---------------------------------------- 475
104.LC599478.1Pseudocercosporapini-densifloraeMUCC534          310 ---------------------------------------- 310
105.N584698.1BipolarissetariaestrainKBS4-2                    1031 ---------------------------------------- 1031

2.OM160859.1_F.buharicum                                       560 ---------------------------------------- 560
1.LC727524.1_F.buharicum_OKI-1_Okura                           682 ---------------------------------------- 682
3.KX302919.1_F.sublunatum                                      521 ---------------------------------------- 521
4.LT996094.1_F.convolutans                                     516 ---------------------------------------- 516
5.OM160861.1_F.abutilonis                                      630 ---------------------------------------- 630
6.OM160874.1_F.guadeloupense                                   560 ---------------------------------------- 560
7.MH392475.1_F.graminearum                                     430 ---------------------------------------- 430
8.MH582420.1_F.solani                                          540 ---------------------------------------- 540
9.MAFF244605_F.oxysporum                                       544 ---------------------------------------- 544
10.MAFF237278_F.contaminatum_Hylocereus                        711 ---------------------------------------- 711
11.MAFF237649_F.concentricum__Riceroot                         678 ---------------------------------------- 678
12.MAFF237650_F.concentricum__Wheat                            679 ---------------------------------------- 679
13.MAFF239869_F.mangiferae__Ryukyupine                         657 ---------------------------------------- 657
14.MAFF240460_F.fujikuroi_Passionfruit                         661 ---------------------------------------- 661
15.MAFF241317_F.graminearum_Wheat                              666 ---------------------------------------- 666
16.MAFF242670_F.ipomoeae_Wheat                                 672 ---------------------------------------- 672
17.MAFF245129_F.concentricum_Fraxinus                          659 ---------------------------------------- 659
18.MAFF245395_F.cugenangense_Rhubarb                           671 ---------------------------------------- 671
19.MAFF246637_F.nirenbergiae_Strawberry                        671 ---------------------------------------- 671
20.MAFF246672_F.nirenbergiae_ChinesePeony                      671 ---------------------------------------- 671
21.MAFF246697_F.commune_Urallicoriceroot                       673 ---------------------------------------- 673
```

22.MAFF246729_F.falciforme_Angelica 691 -- 691
23.MAFF247220_F.duplospermum__Euwallaceasp 694 -- 694
24.MAFF410760_F.odoratissimum_alpha 669 -- 669
25.MAFF244605_FusariumoxysporumSchlechtendal_MAFF244605_Tomato 672 -- 672
26.MAFF241326_F.asiaticum_Wheat 669 -- 669
27.MAFF245014_F.asiaticum_Wildsoybean 666 -- 666
28.MAFF150124_F.asiaticum__Wheat 666 -- 666
29.OM135603.1F.algeriense 699 -- 699
30.MAFF237465_F.penzigii_Aloe 704 -- 704
31.MAFF103054_F.oxysporumSchlechtendalf.sp.cucumerinum_Cucumber 671 -- 671
32.MAFF712246_F.oxysporumSchlechtendalf.sp.dianthi__Carnation 673 -- 673
33.MAFF305558_F.oxysporumSchlechtendalf.sp.fragariae__Watermelon 671 -- 671
34.MAFF744087_F.oxysporumSchlechtendalf.sp.lactucae__Lettuce 671 -- 671
35.MAFF726924_F.oxysporumSchlechtendalf.sp.lagenariae_Whitefloweredgourd 671 -- 671
36.MAFF744003_F.oxysporumSchlechtendalf.sp.lagenariae_Squash) 672 -- 672
37.MAFF305122_F.oxysporumSchlechtendalf.sp.melonis__Melon 671 -- 671
38.MAFF306714_F.oxysporumSchlechtendalf.sp.momordicae_Balsampear 671 -- 671
39.MAFF238905_F.oxysporumSchlechtendalf.sp.radicis-lycopersici_Tomato 674 -- 674
40.MAFF150004_F.oxysporumSchlechtendalf.sp.spinaciae_Spinach 671 -- 671
41.MAFF247034_F.oxysporumSchlechtendal__Goldenchain 671 -- 671
42.MAFF245747_F.oxysporumSchlechtendalf.sp.callistephi__Chinaaster 671 -- 671
43.MAFF305115_FoxysporumSchlechtendalf.sp.batatas__Sweatpotato 703 -- 703
44.MAFF150126_F.asiaticum_Seed 666 -- 666
45.MAFF246738_F.solani_Angelica 703 -- 703
46.MAFF246664_F.cugenangense_Perilla 671 -- 671
47.MH582420.1F.solanistrainMRC256 679 -- 679
48.MAFF240361_F.babinda_Soil 693 -- 693
49.MAFF242368_F.azukicola_Azukibean 708 -- 708
50.MAFF241312_F.asiaticum_Soil,welshonionfield 666 -- 666
51.LT548416.1_F.culmorumpartialtef1a 646 -- 646
52.MAFF150124_F.asiaticum__Wheat 666 -- 666
53.MAFF238806_F.begoniae_Oncidiumsp 705 -- 705
54.MW594399.1_FusariumincarnatumisolateUD01C 689 -- 689
55.OP414923.1Pucciniagraminisf.sp.triticiisolateSHZPgt19 623 -- 623
56.MT027094.1_BipolarisoryzaestrainOrL-2 895 -- 895
57.ON734360.1_AlternariaalternataisolateH126 831 -- 831
58.LC333578.1_StemphyliumlycopersiciSOasp2 265 -- 265
59.HQ718583.1_Colletotrichumgloeosporioidesisolateq-1 567 -- 567
60.JN241603.1_AtheliarolfsiiisolateSR1 1013 -- 1013
61.KJ866474.1_RhizoctoniasolanistrainMHL-1 683 -- 683
62.JQ672424.1AlternariatriticinaisolateEGS17-061 825 -- 825
63.LT707559.1_P.capsicipartialtef1agene 964 -- 964
64.MW090051.1_CurvularialunatastrainCls-3 908 -- 908
65.DQ400892.1_Aspergillusterreus 452 -- 452
66.DQ911416.1_Pythiumsp.quercumstrainPy292 932 -- 932
67.EU797495.1_Phytophthorasp.oaksoilPoland 930 -- 930

68.HM148321.1_Cladosporiumcucumerinum	385	--	385
69.AF398888.1_SclerotiniasclerotiorumisolateSS1	334	--	334
70.AF398888.1_S.sclerotiorumisolateSS1	334	--	334
71.HPAB545908.1_Verticilliumnonalfalfaeisolate	442	--	442
72.EF433315.1_CeratocystisfimbriatavoucherCMW15052	747	--	747
73.MN159912.1_Botrytiscinerea	931	--	931
74.MF034741.1_PeltasterfructicolaisolateSRB92	317	--	317
75.LC440360.1_CercosporaasparagiCOasp2	297	--	297
76.AY944105.1_MagnaportheoryzaeisolateSAG00T3()	255	--	255
77.JX266586.1_CochliobolusmiyabeanusvoucherMFLUCC10-0733	918	--	918
78.MN393253.1_CorynesporacassiicolaisolateQHD001(MN393253.1UNVERIFIED)	305	--	305
79.MF375218.1_AgroatheliarolfsiiisolateBJB24	544	--	544
80.MN106270.1_AgroatheliarolfsiistrainJ-12	509	--	509
81.OQ732628.1_AgroatheliarolfsiiisolateBTCBSr3	499	--	499
82.KY196185.1_ColletotrichumtruncatumstrainPAK53	468	--	468
83.GU935835.1_ColletotrichumcoccodesisolateC96002	1041	--	1041
84.MK085963.1_AlternariatenuissimaisolateSCCZ06	252	--	252
85.MT548042.1_AlternarialongipesstrainKY_2019_012	280	--	280
86.MN356465.1CalonectriamontanaisolateHSP4	489	--	489
87.OL694224.1_CalonectriacanadianastrainF099	504	--	504
88.MK803351.1_NeoscytalidiumdimidiatumstrainKale4-C	299	--	299
89.ON376993.1_CurvulariachiangmaiensisisolateND00J7	938	--	938
90.OQ383346.1_NeoscytalidiumdimidiatumisolateGKH-2	297	--	297
91.MF662595.1_NeoscytalidiumnovaehollandiaeisolateNeNo1	284	--	284
92.EF560588.1Melampsoralini	656	--	656
93.LC590862.1_NeoscytalidiumdimidiatumPSU-HP01TEF1	267	--	267
94.KX278106.1_BotryosphaeriaqingyuanensisstrainCERC2947	284	--	284
95.AJ578763.1_Blumeriagraminisf.sp.hordeicyp51	1418	GATGAAAAATTCGATTATGGGTATGGATTAATTAGTACAG	1457
96.MF490858.1_CurvulariadactylocteniicolastrainCPC28810	930	--	930
97.KT287115.1_Bipolariscactivoraisolate3.8.6	1006	--	1006
98.MT560940.1_CurvulariacactivorastrainHLGH0118	963	--	963
99.OM714565.1_CurvulariaplantarumstrainM0134	983	--	983
100.MN159911.1_BotrytiscinereaSICAUCC19-0003	931	--	931
102.GU294713.1LasiodiplodiatheobromaestrainUCD2430TX	407	--	407
103.KX868094.1_Mycosphaerellasp.isolateCRM20.1	475	--	475
104.LC599478.1Pseudocercosporapini-densifloraeMUCC534	310	--	310
105.N584698.1BipolarissetariaestrainKBS4-2	1031	--	1031
2.OM160859.1_F.buharicum	560	--	560
1.LC727524.1_F.buharicum_OKI-1_Okura	682	--	682
3.KX302919.1_F.sublunatum	521	--	521
4.LT996094.1_F.convolutans	516	--	516
5.OM160861.1_F.abutilonis	630	--	630
6.OM160874.1_F.guadeloupense	560	--	560
7.MH392475.1_F.graminearum	430	--	430
8.MH582420.1_F.solani	540	--	540

```
9.MAFF244605_F.oxysporum                                                  544 ---------------------------------------- 544
10.MAFF237278_F.contaminatum_Hylocereus                                   711 ---------------------------------------- 711
11.MAFF237649_F.concentricum__Riceroot                                    678 ---------------------------------------- 678
12.MAFF237650_F.concentricum__Wheat                                       679 ---------------------------------------- 679
13.MAFF239869_F.mangiferae__Ryukyupine                                    657 ---------------------------------------- 657
14.MAFF240460_F.fujikuroi_Passionfruit                                    661 ---------------------------------------- 661
15.MAFF241317_F.graminearum_Wheat                                         666 ---------------------------------------- 666
16.MAFF242670_F.ipomoeae_Wheat                                            672 ---------------------------------------- 672
17.MAFF245129_F.concentricum_Fraxinus                                     659 ---------------------------------------- 659
18.MAFF245395_F.cugenangense_Rhubarb                                      671 ---------------------------------------- 671
19.MAFF246637_F.nirenbergiae_Strawberry                                   671 ---------------------------------------- 671
20.MAFF246672_F.nirenbergiae_ChinesePeony                                 671 ---------------------------------------- 671
21.MAFF246697_F.commune_Urallicoriceroot                                  673 ---------------------------------------- 673
22.MAFF246729_F.falciforme_Angelica                                       691 ---------------------------------------- 691
23.MAFF247220_F.duplospermum__Euwallaceasp                                694 ---------------------------------------- 694
24.MAFF410760_F.odoratissimum_alpha                                       669 ---------------------------------------- 669
25.MAFF244605_FusariumoxysporumSchlechtendal_MAFF244605_Tomato            672 ---------------------------------------- 672
26.MAFF241326_F.asiaticum_Wheat                                           669 ---------------------------------------- 669
27.MAFF245014_F.asiaticum_Wildsoybean                                     666 ---------------------------------------- 666
28.MAFF150124_F.asiaticum__Wheat                                          666 ---------------------------------------- 666
29.OM135603.1F.algeriense                                                 699 ---------------------------------------- 699
30.MAFF237465_F.penzigii_Aloe                                             704 ---------------------------------------- 704
31.MAFF103054_F.oxysporumSchlechtendalf.sp.cucumerinum_Cucumber           671 ---------------------------------------- 671
32.MAFF712246_F.oxysporumSchlechtendalf.sp.dianthi__Carnation             673 ---------------------------------------- 673
33.MAFF305558_F.oxysporumSchlechtendalf.sp.fragariae__Watermelon          671 ---------------------------------------- 671
34.MAFF744087_F.oxysporumSchlechtendalf.sp.lactucae__Lettuce              671 ---------------------------------------- 671
35.MAFF726924_F.oxysporumSchlechtendalf.sp.lagenariae_Whitefloweredgourd  671 ---------------------------------------- 671
36.MAFF744003_F.oxysporumSchlechtendalf.sp.lagenariae_Squash)             672 ---------------------------------------- 672
37.MAFF305122_F.oxysporumSchlechtendalf.sp.melonis__Melon                 671 ---------------------------------------- 671
38.MAFF306714_F.oxysporumSchlechtendalf.sp.momordicae_Balsampear          671 ---------------------------------------- 671
39.MAFF238905_F.oxysporumSchlechtendalf.sp.radicis-lycopersici_Tomato     674 ---------------------------------------- 674
40.MAFF150004_F.oxysporumSchlechtendalf.sp.spinaciae_Spinach              671 ---------------------------------------- 671
41.MAFF247034_F.oxysporumSchlechtendal__Goldenchain                       671 ---------------------------------------- 671
42.MAFF245747_F.oxysporumSchlechtendalf.sp.callistephi__Chinaaster        671 ---------------------------------------- 671
43.MAFF305115_FoxysporumSchlechtendalf.sp.batatas__Sweatpotato            703 ---------------------------------------- 703
44.MAFF150126_F.asiaticum_Seed                                            666 ---------------------------------------- 666
45.MAFF246738_F.solani_Angelica                                           703 ---------------------------------------- 703
46.MAFF246664_F.cugenangense_Perilla                                      671 ---------------------------------------- 671
47.MH582420.1F.solanistrainMRC256                                         679 ---------------------------------------- 679
48.MAFF240361_F.babinda_Soil                                              693 ---------------------------------------- 693
49.MAFF242368_F.azukicola_Azukibean                                       708 ---------------------------------------- 708
50.MAFF241312_F.asiaticum_Soil,welshonionfield                            666 ---------------------------------------- 666
51.LT548416.1_F.culmorumpartialtef1a                                      646 ---------------------------------------- 646
52.MAFF150124_F.asiaticum__Wheat                                          666 ---------------------------------------- 666
53.MAFF238806_F.begoniae_Oncidiumsp                                       705 ---------------------------------------- 705
54.MW594399.1_FusariumincarnatumisolateUD01C                              689 ---------------------------------------- 689
```

```
55.OP414923.1Pucciniagraminisf.sp.triticiisolateSHZPgt19                  623 ---------------------------------------- 623
56.MT027094.1_BipolarisoryzaestrainOrL-2                                  895 ---------------------------------------- 895
57.ON734360.1_AlternariaalternataisolateH126                              831 ---------------------------------------- 831
58.LC333578.1_StemphyliumlycopersiciSOasp2                                265 ---------------------------------------- 265
59.HQ718583.1_Colletotrichumgloeosporioidesisolateq-1                     567 ---------------------------------------- 567
60.JN241603.1_AtheliarolfsiiisolateSR1                                   1013 ---------------------------------------- 1013
61.KJ866474.1_RhizoctoniasolanistrainMHL-1                                683 ---------------------------------------- 683
62.JQ672424.1AlternariatriticinaisolateEGS17-061                          825 ---------------------------------------- 825
63.LT707559.1_P.capsicipartialtef1agene                                   964 ---------------------------------------- 964
64.MW090051.1_CurvularialunatastrainCls-3                                 908 ---------------------------------------- 908
65.DQ400892.1_Aspergillusterreus                                          452 ---------------------------------------- 452
66.DQ911416.1_Pythiumsp.quercumstrainPy292                                932 ---------------------------------------- 932
67.EU797495.1_Phytophthorasp.oaksoilPoland                                930 ---------------------------------------- 930
68.HM148321.1_Cladosporiumcucumerinum                                     385 ---------------------------------------- 385
69.AF398888.1_SclerotiniasclerotiorumisolateSS1                           334 ---------------------------------------- 334
70.AF398888.1_S.sclerotiorumisolateSS1                                    334 ---------------------------------------- 334
71.HPAB545908.1_Verticilliumnonalfalfaeisolate                            442 ---------------------------------------- 442
72.EF433315.1_CeratocystisfimbriatavoucherCMW15052                        747 ---------------------------------------- 747
73.MN159912.1_Botrytiscinerea                                             931 ---------------------------------------- 931
74.MF034741.1_PeltasterfructicolaisolateSRB92                             317 ---------------------------------------- 317
75.LC440360.1_CercosporaasparagiCOasp2                                    297 ---------------------------------------- 297
76.AY944105.1_MagnaportheoryzaeisolateSAG00T3()                           255 ---------------------------------------- 255
77.JX266586.1_CochliobolusmiyabeanusvoucherMFLUCC10-0733                  918 ---------------------------------------- 918
78.MN393253.1_CorynesporacassiicolaisolateQHD001(MN393253.1UNVERIFIED)    305 ---------------------------------------- 305
79.MF375218.1_AgroatheliarolfsiiisolateBJB24                              544 ---------------------------------------- 544
80.MN106270.1_AgroatheliarolfsiistrainJ-12                                509 ---------------------------------------- 509
81.OQ732628.1_AgroatheliarolfsiiisolateBTCBSr3                            499 ---------------------------------------- 499
82.KY196185.1_ColletotrichumtruncatumstrainPAK53                          468 ---------------------------------------- 468
83.GU935835.1_ColletotrichumcoccodesisolateC96002                        1041 ---------------------------------------- 1041
84.MK085963.1_AlternariatenuissimaisolateSCCZ06                           252 ---------------------------------------- 252
85.MT548042.1_AlternarialongipesstrainKY_2019_012                         280 ---------------------------------------- 280
86.MN356465.1CalonectriamontanaisolateHSP4                                489 ---------------------------------------- 489
87.OL694224.1_CalonectriacanadianastrainF099                              504 ---------------------------------------- 504
88.MK803351.1_NeoscytalidiumdimidiatumstrainKale4-C                       299 ---------------------------------------- 299
89.ON376993.1_CurvulariachiangmaiensisisolateND00J7                       938 ---------------------------------------- 938
90.OQ383346.1_NeoscytalidiumdimidiatumisolateGKH-2                        297 ---------------------------------------- 297
91.MF662595.1_NeoscytalidiumnovaehollandiaeisolateNeNo1                   284 ---------------------------------------- 284
92.EF560588.1Melampsoralini                                               656 ---------------------------------------- 656
93.LC590862.1_NeoscytalidiumdimidiatumPSU-HP01TEF1                        267 ---------------------------------------- 267
94.KX278106.1_BotryosphaeriaqingyuanensisstrainCERC2947                   284 ---------------------------------------- 284
95.AJ578763.1_Blumeriagraminisf.sp.hordeicyp51                           1458 GGGCAGCAAGCCCTTACCTACCGTTTGGGGCCGGACGGCA 1497
96.MF490858.1_CurvulariadactylocteniicolastrainCPC28810                   930 ---------------------------------------- 930
97.KT287115.1_Bipolariscactivoraisolate3.8.6                             1006 ---------------------------------------- 1006
98.MT560940.1_CurvulariacactivorastrainHLGH0118                           963 ---------------------------------------- 963
99.OM714565.1_CurvulariaplantarumstrainM0134                              983 ---------------------------------------- 983
100.MN159911.1_BotrytiscinereaSICAUCC19-0003                              931 ---------------------------------------- 931
```

102.GU294713.1LasiodiplodiatheobromaestrainUCD2430TX	407	--	407
103.KX868094.1_Mycosphaerellasp.isolateCRM20.1	475	--	475
104.LC599478.1Pseudocercosporapini-densifloraeMUCC534	310	--	310
105.N584698.1BipolarissetariaestrainKBS4-2	1031	--	1031
2.OM160859.1_F.buharicum	560	--	560
1.LC727524.1_F.buharicum_OKI-1_Okura	682	--	682
3.KX302919.1_F.sublunatum	521	--	521
4.LT996094.1_F.convolutans	516	--	516
5.OM160861.1_F.abutilonis	630	--	630
6.OM160874.1_F.guadeloupense	560	--	560
7.MH392475.1_F.graminearum	430	--	430
8.MH582420.1_F.solani	540	--	540
9.MAFF244605_F.oxysporum	544	--	544
10.MAFF237278_F.contaminatum_Hylocereus	711	--	711
11.MAFF237649_F.concentricum__Riceroot	678	--	678
12.MAFF237650_F.concentricum__Wheat	679	--	679
13.MAFF239869_F.mangiferae__Ryukyupine	657	--	657
14.MAFF240460_F.fujikuroi_Passionfruit	661	--	661
15.MAFF241317_F.graminearum_Wheat	666	--	666
16.MAFF242670_F.ipomoeae_Wheat	672	--	672
17.MAFF245129_F.concentricum_Fraxinus	659	--	659
18.MAFF245395_F.cugenangense_Rhubarb	671	--	671
19.MAFF246637_F.nirenbergiae_Strawberry	671	--	671
20.MAFF246672_F.nirenbergiae_ChinesePeony	671	--	671
21.MAFF246697_F.commune_Urallicoriceroot	673	--	673
22.MAFF246729_F.falciforme_Angelica	691	--	691
23.MAFF247220_F.duplospermum__Euwallaceasp	694	--	694
24.MAFF410760_F.odoratissimum_alpha	669	--	669
25.MAFF244605_FusariumoxysporumSchlechtendal_MAFF244605_Tomato	672	--	672
26.MAFF241326_F.asiaticum_Wheat	669	--	669
27.MAFF245014_F.asiaticum_Wildsoybean	666	--	666
28.MAFF150124_F.asiaticum__Wheat	666	--	666
29.OM135603.1F.algeriense	699	--	699
30.MAFF237465_F.penzigii_Aloe	704	--	704
31.MAFF103054_F.oxysporumSchlechtendalf.sp.cucumerinum_Cucumber	671	--	671
32.MAFF712246_F.oxysporumSchlechtendalf.sp.dianthi__Carnation	673	--	673
33.MAFF305558_F.oxysporumSchlechtendalf.sp.fragariae__Watermelon	671	--	671
34.MAFF744087_F.oxysporumSchlechtendalf.sp.lactucae__Lettuce	671	--	671
35.MAFF726924_F.oxysporumSchlechtendalf.sp.lagenariae_Whiteflower edgourd	671	--	671
36.MAFF744003_F.oxysporumSchlechtendalf.sp.lagenariae_Squash)	672	--	672
37.MAFF305122_F.oxysporumSchlechtendalf.sp.melonis__Melon	671	--	671
38.MAFF306714_F.oxysporumSchlechtendalf.sp.momordicae_Balsampear	671	--	671
39.MAFF238905_F.oxysporumSchlechtendalf.sp.radicis-lycopersici_Tomato	674	--	674
40.MAFF150004_F.oxysporumSchlechtendalf.sp.spinaciae_Spinach	671	--	671
41.MAFF247034_F.oxysporumSchlechtendal__Goldenchain	671	--	671

```
42.MAFF245747_F.oxysporumSchlechtendalf.sp.callistephi__Chinaaster        671 ---------------------------------------- 671
43.MAFF305115_FoxysporumSchlechtendalf.sp.batatas__Sweatpotato            703 ---------------------------------------- 703
44.MAFF150126_F.asiaticum_Seed                                            666 ---------------------------------------- 666
45.MAFF246738_F.solani_Angelica                                           703 ---------------------------------------- 703
46.MAFF246664_F.cugenangense_Perilla                                      671 ---------------------------------------- 671
47.MH582420.1F.solanistrainMRC256                                         679 ---------------------------------------- 679
48.MAFF240361_F.babinda_Soil                                              693 ---------------------------------------- 693
49.MAFF242368_F.azukicola_Azukibean                                       708 ---------------------------------------- 708
50.MAFF241312_F.asiaticum_Soil,welshonionfield                            666 ---------------------------------------- 666
51.LT548416.1_F.culmorumpartialtef1a                                      646 ---------------------------------------- 646
52.MAFF150124_F.asiaticum__Wheat                                          666 ---------------------------------------- 666
53.MAFF238806_F.begoniae_Oncidiumsp                                       705 ---------------------------------------- 705
54.MW594399.1_FusariumincarnatumisolateUD01C                              689 ---------------------------------------- 689
55.OP414923.1Pucciniagraminisf.sp.triticiisolateSHZPgt19                  623 ---------------------------------------- 623
56.MT027094.1_BipolarisoryzaestrainOrL-2                                  895 ---------------------------------------- 895
57.ON734360.1_AlternariaalternataisolateH126                              831 ---------------------------------------- 831
58.LC333578.1_StemphyliumlycopersiciSOasp2                                265 ---------------------------------------- 265
59.HQ718583.1_Colletotrichumgloeosporioidesisolateq-1                     567 ---------------------------------------- 567
60.JN241603.1_AtheliarolfsiiisolateSR1                                   1013 ---------------------------------------- 1013
61.KJ866474.1_RhizoctoniasolanistrainMHL-1                                683 ---------------------------------------- 683
62.JQ672424.1AlternariatriticinaisolateEGS17-061                          825 ---------------------------------------- 825
63.LT707559.1_P.capsicipartialtef1agene                                   964 ---------------------------------------- 964
64.MW090051.1_CurvularialunatastrainCls-3                                 908 ---------------------------------------- 908
65.DQ400892.1_Aspergillusterreus                                          452 ---------------------------------------- 452
66.DQ911416.1_Pythiumsp.quercumstrainPy292                                932 ---------------------------------------- 932
67.EU797495.1_Phytophthorasp.oaksoilPoland                                930 ---------------------------------------- 930
68.HM148321.1_Cladosporiumcucumerinum                                     385 ---------------------------------------- 385
69.AF398888.1_SclerotiniasclerotiorumisolateSS1                           334 ---------------------------------------- 334
70.AF398888.1_S.sclerotiorumisolateSS1                                    334 ---------------------------------------- 334
71.HPAB545908.1_Verticilliumnonalfalfaeisolate                            442 ---------------------------------------- 442
72.EF433315.1_CeratocystisfimbriatavoucherCMW15052                        747 ---------------------------------------- 747
73.MN159912.1_Botrytiscinerea                                             931 ---------------------------------------- 931
74.MF034741.1_PeltasterfructicolaisolateSRB92                             317 ---------------------------------------- 317
75.LC440360.1_CercosporaasparagiCOasp2                                    297 ---------------------------------------- 297
76.AY944105.1_MagnaportheoryzaeisolateSAG00T3()                           255 ---------------------------------------- 255
77.JX266586.1_CochliobolusmiyabeanusvoucherMFLUCC10-0733                  918 ---------------------------------------- 918
78.MN393253.1_CorynesporacassiicolaisolateQHD001(MN393253.1UNVERIFIED)    305 ---------------------------------------- 305
79.MF375218.1_AgroatheliarolfsiiisolateBJB24                              544 ---------------------------------------- 544
80.MN106270.1_AgroatheliarolfsiistrainJ-12                                509 ---------------------------------------- 509
81.OQ732628.1_AgroatheliarolfsiiisolateBTCBSr3                            499 ---------------------------------------- 499
82.KY196185.1_ColletotrichumtruncatumstrainPAK53                          468 ---------------------------------------- 468
83.GU935835.1_ColletotrichumcoccodesisolateC96002                        1041 ---------------------------------------- 1041
84.MK085963.1_AlternariatenuissimaisolateSCCZ06                           252 ---------------------------------------- 252
85.MT548042.1_AlternarialongipesstrainKY_2019_012                         280 ---------------------------------------- 280
86.MN356465.1CalonectriamontanaisolateHSP4                                489 ---------------------------------------- 489
87.OL694224.1_CalonectriacanadianastrainF099                              504 ---------------------------------------- 504
```

88.MK803351.1_NeoscytalidiumdimidiatumstrainKale4-C	299	--	299
89.ON376993.1_CurvulariachiangmaiensisisolateND00J7	938	--	938
90.OQ383346.1_NeoscytalidiumdimidiatumisolateGKH-2	297	--	297
91.MF662595.1_NeoscytalidiumnovaehollandiaeisolateNeNo1	284	--	284
92.EF560588.1Melampsoralini	656	--	656
93.LC590862.1_NeoscytalidiumdimidiatumPSU-HP01TEF1	267	--	267
94.KX278106.1_BotryosphaeriaqingyuanensisstrainCERC2947	284	--	284
95.AJ578763.1_Blumeriagraminisf.sp.hordeicyp51	1498	TCGCTGCATAGGCGAGCAATTTGCAACGGTGCAATTAGTT	1537
96.MF490858.1_CurvulariadactylocteniicolastrainCPC28810	930	--	930
97.KT287115.1_Bipolariscactivoraisolate3.8.6	1006	--	1006
98.MT560940.1_CurvulariacactivorastrainHLGH0118	963	--	963
99.OM714565.1_CurvulariaplantarumstrainM0134	983	--	983
100.MN159911.1_BotrytiscinereaSICAUCC19-0003	931	--	931
102.GU294713.1LasiodiplodiatheobromaestrainUCD2430TX	407	--	407
103.KX868094.1_Mycosphaerellasp.isolateCRM20.1	475	--	475
104.LC599478.1Pseudocercosporapini-densifloraeMUCC534	310	--	310
105.N584698.1BipolarissetariaestrainKBS4-2	1031	--	1031
2.OM160859.1_F.buharicum	560	--	560
1.LC727524.1_F.buharicum_OKI-1_Okura	682	--	682
3.KX302919.1_F.sublunatum	521	--	521
4.LT996094.1_F.convolutans	516	--	516
5.OM160861.1_F.abutilonis	630	--	630
6.OM160874.1_F.guadeloupense	560	--	560
7.MH392475.1_F.graminearum	430	--	430
8.MH582420.1_F.solani	540	--	540
9.MAFF244605_F.oxysporum	544	--	544
10.MAFF237278_F.contaminatum_Hylocereus	711	--	711
11.MAFF237649_F.concentricum__Riceroot	678	--	678
12.MAFF237650_F.concentricum__Wheat	679	--	679
13.MAFF239869_F.mangiferae__Ryukyupine	657	--	657
14.MAFF240460_F.fujikuroi_Passionfruit	661	--	661
15.MAFF241317_F.graminearum_Wheat	666	--	666
16.MAFF242670_F.ipomoeae_Wheat	672	--	672
17.MAFF245129_F.concentricum_Fraxinus	659	--	659
18.MAFF245395_F.cugenangense_Rhubarb	671	--	671
19.MAFF246637_F.nirenbergiae_Strawberry	671	--	671
20.MAFF246672_F.nirenbergiae_ChinesePeony	671	--	671
21.MAFF246697_F.commune_Urallicoriceroot	673	--	673
22.MAFF246729_F.falciforme_Angelica	691	--	691
23.MAFF247220_F.duplospermum__Euwallaceasp	694	--	694
24.MAFF410760_F.odoratissimum_alpha	669	--	669
25.MAFF244605_FusariumoxysporumSchlechtendal_MAFF244605_Tomato	672	--	672
26.MAFF241326_F.asiaticum_Wheat	669	--	669
27.MAFF245014_F.asiaticum_Wildsoybean	666	--	666
28.MAFF150124_F.asiaticum__Wheat	666	--	666

```
29.OM135603.1F.algeriense                                                  699 ---------------------------------------- 699
30.MAFF237465_F.penzigii_Aloe                                              704 ---------------------------------------- 704
31.MAFF103054_F.oxysporumSchlechtendalf.sp.cucumerinum_Cucumber            671 ---------------------------------------- 671
32.MAFF712246_F.oxysporumSchlechtendalf.sp.dianthi__Carnation              673 ---------------------------------------- 673
33.MAFF305558_F.oxysporumSchlechtendalf.sp.fragariae__Watermelon           671 ---------------------------------------- 671
34.MAFF744087_F.oxysporumSchlechtendalf.sp.lactucae__Lettuce               671 ---------------------------------------- 671
35.MAFF726924_F.oxysporumSchlechtendalf.sp.lagenariae_Whiteflowered gourd  671 ---------------------------------------- 671
36.MAFF744003_F.oxysporumSchlechtendalf.sp.lagenariae_Squash)              672 ---------------------------------------- 672
37.MAFF305122_F.oxysporumSchlechtendalf.sp.melonis__Melon                  671 ---------------------------------------- 671
38.MAFF306714_F.oxysporumSchlechtendalf.sp.momordicae_Balsampear           671 ---------------------------------------- 671
39.MAFF238905_F.oxysporumSchlechtendalf.sp.radicis-lycopersici_Tomato      674 ---------------------------------------- 674
40.MAFF150004_F.oxysporumSchlechtendalf.sp.spinaciae_Spinach               671 ---------------------------------------- 671
41.MAFF247034_F.oxysporumSchlechtendal__Goldenchain                        671 ---------------------------------------- 671
42.MAFF245747_F.oxysporumSchlechtendalf.sp.callistephi__Chinaaster         671 ---------------------------------------- 671
43.MAFF305115_FoxysporumSchlechtendalf.sp.batatas__Sweatpotato             703 ---------------------------------------- 703
44.MAFF150126_F.asiaticum_Seed                                             666 ---------------------------------------- 666
45.MAFF246738_F.solani_Angelica                                            703 ---------------------------------------- 703
46.MAFF246664_F.cugenangense_Perilla                                       671 ---------------------------------------- 671
47.MH582420.1F.solanistrainMRC256                                          679 ---------------------------------------- 679
48.MAFF240361_F.babinda_Soil                                               693 ---------------------------------------- 693
49.MAFF242368_F.azukicola_Azukibean                                        708 ---------------------------------------- 708
50.MAFF241312_F.asiaticum_Soil,welshonionfield                             666 ---------------------------------------- 666
51.LT548416.1_F.culmorumpartialtef1a                                       646 ---------------------------------------- 646
52.MAFF150124_F.asiaticum__Wheat                                           666 ---------------------------------------- 666
53.MAFF238806_F.begoniae_Oncidiumsp                                        705 ---------------------------------------- 705
54.MW594399.1_FusariumincarnatumisolateUD01C                               689 ---------------------------------------- 689
55.OP414923.1Pucciniagraminisf.sp.triticiisolateSHZPgt19                   623 ---------------------------------------- 623
56.MT027094.1_BipolarisoryzaestrainOrL-2                                   895 ---------------------------------------- 895
57.ON734360.1_AlternariaalternataisolateH126                               831 ---------------------------------------- 831
58.LC333578.1_StemphyliumlycopersiciSOasp2                                 265 ---------------------------------------- 265
59.HQ718583.1_Colletotrichumgloeosporioidesisolateq-1                      567 ---------------------------------------- 567
60.JN241603.1_AtheliarolfsiiisolateSR1                                    1013 ---------------------------------------- 1013
61.KJ866474.1_RhizoctoniasolanistrainMHL-1                                 683 ---------------------------------------- 683
62.JQ672424.1AlternariatriticinaisolateEGS17-061                           825 ---------------------------------------- 825
63.LT707559.1_P.capsicipartialtef1agene                                    964 ---------------------------------------- 964
64.MW090051.1_CurvularialunatastrainCls-3                                  908 ---------------------------------------- 908
65.DQ400892.1_Aspergillusterreus                                           452 ---------------------------------------- 452
66.DQ911416.1_Pythiumsp.quercumstrainPy292                                 932 ---------------------------------------- 932
67.EU797495.1_Phytophthorasp.oaksoilPoland                                 930 ---------------------------------------- 930
68.HM148321.1_Cladosporiumcucumerinum                                      385 ---------------------------------------- 385
69.AF398888.1_SclerotiniasclerotiorumisolateSS1                            334 ---------------------------------------- 334
70.AF398888.1_S.sclerotiorumisolateSS1                                     334 ---------------------------------------- 334
71.HPAB545908.1_Verticilliumnonalfalfaeisolate                             442 ---------------------------------------- 442
72.EF433315.1_CeratocystisfimbriatavoucherCMW15052                         747 ---------------------------------------- 747
73.MN159912.1_Botrytiscinerea                                              931 ---------------------------------------- 931
74.MF034741.1_PeltasterfructicolaisolateSRB92                              317 ---------------------------------------- 317
```

```
75.LC440360.1_CercosporaasparagiCOasp2                              297 ---------------------------------------- 297
76.AY944105.1_MagnaportheoryzaeisolateSAG00T3()                     255 ---------------------------------------- 255
77.JX266586.1_CochliobolusmiyabeanusvoucherMFLUCC10-0733            918 ---------------------------------------- 918
78.MN393253.1_CorynesporacassiicolaisolateQHD001(MN393253.1UNVERIFIED) 305 ---------------------------------------- 305
79.MF375218.1_AgroatheliarolfsiiisolateBJB24                        544 ---------------------------------------- 544
80.MN106270.1_AgroatheliarolfsiistrainJ-12                          509 ---------------------------------------- 509
81.OQ732628.1_AgroatheliarolfsiiisolateBTCBSr3                      499 ---------------------------------------- 499
82.KY196185.1_ColletotrichumtruncatumstrainPAK53                    468 ---------------------------------------- 468
83.GU935835.1_ColletotrichumcoccodesisolateC96002                  1041 ---------------------------------------- 1041
84.MK085963.1_AlternariatenuissimaisolateSCCZ06                     252 ---------------------------------------- 252
85.MT548042.1_AlternarialongipesstrainKY_2019_012                   280 ---------------------------------------- 280
86.MN356465.1CalonectriamontanaisolateHSP4                          489 ---------------------------------------- 489
87.OL694224.1_CalonectriacanadianastrainF099                        504 ---------------------------------------- 504
88.MK803351.1_NeoscytalidiumdimidiatumstrainKale4-C                 299 ---------------------------------------- 299
89.ON376993.1_CurvulariachiangmaiensisisolateND00J7                 938 ---------------------------------------- 938
90.OQ383346.1_NeoscytalidiumdimidiatumisolateGKH-2                  297 ---------------------------------------- 297
91.MF662595.1_NeoscytalidiumnovaehollandiaeisolateNeNo1             284 ---------------------------------------- 284
92.EF560588.1Melampsoralini                                         656 ---------------------------------------- 656
93.LC590862.1_NeoscytalidiumdimidiatumPSU-HP01TEF1                  267 ---------------------------------------- 267
94.KX278106.1_BotryosphaeriaqingyuanensisstrainCERC2947             284 ---------------------------------------- 284
95.AJ578763.1_Blumeriagraminisf.sp.hordeicyp51                     1538 ACAATTATGGCCACCATGGTTCGCAGTTTCAAGTTTCACA 1577
96.MF490858.1_CurvulariadactylocteniicolastrainCPC28810             930 ---------------------------------------- 930
97.KT287115.1_Bipolariscactivoraisolate3.8.6                       1006 ---------------------------------------- 1006
98.MT560940.1_CurvulariacactivorastrainHLGH0118                     963 ---------------------------------------- 963
99.OM714565.1_CurvulariaplantarumstrainM0134                        983 ---------------------------------------- 983
100.MN159911.1_BotrytiscinereaSICAUCC19-0003                        931 ---------------------------------------- 931
102.GU294713.1LasiodiplodiatheobromaestrainUCD2430TX                407 ---------------------------------------- 407
103.KX868094.1_Mycosphaerellasp.isolateCRM20.1                      475 ---------------------------------------- 475
104.LC599478.1Pseudocercosporapini-densifloraeMUCC534               310 ---------------------------------------- 310
105.N584698.1BipolarissetariaestrainKBS4-2                         1031 ---------------------------------------- 1031

2.OM160859.1_F.buharicum                                            560 ---------------------------------------- 560
1.LC727524.1_F.buharicum_OKI-1_Okura                                682 ---------------------------------------- 682
3.KX302919.1_F.sublunatum                                           521 ---------------------------------------- 521
4.LT996094.1_F.convolutans                                          516 ---------------------------------------- 516
5.OM160861.1_F.abutilonis                                           630 ---------------------------------------- 630
6.OM160874.1_F.guadeloupense                                        560 ---------------------------------------- 560
7.MH392475.1_F.graminearum                                          430 ---------------------------------------- 430
8.MH582420.1_F.solani                                               540 ---------------------------------------- 540
9.MAFF244605_F.oxysporum                                            544 ---------------------------------------- 544
10.MAFF237278_F.contaminatum_Hylocereus                             711 ---------------------------------------- 711
11.MAFF237649_F.concentricum__Riceroot                              678 ---------------------------------------- 678
12.MAFF237650_F.concentricum__Wheat                                 679 ---------------------------------------- 679
13.MAFF239869_F.mangiferae__Ryukyupine                              657 ---------------------------------------- 657
14.MAFF240460_F.fujikuroi_Passionfruit                              661 ---------------------------------------- 661
15.MAFF241317_F.graminearum_Wheat                                   666 ---------------------------------------- 666
```

16.MAFF242670_F.ipomoeae_Wheat	672	--	672
17.MAFF245129_F.concentricum_Fraxinus	659	--	659
18.MAFF245395_F.cugenangense_Rhubarb	671	--	671
19.MAFF246637_F.nirenbergiae_Strawberry	671	--	671
20.MAFF246672_F.nirenbergiae_ChinesePeony	671	--	671
21.MAFF246697_F.commune_Urallicoriceroot	673	--	673
22.MAFF246729_F.falciforme_Angelica	691	--	691
23.MAFF247220_F.duplospermum__Euwallaceasp	694	--	694
24.MAFF410760_F.odoratissimum_alpha	669	--	669
25.MAFF244605_FusariumoxysporumSchlechtendal_MAFF244605_Tomato	672	--	672
26.MAFF241326_F.asiaticum_Wheat	669	--	669
27.MAFF245014_F.asiaticum_Wildsoybean	666	--	666
28.MAFF150124_F.asiaticum__Wheat	666	--	666
29.OM135603.1F.algeriense	699	--	699
30.MAFF237465_F.penzigii_Aloe	704	--	704
31.MAFF103054_F.oxysporumSchlechtendalf.sp.cucumerinum_Cucumber	671	--	671
32.MAFF712246_F.oxysporumSchlechtendalf.sp.dianthi__Carnation	673	--	673
33.MAFF305558_F.oxysporumSchlechtendalf.sp.fragariae__Watermelon	671	--	671
34.MAFF744087_F.oxysporumSchlechtendalf.sp.lactucae__Lettuce	671	--	671
35.MAFF726924_F.oxysporumSchlechtendalf.sp.lagenariae_Whitefloweredgourd	671	--	671
36.MAFF744003_F.oxysporumSchlechtendalf.sp.lagenariae_Squash)	672	--	672
37.MAFF305122_F.oxysporumSchlechtendalf.sp.melonis__Melon	671	--	671
38.MAFF306714_F.oxysporumSchlechtendalf.sp.momordicae_Balsampear	671	--	671
39.MAFF238905_F.oxysporumSchlechtendalf.sp.radicis-lycopersici_Tomato	674	--	674
40.MAFF150004_F.oxysporumSchlechtendalf.sp.spinaciae_Spinach	671	--	671
41.MAFF247034_F.oxysporumSchlechtendal__Goldenchain	671	--	671
42.MAFF245747_F.oxysporumSchlechtendalf.sp.callistephi__Chinaaster	671	--	671
43.MAFF305115_FoxysporumSchlechtendalf.sp.batatas__Sweatpotato	703	--	703
44.MAFF150126_F.asiaticum_Seed	666	--	666
45.MAFF246738_F.solani_Angelica	703	--	703
46.MAFF246664_F.cugenangense_Perilla	671	--	671
47.MH582420.1F.solanistrainMRC256	679	--	679
48.MAFF240361_F.babinda_Soil	693	--	693
49.MAFF242368_F.azukicola_Azukibean	708	--	708
50.MAFF241312_F.asiaticum_Soil,welshonionfield	666	--	666
51.LT548416.1_F.culmorumpartialtef1a	646	--	646
52.MAFF150124_F.asiaticum__Wheat	666	--	666
53.MAFF238806_F.begoniae_Oncidiumsp	705	--	705
54.MW594399.1_FusariumincarnatumisolateUD01C	689	--	689
55.OP414923.1Pucciniagraminisf.sp.triticiisolateSHZPgt19	623	--	623
56.MT027094.1_BipolarisoryzaestrainOrL-2	895	--	895
57.ON734360.1_AlternariaalternataisolateH126	831	--	831
58.LC333578.1_StemphyliumlycopersiciSOasp2	265	--	265
59.HQ718583.1_Colletotrichumgloeosporioidesisolateq-1	567	--	567
60.JN241603.1_AtheliarolfsiiisolateSR1	1013	--	1013
61.KJ866474.1_RhizoctoniasolanistrainMHL-1	683	--	683

```
62.JQ672424.1AlternariatriticinaisolateEGS17-061                     825 ---------------------------------------- 825
63.LT707559.1_P.capsicipartialtef1agene                              964 ---------------------------------------- 964
64.MW090051.1_CurvularialunatastrainCls-3                            908 ---------------------------------------- 908
65.DQ400892.1_Aspergillusterreus                                     452 ---------------------------------------- 452
66.DQ911416.1_Pythiumsp.quercumstrainPy292                           932 ---------------------------------------- 932
67.EU797495.1_Phytophthorasp.oaksoilPoland                           930 ---------------------------------------- 930
68.HM148321.1_Cladosporiumcucumerinum                                385 ---------------------------------------- 385
69.AF398888.1_SclerotiniasclerotiorumisolateSS1                      334 ---------------------------------------- 334
70.AF398888.1_S.sclerotiorumisolateSS1                               334 ---------------------------------------- 334
71.HPAB545908.1_Verticilliumnonalfalfaeisolate                       442 ---------------------------------------- 442
72.EF433315.1_CeratocystisfimbriatavoucherCMW15052                   747 ---------------------------------------- 747
73.MN159912.1_Botrytiscinerea                                        931 ---------------------------------------- 931
74.MF034741.1_PeltasterfructicolaisolateSRB92                        317 ---------------------------------------- 317
75.LC440360.1_CercosporaasparagiCOasp2                               297 ---------------------------------------- 297
76.AY944105.1_MagnaportheoryzaeisolateSAG00T3()                      255 ---------------------------------------- 255
77.JX266586.1_CochliobolusmiyabeanusvoucherMFLUCC10-0733             918 ---------------------------------------- 918
78.MN393253.1_CorynesporacassiicolaisolateQHD001(MN393253.1UNVERIFIED) 305 ---------------------------------------- 305
79.MF375218.1_AgroatheliarolfsiiisolateBJB24                         544 ---------------------------------------- 544
80.MN106270.1_AgroatheliarolfsiistrainJ-12                           509 ---------------------------------------- 509
81.OQ732628.1_AgroatheliarolfsiiisolateBTCBSr3                       499 ---------------------------------------- 499
82.KY196185.1_ColletotrichumtruncatumstrainPAK53                     468 ---------------------------------------- 468
83.GU935835.1_ColletotrichumcoccodesisolateC96002                   1041 ---------------------------------------- 1041
84.MK085963.1_AlternariatenuissimaisolateSCCZ06                      252 ---------------------------------------- 252
85.MT548042.1_AlternarialongipesstrainKY_2019_012                    280 ---------------------------------------- 280
86.MN356465.1CalonectriamontanaisolateHSP4                           489 ---------------------------------------- 489
87.OL694224.1_CalonectriacanadianastrainF099                         504 ---------------------------------------- 504
88.MK803351.1_NeoscytalidiumdimidiatumstrainKale4-C                  299 ---------------------------------------- 299
89.ON376993.1_CurvulariachiangmaiensisisolateND00J7                  938 ---------------------------------------- 938
90.OQ383346.1_NeoscytalidiumdimidiatumisolateGKH-2                   297 ---------------------------------------- 297
91.MF662595.1_NeoscytalidiumnovaehollandiaeisolateNeNo1              284 ---------------------------------------- 284
92.EF560588.1Melampsoralini                                          656 ---------------------------------------- 656
93.LC590862.1_NeoscytalidiumdimidiatumPSU-HP01TEF1                   267 ---------------------------------------- 267
94.KX278106.1_BotryosphaeriaqingyuanensisstrainCERC2947              284 ---------------------------------------- 284
95.AJ578763.1_Blumeriagraminisf.sp.hordeicyp51                      1578 ACCTTGACGGAAGGAATAGCGTTGCCGAAACGGATTACTC 1617
96.MF490858.1_CurvulariadactylocteniicolastrainCPC28810              930 ---------------------------------------- 930
97.KT287115.1_Bipolariscactivoraisolate3.8.6                        1006 ---------------------------------------- 1006
98.MT560940.1_CurvulariacactivorastrainHLGH0118                      963 ---------------------------------------- 963
99.OM714565.1_CurvulariaplantarumstrainM0134                         983 ---------------------------------------- 983
100.MN159911.1_BotrytiscinereaSICAUCC19-0003                         931 ---------------------------------------- 931
102.GU294713.1LasiodiplodiatheobromaestrainUCD2430TX                 407 ---------------------------------------- 407
103.KX868094.1_Mycosphaerellasp.isolateCRM20.1                       475 ---------------------------------------- 475
104.LC599478.1Pseudocercosporapini-densifloraeMUCC534                310 ---------------------------------------- 310
105.N584698.1BipolarissetariaestrainKBS4-2                          1031 ---------------------------------------- 1031

2.OM160859.1_F.buharicum                                             560 ---------------------------------------- 560
1.LC727524.1_F.buharicum_OKI-1_Okura                                 682 ---------------------------------------- 682
```

```
3.KX302919.1_F.sublunatum 521 ---------------------------------------- 521
4.LT996094.1_F.convolutans 516 ---------------------------------------- 516
5.OM160861.1_F.abutilonis 630 ---------------------------------------- 630
6.OM160874.1_F.guadeloupense 560 ---------------------------------------- 560
7.MH392475.1_F.graminearum 430 ---------------------------------------- 430
8.MH582420.1_F.solani 540 ---------------------------------------- 540
9.MAFF244605_F.oxysporum 544 ---------------------------------------- 544
10.MAFF237278_F.contaminatum_Hylocereus 711 ---------------------------------------- 711
11.MAFF237649_F.concentricum__Riceroot 678 ---------------------------------------- 678
12.MAFF237650_F.concentricum__Wheat 679 ---------------------------------------- 679
13.MAFF239869_F.mangiferae__Ryukyupine 657 ---------------------------------------- 657
14.MAFF240460_F.fujikuroi_Passionfruit 661 ---------------------------------------- 661
15.MAFF241317_F.graminearum_Wheat 666 ---------------------------------------- 666
16.MAFF242670_F.ipomoeae_Wheat 672 ---------------------------------------- 672
17.MAFF245129_F.concentricum_Fraxinus 659 ---------------------------------------- 659
18.MAFF245395_F.cugenangense_Rhubarb 671 ---------------------------------------- 671
19.MAFF246637_F.nirenbergiae_Strawberry 671 ---------------------------------------- 671
20.MAFF246672_F.nirenbergiae_ChinesePeony 671 ---------------------------------------- 671
21.MAFF246697_F.commune_Urallicoriceroot 673 ---------------------------------------- 673
22.MAFF246729_F.falciforme_Angelica 691 ---------------------------------------- 691
23.MAFF247220_F.duplospermum__Euwallaceasp 694 ---------------------------------------- 694
24.MAFF410760_F.odoratissimum_alpha 669 ---------------------------------------- 669
25.MAFF244605_FusariumoxysporumSchlechtendal_MAFF244605_Tomato 672 ---------------------------------------- 672
26.MAFF241326_F.asiaticum_Wheat 669 ---------------------------------------- 669
27.MAFF245014_F.asiaticum_Wildsoybean 666 ---------------------------------------- 666
28.MAFF150124_F.asiaticum__Wheat 666 ---------------------------------------- 666
29.OM135603.1F.algeriense 699 ---------------------------------------- 699
30.MAFF237465_F.penzigii_Aloe 704 ---------------------------------------- 704
31.MAFF103054_F.oxysporumSchlechtendalf.sp.cucumerinum_Cucumber 671 ---------------------------------------- 671
32.MAFF712246_F.oxysporumSchlechtendalf.sp.dianthi__Carnation 673 ---------------------------------------- 673
33.MAFF305558_F.oxysporumSchlechtendalf.sp.fragariae__Watermelon 671 ---------------------------------------- 671
34.MAFF744087_F.oxysporumSchlechtendalf.sp.lactucae__Lettuce 671 ---------------------------------------- 671
35.MAFF726924_F.oxysporumSchlechtendalf.sp.lagenariae_Whitefloweredgourd 671 ---------------------------------------- 671
36.MAFF744003_F.oxysporumSchlechtendalf.sp.lagenariae_Squash) 672 ---------------------------------------- 672
37.MAFF305122_F.oxysporumSchlechtendalf.sp.melonis__Melon 671 ---------------------------------------- 671
38.MAFF306714_F.oxysporumSchlechtendalf.sp.momordicae_Balsampear 671 ---------------------------------------- 671
39.MAFF238905_F.oxysporumSchlechtendalf.sp.radicis-lycopersici_Tomato 674 ---------------------------------------- 674
40.MAFF150004_F.oxysporumSchlechtendalf.sp.spinaciae_Spinach 671 ---------------------------------------- 671
41.MAFF247034_F.oxysporumSchlechtendal__Goldenchain 671 ---------------------------------------- 671
42.MAFF245747_F.oxysporumSchlechtendalf.sp.callistephi__Chinaaster 671 ---------------------------------------- 671
43.MAFF305115_FoxysporumSchlechtendalf.sp.batatas__Sweatpotato 703 ---------------------------------------- 703
44.MAFF150126_F.asiaticum_Seed 666 ---------------------------------------- 666
45.MAFF246738_F.solani_Angelica 703 ---------------------------------------- 703
46.MAFF246664_F.cugenangense_Perilla 671 ---------------------------------------- 671
47.MH582420.1F.solanistrainMRC256 679 ---------------------------------------- 679
48.MAFF240361_F.babinda_Soil 693 ---------------------------------------- 693
```

```
49.MAFF242368_F.azukicola_Azukibean                                    708 ---------------------------------------- 708
50.MAFF241312_F.asiaticum_Soil,welshonionfield                         666 ---------------------------------------- 666
51.LT548416.1_F.culmorumpartialtef1a                                   646 ---------------------------------------- 646
52.MAFF150124_F.asiaticum__Wheat                                       666 ---------------------------------------- 666
53.MAFF238806_F.begoniae_Oncidiumsp                                    705 ---------------------------------------- 705
54.MW594399.1_FusariumincarnatumisolateUD01C                           689 ---------------------------------------- 689
55.OP414923.1Pucciniagraminisf.sp.triticiisolateSHZPgt19               623 ---------------------------------------- 623
56.MT027094.1_BipolarisoryzaestrainOrL-2                               895 ---------------------------------------- 895
57.ON734360.1_AlternariaalternataisolateH126                           831 ---------------------------------------- 831
58.LC333578.1_StemphyliumlycopersiciSOasp2                             265 ---------------------------------------- 265
59.HQ718583.1_Colletotrichumgloeosporioidesisolateq-1                  567 ---------------------------------------- 567
60.JN241603.1_AtheliarolfsiiisolateSR1                                1013 ---------------------------------------- 1013
61.KJ866474.1_RhizoctoniasolanistrainMHL-1                             683 ---------------------------------------- 683
62.JQ672424.1AlternariatriticinaisolateEGS17-061                       825 ---------------------------------------- 825
63.LT707559.1_P.capsicipartialtef1agene                                964 ---------------------------------------- 964
64.MW090051.1_CurvularialunatastrainCls-3                              908 ---------------------------------------- 908
65.DQ400892.1_Aspergillusterreus                                       452 ---------------------------------------- 452
66.DQ911416.1_Pythiumsp.quercumstrainPy292                             932 ---------------------------------------- 932
67.EU797495.1_Phytophthorasp.oaksoilPoland                             930 ---------------------------------------- 930
68.HM148321.1_Cladosporiumcucumerinum                                  385 ---------------------------------------- 385
69.AF398888.1_SclerotiniasclerotiorumisolateSS1                        334 ---------------------------------------- 334
70.AF398888.1_S.sclerotiorumisolateSS1                                 334 ---------------------------------------- 334
71.HPAB545908.1_Verticilliumnonalfalfaeisolate                         442 ---------------------------------------- 442
72.EF433315.1_CeratocystisfimbriatavoucherCMW15052                     747 ---------------------------------------- 747
73.MN159912.1_Botrytiscinerea                                          931 ---------------------------------------- 931
74.MF034741.1_PeltasterfructicolaisolateSRB92                          317 ---------------------------------------- 317
75.LC440360.1_CercosporaasparagiCOasp2                                 297 ---------------------------------------- 297
76.AY944105.1_MagnaportheoryzaeisolateSAG00T3()                        255 ---------------------------------------- 255
77.JX266586.1_CochliobolusmiyabeanusvoucherMFLUCC10-0733               918 ---------------------------------------- 918
78.MN393253.1_CorynesporacassiicolaisolateQHD001(MN393253.1UNVERIFIED) 305 ---------------------------------------- 305
79.MF375218.1_AgroatheliarolfsiiisolateBJB24                           544 ---------------------------------------- 544
80.MN106270.1_AgroatheliarolfsiistrainJ-12                             509 ---------------------------------------- 509
81.OQ732628.1_AgroatheliarolfsiiisolateBTCBSr3                         499 ---------------------------------------- 499
82.KY196185.1_ColletotrichumtruncatumstrainPAK53                       468 ---------------------------------------- 468
83.GU935835.1_ColletotrichumcoccodesisolateC96002                     1041 ---------------------------------------- 1041
84.MK085963.1_AlternariatenuissimaisolateSCCZ06                        252 ---------------------------------------- 252
85.MT548042.1_AlternarialongipesstrainKY_2019_012                      280 ---------------------------------------- 280
86.MN356465.1CalonectriamontanaisolateHSP4                             489 ---------------------------------------- 489
87.OL694224.1_CalonectriacanadianastrainF099                           504 ---------------------------------------- 504
88.MK803351.1_NeoscytalidiumdimidiatumstrainKale4-C                    299 ---------------------------------------- 299
89.ON376993.1_CurvulariachiangmaiensisisolateND00J7                    938 ---------------------------------------- 938
90.OQ383346.1_NeoscytalidiumdimidiatumisolateGKH-2                     297 ---------------------------------------- 297
91.MF662595.1_NeoscytalidiumnovaehollandiaeisolateNeNo1                284 ---------------------------------------- 284
92.EF560588.1Melampsoralini                                            656 ---------------------------------------- 656
93.LC590862.1_NeoscytalidiumdimidiatumPSU-HP01TEF1                     267 ---------------------------------------- 267
94.KX278106.1_BotryosphaeriaqingyuanensisstrainCERC2947                284 ---------------------------------------- 284
```

95.AJ578763.1_Blumeriagraminisf.sp.hordeicyp51	1618	AAGTATGTTTTCTCGGCCAATGGCACCTGCCACAATTGCA	1657
96.MF490858.1_CurvulariadactylocteniicolastrainCPC28810	930	--	930
97.KT287115.1_Bipolariscactivoraisolate3.8.6	1006	--	1006
98.MT560940.1_CurvulariacactivorastrainHLGH0118	963	--	963
99.OM714565.1_CurvulariaplantarumstrainM0134	983	--	983
100.MN159911.1_BotrytiscinereaSICAUCC19-0003	931	--	931
102.GU294713.1LasiodiplodiatheobromaestrainUCD2430TX	407	--	407
103.KX868094.1_Mycosphaerellasp.isolateCRM20.1	475	--	475
104.LC599478.1Pseudocercosporapini-densifloraeMUCC534	310	--	310
105.N584698.1BipolarissetariaestrainKBS4-2	1031	--	1031
2.OM160859.1_F.buharicum	560	---------------------------------------	560
1.LC727524.1_F.buharicum_OKI-1_Okura	682	---------------------------------------	682
3.KX302919.1_F.sublunatum	521	---------------------------------------	521
4.LT996094.1_F.convolutans	516	---------------------------------------	516
5.OM160861.1_F.abutilonis	630	---------------------------------------	630
6.OM160874.1_F.guadeloupense	560	---------------------------------------	560
7.MH392475.1_F.graminearum	430	---------------------------------------	430
8.MH582420.1_F.solani	540	---------------------------------------	540
9.MAFF244605_F.oxysporum	544	---------------------------------------	544
10.MAFF237278_F.contaminatum_Hylocereus	711	---------------------------------------	711
11.MAFF237649_F.concentricum__Riceroot	678	---------------------------------------	678
12.MAFF237650_F.concentricum__Wheat	679	---------------------------------------	679
13.MAFF239869_F.mangiferae__Ryukyupine	657	---------------------------------------	657
14.MAFF240460_F.fujikuroi_Passionfruit	661	---------------------------------------	661
15.MAFF241317_F.graminearum_Wheat	666	---------------------------------------	666
16.MAFF242670_F.ipomoeae_Wheat	672	---------------------------------------	672
17.MAFF245129_F.concentricum_Fraxinus	659	---------------------------------------	659
18.MAFF245395_F.cugenangense_Rhubarb	671	---------------------------------------	671
19.MAFF246637_F.nirenbergiae_Strawberry	671	---------------------------------------	671
20.MAFF246672_F.nirenbergiae_ChinesePeony	671	---------------------------------------	671
21.MAFF246697_F.commune_Urallicoriceroot	673	---------------------------------------	673
22.MAFF246729_F.falciforme_Angelica	691	---------------------------------------	691
23.MAFF247220_F.duplospermum__Euwallaceasp	694	---------------------------------------	694
24.MAFF410760_F.odoratissimum_alpha	669	---------------------------------------	669
25.MAFF244605_FusariumoxysporumSchlechtendal_MAFF244605_Tomato	672	---------------------------------------	672
26.MAFF241326_F.asiaticum_Wheat	669	---------------------------------------	669
27.MAFF245014_F.asiaticum_Wildsoybean	666	---------------------------------------	666
28.MAFF150124_F.asiaticum__Wheat	666	---------------------------------------	666
29.OM135603.1F.algeriense	699	---------------------------------------	699
30.MAFF237465_F.penzigii_Aloe	704	---------------------------------------	704
31.MAFF103054_F.oxysporumSchlechtendalf.sp.cucumerinum_Cucumber	671	---------------------------------------	671
32.MAFF712246_F.oxysporumSchlechtendalf.sp.dianthi__Carnation	673	---------------------------------------	673
33.MAFF305558_F.oxysporumSchlechtendalf.sp.fragariae__Watermelon	671	---------------------------------------	671
34.MAFF744087_F.oxysporumSchlechtendalf.sp.lactucae__Lettuce	671	---------------------------------------	671
35.MAFF726924_F.oxysporumSchlechtendalf.sp.lagenariae_Whitefloweredgourd	671	---------------------------------------	671

```
36.MAFF744003_F.oxysporumSchlechtendalf.sp.lagenariae_Squash)          672 --------------------------------------- 672
37.MAFF305122_F.oxysporumSchlechtendalf.sp.melonis__Melon              671 --------------------------------------- 671
38.MAFF306714_F.oxysporumSchlechtendalf.sp.momordicae_Balsampear       671 --------------------------------------- 671
39.MAFF238905_F.oxysporumSchlechtendalf.sp.radicis-lycopersici_Tomato  674 --------------------------------------- 674
40.MAFF150004_F.oxysporumSchlechtendalf.sp.spinaciae_Spinach           671 --------------------------------------- 671
41.MAFF247034_F.oxysporumSchlechtendal__Goldenchain                    671 --------------------------------------- 671
42.MAFF245747_F.oxysporumSchlechtendalf.sp.callistephi__Chinaaster     671 --------------------------------------- 671
43.MAFF305115_FoxysporumSchlechtendalf.sp.batatas__Sweatpotato         703 --------------------------------------- 703
44.MAFF150126_F.asiaticum_Seed                                         666 --------------------------------------- 666
45.MAFF246738_F.solani_Angelica                                        703 --------------------------------------- 703
46.MAFF246664_F.cugenangense_Perilla                                   671 --------------------------------------- 671
47.MH582420.1F.solanistrainMRC256                                      679 --------------------------------------- 679
48.MAFF240361_F.babinda_Soil                                           693 --------------------------------------- 693
49.MAFF242368_F.azukicola_Azukibean                                    708 --------------------------------------- 708
50.MAFF241312_F.asiaticum_Soil,welshonionfield                         666 --------------------------------------- 666
51.LT548416.1_F.culmorumpartialtef1a                                   646 --------------------------------------- 646
52.MAFF150124_F.asiaticum__Wheat                                       666 --------------------------------------- 666
53.MAFF238806_F.begoniae_Oncidiumsp                                    705 --------------------------------------- 705
54.MW594399.1_FusariumincarnatumisolateUD01C                           689 --------------------------------------- 689
55.OP414923.1Pucciniagraminisf.sp.triticiisolateSHZPgt19               623 --------------------------------------- 623
56.MT027094.1_BipolarisoryzaestrainOrL-2                               895 --------------------------------------- 895
57.ON734360.1_AlternariaalternataisolateH126                           831 --------------------------------------- 831
58.LC333578.1_StemphyliumlycopersiciSOasp2                             265 --------------------------------------- 265
59.HQ718583.1_Colletotrichumgloeosporioidesisolateq-1                  567 --------------------------------------- 567
60.JN241603.1_AtheliarolfsiiisolateSR1                                1013 --------------------------------------- 1013
61.KJ866474.1_RhizoctoniasolanistrainMHL-1                             683 --------------------------------------- 683
62.JQ672424.1AlternariatriticinaisolateEGS17-061                       825 --------------------------------------- 825
63.LT707559.1_P.capsicipartialtef1agene                                964 --------------------------------------- 964
64.MW090051.1_CurvularialunatastrainCls-3                              908 --------------------------------------- 908
65.DQ400892.1_Aspergillusterreus                                       452 --------------------------------------- 452
66.DQ911416.1_Pythiumsp.quercumstrainPy292                             932 --------------------------------------- 932
67.EU797495.1_Phytophthorasp.oaksoilPoland                             930 --------------------------------------- 930
68.HM148321.1_Cladosporiumcucumerinum                                  385 --------------------------------------- 385
69.AF398888.1_SclerotiniasclerotiorumisolateSS1                        334 --------------------------------------- 334
70.AF398888.1_S.sclerotiorumisolateSS1                                 334 --------------------------------------- 334
71.HPAB545908.1_Verticilliumnonalfalfaeisolate                         442 --------------------------------------- 442
72.EF433315.1_CeratocystisfimbriatavoucherCMW15052                     747 --------------------------------------- 747
73.MN159912.1_Botrytiscinerea                                          931 --------------------------------------- 931
74.MF034741.1_PeltasterfructicolaisolateSRB92                          317 --------------------------------------- 317
75.LC440360.1_CercosporaasparagiCOasp2                                 297 --------------------------------------- 297
76.AY944105.1_MagnaportheoryzaeisolateSAG00T3()                        255 --------------------------------------- 255
77.JX266586.1_CochliobolusmiyabeanusvoucherMFLUCC10-0733               918 --------------------------------------- 918
78.MN393253.1_CorynesporacassiicolaisolateQHD001(MN393253.1UNVERIFIED) 305 --------------------------------------- 305
79.MF375218.1_AgroatheliarolfsiiisolateBJB24                           544 --------------------------------------- 544
80.MN106270.1_AgroatheliarolfsiistrainJ-12                             509 --------------------------------------- 509
81.OQ732628.1_AgroatheliarolfsiiisolateBTCBSr3                         499 --------------------------------------- 499
```

```
82.KY196185.1_ColletotrichumtruncatumstrainPAK53          468 --------------------------------------- 468
83.GU935835.1_ColletotrichumcoccodesisolateC96002        1041 --------------------------------------- 1041
84.MK085963.1_AlternariatenuissimaisolateSCCZ06           252 --------------------------------------- 252
85.MT548042.1_AlternarialongipesstrainKY_2019_012         280 --------------------------------------- 280
86.MN356465.1CalonectriamontanaisolateHSP4                489 --------------------------------------- 489
87.OL694224.1_CalonectriacanadianastrainF099              504 --------------------------------------- 504
88.MK803351.1_NeoscytalidiumdimidiatumstrainKale4-C       299 --------------------------------------- 299
89.ON376993.1_CurvulariachiangmaiensisisolateND00J7       938 --------------------------------------- 938
90.OQ383346.1_NeoscytalidiumdimidiatumisolateGKH-2        297 --------------------------------------- 297
91.MF662595.1_NeoscytalidiumnovaehollandiaeisolateNeNo1   284 --------------------------------------- 284
92.EF560588.1Melampsoralini                               656 --------------------------------------- 656
93.LC590862.1_NeoscytalidiumdimidiatumPSU-HP01TEF1        267 --------------------------------------- 267
94.KX278106.1_BotryosphaeriaqingyuanensisstrainCERC2947   284 --------------------------------------- 284
95.AJ578763.1_Blumeriagraminisf.sp.hordeicyp51           1658 TGGGAGAAGAGGGACAAAAAGGACAAAACGGAGTGTTAA 1696
96.MF490858.1_CurvulariadactylocteniicolastrainCPC28810   930 --------------------------------------- 930
97.KT287115.1_Bipolariscactivoraisolate3.8.6             1006 --------------------------------------- 1006
98.MT560940.1_CurvulariacactivorastrainHLGH0118           963 --------------------------------------- 963
99.OM714565.1_CurvulariaplantarumstrainM0134              983 --------------------------------------- 983
100.MN159911.1_BotrytiscinereaSICAUCC19-0003              931 --------------------------------------- 931
102.GU294713.1LasiodiplodiatheobromaestrainUCD2430TX      407 --------------------------------------- 407
103.KX868094.1_Mycosphaerellasp.isolateCRM20.1            475 --------------------------------------- 475
104.LC599478.1Pseudocercosporapini-densifloraeMUCC534     310 --------------------------------------- 310
105.N584698.1BipolarissetariaestrainKBS4-2               1031 --------------------------------------- 1031
```
