# Supplementary material for: Development of a PCR-based assay for specific and sensitive detection of Fusarium buharicum from infected okra plant
Source: PLoS One. 2024 Apr 16;19(4):e0302256. doi: 10.1371/journal.pone.0302256 (PMC11020393; doi:10.1371/journal.pone.0302256)
Supplement: S2 Fig — The sequences were obtained from NCBI Genbank nucleotide databases and aligned by using Multiple Sequence Alignment software CLUSTALW. The blue boxes indicate the F. buharicum species-specific primers (EF1Fb-F1 and EF1Fb-R5) sequences. (PDF) [file pone.0302256.s002.pdf]

**S2 Fig.**

```
SsHAY30         TTGCCGCCGGTACTGGTGAGTTCGAGGCTGGTATCTCCAAGGATGGCCAAACTCGTGAGC
SvPHB17         ----------------------------TGGTATCTCCAAGGATGGTCAGACTCGTGAGC
FbOKI-1         GCCACGTCGACTCTGGCAAGTCGACCACTGTGAGTACTACCCTCGACGGTGTGCTTGCTT
                                            **  *   * *     *        * **

SsHAY30         ACGCTCTTCTTGCCTACACCC---TTGGTGTTAAGCAACTCATCGTTGCCATCAACAAGA
SvPHB17         ACGCTCTCCTCGCCTACACCC---TCGGTGTCAAGCAGCTCATCGTTGCCATCAACAAGA
FbOKI-1         GCACTCGTCAAACCTCCATCCAGATTTCTGGCGAGGTTTTCATCATTTATATCATGCTGA
                 * ***  *   *** ** **   *   **   **    ***** **   ****    **

SsHAY30         TGG----ACACCACCAAGTGGTCTGAGG-AACGTTACCAAGAAATTATCAAGGAGACCTC
SvPHB17         TGG----ACACCACCAAGTGGTCTGAGG-CCCGTTTCTCCGAGATTATCAAGGAGACCTC
FbOKI-1         CATTTTTATACAGACCGGTCACTTGATCTACCAGTGCGGTGGTATCGACAAGCGAACCAT
                       * **   *  **    ***     *  * *   *  **   ****   ***

SsHAY30         CAACTTCATCAAGAAGGTCGGATACAACCCAAAGACCGTTCCTTTCGTTCCTATCTCCGG
SvPHB17         CAACTTCATCAAGAAGGTCGGCTACAACCCCAAGCACGTCCCCTTCGTGCCCATCTCCGG
FbOKI-1         CGAGAAGTTCGAGAAGGTTGGTTATTTCCCTTCGATCGCGCCCTT-ATGCCCATC----G
                * *     ** ******* ** **   ***   *  **  ** **  * ** ***    *

SsHAY30         TTTCAACGGTGATAACATGATCGACAACTCCACCAACTGCCCATGGTACAAGGGTTGGGA
SvPHB17         TTTCAACGGTGACAACATGATCGAGGCCTCCACCAACTGCCCCTGGTACAAGGGCTGGGA
FbOKI-1         ATTTGCCCGTCGAATCGCTCCCTCCGCGACTCGCAACGCGCCGT--TACCCCGCTCGAGC
                 **   * **   * *     *       *   ****   ** *  ***   *   * *

SsHAY30         GAAGGAGGCCAAGGGAGGAGCCAAGTCCACCGGAAAGACCCTTCTCGAGGCTATTGATGC
SvPHB17         GAAGGAGACCAAGG------CCAAGGCCACCGGTAAGACCCTTCTCGAGGCCATCGACGC
FbOKI-1         ACAAAAATTTTGCG----------GTGCGACCGTAATTTTTTTGGTGGGGCATTTTACCC
                  *  *       *          *  *  * * **     **   * ***  *  *  *

SsHAY30         CATCGACCCA-CCTTCCCGCCCAACTGACAAGCCCCTCCGT-CTTCCCCTCCAAGATGTT
SvPHB17         CATCGACCCC-CCCACACGTCCTACCGACAAGCCCCTCCGC-CTTCCCCTTCAGGATGTC
FbOKI-1         CGCCACTCGAGCTTGGCCGCGCAATGCGCTGTTCCCTGCACACATAATCACTTAGGCGCT
                *  *   *   *     **  * *    *    **** *   * *   *     *  *

SsHAY30         TACAAGATTGGTGGTATTGGC-ACGGTGCCAGTCGGTCGT-GTCGAGACCGG--------
SvPHB17         TACAAGATTGGCGGTATTGGC-ACGGTTCCCGTCGGTCGT-GTCGAGACTGGTATCATCA
FbOKI-1         CGTCATGTGACAGTTGCTGACCACCTCGACAATAGGAAGCCGCCGAGCTCGGTAAGGGTT
                    *  *    * *  ** * **     *  * **  *  * ****   **
```
