# Supplementary figures and images for: Development of a PCR-based assay for specific and sensitive detection of Fusarium buharicum from infected okra plant
Source: PLoS One. 2024 Apr 16;19(4):e0302256. doi: 10.1371/journal.pone.0302256 (PMC11020393; doi:10.1371/journal.pone.0302256)

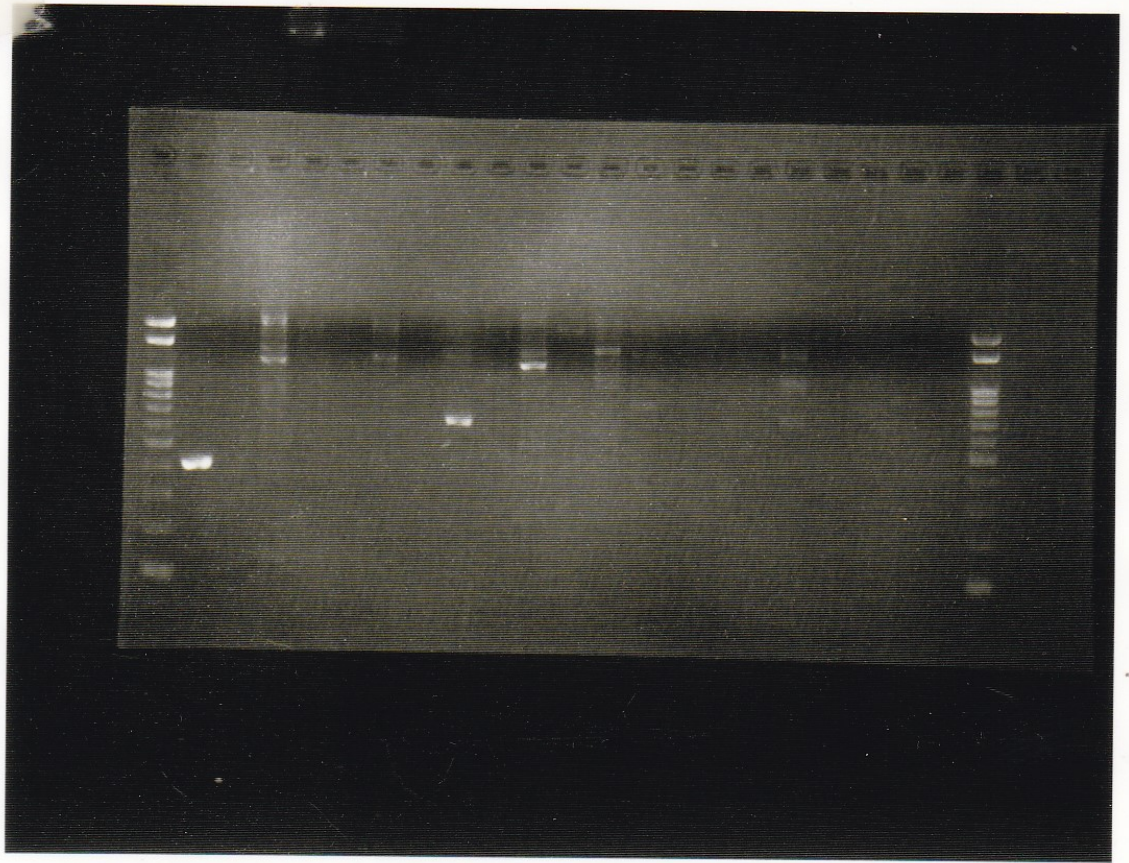

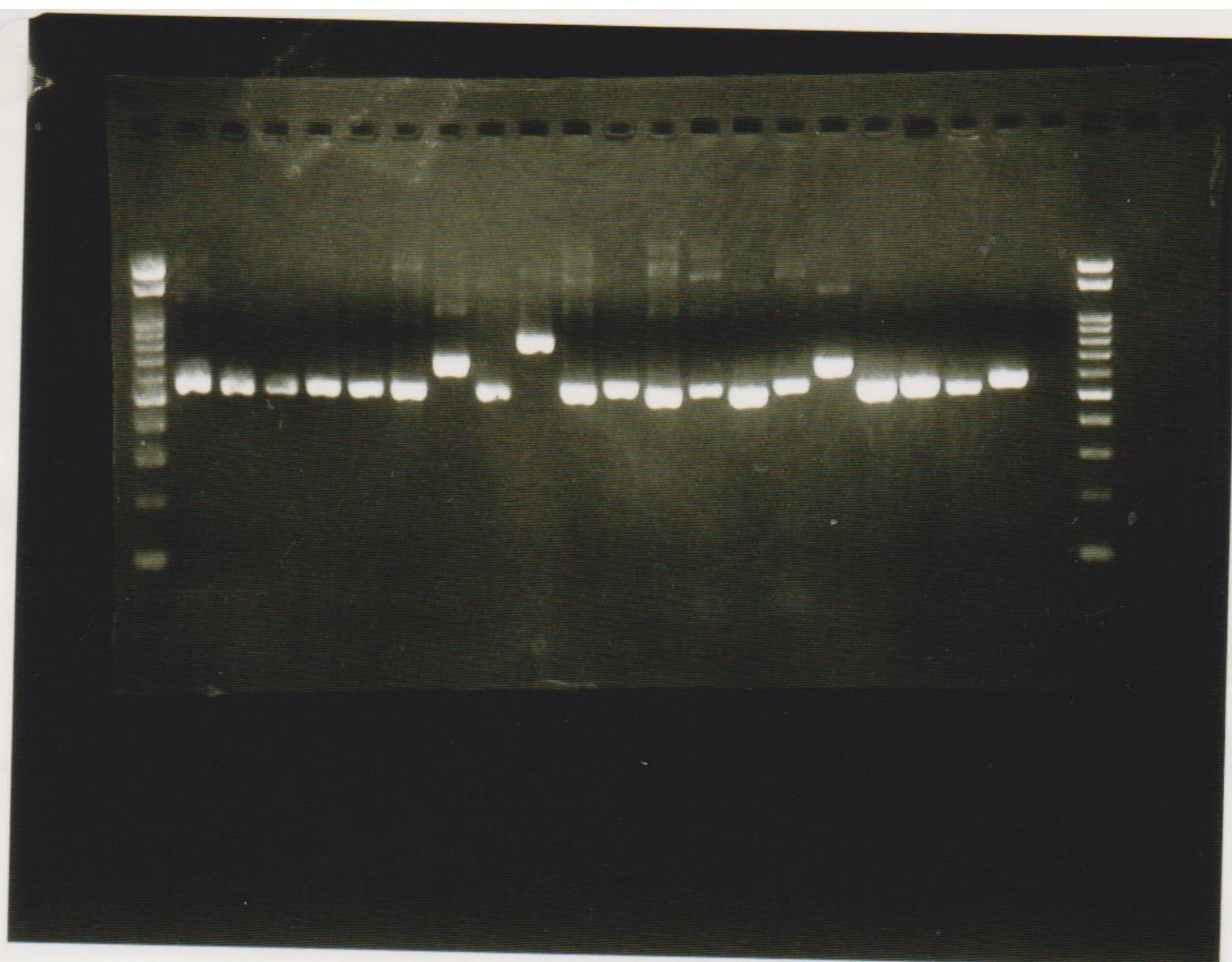

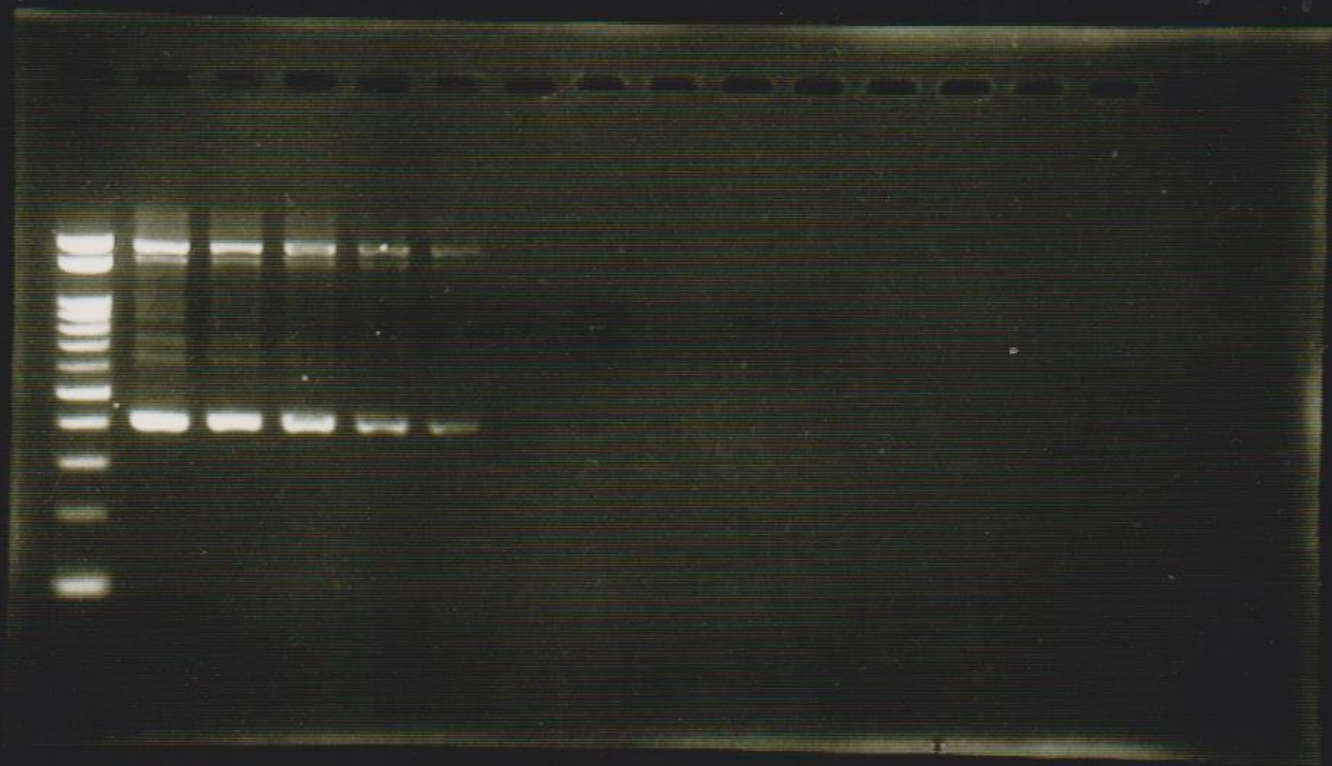

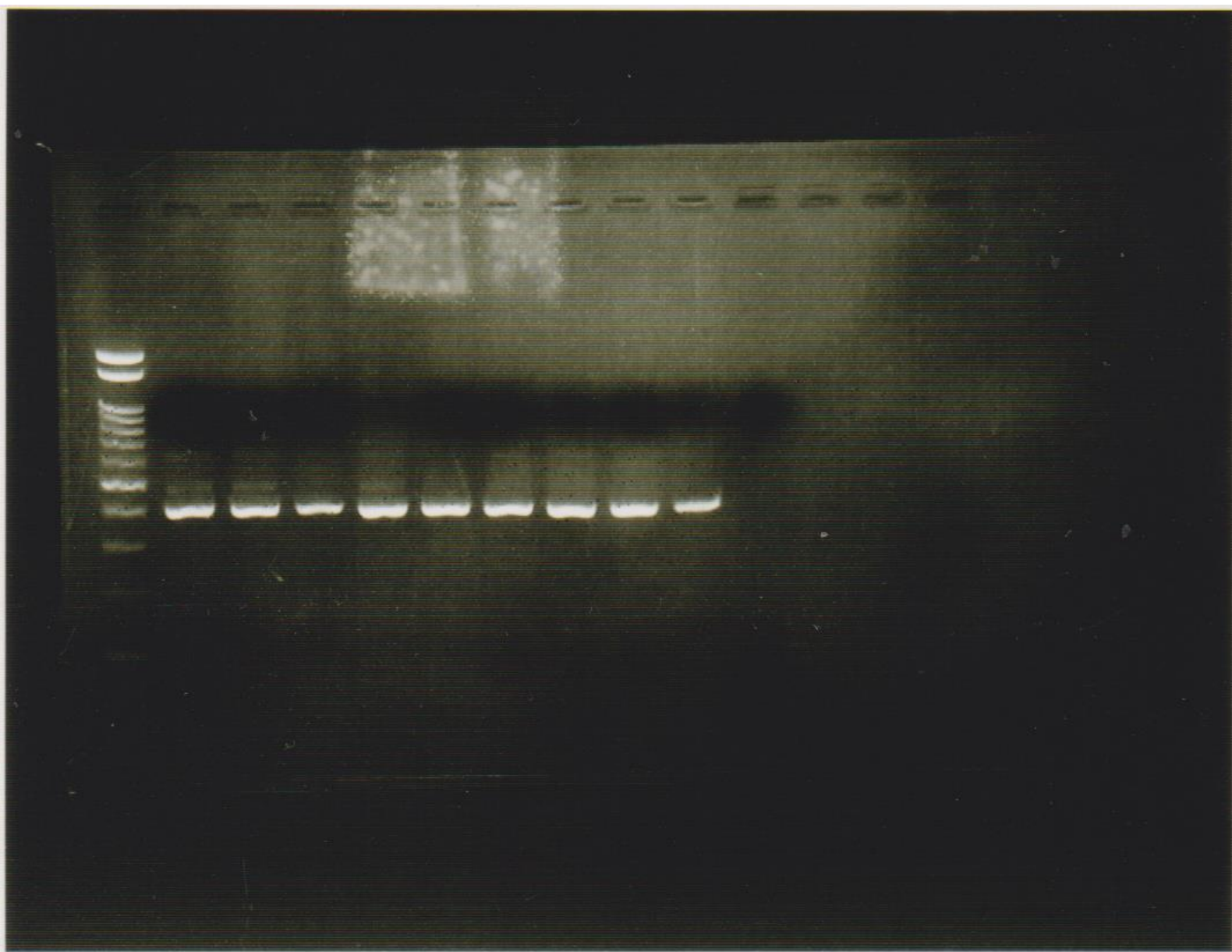

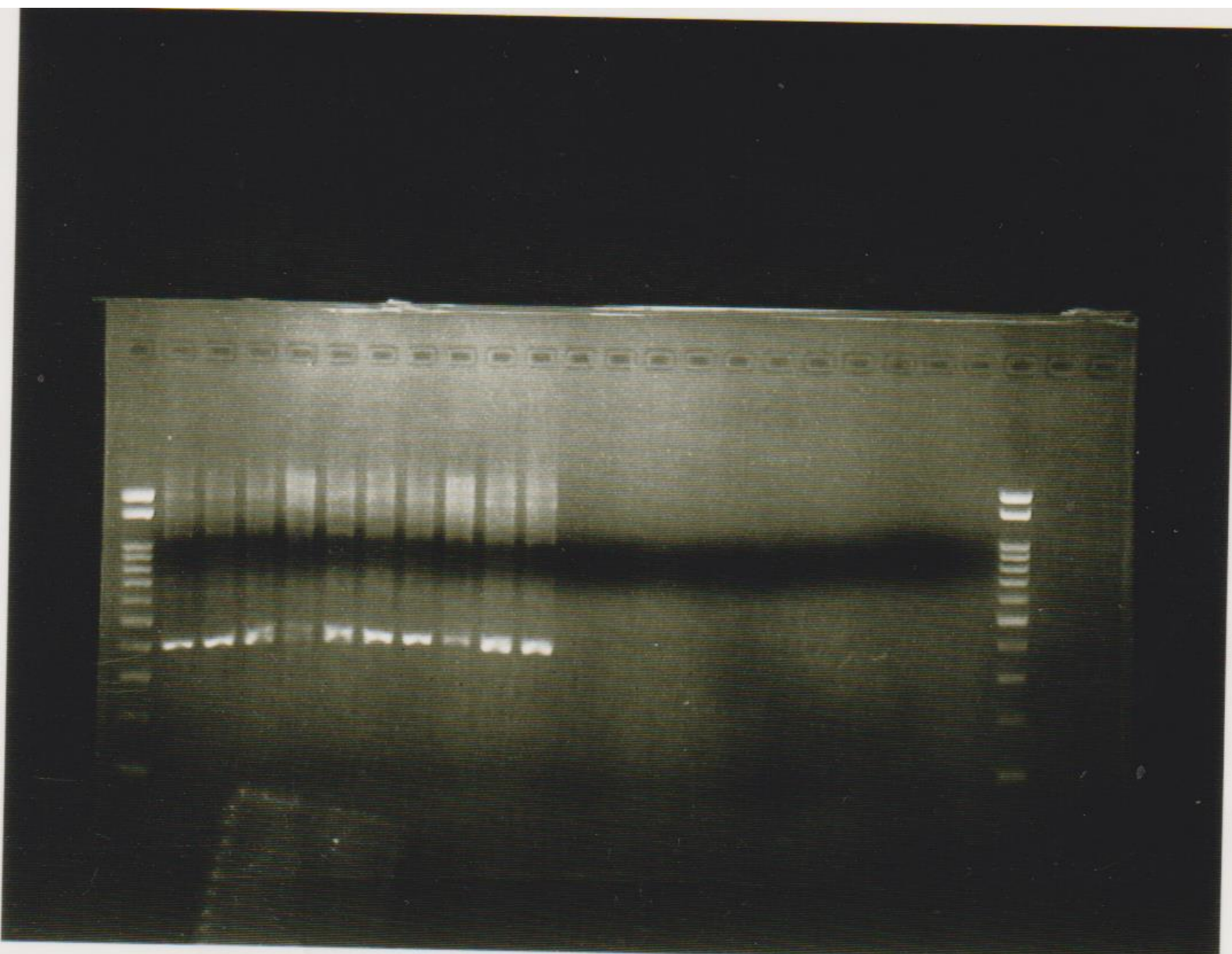

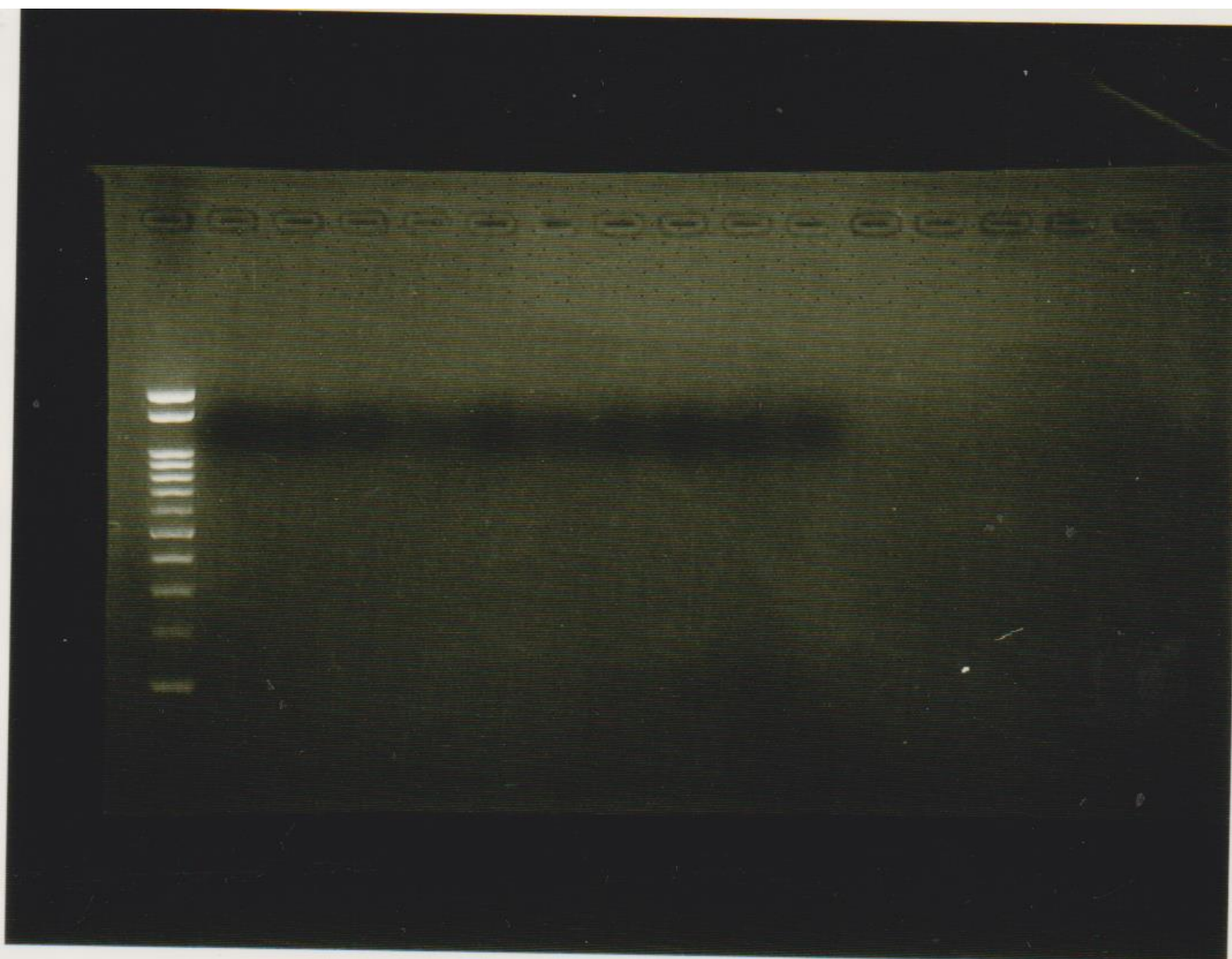

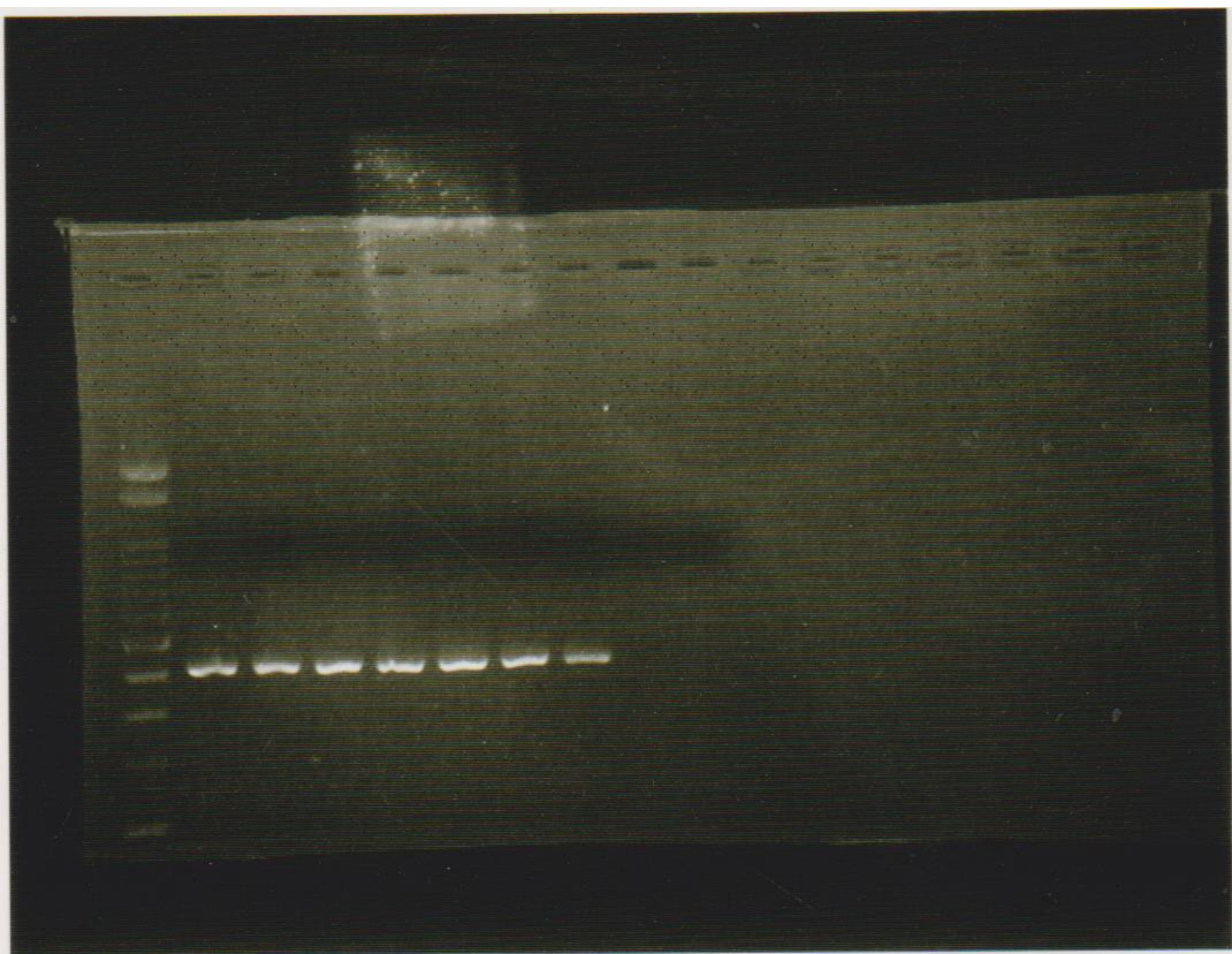

Supplement: S1 Raw images — (PDF) [file pone.0302256.s003.pdf]
